# The Structure of Adamantane Clusters: Atomistic vs. Coarse-Grained Predictions From Global Optimization

**DOI:** 10.3389/fchem.2019.00573

**Published:** 2019-08-16

**Authors:** Javier Hernández-Rojas, Florent Calvo

**Affiliations:** ^1^Departamento de Física e IUdEA, Universidad de La Laguna, San Cristóbal de La Laguna, Spain; ^2^Univ. Grenoble Alpes, CNRS, LIPhy, Grenoble, France

**Keywords:** global optimization, coarse-grained (CG) model, molecular clusters, potential energy surface (PES), all-atom computer simulations

## Abstract

Candidate structures for the global minima of adamantane clusters, (C_10_H_16_)_*N*_, are presented. Based on a rigid model for individual molecules with atom-atom pairwise interactions that include Lennard-Jones and Coulomb contributions, low-energy structures were obtained up to *N* = 42 using the basin-hopping method. The results indicate that adamantane clusters initially grow accordingly with an icosahedral packing scheme, followed above *N* = 14 by a structural transition toward face-centered cubic structures. The special stabilities obtained at *N* = 13, 19, and 38 are consistent with these two structural families, and agree with recent mass spectrometry measurements on cationic adamantane clusters. Coarse-graining the intermolecular potential by averaging over all possible orientations only partially confirm the all-atom results, the magic numbers at 13 and 38 being preserved. However, the details near the structural transition are not captured well, because despite their high symmetry the adamantane molecules are still rather anisotropic.

## 1. Introduction

Global optimization is an important topic in the physical and chemical sciences, whether we want to refine a force field, predict the native structure of a protein or the crystal structure of some condensed material, or find a practical solution to machine learning problems (Andricioaei and Straub, 1996; Huber and McCammon, 1997; Doye and Wales, 1998; Wales and Hodges, 1998; Wawak et al., 1998; Klepeis and Floudas, 1999; Liwo et al., 1999; Nigra and Kais, 1999; Wales and Scheraga, 1999; Middleton et al., 2001; Hernández-Rojas and Wales, 2003; James et al., 2005; Fadda and Fadda, 2010; Heiles and Johnston, 2013; Wu et al., 2014; Ballard et al., 2016, 2017; Das and Wales, 2016). The case of atomic and molecular clusters is enlightening because such systems exhibit strong finite-size effects, with lowest-energy structures that can depend sensitively and non-monotonically with increasing number of constituents (Stillinger and Weber, 1982, 1984; Tsai and Jordan, 1993b). In particular, efficient global optimization algorithms should be able to explore complex energy landscapes with hierarchical or multifunnel character (Dittes, 1996; Nymeyer et al., 1998; Hamacher and Wenzel, 1999; Wenzel and Hamacher, 1999; Xu and Berne, 1999; Stolovitzky and Berne, 2000; Goedecker, 2004; Cheng et al., 2009; Wang et al., 2010; Oakley et al., 2013).

The difficulties in practically solving the global optimization problem for atomic and molecular systems are at least 2-fold. Firstly, the number of available local minima is thought to increase exponentially with size, making systematic enumeration virtually impossible already above a few tens of particles (Hartke et al., 1998; Wales and Hodges, 1998; Nigra and Kais, 1999; Hodges and Wales, 2000; James et al., 2005; Hernández-Rojas et al., 2006, 2016; Hernández-Rojas and Wales, 2014; Bartolomei et al., 2017). Tsai and Jordan thus evaluated that the 147-atom Lennard-Jones cluster could have more than 10^60^ minima (Tsai and Jordan, 1993a). Secondly, the various structural families generally form different funnels in the landscape separated by high energy barriers, making the sampling problem particularly severe, with conventional simulation methods such as basic molecular dynamics or Monte Carlo, even supplemented with simulated annealing protocols, simply unsuccessful (Wales, 2003).

One additional difficulty arises in molecular systems, even described as rigid bodies, because of the interplay between translational and orientational degrees of freedom. In some cases, the molecules themselves are such that they impose drastic constraints on the collective arrangements that can be adopted by the clusters, starting with the dimer. This occurs, e.g., for planar polycyclic aromatic hydrocarbons (PAHs), which tend to assemble into columnar motifs (Rapacioli et al., 2006; Hernández-Rojas et al., 2016; Bartolomei et al., 2017), or conversely for rodlike molecules, such as CO_2_ (Maillet et al., 1998). Even for molecules as relatively simple as water, for which the interactions would seem fairly well-known, water cluster structures are notoriously non-trivial due to the importance and anisotropy of the hydrogen bond (Hartke et al., 1998; Wales and Hodges, 1998; Nigra and Kais, 1999; Hodges and Wales, 2000; James et al., 2005).

In the present work we are interested in clusters of the adamantane molecule (C_10_H_16_). Adamantane is a small hydrocarbon molecule with pure sp^3^ hybridized carbon atoms arranged in a tetrahedral point group, often referred to as a diamondoid. It has a very high thermal stability, and could be found in deep petroleum sources (Dahl et al., 1999, 2010) as well as astrophysical media (Blake et al., 1988; Allamandola et al., 1993; Bauschlicher et al., 2007; Pirali et al., 2008; Steglich et al., 2011). The adamantane molecule is also involved in alkane chemistry (Fokin and Schreiner, 2002), is a versatile building block for larger supramolecular assemblies (Tominaga et al., 2014; Pichierri, 2018) and was found to have some interesting potential in nanomedicine after functionalization (Grillaud et al., 2014; Spilovska et al., 2016; Lee et al., 2018), or even as wheels of nanocars (Chu et al., 2013).

Adamantane clusters were recently synthesized in the cryogenic environment of helium nanodroplets, in which they could be size-selected after ionization by an electron gun (Goulart et al., 2016). In a first approximation, adamantane is roughly spherical and interacts with other molecules via non-covalent forces of the dispersion-repulsion type, with additional Coulomb contributions arising from the partial charges carried by the hydrogen and carbon atoms having different electronegativities. No particular electron delocalization is expected between different molecules, although in the cationic clusters some polarization effects are obviously expected.

So far, the structure of adamantane clusters has not been characterized at the atomistic level of details, but indirect structural information could be drawn from the experimental mass spectra, which show special abundances at the sizes of 13, 19, and 38 molecules. While the former two magic numbers are compatible with icosahedral arrangements, the latter is strongly indicative of a close-packed face-centered cubic structure, suggesting a size-induced structural transition taking place above only a few tens of molecules. Icosahedral-to-cubic transitions are common in atomic and molecular clusters, as they convey the increasing energetic penalty that the highly connected icosahedral structures have to sustain, eventually in favor of less connected but also less strained close-packed structures (Doye et al., 1995; Ikeshoji et al., 2001; Calvo and Carré, 2006). Such a transition has been identified as being strongly influenced by the range of the interparticle potential (Doye et al., 1995; Doye and Wales, 1996). In the present case of adamantane, which has a significant molecular extension while the dispersion interaction is comparatively short-ranged, close packing thus seems natural.

However, the experimental magic numbers do not provide any insight into the orientational ordering within the clusters, and in particular whether the tetrahedral symmetry plays any role on the structures. In order to shed some light onto the relative importance of the translational and orientational degrees of freedom and their interplay, and more generally to confirm whether adamantane clusters do indeed correspond to the speculated structures, we have carried out a systematic global optimization investigation in the size range up to 42 molecules, using the basin-hoping method as our main tool. Two complementary strategies have been employed, namely an all-atom (AA) approach based on a rigid body description, and a highly simplified, coarse-grained (CG) model averaging over all possible orientations.

At the all-atom level, our calculations predict that adamantane clusters are most stable as icosahedra until 14 molecules are reached, and above which the structural arrangement becomes close packed. The special stabilities in the mass spectra are reproduced by the second-energy difference in our all-atom model. At the coarse-grained level, differences appear already above six molecules, although both the icosahedral and cubic motifs at sizes 13 and 38 are correctly reproduced. Comparison between the two models confirms the important role played by the orientational degrees of freedom, despite adamantane being of a rather high symmetry, and shows that the close-packed structures are ideally composed of planes with alternating molecular orientations, a feature that the coarse-grained model is obviously unable to capture.

This paper is organized as follow. We present the potential energy surfaces in section 2 and the methodology employed in the global optimization in section 3. The results are discussed in section 4, and we summarize our conclusions in section 5.

## 2. Potential Energy Surfaces

Complete global optimization using an explicit description of electronic structure is unfeasible for systems containing hundreds or thousands of atoms, which furthermore can adopt many nearly degenerate local minima. For the present system, and using the model described just below, more than 20 local minima are found just for the adamantane dimer within 2 kJ/mol of the putative global minimum. Moreover, the interactions between neutral adamantane molecules are essentially non-covalent in nature, a notorious issue in quantum chemistry dealing with large molecules. However, the closed-shell electronic structure of the adamantane molecule makes classical force fields particularly attractive for modeling the potential energy surface. A primary assumption usually made at low temperatures relevant for cryogenic environments is to treat the molecules as rigid bodies, with all vibrations frozen. In this work, two models were considered for the interactions between adamantane molecules.

### 2.1. All-Atom Model

Following the traditional approach of classical force fields, we assume that adamantane molecules interact with each other through a sum of pairwise forces comprising repulsion-dispersion and Coulomb contributions. The interaction *V*_*ab*_ between two rigid adamantane molecules *a* and *b* is thus expressed by a Lennard-Jones (LJ) part applied between all atoms from *a* and *b*, plus electrostatic interactions between partial charges originating from the electronegativity difference between carbon and hydrogen atoms:

(1) $$\begin{array}{r} V_{ab}=\underset{i,j>i}{\sum }\left\{4\varepsilon_{ij}\left[{\left(\frac{\sigma_{ij}}{r_{ij}}\right)}^{12}-{\left(\frac{\sigma_{ij}}{r_{ij}}\right)}^{6}\right]+\frac{q_{i}q_{j}e^{2}}{4\pi \varepsilon_{0}r_{ij}}\right\}, \end{array}$$

where *q*_*i*_ and *q*_*j*_ are the partial charges on site *i* of molecule *a* and site *j* of molecule *b*, respectively, *r*_*ij*_ is the Cartesian separation between the two sites. In the above expression, all sums were implicitly assumed to be between atoms from different molecules: no intramolecular potential acts for such rigid molecules.

The LJ parameters between sp^3^ carbon and hydrogen atoms are taken from the popular OPLS force field (Jorgensen et al., 1996), and read ε_CC_ = 0.458 kJ/mol, ε_HH_ = 0.066 kJ/mol, σ_CC_ = 3.4 Å, σ_HH_ = 2.649 Å, Lorentz-Berthelot combination rules providing the complementary values for C-H interactions. The partial charges on individual atoms and the equilibrium geometry of isolated adamantane were obtained from a quantum chemical calculation at the DFT/M06-2X/6-311G(d,p) level of theory. They read *q*_C_ = −0.71 and *q*_H_ = +0.14 for carbon and hydrogen atoms in CH_2_ groups, and *q*_C_ = +0.70 and *q*_H_ = −0.055 for carbon and hydrogen atoms in CH groups, in units of the electron charge magnitude.

### 2.2. Comparison With Electronic Structure Calculations

To assess the accuracy and relevance of our simple force field, we have performed dedicated quantum chemical calculations for the adamantane dimer using various levels of theory. Density-functional theory (DFT) is probably the most practical method to deal with such molecules, and here we have chosen the modern functionals PBE0 (Adamo and Barone, 1999), wB97xD (Chai and Head-Gordon, 2008), and M06-2X (Zhao and Truhlar, 2008) as implemented in the Gaussian09 software package (Frisch et al., 2016). While PBE0 does not include explicit dispersion corrections, it performs very well for multipolar descriptions. Both wB97xD and M06-2X are expected to describe non-covalent interactions satisfactorily. Perturbation theory was also employed, using the spin-component-scaled method SCS-MP2 (Grimme, 2003) with basis set superposition errors accounted for using the counterpoise method, as implemented in NWCHEM (Valiev et al., 2010). For these four methods, the two basis sets 6-311G(d,p) and aug-cc-pvDZ were employed independently.

From the resulting geometries, the basic geometric properties of distance *r* between centers of mass and relative orientations measured by the orientational order parameter κ, as defined below in section 3.2, were evaluated. The interaction energy *E*_int_ was also determined from the total energies of the optimized monomer and dimer using the standard equation

(2) $$\begin{array}{r} E_{\text{int}}=E_{\text{dimer}}-2E_{\text{monomer}}, \end{array}$$

and the resulting values for *r*, κ and *E*_int_ are given in Table 1.

**Table 1:** Interaction energy and geometric properties of the adamantane dimer, as predicted by different quantum chemical methods and by the present empirical potential.

**Method**	***E*_int_ (kJ/mol)**	**r (Å)**	**κ**
DFT/PBE0/6-311G(d,p)	−5.19	6.83	−0.31
DFT/PBE0/aug-cc-pvDZ	−7.70	6.59	−0.33
DFT/wB97xD/6-311G(d,p)	−21.33	6.05	−0.25
DFT/wb97xD/aug-cc-pvDZ	−24.72	6.02	−0.26
DFT/M06-2X/6-311G(d,p)	−12.97	6.14	−0.27
DFT/M06-2X/aug-cc-pvDZ	−17.66	6.07	−0.27
SCS-MP2/6-311G(d,p)	−9.97	6.26	−0.31
SCS-MP2/aug-cc-pvDZ	−8.81	6.66	−0.26
Force field	−14.92	6.22	−0.36

*The orientational order parameter κ is defined in section 3.2*.

Unsurprisingly, we find a significant spreading among the DFT results, with a marked dependence of the interaction energy on the functional used, and notably a factor >4 between PBE0 and wB97xD results, SCS-MP2 and M06-2X data lying in-between those extremes. The weaker binding predicted by PBE0 is consistent with this functional not properly accounting for dispersion interactions. Basis set effects further contribute to some variations, although with one magnitude lower. The strong differences between the predictions of PBE0 and wB97xD are comparable to those obtained earlier in other intermolecular interactions problem involving fullerenes and hydrogen (Kaiser et al., 2013; Calvo et al., 2018b).

The force field based on OPLS with multipolar contributions obtained from partial charges derived from DFT performs very satisfactorily against the not-so-extreme quantum chemical predictions from M06-2X and SCS-MP2 both in terms of energy and geometry. The good performance of the force field against the Minnesotta functional M06-2X is also consistent with an earlier study on microhydrated RNA precursors (Bacchus-Montabonel and Calvo, 2015) where this quantum chemical method was found to perform better than MP2 against coupled cluster reference data. Together with the difficulty of obtaining more accurate electronic structure properties for the present 52-atom dimer system, these results indicate that our simple model is chemically reliable.

### 2.3. Coarse-Grained Model

The high symmetry of adamantane encourages us to attempt a simplified description based on a coarse-grained model of the previous all-atom potential. Such an approach has been highly successful in the past for isotropic molecules such as C_60_, for which simple analytical expressions can be obtained for the integrals (Girifalco, 1991). Here we consider a spherical pointlike version, in which the effective potential is obtained by spherical averaging over the relative orientations of the two molecules, at fixed distance between their centers of mass. Averaging was performed using a random sampling procedure employing 10^6^ independent orientational configurations.

**Figure 1:**
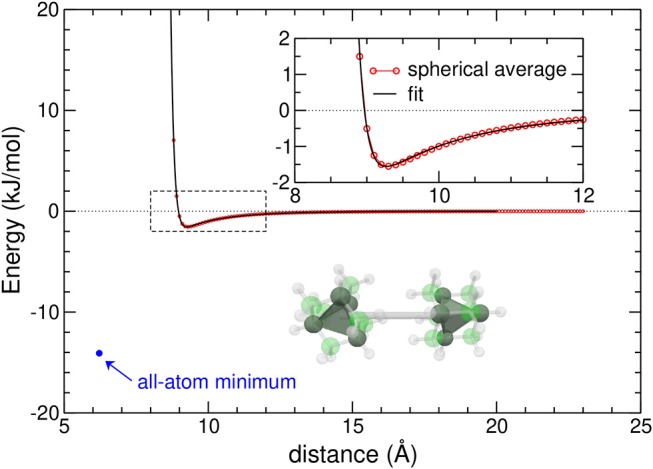
Spherically averaged potential energy curve of the adamantane dimer (red circles) and its best fit giving the coarse-grained potential (black line). The minimum energy in the all-atom model is shown as a blue circle, and the inset highlights the distance range where the potential is minimum. The tetrahedral symmetry of adamantane molecules in the dimer geometry is also shown.

In Figure 1, the variations of the CG potential are represented against increasing distance, together with the geometry of the equilibrium adamantane dimer obtained at the AA level.

The energy and equilibrium position in the AA model, also highlighted in the figure, show that the CG model underestimates the binding energy by about one order of magnitude, owing to the strong repulsion between peripheral hydrogen atoms, and presents an equilibrium position at a larger distance. The effective potential is very steep, as also expected for an interaction between sizeable molecules. It has thus an effectively short range, which should favor close packing (Doye et al., 1995).

The CG potential can be fitted into a simple expression only dependent on the interparticle distance *r* as

(3) $$\begin{array}{r} {Ṽ}_{ab}=A\exp\left[-\alpha \left(r-r_{0}\right)\right]-f_{\text{cut}}\left(\frac{C_{6}}{r^{6}}+\frac{C_{8}}{r^{8}}\right), \end{array}$$

with a short-range cut-off function *f*_cut_ that reads

(4) $$f_{\text{cut}}=\;\{\begin{array}{ll} \exp\left[-{\left(1-d/r\right)}^{2}\right] & \text{if}\;r<d\\ 1 & \text{if}\;r\geq d \end{array}$$

The optimal parameters of the CG potential were found to be *A* = 0.0468 kJ/mol, α = 8.86 Å^−1^, *r*_0_ = 9.405 Å, *C*_6_ = 423040.5 kJ· mol^−1^·Å^6^, *C*_8_ = 56522581.1 kJ· mol^−1^·Å^8^, *d* = 8 Å.

## 3. Global Optimization

The global energy minima were located using the basin-hopping (BH) or Monte Carlo plus minimization method (Li and Scheraga, 1987; Wales and Doye, 1997). The implementation of BH for adamantane clusters differs for the AA and CG potentials due to the presence of orientational degrees of freedom for the former.

### 3.1. Survey by Basin-Hopping

Basin-hopping is a stochastic algorithm that transforms the PES into a collection of basins of attraction and explore them by random large amplitude, collective moves between minima. This transformed PES preserves all local minima, including the global minima, and the search proceeds by successive applications of the Monte Carlo Metropolis acceptance rule to the locally minimized energies. The BH method has been successfully applied to a plethora of atomic and molecular clusters in the past (Wales et al., 2000; Wales, 2003).

For the CG potential, only translational moves have to be considered, and several series of 10^5^ local minimizations were carried out for each cluster size, the fictitious temperature parameter being set such as *k*_B_*T* = 0.5 kJ/mol. No strong influence of this parameter was found here.

For the AA model, the translational and rotational moves can be either managed on a similar footing, or distinguished from one another. In the most general version, a random move thus consists of perturbing all positions of the centers of mass and rotating the molecules, both displacements being performed simultaneously before local minimization is carried out. Here we have chosen to represent the orientational degrees of freedom using angle-axis coordinates $\vec{k}=\left(n,\ell ,m\right)$, a vector that defines a rotation axis passing through the center of mass and with magnitude of the rotation given by $\theta =\sqrt{n^{2}+\ell^{2}+m^{2}}$, relative to a fixed reference frame. This angle-axis representation provides a general framework for rigid body isotropic site-site potentials (Wales, 2005; Chakrabarti and Wales, 2009). The advantage of angle-axis coordinates is that they do not suffer from the so-called gimbal lock problem appearing with Euler angles when rotational axes can become equivalent. Using this framework, the orientational moves consist of perturbing all components of the angle-axis vector producing a new orientation, $\vec{k^{\prime }}=\left(n^{\prime },\ell^{\prime },m^{\prime }\right)$, but with the constraint that the new angle $\theta^{\prime }=\sqrt{n^{\prime 2}+\ell^{\prime 2}+m^{\prime 2}}$ remains between 0 and 2π.

Test runs performed for the 12-molecule cluster and employing 5 × 10^4^ BH steps allowed us to evaluate suitable parameters for the basin-hopping optimizations with the AA model, namely *k*_B_*T* = 1.5 kJ/mol, giving an acceptance ratio of about 20%. Unfortunately, above size 21 the algorithm was found less efficient, and lower-energy structures could be occasionally found simply by removing molecules from neighboring size clusters and conducting short BH runs. We thus implemented an alternative strategy in which the centers of mass positions were borrowed from the CG minima, purely orientational moves being allowed in the subsequent BH minimization. Here only 10^4^ BH collective steps were performed for each cluster size.

The results reported below are thus the results of three combined approaches relying on basin-hopping but altering the entire set of degrees of freedom, only the orientations, or exploring the random removal of one molecule followed by further local search. The orientational minimization was also used to produce all-atom clusters with a specific translational ordering but lying in a different funnel as the global minimum. In practice it allowed us to generate icosahedral and cubic clusters in a broader size range, providing further insight into the related structural transition. In all our BH searches, the geometry was reset to the local minimum before a random perturbation was attempted again.

### 3.2. Structural Indicators

For the analysis of cluster minima, different order parameters and structural indicators were considered to probe the extent of translational and orientational orderings. The bond-orientational order parameter *Q*_6_ involves the relative positions of the molecular centers of mass and is useful to discriminate icosahedral and cubic packings (Calvo et al., 2018a). It is defined as

(5) $$\begin{array}{r} Q_{6}={\left(\frac{4\pi }{13}\overset{m=6}{\underset{m=-6}{\sum }}|{\bar{Q}}_{6m}{|}^{2}\right)}^{1/2}, \end{array}$$

where

(6) $$\begin{array}{r} {\bar{Q}}_{6m}=\frac{1}{N_{b}}\underset{r_{ij}<7.5\;\text{Å}}{\sum }Y_{6m}\left(\theta_{ij},\phi_{ij}\right), \end{array}$$

*N*_*b*_ being the number of bonds defined when the distance between of center of masses of two adamantane molecules is lower than 7.5 Å. *Y*_6*m*_(θ_*ij*_, ϕ_*ij*_) is the spherical harmonic function of degree 6 and order *m*. The *Q*_6_ parameter can be evaluated for both the AA and CG structures.

An orientational order parameter respecting the tetrahedral symmetry of adamantane was constructed to measure the extent of alignment within the clusters. More precisely, and following Fel (Fel, 1995), for each molecule *a* we associate four unit vectors ${\vec{n}}_{k}^{\left(a\right)}$ pointing along the four tetrahedral directions, with Cartesian coordinates $n_{k,\alpha }^{\left(a\right)}$ with α = *x*, *y*, and *z*. From these coordinates a 3-rank tensor $Q_{3}^{\left(a\right)}$ is constructed as

(7) $$\begin{array}{r} Q_{3,\alpha ,\beta ,\gamma }^{\left(a\right)}=\overset{4}{\underset{k=1}{\sum }}n_{k\alpha }^{\left(a\right)}n_{k\beta }^{\left(a\right)}n_{k\gamma }^{\left(a\right)}. \end{array}$$

For a set of molecules, an orientational order parameter κ that is tetrahedrally invariant is defined by considering the pairs of nearest-neighbor molecules as

(8) $$\begin{array}{r} \kappa =\frac{9}{32n_{nn}}\underset{a<b,r_{ab}<7.5\;\text{Å}}{\sum }\text{Tr }Q_{3}^{\left(a\right)}Q_{3}^{\left(b\right)}, \end{array}$$

where *n*_*nn*_ is the number of nearest-neighbor molecules. The prefactor ensures that κ = 1 if all molecules are tetrahedrally aligned.

In addition to purely geometric indicators, energetic parameters were also evaluated to measure the relative stability of the clusters, and quantify the role of orientational strain (*vide infra*).

## 4. Results

The putative global minima of adamantane clusters were obtained with full atomistic details up to size 42. All structures are available in the Supplementary Material. The much less expensive coarse-grained model was able to provide reliable structures in a significantly broader range, although the trends above 42 remain essentially unchanged and will not be discussed specifically.

### 4.1. Energetic Stability

To estimate the relative stability of different cluster sizes, we evaluated the second-energy derivative of the PES, $\Delta^{2}E\left(N\right)=E_{N+1}+E_{N-1}-2E_{N}$, where *E*_*N*_ is the energy of the global minimum for (C_10_H_16_)_*N*_. Maxima in Δ^2^ correspond to clusters with enhanced stability, and are thus closely related to special abundances experimentally measured by mass spectrometry.

**Figure 2:**
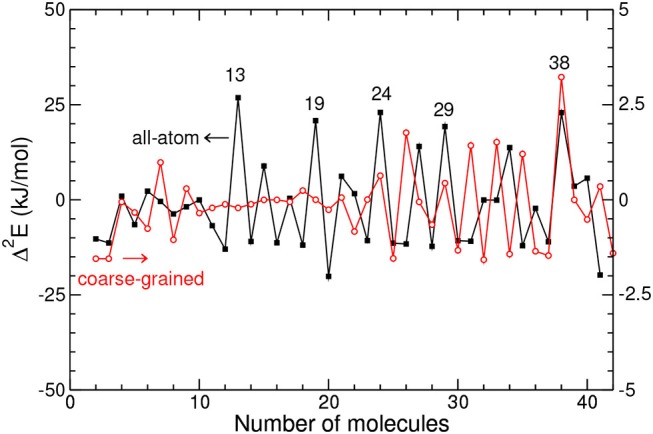
Second-energy derivative vs. cluster size for the all-atom and coarse-grained models.

The variations of Δ^2^*E* with increasing size *N* are presented in Figure 2, as obtained by both the AA and CG models.

From this figure it is clear that the two models do not predict the same special stabilities in the entire size range considered, except at *N* = 38. Prominent peaks in the AA model at *N* = 13, 19, 24, or 29 are not present in the coarse-grained description, and in the range around 30 the differences are rather systematic between the AA and CG models.

The energetic data obtained with atomistic details are essentially consistent with experimental data, indicating that our modeling of adamantane clusters is realistic. The contrasted behaviors between the two models suggest that the mutual orientations of the adamantane molecules play a significant role on the cluster structures.

### 4.2. Main Structural Motifs

**Figure 3:**
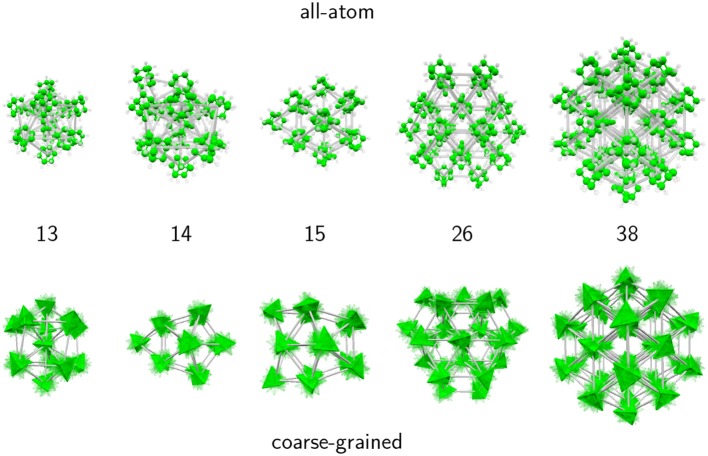
Remarkable structures obtained for selected adamantane clusters with 13–15, 26, and 38 molecules, in the all-atom and coarse-grained models.

Selected structures obtained with the AA and CG models are presented in Figure 3, notably for *N* = 13 and 38 but also *N* = 14, 15 and *N* = 26, which illustrate the differences between the two descriptions. While the true atomic positions are used for the AA structures, we used fuzzy tetrahedra to represent the adamantane molecules in the CG model.

For *N* = 13, the structure in both models corresponds to an icosahedral packing, however for the CG model the structure does not strictly belong to the *I*_*h*_ point group, the symmetry being lowered due to the important strain within the cluster. In the AA description the molecules manage to adopt appropriate orientations that bring the translational structure closer to the perfect icosahedron.

At size 14 both models predict a qualitatively different structural motif, as a capped icosahedron with all atoms, but showing a decahedral arrangement after coarse-graining. Decahedral motifs are known to occur as an intermediate packing scheme on the way from the highly coordinated, but highly strained icosahedra to the low coordinated and weakly strained close packed structures (Doye et al., 1995). Their presence in the CG model is thus not accidental.

At size 15 the all-atom model now predicts a cubic motif while the isotropic potential still yields a (doubly capped) decahedron. The cubic translational arrangement is preserved at sizes 16 and beyond, while the coarse-grained model further experiences some structural changes. At sizes 26 and above, both models favor close-packed cubic structures, leading to the perfect truncated octahedron at *N* = 38 as a strong magic number. These results thus support the interpretation of experimental mass spectra from the Scheier group (Goulart et al., 2016), namely that adamantane clusters exhibit icosahedral and cubic packing as their main structural motifs, at low and large sizes, respectively. Our results indicate that icosahedral packing is the dominant motif only up to *N* = 14, and that orientational effects are already non-negligible at this size.

### 4.3. Structural Analysis

To shed more light onto the respective roles of translational and orientational orderings on the stable structures of adamantane clusters, and to clarify the effects of coarse-graining, we now consider the structural order parameters introduced in section 3.2 in comparison between the two models. Near size 14 where the icosahedral-cubic transition takes place, additional but metastable structures were generated as belonging to the icosahedral and cubic families, by performing basin-hopping global optimization with orientational moves only.

**Figure 4:**
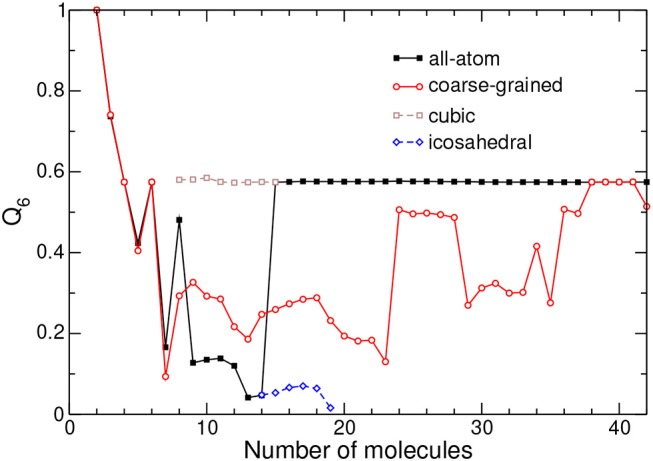
Bond-orientational order parameter *Q*_6_ obtained from the relative positions of the centers of mass of adamantane clusters in the all-atom and coarse-grained models. The values obtained for metastable icosahedral and cubic conformations near the corresponding transition are also shown.

The bond-orientational order parameter *Q*_6_ is shown against increasing cluster size for both models in Figure 4.

Within the all-atom description, *Q*_6_ exhibits irregular, essentially decreasing variations during the completion of icosahedral packing at *N* = 13. Above this size, *Q*_6_ reaches about 0.58 and stays constant at this value, indicating that the face-centered cubic structure is robust and regular with no point defect or stacking fault.

In the CG model, *Q*_6_ displays the same value as in the AA description up to size 7, indicating that translational structures are identical. Differences above the critical size of *N* = 14 show that the cubic packing is less ideal for the CG model, except near size 40 where *Q*_6_ reaches the same value as in the AA model. As confirmed by visual inspection along the lines of Figure 3, decahedral packings are often found, with a signature on *Q*_6_ being lower than ~0.4, except for *N* = 24–28, *N* = 34, and *N* > 36 for which the cubic motif is lower in energy, *Q*_6_ being also higher.

**Figure 5:**
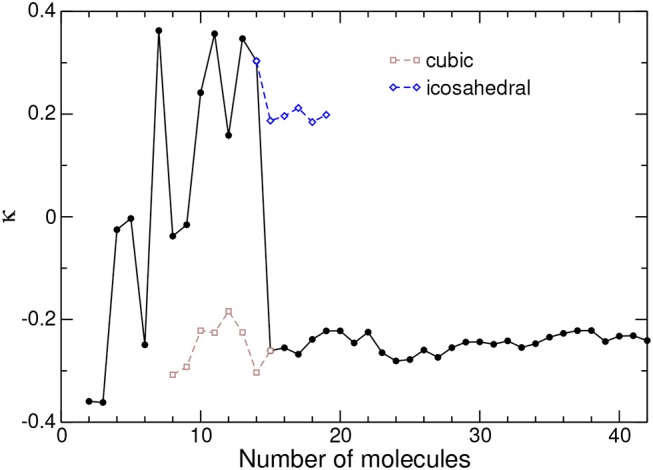
Tetrahedral order parameter κ between nearest-neighbor molecules in adamantane clusters, as obtained from the all-atom model. The values obtained for metastable icosahedral and cubic conformations near the corresponding transition are also shown.

The differences between the AA and CG models further support that orientational ordering plays a role in establishing the close-packed translational ordering itself. To further explore this aspect, the order parameter κ was evaluated for atomistic global minima, the results of which are depicted in Figure 5 against cluster size.

Similar to *Q*_6_, the orientational order parameter displays irregular variations during the completion of the icosahedron at *N* = 13, with positive and negative values alike. The tetrahedra in this size range thus do not possess any robust and specific orientational preference. Once the cubic packing is set at *N* ≥ 15, and as was the case for the translational order parameter, κ reaches an essentially constant value close to −0.25, with fluctuations of magnitude no greater than 0.05.

**Figure 6:**
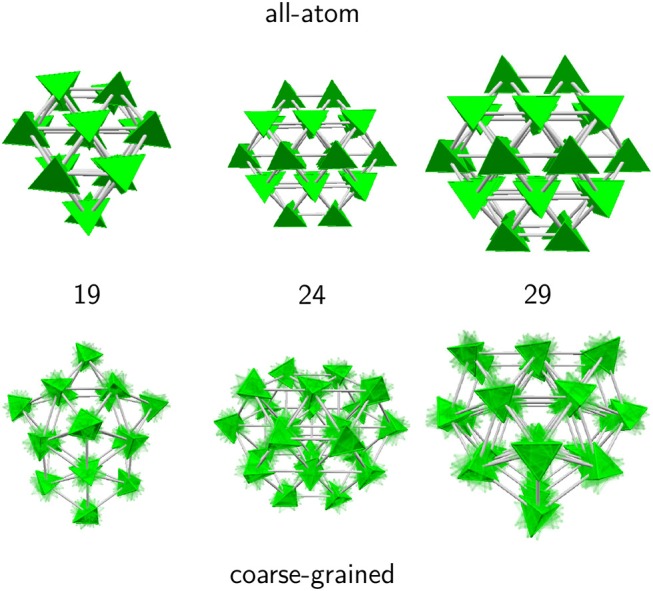
Selected adamantane clusters for which the second energy derivative shows peaks in the 19–29 size range. In the all-atom model, molecules were replaced by their equivalent tetrahedra to emphasize orientational ordering.

This stability further indicates that the clusters adopt a constant growth scheme. However, the negative value of κ also shows that the tetrahedral molecules do not display a single, common orientation within the cluster, as otherwise κ would be closer to unity. To illustrate the specific orientational ordering, we have represented in Figure 6 another set of clusters obtained for both the AA and CG models, and chosen at sizes for which the AA description predicts special stabilities, namely 19, 24, and 29. In the AA model, the atomic details were replaced by equivalent tetrahedra, contrasting with the fuzzy tetrahedra in the CG model.

In this size range, the coarse-grained potential predicts both decahedral (19 and 29) and cubic (24) motifs. The structures obtained with the AA model show the same close-packed motif, with clusters of a given size that are subparts of larger global minima. More interestingly, and as suggested by the indicators previously discussed, the molecules show two different possible orientations that alternate between planes in the largest clusters. While molecules with the same orientation within a same plane are next nearest neighbors, nearest-neighbor molecules precisely belong to different planes and present parallel contact faces.

However, in the dimer at equilibrium (see Figure 1), the two tetrahedra do not display such a relative orientation, and instead rotate in order to maximize dispersive attractions while minimizing Coulomb repulsion between the (positively charged) peripheral hydrogens. In clusters, this difference in relative orientations gives rise to orientational strain (Calvo et al., 1999), which the system exploits to minimize the overall energy while deviating from the ideal orientations that would be adopted in absence of environment.

We have quantified the importance of strain in adamantane clusters by removing from their total potential energy the contribution between nearest-neighbor pairs, as if these pairs were at equilibrium (including their orientational degrees of freedom) (Doye et al., 1995). Omitting the contribution of non-nearest neighbors, the strain energy *V*_strain_ reads

(9) $$\begin{array}{r} V_{\text{strain}}=\overset{nn\;only}{\underset{a<b}{\sum }}V_{ab}-n_{nn}V_{\text{min}}, \end{array}$$

where *V*_min_ denotes the minimum energy in the dimer at equilibrium.

**Figure 7:**
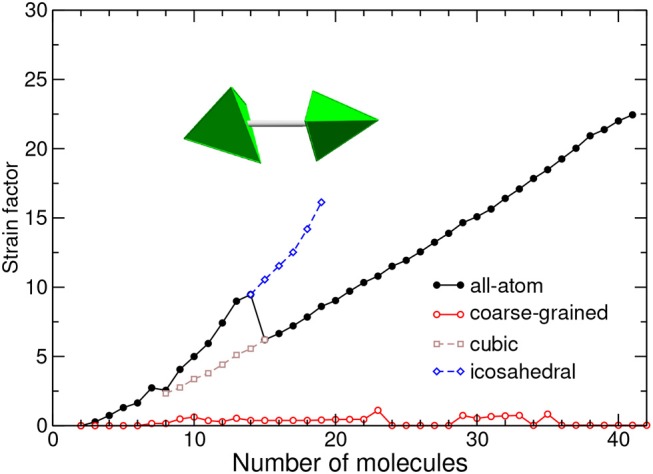
Strain energy normalized by dimer energy, as a function of cluster size and for the all-atom and coarse-grained models. The equivalent tetrahedra in the equilibrium dimer geometry are depicted. The values obtained for metastable icosahedral and cubic conformations near the corresponding transition are also shown.

In order to compare the two models, we have deemed more suitable to further normalize the strain energy by the magnitude of the dimer binding energy, considering thus a strain factor *V*_strain_/|*V*_min_| instead of the absolute strain energy. The variations of the strain factor with increasing size are represented in Figure 7 for both models.

With the coarse-grained description, which ignores orientational degrees of freedom, most structures are either icosahedral or decahedral and thus exhibit moderate strain (Doye et al., 1995), cubic packings being characterized with a very low strain factor. In this respect, the strain factor is an even more direct probe of close packed structures than *Q*_6_ previously considered.

In contrast, the all-atom model shows strongly increasing strain as the cluster size increases, with a peak at *N* = 14 and a change in slope above this size. The growing strain conveys the inability of the adamantane molecules to respect their ideal mutual orientation in the equilibrium dimer. However, in fairness it should be recognized that this orientation is not so meaningful as soon as the cubic motif is established. If instead of the equilibrium dimer we had artificially chosen the orientations between nearest neighbor in close-packed clusters to define the strain energy, the strain factor would be much reduced and similar to the value in the CG model, but the values in icosahedral structures would become negative and less physical.

## 5. Concluding Remarks

The remarkable thermodynamical and chemical stability of adamantane makes it a valuable building block of supramolecular materials, including non-covalent molecular clusters. Recent mass spectrometry measurements under the cryogenic conditions of helium droplets have found magic numbers for cationic adamantane clusters at the sizes of 13, 19, and 38 molecules, as well as others higher suggesting close packed geometries (Goulart et al., 2016). In the present work, we have modeled (neutral) adamantane clusters using a rigid body description and a site-site pairwise force field comprising the traditional Lennard-Jones potential for repulsion-dispersion forces with Coulomb interactions acting between partial charges. A spherically averaged coarse-grained model was also developed, producing an effective pair potential that allows an efficient exploration of the translational structure of adamantane clusters. The all-atom force field was successfully validated against quantum chemistry calculations, which incidentally highlighted the difficulty of producing accurate and reliable interaction energies and geometries for such rather large non-covalent edifices.

Using the basin-hopping algorithm, the putative global minima of (C_10_H_16_)_*N*_ clusters were found to follow icosahedral packing up to *N* = 14 and sharply change into close-packed cubic structure above this size. Translational and orientational order parameters indicate that cubic structures are stabilized by having molecules with two possible orientations in alternating planes. This feature is obviously absent with the CG model, which predicts numerous decahedral structures in the intermediate range 14–35, before the structure eventually also adopts the close-packed cubic motif; this intermediate decahedral phase is absent from the all-atom structures.

Comparison between the all-atom and coarse-grained models highlights and explains the importance of orientational strain in the structure of adamantane clusters, in particular the sharp transition toward the cubic motif which arises due to a combination between the short range of the potential and the optimal orientations presented by the nearest-neighbor molecules with tetrahedral facets parallel to one another.

Here we have neglected the cationic nature of the adamantane clusters in the mass spectrometry experiments, but in a first approximation it could be accounted for by adding a polarization contribution and assuming the *N*-molecule cationic cluster to be made of a single cationic molecule surrounded by *N*−1 neutral ones. Such an additional contribution would bind the first solvation shell more strongly, possibly leading to some structural distortion, and could even modify the details of the icosahedral-to-cubic transition, but would probably not change the qualitative picture or the special stabilities found at 13 or 38. Further efforts should also be devoted to making the basin-hopping optimization method even more efficient for the present clusters. Although we have focused on the chemical physics rather than the algorithmic efficiency, it was clear that basin-hopping in its conventional approach was struggling to locate the correct molecular orientations in medium- to large-size clusters. Having analyzed the structures, such a deceiving efficiency appears more clearly and is most likely due to the collective nature of the orientational ordering in the clusters, where the orientation is constant within a plane but alternates between planes. Tailored moves that incorporate such a specificity should enable much larger clusters to be addressed in the future.

## Data Availability

The raw data supporting the conclusions of this manuscript will be made available by the authors, without undue reservation, to any qualified researcher.

## Author Contributions

JH-R and FC conceived the project, performed the electronic structure calculations and prepared, wrote, and discussed the manuscript. FC built and conducted the coarse-grained calculations. JH-R conducted the all-atom basin-hopping calculations.

### Conflict of Interest Statement

The authors declare that the research was conducted in the absence of any commercial or financial relationships that could be construed as a potential conflict of interest.
