# Supplementary material for: The Structure of Adamantane Clusters: Atomistic vs. Coarse-Grained Predictions From Global Optimization
Source: Front Chem. 2019 Aug 16;7:573. doi: 10.3389/fchem.2019.00573 (PMC6707085; doi:10.3389/fchem.2019.00573)
Supplement: Supplementary file 1 [file Data_Sheet_1.PDF]

Supplementary Material

In this document we provide the Cartesian coordinates (xyz format) of the global minima obtained using the all-atom model.

CARTESIAN COORDINATES

Adamantane monomer

```
26
E=0
C     0.964660    -0.737211    -0.946286
C     1.734989    -0.229457     0.282275
C     0.774783     0.506874     1.229258
C     0.144720     1.697533     0.490474
C    -0.629753     1.194987    -0.737755
C     0.333951     0.456545    -1.679520
C    -0.334224    -0.456467     1.679668
C    -0.144496    -1.697676    -0.489968
C    -1.734905     0.229521    -0.282509
C    -1.109680    -0.964787     0.454356
H     1.651006    -1.261584    -1.620135
H     2.207904    -1.070092     0.804209
H     2.542297     0.443091    -0.032184
H     1.325999     0.867507     2.104695
H    -0.528226     2.243658     1.162596
H     0.924391     2.403740     0.180250
H    -1.077596     2.045526    -1.263177
H    -0.202856     0.109492    -2.570787
H     1.116805     1.140871    -2.028544
H     0.099976    -1.301819     2.227106
H    -1.015913     0.052373     2.371922
H    -0.689287    -2.083591    -1.360180
H     0.294055    -2.564562     0.018895
H    -2.441197     0.749809     0.375723
H    -2.308792    -0.121043    -1.148952
H    -1.899109    -1.651735     0.778631
```

Adamantane$_2$

```
    52
E=-14.0818
   C        1.0849164533       -0.3865436828        2.5451245600
   C       -0.3386929402        0.1556607813        2.3452886951
   C       -0.4442188453        1.5686962342        2.9395517135
   C       -0.1215808524        1.5149538433        4.4407060340
   C        1.3026210469        0.9761493905        4.6462432934
   C        1.4038066029       -0.4352078813        4.0472623736
   C        0.5615635619        2.4930774540        2.2361943808
   C        2.0864978927        0.5428648153        1.8422865857
   C        2.3040040801        1.9016905468        3.9381897892
   C        1.9875159999        1.9564987732        2.4358326053
```

```
H        1.1579193121    -1.3939708840     2.1210128449
H       -0.5873074391     0.1765929058     1.2773540388
H       -1.0651889419    -0.5100366372     2.8269554532
H       -1.4598193211     1.9535046450     2.7954517071
H       -0.2130898425     2.5152576310     4.8810188236
H       -0.8446967778     0.8733804512     4.9587234901
H        1.5302493326     0.9381941248     5.7171276482
H        2.4110236410    -0.8396907865     4.2049743179
H        0.7079775307    -1.1114913255     4.5585758326
H        0.3303300600     2.5571905010     1.1660277306
H        0.4824471115     3.5102377173     2.6386354886
H        3.1057633244     0.1553130697     1.9601799148
H        1.8814234949     0.5717636326     0.7653148133
H        2.2555282304     2.9087409383     4.3698350099
H        3.3272208261     1.5389878781     4.0945205785
H        2.7021085087     2.6172215939     1.9327892632
C       -1.0968778738     0.4183395018    -2.5349496537
C       -0.2864713866     1.5061495139    -1.8134966324
C       -1.1746550770     2.7345904964    -1.5624668979
C       -1.6819318856     3.2791393423    -2.9065593825
C       -2.4959560987     2.1959709517    -3.6311404140
C       -1.6045215351     0.9687478300    -3.8765254914
C       -2.3745528470     2.3262062612    -0.6939865669
C       -2.2966718448     0.0158190724    -1.6635034504
C       -3.6922481469     1.7885364641    -2.7572887962
C       -3.1901337396     1.2402967823    -1.4128334686
H       -0.4618375527    -0.4563519597    -2.7131303703
H        0.0996180804     1.1197817607    -0.8625041845
H        0.5841339831     1.7887757613    -2.4177491076
H       -0.5950056239     3.5086260355    -1.0475279238
H       -2.3015042155     4.1690301941    -2.7419953658
H       -0.8355648221     3.5930868595    -3.5294196350
H       -2.8561090714     2.5862030211    -4.5892934854
H       -2.1684296924     0.1948281451    -4.4113353185
H       -0.7569404567     1.2416444614    -4.5169258289
H       -2.0275754451     1.9544230578     0.2777232698
H       -3.0065632243     3.1993616057    -0.4915121575
H       -2.8724205417    -0.7755842894    -2.1586714722
H       -1.9472847634    -0.3966913726    -0.7092903457
H       -4.3474648136     2.6520398123    -2.5903424494
H       -4.2935188612     1.0296232163    -3.2725452966
H       -4.0443976884     0.9508482430    -0.7909795047
```

Adamantane$_3$

```
    78
E=-38.4426
C       -0.0823714368    -5.1882811048    -0.3736507795
C       -0.6086735213    -5.3678921265    -1.8058971813
C       -1.5436936569    -4.2027736261    -2.1653268250
C       -2.7254040625    -4.1770663233    -1.1838241683
C       -2.2047825520    -3.9944774814     0.2502335052
C       -1.2677531029    -5.1599071361     0.6033560610
C       -0.7698399814    -2.8792632869    -2.0640287587
```

C	0.6880548936	-3.8623373913	-0.2774047806
C	-1.4288263168	-2.6717639755	0.3453916798
C	-0.2439136151	-2.6935832125	-0.6322337929
H	0.5837539877	-6.0199802139	-0.1193611009
H	0.2290189979	-5.4103878834	-2.5124360035
H	-1.1449192473	-6.3207489988	-1.8922491436
H	-1.9172786165	-4.3325900742	-3.1870257137
H	-3.4112274731	-3.3611755889	-1.4423304053
H	-3.2986645383	-5.1089673421	-1.2599034816
H	-3.0488815929	-3.9769894847	0.9482589483
H	-0.9043143607	-5.0520782580	1.6325551930
H	-1.8152787377	-6.1092525169	0.5593347550
H	0.0653612850	-2.8754215030	-2.7748453030
H	-1.4218237815	-2.0407224036	-2.3371579785
H	1.0868938239	-3.7318524488	0.7359551753
H	1.5487680553	-3.8769083208	-0.9569041480
H	-2.0918350049	-1.8292811221	0.1141772407
H	-1.0688058247	-2.5195248390	1.3701714324
H	0.3077843321	-1.7495819909	-0.5633805285
C	-0.7619226053	3.4997452667	-2.0482902855
C	-1.6571442723	3.5047840268	-3.2969441753
C	-1.5562881766	2.1493091900	-4.0135120073
C	-0.0981474082	1.9001991758	-4.4285029873
C	0.8013963227	1.8910720003	-3.1828527057
C	0.6942097109	3.2470568306	-2.4681764307
C	-2.0108824340	1.0340967248	-3.0593444927
C	-1.2191011366	2.3812147067	-1.0991728369
C	0.3406047053	0.7768697384	-2.2305089753
C	-1.1163840321	1.0230431798	-1.8099995103
H	-0.8351078434	4.4669491954	-1.5391044538
H	-2.6975934095	3.7046246133	-3.0136402994
H	-1.3523951916	4.3117853762	-3.9743134831
H	-2.1957503148	2.1549604008	-4.9030865624
H	-0.0163788993	0.9438942543	-4.9592116867
H	0.2333456712	2.6789899855	-5.1259643531
H	1.8404689421	1.7138694796	-3.4811409864
H	1.3474284025	3.2608760197	-1.5872223964
H	1.0401224405	4.0497353599	-3.1307701298
H	-3.0577525650	1.1880662186	-2.7709135685
H	-1.9617215829	0.0626396622	-3.5659880021
H	-0.6003525141	2.3801748843	-0.1935535605
H	-2.2519620893	2.5599386153	-0.7766594794
H	0.4304045368	-0.1992889990	-2.7223098550
H	0.9879609503	0.7464861499	-1.3456520488
H	-1.4433256729	0.2274270768	-1.1315056757
C	0.8556221249	1.6795823924	2.4423838501
C	0.3572380569	0.2671401948	2.1000329806
C	1.4822797952	-0.7521013931	2.3374800602
C	2.6832620581	-0.4015248998	1.4457482958
C	3.1878187965	1.0093051546	1.7861714276
C	2.0583028177	2.0240025094	1.5505256343
C	1.9119348356	-0.7016846771	3.8118087646
C	1.2864100943	1.7235258633	3.9166421215
C	3.6127162360	1.0554937822	3.2619976208

C	2.4141868564	0.7085564408	4.1583276727
H	0.0522076786	2.4046189647	2.2722165020
H	-0.5148201438	0.0158968202	2.7157626408
H	0.0288849311	0.2279259921	1.0542802229
H	1.1245713803	-1.7584382592	2.0933132047
H	3.4863242206	-1.1341346246	1.5914571487
H	2.3953724599	-0.4529927100	0.3887119731
H	4.0436323389	1.2573043525	1.1488896073
H	2.4117327450	3.0385941226	1.7711084634
H	1.7595672304	2.0158206228	0.4952204872
H	1.0687779679	-0.9702962765	4.4597056542
H	2.7020690197	-1.4390135411	3.9985368471
H	1.6253904012	2.7329938498	4.1794423283
H	0.4315626825	1.4977285984	4.5654952225
H	4.4323868454	0.3488259229	3.4397013747
H	3.9943052796	2.0527322688	3.5127370340
H	2.7191374360	0.7414629852	5.2100747939

Adamantane$_4$

104
E=-74.1602

C	-2.5850069753	-1.6245816323	-2.9147333315
C	-1.6921788197	-1.3824004154	-1.6880345496
C	-0.2276984089	-1.6806492062	-2.0448233224
C	-0.1006201757	-3.1453459879	-2.4908751953
C	-0.9893983433	-3.3923846678	-3.7196762829
C	-2.4518798051	-3.0893740106	-3.3587351195
C	0.2143134211	-0.7595762449	-3.1924472022
C	-2.1369444342	-0.7031809534	-4.0598130241
C	-0.5458522457	-2.4666792411	-4.8629481220
C	-0.6739663959	-1.0003140492	-4.4229417098
H	-3.6285307342	-1.4116232651	-2.6581858145
H	-1.7931707870	-0.3447776371	-1.3476357458
H	-2.0143701669	-2.0214994753	-0.8569524318
H	0.4070890619	-1.5067809092	-1.1689801095
H	0.9446342794	-3.3764272696	-2.7294155084
H	-0.3945778191	-3.8149347637	-1.6735044600
H	-0.8976362347	-4.4372964252	-4.0355681550
H	-3.1001696836	-3.2809356206	-4.2224422590
H	-2.7876726992	-3.7582526014	-2.5569724295
H	0.1482806644	0.2897978295	-2.8805340713
H	1.2648339827	-0.9496318040	-3.4436815418
H	-2.7801503007	-0.8518128549	-4.9357079859
H	-2.2449705033	0.3468491407	-3.7622798010
H	0.4914407074	-2.6859857018	-5.1435653012
H	-1.1599339138	-2.6476686304	-5.7535162534
H	-0.3568087260	-0.3418529515	-5.2390848950
C	4.7768681713	-0.4204139198	-0.5480928442
C	3.9338501208	-0.8157348120	-1.7702326552
C	3.9515478937	-2.3426317674	-1.9411885551
C	5.3994750289	-2.8155953052	-2.1418382902
C	6.2465804573	-2.4254355323	-0.9207344460
C	6.2226923927	-0.8983630654	-0.7521371476

C	3.3711913493	-3.0026591315	-0.6808947055
C	4.1938624064	-1.0852059067	0.7085306660
C	5.6602043560	-3.0848061723	0.3370920933
C	4.2129726205	-2.6126296553	0.5440109056
H	4.7631077077	0.6684097758	-0.4281622824
H	2.9029817143	-0.4624033287	-1.6469486556
H	4.3291980678	-0.3318462898	-2.6716228359
H	3.3494517282	-2.6222496969	-2.8127278097
H	5.4235013836	-3.9026729715	-2.2851623894
H	5.8199357192	-2.3668977823	-3.0499894255
H	7.2784992882	-2.7631140676	-1.0662173427
H	6.8404071343	-0.6046502750	0.1052582889
H	6.6581147382	-0.4156480905	-1.6355240855
H	2.3299949052	-2.6896784030	-0.5367104393
H	3.3605807583	-4.0930213405	-0.7984996703
H	4.7750860972	-0.7941713063	1.5919756815
H	3.1673196743	-0.7376634809	0.8764089861
H	5.6891121930	-4.1766370224	0.2376550418
H	6.2683303993	-2.8303751266	1.2136835655
H	3.7964061585	-3.0841578227	1.4408850738
C	-1.6908339843	-1.6277359490	3.4361533072
C	-1.9168159097	-3.1050467873	3.0796864568
C	-0.5663426351	-3.7863495901	2.8094978772
C	0.1362714987	-3.0781897163	1.6410412946
C	0.3675972476	-1.6006427227	1.9935326174
C	-0.9852239644	-0.9250169267	2.2662056082
C	0.3131081741	-3.6891690660	4.0656478075
C	-0.8088049818	-1.5364584464	4.6909999155
C	1.2442625645	-1.5088990803	3.2520139252
C	0.5445562604	-2.2131062205	4.4245108959
H	-2.6547519786	-1.1438882160	3.6281512278
H	-2.4386105871	-3.6145794531	3.8987337877
H	-2.5612137600	-3.1837702369	2.1956579572
H	-0.7298147415	-4.8396113924	2.5561145753
H	1.0933920421	-3.5682604242	1.4251527069
H	-0.4722263580	-3.1570475033	0.7319940177
H	0.8680932696	-1.0976733096	1.1589225039
H	-0.8356647332	0.1361053058	2.4999090179
H	-1.6135042517	-0.9653584567	1.3680879714
H	-0.1672712725	-4.2089600633	4.9034359554
H	1.2735801891	-4.1892447052	3.8917193089
H	-0.6567708985	-0.4862034653	4.9682134162
H	-1.3098830595	-2.0183815639	5.5391919444
H	2.2210482558	-1.9709964621	3.0644673482
H	1.4338753199	-0.4581300601	3.5028057111
H	1.1712648902	-2.1471451207	5.3206889642
C	-0.4415141431	3.6569173508	0.0225536938
C	0.1553458452	3.9446725626	1.4087495138
C	1.6313102534	3.5184363302	1.4371252551
C	1.7328455446	2.0151468918	1.1366934621
C	1.1399816833	1.7221074075	-0.2502265629
C	-0.3346129247	2.1533046310	-0.2744278904
C	2.4085186508	4.3038423635	0.3694760161
C	0.3409822512	4.4424638863	-1.0411957069

C	1.9180519042	2.5123945618	-1.3136113103
C	1.8170789984	4.0168837657	-1.0193963170
H	-1.4937274084	3.9612735417	0.0045458247
H	0.0670604305	5.0125063510	1.6425623431
H	-0.4071113425	3.4037910856	2.1794903905
H	2.0549489096	3.7250182279	2.4261352954
H	2.7809371442	1.6942352485	1.1737964510
H	1.1983155459	1.4408622114	1.9030679986
H	1.2129406951	0.6497920900	-0.4620410633
H	-0.7757034786	1.9311072150	-1.2537302295
H	-0.9059140947	1.5811796398	0.4666974807
H	2.3617894769	5.3785122354	0.5833384134
H	3.4682324707	4.0222421926	0.3927402107
H	-0.0886171238	4.2615268011	-2.0340216539
H	0.2570354607	5.5193575683	-0.8514279993
H	2.9693442792	2.2000455537	-1.3199701542
H	1.5173068213	2.2960538896	-2.3113914178
H	2.3728210559	4.5786534232	-1.7782212776

Adamantane$_5$

130
E=-108.9092

C	-2.4881837513	0.3726311891	-3.3555906024
C	-1.0814200817	-0.2256517812	-3.2017815514
C	-0.3894940835	0.3840613619	-1.9727812508
C	-0.2830232211	1.9062824906	-2.1515397579
C	-1.6877071497	2.5103503346	-2.3027745221
C	-2.3764772480	1.8949119975	-3.5306904038
C	-1.2192516292	0.0765209272	-0.7167112990
C	-3.3124372349	0.0645672726	-2.0959678127
C	-2.5144069877	2.1970586558	-1.0461322190
C	-2.6264895981	0.6756471244	-0.8645001867
H	-2.9795801375	-0.0631613699	-4.2323597665
H	-1.1450502398	-1.3154021206	-3.0962962257
H	-0.4887849454	-0.0284575406	-4.1033637085
H	0.6130522831	-0.0443230014	-1.8647337078
H	0.2275591136	2.3513736011	-1.2889526618
H	0.3239798913	2.1403718730	-3.0344919234
H	-1.6093289479	3.5955043360	-2.4306267450
H	-3.3736050304	2.3325616607	-3.6617041344
H	-1.8067766053	2.1291249760	-4.4381948426
H	-1.2861713349	-1.0076220128	-0.5650804548
H	-0.7253349829	0.4903833582	0.1707555984
H	-4.3264349655	0.4690371510	-2.2018447818
H	-3.4158137895	-1.0199230828	-1.9696633947
H	-2.0433785645	2.6475375355	-0.1640783664
H	-3.5137567290	2.6406215194	-1.1328336109
H	-3.2157320565	0.4542101026	0.0321651211
C	-2.0619378739	-0.5073686930	4.2239331041
C	-1.8390511540	-1.9348371538	3.7012103646
C	-0.3336379391	-2.2367104645	3.6415697753
C	0.3498641847	-1.2330029915	2.7003769318

C	0.1326709886	0.1964832361	3.2202837447
C	-1.3739405202	0.4920060817	3.2813903498
C	0.2681002271	-2.1047090945	5.0491482793
C	-1.4560152804	-0.3802683793	5.6302225747
C	0.7327584816	0.3222828698	4.6291143338
C	0.0503351028	-0.6774772884	5.5751712627
H	-3.1356782842	-0.2941381543	4.2654541651
H	-2.3426798848	-2.6579379390	4.3540618862
H	-2.2841380264	-2.0436737941	2.7047141926
H	-0.1771000273	-3.2550933969	3.2691738347
H	1.4227325945	-1.4506192696	2.6336427861
H	-0.0568159881	-1.3301672424	1.6864610135
H	0.6203131265	0.9103420686	2.5474841814
H	-1.5424363899	1.5173216013	3.6325354294
H	-1.8111482190	0.4257986679	2.2776748251
H	-0.1966165451	-2.8304541525	5.7275054911
H	1.3396852154	-2.3367951740	5.0231689834
H	-1.6266682341	0.6293331236	6.0233886815
H	-1.9518776436	-1.0758257997	6.3180301353
H	1.8124337141	0.1324204062	4.5963785341
H	0.6021795789	1.3447640118	5.0038762785
H	0.4798121110	-0.5867173306	6.5789450584
C	4.9992484766	-0.9111947825	-0.2966817566
C	3.9900163397	-0.4798197015	-1.3718328122
C	3.3659289957	0.8702736012	-0.9861950235
C	2.6483020132	0.7318237747	0.3652083111
C	3.6539188933	0.3034490691	1.4450863853
C	4.2775748551	-1.0449954513	1.0530447741
C	4.4741530359	1.9278609468	-0.8672878728
C	6.1033883346	0.1509870809	-0.1800986656
C	4.7617120635	1.3621636352	1.5575672076
C	5.4846418089	1.5022910284	0.2092413377
H	5.4427832092	-1.8739066407	-0.5733992099
H	4.4880881491	-0.4001526399	-2.3456445934
H	3.2063023707	-1.2397655827	-1.4777904997
H	2.6471637397	1.1761450867	-1.7543784603
H	2.1811471388	1.6846518069	0.6422317397
H	1.8409583202	-0.0065962014	0.2891343711
H	3.1400237873	0.2048455971	2.4074988308
H	4.9834734416	-1.3721557074	1.8261159171
H	3.4991409848	-1.8150508927	0.9895296210
H	4.9818751608	2.0520876641	-1.8314569663
H	4.0388551256	2.9013450966	-0.6110114620
H	6.8421302164	-0.1553261613	0.5705209735
H	6.6392904828	0.2429881633	-1.1325184980
H	4.3319053087	2.3260422557	1.8559330131
H	5.4758281635	1.0779889155	2.3400299705
H	6.2737045659	2.2578781433	0.2912034060
C	-0.4198046526	-3.8738393110	-1.2255270560
C	-0.7849566765	-4.2973804969	0.2055077522
C	0.2979989191	-5.2347836222	0.7617774723
C	0.4008289039	-6.4804624799	-0.1316194109
C	0.7689636681	-6.0627840786	-1.5637256925
C	-0.3149813591	-5.1229423867	-2.1139136420

```
   C       1.6489411199       -4.5028953235        0.7710032988
   C       0.9328415006       -3.1450884832       -1.2107836019
   C       2.1184573049       -5.3283633764       -1.5486481511
   C       2.0200503772       -4.0798501014       -0.6588299488
   H      -1.1931670317       -3.2058549470       -1.6202639938
   H      -0.8832144543       -3.4130063308        0.8465689790
   H      -1.7584648216       -4.8025596780        0.2108224154
   H       0.0358998894       -5.5350262356        1.7822896589
   H       1.1573551625       -7.1674129393        0.2666568777
   H      -0.5518842057       -7.0237790298       -0.1315843703
   H       0.8411843842       -6.9523467492       -2.1989508205
   H      -0.0741491417       -4.8327771586       -3.1438503504
   H      -1.2803638732       -5.6423237865       -2.1493877331
   H       1.5960811685       -3.6218752544        1.4220375051
   H       2.4273282294       -5.1556713975        1.1843193574
   H       1.1954478041       -2.8191102233       -2.2245751565
   H       0.8662560799       -2.2402812930       -0.5946268204
   H       2.9053564143       -5.9950699413       -1.1756734289
   H       2.4027273920       -5.0424190497       -2.5686439405
   H       2.9832067055       -3.5578536233       -0.6488666368
   C       0.0464717860        4.9466411779        0.7166369770
   C      -0.3025868896        6.3533886279        1.2263461213
   C       0.4321157544        6.6236279018        2.5485171400
   C       0.0045127571        5.5792988736        3.5911006478
   C       0.3541920346        4.1703395592        3.0876476644
   C      -0.3799854664        3.9065136423        1.7638934234
   C       1.9478653289        6.5213854169        2.3186473306
   C       1.5632474335        4.8491761105        0.4911620337
   C       1.8700494903        4.0743644027        2.8559169606
   C       2.3024169160        5.1148708825        1.8115748570
   H      -0.4784540718        4.7559423611       -0.2258048169
   H      -0.0218718185        7.1052314403        0.4789083731
   H      -1.3859051399        6.4415240862        1.3733361508
   H       0.1822389836        7.6272923240        2.9097732517
   H       0.5074562698        5.7736537664        4.5461560988
   H      -1.0733067106        5.6544978993        3.7795295348
   H       0.0479731402        3.4274593884        3.8322331736
   H      -0.1543683892        2.8955296276        1.4033744973
   H      -1.4646838819        3.9516783136        1.9199970682
   H       2.2706683860        7.2758563005        1.5910430794
   H       2.4846097355        6.7314290665        3.2517177280
   H       1.8232662792        3.8551788786        0.1072910714
   H       1.8781974630        5.5743714062       -0.2688755383
   H       2.4059124409        4.2418521182        3.7980511118
   H       2.1356219854        3.0661623200        2.5153808231
   H       3.3834713778        5.0453232907        1.6480692411
```

Adamantane$_6$

```
   156
E=-150.1757
   C      -3.5647197816        0.8744813399        1.6490423858
   C      -2.4490412197       -0.1679899973        1.8196255514
   C      -2.7100240406       -1.0060665223        3.0807635884
```

C	-2.7414154124	-0.0825067037	4.3081590652
C	-3.8577942322	0.9604306941	4.1441872130
C	-3.5941063738	1.7932174697	2.8801140713
C	-4.0642344343	-1.7194392059	2.9465844120
C	-4.9165648232	0.1559235721	1.5181360373
C	-5.2090727439	0.2421621205	4.0068161248
C	-5.1839537138	-0.6806047850	2.7787623691
H	-3.3766457257	1.4709591635	0.7496247490
H	-2.4033245941	-0.8184651162	0.9378524156
H	-1.4759384665	0.3323000427	1.8953438895
H	-1.9137093395	-1.7489869465	3.2000899959
H	-2.9072356482	-0.6719100284	5.2180844939
H	-1.7732014939	0.4187018818	4.4273517909
H	-3.8779542925	1.6180539506	5.0200717742
H	-4.3739489588	2.5553664811	2.7620460113
H	-2.6411467563	2.3280132093	2.9741371124
H	-4.0490908422	-2.3984130143	2.0854216437
H	-4.2534081406	-2.3367662732	3.8331248003
H	-5.7197909482	0.8889741606	1.3750911502
H	-4.9154789930	-0.4895314460	0.6314875785
H	-5.4185938196	-0.3412655909	4.9115431664
H	-6.0176052526	0.9768358776	3.9093753544
H	-6.1479420432	-1.1922272802	2.6827486903
C	-1.6645474688	0.6605534401	-3.2531818723
C	-1.7726703809	-0.0013437056	-4.6354934737
C	-3.2222878721	-0.4444285540	-4.8869659164
C	-3.6429515019	-1.4485468764	-3.8028827470
C	-3.5396390396	-0.7906060692	-2.4181996487
C	-2.0893005762	-0.3462675904	-2.1731942088
C	-4.1464192111	0.7817672212	-4.8302595083
C	-2.5924892294	1.8841398327	-3.2011512745
C	-4.4625566414	0.4367562681	-2.3678114639
C	-4.0440693583	1.4456035970	-3.4482471239
H	-0.6303838229	0.9754510895	-3.0758269914
H	-1.4526053364	0.7003645075	-5.4151701400
H	-1.1001250195	-0.8659195056	-4.6915629126
H	-3.2971530881	-0.9155333405	-5.8732428202
H	-4.6701970455	-1.7879470740	-3.9829688781
H	-3.0032649097	-2.3384249298	-3.8449928138
H	-3.8394715203	-1.5084171738	-1.6469044772
H	-1.9975774524	0.1076579802	-1.1789587816
H	-1.4220726904	-1.2166818716	-2.1860306977
H	-3.8703319644	1.4985661382	-5.6130821057
H	-5.1825231909	0.4810806966	-5.0275142072
H	-2.5091016774	2.3781264216	-2.2254356505
H	-2.2880891157	2.6197709664	-3.9554365354
H	-5.5043007748	0.1305991334	-2.5222619899
H	-4.4133895243	0.9041714971	-1.3768166865
H	-4.7034857460	2.3196999260	-3.4111615647
C	0.2112371550	-4.5483340822	0.2341880338
C	-0.7558608847	-3.3559478456	0.2935193350
C	-2.1875882915	-3.8339284407	0.0060395506
C	-2.2411276656	-4.4690165876	-1.3919033646
C	-1.2777935437	-5.6643411037	-1.4558840179

C	0.1515508254	-5.1817632507	-1.1642690350
C	-2.5954499433	-4.8795482464	1.0553814979
C	-0.2028146781	-5.5917520584	1.2833718884
C	-1.6875813254	-6.7051548339	-0.4025488722
C	-1.6327277500	-6.0757793369	0.9978522573
H	1.2312794340	-4.2053977038	0.4391712516
H	-0.7083436324	-2.8812219117	1.2809954874
H	-0.4593292652	-2.5950666955	-0.4386563495
H	-2.8754404864	-2.9823038525	0.0494166185
H	-3.2632968049	-4.7965261351	-1.6172135124
H	-1.9709996138	-3.7272558466	-2.1532685744
H	-1.3172197300	-6.1151794829	-2.4535006134
H	0.8527891868	-6.0228162550	-1.2263998964
H	0.4642697868	-4.4528852918	-1.9218458432
H	-2.5816261098	-4.4336089991	2.0572712941
H	-3.6235278582	-5.2147320102	0.8722397373
H	0.4926054607	-6.4397256147	1.2652365302
H	-0.1463086082	-5.1574770673	2.2888424972
H	-2.6998590388	-7.0723596602	-0.6105691641
H	-1.0193381261	-7.5734909723	-0.4516246095
H	-1.9257069563	-6.8191129198	1.7474308541
C	3.3931409820	-0.1579176343	-3.8660350945
C	4.5077209796	-1.2018145908	-3.6969956553
C	4.2458934290	-2.0414334048	-2.4370591395
C	4.2155211249	-1.1196130627	-1.2083311008
C	3.1002415856	-0.0752588456	-1.3707585363
C	3.3647684731	0.7590715993	-2.6336378822
C	2.8909240293	-2.7531776667	-2.5722200026
C	2.0405315536	-0.8748541991	-3.9979306984
C	1.7481987756	-0.7918974874	-1.5091184758
C	1.7722995589	-1.7129173275	-2.7385034281
H	3.5818161878	0.4396580593	-4.7645976617
H	4.5527192676	-1.8510645969	-4.5797081435
H	5.4813553944	-0.7026648042	-3.6205896611
H	5.0414251588	-2.7853678629	-2.3188329141
H	4.0491075698	-1.7101529915	-0.2992513261
H	5.1842691420	-0.6196022935	-1.0884492668
H	3.0808072183	0.5811207287	-0.4939251998
H	2.5857292499	1.5222153596	-2.7505785927
H	4.3182969105	1.2927219398	-2.5388767027
H	2.9053198222	-3.4309236927	-3.4343621949
H	2.7011266675	-3.3715828236	-1.6865647182
H	1.2380771570	-0.1407480165	-4.1398893785
H	2.0409042222	-1.5190298282	-4.8855096915
H	1.5380905568	-1.3764080465	-0.6052269236
H	0.9404412501	-0.0562283314	-1.6054705578
H	0.8077668825	-2.2233799123	-2.8352214155
C	1.9824001417	-2.2033599284	4.8371319253
C	1.0377986199	-0.9916208644	4.8375377286
C	1.5025472996	0.0292715231	3.7874230487
C	2.9291851616	0.4906200122	4.1225495852
C	3.8789278103	-0.7172257548	4.1204620890
C	3.4078817041	-1.7363986394	5.1694369243
C	1.4940643901	-0.6288992265	2.3990680033

C	1.9721224999	-2.8558294789	3.4460443615
C	3.8641222228	-1.3738629812	2.7314543412
C	2.4399158369	-1.8398647554	2.3927446254
H	1.6494605648	-2.9297586370	5.5865356110
H	0.0119374585	-1.3136797417	4.6216240645
H	1.0220196863	-0.5280392075	5.8314167110
H	0.8273503128	0.8920536423	3.7890647815
H	3.2651945464	1.2352884725	3.3908232066
H	2.9460143405	0.9804539511	5.1036890426
H	4.8952571227	-0.3860533256	4.3601454025
H	4.0893513298	-2.5954982181	5.1920798679
H	3.4335555676	-1.2861598071	6.1692392521
H	0.4768678061	-0.9449187156	2.1377657524
H	1.8057848327	0.0960739071	1.6374397603
H	2.6274941997	-3.7352755877	3.4383184554
H	0.9630507857	-3.2108894711	3.2042479048
H	4.2169224064	-0.6622063247	1.9752170575
H	4.5541696277	-2.2261357645	2.7107002461
H	2.4312087725	-2.3072510838	1.4019248081
C	-0.4240760078	5.3175758793	0.3862045948
C	1.0229772610	5.7403384576	0.6826711166
C	1.9343497530	4.5032788119	0.6887585420
C	1.4544428019	3.5228195086	1.7698860489
C	0.0083694693	3.0942219819	1.4764871226
C	-0.8980201834	4.3349199551	1.4679815569
C	1.8701831356	3.8170489962	-0.6844718512
C	-0.4819094727	4.6296640561	-0.9865223876
C	-0.0511074761	2.4119921651	0.1010747677
C	0.4250276971	3.3895370053	-0.9842579632
H	-1.0721055380	6.2008392002	0.3825489418
H	1.3675634114	6.4583942993	-0.0712439625
H	1.0741804197	6.2498712945	1.6525715671
H	2.9655918325	4.8070986919	0.8997559482
H	2.1095949060	2.6436982401	1.7977864348
H	1.5137721036	3.9937020885	2.7586250305
H	-0.3318322407	2.3957241731	2.2487643705
H	-1.9375018167	4.0403577820	1.2790987891
H	-0.8807047492	4.8200201424	2.4515245565
H	2.2298860307	4.4993461977	-1.4641643742
H	2.5320180395	2.9426377353	-0.6990583586
H	-1.5141385165	4.3410452004	-1.2193303985
H	-0.1636846744	5.3271049359	-1.7707851279
H	0.5772266793	1.5130972658	0.0996102811
H	-1.0754533971	2.0827677680	-0.1117438904
H	0.3824461889	2.9012430391	-1.9640566778

Adamantane$_7$

```
       182
E=-189.1716
C    -3.765802    -1.955108    17.355266
C    -3.672608    -0.734662    18.283926
C    -3.258572     0.503639    17.473902
C    -1.891381     0.250550    16.820308
```

C	-1.979690	-0.967469	15.887818
C	-2.397389	-2.201928	16.701810
C	-4.303923	0.768837	16.379507
C	-4.810256	-1.683433	16.261532
C	-3.028601	-0.698838	14.797700
C	-4.398618	-0.448086	15.446102
H	-4.060846	-2.836761	17.934702
H	-4.638243	-0.559119	18.773278
H	-2.943109	-0.923890	19.080780
H	-3.193305	1.372647	18.137869
H	-1.575252	1.135615	16.254995
H	-1.130956	0.079078	17.591804
H	-1.003790	-1.146345	15.423414
H	-2.444959	-3.083469	16.050974
H	-1.645717	-2.417131	17.471002
H	-5.281616	0.971761	16.833131
H	-4.029409	1.662502	15.806062
H	-4.901234	-2.556081	15.603348
H	-5.796454	-1.524064	16.714054
H	-2.732395	0.169281	14.196493
H	-3.086887	-1.553469	14.112738
H	-5.145021	-0.256230	14.667434
C	0.489274	5.813209	18.633502
C	0.346518	4.338445	19.040040
C	-0.335784	3.552117	17.909949
C	-1.726611	4.147810	17.643121
C	-1.589479	5.622232	17.233023
C	-0.903608	6.403197	18.364668
C	0.514130	3.653843	16.633984
C	1.337491	5.908809	17.355874
C	-0.736000	5.718857	15.959063
C	0.657265	5.127112	16.221547
H	0.975661	6.371985	19.440538
H	1.332834	3.909823	19.254972
H	-0.241787	4.258324	19.962228
H	-0.436014	2.500873	18.201716
H	-2.232376	3.582055	16.851301
H	-2.350994	4.063761	18.540861
H	-2.582226	6.044974	17.043839
H	-0.817337	7.462204	18.092588
H	-1.513604	6.359460	19.275146
H	1.503916	3.213117	16.804048
H	0.046998	3.080085	15.824402
H	1.464214	6.959096	17.066331
H	2.341548	5.507785	17.539749
H	-1.224425	5.181093	15.137438
H	-0.647307	6.765673	15.643983
H	1.264126	5.196379	15.312058
C	2.777827	-2.163146	17.800529
C	1.708931	-1.436454	16.969887
C	1.929496	0.081839	17.054093
C	1.832103	0.529774	18.520489
C	2.901236	-0.191837	19.355436
C	2.678444	-1.709446	19.265027

C	3.324783	0.424444	16.509496
C	4.170810	-1.814896	17.253477
C	4.294066	0.151008	18.804784
C	4.397712	-0.297784	17.339148
H	2.618648	-3.245286	17.739333
H	1.755141	-1.766450	15.925044
H	0.708313	-1.692101	17.339158
H	1.166674	0.597882	16.460723
H	1.968047	1.615729	18.591751
H	0.833448	0.308811	18.916367
H	2.829576	0.128612	20.400532
H	3.423772	-2.236174	19.873311
H	1.694903	-1.970181	19.674471
H	3.401581	0.128578	15.456215
H	3.486986	1.508492	16.546423
H	4.942593	-2.344125	17.825549
H	4.261554	-2.150777	16.213425
H	4.473589	1.230234	18.881344
H	5.068051	-0.342222	19.405314
H	5.391171	-0.052059	16.948114
C	5.102410	-1.171038	23.940686
C	4.026598	-1.017458	22.854581
C	2.967339	-2.119106	23.013631
C	3.639128	-3.494316	22.879676
C	4.714144	-3.654513	23.965762
C	5.768725	-2.548704	23.804538
C	2.317440	-2.003802	24.401121
C	4.446754	-1.057689	25.325681
C	4.060000	-3.534847	25.350865
C	3.389228	-2.159839	25.491091
H	5.856618	-0.384919	23.825367
H	3.556336	-0.029379	22.927291
H	4.485472	-1.077791	21.860223
H	2.201065	-2.007335	22.238636
H	2.889448	-4.289555	22.971500
H	4.090665	-3.597787	21.885562
H	5.192087	-4.635360	23.867999
H	6.553330	-2.663501	24.562287
H	6.258318	-2.635633	22.826929
H	1.815817	-1.033854	24.503147
H	1.545259	-2.773548	24.519698
H	5.208010	-1.145221	26.110426
H	3.983158	-0.070744	25.443059
H	3.317968	-4.330982	25.486429
H	4.814210	-3.667774	26.136016
H	2.922956	-2.076556	26.478970
C	2.882836	4.262801	21.949217
C	3.265965	2.829920	21.548409
C	4.770404	2.612705	21.772981
C	5.562424	3.613855	20.918083
C	5.185297	5.048959	21.317033
C	3.679727	5.259832	21.094014
C	5.101271	2.837603	23.256470
C	3.218286	4.482997	23.432435

C	5.514621	5.267628	22.801776
C	4.722590	4.270679	23.661444
H	1.810076	4.415612	21.788147
H	2.688901	2.106967	22.137533
H	3.013731	2.654261	20.495568
H	5.041211	1.590340	21.487112
H	6.639089	3.455569	21.054445
H	5.350490	3.451317	19.854353
H	5.750706	5.760944	20.705937
H	3.400666	6.287539	21.356517
H	3.434448	5.126846	20.033292
H	4.557964	2.115308	23.877714
H	6.170035	2.666508	23.433397
H	2.930298	5.496658	23.736655
H	2.641200	3.789421	24.055878
H	6.590483	5.138833	22.971336
H	5.268689	6.295669	23.094376
H	4.959062	4.427715	24.719589
C	-0.282635	1.610611	21.560907
C	-1.602914	1.454971	20.790697
C	-2.674558	0.854163	21.713499
C	-2.205076	-0.521738	22.210419
C	-0.886202	-0.371476	22.984306
C	0.180764	0.232840	22.058384
C	-2.888384	1.783853	22.917968
C	-0.502852	2.538769	22.765471
C	-1.104335	0.561685	24.185290
C	-1.571081	1.940265	23.693444
H	0.479969	2.038827	20.901228
H	-1.935092	2.429095	20.411920
H	-1.454079	0.809013	19.916889
H	-3.615124	0.744618	21.162481
H	-2.970930	-0.970212	22.854776
H	-2.067345	-1.202082	21.361291
H	-0.553128	-1.353630	23.337127
H	1.133613	0.327114	22.593321
H	0.361090	-0.434329	21.206711
H	-3.243885	2.764447	22.579014
H	-3.665618	1.375149	23.575008
H	0.438050	2.674615	23.312506
H	-0.816070	3.532358	22.422785
H	-1.850533	0.132193	24.864658
H	-0.174432	0.661264	24.758290
H	-1.726595	2.604015	24.551057
C	-1.413093	-5.081087	19.213526
C	0.049545	-4.614673	19.152435
C	0.685147	-4.714988	20.547748
C	-0.096202	-3.826756	21.528047
C	-1.559397	-4.290905	21.595381
C	-2.189153	-4.192064	20.197317
C	0.630083	-6.173292	21.028484
C	-1.462387	-6.538641	19.697317
C	-1.608852	-5.750421	22.072982
C	-0.831316	-6.643515	21.094173

```
H     -1.864368     -5.008570     18.217934
H      0.611039     -5.228366     18.437706
H      0.099203     -3.580656     18.790087
H      1.728040     -4.382509     20.501718
H      0.360258     -3.874203     22.524164
H     -0.048483     -2.779068     21.207222
H     -2.114617     -3.655946     22.294385
H     -3.240549     -4.502041     20.235345
H     -2.178892     -3.150748     19.853027
H      1.201791     -6.816367     20.348454
H      1.098538     -6.261454     22.016214
H     -2.500830     -6.890531     19.725708
H     -0.927495     -7.187296     18.993119
H     -1.179372     -5.831775     23.078838
H     -2.649989     -6.087805     22.144879
H     -0.867218     -7.683572     21.436579
```

Adamantane$_8$

```
   208
E=-228.5912
   C        1.1179787438        0.9792823608       -4.7517447828
   C        2.2770424839        1.6727192290       -5.4840700623
   C        3.5392522699        0.8003849767       -5.4015300637
   C        3.9166942367        0.5894611781       -3.9272517569
   C        2.7618889512       -0.1067242676       -3.1905989002
   C        1.5015553067        0.7677282235       -3.2791311165
   C        3.2599876947       -0.5620201576       -6.0547176130
   C        0.8449033555       -0.3833804833       -5.4071275409
   C        2.4841952771       -1.4666199508       -3.8496164492
   C        2.1032079921       -1.2609667701       -5.3236589223
   H        0.2192779954        1.6028732245       -4.8115873234
   H        2.0085494520        1.8485328799       -6.5327515226
   H        2.4693605159        2.6556029862       -5.0369619618
   H        4.3641481580        1.2964302090       -5.9247628539
   H        4.8285441336       -0.0157072839       -3.8554338358
   H        4.1380609865        1.5534750493       -3.4534066745
   H        3.0329604347       -0.2555696017       -2.1396879347
   H        0.6740542064        0.2910264318       -2.7397212813
   H        1.6799736317        1.7348117207       -2.7934406197
   H        3.0091047385       -0.4280457301       -7.1139365089
   H        4.1601886768       -1.1874928648       -6.0192867845
   H        0.0052215669       -0.8801714189       -4.9059983430
   H        0.5513822810       -0.2451612515       -6.4547437262
   H        3.3706542048       -2.1081904493       -3.7761599296
   H        1.6745521767       -1.9832013480       -3.3199120403
   H        1.9061221781       -2.2315758843       -5.7919802147
   C        3.1919611951       -0.2288170271        5.2892151201
   C        3.0381538414        0.8713507683        6.3506094289
   C        2.5055176077        0.2618655210        7.6566644885
   C        1.1402815446       -0.3918874596        7.3936334151
   C        1.2889111376       -1.4953780510        6.3348016947
   C        1.8250402512       -0.8814827636        5.0322487562
   C        3.4910552211       -0.8040598065        8.1599628457
```

C	4.1761403422	-1.2931142774	5.7987340339
C	2.2778803648	-2.5566455446	6.8411467849
C	3.6459361621	-1.9084327594	7.1028513418
H	3.5715364305	0.2078024612	4.3589486028
H	4.0035707342	1.3600326918	6.5291926140
H	2.3518083925	1.6473541750	5.9904691831
H	2.3971777953	1.0478983833	8.4121123529
H	0.7399051060	-0.8135778603	8.3235435339
H	0.4209373594	0.3623322701	7.0522367451
H	0.3143429234	-1.9596570447	6.1484945504
H	1.9171207349	-1.6559928431	4.2611320589
H	1.1176316995	-0.1361758899	4.6487521421
H	4.4652439103	-0.3469359617	8.3716944272
H	3.1312374880	-1.2334323122	9.1028327781
H	4.3105698638	-2.0745262108	5.0408941387
H	5.1620428847	-0.8436840366	5.9683438338
H	1.8974421383	-3.0167614088	7.7611349060
H	2.3774838300	-3.3611690212	6.1023733582
H	4.3495348560	-2.6664345207	7.4642817102
C	-2.5528366657	4.7353587276	-3.0355856506
C	-2.1058326131	3.5639607797	-3.9236160461
C	-2.4830791761	2.2326553029	-3.2555859560
C	-1.7887346692	2.1343763165	-1.8885911647
C	-2.2347119351	3.3021280746	-0.9950773353
C	-1.8584697443	4.6307097431	-1.6690644063
C	-4.0057039984	2.1760139700	-3.0576065696
C	-4.0754026069	4.6722408123	-2.8387206271
C	-3.7580063579	3.2431475083	-0.8031670175
C	-4.4573501930	3.3441533424	-2.1673377459
H	-2.2827737993	5.6833953706	-3.5135001846
H	-2.5786792220	3.6375757843	-4.9103929554
H	-1.0222289152	3.6088209720	-4.0871487593
H	-2.1639621614	1.3989318109	-3.8906891926
H	-2.0343815178	1.1786590979	-1.4099272539
H	-0.6996968359	2.1537850819	-2.0169463019
H	-1.7380638722	3.2303316013	-0.0213040003
H	-2.1538369712	5.4729967174	-1.0316661838
H	-0.7705849789	4.6945609892	-1.7931958033
H	-4.5142261709	2.2244412343	-4.0281198742
H	-4.2898632370	1.2213254130	-2.5986332092
H	-4.4102304694	5.5157202035	-2.2227417054
H	-4.5841746112	4.7649294044	-3.8058394004
H	-4.0383854394	2.3071422083	-0.3050709980
H	-4.0869514367	4.0604554757	-0.1498922879
H	-5.5432447549	3.3013894786	-2.0285791213
C	-1.7976939128	-4.3741832258	3.4063098763
C	-0.3104169268	-4.0398469361	3.5981696456
C	0.3774945370	-5.1659757001	4.3852943294
C	-0.2966938413	-5.3128296574	5.7580112554
C	-1.7840945294	-5.6503815623	5.5719877140
C	-2.4664023691	-4.5232165713	4.7814808494
C	0.2429434443	-6.4851592584	3.6091234909
C	-1.9263082956	-5.6952496172	2.6322370862
C	-1.9132772162	-6.9680642042	4.7923990632

C	-1.2431602726	-6.8254787957	3.4173141794
H	-2.2862347580	-3.5700723566	2.8452009007
H	0.1753190840	-3.9104178791	2.6234694864
H	-0.2053790102	-3.0884016860	4.1335866152
H	1.4378412234	-4.9257786747	4.5204125921
H	0.1987714601	-6.1009444960	6.3378785263
H	-0.1908162810	-4.3837081028	6.3310542058
H	-2.2627988432	-5.7540664196	6.5518551389
H	-3.5340198864	-4.7425035372	4.6587139849
H	-2.3993296525	-3.5801194186	5.3374015804
H	0.7383541592	-6.4013157655	2.6342519268
H	0.7471790627	-7.2938200957	4.1519377784
H	-2.9843382487	-5.9349372373	2.4706212581
H	-1.4690382582	-5.5963872546	1.6403251975
H	-1.4466172390	-7.7854517842	5.3553591019
H	-2.9710758218	-7.2310145310	4.6703978890
H	-1.3357836551	-7.7660147863	2.8631818280
C	-1.7914866807	-1.6914300474	-2.3839923031
C	-1.5343550919	-2.8706262116	-3.3347622601
C	-1.9205133703	-4.1878962921	-2.6443748602
C	-1.0776475502	-4.3616648149	-1.3716496255
C	-1.3333636373	-3.1862924274	-0.4156078447
C	-0.9490927119	-1.8715442113	-1.1118254775
C	-3.4092627569	-4.1471500404	-2.2665449586
C	-3.2807549796	-1.6571243897	-2.0073627641
C	-2.8236555817	-3.1477043153	-0.0437543066
C	-3.6709518731	-2.9709431637	-1.3130416044
H	-1.5153518808	-0.7534354860	-2.8779816806
H	-2.1144964986	-2.7444165347	-4.2568462145
H	-0.4772262108	-2.8945973366	-3.6260632608
H	-1.7369065860	-5.0271687057	-3.3242255908
H	-1.3300356025	-5.3089650121	-0.8799388130
H	-0.0127216382	-4.4121447004	-1.6290204223
H	-0.7310590881	-3.3119948185	0.4907456619
H	-1.1083128101	-1.0253621617	-0.4324209301
H	0.1182468200	-1.8777049135	-1.3642679806
H	-4.0245505883	-4.0440380045	-3.1685565255
H	-3.7020607347	-5.0904324587	-1.7897631606
H	-3.4815177000	-0.8066607591	-1.3444878468
H	-3.8928450707	-1.5099162385	-2.9053796513
H	-3.1067932530	-4.0734439549	0.4716651094
H	-3.0159756113	-2.3245432022	0.6550903391
H	-4.7331385006	-2.9441945955	-1.0460326421
C	-2.5821545662	0.7648869466	2.0259201479
C	-2.4141345819	2.1585608851	2.6504407618
C	-2.7500157154	2.0999983463	4.1486756353
C	-4.2016607161	1.6294388620	4.3268563771
C	-4.3745981792	0.2343450632	3.7066041866
C	-4.0341756938	0.2975773644	2.2095870807
C	-1.8050156919	1.1077000363	4.8437363860
C	-1.6371252769	-0.2236605227	2.7263773540
C	-3.4251770272	-0.7531897002	4.4023834211
C	-1.9716176543	-0.2886847059	4.2244901550
H	-2.3424628942	0.8088979135	0.9578748928

H	-1.3862365013	2.5135072781	2.5084184142
H	-3.0705225626	2.8778852897	2.1457828789
H	-2.6290061386	3.0945455402	4.5919805108
H	-4.4601452494	1.6026362014	5.3923723456
H	-4.8889940475	2.3399281873	3.8518480046
H	-5.4103184074	-0.0988543463	3.8343159812
H	-4.1728174266	-0.6886304194	1.7500891844
H	-4.7188085312	0.9841946874	1.6969338084
H	-0.7655864531	1.4425114220	4.7421279816
H	-2.0222602231	1.0712790184	5.9181253204
H	-1.7326368200	-1.2189504407	2.2755195747
H	-0.5950873095	0.0884445090	2.5867128539
H	-3.6701491000	-0.8222805874	5.4691697766
H	-3.5534963813	-1.7581483999	3.9821963126
H	-1.2962419433	-0.9936422565	4.7216342027
C	2.7796780615	-3.3578596971	-0.3941238220
C	2.5649401756	-4.6709198084	0.3742149987
C	3.6158569753	-4.7985861779	1.4878705206
C	5.0204727062	-4.7920764406	0.8654076019
C	5.2417811621	-3.4793645633	0.0981286774
C	4.1864648586	-3.3548650657	-1.0117151776
C	3.4819003713	-3.6103696981	2.4527475349
C	2.6473751279	-2.1735100944	0.5758033435
C	5.1032425791	-2.2944122852	1.0663380223
C	3.6989334973	-2.2945980267	1.6893642840
H	2.0296646038	-3.2689324645	-1.1876485478
H	1.5561197498	-4.6953717144	0.8036509147
H	2.6383379055	-5.5236955022	-0.3115974745
H	3.4605153666	-5.7351446659	2.0346920951
H	5.7796035962	-4.9028561890	1.6491837528
H	5.1366068845	-5.6472943032	0.1886724956
H	6.2435239860	-3.4771690370	-0.3452165994
H	4.3455543801	-2.4305994132	-1.5803883415
H	4.2880503772	-4.1844293578	-1.7219843630
H	2.4899448394	-3.6144527842	2.9205547872
H	4.2147274094	-3.7002036545	3.2637516359
H	2.7783531195	-1.2282341775	0.0351598200
H	1.6404059994	-2.1530549292	1.0098005894
H	5.8640256788	-2.3609382426	1.8534856372
H	5.2792270151	-1.3513951861	0.5346301328
H	3.6022004583	-1.4494449335	2.3797327883
C	1.6342793978	3.0881018736	-0.0563780495
C	1.3316519511	1.6833329953	0.4873358331
C	2.1880557226	1.4136572027	1.7342524261
C	3.6752534586	1.5100150357	1.3608814644
C	3.9841944879	2.9143302195	0.8192532172
C	3.1229113079	3.1803377389	-0.4250848764
C	1.8650486407	2.4620531853	2.8100417718
C	1.3121764718	4.1320008947	1.0241178057
C	3.6562095863	3.9590244222	1.8971094707
C	2.1690503676	3.8688606017	2.2719076808
H	1.0229362739	3.2782288686	-0.9453218276
H	0.2669555528	1.5968756643	0.7356873228
H	1.5399503159	0.9299035101	-0.2820198462

H	1.9702547214	0.4118361245	2.1205216361
H	4.2977818489	1.2995746664	2.2389647007
H	3.9242622565	0.7531681147	0.6072021556
H	5.0448034791	2.9805478021	0.5532225053
H	3.3483360945	4.1729055642	-0.8336903308
H	3.3623989730	2.4533107677	-1.2106141817
H	0.8101157494	2.3907545066	3.1013649048
H	2.4565943009	2.2686134606	3.7130855493
H	1.5047024996	5.1415174752	0.6409635089
H	0.2473396080	4.0894512545	1.2830981727
H	4.2786146829	3.7920018290	2.7845402254
H	3.8917026174	4.9653354396	1.5298953380
H	1.9372000348	4.6140126315	3.0408313306

$Adamantane_9$

234
E=-271.7277

C	4.753925	0.876073	27.228733
C	5.887795	0.581270	28.222721
C	6.132764	-0.933827	28.296372
C	4.847385	-1.635248	28.761406
C	3.710551	-1.346216	27.768947
C	3.472039	0.169959	27.696331
C	6.521021	-1.454918	26.904068
C	5.146228	0.350585	25.839162
C	4.105051	-1.865784	26.377849
C	5.388534	-1.165057	25.906788
H	4.580763	1.956601	27.177907
H	6.804671	1.096436	27.911796
H	5.627417	0.968748	29.215201
H	6.941904	-1.141648	29.005166
H	5.015381	-2.716393	28.837304
H	4.569400	-1.286674	29.763442
H	2.795098	-1.846782	28.102756
H	2.648761	0.388792	27.005467
H	3.169285	0.550510	28.679438
H	7.449357	-0.977305	26.568042
H	6.717446	-2.533048	26.947490
H	4.353010	0.573334	25.115156
H	6.050242	0.860728	25.485224
H	4.259646	-2.951039	26.411604
H	3.292727	-1.683618	25.663769
H	5.668542	-1.537126	24.915118
C	-1.323830	-1.227830	26.358734
C	-2.443493	-2.245665	26.625101
C	-2.274983	-3.457828	25.696007
C	-2.342562	-2.991567	24.233652
C	-1.222709	-1.975488	23.960857
C	-1.393662	-0.767294	24.894662
C	-0.910552	-4.114008	25.957820
C	0.038022	-1.889936	26.619355
C	0.139070	-2.635177	24.227508
C	0.212949	-3.100598	25.689757

H	-1.445941	-0.364454	27.021892
H	-2.418112	-2.567879	27.673083
H	-3.422290	-1.778953	26.460517
H	-3.073962	-4.182428	25.888033
H	-2.243481	-3.851286	23.559914
H	-3.319812	-2.538425	24.027495
H	-1.273161	-1.644073	22.917945
H	-0.611309	-0.024631	24.696317
H	-2.353965	-0.274455	24.699964
H	-0.856105	-4.470530	26.993563
H	-0.786245	-4.992838	25.313585
H	0.845759	-1.166968	26.452397
H	0.108247	-2.206698	27.666966
H	0.282088	-3.488417	23.553457
H	0.948605	-1.925824	24.016710
H	1.184272	-3.571130	25.877856
C	-0.825684	2.931650	25.799652
C	-1.375194	4.245026	26.377291
C	-0.210623	5.173902	26.754010
C	0.673405	4.482107	27.802992
C	1.228293	3.169154	27.229267
C	0.060048	2.245559	26.850953
C	0.626996	5.473748	25.501302
C	0.013120	3.237574	24.549146
C	2.061873	3.473925	25.975076
C	1.181457	4.163204	24.921508
H	-1.657449	2.270871	25.531981
H	-2.026380	4.735747	25.643887
H	-1.991836	4.037996	27.260379
H	-0.604716	6.109850	27.165079
H	1.497884	5.143702	28.095198
H	0.092441	4.279824	28.710966
H	1.857747	2.677444	27.978970
H	0.443055	1.295866	26.458072
H	-0.531608	2.003294	27.742149
H	0.013118	5.986786	24.751114
H	1.451086	6.152025	25.753589
H	0.394637	2.305421	24.114943
H	-0.612474	3.710962	23.782795
H	2.911031	4.117472	26.235069
H	2.480894	2.546076	25.567053
H	1.777563	4.380195	24.028298
C	-4.538568	0.514185	28.585658
C	-3.904058	1.378604	27.485312
C	-4.765265	2.628326	27.245580
C	-6.175332	2.199250	26.811928
C	-6.815938	1.336780	27.910415
C	-5.949286	0.090561	28.148741
C	-4.858501	3.439542	28.547127
C	-4.631847	1.330997	29.883759
C	-6.903575	2.151013	29.210445
C	-5.495440	2.580033	29.650153
H	-3.923234	-0.376239	28.754825
H	-2.887792	1.671431	27.775461

H	-3.814683	0.799979	26.557865
H	-4.310727	3.243407	26.461133
H	-6.794525	3.083518	26.618207
H	-6.125343	1.635464	25.872513
H	-7.820854	1.031598	27.598878
H	-6.406312	-0.544303	28.917532
H	-5.895589	-0.510848	27.233098
H	-3.860006	3.770326	28.857652
H	-5.455372	4.345026	28.383796
H	-5.064755	0.717895	30.683567
H	-3.628927	1.623940	30.217068
H	-7.535869	3.034264	29.059149
H	-7.378109	1.552890	29.997866
H	-5.559957	3.160857	30.576830
C	3.041293	4.346709	30.694392
C	4.017084	3.806021	29.637950
C	5.460576	4.139238	30.045689
C	5.618804	5.663302	30.158131
C	4.647101	6.209091	31.215871
C	3.205713	5.870235	30.805358
C	5.766830	3.494594	31.406362
C	3.353760	3.701002	32.053206
C	4.955018	5.558901	32.573506
C	4.795199	4.034629	32.467215
H	2.012889	4.108314	30.401709
H	3.896018	2.721034	29.533651
H	3.791931	4.245747	28.658715
H	6.154565	3.752337	29.291385
H	6.651201	5.915153	30.429398
H	5.422072	6.135090	29.187787
H	4.761267	7.295793	31.293933
H	2.500059	6.271925	31.542694
H	2.966045	6.346021	29.846627
H	5.677762	2.403782	31.335438
H	6.801573	3.709301	31.699691
H	2.650225	4.063584	32.812528
H	3.221700	2.613963	31.992676
H	5.975588	5.809159	32.887553
H	4.280799	5.955559	33.342308
H	5.015641	3.572771	33.435895
C	6.035538	-1.899862	32.547857
C	4.575000	-1.745812	32.999167
C	4.511333	-0.874206	34.262972
C	5.326733	-1.539971	35.382047
C	6.789354	-1.693710	34.937060
C	6.847174	-2.562882	33.671301
C	5.103729	0.510065	33.956948
C	6.623596	-0.512794	32.245901
C	7.375917	-0.307465	34.628836
C	6.565726	0.361463	33.507956
H	6.078699	-2.521689	31.646981
H	3.979131	-1.291934	32.198126
H	4.137940	-2.731544	33.199823
H	3.469103	-0.765252	34.582615

H	5.272612	-0.936887	36.296496
H	4.902290	-2.521785	35.624419
H	7.368629	-2.169030	35.736159
H	7.888496	-2.697051	33.354106
H	6.450022	-3.563005	33.883437
H	4.518255	1.005927	33.173254
H	5.046383	1.148593	34.846795
H	7.660705	-0.610631	31.902637
H	6.064384	-0.035928	31.431875
H	7.358248	0.317272	35.530063
H	8.426796	-0.401606	34.329244
H	6.985025	1.349852	33.289953
C	0.795392	-1.222110	32.103957
C	1.186340	-1.210383	30.618155
C	0.422089	-0.094469	29.889039
C	-1.088461	-0.342394	30.020269
C	-1.485554	-0.352029	31.504564
C	-0.716131	-1.467016	32.229643
C	0.771869	1.260475	30.523523
C	1.144746	0.135740	32.732561
C	-1.130534	1.003347	32.135159
C	0.379827	1.254746	32.009249
H	1.341119	-2.018430	32.621804
H	2.267228	-1.056678	30.514293
H	0.958973	-2.181692	30.162289
H	0.702776	-0.087589	28.830052
H	-1.645243	0.437263	29.486428
H	-1.355295	-1.298295	29.553463
H	-2.562704	-0.529474	31.595723
H	-1.005481	-1.497673	33.287141
H	-0.976654	-2.442946	31.802249
H	1.845165	1.460147	30.418621
H	0.246911	2.067957	29.998904
H	0.889162	0.133260	33.799177
H	2.224743	0.314533	32.666258
H	-1.688286	1.806722	31.638855
H	-1.427826	1.016700	33.190813
H	0.630974	2.221706	32.458874
C	-0.722527	6.202981	30.968475
C	-2.048810	6.145200	30.195030
C	-3.216169	5.969792	31.178624
C	-3.025149	4.665555	31.967881
C	-1.701294	4.719227	32.745978
C	-0.537837	4.898239	31.758421
C	-3.241574	7.155816	32.155094
C	-0.754498	7.388785	31.945099
C	-1.730245	5.908671	33.718149
C	-1.918050	7.216018	32.933408
H	0.108317	6.327816	30.265464
H	-2.183884	7.062863	29.609896
H	-2.030952	5.313304	29.480479
H	-4.160755	5.929417	30.625077
H	-3.863179	4.518700	32.659977
H	-3.024892	3.808037	31.284129

```
H     -1.566956      3.788225     33.307437
H      0.415771      4.918227     32.299899
H     -0.493232      4.044626     31.071155
H     -3.398188      8.092555     31.606617
H     -4.082937      7.051938     32.850903
H      0.195572      7.453465     32.489322
H     -0.867107      8.329068     31.391974
H     -2.545224      5.783611     34.441328
H     -0.798039      5.946176     34.294821
H     -1.938989      8.062838     33.628179
C      0.269200      3.674821     36.574760
C      1.208557      4.796179     37.044598
C      2.624548      4.235683     37.248973
C      2.583686      3.123172     38.307791
C      1.647787      1.997323     37.841460
C      0.234384      2.563614     37.635177
C      3.138938      3.656103     35.922217
C      0.789693      3.097182     35.249480
C      2.164293      1.423681     36.512968
C      2.204177      2.531825     35.449636
H     -0.739675      4.076434     36.429894
H      1.227118      5.606313     36.305633
H      0.836972      5.229687     37.981053
H      3.292675      5.036973     37.583380
H      3.592709      2.728241     38.477376
H      2.236591      3.527846     39.266237
H      1.619784      1.205599     38.598055
H     -0.448814      1.765198     37.320951
H     -0.154546      2.958047     38.581821
H      3.192912      4.444711     35.161940
H      4.157347      3.269800     36.050457
H      0.115916      2.308777     34.892494
H      0.801696      3.876602     34.478041
H      3.165785      0.998480     36.650938
H      1.515669      0.604630     36.179190
H      2.573159      2.121126     34.503365
```

Adamantane$_{10}$

```
         260
E=-316.6993
C      4.793703      0.902316     27.195344
C      5.922731      0.620432     28.198550
C      6.179833     -0.892295     28.279229
C      4.897018     -1.602898     28.737352
C      3.765065     -1.326804     27.735673
C      3.514402      0.187058     27.656072
C      6.582573     -1.414846     26.891594
C      5.200507      0.375411     25.810487
C      4.174026     -1.847776     26.349284
C      5.454995     -1.137903     25.885123
H      4.611876      1.981172     27.139518
H      6.837502      1.142217     27.892504
H      5.651893      1.009087     29.187765
```

H	6.985499	-1.090899	28.994593
H	5.073502	-2.682329	28.818215
H	4.608831	-1.253260	29.736130
H	2.851427	-1.833900	28.064563
H	2.694376	0.396615	26.958498
H	3.201318	0.568399	28.635631
H	7.509301	-0.930601	26.560660
H	6.787696	-2.491134	26.940177
H	4.410748	0.589024	25.079976
H	6.102772	0.891919	25.461352
H	4.337452	-2.931574	26.387923
H	3.365429	-1.674871	25.628701
H	5.745326	-1.510984	24.896807
C	-1.327243	-1.233834	26.369671
C	-2.435672	-2.256173	26.664273
C	-2.284942	-3.468386	25.732192
C	-2.390492	-3.003632	24.271607
C	-1.282024	-1.983051	23.970576
C	-1.435063	-0.774816	24.907428
C	-0.911702	-4.118570	25.960800
C	0.043415	-1.889956	26.597155
C	0.088699	-2.636752	24.204088
C	0.200541	-3.100640	25.664412
H	-1.436655	-0.370430	27.035007
H	-2.383123	-2.577406	27.711546
H	-3.420200	-1.793744	26.523505
H	-3.075865	-4.196199	25.944365
H	-2.304379	-3.863484	23.596258
H	-3.374438	-2.554799	24.089250
H	-1.359550	-1.652721	22.928981
H	-0.660994	-0.029015	24.689335
H	-2.401955	-0.286206	24.736082
H	-0.830251	-4.473997	26.995147
H	-0.799563	-4.997402	25.314339
H	0.843716	-1.163717	26.409828
H	0.140761	-2.205546	27.642949
H	0.218700	-3.489940	23.527340
H	0.889776	-1.924156	23.972900
H	1.178192	-3.566902	25.828874
C	-0.855803	2.907079	25.814499
C	-1.408098	4.213334	26.405477
C	-0.246442	5.153479	26.762740
C	0.664125	4.468598	27.793397
C	1.221865	3.162814	27.206234
C	0.056452	2.227914	26.847457
C	0.564530	5.464889	25.495405
C	-0.043668	3.224574	24.549359
C	2.028720	3.479109	25.937504
C	1.121705	4.161523	24.902188
H	-1.685486	2.238248	25.560729
H	-2.077904	4.698874	25.685518
H	-2.005994	3.997878	27.299373
H	-0.642530	6.084327	27.183362
H	1.486973	5.138113	28.071835

H	0.102312	4.258205	28.711524
H	1.870259	2.676019	27.942891
H	0.441937	1.283165	26.445193
H	-0.515947	1.977469	27.748909
H	-0.068522	5.973173	24.758026
H	1.386072	6.151171	25.733979
H	0.339376	2.297441	24.105851
H	-0.688322	3.693125	23.795940
H	2.875830	4.130908	26.183230
H	2.449703	2.556613	25.519485
H	1.698778	4.386747	23.998578
C	-4.512958	0.479106	28.751527
C	-3.950686	1.344810	27.613549
C	-4.842308	2.579789	27.411582
C	-6.266596	2.126614	27.055935
C	-6.835083	1.262754	28.192366
C	-5.938151	0.031349	28.392619
C	-4.879055	3.399017	28.710930
C	-4.550015	1.303903	30.047398
C	-6.866366	2.085071	29.489883
C	-5.443686	2.538206	29.851679
H	-3.876043	-0.400801	28.893698
H	-2.925102	1.654844	27.847617
H	-3.901589	0.760817	26.686460
H	-4.439254	3.195819	26.600199
H	-6.908567	3.000127	26.889490
H	-6.257684	1.556784	26.118882
H	-7.850275	0.940380	27.936420
H	-6.344214	-0.604613	29.188628
H	-5.923708	-0.575870	27.479352
H	-3.870718	3.746887	28.966036
H	-5.497457	4.294298	28.573511
H	-4.930691	0.690286	30.872963
H	-3.535481	1.614170	30.325269
H	-7.519162	2.957683	29.366501
H	-7.289486	1.485721	30.305182
H	-5.468036	3.124761	30.776670
C	3.052672	4.354554	30.706068
C	4.033397	3.819268	29.651443
C	5.474078	4.162998	30.060395
C	5.621625	5.688264	30.171036
C	4.644952	6.228681	31.226955
C	3.206416	5.879323	30.815239
C	5.783222	3.522249	31.422255
C	3.368038	3.712783	32.066075
C	4.955799	5.582393	32.585786
C	4.806656	4.056915	32.481295
H	2.026281	4.108667	30.412521
H	3.919964	2.733335	29.548418
H	3.806333	4.256159	28.671382
H	6.171595	3.779935	29.307389
H	6.651941	5.947607	30.443153
H	5.422748	6.157424	29.199855
H	4.751503	7.316247	31.303734

H	2.497152	6.277072	31.551246
H	2.964564	6.352200	29.855617
H	5.701787	2.430755	31.352648
H	6.816115	3.744493	31.716485
H	2.661138	4.071467	32.824122
H	3.243575	2.624778	32.006810
H	5.974251	5.840116	32.900671
H	4.277967	5.975368	33.353301
H	5.029176	3.597847	33.450826
C	6.102244	-1.851065	32.564137
C	4.634507	-1.730929	33.002201
C	4.540356	-0.870074	34.271460
C	5.358737	-1.526747	35.393725
C	6.828488	-1.646575	34.962007
C	6.916763	-2.505104	33.690755
C	5.106456	0.528492	33.981058
C	6.663909	-0.449823	32.277780
C	7.388737	-0.246141	34.669363
C	6.575514	0.413816	33.545364
H	6.167148	-2.465275	31.659348
H	4.036984	-1.283809	32.198595
H	4.216344	-2.727034	33.191585
H	3.493032	-0.785303	34.581644
H	5.283140	-0.931604	36.311847
H	4.952702	-2.518974	35.624974
H	7.409891	-2.115465	35.763356
H	7.963684	-2.615122	33.382831
H	6.538688	-3.514844	33.891907
H	4.518275	1.017677	33.195197
H	5.027127	1.159182	34.874798
H	7.706107	-0.523416	31.944006
H	6.102676	0.021142	31.461712
H	7.349254	0.371510	35.574772
H	8.444202	-0.316068	34.379418
H	6.976033	1.412331	33.338480
C	0.849095	-1.222733	32.079058
C	1.234388	-1.229207	30.591748
C	0.489055	-0.103915	29.857480
C	-1.025489	-0.321545	29.996187
C	-1.416892	-0.312871	31.481999
C	-0.666386	-1.437365	32.212203
C	0.867495	1.248487	30.480844
C	1.227141	0.132540	32.696523
C	-1.033221	1.039834	32.101452
C	0.481224	1.260979	31.968040
H	1.381312	-2.025770	32.600580
H	2.317637	-1.097246	30.482707
H	0.986469	-2.199155	30.143765
H	0.765683	-0.110047	28.797421
H	-1.569145	0.464933	29.458834
H	-1.312613	-1.275401	29.537303
H	-2.496913	-0.468730	31.578494
H	-0.952141	-1.454848	33.270978
H	-0.947434	-2.411087	31.792850

H	1.944039	1.426545	30.370461
H	0.356232	2.062232	29.952392
H	0.975727	0.142640	33.764084
H	2.310133	0.289870	32.624862
H	-1.577240	1.850312	31.601466
H	-1.326072	1.066494	33.158094
H	0.752811	2.226073	32.409723
C	-0.684812	6.228579	30.924130
C	-2.016045	6.183977	30.158355
C	-3.179818	6.025783	31.149093
C	-2.999838	4.722032	31.941739
C	-1.671054	4.762569	32.712193
C	-0.511163	4.924399	31.717502
C	-3.185604	7.215307	32.121610
C	-0.697165	7.417962	31.896839
C	-1.680370	5.955542	33.680420
C	-1.857073	7.262380	32.892260
H	0.143463	6.341151	30.216035
H	-2.143520	7.101206	29.570841
H	-2.012116	5.349525	29.446578
H	-4.127936	5.994786	30.601006
H	-3.835621	4.587490	32.639035
H	-3.013638	3.862274	31.260946
H	-1.544608	3.831931	33.276083
H	0.445665	4.934864	32.253539
H	-0.480593	4.068006	31.032933
H	-3.334183	8.151992	31.570808
H	-4.024189	7.123792	32.822490
H	0.256673	7.473165	32.435487
H	-0.801727	8.357649	31.341129
H	-2.492670	5.842623	34.408594
H	-0.744535	5.983895	34.251710
H	-1.864017	8.111724	33.584225
C	-4.864323	-0.141331	35.457435
C	-6.317428	0.357000	35.430303
C	-6.340603	1.878355	35.216076
C	-5.583793	2.565471	36.363054
C	-4.128669	2.072894	36.392877
C	-4.111146	0.550988	36.603688
C	-5.656528	2.214994	33.882095
C	-4.184447	0.200275	34.122511
C	-3.450260	2.408556	35.055780
C	-4.201645	1.721152	33.905502
H	-4.850053	-1.225991	35.610468
H	-6.872792	-0.144795	34.628739
H	-6.819523	0.102125	36.371521
H	-7.377562	2.231371	35.196504
H	-5.610375	3.654052	36.232128
H	-6.073567	2.349410	37.320286
H	-3.591220	2.563634	37.211638
H	-3.076747	0.188871	36.646782
H	-4.574490	0.299211	37.565405
H	-6.199033	1.747341	33.051537
H	-5.683738	3.297537	33.708331

```
H     -3.151588     -0.168775     34.121255
H     -4.701590     -0.303469     33.296942
H     -3.438908      3.494381     34.901921
H     -2.403967      2.079947     35.071687
H     -3.716885      1.962031     32.953013
C      0.069211      3.633581     36.458973
C      0.964567      4.756200     37.005461
C      2.365390      4.202108     37.308167
C      2.254387      3.080213     38.351947
C      1.362252      1.953094     37.809172
C     -0.035888      2.513011     37.504801
C      2.979010      3.637060     36.017825
C      0.688798      3.070496     35.170566
C      1.978071      1.394011     36.517303
C      2.088458      2.511572     35.468914
H     -0.928927      4.030624     36.244064
H      1.031893      5.572823     36.276541
H      0.522810      5.179400     37.915793
H      3.002181      5.004292     37.697122
H      3.250824      2.689638     38.591575
H      1.835631      3.474531     39.285763
H      1.284104      1.154672     38.555137
H     -0.689202      1.713439     37.134894
H     -0.495439      2.896951     38.423704
H      3.083348      4.432548     35.270063
H      3.987774      3.255525     36.216976
H      0.047940      2.281360     34.758786
H      0.752151      3.856652     34.408527
H      2.969500      0.973398     36.724601
H      1.360799      0.574174     36.130255
H      2.528191      2.111250     34.548791
```

Adamantane$_{11}$

```
        286
E=-361.7021
C     -0.449827     -5.667768     33.692940
C     -1.578517     -6.503625     34.315825
C     -2.878164     -6.293299     33.523592
C     -3.261223     -4.805882     33.559680
C     -2.136900     -3.965344     32.934940
C     -0.839047     -4.181926     33.728422
C     -2.661421     -6.727194     32.065666
C     -0.239283     -6.103820     32.234696
C     -1.921628     -4.405393     31.478661
C     -1.535261     -5.891748     31.437249
H      0.475638     -5.818965     34.259300
H     -1.304879     -7.565630     34.315599
H     -1.725795     -6.214391     35.363491
H     -3.681335     -6.890581     33.969077
H     -4.198975     -4.645755     33.013935
H     -3.438301     -4.487453     34.594138
H     -2.411907     -2.905309     32.962243
H     -0.032373     -3.571633     33.304410
```

H	-0.973054	-3.852070	34.765831
H	-2.407265	-7.793236	32.023299
H	-3.588525	-6.599832	31.493747
H	0.578575	-5.528194	31.784406
H	0.057685	-7.158857	32.196416
H	-2.835633	-4.237915	30.896112
H	-1.134595	-3.798619	31.014581
H	-1.382722	-6.203813	30.398212
C	-5.931633	-5.320268	36.510100
C	-7.316266	-5.739095	37.027748
C	-7.325626	-5.713791	38.564050
C	-6.262889	-6.687675	39.095641
C	-4.875340	-6.271540	38.583347
C	-4.872351	-6.295022	37.046993
C	-7.002476	-4.293022	39.051677
C	-5.612550	-3.900285	37.002828
C	-4.558514	-4.849192	39.070462
C	-5.616906	-3.870668	38.538913
H	-5.927052	-5.339196	35.414778
H	-8.085448	-5.063101	36.635165
H	-7.563787	-6.744757	36.666736
H	-8.313896	-6.012175	38.930896
H	-6.273332	-6.693637	40.192324
H	-6.492550	-7.709799	38.771027
H	-4.119395	-6.967514	38.963104
H	-3.880768	-6.018793	36.668277
H	-5.076991	-7.310307	36.686026
H	-7.765347	-3.589744	38.696342
H	-7.025083	-4.257470	40.147605
H	-4.634449	-3.581236	36.622668
H	-6.351315	-3.190893	36.610721
H	-4.538571	-4.822766	40.166693
H	-3.561102	-4.547592	38.728159
H	-5.389696	-2.857253	38.887647
C	-1.986305	5.222574	33.741010
C	-3.130246	4.927352	32.758753
C	-4.480082	5.228508	33.428230
C	-4.636325	4.347983	34.677515
C	-3.496239	4.641469	35.664924
C	-2.148896	4.342205	34.989605
C	-4.525024	6.708040	33.840243
C	-2.037679	6.702320	34.151691
C	-3.543291	6.122571	36.070950
C	-3.384363	7.007594	34.825268
H	-1.025010	5.007025	33.261885
H	-3.015142	5.534843	31.852973
H	-3.092896	3.877567	32.443301
H	-5.294165	5.017867	32.725958
H	-5.605624	4.539396	35.153622
H	-4.625748	3.288243	34.395212
H	-3.609350	4.012249	36.554494
H	-1.326633	4.528867	35.691037
H	-2.094048	3.282230	34.713159
H	-4.435237	7.348954	32.954803

H	-5.492107	6.940358	34.302426
H	-1.212962	6.931069	34.837631
H	-1.903783	7.342225	33.271151
H	-4.493166	6.345239	36.571923
H	-2.746148	6.340631	36.792045
H	-3.418694	8.063145	35.116615
C	-2.884125	-2.327620	41.549575
C	-4.292023	-1.754519	41.773095
C	-4.383306	-1.134090	43.175838
C	-4.095653	-2.214252	44.229856
C	-2.687224	-2.788821	44.012815
C	-2.600141	-3.404661	42.607787
C	-3.344098	-0.009587	43.303627
C	-1.849029	-1.199700	41.681186
C	-1.652286	-1.660118	44.137979
C	-1.933743	-0.578538	43.083839
H	-2.821265	-2.769595	40.549167
H	-4.516078	-0.998074	41.011306
H	-5.042030	-2.547187	41.663010
H	-5.387703	-0.725635	43.332806
H	-4.177514	-1.788033	45.237076
H	-4.842629	-3.014490	44.162762
H	-2.484545	-3.559027	44.764980
H	-1.604814	-3.836502	42.447152
H	-3.320368	-4.226296	42.512040
H	-3.549887	0.779220	42.569958
H	-3.412390	0.454526	44.294974
H	-0.840433	-1.592246	41.503313
H	-2.029205	-0.432553	40.918440
H	-1.690806	-1.224321	45.143671
H	-0.640120	-2.061158	44.005114
H	-1.195251	0.225562	43.174947
C	-0.772920	2.276361	30.515273
C	0.385483	2.055807	29.530366
C	0.966282	0.645875	29.719357
C	1.481289	0.496308	31.159095
C	0.325829	0.712910	32.148490
C	-0.253605	2.122518	31.953022
C	-0.133081	-0.396188	29.461879
C	-1.867847	1.230205	30.255195
C	-0.772173	-0.328962	31.884619
C	-1.292846	-0.181577	30.446765
H	-1.185355	3.282069	30.379044
H	0.032946	2.182962	28.499695
H	1.165895	2.808954	29.693704
H	1.791620	0.490621	29.015795
H	1.916921	-0.500158	31.300997
H	2.281104	1.221950	31.350394
H	0.695398	0.606355	33.174216
H	-1.067198	2.297423	32.667472
H	0.515374	2.877049	32.158773
H	-0.495942	-0.314464	28.430138
H	0.274272	-1.408173	29.574946
H	-2.710448	1.389767	30.938979

H	-2.260713	1.341549	29.237346
H	-0.376404	-1.339981	32.039623
H	-1.594658	-0.197918	32.598279
H	-2.075178	-0.925510	30.260301
C	-1.267174	-0.947542	35.534197
C	-2.782667	-1.187665	35.455430
C	-3.467959	-0.590720	36.694375
C	-2.907307	-1.260349	37.958382
C	-1.392004	-1.020627	38.043800
C	-0.712054	-1.616110	36.801216
C	-3.188867	0.919087	36.752534
C	-0.994055	0.563308	35.594890
C	-1.118045	0.490246	38.098051
C	-1.674190	1.164334	36.834503
H	-0.780570	-1.374031	34.650210
H	-3.189240	-0.732700	34.544146
H	-2.989804	-2.262885	35.392590
H	-4.548388	-0.762573	36.635829
H	-3.403367	-0.856617	38.849327
H	-3.117204	-2.336653	37.939200
H	-0.994398	-1.499336	38.945352
H	0.373298	-1.469120	36.859269
H	-0.882710	-2.698836	36.761706
H	-3.602342	1.413463	35.865141
H	-3.689230	1.360626	37.622887
H	0.086327	0.749016	35.630725
H	-1.369019	1.050405	34.686651
H	-1.582261	0.924826	38.991618
H	-0.039933	0.674546	38.179748
H	-1.478430	2.241493	36.875203
C	3.111518	5.339487	35.258547
C	4.231299	4.397831	34.789550
C	3.943774	2.968005	35.273162
C	3.871195	2.954665	36.807764
C	2.750532	3.892675	37.282270
C	3.040886	5.320063	36.793184
C	2.599988	2.496237	34.696565
C	1.770053	4.861591	34.681455
C	1.409805	3.418936	36.700241
C	1.476125	3.433365	35.165432
H	3.318531	6.358221	34.912954
H	4.306482	4.417528	33.695616
H	5.197578	4.739643	35.179963
H	4.743073	2.298282	34.937186
H	3.686082	1.934943	37.166591
H	4.831407	3.270807	37.233091
H	2.700992	3.881992	38.376564
H	2.257850	6.004126	37.142296
H	3.986233	5.678317	37.218569
H	2.644304	2.481068	33.600827
H	2.392406	1.468799	35.019231
H	0.964572	5.537973	34.992428
H	1.800710	4.888659	33.585514
H	1.181096	2.407531	37.057411

H	0.597776	4.068916	37.048235
H	0.519604	3.094818	34.752270
C	3.682772	-1.132530	33.948770
C	2.294742	-1.633170	34.376927
C	2.434780	-2.954033	35.149368
C	3.102824	-4.002928	34.247157
C	4.493033	-3.508559	33.818408
C	4.347782	-2.186073	33.049722
C	3.305683	-2.725679	36.394427
C	4.550087	-0.908541	35.197185
C	5.358634	-3.278304	35.066788
C	4.696332	-2.227677	35.971102
H	3.580667	-0.191008	33.398115
H	1.799783	-0.881173	35.003224
H	1.659497	-1.778983	33.494797
H	1.444053	-3.308818	35.454120
H	3.190384	-4.957028	34.780902
H	2.481261	-4.190263	33.363178
H	4.967172	-4.257633	33.174880
H	5.332258	-1.832057	32.720413
H	3.748582	-2.341268	32.144265
H	2.830074	-1.993522	37.058188
H	3.397367	-3.657714	36.965160
H	5.537894	-0.531087	34.906046
H	4.095689	-0.144132	35.839026
H	5.486265	-4.219671	35.614836
H	6.361228	-2.944276	34.773197
H	5.314580	-2.065231	36.860838
C	0.283523	-6.836380	37.898646
C	0.702741	-5.433238	37.433853
C	0.851186	-4.506210	38.650215
C	-0.491428	-4.421184	39.392245
C	-0.914037	-5.821719	39.862148
C	-1.057578	-6.745499	38.642734
C	1.919787	-5.075144	39.596364
C	1.354016	-7.399224	38.846380
C	0.159201	-6.387603	40.804840
C	1.503442	-6.477555	40.066484
H	0.178045	-7.495551	37.030039
H	1.649464	-5.486198	36.882816
H	-0.044974	-5.028004	36.741278
H	1.150657	-3.506574	38.316550
H	-0.403776	-3.745905	40.251996
H	-1.259604	-3.997806	38.733829
H	-1.871666	-5.758760	40.390382
H	-1.378465	-7.744255	38.962864
H	-1.836092	-6.363530	37.971174
H	2.889191	-5.122171	39.085600
H	2.048893	-4.411622	40.460021
H	1.076328	-8.409907	39.169498
H	2.312920	-7.486987	38.321442
H	0.258123	-5.747204	41.689669
H	-0.140433	-7.379737	41.163803
H	2.267472	-6.880686	40.740275

C	3.900974	0.979820	40.238826
C	4.188312	-0.321238	39.473797
C	3.362788	-1.469682	40.074245
C	3.740720	-1.649455	41.552520
C	3.452393	-0.351821	42.323181
C	4.277374	0.793856	41.716737
C	1.867795	-1.130892	39.969318
C	2.404576	1.312266	40.132915
C	1.957294	-0.015517	42.212208
C	1.574799	0.168324	40.735676
H	4.490598	1.797140	39.809361
H	3.943084	-0.195979	38.412239
H	5.257647	-0.559418	39.526211
H	3.568786	-2.396130	39.526937
H	3.172123	-2.480251	41.987643
H	4.802082	-1.911298	41.640869
H	3.723079	-0.482117	43.376652
H	4.095671	1.722788	42.270844
H	5.348315	0.575511	41.808335
H	1.579218	-1.019991	38.917054
H	1.266626	-1.952344	40.377538
H	2.189766	2.250825	40.658236
H	2.126542	1.466209	39.083269
H	1.357229	-0.817325	42.659246
H	1.734357	0.898709	42.775639
H	0.508454	0.407493	40.658494
C	-2.668076	5.069317	40.027710
C	-1.796270	6.302273	40.311180
C	-0.532992	5.879855	41.077109
C	-0.938885	5.222112	42.404917
C	-1.808645	3.986294	42.127107
C	-3.068465	4.413481	41.358141
C	0.261200	4.871722	40.232290
C	-1.868241	4.063642	39.185197
C	-1.011482	2.983117	41.279225
C	-0.605706	3.635142	39.948641
H	-3.568157	5.372668	39.481870
H	-1.519838	6.792566	39.369941
H	-2.363042	7.036461	40.896577
H	0.087330	6.760353	41.277632
H	-0.044574	4.933889	42.970595
H	-1.490178	5.937778	43.026811
H	-2.097270	3.519337	43.075103
H	-3.708320	3.542636	41.170576
H	-3.657749	5.114553	41.961649
H	0.575515	5.334942	39.289173
H	1.176033	4.576854	40.760462
H	-2.487059	3.186934	38.958384
H	-1.592129	4.513477	38.223859
H	-0.118616	2.655140	41.825154
H	-1.614648	2.086647	41.090818
H	-0.037026	2.918652	39.345736

Adamantane$_{12}$

```
         312
E=-413.4912
C     -0.536122     -5.584138     33.561152
C     -1.738791     -6.381207     34.089217
C     -2.987160     -6.049642     33.256995
C     -3.283048     -4.545573     33.360309
C     -2.084376     -3.743473     32.830474
C     -0.838220     -4.081229     33.663519
C     -2.731610     -6.417689     31.787292
C     -0.286868     -5.953938     32.090755
C     -1.830729     -4.117651     31.362009
C     -1.531242     -5.620580     31.253581
H      0.352644     -5.821782     34.155904
H     -1.528531     -7.456471     34.040937
H     -1.915309     -6.139737     35.144435
H     -3.843221     -6.619434     33.634862
H     -4.184461     -4.299375     32.786115
H     -3.486974     -4.272474     34.402751
H     -2.297323     -2.671532     32.905599
H      0.021143     -3.499829     33.307944
H     -0.998651     -3.799539     34.711322
H     -2.539138     -7.493535     31.695943
H     -3.623169     -6.203406     31.185596
H      0.582776     -5.406375     31.707492
H     -0.051235     -7.021659     32.005866
H     -2.706386     -3.863620     30.752491
H     -0.989373     -3.536418     30.965457
H     -1.351208     -5.885589     30.205913
C     -6.059888     -4.901995     36.098016
C     -7.504784     -5.219602     36.512726
C     -7.603489     -5.281384     38.044847
C     -6.665535     -6.378345     38.571504
C     -5.218299     -6.063991     38.162120
C     -5.125763     -5.999804     36.629715
C     -7.184487     -3.926877     38.636909
C     -5.645304     -3.548184     36.694893
C     -4.805413     -4.707298     38.753427
C     -5.738635     -3.606130     38.227338
H     -5.991675     -4.858634     35.005507
H     -8.185762     -4.453908     36.121941
H     -7.819099     -6.175714     36.076828
H     -8.634566     -5.507376     38.338265
H     -6.741879     -6.445982     39.663499
H     -6.965733     -7.354557     38.171820
H     -4.551499     -6.847585     38.538159
H     -4.092661     -5.795463     36.323266
H     -5.398305     -6.969459     36.195586
H     -7.858977     -3.136775     38.285288
H     -7.269174     -3.952076     39.730082
H     -4.621698     -3.299946     36.388944
H     -6.293088     -2.752136     36.308314
H     -4.848628     -4.745530     39.848639
H     -3.766424     -4.480359     38.485164
```

H	-5.443090	-2.639727	38.650383
C	-5.563070	-1.338486	33.279878
C	-5.921157	-0.028816	33.998991
C	-6.694457	0.893322	33.043679
C	-5.821880	1.203070	31.817664
C	-5.463283	-0.103376	31.092740
C	-4.693485	-1.023139	32.053152
C	-7.981344	0.189015	32.586879
C	-6.853117	-2.037031	32.822975
C	-6.753236	-0.805471	30.641276
C	-7.628201	-1.120247	31.864222
H	-5.011691	-1.994281	33.962538
H	-6.525453	-0.240997	34.889237
H	-5.008565	0.470309	34.346844
H	-6.949206	1.826178	33.558624
H	-6.355612	1.877318	31.136946
H	-4.907949	1.724111	32.127666
H	-4.840773	0.119815	30.219404
H	-4.413902	-1.952141	31.541533
H	-3.759316	-0.541714	32.367056
H	-8.623926	-0.019838	33.450768
H	-8.552305	0.845331	31.918983
H	-6.611909	-2.984425	32.325723
H	-7.474962	-2.284837	33.691686
H	-7.303255	-0.166808	29.939560
H	-6.510134	-1.730279	30.104064
H	-8.547624	-1.620276	31.540367
C	-2.013078	5.102755	33.717627
C	-3.113972	4.658774	32.742180
C	-4.493939	4.885012	33.378938
C	-4.596578	4.066998	34.675381
C	-3.499463	4.509227	35.656032
C	-2.122047	4.284332	35.013308
C	-4.666038	6.376794	33.704145
C	-2.191585	6.594142	34.041426
C	-3.673702	6.002056	35.975150
C	-3.568809	6.824971	34.682071
H	-1.030202	4.940430	33.261870
H	-3.035208	5.221248	31.804016
H	-2.986067	3.599436	32.488417
H	-5.277292	4.568325	32.681548
H	-5.585410	4.204476	35.129438
H	-4.495042	2.997428	34.455006
H	-3.574216	3.924375	36.579293
H	-1.328566	4.577788	35.711332
H	-1.976463	3.218515	34.799256
H	-4.615547	6.971588	32.784086
H	-5.655796	6.554363	34.141958
H	-1.398866	6.928796	34.721574
H	-2.097129	7.191853	33.126779
H	-4.646112	6.173647	36.452426
H	-2.908121	6.325795	36.690723
H	-3.693811	7.888926	34.911468
C	-2.983888	-2.391530	41.507831

C	-4.373308	-1.764377	41.699591
C	-4.484938	-1.172017	43.112956
C	-4.277476	-2.286599	44.149833
C	-2.887801	-2.915385	43.964530
C	-2.780099	-3.502932	42.548856
C	-3.404586	-0.095704	43.300524
C	-1.907627	-1.311715	41.699166
C	-1.811451	-1.834638	44.149438
C	-2.012663	-0.718900	43.112670
H	-2.906494	-2.813392	40.499787
H	-4.540392	-0.982342	40.949100
H	-5.151633	-2.521890	41.546823
H	-5.476087	-0.724945	43.247259
H	-4.374725	-1.879820	45.163703
H	-5.054508	-3.052758	44.039942
H	-2.742318	-3.710044	44.704395
H	-1.798860	-3.972898	42.410045
H	-3.530365	-4.290960	42.410686
H	-3.553010	0.717255	42.579480
H	-3.486184	0.348703	44.299863
H	-0.910931	-1.742609	41.544486
H	-2.030415	-0.520830	40.949378
H	-1.864971	-1.420096	45.163394
H	-0.813031	-2.275169	44.039642
H	-1.244734	0.050943	43.246376
C	-0.654690	2.310489	30.612130
C	0.467351	2.087836	29.586443
C	1.052133	0.676934	29.754784
C	1.618071	0.526654	31.175199
C	0.499124	0.745350	32.205265
C	-0.084526	2.155925	32.030403
C	-0.057516	-0.363276	29.536993
C	-1.760006	1.266179	30.391583
C	-0.609394	-0.294671	31.981037
C	-1.180929	-0.146564	30.562708
H	-1.070019	3.316886	30.490566
H	0.078379	2.215474	28.569025
H	1.254392	2.839665	29.721619
H	1.851502	0.520184	29.022168
H	2.056800	-0.470540	31.301600
H	2.425456	1.250948	31.337627
H	0.904976	0.638283	33.217129
H	-0.871735	2.332307	32.773477
H	0.692609	2.909162	32.208373
H	-0.456922	-0.281051	28.518882
H	0.351894	-1.375943	29.635592
H	-2.577327	1.427263	31.105050
H	-2.188850	1.378076	29.388423
H	-0.210058	-1.306348	32.121962
H	-1.405591	-0.162135	32.723647
H	-1.970702	-0.889178	30.404489
C	-1.082201	-0.931080	35.601343
C	-2.596329	-1.153633	35.465437
C	-3.318588	-0.571891	36.690538

C	-2.809253	-1.270131	37.960769
C	-1.295552	-1.048096	38.103306
C	-0.578490	-1.628255	36.874410
C	-3.026643	0.933571	36.786153
C	-0.796303	0.575444	35.699301
C	-1.008589	0.458557	38.194857
C	-1.513392	1.161199	36.925323
H	-0.569224	-1.346696	34.727142
H	-2.966180	-0.678010	34.549072
H	-2.811863	-2.225283	35.375648
H	-4.397966	-0.731175	36.591247
H	-3.332149	-0.877292	38.841175
H	-3.029085	-2.343624	37.914487
H	-0.934558	-1.547187	39.009197
H	0.505578	-1.493871	36.972923
H	-0.758459	-2.708217	36.809065
H	-3.403835	1.448239	35.894145
H	-3.552741	1.364626	37.646528
H	0.283962	0.748990	35.776151
H	-1.134353	1.082801	34.787671
H	-1.499447	0.881852	39.079559
H	0.067787	0.629892	38.317429
H	-1.308432	2.235308	36.992629
C	3.210522	5.326270	35.427065
C	4.339185	4.387268	34.974320
C	4.046058	2.956010	35.450273
C	3.948672	2.939368	36.983468
C	2.819044	3.874713	37.441700
C	3.115093	5.303548	36.960314
C	2.712502	2.483435	34.851030
C	1.879305	4.847571	34.827353
C	1.488638	3.400182	36.837071
C	1.579767	3.417904	35.303570
H	3.421544	6.346031	35.086964
H	4.432032	4.409355	33.881789
H	5.298495	4.729712	35.381025
H	4.851712	2.288184	35.125902
H	3.759335	1.918626	37.337153
H	4.901390	3.256060	37.424932
H	2.751816	3.861678	38.535024
H	2.325465	5.985711	37.298112
H	4.052883	5.662328	37.401682
H	2.774569	2.470613	33.756124
H	2.501296	1.455018	35.168180
H	1.067864	5.522100	35.126637
H	1.927654	4.876967	33.732110
H	1.255725	2.387695	37.188411
H	0.670091	4.048221	37.173211
H	0.630576	3.078787	34.874283
C	3.807518	-1.188701	34.073342
C	2.394948	-1.660203	34.451384
C	2.477658	-2.999611	35.199782
C	3.143612	-4.047818	34.295231
C	4.558211	-3.582630	33.916527

C	4.470226	-2.241535	33.171767
C	3.317889	-2.819892	36.473557
C	4.643951	-1.013315	35.350278
C	5.393098	-3.400935	35.193510
C	4.732867	-2.351194	36.100364
H	3.746280	-0.233897	33.539760
H	1.902094	-0.907854	35.078918
H	1.781870	-1.771075	33.548725
H	1.469507	-3.333583	35.468818
H	3.190474	-5.014461	34.811238
H	2.543164	-4.200522	33.390246
H	5.030838	-4.331149	33.271243
H	5.472822	-1.907670	32.877997
H	3.893593	-2.362289	32.246623
H	2.842762	-2.088647	37.138668
H	3.368384	-3.765337	37.027107
H	5.649389	-0.656881	35.095236
H	4.191505	-0.249968	35.994756
H	5.479923	-4.356220	35.725214
H	6.412236	-3.088253	34.935533
H	5.329180	-2.223392	37.010488
C	0.020287	-6.897599	37.759697
C	0.530879	-5.503694	37.363582
C	0.702368	-4.637032	38.620738
C	-0.651022	-4.503765	39.335597
C	-1.164974	-5.895080	39.736851
C	-1.331307	-6.758665	38.476891
C	1.712803	-5.305413	39.565768
C	1.032921	-7.560080	38.706607
C	-0.149651	-6.560549	40.678659
C	1.204990	-6.698974	39.967299
H	-0.101486	-7.513700	36.862089
H	1.486289	-5.589560	36.832028
H	-0.174972	-5.027655	36.672124
H	1.066997	-3.643921	38.336021
H	-0.545895	-3.870195	40.224638
H	-1.377093	-4.009817	38.678565
H	-2.130131	-5.797677	40.245804
H	-1.716926	-7.749096	38.747648
H	-2.069754	-6.304882	37.804763
H	2.690198	-5.388322	39.075196
H	1.858530	-4.686154	40.459140
H	0.689657	-8.565057	38.980811
H	1.997843	-7.682433	38.199937
H	-0.035901	-5.963447	41.591538
H	-0.514635	-7.547194	40.988956
H	1.927707	-7.173107	40.640446
C	3.926575	0.802840	40.405285
C	4.208418	-0.490034	39.624498
C	3.360323	-1.636889	40.195823
C	3.715201	-1.843759	41.676267
C	3.432248	-0.554381	42.462618
C	4.279849	0.589806	41.885248
C	1.871281	-1.277664	40.075399

```
C      2.436062      1.155754     40.283760
C      1.943154     -0.197549     42.336109
C      1.583740      0.013419     40.857407
H      4.532291      1.619035     39.996576
H      3.979693     -0.345647     38.561686
H      5.273865     -0.742489     39.688066
H      3.562444     -2.557488     39.637315
H      3.130261     -2.673778     42.090718
H      4.771879     -2.120314     41.775289
H      3.686477     -0.704004     43.517620
H      4.101954      1.512478     42.450928
H      5.346599      0.356569     41.988318
H      1.598906     -1.147225     39.021058
H      1.254269     -2.097524     40.462644
H      2.225591      2.088906     40.820349
H      2.174727      1.329054     39.232841
H      1.326945     -0.998369     42.762480
H      1.723719      0.710801     42.910316
H      0.521641      0.267199     40.769133
C     -2.618302      5.004731     40.152269
C     -1.751739      6.234254     40.465197
C     -0.492633      5.800497     41.231657
C     -0.905885      5.116534     42.543856
C     -1.770433      3.884047     42.236547
C     -3.026074      4.322638     41.467164
C      0.310015      4.810665     40.373299
C     -1.810034      4.017325     39.296302
C     -0.964807      2.899234     41.375242
C     -0.551637      3.577534     40.060158
H     -3.515416      5.316163     39.606095
H     -1.470154      6.743101     39.535403
H     -2.324465      6.955714     41.060540
H      0.123958      6.678575     41.453203
H     -0.014717      4.819835     43.110112
H     -1.463334      5.818818     43.175444
H     -2.064319      3.398372     43.173456
H     -3.662311      3.453916     41.258624
H     -3.621346      5.010585     42.079829
H      0.629567      5.292574     39.441374
H      1.221979      4.508088     40.902073
H     -2.424951      3.143543     39.048555
H     -1.528524      4.486104     38.345640
H     -0.074842      2.563162     41.920985
H     -1.564283      2.005000     41.165624
H      0.023069      2.874108     39.447660
```

Adamantane$_{13}$

```
         338
E=-478.2573
C     -0.352066     -5.558907     33.738693
C     -1.594474     -6.300902     34.254881
C     -2.804075     -5.967534     33.367930
C     -3.059273     -4.452918     33.403465
```

C	-1.820651	-3.705856	32.885178
C	-0.613469	-4.045315	33.773111
C	-2.510296	-6.399794	31.923065
C	-0.064604	-5.992770	32.292983
C	-1.528994	-4.144140	31.441717
C	-1.269997	-5.657917	31.401156
H	0.508979	-5.797744	34.372466
H	-1.414159	-7.382668	34.254979
H	-1.799111	-6.013494	35.293377
H	-3.688560	-6.498042	33.737435
H	-3.933390	-4.204379	32.789458
H	-3.289940	-4.133655	34.427046
H	-2.004646	-2.626283	32.911924
H	0.273908	-3.502430	33.425540
H	-0.800613	-3.718472	34.803222
H	-2.346327	-7.483333	31.879558
H	-3.374580	-6.184484	31.283152
H	0.832983	-5.485054	31.919408
H	0.142504	-7.069143	32.257110
H	-2.375969	-3.889877	30.793025
H	-0.658240	-3.602525	31.052608
H	-1.062787	-5.968637	30.371221
C	-6.043591	-4.499344	36.046026
C	-5.242491	-5.661543	36.652751
C	-5.387791	-5.649477	38.182357
C	-4.856821	-4.317312	38.733589
C	-5.656762	-3.151377	38.132260
C	-5.511113	-3.170060	36.602756
C	-6.871238	-5.798231	38.553903
C	-7.525394	-4.651591	36.422891
C	-7.139610	-3.306634	38.503465
C	-7.676362	-4.636205	37.951755
H	-5.938485	-4.509636	34.955633
H	-5.598936	-6.616460	36.248013
H	-4.185097	-5.574307	36.374792
H	-4.816319	-6.479216	38.612751
H	-4.936678	-4.304416	39.827350
H	-3.792641	-4.207134	38.492269
H	-5.276159	-2.202697	38.526210
H	-6.061812	-2.330189	36.161946
H	-4.458533	-3.039156	36.323619
H	-7.258464	-6.755375	38.184083
H	-6.986230	-5.810388	39.644527
H	-8.111977	-3.838455	35.978224
H	-7.923208	-5.588644	36.014927
H	-7.259797	-3.275592	39.593148
H	-7.719061	-2.468837	38.096896
H	-8.733495	-4.745399	38.217833
C	-6.254528	2.000264	37.296002
C	-6.233464	0.680840	38.082934
C	-7.191004	0.775516	39.280894
C	-6.747860	1.926530	40.196993
C	-6.770069	3.249381	39.415661
C	-5.813917	3.148252	38.217123

C	-8.615317	1.049052	38.773644
C	-7.681316	2.270144	36.793621
C	-8.195297	3.516492	38.907614
C	-8.642159	2.369540	37.988341
H	-5.571130	1.930731	36.442631
H	-6.526545	-0.151111	37.431249
H	-5.215549	0.467424	38.431148
H	-7.174807	-0.166424	39.840156
H	-7.412349	1.991751	41.067083
H	-5.739181	1.734349	40.582335
H	-6.453284	4.068418	40.070546
H	-5.805333	4.093951	37.661656
H	-4.788565	2.978102	38.567381
H	-8.952766	0.224373	38.134215
H	-9.312187	1.099715	39.619007
H	-7.705721	3.199852	36.212205
H	-8.001258	1.466599	36.119298
H	-8.885281	3.610081	39.754951
H	-8.229053	4.469036	38.364948
H	-9.658648	2.561438	37.627544
C	-5.284812	-1.220028	33.261295
C	-5.616309	0.095502	33.982457
C	-6.407795	1.017768	33.042283
C	-5.564679	1.315761	31.792962
C	-5.232800	0.003422	31.065961
C	-4.444670	-0.916422	32.011310
C	-7.710254	0.319437	32.621895
C	-6.590396	-1.912578	32.840854
C	-6.538174	-0.692651	30.650974
C	-7.383787	-0.995672	31.897313
H	-4.720417	-1.875908	33.933152
H	-6.199142	-0.108332	34.888817
H	-4.692069	0.590510	34.304482
H	-6.643535	1.954811	33.558678
H	-6.111268	1.989990	31.122504
H	-4.639924	1.832486	32.077001
H	-4.631261	0.218233	30.175986
H	-4.183995	-1.849752	31.497590
H	-3.499833	-0.439422	32.298943
H	-8.332008	0.119045	33.502860
H	-8.293846	0.976037	31.965291
H	-6.367834	-2.863983	32.342564
H	-7.191543	-2.152031	33.726317
H	-7.101801	-0.054007	29.960122
H	-6.314591	-1.621683	30.112572
H	-8.314252	-1.491420	31.599456
C	-1.812192	5.187149	33.622529
C	-2.896834	4.726986	32.636433
C	-4.285852	4.926047	33.262497
C	-4.384413	4.101609	34.555183
C	-3.303559	4.559934	35.546445
C	-1.917067	4.362193	34.914398
C	-4.487476	6.413299	33.591464
C	-2.020203	6.673897	33.950034

C	-3.507292	6.048216	35.869310
C	-3.406595	6.877582	34.580023
H	-0.822829	5.044191	33.174385
H	-2.820491	5.294194	31.700923
H	-2.747776	3.671050	32.380048
H	-5.057600	4.597857	32.557527
H	-5.379268	4.219625	35.001491
H	-4.261809	3.034847	34.331930
H	-3.375366	3.970479	36.467008
H	-1.134780	4.667335	35.620005
H	-1.750543	3.299953	34.697847
H	-4.440132	7.012247	32.673937
H	-5.483847	6.571441	34.021662
H	-1.239274	7.020281	34.637903
H	-1.929001	7.276532	33.038293
H	-4.486535	6.200551	36.339091
H	-2.753590	6.383071	36.592339
H	-3.552632	7.938275	34.812084
C	-3.170862	-2.002730	41.421709
C	-2.067433	-0.958009	41.648835
C	-2.291813	-0.249966	42.993931
C	-3.661852	0.445211	42.980898
C	-4.769656	-0.595747	42.757105
C	-4.538725	-1.303195	41.412808
C	-2.258884	-1.287317	44.126896
C	-3.135122	-3.035684	42.558668
C	-4.730218	-1.632538	43.890350
C	-3.362933	-2.332923	43.905770
H	-3.009172	-2.506119	40.462248
H	-1.083026	-1.441266	41.637109
H	-2.070530	-0.225015	40.832953
H	-1.503678	0.494007	43.154041
H	-3.825480	0.971517	43.929099
H	-3.692303	1.202936	42.188572
H	-5.745388	-0.097846	42.747637
H	-5.334585	-2.035771	41.231413
H	-4.585100	-0.576504	40.592596
H	-1.278311	-1.777360	44.161114
H	-2.398811	-0.791261	45.094977
H	-3.905550	-3.799465	42.397191
H	-2.169756	-3.556064	42.564267
H	-4.913006	-1.142958	44.854569
H	-5.529885	-2.370571	43.753217
H	-3.336486	-3.071330	44.714661
C	-0.294586	2.312061	30.580400
C	0.845390	2.077826	29.577318
C	1.431898	0.671742	29.777035
C	1.974201	0.545110	31.208929
C	0.837258	0.775465	32.216502
C	0.251993	2.181127	32.010290
C	0.329540	-0.375609	29.556460
C	-1.392551	1.260584	30.357206
C	-0.263861	-0.271791	31.989509
C	-0.811788	-0.147319	30.559639

H	-0.711121	3.315005	30.436486
H	0.473279	2.188581	28.551648
H	1.627540	2.834424	29.714259
H	2.244070	0.506712	29.060495
H	2.414021	-0.448476	31.357934
H	2.776321	1.274663	31.373880
H	1.226272	0.685253	33.236590
H	-0.548262	2.365989	32.737190
H	1.023509	2.939750	32.189825
H	-0.052841	-0.310309	28.530616
H	0.740568	-1.385193	29.677411
H	-2.222363	1.429592	31.054233
H	-1.804718	1.355675	29.345359
H	0.136374	-1.279777	32.152589
H	-1.072950	-0.130809	32.716463
H	-1.596309	-0.895065	30.399473
C	-0.774300	-0.842712	35.598297
C	-2.284463	-1.048599	35.404384
C	-3.049093	-0.422177	36.580773
C	-2.604100	-1.090436	37.890681
C	-1.094650	-0.884871	38.091212
C	-0.335029	-1.509748	36.910768
C	-2.739312	1.081329	36.646318
C	-0.470644	0.662004	35.665894
C	-0.789658	0.619777	38.152453
C	-1.230088	1.292394	36.843311
H	-0.231094	-1.290177	34.758766
H	-2.608309	-0.594559	34.460084
H	-2.511769	-2.119377	35.335700
H	-4.125552	-0.569650	36.440151
H	-3.157812	-0.665424	38.736626
H	-2.837561	-2.161747	37.865316
H	-0.779556	-1.362549	39.025336
H	0.745782	-1.387249	37.051512
H	-0.527959	-2.588613	36.868243
H	-3.070944	1.575194	35.724931
H	-3.294817	1.544006	37.471047
H	0.607857	0.823074	35.783741
H	-0.762453	1.147470	34.726783
H	-1.311097	1.074920	39.003196
H	0.282998	0.780004	38.315859
H	-1.012362	2.365127	36.889021
C	1.841936	3.520785	35.342602
C	2.152203	4.940056	34.842612
C	3.458076	5.440756	35.478968
C	3.305774	5.457077	37.007688
C	2.998354	4.039155	37.513551
C	1.693874	3.541870	36.871690
C	4.607171	4.496768	35.092628
C	2.995192	2.581882	34.956069
C	4.148322	3.098546	37.121681
C	4.302836	3.076277	35.593324
H	0.910576	3.165848	34.887962
H	2.240355	4.943426	33.749475

H	1.326864	5.616407	35.096221
H	3.677618	6.452540	35.120665
H	4.225344	5.831979	37.473194
H	2.500914	6.142869	37.298797
H	2.889651	4.053293	38.603520
H	1.452225	2.537421	37.240001
H	0.860317	4.193510	37.160701
H	4.740825	4.491184	34.004078
H	5.549262	4.854556	35.525394
H	2.776279	1.560226	35.289675
H	3.099474	2.543105	33.865023
H	5.082702	3.431638	37.589442
H	3.950480	2.086388	37.494990
H	5.123477	2.405598	35.315858
C	4.134614	-1.183574	34.308908
C	2.697037	-1.624942	34.623790
C	2.719800	-2.942611	35.413842
C	3.417529	-4.025836	34.577036
C	4.857074	-3.590825	34.261786
C	4.828874	-2.271291	33.475032
C	3.493351	-2.736448	36.725294
C	4.904140	-0.981597	35.623435
C	5.625148	-3.382521	35.576094
C	4.933032	-2.297771	36.415462
H	4.116117	-0.244238	33.745510
H	2.182559	-0.847905	35.201939
H	2.131482	-1.754329	33.693005
H	1.693880	-3.255101	35.637729
H	3.422435	-4.977333	35.122459
H	2.864090	-4.197268	33.645816
H	5.352361	-4.364292	33.664719
H	5.850568	-1.959567	33.226161
H	4.300764	-2.411702	32.524052
H	2.994253	-1.979627	37.342513
H	3.500193	-3.665696	37.307774
H	5.926936	-0.646208	35.412724
H	4.429265	-0.193601	36.220356
H	5.669285	-4.322732	36.139010
H	6.661131	-3.091128	35.364451
H	5.481699	-2.151020	37.352307
C	-0.024987	-6.734402	38.020080
C	0.555886	-5.370728	37.615650
C	0.689549	-4.474690	38.856699
C	-0.695119	-4.274734	39.491649
C	-1.279532	-5.635556	39.900953
C	-1.407788	-6.528778	38.657309
C	1.622519	-5.149822	39.873829
C	0.910286	-7.403661	39.039052
C	-0.341404	-6.308040	40.915062
C	1.044136	-6.513091	40.283810
H	-0.119658	-7.371503	37.133937
H	1.535134	-5.504454	37.140337
H	-0.093822	-4.890882	36.873639
H	1.104385	-3.503219	38.566109

H	-0.615969	-3.619857	40.367851
H	-1.366237	-3.775315	38.782399
H	-2.266864	-5.490621	40.352858
H	-1.842714	-7.497255	38.932730
H	-2.091869	-6.069494	37.933382
H	2.621580	-5.280168	39.440560
H	1.741436	-4.510507	40.756987
H	0.516917	-8.388019	39.320650
H	1.896367	-7.573447	38.590080
H	-0.256250	-5.689205	41.816510
H	-0.757757	-7.272304	41.230989
H	1.711739	-6.992137	41.008428
C	3.924027	1.019303	40.650897
C	2.445967	1.372086	40.424038
C	1.553538	0.251931	40.980614
C	1.882778	-1.065575	40.262124
C	3.359481	-1.424851	40.488345
C	4.247292	-0.300231	39.933397
C	1.818541	0.090325	42.485422
C	4.182704	0.856217	42.156698
C	3.620119	-1.581750	41.994395
C	3.295236	-0.265651	42.717293
H	4.558460	1.818968	40.253228
H	2.206258	2.323376	40.914262
H	2.252765	1.509721	39.353205
H	0.500217	0.505814	40.818302
H	1.237806	-1.869588	40.636865
H	1.679176	-0.970362	39.188655
H	3.592068	-2.364120	39.974803
H	5.305538	-0.553584	40.070847
H	4.085893	-0.191686	38.853977
H	1.567846	1.017883	43.014218
H	1.172969	-0.694059	42.898715
H	5.239981	0.624177	42.333672
H	3.973585	1.797776	42.678710
H	3.006042	-2.395072	42.399653
H	4.667089	-1.858455	42.168327
H	3.481135	-0.379576	43.790937
C	-2.747588	5.108850	39.844947
C	-1.993462	6.398104	40.205091
C	-0.789034	6.062753	41.098313
C	-1.282689	5.380937	42.383568
C	-2.035166	4.088974	42.029285
C	-3.236174	4.429311	41.133352
C	0.152424	5.109276	40.346429
C	-1.800927	4.158208	39.095907
C	-1.090781	3.140637	41.274615
C	-0.596577	3.816737	39.986633
H	-3.605886	5.350161	39.208391
H	-1.656342	6.904645	39.292677
H	-2.664539	7.093230	40.724135
H	-0.252540	6.983299	41.353373
H	-0.433143	5.154358	43.039152
H	-1.940867	6.058675	42.940639

```
H     -2.386636      3.604903     42.946996
H     -3.794016      3.517098     40.889129
H     -3.929219      5.089905     41.668407
H      0.529148      5.591355     39.436201
H      1.026591      4.877399     40.966831
H     -2.333513      3.241510     38.814814
H     -1.459380      4.624336     38.163797
H     -0.238006      2.874271     41.910729
H     -1.610407      2.205327     41.033474
H      0.076987      3.139373     39.450219
```

Adamantane$_{14}$

```
        364
E=-516.1665
C      3.449840      0.607551     21.739868
C      3.338589      0.492387     20.211815
C      3.123681     -0.976712     19.816194
C      4.312751     -1.815683     20.308827
C      4.426806     -1.706555     21.837221
C      4.637977     -0.235546     22.227715
C      1.831679     -1.495149     20.466383
C      2.156655      0.085357     22.384796
C      3.131366     -2.222661     22.482361
C      1.939315     -1.384831     21.995225
H      3.603227      1.655528     22.019735
H      2.506070      1.106292     19.847448
H      4.249057      0.879880     19.738688
H      3.044080     -1.056382     18.726422
H      4.180721     -2.864012     20.014836
H      5.239929     -1.468615     19.836872
H      5.275453     -2.304982     22.186122
H      4.740909     -0.146074     23.316000
H      5.571157      0.139459     21.790079
H      0.970968     -0.917955     20.107414
H      1.656845     -2.538115     20.175716
H      2.215221      0.181265     23.475837
H      1.302387      0.691129     22.059181
H      2.978640     -3.278254     22.226989
H      3.207853     -2.169017     23.575172
H      1.016588     -1.754570     22.455786
C     -1.753657      4.073682     22.832277
C     -0.810445      2.939682     23.262369
C      0.615972      3.486002     23.429170
C      1.095550      4.073380     22.092967
C      0.157286      5.210357     21.659548
C     -1.268037      4.659728     21.497661
C      0.616708      4.589019     24.498816
C     -1.746931      5.174841     23.903888
C      0.158739      6.308827     22.733836
C     -0.323115      5.727162     24.071644
H     -2.769871      3.682068     22.713719
H     -1.157297      2.498175     24.204440
H     -0.820958      2.138156     22.513772
```

H	1.286597	2.675954	23.736369
H	2.121024	4.449002	22.193799
H	1.118695	3.291370	21.324345
H	0.500777	5.627220	20.706507
H	-1.944768	5.457822	21.168956
H	-1.286887	3.888341	20.718183
H	0.295810	4.178617	25.463922
H	1.633722	4.974114	24.641008
H	-2.432772	5.981715	23.618280
H	-2.109794	4.773833	24.857975
H	1.167611	6.724118	22.845766
H	-0.492194	7.136438	22.426735
H	-0.320901	6.511595	24.836437
C	-2.297974	-2.002059	21.079907
C	-1.912132	-0.967314	22.148089
C	-3.119207	-0.069604	22.461205
C	-3.560368	0.655162	21.180370
C	-3.950454	-0.375263	20.109406
C	-2.741383	-1.272456	19.802587
C	-4.277253	-0.936303	22.979425
C	-3.457882	-2.863920	21.602167
C	-5.106057	-1.241552	20.633677
C	-4.668897	-1.971055	21.913116
H	-1.436070	-2.640802	20.858008
H	-1.573775	-1.474993	23.059411
H	-1.071877	-0.357647	21.794210
H	-2.842016	0.666967	23.223383
H	-4.409631	1.315038	21.395249
H	-2.748933	1.293414	20.810151
H	-4.264082	0.143655	19.197041
H	-3.000819	-2.000550	19.024424
H	-1.915507	-0.668416	19.407563
H	-3.982957	-1.444072	23.905922
H	-5.139236	-0.304209	23.224936
H	-3.729511	-3.620786	20.856265
H	-3.148096	-3.405336	22.504197
H	-5.982700	-0.615202	20.838617
H	-5.407834	-1.968661	19.869996
H	-5.494201	-2.587730	22.285722
C	0.607876	3.566028	16.751824
C	1.132015	2.415550	15.878748
C	0.243931	1.175686	16.065823
C	0.267011	0.751124	17.542191
C	-0.259264	1.897267	18.419856
C	0.629857	3.135415	18.226465
C	-1.197629	1.515809	15.657152
C	-0.834505	3.900055	16.340840
C	-1.699365	2.235962	18.004974
C	-1.727484	2.664203	16.529825
H	1.242468	4.448789	16.617155
H	1.141010	2.716850	14.824296
H	2.168323	2.180190	16.150086
H	0.619115	0.356904	15.442028
H	-0.347880	-0.145713	17.685193

H	1.288172	0.486297	17.842113
H	-0.241392	1.592567	19.471943
H	0.276498	3.955682	18.863071
H	1.657423	2.912980	18.538888
H	-1.232200	1.801051	14.598721
H	-1.838158	0.632484	15.768028
H	-1.213585	4.734427	16.943434
H	-0.861562	4.227355	15.294465
H	-2.349025	1.365434	18.156430
H	-2.094228	3.039839	18.638086
H	-2.755038	2.904755	16.235688
C	-0.923840	-5.885976	22.644582
C	-0.022836	-7.088484	22.324034
C	0.888505	-7.388846	23.524115
C	1.760321	-6.159236	23.821512
C	0.864080	-4.954064	24.145884
C	-0.047486	-4.660169	22.944377
C	0.023211	-7.707757	24.753080
C	-1.784665	-6.209242	23.875609
C	-0.001538	-5.279225	25.372958
C	-0.877745	-6.506594	25.079574
H	-1.572505	-5.673410	21.787751
H	-0.636242	-7.966360	22.087817
H	0.583638	-6.876952	21.434992
H	1.529578	-8.246847	23.293580
H	2.430171	-6.368220	24.664414
H	2.398129	-5.932055	22.958700
H	1.487701	-4.078526	24.357020
H	-0.679240	-3.788800	23.155492
H	0.558277	-4.406024	22.066060
H	-0.589968	-8.596788	24.562108
H	0.662826	-7.942989	25.612377
H	-2.447830	-5.365676	24.102778
H	-2.429408	-7.071799	23.668100
H	0.637200	-5.472376	26.243316
H	-0.631999	-4.418593	25.627507
H	-1.493765	-6.737233	25.955687
C	-4.989681	-5.247905	26.255650
C	-5.425040	-4.140764	25.283392
C	-5.869397	-2.900571	26.074228
C	-7.047274	-3.274147	26.987189
C	-6.616354	-4.379199	27.963956
C	-6.169702	-5.615472	27.168295
C	-4.698998	-2.398898	26.934042
C	-3.821232	-4.740021	27.114561
C	-5.443753	-3.874051	28.818688
C	-4.261867	-3.501928	27.910440
H	-4.673532	-6.131118	25.689881
H	-4.597516	-3.882607	24.611610
H	-6.247015	-4.497981	24.651169
H	-6.178809	-2.112343	25.379069
H	-7.386932	-2.391683	27.542838
H	-7.897520	-3.615680	26.384452
H	-7.457761	-4.644297	28.613422

H	-5.877961	-6.419638	27.854713
H	-7.004529	-5.998773	26.569025
H	-3.857523	-2.108847	26.293241
H	-4.998006	-1.501624	27.489314
H	-3.487278	-5.528919	27.799478
H	-2.964817	-4.491708	26.476085
H	-5.755192	-3.002364	29.406894
H	-5.139578	-4.647116	29.534798
H	-3.427032	-3.141508	28.521503
C	0.469601	-1.933806	26.858192
C	1.335868	-0.950168	27.659829
C	0.750938	0.466336	27.548813
C	0.723871	0.891602	26.072715
C	-0.143904	-0.087263	25.266677
C	0.443072	-1.502422	25.383851
C	-0.681170	0.470699	28.105444
C	-0.961630	-1.923354	27.417134
C	-1.573636	-0.081933	25.829323
C	-1.552429	-0.509570	27.304763
H	0.888425	-2.942890	26.938469
H	1.380281	-1.258034	28.711484
H	2.365497	-0.962469	27.282118
H	1.369786	1.165840	28.121534
H	0.326563	1.909822	25.981985
H	1.742913	0.911857	25.667750
H	-0.161310	0.217542	24.214613
H	-0.156328	-2.208357	24.796193
H	1.457045	-1.524717	24.966310
H	-0.674674	0.188661	29.165278
H	-1.103190	1.481439	28.049432
H	-1.585772	-2.637349	26.866086
H	-0.959009	-2.247883	28.464718
H	-2.011608	0.919024	25.734020
H	-2.208933	-0.762198	25.249148
H	-2.572514	-0.504659	27.704330
C	4.199638	-3.271733	27.835228
C	4.904756	-4.480650	28.469193
C	6.100703	-4.898792	27.599813
C	5.602794	-5.273465	26.195478
C	4.899290	-4.066691	25.555413
C	3.706658	-3.650413	26.430220
C	7.087278	-3.725722	27.493200
C	5.191264	-2.102868	27.728549
C	5.888683	-2.895526	25.454384
C	6.387379	-2.514859	26.856754
H	3.347310	-2.975425	28.456394
H	5.244188	-4.230456	29.481576
H	4.201372	-5.316366	28.568103
H	6.602248	-5.760465	28.053971
H	6.445517	-5.593224	25.570635
H	4.912075	-6.123303	26.254957
H	4.544694	-4.336257	24.554591
H	3.183292	-2.801446	25.973811
H	2.982094	-4.471373	26.493562

H	7.467020	-3.460815	28.487411
H	7.955494	-4.018306	26.890349
H	4.694034	-1.226174	27.295920
H	5.536811	-1.810025	28.727348
H	6.736300	-3.173201	24.816215
H	5.404180	-2.033399	24.980086
H	7.093038	-1.680108	26.782689
C	0.087174	-7.158766	30.548335
C	-0.804149	-6.664279	31.697949
C	-0.844248	-5.128264	31.699990
C	0.580016	-4.582156	31.883531
C	1.475213	-5.071249	30.734480
C	1.509065	-6.607413	30.734646
C	-1.410243	-4.632105	30.360488
C	-0.481739	-6.657573	29.211888
C	0.903156	-4.575699	29.397351
C	-0.520258	-5.121685	29.207512
H	0.114571	-8.253918	30.548816
H	-1.818164	-7.067438	31.588550
H	-0.418643	-7.031627	32.656799
H	-1.480711	-4.777948	32.520039
H	0.562255	-3.485797	31.906744
H	0.989195	-4.912867	32.845850
H	2.490174	-4.681174	30.867687
H	2.161208	-6.969599	29.930596
H	1.935086	-6.974057	31.676484
H	-2.435364	-4.997855	30.225617
H	-1.461972	-3.536594	30.356916
H	0.134613	-7.021314	28.380745
H	-1.490338	-7.059690	29.057651
H	0.891381	-3.479229	29.376557
H	1.544918	-4.901323	28.569658
H	-0.926664	-4.766918	28.253980
C	3.405969	-0.101956	30.596993
C	2.195380	0.783715	30.929627
C	2.033531	0.890615	32.453874
C	1.820220	-0.512315	33.042717
C	3.029760	-1.401634	32.715561
C	3.189317	-1.502351	31.190653
C	3.303975	1.507959	33.058563
C	4.672800	0.518452	31.206237
C	4.297798	-0.778316	33.319124
C	4.517375	0.623914	32.731089
H	3.519418	-0.177027	29.509977
H	2.327579	1.781433	30.493949
H	1.286870	0.360899	30.483770
H	1.170225	1.522941	32.688722
H	1.683259	-0.447354	34.128939
H	0.904894	-0.957259	32.633990
H	2.875258	-2.401407	33.135836
H	4.038226	-2.151218	30.943085
H	2.298385	-1.965217	30.749094
H	3.457429	2.518977	32.662166
H	3.193123	1.607418	34.145141

H	5.548166	-0.094120	30.958270
H	4.849579	1.511824	30.776446
H	4.204697	-0.718330	34.410248
H	5.166244	-1.414685	33.109840
H	5.421522	1.066937	33.162944
C	-2.251049	-0.055633	33.194265
C	-1.913438	0.746728	31.928240
C	-3.066744	0.633994	30.919216
C	-3.277596	-0.842393	30.550070
C	-3.618671	-1.649161	31.812489
C	-2.464141	-1.530267	32.819400
C	-4.352505	1.188462	31.551808
C	-3.538734	0.501144	33.821013
C	-4.902744	-1.088675	32.443160
C	-4.696087	0.387047	32.817035
H	-1.427551	0.025871	33.912122
H	-1.738534	1.798631	32.184447
H	-0.984990	0.370578	31.481644
H	-2.824142	1.207235	30.017647
H	-4.085862	-0.934324	29.814466
H	-2.373056	-1.245833	30.079010
H	-3.768020	-2.701430	31.547022
H	-2.686439	-2.118411	33.718085
H	-1.545213	-1.946265	32.388708
H	-4.223060	2.248471	31.801730
H	-5.179641	1.131217	30.833893
H	-3.779674	-0.050423	34.737920
H	-3.393858	1.548923	34.110720
H	-5.739759	-1.185230	31.741091
H	-5.168619	-1.669406	33.334803
H	-5.612736	0.785072	33.266012
C	0.260492	5.941943	30.841543
C	1.129768	6.257336	29.614567
C	0.609360	5.479570	28.395875
C	0.666127	3.972955	28.691403
C	-0.204258	3.651391	29.916121
C	0.317970	4.434136	31.130836
C	-0.844882	5.887978	28.114495
C	-1.192726	6.349685	30.553706
C	-1.656359	4.064843	29.631172
C	-1.719044	5.571608	29.337959
H	0.633295	6.497190	31.709201
H	1.114204	7.334431	29.408766
H	2.173923	5.987855	29.814748
H	1.230222	5.706373	27.522161
H	0.315647	3.405527	27.820744
H	1.702282	3.663818	28.874905
H	-0.161846	2.576912	30.125269
H	-0.283076	4.198132	32.017409
H	1.347873	4.132896	31.357845
H	-0.897594	6.958356	27.881371
H	-1.221895	5.353349	27.234231
H	-1.820444	6.148422	31.430325
H	-1.250510	7.428294	30.363908

H	-2.047935	3.498820	28.777296
H	-2.292511	3.821693	30.490913
H	-2.754958	5.864432	29.134521
C	-7.143944	2.523507	26.532745
C	-6.649623	2.895154	27.939197
C	-5.122295	3.062580	27.925271
C	-4.469669	1.743616	27.483840
C	-4.958318	1.368690	26.076161
C	-6.486104	1.205990	26.094711
C	-4.741156	4.174494	26.935741
C	-6.757823	3.637226	25.547104
C	-4.578074	2.485055	25.091337
C	-5.230867	3.805878	25.526693
H	-8.232877	2.404410	26.544930
H	-7.127388	3.824123	28.273095
H	-6.935949	2.115961	28.656015
H	-4.772133	3.328075	28.928857
H	-3.377559	1.844243	27.489102
H	-4.717672	0.944694	28.193116
H	-4.492206	0.427844	25.763758
H	-6.846121	0.918628	25.099333
H	-6.769864	0.397245	26.779101
H	-5.183832	5.127322	27.250373
H	-3.653719	4.316938	26.930932
H	-7.123360	3.393397	24.542152
H	-7.236650	4.580019	25.838006
H	-3.487867	2.598564	25.054139
H	-4.903695	2.220060	24.078069
H	-4.958225	4.600704	24.823722
C	3.676909	3.076851	26.427012
C	4.114139	1.978115	27.407927
C	5.645070	1.847837	27.392754
C	6.110968	1.484639	25.974490
C	5.678891	2.581987	24.989582
C	4.147906	2.710677	25.011188
C	6.273987	3.187086	27.807079
C	4.310716	4.412912	26.844356
C	6.307275	3.919506	25.410190
C	5.841979	4.288860	26.826990
H	2.585275	3.167929	26.439204
H	3.766401	2.216830	28.420264
H	3.652677	1.022345	27.131323
H	5.954583	1.064350	28.093209
H	7.201434	1.369049	25.954983
H	5.684657	0.520048	25.673361
H	6.011605	2.320694	23.979099
H	3.824571	3.477839	24.297127
H	3.686757	1.767903	24.692602
H	5.966078	3.448896	28.826630
H	7.367305	3.101336	27.818772
H	3.989634	5.210451	26.163302
H	3.967459	4.695498	27.846892
H	7.401201	3.846990	25.380429
H	6.022742	4.707953	24.702830

```
H      6.291398      5.242407     27.125353
```

Adamantane$_{15}$

```
   390
E=-565.0277
   C        0.9364278436        4.9994906865        3.5257390117
   C        0.6054972257        4.5036646919        2.1096792431
   C        1.7115293444        4.9384219771        1.1357015747
   C        1.8128129091        6.4714503441        1.1320302369
   C        2.1467808290        6.9726580535        2.5455942128
   C        1.0398490306        6.5323596741        3.5160504657
   C        3.0526827532        4.3414373274        1.5892512297
   C        2.2794359728        4.4018166862        3.9730215940
   C        3.4865697346        6.3701905946        2.9958528334
   C        3.3895993908        4.8369675150        3.0041892033
   H        0.1466548806        4.6887149254        4.2184054705
   H        0.5084801069        3.4112786778        2.1061823878
   H       -0.3610093651        4.9088370028        1.7861784432
   H        1.4738115319        4.5833763715        0.1268807230
   H        2.5860736681        6.7955017764        0.4249832912
   H        0.8674896256        6.9106416179        0.7909583112
   H        2.2179527903        8.0657999310        2.5407026408
   H        1.2562499597        6.9010708440        4.5260942027
   H        0.0808375062        6.9729519741        3.2174635118
   H        3.0012935542        3.2459739406        1.5767935908
   H        3.8476506938        4.6290075507        0.8905648914
   H        2.5174579448        4.7320709882        4.9914885055
   H        2.2133173195        3.3075029310        4.0024252967
   H        4.2894243728        6.6926988492        2.3218863275
   H        3.7466499007        6.7365434190        3.9964071628
   H        4.3458418148        4.4092037750        3.3249201071
   C       -0.7354317784       -0.3384388265        4.3256244410
   C       -0.6763745840        1.1966117173        4.2988693549
   C       -0.3842528830        1.7295794240        5.7100971106
   C       -1.4977710507        1.2754640585        6.6662897367
   C       -1.5592287997       -0.2594765549        6.6992611152
   C       -1.8479431639       -0.7873884105        5.2854368346
   C        0.9622278393        1.1715569693        6.1962977196
   C        0.6122633804       -0.8908482195        4.8150203705
   C       -0.2096627227       -0.8125687320        7.1824739290
   C        0.9071131190       -0.3637243791        6.2276984471
   H       -0.9439896960       -0.7162954846        3.3187293765
   H        0.1008133246        1.5315390102        3.6013491642
   H       -1.6272169628        1.6027294075        3.9329191513
   H       -0.3420319389        2.8241294692        5.6885508367
   H       -1.3110354758        1.6667070412        7.6737199671
   H       -2.4626686132        1.6834980763        6.3417345366
   H       -2.3540803961       -0.5809314997        7.3811160838
   H       -1.9141099727       -1.8821087646        5.2986633025
   H       -2.8192684702       -0.4162250286        4.9365188767
   H        1.7700665079        1.5053401312        5.5338616392
   H        1.1914377789        1.5604196670        7.1958385467
   H        0.5900387204       -1.9874275952        4.8191374115
```

H	1.4131566925	-0.5930468759	4.1275299260
H	-0.0003319300	-0.4584319382	8.1991281209
H	-0.2470109444	-1.9077018302	7.2299466387
H	1.8685687987	-0.7585262966	6.5740856947
C	-3.8896655633	-0.8708639203	1.6827670268
C	-4.2071954376	-1.3588231414	0.2609181121
C	-5.5627352082	-0.7939660553	-0.1911901113
C	-6.6600477523	-1.2715480283	0.7722767811
C	-6.3490044653	-0.7835383349	2.1956549691
C	-4.9912710490	-1.3480844355	2.6415178164
C	-5.5052132971	0.7413606591	-0.1752672627
C	-3.8360877448	0.6647018692	1.6934383644
C	-6.2889861767	0.7517252808	2.2055347978
C	-5.1908414085	1.2344599803	1.2456957318
H	-2.9231042288	-1.2748303418	2.0032043014
H	-3.4174777967	-1.0422589897	-0.4310825661
H	-4.2295967251	-2.4551100050	0.2363080675
H	-5.7868176140	-1.1426683689	-1.2053303498
H	-7.6363874916	-0.8910149648	0.4485296321
H	-6.7253770360	-2.3662270449	0.7559955289
H	-7.1322738731	-1.1256221084	2.8808675396
H	-4.7661545551	-1.0230867280	3.6645841359
H	-5.0271168314	-2.4441670181	2.6587486037
H	-4.7392378866	1.0972998525	-0.8748564532
H	-6.4616863471	1.1569300440	-0.5148179222
H	-3.5896776269	1.0254062729	2.6995057577
H	-3.0403390342	1.0182376089	1.0266885468
H	-7.2588624959	1.1679741796	1.9073825292
H	-6.0875196106	1.1139567541	3.2209975658
H	-5.1498924820	2.3292245448	1.2536604825
C	-3.2336609266	5.6824713061	3.1291384353
C	-4.6928995795	6.0458061898	2.8141875265
C	-5.5999915311	5.6018146697	3.9722070274
C	-5.4786554043	4.0817743088	4.1598307428
C	-4.0213952390	3.7130663818	4.4781923746
C	-3.1182779328	4.1622444030	3.3190701876
C	-5.1608341369	6.3100248017	5.2630864252
C	-2.8007553018	6.3909862099	4.4220213013
C	-3.5859903512	4.4263957728	5.7675014685
C	-3.7025813465	5.9472475107	5.5840286212
H	-2.5887715264	5.9996194249	2.3023104832
H	-4.7860968721	7.1270029145	2.6558850930
H	-5.0080395293	5.5606210144	1.8823965002
H	-6.6397691673	5.8623482815	3.7457752368
H	-6.1372440815	3.7493217103	4.9713682586
H	-5.8080385874	3.5626009613	3.2516342344
H	-3.9371681825	2.6289464494	4.6110016927
H	-2.0767450309	3.8869566132	3.5249621173
H	-3.4055700375	3.6441960967	2.3959948673
H	-5.2620202915	7.3962583441	5.1504182056
H	-5.8134393475	6.0158050110	6.0939741511
H	-1.7533365176	6.1558972731	4.6470641753
H	-2.8605189351	7.4784851870	4.2934400744
H	-4.2109693353	4.0998002912	6.6074744778

H	-2.5529663276	4.1552736479	6.0171396281
H	-3.3922714789	6.4540977084	6.5043874461
C	4.2743298269	0.7435413738	3.7567498764
C	4.3945545800	-0.7688798529	3.9990550571
C	5.8581043335	-1.1327931208	4.2932364561
C	6.7325758499	-0.7340068140	3.0946052110
C	6.6184735561	0.7785721556	2.8496717898
C	5.1529221221	1.1376711047	2.5595805163
C	6.3303932345	-0.3727483238	5.5422622877
C	4.7503657763	1.4984746670	5.0075145432
C	7.0883497198	1.5338521166	4.1024697572
C	6.2148900282	1.1408017846	5.3036464129
H	3.2303272444	1.0007913747	3.5470100975
H	3.7559195412	-1.0676964234	4.8390465437
H	4.0396796562	-1.3195563943	3.1194122351
H	5.9413759368	-2.2114164490	4.4662226041
H	7.7777077578	-1.0071087995	3.2842667943
H	6.4186420731	-1.2845673529	2.1995164888
H	7.2422530637	1.0605008977	1.9944151908
H	5.0606227494	2.2127842558	2.3631672333
H	4.8110655547	0.6205722171	1.6547481142
H	5.7274989827	-0.6635242185	6.4110568402
H	7.3687500324	-0.6390130826	5.7741059799
H	4.6501714846	2.5799794541	4.8548524580
H	4.1189286826	1.2401563024	5.8661865808
H	8.1398456554	1.3010204185	4.3097466090
H	7.0309704289	2.6159834565	3.9331640251
H	6.5516927838	1.6798296444	6.1959818732
C	3.8980722623	5.8736523610	-2.3930551143
C	4.0554241064	4.3526189527	-2.2437851349
C	3.6019170190	3.6545602387	-3.5352954122
C	2.1282643272	3.9938229449	-3.8067661717
C	1.9656387185	5.5140111880	-3.9600223952
C	2.4237060944	6.2073523845	-2.6675835099
C	4.4617180618	4.1477298110	-4.7093126043
C	4.7575847671	6.3608653232	-3.5698339966
C	2.8299218906	6.0026352659	-5.1326243246
C	4.3052915449	5.6684299723	-4.8646001474
H	4.2216778951	6.3693170696	-1.4712603319
H	5.1007499884	4.1015756956	-2.0269837588
H	3.4607614712	3.9950803256	-1.3943766116
H	3.7154945757	2.5702990823	-3.4268309715
H	1.7866068679	3.4848506203	-4.7162024086
H	1.5001989799	3.6294762720	-2.9847925923
H	0.9142413132	5.7535840888	-4.1529391164
H	2.2940552947	7.2928261971	-2.7564818335
H	1.8006119330	5.8825319282	-1.8254290197
H	5.5148153084	3.8935733247	-4.5383542934
H	4.1601970948	3.6419699996	-5.6345830398
H	4.6699872427	7.4491764121	-3.6742507599
H	5.8156263122	6.1454361558	-3.3777475271
H	2.5004552461	5.5293558786	-6.0655103557
H	2.7069625892	7.0843735291	-5.2656220350
H	4.9198633872	6.0170432763	-5.7018694699

C	-3.4076659887	0.1839293574	-3.7911535381
C	-4.8989943220	0.4350519411	-4.0621904533
C	-5.3364265336	-0.3390080039	-5.3153615130
C	-4.5090825294	0.1340249294	-6.5204297915
C	-3.0165737254	-0.1176923493	-6.2559250938
C	-2.5851564673	0.6554714394	-5.0001214017
C	-5.0997296093	-1.8409749471	-5.0943178629
C	-3.1763319722	-1.3194399672	-3.5736403183
C	-2.7857290394	-1.6201248987	-6.0320024702
C	-3.6087120492	-2.0976412579	-4.8258006434
H	-3.0978411282	0.7367859587	-2.8975832476
H	-5.4968989541	0.1215157935	-3.1979353473
H	-5.0801198714	1.5078924541	-4.2006069559
H	-6.4000923764	-0.1589632681	-5.5063733434
H	-4.8260159093	-0.3973536735	-7.4259942169
H	-4.6839862033	1.2014340447	-6.7018178543
H	-2.4286655326	0.2207851043	-7.1160678204
H	-1.5159784094	0.5000790892	-4.8112374753
H	-2.7255948294	1.7322096673	-5.1546875783
H	-5.7007842643	-2.1970344548	-4.2488647283
H	-5.4262745595	-2.4066639850	-5.9753386158
H	-2.1177775453	-1.5097986536	-3.3586442520
H	-3.7437130718	-1.6652438145	-2.7011397533
H	-3.0719545385	-2.1824388993	-6.9290509214
H	-1.7200150334	-1.8159248943	-5.8620581566
H	-3.4432888789	-3.1690846156	-4.6680222876
C	-3.8325424616	4.4558505803	-0.9813633683
C	-3.7593604841	5.9903070534	-0.9554502560
C	-2.6909596203	6.4759119585	-1.9472470665
C	-1.3264658364	5.8903518717	-1.5526283753
C	-1.3931360163	4.3555171246	-1.5799091278
C	-2.4651318402	3.8753388594	-0.5893538314
C	-3.0573207972	6.0023876416	-3.3622893404
C	-4.1964172811	3.9878567437	-2.3989400466
C	-1.7632563750	3.8872880189	-2.9957151175
C	-3.1287604679	4.4677865592	-3.3940438405
H	-4.5945627814	4.1117661432	-0.2735211102
H	-4.7354671719	6.4183531510	-1.2137601969
H	-3.5185826413	6.3377931792	0.0565931135
H	-2.6409056362	7.5701660692	-1.9275191327
H	-0.5510996912	6.2460292592	-2.2419453216
H	-1.0432694023	6.2366496253	-0.5512629128
H	-0.4194902642	3.9402563046	-1.2977372993
H	-2.5092350625	2.7794388034	-0.5844945346
H	-2.2019298736	4.1855924728	0.4291972317
H	-4.0206934146	6.4300067717	-3.6654248775
H	-2.3116994529	6.3594048375	-4.0829978752
H	-4.2720365144	2.8939894057	-2.4260175495
H	-5.1800102188	4.3803095275	-2.6840445791
H	-0.9954500928	4.2073435629	-3.7104569990
H	-1.7942769767	2.7915860178	-3.0336611032
H	-3.3907146584	4.1333429138	-4.4038840029
C	3.6469658273	-1.0281327944	-6.3255081485
C	3.7877312401	0.5017251492	-6.3080860432

C	2.4648492022	1.1497955690	-6.7450543348
C	2.1143146119	0.6828682630	-8.1661638328
C	1.9692618005	-0.8466283063	-8.1886756001
C	3.2932755998	-1.4895993459	-7.7476294469
C	1.3469738977	0.7252249581	-5.7802305025
C	2.5262499282	-1.4468116364	-5.3613242164
C	0.8530016221	-1.2662745640	-7.2198647783
C	1.2002495087	-0.8042594135	-5.7964159171
H	4.5911183893	-1.4881354946	-6.0139264006
H	4.0604585150	0.8434417165	-5.3023120256
H	4.5980626506	0.8119952746	-6.9789420737
H	2.5673275257	2.2404820726	-6.7313504912
H	1.1813290209	1.1546490085	-8.4975514167
H	2.8952908694	0.9968628423	-8.8692560269
H	1.7199066885	-1.1772896551	-9.2028420759
H	3.2094354905	-2.5827569076	-7.7783380975
H	4.0951261275	-1.2144250597	-8.4435415760
H	1.5743387943	1.0704221305	-4.7643662447
H	0.4004866005	1.1971691636	-6.0705126450
H	2.4293275171	-2.5392644372	-5.3490711776
H	2.7754242354	-1.1396351495	-4.3384294909
H	-0.1023027231	-0.8291497046	-7.5347194448
H	0.7254718205	-2.3554136547	-7.2417106371
H	0.4030623915	-1.1038418278	-5.1072187170
C	5.9485103174	-0.3359225530	-1.2921680096
C	6.1073830247	1.1921233693	-1.2717014305
C	7.2244487368	1.6109421730	-2.2400549029
C	8.5441424240	0.9520010020	-1.8103423033
C	8.3917801993	-0.5767967968	-1.8320193937
C	7.2714610817	-0.9900030499	-0.8650180106
C	6.8653436852	1.1502596757	-3.6611831962
C	5.5922580666	-0.7912383887	-2.7157966753
C	8.0292391973	-1.0319858602	-3.2540254114
C	6.7083391357	-0.3780636621	-3.6874473080
H	5.1518974039	-0.6323737064	-0.6010570854
H	5.1632769910	1.6727930249	-1.5551661241
H	6.3435076878	1.5316049223	-0.2558521415
H	7.3355852728	2.7007522072	-2.2242621503
H	9.3542940572	1.2593878471	-2.4826700316
H	8.8227905369	1.2878478217	-0.8041528148
H	9.3335894395	-1.0444198648	-1.5248182196
H	7.1655938143	-2.0816430544	-0.8560814561
H	7.5277178547	-0.6889156795	0.1580448955
H	5.9349771193	1.6295178935	-3.9891552803
H	7.6466189949	1.4604672507	-4.3656073655
H	5.4557984093	-1.8792695907	-2.7395470033
H	4.6393043123	-0.3458454542	-3.0260596686
H	8.8304358464	-0.7598918496	-3.9517838039
H	7.9374470379	-2.1244207247	-3.2875711583
H	6.4517605223	-0.7033201573	-4.7016589876
C	-1.1934680575	-6.0685541944	-2.6466914014
C	-1.0978002303	-4.5352575509	-2.6258868603
C	0.0358006422	-4.0731827608	-3.5545360907
C	1.3647106163	-4.6768150475	-3.0748756315

C	1.2756182424	-6.2105996606	-3.0966509144
C	0.1385895061	-6.6672021877	-2.1694908457
C	-0.2522663754	-4.5510677414	-4.9861204459
C	-1.4788140281	-6.5409656921	-4.0806899398
C	0.9839286675	-6.6831378698	-4.5292565854
C	-0.3458464517	-6.0845772508	-5.0126498750
H	-2.0019831234	-6.3958532080	-1.9839029934
H	-2.0497488703	-4.0941141483	-2.9451329068
H	-0.9128385922	-4.1843275733	-1.6033460531
H	0.1018188415	-2.9797140992	-3.5385819409
H	2.1856149945	-4.3379981005	-3.7184406973
H	1.5926319331	-4.3278007655	-2.0604319178
H	2.2238780206	-6.6387522339	-2.7538137708
H	0.0770068404	-7.7622332362	-2.1605344034
H	0.3449539742	-6.3537976063	-1.1388744658
H	-1.1888180893	-4.1108654212	-5.3493405980
H	0.5408585505	-4.2106773475	-5.6628186733
H	-1.5699002431	-7.6337071500	-4.1055217856
H	-2.4371849840	-6.1354859271	-4.4269125851
H	1.7982544876	-6.3800022213	-5.1984353604
H	0.9379792272	-7.7784760643	-4.5622405094
H	-0.5518703754	-6.4221256607	-6.0343469521
C	-0.5624273071	0.2206496693	-0.7788780567
C	-0.3759745672	-1.2867404404	-0.5473738628
C	1.1039059188	-1.5896216842	-0.2660019694
C	1.9516858533	-1.1463408163	-1.4681746142
C	1.7713236881	0.3614849377	-1.7023494174
C	0.2896790804	0.6594452257	-1.9796487705
C	1.5525343805	-0.8179241102	0.9846001995
C	-0.1098243102	0.9873897262	0.4734026493
C	2.2177728974	1.1282954194	-0.4479894353
C	1.3707778149	0.6907724759	0.7567589860
H	-1.6179783963	0.4343648306	-0.9794957475
H	-0.9950542390	-1.6178232950	0.2951558141
H	-0.7135079227	-1.8465603166	-1.4280697805
H	1.2344134135	-2.6647635946	-0.1007012805
H	3.0088935377	-1.3757192469	-1.2877463369
H	1.6549362224	-1.7041988032	-2.3646060208
H	2.3761698496	0.6751565253	-2.5601488088
H	0.1501291301	1.7308411354	-2.1683343710
H	-0.0364672568	0.1338855910	-2.8854050761
H	0.9690314382	-1.1396269589	1.8557303091
H	2.6029929503	-1.0410252045	1.2073016905
H	-0.2572115294	2.0645601290	0.3285643121
H	-0.7233033684	0.6969579233	1.3348268548
H	3.2797499227	0.9393113712	-0.2497274303
H	2.1130108633	2.2080253477	-0.6097709207
H	1.6908468590	1.2381029109	1.6501989142
C	4.7278204476	-3.5710321897	0.9871820197
C	3.5951875833	-4.0451934455	1.9107066036
C	3.5101299789	-5.5791177034	1.8825105436
C	3.2308767358	-6.0470927903	0.4460340358
C	4.3624704110	-5.5780990331	-0.4815690385
C	4.4456238728	-4.0442025983	-0.4470257757

C	4.8449593784	-6.1717564888	2.3597947061
C	6.0594595060	-4.1686675025	1.4673030516
C	5.6952476496	-6.1702896597	0.0019071964
C	5.9807365197	-5.7027317586	1.4372230045
H	4.7865894862	-2.4773235831	1.0085692587
H	3.7735785446	-3.6965688164	2.9350938771
H	2.6412667055	-3.6097374216	1.5892017524
H	2.7023578283	-5.9149726379	2.5420499791
H	3.1479626831	-7.1402936900	0.4161347315
H	2.2703739350	-5.6468837778	0.0994474524
H	4.1610126809	-5.9123642489	-1.5051559124
H	5.2373688735	-3.6949216611	-1.1209013729
H	3.5067545284	-3.6084463004	-0.8098200027
H	5.0472033814	-5.8623221973	3.3923564993
H	4.7903813938	-7.2671318032	2.3625193080
H	6.8799713164	-3.8209567843	0.8278854742
H	6.2822632075	-3.8232075716	2.4840502157
H	5.6559426670	-7.2656558456	-0.0361195384
H	6.5090340117	-5.8592219729	-0.6643913116
H	6.9310179322	-6.1263391450	1.7804018538
C	-5.8602204387	-5.0129731338	-0.6987781408
C	-5.0560019939	-4.5389498792	-1.9190725340
C	-5.4878613914	-5.3289593558	-3.1641973995
C	-5.2323170476	-6.8259040623	-2.9311706902
C	-6.0368668135	-7.3060961856	-1.7133767617
C	-5.6039015784	-6.5107600560	-0.4720357312
C	-6.9864736036	-5.1022940325	-3.4163376308
C	-7.3578199612	-4.7865797757	-0.9574061980
C	-7.5340586637	-7.0740148400	-1.9688938623
C	-7.7954586185	-5.5777820502	-2.1995521741
H	-5.5506754778	-4.4487256862	0.1877411568
H	-5.2138124256	-3.4657308300	-2.0804060185
H	-3.9830924440	-4.6780854895	-1.7389094447
H	-4.9136287025	-4.9889751171	-4.0330783971
H	-5.5181921908	-7.3991983842	-3.8213762499
H	-4.1624292414	-7.0047076470	-2.7692308737
H	-5.8527296648	-8.3733648041	-1.5488175128
H	-6.1571192745	-6.8577913029	0.4091482076
H	-4.5406404451	-6.6842740159	-0.2662506103
H	-7.1806185802	-4.0394791505	-3.6049333065
H	-7.3030368107	-5.6461646524	-4.3145724241
H	-7.9421192174	-5.1022667610	-0.0845182790
H	-7.5574672624	-3.7179844384	-1.1023331074
H	-7.8606318512	-7.6519245043	-2.8419278642
H	-8.1215242950	-7.4316288653	-1.1145160819
H	-8.8632663267	-5.4143200071	-2.3820804249
C	-0.6832753139	-4.9540662215	1.4631644936
C	-0.5340028884	-6.4601542643	1.7277402090
C	-1.4217464622	-6.8694924727	2.9131966701
C	-0.9957497643	-6.0841989668	4.1631121617
C	-1.1460370299	-4.5769790770	3.9050253646
C	-0.2593148362	-4.1735648155	2.7167801489
C	-2.8880872283	-6.5477238062	2.5859351382
C	-2.1515652731	-4.6376176191	1.1392329700

```
C      -2.6129433130      -4.2609926262       3.5747638089
C      -3.0430843045      -5.0416712520       2.3234583445
H      -0.0494734727      -4.6643208215       0.6179115378
H      -0.8141602753      -7.0293106378       0.8331427496
H       0.5141888378      -6.7017895069       1.9418870600
H      -1.3141773252      -7.9437089857       3.0996756647
H      -1.6094421415      -6.3823709900       5.0217925600
H       0.0441696939      -6.3198460295       4.4198087260
H      -0.8412781596      -4.0191274240       4.7971980521
H      -0.3421967327      -3.0952291168       2.5343291911
H       0.7937543431      -4.3749890381       2.9479779739
H      -3.2121580360      -7.1179110062       1.7069011555
H      -3.5346361261      -6.8533028344       3.4174573806
H      -2.2678946994      -3.5676441421       0.9279951043
H      -2.4615897372      -5.1744207815       0.2345307839
H      -3.2551460087      -4.5266975008       4.4231709121
H      -2.7376591140      -3.1841195338       3.4081644007
H      -4.0892915345      -4.8155546791       2.0898530562
```

Adamantane$_{16}$

```
        416
E=-605.0058
C     -1.726931     -1.316421      2.789395
C     -1.256878     -0.818169      4.164670
C     -1.984519      0.487601      4.520275
C     -1.672735      1.550292      3.455414
C     -2.143712      1.058312      2.078082
C     -1.415973     -0.249130      1.728889
C     -3.499301      0.232731      4.555857
C     -3.242361     -1.566974      2.829891
C     -3.657989      0.801502      2.119888
C     -3.974843     -0.262982      3.181361
H     -1.206865     -2.247175      2.537725
H     -1.454383     -1.579612      4.928822
H     -0.172571     -0.653494      4.153994
H     -1.648282      0.840698      5.501373
H     -2.170780      2.493993      3.708861
H     -0.595774      1.756462      3.432946
H     -1.919938      1.817235      1.320449
H     -1.728750     -0.600978      0.738264
H     -0.334434     -0.074787      1.675430
H     -3.738782     -0.509646      5.326805
H     -4.029183      1.153671      4.827752
H     -3.587431     -1.942976      1.859042
H     -3.476222     -2.341279      3.570512
H     -4.191096      1.732045      2.349460
H     -4.010608      0.468904      1.136017
H     -5.054901     -0.444361      3.210103
C      1.439712     -9.892738     -0.583232
C      0.463530     -8.809044     -0.100261
C     -0.981399     -9.248607     -0.382959
C     -1.161310     -9.465086     -1.893328
C     -0.189281    -10.550448     -2.381238
```

C	1.253606	-10.107750	-2.093070
C	-1.267026	-10.564650	0.356798
C	1.147873	-11.206896	0.157554
C	-0.476514	-11.863096	-1.636128
C	-0.294958	-11.652728	-0.125159
H	2.469137	-9.577319	-0.380980
H	0.599835	-8.630678	0.973210
H	0.674197	-7.860257	-0.608567
H	-1.675655	-8.474770	-0.037312
H	-2.195100	-9.760077	-2.110391
H	-0.979464	-8.527540	-2.432583
H	-0.318924	-10.702705	-3.458298
H	1.959167	-10.865578	-2.454796
H	1.478417	-9.181758	-2.636187
H	-1.162359	-10.419413	1.438827
H	-2.302432	-10.879154	0.178318
H	1.852075	-11.984027	-0.163638
H	1.295555	-11.071982	1.235862
H	-1.498109	-12.200575	-1.848891
H	0.197948	-12.652198	-1.990225
H	-0.500673	-12.589454	0.404441
C	2.810499	4.522954	2.461926
C	3.127665	5.561113	1.374629
C	2.638586	5.050195	0.010548
C	3.346110	3.727344	-0.321002
C	3.029723	2.684224	0.761914
C	3.517433	3.201355	2.124140
C	1.121447	4.814391	0.069496
C	1.292784	4.289051	2.514609
C	1.511581	2.454707	0.819371
C	0.799394	3.774269	1.153720
H	3.160151	4.889013	3.433447
H	2.645426	6.516560	1.613998
H	4.207370	5.750825	1.340001
H	2.865231	5.792155	-0.763025
H	3.020046	3.362227	-1.302444
H	4.429474	3.885447	-0.385667
H	3.535581	1.742178	0.523783
H	3.315281	2.456791	2.903708
H	4.603940	3.349783	2.102959
H	0.601618	5.755707	0.285222
H	0.756777	4.467507	-0.904938
H	1.051066	3.564388	3.301650
H	0.776965	5.221565	2.773660
H	1.153182	2.066838	-0.141840
H	1.273941	1.696434	1.575357
H	-0.282859	3.608806	1.193724
C	-5.657678	-7.119343	-0.789352
C	-7.171435	-7.378776	-0.746688
C	-7.475548	-8.516516	0.240235
C	-6.748922	-9.791848	-0.213625
C	-5.233936	-9.538856	-0.255097
C	-4.935578	-8.398234	-1.240445
C	-6.981902	-8.121181	1.640526

C	-5.169092	-6.727198	0.613664
C	-4.745958	-9.140452	1.146290
C	-5.467509	-7.863567	1.603647
H	-5.443043	-6.308136	-1.493614
H	-7.701477	-6.467129	-0.445547
H	-7.535809	-7.641515	-1.747264
H	-8.555359	-8.699383	0.269464
H	-6.974333	-10.617373	0.472346
H	-7.106466	-10.096732	-1.204616
H	-4.718023	-10.449183	-0.579423
H	-3.854605	-8.220619	-1.294447
H	-5.260638	-8.678561	-2.249768
H	-7.507809	-7.222943	1.986105
H	-7.210722	-8.917929	2.358626
H	-4.092403	-6.519143	0.592409
H	-5.663453	-5.804270	0.940113
H	-4.935736	-9.954594	1.856201
H	-3.661526	-8.976631	1.134729
H	-5.118736	-7.581365	2.603132
C	-6.141869	-6.244062	-7.324771
C	-6.445980	-7.549531	-6.573872
C	-6.207431	-7.350533	-5.069064
C	-4.742119	-6.952847	-4.834213
C	-4.433301	-5.645808	-5.580662
C	-4.676771	-5.849033	-7.084115
C	-7.126594	-6.233429	-4.551369
C	-7.061678	-5.130097	-6.801338
C	-5.357238	-4.533084	-5.062088
C	-6.824061	-4.924615	-5.297420
H	-6.312329	-6.388152	-8.397287
H	-7.483986	-7.854417	-6.753652
H	-5.808137	-8.357669	-6.952054
H	-6.425395	-8.282323	-4.535636
H	-4.553960	-6.827250	-3.761050
H	-4.074844	-7.750910	-5.181622
H	-3.388042	-5.364520	-5.412260
H	-4.440747	-4.928336	-7.631450
H	-4.008044	-6.627297	-7.471526
H	-8.177394	-6.513279	-4.693984
H	-6.979420	-6.094819	-3.473426
H	-6.868542	-4.196829	-7.344208
H	-8.110987	-5.391355	-6.984237
H	-5.179724	-4.364564	-3.993020
H	-5.132827	-3.588918	-5.573122
H	-7.481287	-4.130385	-4.926604
C	-0.512669	-5.417686	-2.297991
C	-2.032791	-5.637730	-2.260686
C	-2.737003	-4.330625	-1.865062
C	-2.248196	-3.889043	-0.477070
C	-0.728513	-3.664498	-0.509481
C	-0.029450	-4.973188	-0.908963
C	-2.400403	-3.239719	-2.893368
C	-0.182081	-4.324219	-3.325604
C	-0.396858	-2.575647	-1.541538

C	-0.881004	-3.013810	-2.932172
H	-0.012585	-6.350695	-2.579986
H	-2.388668	-5.975414	-3.241529
H	-2.280117	-6.430003	-1.543689
H	-3.820674	-4.489680	-1.839649
H	-2.758870	-2.966834	-0.174414
H	-2.499857	-4.650686	0.270875
H	-0.382190	-3.350297	0.481169
H	1.058168	-4.831787	-0.916615
H	-0.241465	-5.753998	-0.168446
H	-2.762448	-3.532814	-3.886270
H	-2.913149	-2.306014	-2.632372
H	0.902893	-4.171856	-3.376733
H	-0.505225	-4.637447	-4.325717
H	-0.874601	-1.630179	-1.257511
H	0.684178	-2.391294	-1.559930
H	-0.644184	-2.236074	-3.666538
C	-1.306914	-2.242832	-9.450412
C	-2.268606	-1.144210	-8.972120
C	-2.070072	-0.899090	-7.468307
C	-2.352383	-2.198743	-6.699120
C	-1.391025	-3.300369	-7.171472
C	-1.591001	-3.539076	-8.676123
C	-0.620025	-0.460904	-7.211111
C	0.141116	-1.800690	-9.188370
C	0.057336	-2.855989	-6.915519
C	0.345763	-1.558017	-7.685145
H	-1.450229	-2.416163	-10.522572
H	-2.090386	-0.217915	-9.531536
H	-3.305777	-1.440221	-9.171135
H	-2.756299	-0.115090	-7.129600
H	-2.233401	-2.031460	-5.621754
H	-3.391147	-2.512612	-6.858218
H	-1.594466	-4.226078	-6.622179
H	-0.923921	-4.337859	-9.022412
H	-2.616202	-3.877090	-8.870254
H	-0.410625	0.477803	-7.738194
H	-0.470697	-0.264068	-6.142565
H	0.838973	-2.568269	-9.544480
H	0.362962	-0.885720	-9.750841
H	0.218940	-2.700601	-5.841947
H	0.753599	-3.642935	-7.230001
H	1.378607	-1.242447	-7.501034
C	-8.573477	-0.018337	-6.769104
C	-8.091051	0.403975	-5.372858
C	-6.571304	0.629445	-5.394886
C	-5.869401	-0.673269	-5.808065
C	-6.346036	-1.098667	-7.205478
C	-7.866414	-1.319328	-7.178934
C	-6.235038	1.732280	-6.410499
C	-8.232295	1.086581	-7.780884
C	-6.010826	0.008688	-8.216485
C	-6.712978	1.313114	-7.809422
H	-9.657013	-0.178766	-6.751149

H	-8.603465	1.321330	-5.058814
H	-8.345872	-0.369060	-4.637715
H	-6.229640	0.930994	-4.398583
H	-4.781949	-0.530772	-5.808166
H	-6.085185	-1.464717	-5.080121
H	-5.844770	-2.027843	-7.497730
H	-8.217071	-1.642912	-8.166506
H	-8.117600	-2.122527	-6.475545
H	-6.713341	2.674375	-6.116357
H	-5.153879	1.916145	-6.421606
H	-8.590157	0.806058	-8.779001
H	-8.746383	2.016725	-7.510136
H	-4.925852	0.163013	-8.259322
H	-6.328005	-0.291641	-9.222549
H	-6.472405	2.101557	-8.531039
C	-2.601558	1.838291	-4.409956
C	-3.549087	2.945706	-3.923741
C	-3.313455	3.211206	-2.428769
C	-3.579550	1.923021	-1.634904
C	-2.632215	0.812685	-2.115131
C	-2.869295	0.553591	-3.610995
C	-1.856690	3.649504	-2.213086
C	-1.146682	2.280617	-4.189435
C	-1.177152	1.257164	-1.900737
C	-0.904959	2.543647	-2.695166
H	-2.771318	1.650435	-5.475789
H	-3.382692	3.863726	-4.500182
H	-4.591396	2.649405	-4.093299
H	-3.989640	4.001444	-2.084409
H	-3.434000	2.104960	-0.563190
H	-4.622514	1.609421	-1.764274
H	-2.824069	-0.104830	-1.548204
H	-2.212467	-0.251489	-3.962262
H	-3.899604	0.215329	-3.775378
H	-1.658311	4.580310	-2.758138
H	-1.680959	3.860799	-1.151339
H	-0.459262	1.506529	-4.551704
H	-0.936770	3.187168	-4.769833
H	-0.989109	1.427055	-0.833687
H	-0.490350	0.464292	-2.221085
H	0.132702	2.859309	-2.540673
C	-9.825452	3.925916	-1.576466
C	-9.085152	2.632364	-1.949644
C	-7.575960	2.905958	-2.041503
C	-7.316662	3.973669	-3.115378
C	-8.052434	5.270862	-2.745464
C	-9.560366	4.991146	-2.651404
C	-7.067628	3.416105	-0.684249
C	-9.311407	4.432950	-0.220139
C	-7.543317	5.774831	-1.386218
C	-7.803090	4.711728	-0.307990
H	-10.901110	3.728737	-1.511731
H	-9.282350	1.856032	-1.200542
H	-9.456980	2.249950	-2.908005

H	-7.050110	1.982258	-2.307108
H	-6.240166	4.163525	-3.204635
H	-7.657221	3.614214	-4.093983
H	-7.866605	6.030406	-3.512672
H	-10.099890	5.914300	-2.407139
H	-9.940812	4.650075	-3.621925
H	-7.227753	2.654773	0.088776
H	-5.986930	3.596940	-0.731400
H	-9.847076	5.345762	0.067663
H	-9.511705	3.688976	0.560336
H	-6.470889	5.996769	-1.445034
H	-8.046620	6.712218	-1.119841
H	-7.438989	5.072552	0.660248
C	4.036806	0.460090	-2.576401
C	4.392549	1.525083	-3.625124
C	3.944484	1.053065	-5.017026
C	4.656382	-0.264153	-5.361389
C	4.301534	-1.333993	-4.317151
C	4.748315	-0.855709	-2.926980
C	2.425237	0.823606	-5.012023
C	2.517188	0.232700	-2.577625
C	2.781359	-1.557124	-4.313503
C	2.064697	-0.243243	-3.966576
H	4.357256	0.798436	-1.584966
H	3.907139	2.476317	-3.375597
H	5.473619	1.710078	-3.620782
H	4.198589	1.814110	-5.763033
H	4.359825	-0.601416	-6.361971
H	5.741918	-0.109978	-5.387787
H	4.810569	-2.271985	-4.564343
H	4.518491	-1.619642	-2.174283
H	5.835582	-0.712375	-2.910091
H	1.902940	1.761534	-4.787676
H	2.089951	0.504583	-6.006328
H	2.247668	-0.511335	-1.818172
H	1.997496	1.160660	-2.310098
H	2.451747	-1.917500	-5.295492
H	2.516747	-2.333952	-3.585846
H	0.981016	-0.404127	-3.964979
C	3.718511	-5.214188	-2.495498
C	3.417429	-6.535272	-1.771157
C	4.393598	-7.622348	-2.246880
C	4.235726	-7.820417	-3.762233
C	4.538793	-6.502655	-4.492000
C	3.562523	-5.418330	-4.010250
C	5.833518	-7.183561	-1.938840
C	5.160115	-4.781639	-2.186260
C	5.978153	-6.066370	-4.177944
C	6.140499	-5.863926	-2.663710
H	3.021408	-4.440694	-2.155109
H	3.505200	-6.398639	-0.686556
H	2.384091	-6.845092	-1.969089
H	4.177479	-8.563162	-1.728761
H	4.913743	-8.608752	-4.111056

H	3.216814	-8.153137	-3.994548
H	4.425093	-6.646076	-5.572070
H	3.755402	-4.476651	-4.538458
H	2.531646	-5.708288	-4.247307
H	5.966376	-7.058274	-0.857411
H	6.539386	-7.960494	-2.256579
H	5.381095	-3.828248	-2.681446
H	5.279999	-4.614111	-1.109078
H	6.686942	-6.823597	-4.534376
H	6.214058	-5.136526	-4.709645
H	7.167407	-5.553436	-2.441662
C	-8.069211	-0.913294	-0.194358
C	-8.325797	-1.110439	-1.696317
C	-7.392573	-2.201560	-2.243583
C	-5.931830	-1.779311	-2.023614
C	-5.668811	-1.583713	-0.522458
C	-6.606716	-0.494460	0.020505
C	-7.658487	-3.519114	-1.499181
C	-8.333896	-2.234241	0.544551
C	-5.939536	-2.902637	0.217731
C	-7.400206	-3.327791	0.003416
H	-8.735300	-0.134940	0.193713
H	-9.372557	-1.390092	-1.866079
H	-8.158253	-0.168575	-2.232764
H	-7.577626	-2.340832	-3.314398
H	-5.255062	-2.541515	-2.428443
H	-5.722668	-0.849452	-2.566263
H	-4.627033	-1.282200	-0.368068
H	-6.415336	-0.330864	1.088014
H	-6.409261	0.458323	-0.485652
H	-8.693012	-3.843521	-1.664569
H	-7.011268	-4.311421	-1.894389
H	-8.173947	-2.101320	1.621466
H	-9.380532	-2.535005	0.414730
H	-5.262700	-3.684590	-0.147317
H	-5.735701	-2.781899	1.288637
H	-7.591211	-4.268539	0.531393
C	2.537384	-1.172265	2.581483
C	2.304851	-1.370460	1.075750
C	3.269298	-2.438776	0.537702
C	4.717240	-1.984691	0.777945
C	4.956249	-1.787895	2.282955
C	3.987221	-0.721539	2.816634
C	3.023255	-3.764228	1.274880
C	2.292717	-2.501065	3.313176
C	4.705483	-3.114812	3.015855
C	3.257603	-3.571818	2.781257
H	1.849052	-0.410180	2.962974
H	1.266908	-1.672897	0.891525
H	2.458253	-0.423441	0.544172
H	3.101366	-2.578845	-0.535828
H	5.416104	-2.730339	0.379882
H	4.912597	-1.048725	0.240676
H	5.988923	-1.463670	2.451805

H	4.160977	-0.557000	3.887009
H	4.169886	0.236972	2.315766
H	1.998487	-4.111146	1.095063
H	3.693127	-4.540662	0.885967
H	2.435616	-2.367924	4.392458
H	1.254850	-2.824727	3.168830
H	5.404338	-3.880326	2.657514
H	4.892604	-2.992853	4.089669
H	3.080833	-4.518220	3.304062
C	0.188543	-5.744753	4.349831
C	-0.804246	-4.713636	4.908177
C	-2.242954	-5.206612	4.688976
C	-2.492229	-5.396833	3.184940
C	-1.503773	-6.429680	2.621835
C	-0.066945	-5.933779	2.846677
C	-2.436278	-6.549497	5.410141
C	-0.011002	-7.086037	5.072386
C	-1.698557	-7.769359	3.348508
C	-1.447363	-7.585187	4.852911
H	1.213488	-5.391301	4.506898
H	-0.619653	-4.553608	5.977311
H	-0.658835	-3.745855	4.412992
H	-2.949035	-4.470219	5.088301
H	-3.523125	-5.729733	3.013805
H	-2.376729	-4.440795	2.660053
H	-1.682962	-6.563272	1.549411
H	0.648934	-6.653353	2.431110
H	0.091692	-4.987405	2.315460
H	-2.281467	-6.424069	6.488629
H	-3.465992	-6.902811	5.277135
H	0.706408	-7.825848	4.696888
H	0.186954	-6.969045	6.144713
H	-2.715343	-8.144288	3.180011
H	-1.012049	-8.521637	2.941385
H	-1.587214	-8.541187	5.369396
C	-1.472675	-8.425097	-6.958987
C	-1.110910	-7.289008	-7.928013
C	0.411305	-7.079909	-7.937703
C	0.884220	-6.716755	-6.521751
C	0.527603	-7.851258	-5.548800
C	-0.994867	-8.058666	-5.545513
C	1.100489	-8.377711	-8.386620
C	-0.778828	-9.719411	-7.410953
C	1.216049	-9.147255	-6.003959
C	0.744079	-9.516550	-7.418566
H	-2.558213	-8.572358	-6.953419
H	-1.464690	-7.528509	-8.938068
H	-1.615718	-6.363154	-7.626491
H	0.666990	-6.269888	-8.629640
H	1.967466	-6.545201	-6.519555
H	0.414512	-5.780681	-6.196181
H	0.865185	-7.589960	-4.539935
H	-1.264941	-8.853330	-4.839443
H	-1.497912	-7.146471	-5.202257

H	0.787532	-8.638090	-9.405000
H	2.187537	-8.235285	-8.416190
H	-1.045703	-10.543740	-6.738454
H	-1.125694	-10.002650	-8.412062
H	2.305157	-9.018703	-5.992332
H	0.985689	-9.961051	-5.305650
H	1.236343	-10.440445	-7.741587

Adamantane$_{17}$

442
E=-656.2810

C	-1.725697	-1.323545	2.793800
C	-1.248042	-0.850618	4.175382
C	-1.967696	0.452706	4.555379
C	-1.654425	1.531380	3.507157
C	-2.132980	1.064763	2.123626
C	-1.413212	-0.240339	1.750011
C	-3.483617	0.204914	4.592434
C	-3.242226	-1.567099	2.835841
C	-3.648383	0.814926	2.166869
C	-3.966746	-0.265461	3.211692
H	-1.211317	-2.252592	2.524738
H	-1.446609	-1.623664	4.927514
H	-0.162956	-0.691253	4.163355
H	-1.626048	0.787732	5.540935
H	-2.146712	2.473232	3.778128
H	-0.576517	1.732467	3.484057
H	-1.908133	1.835049	1.377870
H	-1.731430	-0.574076	0.754862
H	-0.331002	-0.070582	1.695379
H	-3.724038	-0.548971	5.351836
H	-4.007789	1.123862	4.881592
H	-3.592787	-1.925153	1.860186
H	-3.477302	-2.352430	3.564369
H	-4.175887	1.744196	2.413882
H	-4.006319	0.500524	1.178943
H	-5.047602	-0.441847	3.241490
C	1.435685	-9.904140	-0.589055
C	0.462068	-8.819757	-0.102467
C	-0.983973	-9.255749	-0.385008
C	-1.165637	-9.468026	-1.895764
C	-0.196181	-10.554057	-2.387290
C	1.247830	-10.114940	-2.099270
C	-1.271549	-10.573105	0.351650
C	1.141902	-11.219602	0.148643
C	-0.485349	-11.868029	-1.645270
C	-0.302054	-11.661867	-0.133931
H	2.465907	-9.591267	-0.386909
H	0.599666	-8.644393	0.971333
H	0.674161	-7.870096	-0.608544
H	-1.676397	-8.481427	-0.036781
H	-2.200197	-9.760425	-2.112664
H	-0.982413	-8.529471	-2.432792

H	-0.327068	-10.703316	-3.464620
H	1.951577	-10.873234	-2.463545
H	1.473992	-9.188013	-2.640228
H	-1.165646	-10.430830	1.433952
H	-2.307730	-10.885111	0.173284
H	1.844287	-11.997299	-0.175148
H	1.290796	-11.087725	1.227159
H	-1.507795	-12.202951	-1.857989
H	0.187242	-12.657555	-2.001968
H	-0.509153	-12.599531	0.393465
C	2.797871	4.516554	2.455462
C	3.163947	5.556621	1.385494
C	2.703814	5.064729	0.004453
C	3.396167	3.731569	-0.317694
C	3.030903	2.686559	0.747878
C	3.489831	3.184701	2.127075
C	1.181593	4.856387	0.019541
C	1.275283	4.310169	2.464288
C	1.507835	2.484532	0.761496
C	0.810683	3.814470	1.086287
H	3.126946	4.869037	3.439116
H	2.692651	6.518986	1.618868
H	4.247461	5.726731	1.382247
H	2.965316	5.808027	-0.756737
H	3.090961	3.379667	-1.310604
H	4.483601	3.870228	-0.351111
H	3.526012	1.737150	0.516499
H	3.252594	2.438306	2.894916
H	4.579022	3.313313	2.137136
H	0.673129	5.805487	0.228132
H	0.837938	4.523373	-0.967290
H	0.998655	3.584342	3.238658
H	0.769462	5.250079	2.716228
H	1.169336	2.110330	-0.212263
H	1.235537	1.725255	1.504676
H	-0.275088	3.668611	1.095026
C	-5.656209	-7.114750	-0.778036
C	-7.170592	-7.369893	-0.731900
C	-7.475683	-8.506630	0.255877
C	-6.753700	-9.784077	-0.199444
C	-5.238102	-9.535378	-0.244391
C	-4.938755	-8.395743	-1.230581
C	-6.977743	-8.112498	1.654987
C	-5.163331	-6.723793	0.623809
C	-4.745819	-9.138160	1.155827
C	-5.462714	-7.859175	1.614634
H	-5.440878	-6.304253	-1.482902
H	-7.697368	-6.456708	-0.429690
H	-7.537978	-7.631740	-1.731608
H	-8.555938	-8.686437	0.287583
H	-6.979888	-10.608865	0.487157
H	-7.114353	-10.088086	-1.189577
H	-4.725504	-10.447206	-0.569754
H	-3.857410	-8.221195	-1.287061

H	-5.266897	-8.675290	-2.239122
H	-7.500321	-7.212727	2.001627
H	-7.207186	-8.908495	2.373720
H	-4.086109	-6.518789	0.600082
H	-5.654338	-5.799425	0.951245
H	-4.936288	-9.951663	1.866286
H	-3.660957	-8.977410	1.141781
H	-5.110877	-7.577822	2.613283
C	-6.142147	-6.248153	-7.315312
C	-6.447697	-7.551801	-6.561835
C	-6.207333	-7.350575	-5.057612
C	-4.741128	-6.954930	-4.824896
C	-4.430864	-5.649714	-5.573931
C	-4.676155	-5.855155	-7.076786
C	-7.124119	-6.231034	-4.540966
C	-7.059578	-5.131741	-6.792922
C	-5.352430	-4.534544	-5.056393
C	-6.820136	-4.924032	-5.289599
H	-6.313905	-6.393830	-8.387406
H	-7.486387	-7.855264	-6.740061
H	-5.811578	-8.361662	-6.939231
H	-6.426326	-8.281067	-4.522343
H	-4.551701	-6.827775	-3.752140
H	-4.075529	-7.754712	-5.171566
H	-3.384971	-5.369880	-5.407048
H	-4.439133	-4.935810	-7.625960
H	-4.009110	-6.635210	-7.463495
H	-8.175525	-6.509376	-4.682059
H	-6.975651	-6.090790	-3.463412
H	-6.865419	-4.199746	-7.337611
H	-8.109502	-5.391563	-6.974332
H	-5.173581	-4.364455	-3.987796
H	-5.126946	-3.591648	-5.569296
H	-7.475668	-4.128059	-4.919525
C	-0.509359	-5.421785	-2.293079
C	-2.029525	-5.641422	-2.255154
C	-2.733090	-4.334703	-1.857114
C	-2.243279	-3.895367	-0.468763
C	-0.723548	-3.671236	-0.501787
C	-0.025135	-4.979534	-0.903684
C	-2.396801	-3.242346	-2.883981
C	-0.179082	-4.326867	-3.319246
C	-0.392208	-2.580927	-1.532405
C	-0.877358	-3.016841	-2.923396
H	-0.009737	-6.354518	-2.576797
H	-2.386119	-5.977515	-3.236283
H	-2.276644	-6.434703	-1.539200
H	-3.816793	-4.493466	-1.831262
H	-2.753482	-2.973462	-0.164394
H	-2.494704	-4.658063	0.278187
H	-0.376509	-3.358638	0.489119
H	1.062521	-4.838452	-0.911804
H	-0.236925	-5.761397	-0.164216
H	-2.759557	-3.533830	-3.877098

H	-2.909098	-2.308880	-2.621253
H	0.905907	-4.174756	-3.370824
H	-0.502947	-4.638485	-4.319628
H	-0.869484	-1.635745	-1.246651
H	0.688874	-2.396876	-1.551196
H	-0.640760	-2.238068	-3.656734
C	-1.309178	-2.235737	-9.442179
C	-2.270365	-1.137577	-8.961818
C	-2.071291	-0.894951	-7.457672
C	-2.353790	-2.195753	-6.690499
C	-1.392935	-3.296927	-7.164925
C	-1.593450	-3.533138	-8.669898
C	-0.621023	-0.457652	-7.200213
C	0.139076	-1.794490	-9.179868
C	0.055649	-2.853432	-6.908699
C	0.344262	-1.554312	-7.676316
H	-1.452877	-2.407289	-10.514574
H	-2.092015	-0.210437	-9.519792
H	-3.307694	-1.432928	-9.160993
H	-2.757159	-0.111276	-7.117489
H	-2.234423	-2.030248	-5.612901
H	-3.392704	-2.509026	-6.849785
H	-1.596509	-4.223456	-6.617065
H	-0.926736	-4.331577	-9.017682
H	-2.618821	-3.870505	-8.864261
H	-0.411479	0.481837	-7.725844
H	-0.471304	-0.262591	-6.131397
H	0.836574	-2.561719	-9.537432
H	0.361048	-0.878685	-9.740928
H	0.217633	-2.699831	-5.834927
H	0.751559	-3.640095	-7.224666
H	1.377266	-1.239376	-7.492012
C	-8.571365	-0.017423	-6.769544
C	-8.088570	0.397267	-5.371143
C	-6.568207	0.618718	-5.391331
C	-5.869602	-0.684010	-5.810016
C	-6.346614	-1.101796	-7.209595
C	-7.867598	-1.318458	-7.184870
C	-6.228398	1.725218	-6.401766
C	-8.226637	1.091129	-7.776134
C	-6.007851	0.009205	-8.215404
C	-6.706697	1.313674	-7.802827
H	-9.655341	-0.174997	-6.752900
H	-8.598674	1.314577	-5.053232
H	-8.345888	-0.378390	-4.639639
H	-6.226279	0.914835	-4.393490
H	-4.781769	-0.544459	-5.808885
H	-6.087928	-1.478153	-5.085774
H	-5.847702	-2.030996	-7.505774
H	-8.218583	-1.636625	-8.174084
H	-8.121344	-2.124145	-6.485255
H	-6.704313	2.667265	-6.103626
H	-5.146740	1.906203	-6.411458
H	-8.584705	0.816089	-8.775703

H	-8.738357	2.021428	-7.501461
H	-4.922441	0.160783	-8.256957
H	-6.325286	-0.285714	-9.222986
H	-6.463594	2.104716	-8.520742
C	-4.531693	7.357661	2.604984
C	-4.809898	7.061233	1.123324
C	-3.871058	5.948845	0.631258
C	-2.412761	6.402429	0.798823
C	-2.128117	6.697343	2.279671
C	-3.071680	7.807530	2.767643
C	-4.107121	4.678662	1.462943
C	-4.766570	6.083753	3.431385
C	-2.369037	5.425460	3.107323
C	-3.827133	4.969342	2.945584
H	-5.201826	8.151031	2.953689
H	-5.855411	6.758296	0.989673
H	-4.663759	7.968677	0.524876
H	-4.071541	5.738761	-0.425131
H	-1.732440	5.624230	0.432180
H	-2.224951	7.298012	0.194246
H	-1.088127	7.021042	2.396669
H	-2.865253	8.041753	3.819067
H	-2.895293	8.728188	2.198209
H	-5.139729	4.331500	1.336314
H	-3.455782	3.870675	1.108332
H	-4.591001	6.287582	4.494694
H	-5.811028	5.762319	3.338936
H	-1.687739	4.630112	2.781636
H	-2.149526	5.617290	4.164669
H	-3.996880	4.062176	3.535909
C	-2.598700	1.845215	-4.391207
C	-3.558162	2.938207	-3.895843
C	-3.333506	3.185452	-2.396059
C	-3.593630	1.884557	-1.621167
C	-2.634374	0.788563	-2.110584
C	-2.860526	0.547722	-3.611183
C	-1.881693	3.632679	-2.165802
C	-1.148838	2.296389	-4.156071
C	-1.184307	1.241973	-1.881543
C	-0.918067	2.541223	-2.656985
H	-2.760646	1.670359	-5.460463
H	-3.395957	3.865239	-4.458900
H	-4.596980	2.635721	-4.075595
H	-4.018190	3.965433	-2.045169
H	-3.455878	2.053220	-0.546256
H	-4.633197	1.564227	-1.760971
H	-2.821985	-0.138044	-1.557185
H	-2.195034	-0.247163	-3.969294
H	-3.887029	0.203309	-3.786252
H	-1.687796	4.572333	-2.697111
H	-1.713945	3.831075	-1.100282
H	-0.452947	1.532889	-4.524582
H	-0.943001	3.212361	-4.722979
H	-1.003938	1.398999	-0.811203

H	-0.489125	0.459122	-2.208395
H	0.116032	2.863233	-2.492065
C	-9.855715	3.932668	-1.610368
C	-9.127017	2.640103	-2.008938
C	-7.614010	2.898161	-2.080840
C	-7.332782	3.988869	-3.125690
C	-8.056881	5.285101	-2.730254
C	-9.568717	5.020854	-2.656293
C	-7.112089	3.368746	-0.707008
C	-9.348109	4.400098	-0.237491
C	-7.554262	5.749453	-1.354577
C	-7.835954	4.663248	-0.305265
H	-10.934110	3.746581	-1.559911
H	-9.339875	1.847932	-1.281004
H	-9.494573	2.285782	-2.979665
H	-7.096453	1.975176	-2.364600
H	-6.253411	4.168229	-3.200835
H	-7.668625	3.657631	-4.115815
H	-7.855420	6.061070	-3.476803
H	-10.099761	5.943978	-2.393997
H	-9.944304	4.708262	-3.638229
H	-7.287921	2.590545	0.045536
H	-6.029000	3.538003	-0.740247
H	-9.875809	5.311767	0.068106
H	-9.563977	3.639536	0.522579
H	-6.478865	5.960181	-1.398534
H	-8.049125	6.685835	-1.069510
H	-7.476462	4.995835	0.674736
C	4.039264	0.464564	-2.587160
C	4.396988	1.525084	-3.639735
C	3.947741	1.049007	-5.029872
C	4.656917	-0.270870	-5.369641
C	4.300079	-1.336255	-4.321534
C	4.748054	-0.853914	-2.933150
C	2.428033	0.822639	-5.023811
C	2.519190	0.240245	-2.587333
C	2.779456	-1.556296	-4.316850
C	2.065509	-0.239738	-3.974505
H	4.360561	0.805799	-1.596990
H	3.913543	2.478182	-3.393530
H	5.478430	1.707907	-3.636224
H	4.203261	1.806868	-5.778633
H	4.359514	-0.611103	-6.368966
H	5.742758	-0.118986	-5.396761
H	4.807175	-2.276151	-4.565455
H	4.516810	-1.614689	-2.177694
H	5.835611	-0.712720	-2.916944
H	1.907670	1.762417	-4.802733
H	2.091940	0.500747	-6.016918
H	2.248291	-0.500527	-1.825186
H	2.001419	1.170203	-2.323040
H	2.448954	-1.919509	-5.297495
H	2.513394	-2.329984	-3.586383
H	0.981506	-0.398423	-3.972163

C	3.719547	-5.214659	-2.491237
C	3.419334	-6.535084	-1.765334
C	4.395353	-7.622426	-2.240760
C	4.236299	-7.822041	-3.755786
C	4.538493	-6.504944	-4.487113
C	3.562380	-5.420348	-4.005657
C	5.835426	-7.183023	-1.934321
C	5.161307	-4.781494	-2.183595
C	5.978013	-6.068037	-4.174656
C	6.141538	-5.864046	-2.660756
H	3.022553	-4.440976	-2.151057
H	3.507952	-6.397349	-0.680941
H	2.385904	-6.845323	-1.962122
H	4.179854	-8.562769	-1.721528
H	4.914203	-8.610579	-4.104370
H	3.217271	-8.155212	-3.986946
H	4.423952	-6.649468	-5.566947
H	3.754630	-4.479155	-4.534961
H	2.531374	-5.710763	-4.241592
H	5.969131	-7.056627	-0.853126
H	6.541205	-7.960122	-2.251855
H	5.381683	-3.828550	-2.679911
H	5.282026	-4.612864	-1.106678
H	6.686676	-6.825468	-4.530904
H	6.213288	-5.138674	-4.707475
H	7.168558	-5.553115	-2.439848
C	-8.058080	-0.901175	-0.192057
C	-8.313244	-1.104964	-1.693372
C	-7.381851	-2.200849	-2.234201
C	-5.920417	-1.780900	-2.014428
C	-5.658823	-1.578675	-0.513902
C	-6.594895	-0.484669	0.022628
C	-7.651726	-3.514187	-1.483800
C	-8.326728	-2.217936	0.552870
C	-5.933506	-2.893394	0.232288
C	-7.394882	-3.316221	0.018183
H	-8.722861	-0.119433	0.191422
H	-9.360432	-1.383029	-1.863103
H	-8.142864	-0.166083	-2.234136
H	-7.565887	-2.344853	-3.304566
H	-5.244899	-2.546597	-2.414730
H	-5.708437	-0.854148	-2.561285
H	-4.616547	-1.278815	-0.359654
H	-6.404472	-0.316371	1.089577
H	-6.394616	0.465208	-0.487861
H	-8.686788	-3.837012	-1.648927
H	-7.005839	-4.309873	-1.874373
H	-8.167818	-2.080195	1.629332
H	-9.373890	-2.516919	0.423180
H	-5.258016	-3.678648	-0.128139
H	-5.730731	-2.767965	1.302856
H	-7.588710	-4.253973	0.550442
C	2.538737	-1.161309	2.577147
C	2.304628	-1.360752	1.071823

C	3.268830	-2.429180	0.533557
C	4.716888	-1.974446	0.771870
C	4.957472	-1.776402	2.276464
C	3.988685	-0.709938	2.810368
C	3.024013	-3.754135	1.272034
C	2.295291	-2.489617	3.310141
C	4.707928	-3.102827	3.010670
C	3.259940	-3.560476	2.778005
H	1.850578	-0.399147	2.958794
H	1.266582	-1.663662	0.888964
H	2.457149	-0.414098	0.539341
H	3.099773	-2.570138	-0.539680
H	5.415556	-2.720182	0.373626
H	4.911361	-1.038836	0.233659
H	5.990226	-1.451716	2.443937
H	4.163554	-0.544510	3.880424
H	4.170499	0.248240	2.308555
H	1.999160	-4.101519	1.093604
H	3.693709	-4.530658	0.882997
H	2.439323	-2.355590	4.389163
H	1.257372	-2.813721	3.167177
H	5.406637	-3.868397	2.652164
H	4.896180	-2.979972	4.084184
H	3.084041	-4.506527	3.301739
C	0.209375	-5.767206	4.347140
C	-0.775050	-4.729296	4.907705
C	-2.217564	-5.213117	4.693177
C	-2.472577	-5.402791	3.190034
C	-1.492511	-6.442407	2.624724
C	-0.051840	-5.955645	2.844896
C	-2.417398	-6.554210	5.415899
C	0.003335	-7.106658	5.071269
C	-1.693767	-7.780281	3.352956
C	-1.436874	-7.596668	4.856464
H	1.237049	-5.420277	4.500876
H	-0.586217	-4.569705	5.976164
H	-0.624874	-3.762827	4.411380
H	-2.917670	-4.471890	5.094079
H	-3.506113	-5.729140	3.022233
H	-2.352477	-4.447893	2.664106
H	-1.675779	-6.575599	1.552940
H	0.658122	-6.680128	2.427712
H	0.111315	-5.010695	2.312516
H	-2.258543	-6.429019	6.493826
H	-3.449769	-6.900954	5.286238
H	0.714819	-7.851359	4.694163
H	0.205261	-6.990187	6.142913
H	-2.713457	-8.148749	3.187781
H	-1.013361	-8.537272	2.944327
H	-1.581350	-8.551376	5.374064
C	-1.470971	-8.422817	-6.957737
C	-1.110333	-7.285746	-7.926031
C	0.411671	-7.075113	-7.935563
C	0.884200	-6.712389	-6.519372

C	0.528708	-7.847872	-5.547153
C	-0.993554	-8.056811	-5.544022
C	1.102165	-8.371935	-8.385299
C	-0.775818	-9.716145	-7.410520
C	1.218461	-9.142885	-6.003129
C	0.746884	-9.511750	-7.417979
H	-2.556361	-8.571172	-6.952280
H	-1.463857	-7.524956	-8.936245
H	-1.616075	-6.360593	-7.623926
H	0.666554	-6.264393	-8.626978
H	1.967273	-6.539749	-6.517049
H	0.413547	-5.776996	-6.193211
H	0.866012	-7.586880	-4.538115
H	-1.262840	-8.852196	-4.838464
H	-1.497520	-7.145341	-5.200191
H	0.789485	-8.631977	-9.403850
H	2.189070	-8.228398	-8.414761
H	-1.041876	-10.541172	-6.738552
H	-1.122384	-9.999092	-8.411815
H	2.307440	-9.013247	-5.991402
H	0.988908	-9.957358	-5.305344
H	1.240081	-10.434944	-7.741582

Adamantane$_{18}$

468

E=-707.1818

C	-1.738897	-1.347107	2.801062
C	-1.249426	-0.877582	4.179666
C	-1.960641	0.428532	4.565922
C	-1.650036	1.506806	3.516495
C	-2.140394	1.043595	2.135954
C	-1.429027	-0.264303	1.756078
C	-3.477338	0.187365	4.613457
C	-3.256148	-1.584038	2.853592
C	-3.656537	0.800375	2.189672
C	-3.972266	-0.279585	3.235735
H	-1.230520	-2.278151	2.527529
H	-1.446077	-1.650455	4.932478
H	-0.163762	-0.722972	4.160120
H	-1.610574	0.761127	5.549344
H	-2.136251	2.450555	3.791792
H	-0.571444	1.703178	3.485964
H	-1.917430	1.813587	1.389329
H	-1.755727	-0.595703	0.762898
H	-0.346494	-0.099249	1.693958
H	-3.715705	-0.566171	5.373852
H	-3.995409	1.108333	4.907146
H	-3.615159	-1.939632	1.880112
H	-3.489527	-2.369014	3.583048
H	-4.178192	1.731720	2.441249
H	-4.022817	0.488477	1.204013
H	-5.053650	-0.451252	3.273005
C	1.431309	-9.904505	-0.584573

C	0.456003	-8.815093	-0.112827
C	-0.988571	-9.249825	-0.404662
C	-1.158148	-9.471118	-1.915529
C	-0.186966	-10.562200	-2.392238
C	1.255539	-10.124304	-2.094961
C	-1.285054	-10.561972	0.337739
C	1.128614	-11.214739	0.158804
C	-0.485080	-11.870926	-1.644509
C	-0.313888	-11.655748	-0.133007
H	2.460484	-9.592524	-0.375821
H	0.585063	-8.633340	0.960970
H	0.674311	-7.869042	-0.623006
H	-1.682207	-8.471914	-0.067010
H	-2.191495	-9.762708	-2.139199
H	-0.968476	-8.536305	-2.456828
H	-0.309231	-10.717885	-3.469671
H	1.960665	-10.886312	-2.448666
H	1.488156	-9.201233	-2.639761
H	-1.187829	-10.413188	1.419980
H	-2.320383	-10.872926	0.152710
H	1.832001	-11.995894	-0.154310
H	1.268838	-11.076465	1.237681
H	-1.506437	-12.205037	-1.863634
H	0.188763	-12.664053	-1.990703
H	-0.527352	-12.589680	0.398456
C	2.780039	4.510104	2.462235
C	3.142386	5.556699	1.397375
C	2.685808	5.069026	0.013660
C	3.384535	3.740516	-0.313891
C	3.023025	2.689002	0.746551
C	3.478371	3.182928	2.128449
C	1.164539	4.853690	0.026324
C	1.258399	4.296752	2.468644
C	1.500882	2.479984	0.757773
C	0.797369	3.805238	1.087949
H	3.106571	4.859589	3.447804
H	2.666488	6.515835	1.634678
H	4.225117	5.731753	1.395954
H	2.944643	5.816973	-0.743879
H	3.081876	3.391763	-1.308690
H	4.471356	3.884275	-0.345621
H	3.522675	1.742922	0.511326
H	3.243811	2.431965	2.892649
H	4.566955	3.316449	2.140152
H	0.651558	5.799504	0.238747
H	0.823341	4.523622	-0.962348
H	0.984348	3.566147	3.239429
H	0.748063	5.233190	2.724377
H	1.165014	2.108692	-0.218010
H	1.231342	1.716091	1.497219
H	-0.287734	3.654400	1.094971
C	-5.630162	-7.108993	-0.766784
C	-7.149851	-7.316095	-0.676032
C	-7.463732	-8.413481	0.352646

C	-6.792077	-9.725278	-0.081538
C	-5.271337	-9.524617	-0.170937
C	-4.963068	-8.424112	-1.197975
C	-6.918953	-7.993950	1.726691
C	-5.090468	-6.692398	0.610215
C	-4.732091	-9.101797	1.204262
C	-5.398527	-7.788517	1.641757
H	-5.408576	-6.326501	-1.500758
H	-7.641231	-6.378709	-0.388655
H	-7.550104	-7.595751	-1.658143
H	-8.547737	-8.559056	0.416112
H	-7.025401	-10.522249	0.634880
H	-7.186622	-10.046985	-1.052976
H	-4.794669	-10.460890	-0.481144
H	-3.878758	-8.284551	-1.286066
H	-5.324861	-8.722916	-2.189338
H	-7.405416	-7.068793	2.058766
H	-7.153855	-8.761115	2.474430
H	-4.008537	-6.521322	0.554037
H	-5.545132	-5.744197	0.921589
H	-4.928781	-9.887890	1.943308
H	-3.643605	-8.974885	1.158902
H	-5.013254	-7.488925	2.622603
C	-6.152060	-6.251672	-7.298417
C	-6.457747	-7.558307	-6.550188
C	-6.212176	-7.364733	-5.045802
C	-4.744463	-6.973050	-4.815910
C	-4.434044	-5.664878	-5.559705
C	-4.684545	-5.862664	-7.062727
C	-7.125111	-6.245894	-4.520883
C	-7.065627	-5.135988	-6.767767
C	-5.351765	-4.550404	-5.033900
C	-6.820969	-4.935924	-5.264248
H	-6.327534	-6.391895	-8.370637
H	-7.497594	-7.858898	-6.726525
H	-5.824425	-8.367591	-6.933483
H	-6.431275	-8.297343	-4.514276
H	-4.551354	-6.851387	-3.743174
H	-4.081542	-7.772459	-5.168525
H	-3.387077	-5.387875	-5.394848
H	-4.447485	-4.941170	-7.608270
H	-4.020270	-6.642158	-7.455291
H	-8.177506	-6.521517	-4.659926
H	-6.972920	-6.111084	-3.443155
H	-6.871390	-4.201782	-7.308626
H	-8.116634	-5.392902	-6.947043
H	-5.169164	-4.385767	-3.965084
H	-5.126079	-3.605509	-5.543022
H	-7.473755	-4.140456	-4.888280
C	-8.679921	-4.342262	5.404741
C	-8.360749	-3.267592	4.354124
C	-6.838856	-3.090108	4.238815
C	-6.271845	-2.660792	5.600664
C	-6.585606	-3.733771	6.654787

C	-8.108128	-3.909907	6.763638
C	-6.201146	-4.423874	3.820264
C	-8.037691	-5.672805	4.982671
C	-5.948995	-5.066057	6.229962
C	-6.515082	-5.501450	4.869733
H	-9.765282	-4.467053	5.485533
H	-8.781288	-3.553989	3.382602
H	-8.829985	-2.316825	4.634859
H	-6.613599	-2.323888	3.488847
H	-5.187874	-2.511283	5.526415
H	-6.704372	-1.699235	5.902632
H	-6.180939	-3.425350	7.624925
H	-8.346596	-4.659743	7.527736
H	-8.573211	-2.970563	7.086608
H	-6.581823	-4.732388	2.839071
H	-5.115867	-4.305234	3.715726
H	-8.275577	-6.454131	5.714748
H	-8.451503	-6.002525	4.022045
H	-4.859304	-4.959090	6.167117
H	-6.148643	-5.836214	6.984922
H	-6.059682	-6.451167	4.568240
C	-0.514082	-5.420701	-2.297122
C	-2.033980	-5.641191	-2.253770
C	-2.736527	-4.336166	-1.848441
C	-2.241284	-3.901311	-0.460607
C	-0.721804	-3.676344	-0.499040
C	-0.024423	-4.982938	-0.908210
C	-2.404917	-3.240170	-2.872951
C	-0.188483	-4.322149	-3.320897
C	-0.395157	-2.582386	-1.527287
C	-0.885752	-3.013807	-2.917768
H	-0.015184	-6.352228	-2.586031
H	-2.394401	-5.974122	-3.234578
H	-2.277821	-6.437015	-1.539516
H	-3.820042	-4.495532	-1.818729
H	-2.750673	-2.980687	-0.151040
H	-2.489319	-4.666657	0.284764
H	-0.370887	-3.366946	0.491505
H	1.063125	-4.841311	-0.920268
H	-0.232845	-5.767407	-0.170547
H	-2.771569	-3.528454	-3.865570
H	-2.916575	-2.307845	-2.604976
H	0.896217	-4.169347	-3.376363
H	-0.516265	-4.630525	-4.321008
H	-0.871706	-1.638407	-1.236389
H	0.685754	-2.397757	-1.549842
H	-0.652495	-2.232438	-3.649412
C	-1.317072	-2.242638	-9.436997
C	-2.277359	-1.143734	-8.956536
C	-2.076799	-0.900118	-7.452747
C	-2.359135	-2.200255	-6.684386
C	-1.399176	-3.302169	-7.158909
C	-1.601174	-3.539369	-8.663529
C	-0.626113	-0.463243	-7.196935

C	0.131607	-1.801809	-9.176333
C	0.049828	-2.859096	-6.904333
C	0.338276	-1.560645	-7.673142
H	-1.461830	-2.414896	-10.509136
H	-2.099135	-0.217067	-9.515335
H	-3.314993	-1.438794	-9.154545
H	-2.762027	-0.115915	-7.112492
H	-2.238707	-2.034032	-5.607017
H	-3.398326	-2.513208	-6.842493
H	-1.602633	-4.228221	-6.610202
H	-0.935114	-4.338334	-9.011356
H	-2.626864	-3.876446	-8.856707
H	-0.416660	0.475784	-7.723428
H	-0.475329	-0.267481	-6.128396
H	0.828455	-2.569585	-9.533990
H	0.353445	-0.886498	-9.738250
H	0.212863	-2.704797	-5.830821
H	0.745119	-3.646275	-7.220378
H	1.371581	-1.246009	-7.490013
C	-8.571355	-0.016691	-6.788977
C	-8.094216	0.395304	-5.387841
C	-6.573520	0.614989	-5.401020
C	-5.874616	-0.687928	-5.818619
C	-6.345970	-1.103025	-7.220913
C	-7.867302	-1.317926	-7.203192
C	-6.227978	1.722618	-6.408268
C	-8.220909	1.092979	-7.792355
C	-6.001490	0.009100	-8.223532
C	-6.700606	1.313767	-7.812040
H	-9.655578	-0.173009	-6.777329
H	-8.604632	1.312733	-5.070774
H	-8.355653	-0.381157	-4.658655
H	-6.235627	0.909186	-4.401239
H	-4.786634	-0.549665	-5.812503
H	-6.097052	-1.482910	-5.096552
H	-5.846855	-2.032363	-7.516315
H	-8.214315	-1.634176	-8.194420
H	-8.125063	-2.124373	-6.505924
H	-6.704090	2.664774	-6.110785
H	-5.146076	1.902339	-6.412938
H	-8.574909	0.819880	-8.793903
H	-8.732735	2.023464	-7.518513
H	-4.915730	0.159459	-8.260092
H	-6.314845	-0.283914	-9.232946
H	-6.453424	2.105610	-8.527676
C	-4.546719	7.331857	2.635049
C	-4.830492	7.043108	1.152929
C	-3.893856	5.932951	0.651713
C	-2.434795	6.385199	0.816212
C	-2.144586	6.672436	2.297489
C	-3.085962	7.780416	2.794617
C	-4.127282	4.658603	1.477752
C	-4.778981	6.053811	3.455781
C	-2.382886	5.396411	3.119504

C	-3.841725	4.941599	2.960817
H	-5.215289	8.123649	2.990283
H	-5.876595	6.741197	1.021591
H	-4.686252	7.953557	0.558598
H	-4.098308	5.728342	-0.404990
H	-1.756098	5.608667	0.443081
H	-2.248910	7.283805	0.215537
H	-1.104061	6.995195	2.412301
H	-2.875576	8.009186	3.846457
H	-2.911362	8.703920	2.229255
H	-5.160469	4.312428	1.353161
H	-3.477533	3.852232	1.116607
H	-4.599419	6.252137	4.519464
H	-5.823883	5.733193	3.365546
H	-1.703067	4.602520	2.787237
H	-2.159408	5.582754	4.177000
H	-4.009602	4.031477	3.547115
C	-2.602341	1.848132	-4.389321
C	-3.561629	2.939461	-3.889969
C	-3.336558	3.181575	-2.389410
C	-3.596520	1.878052	-1.618894
C	-2.637436	0.783700	-2.112310
C	-2.864003	0.547993	-3.613661
C	-1.884667	3.627961	-2.158020
C	-1.152399	2.298449	-4.153039
C	-1.187291	1.236274	-1.882115
C	-0.921213	2.538155	-2.653189
H	-2.764583	1.676933	-5.459124
H	-3.399542	3.868404	-4.449902
H	-4.600507	2.637626	-4.070471
H	-4.021119	3.960378	-2.035674
H	-3.458471	2.043039	-0.543450
H	-4.636136	1.558238	-1.759509
H	-2.824931	-0.144784	-1.562027
H	-2.198638	-0.245689	-3.974664
H	-3.890566	0.204216	-3.789627
H	-1.690879	4.569417	-2.686171
H	-1.716623	3.822712	-1.091875
H	-0.456636	1.536186	-4.524343
H	-0.946682	3.216344	-4.716872
H	-1.006627	1.389639	-0.811294
H	-0.492226	0.454519	-2.211826
H	0.112942	2.859563	-2.487450
C	-9.860613	3.958058	-1.643419
C	-9.131018	2.667250	-2.046024
C	-7.616981	2.922931	-2.103223
C	-7.324955	4.021258	-3.137073
C	-8.049917	5.315748	-2.737534
C	-9.562813	5.053860	-2.678316
C	-7.125722	3.381748	-0.721578
C	-9.363666	4.413716	-0.262707
C	-7.557988	5.768315	-1.354088
C	-7.850506	4.674458	-0.315745
H	-10.939740	3.773673	-1.603459

H	-9.351458	1.869806	-1.326150
H	-9.491081	2.321271	-3.022552
H	-7.098791	2.001200	-2.389888
H	-6.244658	4.199109	-3.201764
H	-7.653100	3.698451	-4.132549
H	-7.840748	6.097159	-3.476245
H	-10.094309	5.975925	-2.413225
H	-9.930734	4.749712	-3.665790
H	-7.309317	2.598008	0.023324
H	-6.042077	3.549160	-0.744415
H	-9.892203	5.323980	0.045619
H	-9.587327	3.647633	0.489525
H	-6.481867	5.977297	-1.387380
H	-8.053470	6.703390	-1.065820
H	-7.498625	4.998647	0.669817
C	4.036040	0.472190	-2.597214
C	4.389306	1.534861	-3.649125
C	3.940303	1.058625	-5.039287
C	4.653462	-0.258609	-5.380967
C	4.301093	-1.326138	-4.333530
C	4.748802	-0.843642	-2.945114
C	2.421348	0.827292	-5.032010
C	2.516705	0.242909	-2.596169
C	2.781201	-1.551147	-4.327622
C	2.063285	-0.237249	-3.983365
H	4.357155	0.813541	-1.607025
H	3.902985	2.486144	-3.401567
H	5.470148	1.721209	-3.646459
H	4.192641	1.818020	-5.787572
H	4.356228	-0.598872	-6.380332
H	5.738776	-0.103155	-5.408965
H	4.811024	-2.264144	-4.578813
H	4.520755	-1.605877	-2.190158
H	5.835907	-0.698913	-2.929798
H	1.898130	1.765158	-4.809555
H	2.085371	0.505238	-6.025102
H	2.248946	-0.499459	-1.834465
H	1.996152	1.170923	-2.330512
H	2.450962	-1.914515	-5.308297
H	2.518356	-2.326386	-3.597635
H	0.979808	-0.399475	-3.980152
C	3.714771	-5.207611	-2.501883
C	3.413468	-6.527553	-1.775554
C	4.388260	-7.615985	-2.251002
C	4.228546	-7.815825	-3.765929
C	4.531826	-6.499214	-4.497682
C	3.556937	-5.413527	-4.016203
C	5.828862	-7.177936	-1.945113
C	5.157054	-4.775801	-2.194790
C	5.971874	-6.063659	-4.185774
C	6.136063	-5.859448	-2.671976
H	3.018650	-4.433150	-2.161686
H	3.502553	-6.389632	-0.691224
H	2.379669	-6.836815	-1.971948

H	4.171985	-8.555982	-1.731466
H	4.905560	-8.605125	-4.114519
H	3.209118	-8.148042	-3.996694
H	4.416812	-6.643897	-5.577444
H	3.749961	-4.472660	-4.545804
H	2.525572	-5.702977	-4.251751
H	5.963022	-7.051399	-0.863991
H	6.533772	-7.955816	-2.262665
H	5.378225	-3.823202	-2.691415
H	5.278269	-4.607019	-1.117953
H	6.679676	-6.821884	-4.542046
H	6.207910	-5.134665	-4.718900
H	7.163459	-5.549482	-2.451459
C	-8.056165	-0.858553	-0.216586
C	-8.314867	-1.075373	-1.715468
C	-7.390989	-2.182125	-2.246978
C	-5.926692	-1.768979	-2.033459
C	-5.661571	-1.553771	-0.535359
C	-6.590146	-0.448910	-0.008123
C	-7.667698	-3.486433	-1.483431
C	-8.331675	-2.166345	0.541518
C	-5.943102	-2.859471	0.223998
C	-7.407351	-3.275440	0.016174
H	-8.715588	-0.069080	0.160246
H	-9.363987	-1.348684	-1.880940
H	-8.139608	-0.142852	-2.265596
H	-7.577540	-2.335407	-3.315617
H	-5.256460	-2.542638	-2.427284
H	-5.709917	-0.848900	-2.589622
H	-4.617254	-1.258786	-0.385574
H	-6.397066	-0.271409	1.056855
H	-6.384875	0.494719	-0.528129
H	-8.704957	-3.804530	-1.643881
H	-7.027264	-4.289804	-1.867192
H	-8.170279	-2.019114	1.616354
H	-9.380835	-2.460183	0.416289
H	-5.272956	-3.652298	-0.129764
H	-5.737925	-2.724876	1.292993
H	-7.606064	-4.206772	0.557822
C	2.543449	-1.156028	2.559918
C	2.316988	-1.352146	1.052988
C	3.284376	-2.418905	0.517136
C	4.731008	-1.964057	0.763747
C	4.963948	-1.769333	2.269977
C	3.991998	-0.704530	2.801440
C	3.036475	-3.745651	1.251357
C	2.296947	-2.486113	3.288654
C	4.711342	-3.097542	2.999899
C	3.264753	-3.555318	2.758929
H	1.853028	-0.395050	2.939841
H	1.280014	-1.655110	0.864233
H	2.471738	-0.404212	0.523435
H	3.120773	-2.557494	-0.557254
H	5.432014	-2.708566	0.367318

H	4.927743	-1.027135	0.228651
H	5.995697	-1.444560	2.443370
H	4.161419	-0.541462	3.872734
H	4.175880	0.254872	2.302728
H	2.012693	-4.093094	1.066995
H	3.708477	-4.520981	0.863918
H	2.435501	-2.354481	4.368688
H	1.259910	-2.810363	3.139746
H	5.412198	-3.861975	2.643162
H	4.894148	-2.977048	4.074622
H	3.086670	-4.502640	3.279619
C	0.272414	-5.790557	4.390530
C	-0.712994	-4.728426	4.901836
C	-2.155427	-5.197163	4.655609
C	-2.370179	-5.413839	3.149826
C	-1.389016	-6.477712	2.633687
C	0.051454	-6.005896	2.885444
C	-2.394431	-6.520540	5.398805
C	0.027189	-7.112181	5.134923
C	-1.629554	-7.797741	3.382286
C	-1.413017	-7.587142	4.888608
H	1.300035	-5.454356	4.566805
H	-0.552438	-4.549751	5.971896
H	-0.535290	-3.774311	4.390917
H	-2.856252	-4.438668	5.021415
H	-3.402971	-5.729673	2.958918
H	-2.221855	-4.471488	2.608688
H	-1.543547	-6.630139	1.559939
H	0.762829	-6.748115	2.503315
H	0.242856	-5.074197	2.339293
H	-2.264587	-6.375662	6.478151
H	-3.427429	-6.856006	5.246453
H	0.738662	-7.873820	4.793294
H	0.200140	-6.976723	6.209399
H	-2.649169	-8.155812	3.195238
H	-0.948417	-8.571888	3.008504
H	-1.585466	-8.529145	5.420718
C	-1.477232	-8.430473	-6.965261
C	-1.118827	-7.287325	-7.927208
C	0.402689	-7.073122	-7.934944
C	0.873788	-6.717008	-6.516601
C	0.520524	-7.858577	-5.550718
C	-1.001251	-8.071052	-5.549374
C	1.096371	-8.365879	-8.391425
C	-0.778898	-9.719709	-7.424767
C	1.213464	-9.149494	-6.013429
C	0.743330	-9.511753	-7.430464
H	-2.562278	-8.581367	-6.961074
H	-1.471376	-7.521859	-8.938857
H	-1.626836	-6.365000	-7.620296
H	0.655980	-6.258069	-8.621834
H	1.956457	-6.541877	-6.512877
H	0.400835	-5.784493	-6.185564
H	0.856804	-7.602292	-4.540133

```
H     -1.268987     -8.870881     -4.848263
H     -1.507469     -7.162633     -5.200811
H      0.784717     -8.621105     -9.411507
H      2.182953     -8.219670     -8.419641
H     -1.043323    -10.548989     -6.757406
H     -1.124392     -9.998012     -8.427733
H      2.302135     -9.017401     -6.000532
H      0.985509     -9.968276     -5.320178
H      1.238798    -10.432031     -7.758868
```

Adamantane$_{19}$

```
         494
E=-769.9942
C     -1.700914     -1.357757      2.808790
C     -1.215213     -0.888077      4.188673
C     -1.912056      0.429014      4.563686
C     -1.579913      1.498136      3.511494
C     -2.066405      1.034773      2.129638
C     -1.369479     -0.284074      1.761014
C     -3.432073      0.207847      4.601421
C     -3.221466     -1.574658      2.851496
C     -3.585940      0.811574      2.173570
C     -3.923203     -0.259183      3.222368
H     -1.202795     -2.296635      2.543281
H     -1.427309     -1.654726      4.943654
H     -0.127511     -0.747712      4.176225
H     -1.564709      0.761708      5.548039
H     -2.055720      2.449438      3.778868
H     -0.498659      1.680299      3.487816
H     -1.828081      1.798227      1.381047
H     -1.693376     -0.615925      0.767067
H     -0.284467     -0.133429      1.705922
H     -3.685682     -0.538887      5.363594
H     -3.940161      1.136873      4.887063
H     -3.578127     -1.930184      1.877128
H     -3.470276     -2.353041      3.582913
H     -4.097174      1.750822      2.417026
H     -3.949212      0.499776      1.186767
H     -5.006975     -0.416574      3.252650
C      1.423603     -9.901942     -0.581032
C      0.451102     -8.810965     -0.107118
C     -0.994823     -9.243441     -0.395598
C     -1.168225     -9.464563     -1.906056
C     -0.199860    -10.557204     -2.384925
C      1.244012    -10.121561     -2.090997
C     -1.291663    -10.555072      0.347571
C      1.120556    -11.211649      0.163128
C     -0.498313    -11.865410     -1.636423
C     -0.323306    -11.650406     -0.125334
H      2.453745     -9.591569     -0.374669
H      0.582918     -8.629346      0.966368
H      0.669724     -7.865292     -0.617861
H     -1.686454     -8.464416     -0.056404
```

H	-2.202541	-9.754540	-2.127328
H	-0.978327	-8.530085	-2.447854
H	-0.324849	-10.712765	-3.462063
H	1.947122	-10.884701	-2.446271
H	1.476828	-9.198892	-2.636393
H	-1.191714	-10.406371	1.429576
H	-2.327903	-10.864407	0.164946
H	1.821990	-11.993932	-0.151550
H	1.263479	-11.073527	1.241671
H	-1.520698	-12.197927	-1.853175
H	0.173481	-12.659620	-1.984113
H	-0.537017	-12.583966	0.406683
C	2.793959	4.509635	2.456272
C	3.176569	5.550536	1.392920
C	2.726519	5.065545	0.006128
C	3.413656	3.729067	-0.313429
C	3.031868	2.683231	0.745519
C	3.480769	3.174485	2.130475
C	1.202967	4.866599	0.006195
C	1.270111	4.312666	2.450079
C	1.507522	2.490609	0.744128
C	0.815542	3.823887	1.066274
H	3.115864	4.857195	3.444042
H	2.709029	6.515118	1.624695
H	4.261095	5.713936	1.400430
H	2.999805	5.809430	-0.750335
H	3.115708	3.381967	-1.310227
H	4.502192	3.861080	-0.336144
H	3.523280	1.731451	0.516004
H	3.231665	2.427333	2.893809
H	4.570588	3.296313	2.151222
H	0.698415	5.818218	0.212796
H	0.866632	4.538607	-0.984831
H	0.981683	3.586308	3.219631
H	0.767734	5.254953	2.700025
H	1.175964	2.121359	-0.233901
H	1.223512	1.730865	1.482432
H	-0.271143	3.684736	1.064307
C	-5.628579	-7.088493	-0.759900
C	-7.148672	-7.292889	-0.669770
C	-7.465090	-8.387475	0.361111
C	-6.795292	-9.701335	-0.069689
C	-5.274161	-9.503378	-0.158451
C	-4.963351	-8.405661	-1.187703
C	-6.920593	-7.965800	1.734611
C	-5.089174	-6.669739	0.616557
C	-4.735191	-9.078403	1.216191
C	-5.399771	-7.763060	1.650304
H	-5.405184	-6.307995	-1.495446
H	-7.638709	-6.354061	-0.384819
H	-7.548690	-7.574061	-1.651545
H	-8.549378	-8.531123	0.424128
H	-7.030438	-10.496332	0.648325
H	-7.189680	-10.024544	-1.040694

H	-4.798818	-10.441121	-0.466245
H	-3.878750	-8.268082	-1.275332
H	-5.324934	-8.706064	-2.178659
H	-7.405764	-7.039109	2.064291
H	-7.157289	-8.730918	2.483880
H	-4.006923	-6.500570	0.560770
H	-5.542494	-5.720102	0.925507
H	-4.933700	-9.862531	1.956836
H	-3.646466	-8.953384	1.171326
H	-5.014698	-7.461931	2.630758
C	-6.159542	-6.239651	-7.283139
C	-6.468300	-7.542460	-6.529521
C	-6.220716	-7.343730	-5.026137
C	-4.751650	-6.955422	-4.799177
C	-4.438152	-5.651071	-5.548372
C	-4.690677	-5.853994	-7.050371
C	-7.129891	-6.220201	-4.504723
C	-7.069353	-5.119252	-6.755981
C	-5.352125	-4.531890	-5.026050
C	-6.822664	-4.914033	-5.253488
H	-6.336456	-6.383548	-8.354636
H	-7.509185	-7.840713	-6.703690
H	-5.837700	-8.355072	-6.910254
H	-6.442009	-8.273617	-4.490763
H	-4.557155	-6.830134	-3.727108
H	-4.091395	-7.758126	-5.149298
H	-3.390226	-5.376473	-5.385597
H	-4.451468	-4.935330	-7.599734
H	-4.029047	-6.636943	-7.440519
H	-8.183216	-6.493304	-4.641684
H	-6.976270	-6.081628	-3.427675
H	-6.872926	-4.187733	-7.300670
H	-8.121274	-5.373807	-6.933250
H	-5.168015	-4.363613	-3.958059
H	-5.124188	-3.589650	-5.539074
H	-7.472774	-4.115209	-4.880009
C	-8.669546	-4.332952	5.404178
C	-8.361600	-3.248650	4.360127
C	-6.842207	-3.044785	4.256027
C	-6.291910	-2.611524	5.623472
C	-6.594498	-3.694059	6.671072
C	-8.114500	-3.896560	6.768716
C	-6.178986	-4.365743	3.836506
C	-8.001837	-5.650597	4.981182
C	-5.932359	-5.013530	6.245276
C	-6.481640	-5.452858	4.879433
H	-9.753145	-4.476552	5.476982
H	-8.770552	-3.538137	3.384583
H	-8.848899	-2.307189	4.641453
H	-6.624948	-2.271732	3.510726
H	-5.210168	-2.443261	5.557300
H	-6.742835	-1.658738	5.926327
H	-6.201782	-3.382815	7.645213
H	-8.345344	-4.653514	7.528122

H	-8.597739	-2.966625	7.092257
H	-6.547640	-4.676625	2.851477
H	-5.095203	-4.228197	3.739931
H	-8.231343	-6.438894	5.708446
H	-8.403398	-5.983331	4.016405
H	-4.844249	-4.887752	6.190375
H	-6.123981	-5.790102	6.995731
H	-6.008051	-6.393418	4.577259
C	-0.515068	-5.417097	-2.288805
C	-2.035903	-5.630287	-2.241953
C	-2.731665	-4.320237	-1.841160
C	-2.232053	-3.881652	-0.456068
C	-0.711607	-3.663961	-0.498019
C	-0.021025	-4.975572	-0.902640
C	-2.396675	-3.230283	-2.871001
C	-0.186075	-4.324555	-3.317913
C	-0.381596	-2.576037	-1.531581
C	-0.876548	-3.011222	-2.919343
H	-0.021012	-6.352197	-2.574474
H	-2.399530	-5.965806	-3.220695
H	-2.282248	-6.421840	-1.523823
H	-3.815861	-4.474405	-1.808942
H	-2.736616	-2.957316	-0.149676
H	-2.482398	-4.642572	0.293054
H	-0.357577	-3.351888	0.490577
H	1.067151	-4.839085	-0.917131
H	-0.231859	-5.755834	-0.161208
H	-2.766346	-3.521178	-3.861738
H	-2.903523	-2.294417	-2.606249
H	0.899230	-4.177069	-3.375858
H	-0.516982	-4.635755	-4.316119
H	-0.853242	-1.628581	-1.244016
H	0.700125	-2.396563	-1.556747
H	-0.640883	-2.234151	-3.654783
C	-1.314680	-2.241323	-9.434971
C	-2.272032	-1.139697	-8.954887
C	-2.069807	-0.895089	-7.451482
C	-2.354639	-2.193809	-6.681649
C	-1.397617	-3.298438	-7.155793
C	-1.601266	-3.536628	-8.660033
C	-0.617913	-0.461375	-7.197147
C	0.135220	-1.803646	-9.175788
C	0.052608	-2.858525	-6.902699
C	0.343549	-1.561505	-7.672983
H	-1.460621	-2.414283	-10.506837
H	-2.092038	-0.213996	-9.514719
H	-3.310501	-1.432509	-9.151856
H	-2.752943	-0.108946	-7.111496
H	-2.233038	-2.026820	-5.604530
H	-3.394677	-2.504472	-6.838697
H	-1.602850	-4.223475	-6.606034
H	-0.937338	-4.337495	-9.007563
H	-2.627885	-3.871481	-8.852139
H	-0.406637	0.476643	-7.724708

H	-0.465892	-0.264928	-6.128910
H	0.830002	-2.573406	-9.533201
H	0.358800	-0.889406	-9.738758
H	0.216787	-2.703564	-5.829456
H	0.745818	-3.647643	-7.218479
H	1.377722	-1.249120	-7.490910
C	-8.562196	-0.010063	-6.788475
C	-8.084055	0.407048	-5.389195
C	-6.563312	0.626291	-5.404168
C	-5.865007	-0.678358	-5.817338
C	-6.337365	-1.098576	-7.217767
C	-7.858739	-1.313019	-7.198269
C	-6.218133	1.730056	-6.415773
C	-8.212110	1.095755	-7.796221
C	-5.993244	0.009702	-8.224759
C	-6.691765	1.316078	-7.817703
H	-9.646451	-0.166059	-6.775548
H	-8.594035	1.325787	-5.075236
H	-8.345223	-0.366613	-4.656943
H	-6.224704	0.924139	-4.405711
H	-4.776987	-0.540352	-5.812434
H	-6.087183	-1.470576	-5.092160
H	-5.838676	-2.029142	-7.510009
H	-8.206468	-1.632886	-8.188085
H	-8.116259	-2.116786	-6.497825
H	-6.693815	2.673440	-6.121512
H	-5.136189	1.909480	-6.421808
H	-8.566822	0.819003	-8.796513
H	-8.723525	2.027390	-7.525534
H	-4.907470	0.159643	-8.262576
H	-6.307320	-0.287005	-9.232869
H	-6.444839	2.105176	-8.536454
C	-4.605458	7.246449	2.643767
C	-4.834952	6.999296	1.144823
C	-3.846612	5.938376	0.636308
C	-2.409312	6.432580	0.860759
C	-2.173312	6.678463	2.359009
C	-3.166191	7.737192	2.863221
C	-4.061122	4.629558	1.412047
C	-4.818520	5.934157	3.414118
C	-2.392514	5.367947	3.130522
C	-3.829678	4.870991	2.911799
H	-5.310836	8.003182	3.004192
H	-5.865813	6.667737	0.971255
H	-4.704079	7.933772	0.585735
H	-4.012406	5.763352	-0.432413
H	-1.693803	5.692034	0.483274
H	-2.236246	7.356815	0.296230
H	-1.148239	7.031228	2.516586
H	-2.995145	7.936712	3.928069
H	-3.006296	8.684719	2.334445
H	-5.077777	4.253979	1.244153
H	-3.373874	3.857843	1.044652
H	-4.677586	6.101846	4.488834

H	-5.848771	5.582547	3.281027
H	-1.676511	4.608703	2.793319
H	-2.207117	5.525325	4.200019
H	-3.983936	3.936337	3.462141
C	-2.602701	1.861111	-4.385812
C	-3.565212	2.948722	-3.884555
C	-3.339476	3.190299	-2.384010
C	-3.594218	1.885227	-1.614373
C	-2.631894	0.794587	-2.109696
C	-2.859149	0.559412	-3.611026
C	-1.888888	3.641442	-2.153672
C	-1.154071	2.316172	-4.150575
C	-1.183074	1.251912	-1.880545
C	-0.922212	2.555364	-2.650747
H	-2.765422	1.690288	-5.455602
H	-3.406857	3.878699	-4.443838
H	-4.603233	2.643497	-4.064289
H	-4.026340	3.966452	-2.028915
H	-3.455662	2.049750	-0.538924
H	-4.632875	1.561986	-1.754235
H	-2.815668	-0.135009	-1.560036
H	-2.191436	-0.231679	-3.973382
H	-3.884706	0.212283	-3.786273
H	-1.698843	4.584013	-2.681194
H	-1.720449	3.835839	-1.087525
H	-0.456078	1.556614	-4.523235
H	-0.952052	3.235255	-4.713812
H	-1.001869	1.404963	-0.809771
H	-0.485672	0.472823	-2.211628
H	0.111004	2.880159	-2.485754
C	-9.881243	3.979120	-1.704537
C	-9.151981	2.688381	-2.107963
C	-7.636392	2.938449	-2.145489
C	-7.327934	4.043086	-3.167780
C	-8.052513	5.337512	-2.767339
C	-9.567009	5.081217	-2.727824
C	-7.159666	3.385262	-0.754848
C	-9.398832	4.422758	-0.314785
C	-7.575164	5.778060	-1.374935
C	-7.884149	4.677862	-0.348125
H	-10.961475	3.798741	-1.678614
H	-9.383989	1.886611	-1.396577
H	-9.501855	2.350937	-3.091158
H	-7.118449	2.016771	-2.432773
H	-6.246266	4.217106	-3.218466
H	-7.645579	3.728825	-4.169377
H	-7.831616	6.123430	-3.497808
H	-10.098002	6.003439	-2.462267
H	-9.924449	4.785714	-3.721752
H	-7.355074	2.596868	-0.017904
H	-6.075185	3.548527	-0.763721
H	-9.927421	5.332853	-0.006048
H	-9.634319	3.652127	0.429146
H	-6.497921	5.982998	-1.394046

H	-8.070367	6.712978	-1.085684
H	-7.542648	4.993482	0.643848
C	4.041965	0.472264	-2.602135
C	4.395397	1.533125	-3.655816
C	3.943487	1.056027	-5.044739
C	4.653933	-0.262788	-5.385973
C	4.301383	-1.328514	-4.336762
C	4.752007	-0.845155	-2.949589
C	2.424162	0.827232	-5.034883
C	2.522255	0.245516	-2.598513
C	2.781130	-1.550984	-4.328282
C	2.065925	-0.235494	-3.984459
H	4.365155	0.814228	-1.612833
H	3.911038	2.485503	-3.408624
H	5.476551	1.717676	-3.655010
H	4.195949	1.814133	-5.794289
H	4.354612	-0.603714	-6.384488
H	5.739459	-0.109175	-5.415802
H	4.809378	-2.267651	-4.581730
H	4.523842	-1.606133	-2.193402
H	5.839374	-0.702219	-2.936095
H	1.902844	1.766226	-4.812724
H	2.086138	0.504587	-6.027089
H	2.254421	-0.495522	-1.835542
H	2.003652	1.174703	-2.333143
H	2.448793	-1.914938	-5.308031
H	2.518106	-2.324937	-3.596995
H	0.982186	-0.395911	-3.979411
C	3.714048	-5.210836	-2.508183
C	3.410713	-6.529214	-1.779861
C	4.383647	-7.619934	-2.253867
C	4.223352	-7.821744	-3.768472
C	4.528656	-6.506709	-4.502213
C	3.555622	-5.418724	-4.022172
C	5.825015	-7.183787	-1.948864
C	5.157086	-4.780929	-2.201967
C	5.969466	-6.073046	-4.191188
C	6.134244	-5.866874	-2.677719
H	3.019249	-4.434739	-2.169012
H	3.500207	-6.389841	-0.695749
H	2.376378	-6.837076	-1.975627
H	4.165927	-8.558810	-1.732911
H	4.899018	-8.612661	-4.116012
H	3.203343	-8.152636	-3.998577
H	4.413223	-6.652794	-5.581742
H	3.750092	-4.478954	-4.553190
H	2.523746	-5.706836	-4.257121
H	5.959564	-7.055878	-0.867952
H	6.528600	-7.963284	-2.265387
H	5.379727	-3.829425	-2.700031
H	5.278758	-4.610759	-1.125400
H	6.675969	-6.832950	-4.546460
H	6.206928	-5.145225	-4.725721
H	7.162182	-5.558261	-2.457830

C	-8.061184	-0.842691	-0.199423
C	-8.310611	-1.058409	-1.700035
C	-7.375235	-2.156605	-2.229176
C	-5.915387	-1.732202	-2.007428
C	-5.659524	-1.518042	-0.507569
C	-6.599561	-0.421767	0.017270
C	-7.645310	-3.464696	-1.469736
C	-8.330006	-2.154244	0.554573
C	-5.934389	-2.827555	0.247653
C	-7.394195	-3.254792	0.031595
H	-8.728798	-0.059319	0.175717
H	-9.356663	-1.339759	-1.871356
H	-8.140073	-0.123355	-2.247340
H	-7.555184	-2.309118	-3.299056
H	-5.237007	-2.499637	-2.399474
H	-5.703204	-0.809244	-2.560585
H	-4.618370	-1.215024	-0.351915
H	-6.413259	-0.244975	1.083573
H	-6.399251	0.524572	-0.499739
H	-8.679168	-3.790746	-1.636068
H	-6.996539	-4.262102	-1.851930
H	-8.175193	-2.007996	1.630511
H	-9.376134	-2.456206	0.423452
H	-5.256142	-3.614243	-0.104370
H	-5.735668	-2.693580	1.317945
H	-7.588153	-4.188828	0.570304
C	2.572080	-1.164267	2.555167
C	2.347565	-1.364788	1.048526
C	3.314496	-2.434308	0.517373
C	4.761294	-1.980357	0.764665
C	4.992288	-1.781239	2.270620
C	4.020798	-0.713684	2.797383
C	3.064012	-3.758491	1.255336
C	2.323000	-2.491808	3.287658
C	4.737103	-3.106892	3.004282
C	3.290337	-3.563755	2.762640
H	1.881988	-0.401327	2.931738
H	1.310516	-1.667148	0.859212
H	2.504166	-0.418675	0.516267
H	3.152281	-2.576034	-0.556818
H	5.462012	-2.726891	0.371553
H	4.959876	-1.045321	0.226958
H	6.024158	-1.457113	2.444497
H	4.188863	-0.547495	3.868411
H	4.206501	0.243959	2.295972
H	2.040098	-4.105330	1.070571
H	3.735679	-4.535786	0.871267
H	2.460149	-2.356992	4.367478
H	1.285805	-2.815329	3.138256
H	5.437593	-3.873228	2.650923
H	4.918499	-2.983283	4.078889
H	3.110415	-4.509256	3.286000
C	-6.872157	2.640999	4.551139
C	-7.082721	1.320236	5.307334

C	-8.524358	0.829323	5.103274
C	-9.502592	1.887372	5.636226
C	-9.298391	3.209837	4.881047
C	-7.854664	3.694466	5.085335
C	-8.779949	0.609962	3.604159
C	-7.131654	2.416535	3.053399
C	-9.551520	2.984439	3.382429
C	-8.573521	1.929166	2.843763
H	-5.843959	2.989312	4.698102
H	-6.373189	0.564275	4.949818
H	-6.881924	1.463216	6.376067
H	-8.672062	-0.112690	5.642794
H	-10.535601	1.539651	5.514406
H	-9.344162	2.039652	6.710733
H	-9.996419	3.962773	5.263006
H	-7.701140	4.648867	4.567152
H	-7.667109	3.879162	6.150083
H	-8.101657	-0.158752	3.214500
H	-9.800610	0.240695	3.446909
H	-6.964520	3.348064	2.499037
H	-6.423749	1.679715	2.654995
H	-10.585478	2.656377	3.220796
H	-9.428710	3.926396	2.834159
H	-8.755833	1.769404	1.775358
C	0.279624	-5.791826	4.388990
C	-0.695396	-4.722881	4.905978
C	-2.142459	-5.181831	4.668649
C	-2.367831	-5.397394	3.164260
C	-1.397097	-6.468054	2.642465
C	0.048059	-6.006009	2.885334
C	-2.385957	-6.503372	5.413653
C	0.029927	-7.111571	5.135227
C	-1.642072	-7.786235	3.392879
C	-1.414955	-7.576761	4.897799
H	1.310566	-5.462596	4.558924
H	-0.527125	-4.545051	5.974993
H	-0.514301	-3.770120	4.393723
H	-2.835862	-4.418489	5.038496
H	-3.403893	-5.706225	2.979718
H	-2.216378	-4.456205	2.621968
H	-1.559186	-6.619677	1.569718
H	0.752023	-6.753151	2.499096
H	0.242484	-5.075766	2.337771
H	-2.248570	-6.359127	6.492149
H	-3.422124	-6.831824	5.267672
H	0.734102	-7.878123	4.789492
H	0.210323	-6.977041	6.208594
H	-2.665223	-8.137390	3.212128
H	-0.968512	-8.565096	3.015180
H	-1.590582	-8.517440	5.431209
C	-1.491683	-8.426089	-6.954415
C	-1.132124	-7.286506	-7.920154
C	0.390027	-7.077070	-7.932221
C	0.865773	-6.718834	-6.515965

```
C      0.511369     -7.856845     -5.546307
C     -1.011051     -8.064568     -5.540636
C      1.078540     -8.373137     -8.387140
C     -0.798514     -9.718657     -7.412380
C      1.199130     -9.151085     -6.007459
C      0.724334     -9.515466     -7.422394
H     -2.577179     -8.573586     -6.947145
H     -1.487926     -7.522502     -8.930323
H     -1.636492     -6.361826     -7.614324
H      0.644138     -6.264554     -8.621809
H      1.948987     -6.547072     -6.515380
H      0.396552     -5.784012     -6.186122
H      0.850967     -7.599050     -4.537217
H     -1.279524     -8.861779     -4.836830
H     -1.513568     -7.153694     -5.193123
H      0.763547     -8.629973     -9.405791
H      2.165498     -8.230389     -8.418432
H     -1.063852    -10.545415     -6.742257
H     -1.147376     -9.998421     -8.413773
H      2.288235     -9.022357     -5.997608
H      0.970359     -9.967393     -5.311564
H      1.216114    -10.438114     -7.749691
```

Adamantane$_{20}$

```
        520
E=-811.9795
C     -1.696901     -1.345080      2.767740
C     -1.214915     -0.878044      4.149822
C     -1.916157      0.435959      4.527461
C     -1.585256      1.509085      3.478962
C     -2.068046      1.048369      2.094924
C     -1.366728     -0.267407      1.723668
C     -3.435599      0.210355      4.561926
C     -3.216899     -1.566433      2.807186
C     -3.587011      0.820718      2.135580
C     -3.923022     -0.254049      3.180673
H     -1.195649     -2.281757      2.500360
H     -1.426121     -1.647491      4.902201
H     -0.127598     -0.734546      4.139662
H     -1.571454      0.766769      5.513377
H     -2.064233      2.458245      3.748280
H     -0.504486      1.694394      3.457683
H     -1.830614      1.814679      1.348971
H     -1.687967     -0.597279      0.728201
H     -0.282057     -0.113513      1.670890
H     -3.688388     -0.539317      5.321483
H     -3.946825      1.137094      4.849392
H     -3.570867     -1.920131      1.831173
H     -3.464745     -2.347651      3.535904
H     -4.101341      1.757792      2.380885
H     -3.947695      0.510765      1.147247
H     -5.006391     -0.414613      3.208623
C      1.428383     -9.892083     -0.570369
```

C	0.453087	-8.802844	-0.098202
C	-0.991643	-9.238779	-0.387454
C	-1.163344	-9.461626	-1.897854
C	-0.192178	-10.552542	-2.374975
C	1.250490	-10.113440	-2.080280
C	-1.286174	-10.550404	0.356644
C	1.127642	-11.201798	0.174714
C	-0.488332	-11.860742	-1.625550
C	-0.315013	-11.644009	-0.114512
H	2.457669	-9.579245	-0.363458
H	0.583640	-8.619981	0.975226
H	0.670024	-7.857142	-0.609611
H	-1.685271	-8.460988	-0.049506
H	-2.196837	-9.754090	-2.119695
H	-0.975082	-8.527210	-2.440330
H	-0.315958	-10.709336	-3.452074
H	1.955573	-10.875335	-2.434313
H	1.481698	-9.190742	-2.626310
H	-1.187422	-10.400522	1.438595
H	-2.321579	-10.862197	0.173464
H	1.831059	-11.982803	-0.138707
H	1.269394	-11.062403	1.253247
H	-1.509803	-12.195716	-1.842823
H	0.185499	-12.653769	-1.971995
H	-0.527082	-12.577568	0.418163
C	2.798038	4.514800	2.451337
C	3.183277	5.551900	1.385224
C	2.734100	5.063276	-0.000575
C	3.419736	3.724873	-0.315261
C	3.035317	2.682823	0.746465
C	3.483357	3.177706	2.130408
C	1.210274	4.866441	-0.001601
C	1.273927	4.319923	2.444045
C	1.510708	2.492309	0.743961
C	0.820221	3.827527	1.061239
H	3.119326	4.864945	3.438395
H	2.716819	6.517836	1.613514
H	4.268020	5.713820	1.393445
H	3.009261	5.804456	-0.759012
H	3.122417	3.375127	-1.311321
H	4.508479	3.855309	-0.337164
H	3.525664	1.729664	0.520422
H	3.232366	2.433248	2.895752
H	4.573321	3.298088	2.151999
H	0.706814	5.819388	0.201514
H	0.874588	4.535874	-0.991991
H	0.983634	3.596331	3.215499
H	0.772580	5.263668	2.690536
H	1.179727	2.120517	-0.233301
H	1.224822	1.735230	1.484275
H	-0.266653	3.689876	1.058484
C	-5.628621	-7.087687	-0.764410
C	-7.148849	-7.291707	-0.675711
C	-7.466211	-8.390255	0.350655

C	-6.796873	-9.702706	-0.085128
C	-5.275610	-9.505131	-0.172485
C	-4.963855	-8.403455	-1.197211
C	-6.922067	-7.974342	1.726051
C	-5.089571	-6.674705	0.613929
C	-4.736992	-9.085919	1.204064
C	-5.401113	-7.772003	1.643168
H	-5.404553	-6.304362	-1.496737
H	-7.638548	-6.353790	-0.387203
H	-7.548605	-7.568755	-1.658763
H	-8.550594	-8.533630	0.412661
H	-7.032692	-10.500455	0.629605
H	-7.191023	-10.021836	-1.057577
H	-4.800596	-10.441865	-0.483836
H	-3.879153	-8.266052	-1.283852
H	-5.325181	-8.699717	-2.189507
H	-7.406924	-7.048743	2.059241
H	-7.159434	-8.742337	2.472157
H	-4.007216	-6.505837	0.559254
H	-5.542558	-5.726093	0.926493
H	-4.936179	-9.872908	1.941486
H	-3.648189	-8.961249	1.160137
H	-5.016292	-7.474985	2.624974
C	-6.158065	-6.241799	-7.288015
C	-6.467718	-7.543674	-6.533152
C	-6.220613	-7.343487	-5.029883
C	-4.751452	-6.955628	-4.802770
C	-4.437059	-5.652208	-5.553210
C	-4.689109	-5.856584	-7.055092
C	-7.129461	-6.218988	-4.509990
C	-7.067553	-5.120424	-6.762376
C	-5.350708	-4.532055	-5.032407
C	-6.821339	-4.913748	-5.260004
H	-6.334640	-6.386733	-8.359428
H	-7.508677	-7.841623	-6.707405
H	-5.837354	-8.356979	-6.912796
H	-6.442544	-8.272710	-4.493621
H	-4.557305	-6.829309	-3.730759
H	-4.091439	-7.759006	-5.151801
H	-3.389067	-5.377929	-5.390324
H	-4.449262	-4.938607	-7.605325
H	-4.027697	-6.640250	-7.444169
H	-8.182861	-6.491742	-4.647066
H	-6.976184	-6.079361	-3.433030
H	-6.870483	-4.189567	-7.307964
H	-8.119525	-5.374673	-6.939778
H	-5.166925	-4.362746	-3.964523
H	-5.122135	-3.590458	-5.546329
H	-7.471217	-4.114230	-4.887608
C	-8.689057	-4.344496	5.387463
C	-8.368481	-3.242015	4.366507
C	-6.847379	-3.044365	4.276305
C	-6.303924	-2.640411	5.655408
C	-6.619158	-3.741220	6.679982

C	-8.140818	-3.937381	6.763752
C	-6.188329	-4.360572	3.835802
C	-8.025479	-5.657347	4.943580
C	-5.961140	-5.055814	6.233227
C	-6.503620	-5.465870	4.855615
H	-9.773856	-4.483636	5.450391
H	-8.772448	-3.510511	3.382915
H	-8.852689	-2.303523	4.662695
H	-6.621108	-2.258322	3.547453
H	-5.220897	-2.476730	5.599574
H	-6.751844	-1.691205	5.973577
H	-6.231303	-3.450858	7.662482
H	-8.380683	-4.707526	7.506925
H	-8.621308	-3.011244	7.101951
H	-6.552047	-4.650492	2.842579
H	-5.103219	-4.227028	3.748993
H	-8.263955	-6.458219	5.654023
H	-8.422354	-5.969328	3.969969
H	-4.872043	-4.934860	6.187886
H	-6.161826	-5.845613	6.967331
H	-6.032978	-6.402988	4.538511
C	-0.515009	-5.416607	-2.289505
C	-2.035823	-5.629449	-2.240467
C	-2.731022	-4.318036	-1.843169
C	-2.229984	-3.875097	-0.459979
C	-0.709552	-3.657738	-0.504131
C	-0.019537	-4.970724	-0.905246
C	-2.396915	-3.231418	-2.876815
C	-0.186897	-4.327398	-3.322421
C	-0.380427	-2.573162	-1.541486
C	-0.876808	-3.012710	-2.927361
H	-0.021355	-6.352678	-2.572676
H	-2.400462	-5.968043	-3.217772
H	-2.281558	-6.418673	-1.519570
H	-3.815205	-4.471960	-1.809386
H	-2.734126	-2.949722	-0.156041
H	-2.479683	-4.633589	0.291815
H	-0.354504	-3.342557	0.483113
H	1.068641	-4.834424	-0.921250
H	-0.229736	-5.748587	-0.161119
H	-2.767603	-3.525426	-3.866252
H	-2.903382	-2.294646	-2.614550
H	0.898369	-4.180238	-3.381910
H	-0.518832	-4.641739	-4.319299
H	-0.851669	-1.624731	-1.256480
H	0.701291	-2.393909	-1.568296
H	-0.641774	-2.238021	-3.665511
C	-1.316946	-2.241833	-9.439524
C	-2.274462	-1.140651	-8.958749
C	-2.071795	-0.896479	-7.455333
C	-2.355978	-2.195538	-6.685831
C	-1.398790	-3.299725	-7.160666
C	-1.602882	-3.537479	-8.664915
C	-0.619943	-0.462417	-7.201350

C	0.132912	-1.803811	-9.180691
C	0.051391	-2.859465	-6.907922
C	0.341683	-1.562104	-7.677877
H	-1.463202	-2.414483	-10.511396
H	-2.094934	-0.214712	-9.518337
H	-3.312910	-1.433707	-9.155460
H	-2.755049	-0.110651	-7.114853
H	-2.234058	-2.028868	-5.608698
H	-3.395977	-2.506458	-6.842626
H	-1.603560	-4.225005	-6.611143
H	-0.938834	-4.338034	-9.012936
H	-2.629467	-3.872575	-8.856781
H	-0.409127	0.475838	-7.728673
H	-0.467616	-0.266278	-6.133100
H	0.827802	-2.573246	-9.538595
H	0.356028	-0.889319	-9.743436
H	0.215890	-2.704809	-5.834684
H	0.744728	-3.648273	-7.224200
H	1.375825	-1.249472	-7.496055
C	-8.568294	-0.011459	-6.783726
C	-8.087989	0.404675	-5.384897
C	-6.567353	0.624495	-5.402209
C	-5.869252	-0.679553	-5.817612
C	-6.343774	-1.098791	-7.217602
C	-7.865035	-1.313815	-7.195761
C	-6.224260	1.729223	-6.413472
C	-8.220288	1.095321	-7.791137
C	-6.001733	0.010446	-8.224248
C	-6.700061	1.316226	-7.814957
H	-9.652468	-0.167867	-6.769132
H	-8.597787	1.322966	-5.069334
H	-8.347657	-0.369687	-4.652852
H	-6.227202	0.921646	-4.404068
H	-4.781276	-0.541147	-5.814397
H	-6.089932	-1.472452	-5.092721
H	-5.845226	-2.028929	-7.511439
H	-8.214285	-1.632993	-8.185264
H	-8.121096	-2.118255	-6.495556
H	-6.699803	2.672187	-6.117645
H	-5.142394	1.909054	-6.421151
H	-8.576554	0.819263	-8.791069
H	-8.731597	2.026542	-7.518835
H	-4.916078	0.160822	-8.263740
H	-6.317369	-0.285546	-9.232081
H	-6.454619	2.106008	-8.533464
C	-4.599349	7.256490	2.634816
C	-4.826559	7.009901	1.135432
C	-3.838074	5.948489	0.628230
C	-2.400824	6.441707	0.855160
C	-2.167107	6.687021	2.353861
C	-3.160127	7.746247	2.856752
C	-4.054686	4.639592	1.403253
C	-4.814507	5.944120	3.404452
C	-2.388406	5.376431	3.124650

C	-3.825531	4.880459	2.903448
H	-5.304827	8.013575	2.994304
H	-5.857349	6.679052	0.960092
H	-4.694177	7.944449	0.576819
H	-4.002239	5.773869	-0.440809
H	-1.685176	5.700807	0.478634
H	-2.226246	7.365988	0.291172
H	-1.142066	7.039084	2.513206
H	-2.990687	7.945361	3.921932
H	-2.998763	8.693819	2.328502
H	-5.071308	4.264714	1.233598
H	-3.367336	3.867539	1.036762
H	-4.675217	6.111420	4.479443
H	-5.844765	5.593209	3.269586
H	-1.672342	4.616822	2.788401
H	-2.204651	5.533393	4.194491
H	-3.981285	3.945751	3.453276
C	-2.603533	1.866008	-4.401332
C	-3.564230	2.956207	-3.902221
C	-3.336694	3.201797	-2.402599
C	-3.592231	1.899237	-1.628981
C	-2.631719	0.806026	-2.122149
C	-2.860768	0.566838	-3.622573
C	-1.885333	3.651845	-2.175006
C	-1.154120	2.319992	-4.168850
C	-1.182120	1.262255	-1.895760
C	-0.920465	2.563186	-2.669944
H	-2.767536	1.692326	-5.470466
H	-3.405318	3.884391	-4.464317
H	-4.602797	2.651723	-4.080041
H	-4.022265	3.979789	-2.049035
H	-3.452396	2.066664	-0.554146
H	-4.631417	1.576851	-1.766877
H	-2.816060	-0.121774	-1.569652
H	-2.194373	-0.226090	-3.983336
H	-3.886918	0.220447	-3.795799
H	-1.694684	4.592676	-2.705409
H	-1.715587	3.849084	-1.109588
H	-0.457418	1.558531	-4.540037
H	-0.951562	3.237217	-4.734912
H	-0.999654	1.418145	-0.825609
H	-0.485990	0.481383	-2.225314
H	0.113307	2.887204	-2.506917
C	-9.879963	3.973725	-1.677392
C	-9.154713	2.682338	-2.085945
C	-7.639124	2.930772	-2.133112
C	-7.336031	4.035441	-3.156973
C	-8.056615	5.330510	-2.751432
C	-9.571104	5.075848	-2.702287
C	-7.152995	3.376567	-0.745403
C	-9.388159	4.416339	-0.290609
C	-7.569857	5.770038	-1.361966
C	-7.873443	4.669807	-0.333582
H	-10.960202	3.794511	-1.644598

H	-9.383023	1.880565	-1.373368
H	-9.511258	2.345629	-3.066993
H	-7.124041	2.008635	-2.424040
H	-6.254522	4.208303	-3.214540
H	-7.660441	3.721886	-4.156621
H	-7.839555	6.116450	-3.483028
H	-10.099376	5.998550	-2.433001
H	-9.935239	4.781091	-3.694004
H	-7.344528	2.588121	-0.007498
H	-6.068417	3.538655	-0.761180
H	-9.913763	5.326896	0.021836
H	-9.619705	3.645697	0.454547
H	-6.492537	5.973812	-1.387920
H	-8.062175	6.705390	-1.069213
H	-7.525238	4.984700	0.656290
C	4.037807	0.470258	-2.597673
C	4.395159	1.529000	-3.652163
C	3.946185	1.050201	-5.041453
C	4.655918	-0.270005	-5.378771
C	4.299454	-1.333620	-4.328740
C	4.747154	-0.848561	-2.941214
C	2.426569	0.823225	-5.035064
C	2.517824	0.245319	-2.597522
C	2.778921	-1.554272	-4.323739
C	2.064418	-0.237381	-3.983840
H	4.358908	0.813432	-1.608109
H	3.911311	2.482349	-3.407726
H	5.476526	1.712269	-3.648925
H	4.201439	1.806797	-5.791582
H	4.358711	-0.612184	-6.377489
H	5.741699	-0.117728	-5.406107
H	4.806948	-2.273753	-4.570915
H	4.516177	-1.608048	-2.184382
H	5.834653	-0.706894	-2.925205
H	1.905811	1.763193	-4.815732
H	2.090664	0.499383	-6.027600
H	2.247184	-0.494169	-1.834038
H	1.999660	1.175547	-2.334957
H	2.448624	-1.919409	-5.303737
H	2.513133	-2.326733	-3.591875
H	0.980480	-0.396504	-3.981269
C	3.714656	-5.210185	-2.504644
C	3.409683	-6.528013	-1.776011
C	4.384591	-7.618725	-2.245965
C	4.229082	-7.822049	-3.760865
C	4.536043	-6.507567	-4.494908
C	3.561019	-5.419584	-4.018918
C	5.824802	-7.181611	-1.936914
C	5.156538	-4.779309	-2.194369
C	5.975680	-6.072932	-4.179834
C	6.135673	-5.865245	-2.666059
H	3.018448	-4.434093	-2.168364
H	3.495753	-6.387569	-0.691761
H	2.376104	-6.836548	-1.974687

H	4.165696	-8.557208	-1.724792
H	4.906191	-8.612977	-4.105559
H	3.209946	-8.153638	-3.993814
H	4.424023	-6.654732	-5.574650
H	3.756693	-4.480228	-4.550224
H	2.530010	-5.708405	-4.256788
H	5.955943	-7.052612	-0.855712
H	6.529729	-7.961077	-2.250514
H	5.380277	-3.828173	-2.692646
H	5.274795	-4.608060	-1.117592
H	6.683635	-6.832840	-4.532194
H	6.214363	-5.145508	-4.714511
H	7.162780	-5.555940	-2.443281
C	-8.056968	-0.841120	-0.200032
C	-8.311105	-1.060161	-1.699371
C	-7.380261	-2.162395	-2.228107
C	-5.918628	-1.741499	-2.011488
C	-5.658061	-1.524037	-0.512915
C	-6.593584	-0.423722	0.011538
C	-7.651919	-3.467680	-1.464417
C	-8.327397	-2.149886	0.558221
C	-5.934525	-2.830742	0.246575
C	-7.396107	-3.254456	0.035653
H	-8.721356	-0.054872	0.174821
H	-9.358406	-1.339031	-1.867078
H	-8.139447	-0.127061	-2.249654
H	-7.563564	-2.317271	-3.297079
H	-5.243476	-2.511886	-2.403318
H	-5.705376	-0.820629	-2.567703
H	-4.615641	-1.223528	-0.360919
H	-6.403869	-0.244594	1.076848
H	-6.392041	0.520660	-0.508561
H	-8.687137	-3.791271	-1.627048
H	-7.006432	-4.267927	-1.846234
H	-8.169231	-2.001187	1.633335
H	-9.374722	-2.449260	0.430769
H	-5.259450	-3.620273	-0.105181
H	-5.732500	-2.694455	1.315957
H	-7.591206	-4.186495	0.577400
C	-2.680114	-2.341258	7.652328
C	-1.236921	-1.848679	7.839790
C	-0.942526	-1.661419	9.336192
C	-1.917488	-0.628229	9.921356
C	-3.362636	-1.117415	9.739288
C	-3.650727	-1.306459	8.241886
C	-1.126603	-3.002625	10.062878
C	-2.859274	-3.680963	8.383109
C	-3.540547	-2.460228	10.464502
C	-2.569705	-3.498026	9.880820
H	-2.887977	-2.473880	6.584941
H	-0.533593	-2.568957	7.404749
H	-1.091109	-0.901440	7.306471
H	0.087072	-1.310752	9.467515
H	-1.704370	-0.470743	10.985610

H	-1.782980	0.340262	9.424579
H	-4.055943	-0.379062	10.156672
H	-4.686449	-1.636839	8.096647
H	-3.547319	-0.349876	7.715354
H	-0.421751	-3.744806	9.668941
H	-0.900417	-2.886304	11.129716
H	-3.880724	-4.053891	8.239712
H	-2.184723	-4.434390	7.958741
H	-3.356378	-2.335018	11.538384
H	-4.574484	-2.810730	10.359206
H	-2.697605	-4.454549	10.399430
C	2.560505	-1.160196	2.558828
C	2.344822	-1.366031	1.051613
C	3.309779	-2.442665	0.531354
C	4.757208	-1.994943	0.786189
C	4.979368	-1.790546	2.292766
C	4.009871	-0.715872	2.808614
C	3.047787	-3.762400	1.273277
C	2.299935	-2.483319	3.295303
C	4.712696	-3.111746	3.030364
C	3.265246	-3.562355	2.781192
H	1.871837	-0.392189	2.927626
H	1.307515	-1.663978	0.856795
H	2.509679	-0.423005	0.516379
H	3.153867	-2.588170	-0.543265
H	5.456694	-2.746668	0.400851
H	4.964025	-1.063228	0.245829
H	6.011707	-1.470873	2.472021
H	4.171777	-0.545949	3.880004
H	4.203686	0.238669	2.304367
H	2.023359	-4.104872	1.083284
H	3.718008	-4.544701	0.896923
H	2.430711	-2.344574	4.375416
H	1.262116	-2.802255	3.140485
H	5.411593	-3.883101	2.684861
H	4.887694	-2.984452	4.105602
H	3.077131	-4.504691	3.307372
C	-6.832238	2.607456	4.569442
C	-7.053951	1.287312	5.323528
C	-8.503450	0.815806	5.129648
C	-9.463518	1.884912	5.673459
C	-9.248137	3.206838	4.920446
C	-7.796628	3.672032	5.114517
C	-8.773852	0.604229	3.632014
C	-7.106601	2.390823	3.073199
C	-9.516152	2.989191	3.423271
C	-8.556328	1.922881	2.873756
H	-5.798446	2.941923	4.709138
H	-6.357214	0.523243	4.958167
H	-6.842773	1.424501	6.391018
H	-8.659104	-0.125799	5.667643
H	-10.501912	1.551024	5.558945
H	-9.294534	2.031935	6.747090
H	-9.933215	3.967658	5.310147

H	-7.634822	4.625885	4.597847
H	-7.598178	3.851118	6.178249
H	-8.108774	-0.172089	3.234726
H	-9.800463	0.248742	3.481911
H	-6.931774	3.321736	2.520177
H	-6.411568	1.646038	2.667019
H	-10.555557	2.675092	3.269009
H	-9.385459	3.931090	2.876726
H	-8.749240	1.768670	1.806398
C	0.252963	-5.784803	4.412736
C	-0.731980	-4.726849	4.933510
C	-2.174580	-5.191211	4.680081
C	-2.387151	-5.391899	3.171776
C	-1.406447	-6.451539	2.646143
C	0.034186	-5.984153	2.905131
C	-2.416401	-6.521974	5.409041
C	0.004922	-7.113818	5.142900
C	-1.649807	-7.779008	3.380538
C	-1.435460	-7.584405	4.889323
H	1.280706	-5.451727	4.594164
H	-0.572944	-4.559547	6.005634
H	-0.552285	-3.767675	4.432863
H	-2.875075	-4.435709	5.052654
H	-3.420015	-5.704449	2.975918
H	-2.236801	-4.444137	2.640741
H	-1.559420	-6.592563	1.570616
H	0.745276	-6.723222	2.516423
H	0.227609	-5.047043	2.369041
H	-2.288133	-6.388526	6.490047
H	-3.449558	-6.854557	5.251532
H	0.716021	-7.872728	4.794488
H	0.176293	-6.989792	6.219008
H	-2.669552	-8.133849	3.188121
H	-0.969007	-8.550054	2.999795
H	-1.609918	-8.531696	5.411295
C	-1.487399	-8.426923	-6.948653
C	-1.128765	-7.289145	-7.916861
C	0.393332	-7.079401	-7.930400
C	0.869988	-6.718303	-6.515178
C	0.516512	-7.854499	-5.543057
C	-1.005859	-8.062542	-5.535917
C	1.081811	-8.376202	-8.383275
C	-0.794268	-9.720229	-7.404583
C	1.204234	-9.149486	-6.002168
C	0.728529	-9.516726	-7.416058
H	-2.572858	-8.574642	-6.940337
H	-1.485221	-7.527186	-8.926320
H	-1.633121	-6.363980	-7.612481
H	0.646783	-6.268175	-8.621748
H	1.953165	-6.546304	-6.515685
H	0.400793	-5.782943	-6.186829
H	0.856758	-7.594666	-4.534708
H	-1.273666	-8.858439	-4.830372
H	-1.508332	-7.151103	-5.189829

```
H      0.766163     -8.635091     -9.401203
H      2.168715     -8.233279     -8.415604
H     -1.058956    -10.545738     -6.732666
H     -1.143767    -10.002020     -8.405185
H      2.293317     -9.020502     -5.993328
H      0.976128     -9.964486     -5.304524
H      1.220282    -10.439903     -7.741900
```

Adamantane$_{21}$

```
         546
E=-874.0944
C     -4.198021      3.242865     59.690065
C     -4.098097      2.227476     60.838797
C     -4.514930      0.835548     60.339055
C     -5.962587      0.890850     59.827512
C     -6.067623      1.902879     58.676134
C     -5.646436      3.292000     59.180008
C     -3.586488      0.407444     59.192097
C     -3.269938      2.808509     58.545101
C     -5.134921      1.472425     57.533544
C     -3.685224      1.418513     58.039229
H     -3.900676      4.234446     60.048480
H     -3.072663      2.197051     61.226470
H     -4.742490      2.536861     61.670657
H     -4.442386      0.113139     61.159511
H     -6.278961     -0.102249     59.486165
H     -6.639300      1.176401     60.641991
H     -7.100591      1.941034     58.313409
H     -5.735882      4.028464     58.372180
H     -6.317800      3.620317     59.982789
H     -2.551301      0.343691     59.548692
H     -3.861958     -0.593765     58.839210
H     -3.316595      3.536813     57.726326
H     -2.229500      2.787576     58.891233
H     -5.436869      0.489730     57.151501
H     -5.215794      2.176278     56.696278
H     -3.021805      1.110936     57.223435
C     -2.154950     -1.820839     55.951386
C     -3.627398     -1.778790     55.514680
C     -4.059663     -3.165020     55.012219
C     -3.181684     -3.570238     53.818319
C     -1.707899     -3.616890     54.250395
C     -1.281750     -2.229616     54.755184
C     -3.887420     -4.192309     56.141732
C     -1.988020     -2.851378     57.078803
C     -1.541599     -4.642648     55.382163
C     -2.415427     -4.240242     56.579969
H     -1.848772     -0.831656     56.308992
H     -4.260618     -1.468188     56.354496
H     -3.763010     -1.032552     54.722411
H     -5.109891     -3.132851     54.701937
H     -3.493801     -4.550266     53.437549
```

H	-3.310104	-2.855440	52.996476
H	-1.083814	-3.905439	53.397573
H	-0.225004	-2.244094	55.048519
H	-1.376123	-1.491072	53.949812
H	-4.524911	-3.926503	56.993664
H	-4.211142	-5.183298	55.801176
H	-0.943960	-2.876322	57.413935
H	-2.592226	-2.560956	57.946760
H	-1.824474	-5.641653	55.028911
H	-0.489353	-4.700365	55.686158
H	-2.295980	-4.972967	57.385635
C	4.444579	4.744542	60.406391
C	4.568884	3.731752	61.555037
C	6.015903	3.707272	62.071263
C	6.957869	3.303301	60.926730
C	6.839835	4.314857	59.776196
C	5.390626	4.338089	59.266098
C	6.400039	5.106783	62.575840
C	4.832412	6.141375	60.915695
C	7.221390	5.712938	60.286607
C	6.280203	6.123006	61.429523
H	3.412391	4.760260	60.039714
H	3.883249	3.998595	62.368321
H	4.276334	2.733102	61.208533
H	6.102079	2.984965	62.890491
H	7.992632	3.262700	61.287942
H	6.707134	2.297013	60.569840
H	7.511807	4.024578	58.961187
H	5.296939	5.042461	58.430605
H	5.112201	3.350203	58.879394
H	5.748183	5.399773	63.407751
H	7.425337	5.097744	62.965091
H	4.728050	6.877898	60.109692
H	4.152283	6.451720	61.718110
H	8.260896	5.714950	60.636237
H	7.160781	6.441555	59.469044
H	6.554079	7.119749	61.792489
C	2.978915	1.061148	58.856597
C	1.842586	1.559110	59.762895
C	0.487888	1.306536	59.083226
C	0.442562	2.057657	57.743744
C	1.575336	1.561195	56.832012
C	2.927237	1.812742	57.517600
C	0.319530	-0.199166	58.827671
C	2.804101	-0.444060	58.602169
C	1.404589	0.054767	56.582500
C	1.452270	-0.701429	57.918966
H	3.943712	1.242342	59.342853
H	1.880058	1.044985	60.730884
H	1.966913	2.629476	59.967378
H	-0.320808	1.661802	59.731365
H	-0.528097	1.901389	57.257679
H	0.541946	3.136647	57.913323
H	1.541415	2.098412	55.877918

H	3.746224	1.480601	56.868055
H	3.070864	2.887473	57.682842
H	0.329275	-0.746372	59.778095
H	-0.652960	-0.394638	58.359819
H	3.621347	-0.816428	57.972528
H	2.857973	-0.994645	59.549119
H	0.451054	-0.136827	56.075666
H	2.196211	-0.308394	55.915857
H	1.329836	-1.775173	57.739148
C	2.230472	-5.656550	59.088086
C	0.756143	-5.629043	58.656600
C	-0.128131	-5.247363	59.853819
C	0.059233	-6.280966	60.974983
C	1.531826	-6.310426	61.412600
C	2.411902	-6.688989	60.211309
C	0.283107	-3.859184	60.368241
C	2.635456	-4.267039	59.604026
C	1.939090	-4.919477	61.922630
C	1.756442	-3.882424	60.803968
H	2.859066	-5.929052	58.233263
H	0.615927	-4.910126	57.840370
H	0.460029	-6.611175	58.268418
H	-1.178737	-5.227815	59.543769
H	-0.582423	-6.030445	61.828432
H	-0.249585	-7.274201	60.627175
H	1.663144	-7.048333	62.211535
H	3.464850	-6.733008	60.515164
H	2.145052	-7.689663	59.850170
H	0.134802	-3.107177	59.583813
H	-0.354177	-3.566821	61.211584
H	3.692593	-4.267849	59.896357
H	2.528442	-3.523054	58.805407
H	1.330827	-4.645053	62.792990
H	2.983462	-4.932307	62.257424
H	2.046766	-2.891319	61.169581
C	-4.949161	-3.413103	60.032915
C	-3.596807	-3.168423	60.719831
C	-3.550401	-3.922019	62.058073
C	-4.684696	-3.419928	62.964565
C	-6.040077	-3.665070	62.283569
C	-6.079975	-2.912466	60.944561
C	-3.732360	-5.426023	61.801842
C	-5.127025	-4.918484	59.781637
C	-6.215608	-5.169639	62.026284
C	-5.085217	-5.676200	61.117516
H	-4.980243	-2.874855	59.079273
H	-2.778626	-3.502840	60.070618
H	-3.449665	-2.094589	60.887704
H	-2.585178	-3.746613	62.545788
H	-4.650002	-3.936531	63.931402
H	-4.556005	-2.350664	63.171852
H	-6.847179	-3.305872	62.931343
H	-7.050772	-3.063142	60.456898
H	-5.976399	-1.834116	61.116058

H	-2.917147	-5.802623	61.172210
H	-3.681809	-5.977467	62.748514
H	-6.080553	-5.104694	59.272610
H	-4.336092	-5.285081	59.116213
H	-6.208242	-5.717095	62.976576
H	-7.189084	-5.360383	61.558390
H	-5.211711	-6.749125	60.935633
C	5.526695	-0.534561	61.704199
C	4.386376	-0.051743	62.613674
C	4.429012	-0.812470	63.948002
C	4.270615	-2.317208	63.681316
C	5.410553	-2.806143	62.774458
C	5.365666	-2.040041	61.443286
C	5.778818	-0.557511	64.636342
C	6.873575	-0.279775	62.398422
C	6.757930	-2.545732	63.465469
C	6.922632	-1.041655	63.731684
H	5.494353	0.008804	60.753505
H	4.477464	1.026556	62.791731
H	3.419669	-0.211136	62.120699
H	3.615519	-0.466250	64.595031
H	4.279370	-2.869928	64.628564
H	3.301663	-2.515884	63.207485
H	5.295291	-3.878970	62.585239
H	6.162921	-2.393860	60.778333
H	4.416243	-2.234105	60.929560
H	5.896390	0.511451	64.851589
H	5.814104	-1.080185	65.599877
H	7.697637	-0.601615	61.750087
H	7.009546	0.794276	62.573591
H	6.810714	-3.102724	64.408757
H	7.579820	-2.909108	62.836645
H	7.883523	-0.857982	64.224840
C	-0.577601	4.300490	61.746653
C	-1.701011	4.840439	62.644978
C	-1.483642	6.339348	62.903778
C	-0.128311	6.544790	63.597549
C	0.999416	6.009184	62.701855
C	0.775489	4.511214	62.443231
C	-1.488771	7.093028	61.564850
C	-0.585608	5.059333	60.410599
C	0.987770	6.763299	61.363232
C	-0.364688	6.558507	60.663830
H	-0.734324	3.231829	61.563592
H	-2.675305	4.677839	62.168486
H	-1.717640	4.293301	63.595407
H	-2.286015	6.722306	63.543957
H	0.029814	7.609887	63.805928
H	-0.117967	6.027764	64.564726
H	1.964615	6.156194	63.198672
H	1.585231	4.111584	61.820671
H	0.802234	3.958043	63.389944
H	-2.458832	6.971980	61.067691
H	-1.353607	8.167576	61.737796

H	0.199463	4.668891	59.751648
H	-1.540002	4.901659	59.893850
H	1.165897	7.832204	61.532142
H	1.801679	6.404037	60.721791
H	-0.371332	7.097292	59.709934
C	8.371653	2.689971	65.311751
C	9.322942	2.315659	64.164788
C	10.773149	2.314382	64.672578
C	11.129368	3.713126	65.198955
C	10.182740	4.091357	66.348626
C	8.734007	4.087699	65.836666
C	10.912911	1.292003	65.810998
C	8.517522	1.666175	66.448198
C	10.323567	3.064266	67.482640
C	9.965403	1.663909	66.962096
H	7.338453	2.690518	64.947599
H	9.062372	1.327595	63.766586
H	9.214388	3.029067	63.338823
H	11.449558	2.046711	63.853366
H	12.169534	3.730544	65.546242
H	11.052765	4.450570	64.390770
H	10.438182	5.089072	66.721939
H	8.049006	4.375689	66.643389
H	8.614821	4.832083	65.040007
H	10.681342	0.284818	65.443860
H	11.949144	1.267799	66.169422
H	7.828102	1.910725	67.265445
H	8.243352	0.666050	66.091353
H	11.349427	3.070445	67.870443
H	9.667224	3.334484	68.318798
H	10.066896	0.932925	67.771794
C	-6.904354	1.619423	63.785946
C	-8.036761	2.146986	64.680326
C	-8.018776	1.411035	66.029031
C	-8.212499	-0.094235	65.790114
C	-7.080633	-0.627854	64.898466
C	-7.100752	0.113584	63.552803
C	-6.666348	1.646292	66.719235
C	-5.554882	1.854691	64.482021
C	-5.730512	-0.387038	65.591225
C	-5.530501	1.117410	65.829714
H	-6.919098	2.145183	62.824973
H	-7.920292	3.225962	64.838372
H	-9.004782	2.001693	64.185576
H	-8.826574	1.789141	66.665258
H	-8.221545	-0.628840	66.747701
H	-9.183808	-0.278374	65.315238
H	-7.221084	-1.700987	64.729034
H	-6.309290	-0.272065	62.898718
H	-8.052242	-0.067178	63.038050
H	-6.523801	2.716002	66.914775
H	-6.648215	1.141387	67.692670
H	-4.735919	1.500671	63.844080
H	-5.393633	2.928277	64.637487

H	-5.695629	-0.927055	66.545155
H	-4.914821	-0.782148	64.973568
H	-4.567705	1.287117	66.324156
C	-2.109173	-0.209351	63.596837
C	-1.180113	-0.593678	62.435061
C	0.276238	-0.626155	62.923876
C	0.665943	0.761821	63.455008
C	-0.258347	1.149957	64.619458
C	-1.713374	1.177502	64.126422
C	0.411082	-1.658967	64.053437
C	-1.968278	-1.243682	64.724336
C	-0.122587	0.112445	65.744568
C	-0.514192	-1.277147	65.219282
H	-3.146788	-0.186558	63.246202
H	-1.464674	-1.573780	62.033619
H	-1.285639	0.127442	61.615425
H	0.936766	-0.900921	62.094121
H	1.710648	0.756975	63.788812
H	0.593049	1.506238	62.652896
H	0.020951	2.139981	64.996146
H	-2.382262	1.472834	64.943938
H	-1.828557	1.929589	63.336436
H	0.155548	-2.658957	63.682548
H	1.451202	-1.705430	64.398145
H	-2.642280	-0.991771	65.552139
H	-2.266133	-2.235874	64.364342
H	0.908124	0.096351	66.118993
H	-0.762787	0.389256	66.591022
H	-0.416271	-2.015591	66.022626
C	-6.880811	-3.778403	67.744097
C	-6.769944	-4.768761	68.913491
C	-7.127802	-6.183215	68.431606
C	-8.565456	-6.187824	67.889778
C	-8.681258	-5.201050	66.717697
C	-8.319039	-3.789173	67.203854
C	-6.161249	-6.599234	67.312011
C	-5.914443	-4.200647	66.626604
C	-7.710459	-5.619190	65.602599
C	-6.270693	-5.613226	66.138587
H	-6.625529	-2.770717	68.089798
H	-5.752430	-4.756298	69.322442
H	-7.441963	-4.467126	69.726116
H	-7.047540	-6.887707	69.266792
H	-8.840056	-7.197383	67.560779
H	-9.268579	-5.911448	68.684819
H	-9.707091	-5.205601	66.333386
H	-8.416940	-3.071001	66.380684
H	-9.018052	-3.470090	67.986507
H	-5.132186	-6.620293	67.690720
H	-6.394458	-7.615675	66.972461
H	-5.969077	-3.489327	65.793515
H	-4.881393	-4.178924	66.994157
H	-7.970153	-6.618583	65.233018
H	-7.798048	-4.933833	64.750790

H	-5.580063	-5.912099	65.342375
C	3.587784	4.459100	65.526903
C	2.449838	4.965256	66.426609
C	2.461175	4.199147	67.758488
C	2.270484	2.699443	67.484679
C	3.407880	2.187171	66.587564
C	3.394385	2.958695	65.258838
C	3.809488	4.420167	68.461334
C	4.933108	4.679996	66.235602
C	4.753865	2.413689	67.293077
C	4.950849	3.912588	67.566476
H	3.577774	5.006345	64.577939
H	2.564208	6.040517	66.609545
H	1.484740	4.830164	65.923330
H	1.649434	4.562022	68.398554
H	2.256732	2.143407	68.429926
H	1.302032	2.525064	67.000376
H	3.269585	1.117984	66.393244
H	4.189899	2.588665	64.600657
H	2.445970	2.788609	64.734855
H	3.949738	5.485347	68.681691
H	3.822793	3.893495	69.423245
H	5.755996	4.341252	65.594427
H	5.092277	5.749972	66.416087
H	4.784069	1.852369	68.234791
H	5.573416	2.033405	66.671236
H	5.910696	4.072089	68.069967
C	4.195316	-2.741699	67.774296
C	2.733288	-2.713808	67.302831
C	1.815973	-2.340801	68.477715
C	1.974370	-3.380946	69.597294
C	3.434498	-3.410830	70.074828
C	4.347723	-3.780703	68.895777
C	2.210644	-0.955164	69.011601
C	4.583691	-1.354749	68.309536
C	3.825312	-2.022403	70.604231
C	3.671555	-0.978815	69.487303
H	4.847502	-3.008013	66.935345
H	2.614219	-1.990133	66.487448
H	2.449560	-3.694001	66.900747
H	0.774203	-2.320964	68.139176
H	1.309202	-3.136625	70.434381
H	1.676858	-4.372509	69.235153
H	3.545179	-4.153404	70.872565
H	5.392045	-3.824969	69.228039
H	4.092539	-4.779558	68.521414
H	2.082563	-0.198606	68.227986
H	1.550051	-0.668933	69.838959
H	5.632442	-1.355730	68.630655
H	4.497287	-0.606063	67.512816
H	3.193022	-1.754231	71.459281
H	4.860163	-2.035683	70.967374
H	3.950097	0.010480	69.866723
C	8.362112	-2.956474	68.764555

C	8.315504	-2.185751	67.436249
C	9.475805	-2.634727	66.534542
C	10.809515	-2.354540	67.243880
C	10.862774	-3.125569	68.571880
C	9.698697	-2.675642	69.468225
C	9.353037	-4.141391	66.259441
C	8.241747	-4.462267	68.483400
C	10.736010	-4.630960	68.291764
C	9.402215	-4.916938	67.585044
H	7.534309	-2.634767	69.405926
H	7.357286	-2.361110	66.932462
H	8.381682	-1.107387	67.625202
H	9.440283	-2.084376	65.587896
H	11.646957	-2.652549	66.601433
H	10.919174	-1.279082	67.428866
H	11.814177	-2.923899	69.076073
H	9.736683	-3.204620	70.428280
H	9.788861	-1.605906	69.692979
H	8.413856	-4.353540	65.734306
H	10.165536	-4.470641	65.600446
H	8.253069	-5.022909	69.426028
H	7.282661	-4.679025	67.997675
H	11.572315	-4.969213	67.668069
H	10.793039	-5.194630	69.230907
H	9.313846	-5.990572	67.385671
C	-2.532650	3.734149	69.025429
C	-1.583433	3.366456	67.874615
C	-1.682266	4.422266	66.762658
C	-3.125824	4.477013	66.239597
C	-4.079315	4.847486	67.386119
C	-3.974065	3.790814	68.496665
C	-1.294614	5.796615	67.329829
C	-2.142267	5.110140	69.586853
C	-3.685371	6.220515	67.952097
C	-2.243271	6.170255	68.479419
H	-2.460817	2.980144	69.816899
H	-0.552343	3.302403	68.242693
H	-1.840346	2.376907	67.477449
H	-1.004293	4.158415	65.943499
H	-3.205045	5.213594	65.430877
H	-3.409181	3.506949	65.813521
H	-5.107876	4.885391	67.011053
H	-4.664972	4.033005	69.313338
H	-4.272837	2.808599	68.110702
H	-0.258340	5.777901	67.688467
H	-1.342658	6.556288	66.540259
H	-2.800158	5.375399	70.423403
H	-1.120944	5.078219	69.985147
H	-3.774760	6.987921	67.173691
H	-4.371562	6.506058	68.758678
H	-1.963993	7.150131	68.882029
C	1.691647	4.043640	73.343775
C	0.246217	4.101950	72.826168
C	-0.151118	2.737859	72.241076

C	0.794907	2.385750	71.082843
C	2.241953	2.323041	71.595710
C	2.633046	3.688066	72.182821
C	-0.039084	1.663299	73.333578
C	1.798508	2.965914	74.433745
C	2.347919	1.249905	72.690188
C	1.405828	1.599043	73.852434
H	1.972957	5.017062	73.760217
H	-0.434947	4.375144	73.641099
H	0.152757	4.881297	72.060115
H	-1.182151	2.781574	71.873182
H	0.508946	1.422460	70.643348
H	0.710435	3.134968	70.286355
H	2.914645	2.072423	70.768229
H	3.671476	3.662765	72.534940
H	2.581532	4.460357	71.405717
H	-0.725017	1.891212	74.158434
H	-0.338870	0.687312	72.933065
H	2.821944	2.928341	74.826383
H	1.144795	3.217787	75.277533
H	2.089667	0.266521	72.278994
H	3.381429	1.180940	73.050834
H	1.482333	0.832596	74.631507
C	7.540844	2.320834	70.401133
C	6.372224	2.776110	71.288622
C	6.389666	1.994409	72.611343
C	6.255534	0.492511	72.316797
C	7.423777	0.031072	71.431818
C	7.403942	0.818070	70.112278
C	7.718465	2.253242	73.338042
C	8.866570	2.579367	71.133634
C	8.750017	0.295285	72.161211
C	8.890471	1.796601	72.455437
H	7.526437	2.879121	69.458680
H	6.446265	3.852336	71.486074
H	5.420294	2.613939	70.768543
H	5.556008	2.321009	73.242717
H	6.246559	-0.075338	73.255053
H	5.301326	0.290728	71.815173
H	7.325792	-1.039772	71.222676
H	8.221913	0.483982	69.462452
H	6.470219	0.621763	69.571350
H	7.818140	3.319826	73.573198
H	7.735248	1.715468	74.293736
H	9.710543	2.277280	70.501614
H	8.985599	3.651893	71.329284
H	8.785204	-0.276278	73.096569
H	9.591803	-0.048668	71.547977
H	9.836302	1.982980	72.975933
C	-1.606311	-1.056104	70.686096
C	-1.625314	-0.276141	69.362510
C	-2.970647	-0.491693	68.652179
C	-3.161998	-1.989391	68.368044
C	-3.146897	-2.774078	69.689007

C	-1.801277	-2.552374	70.396891
C	-4.109206	0.003544	69.557283
C	-2.748244	-0.558870	71.585926
C	-4.284665	-2.272797	70.591746
C	-4.096246	-0.775865	70.881468
H	-0.646374	-0.901272	71.190731
H	-1.466606	0.792229	69.552586
H	-0.802421	-0.609248	68.718430
H	-2.982188	0.065725	67.709079
H	-4.110563	-2.154271	67.842784
H	-2.366146	-2.351974	67.706223
H	-3.282922	-3.841556	69.483978
H	-1.769701	-3.123516	71.332715
H	-0.981277	-2.925299	69.771186
H	-3.996614	1.077024	69.751701
H	-5.074310	-0.127311	69.053017
H	-2.732990	-1.094068	72.543176
H	-2.610650	0.504641	71.815644
H	-5.253061	-2.442956	70.105873
H	-4.297508	-2.839429	71.530749
H	-4.908599	-0.420139	71.524734
C	0.354962	-6.059258	66.899344
C	0.325275	-5.298542	65.564798
C	-1.015176	-5.546269	64.855731
C	-1.181398	-7.050791	64.592612
C	-1.155529	-7.816332	65.924597
C	0.185112	-7.562484	66.631130
C	-2.163195	-5.057086	65.752136
C	-0.796455	-5.568209	67.790442
C	-2.302853	-7.321127	66.818551
C	-2.139560	-5.817340	67.087264
H	1.311403	-5.881499	67.403063
H	0.466074	-4.225117	65.739915
H	1.154581	-5.627136	64.926653
H	-1.034357	-5.002532	63.904801
H	-2.126268	-7.238700	64.068428
H	-0.378615	-7.409552	63.937108
H	-1.273639	-8.888703	65.734547
H	0.224563	-8.119714	67.575007
H	1.012149	-7.930674	66.011918
H	-2.068597	-3.979250	65.931458
H	-3.125196	-5.210953	65.248438
H	-0.773949	-6.089470	68.755210
H	-0.676750	-4.499428	68.005237
H	-3.267529	-7.514082	66.333798
H	-2.307893	-7.874536	67.765482
H	-2.958698	-5.465969	67.724289
C	5.461739	-8.399844	66.279136
C	5.879957	-7.004222	66.766904
C	5.689166	-5.980614	65.636957
C	6.554378	-6.384108	64.433193
C	6.138132	-7.778256	63.939233
C	6.327387	-8.797207	65.073635
C	4.211701	-5.956812	65.215860

```
C      3.984674     -8.369588     65.856867
C      4.659420     -7.750126     63.522876
C      3.789412     -7.350042     64.724235
H      5.598734     -9.128034     67.086033
H      5.283268     -6.716202     67.640825
H      6.928253     -7.017518     67.089164
H      5.987402     -4.986259     65.987100
H      6.442203     -5.649625     63.626466
H      7.614340     -6.385991     64.714853
H      6.756608     -8.064063     63.081415
H      6.052299     -9.801007     64.727690
H      7.383435     -8.842163     65.366417
H      3.583621     -5.649944     66.060954
H      4.058885     -5.215408     64.422261
H      3.667778     -9.365991     65.525575
H      3.353484     -8.105357     66.713919
H      4.513848     -7.039983     62.699879
H      4.354952     -8.735194     63.148834
H      2.735596     -7.329998     64.425371
```

Adamantane$_{22}$

```
         572
E=-930.0239
C     -1.618582     -5.899778      9.762498
C     -1.430451     -5.900599      8.237641
C      0.067793     -5.858213      7.899382
C      0.687582     -4.583514      8.492217
C      0.504947     -4.579001     10.017841
C     -0.994396     -4.625067     10.350699
C      0.761043     -7.089059      8.503710
C     -0.921241     -7.130888     10.361689
C      1.196634     -5.813303     10.616843
C      0.578241     -7.091087     10.029388
H     -2.687276     -5.929792     10.001456
H     -1.889818     -6.796078      7.801943
H     -1.940280     -5.035819      7.795847
H      0.199576     -5.859441      6.811761
H      1.753745     -4.531552      8.240313
H      0.214926     -3.695982      8.054338
H      0.947048     -3.669136     10.438248
H     -1.139512     -4.602289     11.437616
H     -1.496845     -3.738043      9.946084
H      0.342187     -8.006884      8.073603
H      1.828615     -7.080333      8.252616
H     -1.065736     -7.153021     11.448717
H     -1.370556     -8.048831      9.963876
H      2.271826     -5.782895     10.402706
H      1.090909     -5.811236     11.708532
H      1.072977     -7.969994     10.457181
C     -2.045863    -10.942889      4.032041
C     -0.660909    -10.726560      3.403004
C     -0.181518     -9.293857      3.683182
C     -1.181015     -8.295445      3.079340
```

C	-2.567533	-8.505376	3.707355
C	-3.040659	-9.940215	3.427470
C	-0.096901	-9.073200	5.201385
C	-1.956016	-10.718338	5.549424
C	-2.476875	-8.287219	5.225552
C	-1.480657	-9.285499	5.834839
H	-2.385837	-11.964698	3.830959
H	0.055255	-11.449993	3.811031
H	-0.707998	-10.902557	2.321450
H	0.806279	-9.142038	3.234301
H	-0.839330	-7.268267	3.255442
H	-1.236583	-8.428883	1.992159
H	-3.278547	-7.792930	3.275003
H	-4.038902	-10.097281	3.853857
H	-3.129570	-10.102719	2.346422
H	0.629252	-9.766215	5.643275
H	0.262965	-8.059325	5.414427
H	-2.934833	-10.889997	6.013587
H	-1.262599	-11.440888	5.996473
H	-2.158359	-7.259809	5.439545
H	-3.465253	-8.414345	5.683716
H	-1.416940	-9.128112	6.917160
C	3.367344	7.541288	2.609584
C	2.959527	6.094918	2.289766
C	1.611922	5.776192	2.955595
C	1.742459	5.948150	4.476726
C	2.147259	7.394155	4.802476
C	3.493295	7.708887	4.131598
C	0.541008	6.741744	2.424896
C	2.291907	8.502086	2.079254
C	1.075297	8.355709	4.266710
C	0.942990	8.189839	2.745123
H	4.328317	7.766361	2.134210
H	2.885767	5.955596	1.204464
H	3.729150	5.399951	2.647052
H	1.322857	4.744729	2.725788
H	0.791621	5.703903	4.965713
H	2.490469	5.250196	4.871942
H	2.240258	7.514278	5.887359
H	3.803729	8.732816	4.372773
H	4.272475	7.042376	4.520982
H	0.422477	6.615135	1.341957
H	-0.430589	6.511774	2.878709
H	2.581400	9.540142	2.283291
H	2.205154	8.405870	0.990229
H	0.112756	8.154162	4.752210
H	1.342553	9.391051	4.510855
H	0.177946	8.875755	2.365022
C	5.562154	4.850391	8.190002
C	5.149410	3.406149	7.866906
C	3.791998	3.096395	8.516859
C	3.905317	3.269345	10.039256
C	4.314972	4.713247	10.368248
C	5.670851	5.019011	9.713232

C	2.733424	4.067807	7.972276
C	4.499024	5.817077	7.645739
C	3.255387	5.680662	9.818591
C	3.140340	5.513809	8.295709
H	6.530114	5.069066	7.725950
H	5.087831	3.266001	6.780951
H	5.910447	2.706942	8.234172
H	3.499440	2.066436	8.284735
H	2.947212	3.031451	10.517062
H	4.644270	2.567327	10.444194
H	4.395676	4.834079	11.454036
H	5.984595	6.041319	9.956987
H	6.441233	4.348240	10.112677
H	2.627127	3.940646	6.888132
H	1.755070	3.844274	8.414645
H	4.792360	6.853595	7.852092
H	4.424760	5.720110	6.555858
H	2.285881	5.485534	10.292722
H	3.525990	6.714648	10.064785
H	2.384100	6.203910	7.905695
C	10.211352	-1.091844	3.551232
C	9.720047	-2.509348	3.219745
C	8.344660	-2.745698	3.862708
C	8.460858	-2.586066	5.386343
C	8.949035	-1.168754	5.723712
C	10.322691	-0.936280	5.075661
C	7.344323	-1.714304	3.318333
C	9.206202	-0.064916	3.007137
C	7.947487	-0.141273	5.174240
C	7.829906	-0.294718	3.650142
H	11.192010	-0.925507	3.092155
H	9.655448	-2.641044	2.132908
H	10.439275	-3.251569	3.586858
H	7.996081	-3.756681	3.624607
H	7.488908	-2.772202	5.859125
H	9.157716	-3.330027	5.791023
H	9.031652	-1.057410	6.810370
H	10.691945	0.065830	5.325444
H	11.052696	-1.650774	5.475123
H	7.235793	-1.830545	2.233185
H	6.353111	-1.884815	3.755663
H	9.556035	0.952687	3.219502
H	9.131365	-0.152774	1.916523
H	6.966556	-0.284057	5.643500
H	8.274309	0.874896	5.426340
H	7.115058	0.438244	3.260259
C	8.655205	6.346742	4.251065
C	7.176125	6.253192	4.656227
C	6.564166	4.961376	4.092605
C	6.676897	4.971054	2.560402
C	8.154710	5.061400	2.149535
C	8.762721	6.352754	2.718459
C	7.327845	3.750627	4.650883
C	9.413321	5.132495	4.809463

C	8.914539	3.850701	2.713097
C	8.807446	3.838103	4.245617
H	9.089458	7.268405	4.653646
H	7.083208	6.268555	5.748867
H	6.627547	7.124849	4.279096
H	5.509815	4.896484	4.383156
H	6.225131	4.062376	2.144389
H	6.119128	5.820068	2.146972
H	8.232625	5.068705	1.056866
H	9.813279	6.439428	2.415506
H	8.242164	7.226330	2.307526
H	7.238467	3.719523	5.743531
H	6.887864	2.820733	4.270688
H	10.475587	5.197913	4.544306
H	9.360261	5.126770	5.904867
H	8.502472	2.922264	2.299547
H	9.967644	3.892691	2.409499
H	9.349280	2.973985	4.645576
C	3.533089	-1.833069	9.678956
C	3.729402	-1.826992	8.155142
C	5.229188	-1.775187	7.825125
C	5.838847	-0.499446	8.426164
C	5.648005	-0.501804	9.950788
C	4.147169	-0.557274	10.275385
C	5.925866	-3.004582	8.428464
C	4.233888	-3.062686	10.277179
C	6.343167	-1.734642	10.548780
C	5.734893	-3.013482	9.953116
H	2.463307	-1.869793	9.912036
H	3.277256	-2.723259	7.713552
H	3.217257	-0.963288	7.713920
H	5.366810	-1.771508	6.738233
H	6.906047	-0.440738	8.180208
H	5.363728	0.387195	7.989145
H	6.082889	0.408814	10.377049
H	3.996100	-0.539474	11.361583
H	3.642084	0.328568	9.871466
H	5.514321	-3.922997	7.992597
H	6.994707	-2.989105	8.183161
H	4.083683	-3.089793	11.363320
H	3.791711	-3.981508	9.873441
H	7.419312	-1.697586	10.340557
H	6.231576	-1.737358	11.639883
H	6.232097	-3.891341	10.380202
C	-4.752432	-3.358462	8.214371
C	-5.772941	-2.395486	7.588413
C	-7.149035	-2.601878	8.240118
C	-7.606372	-4.051302	8.015486
C	-6.590626	-5.018837	8.642418
C	-5.215866	-4.805996	7.989978
C	-7.043678	-2.330071	9.748698
C	-4.653152	-3.084156	9.722962
C	-6.486251	-4.740662	10.149886
C	-6.026169	-3.293031	10.379963

H	-3.772250	-3.209855	7.748249
H	-5.443932	-1.358068	7.723505
H	-5.839015	-2.568932	6.507440
H	-7.874886	-1.913932	7.792728
H	-8.597364	-4.205949	8.459195
H	-7.704852	-4.253188	6.941988
H	-6.918412	-6.051511	8.480588
H	-4.485264	-5.504939	8.415027
H	-5.271861	-5.021636	6.916040
H	-6.737682	-1.291627	9.924299
H	-8.024720	-2.455460	10.222688
H	-3.911976	-3.751997	10.178641
H	-4.304802	-2.058917	9.897021
H	-7.457392	-4.907771	10.631319
H	-5.778657	-5.438876	10.613322
H	-5.952850	-3.096382	11.455232
C	-4.141815	-8.241980	-1.647943
C	-2.743580	-7.981625	-2.229094
C	-2.313590	-6.539057	-1.920774
C	-3.320161	-5.562713	-2.548480
C	-4.720103	-5.816707	-1.968345
C	-5.143783	-7.261240	-2.276184
C	-2.284818	-6.330802	-0.398739
C	-4.107898	-8.029682	-0.126521
C	-4.685225	-5.610784	-0.446138
C	-3.682112	-6.587140	0.186901
H	-4.446516	-9.270760	-1.869031
H	-2.021319	-8.689004	-1.804024
H	-2.750376	-8.148416	-3.313108
H	-1.316245	-6.355832	-2.335523
H	-3.013083	-4.528278	-2.352253
H	-3.336426	-5.687142	-3.638025
H	-5.436119	-5.119933	-2.417652
H	-6.150684	-7.449779	-1.884306
H	-5.192755	-7.415698	-3.360953
H	-1.554528	-7.007829	0.060768
H	-1.960413	-5.309522	-0.165111
H	-5.096221	-8.232638	0.303624
H	-3.409663	-8.737181	0.336806
H	-4.402540	-4.577120	-0.212805
H	-5.684140	-5.769432	-0.021916
H	-3.658211	-6.438501	1.272067
C	1.042075	-4.139730	-1.702069
C	2.427110	-3.870674	-2.310241
C	2.862903	-2.431841	-1.992751
C	1.842272	-1.448334	-2.585604
C	0.455505	-1.711017	-1.978329
C	0.026033	-3.151816	-2.295501
C	2.924846	-2.243256	-0.469141
C	1.109147	-3.947089	-0.179132
C	0.523558	-1.524762	-0.454711
C	1.540849	-2.508352	0.143549
H	0.733209	-5.165828	-1.929739
H	3.158909	-4.582865	-1.910260

H	2.396696	-4.023442	-3.395915
H	3.850829	-2.242407	-2.426806
H	2.152968	-0.416258	-2.382765
H	1.802246	-1.558681	-3.676051
H	-0.270574	-1.009113	-2.402813
H	-0.971944	-3.346277	-1.884170
H	-0.046571	-3.292280	-3.380848
H	3.665408	-2.925535	-0.034516
H	3.253691	-1.224801	-0.229464
H	0.130590	-4.156443	0.269860
H	1.817763	-4.659915	0.259617
H	0.810680	-0.493956	-0.214255
H	-0.465744	-1.689744	-0.010834
H	1.588399	-2.373734	1.229766
C	8.012334	-3.623137	-0.353681
C	6.521847	-3.738978	0.000740
C	5.945890	-5.033408	-0.593967
C	6.109752	-5.008770	-2.121380
C	7.599206	-4.896099	-2.481572
C	8.171045	-3.602228	-1.881702
C	6.706050	-6.239277	-0.020532
C	8.766976	-4.832576	0.219659
C	8.355341	-6.102014	-1.902983
C	8.197140	-6.129502	-0.375088
H	8.420887	-2.699643	0.071000
H	6.392187	-3.734444	1.089730
H	5.974970	-2.871038	-0.387278
H	4.883326	-5.114205	-0.339574
H	5.683979	-5.919384	-2.559964
H	5.555200	-4.163266	-2.546175
H	7.713581	-4.878157	-3.570904
H	9.229952	-3.499590	-2.148435
H	7.653280	-2.731716	-2.302544
H	6.580529	-6.281160	1.068195
H	6.291129	-7.171271	-0.423100
H	9.836593	-4.751377	-0.009086
H	8.677328	-4.848647	1.312575
H	7.969415	-7.031903	-2.337937
H	9.417393	-6.044016	-2.170651
H	8.736414	-6.990191	0.035580
C	8.020725	1.730712	-2.032690
C	7.592554	0.293547	-2.367071
C	6.217180	0.003870	-1.746276
C	6.301206	0.172679	-0.221517
C	6.726160	1.609543	0.118693
C	8.100047	1.895262	-0.507203
C	5.185415	0.992424	-2.311089
C	6.984327	2.714610	-2.597287
C	5.693430	2.594123	-0.451222
C	5.607780	2.431439	-1.976491
H	9.001472	1.935071	-2.475952
H	7.551605	0.156057	-3.454339
H	8.334813	-0.417946	-1.985257
H	5.913655	-1.021067	-1.986410

H	5.329725	-0.051071	0.235667
H	7.020554	-0.541341	0.197492
H	6.785933	1.727423	1.206158
H	8.424387	2.912202	-0.255011
H	8.851412	1.212006	-0.092968
H	5.099926	0.868610	-3.397458
H	4.194658	0.783403	-1.889731
H	7.289271	3.746134	-2.382890
H	6.931447	2.620498	-3.688665
H	4.711290	2.413317	0.002130
H	5.974773	3.623406	-0.197474
H	4.870653	3.133789	-2.380964
C	-6.753933	-8.439233	4.450965
C	-8.247437	-8.600550	4.773469
C	-9.041523	-7.443205	4.148223
C	-8.534124	-6.110465	4.719884
C	-7.041232	-5.942511	4.397438
C	-6.252299	-7.103903	5.021696
C	-8.840442	-7.452413	2.624980
C	-6.559024	-8.446355	2.926857
C	-6.845058	-5.955999	2.873605
C	-7.348213	-7.288246	2.297238
H	-6.189669	-9.265116	4.897767
H	-8.614261	-9.559975	4.389181
H	-8.397568	-8.615869	5.859829
H	-10.106083	-7.560556	4.378933
H	-9.107169	-5.276970	4.295901
H	-8.689849	-6.082398	5.805157
H	-6.681547	-4.992018	4.806387
H	-5.181593	-6.985332	4.815551
H	-6.367256	-7.093235	6.112407
H	-9.217641	-8.390688	2.200483
H	-9.418244	-6.641836	2.164607
H	-5.493675	-8.352416	2.683536
H	-6.896106	-9.402543	2.508719
H	-7.388038	-5.119602	2.417062
H	-5.784946	-5.816427	2.629377
H	-7.208046	-7.295820	1.210709
C	5.030262	-5.197538	3.602305
C	4.553758	-6.625814	3.296440
C	3.173417	-6.859376	3.929733
C	3.271899	-6.665928	5.450696
C	3.745214	-5.237742	5.762412
C	5.123909	-5.008180	5.124097
C	2.170912	-5.848138	3.352523
C	4.022977	-4.190797	3.025420
C	2.741584	-4.230536	5.180177
C	2.641675	-4.417820	3.658603
H	6.014476	-5.033202	3.150170
H	4.501935	-6.781479	2.212090
H	5.274812	-7.354068	3.687182
H	2.835412	-7.878064	3.709927
H	2.296401	-6.849692	5.917054
H	3.970193	-7.395292	5.878795

H	3.815216	-5.102282	6.847215
H	5.482531	-3.997942	5.355994
H	5.855171	-5.707927	5.546693
H	2.075022	-5.988671	2.269060
H	1.176413	-6.017202	3.782896
H	4.362461	-3.166038	3.219367
H	3.960611	-4.302800	1.936234
H	1.756801	-4.371107	5.641978
H	3.057642	-3.206539	5.413635
H	1.925319	-3.699279	3.245344
C	2.833833	-7.758601	-0.230209
C	1.338194	-7.880253	0.099713
C	0.782258	-9.187895	-0.485060
C	0.972915	-9.185295	-2.009557
C	2.467646	-9.066909	-2.345218
C	3.019378	-7.759778	-1.755348
C	1.540900	-10.379128	0.120100
C	3.586986	-8.953446	0.374814
C	3.222173	-10.258139	-1.734976
C	3.037189	-10.263538	-0.209854
H	3.228097	-6.825693	0.187343
H	1.189273	-7.860106	1.186060
H	0.791973	-7.022469	-0.311142
H	-0.284022	-9.272809	-0.248160
H	0.561587	-10.105667	-2.441509
H	0.419833	-8.350563	-2.456969
H	2.601128	-9.064703	-3.432520
H	4.082062	-7.653242	-2.004984
H	2.502825	-6.899703	-2.198545
H	1.396458	-10.405363	1.206969
H	1.139941	-11.320246	-0.275327
H	4.659862	-8.867686	0.163673
H	3.478149	-8.953540	1.466104
H	2.850761	-11.197427	-2.162303
H	4.288342	-10.196227	-1.984806
H	3.575355	-11.113784	0.223400
C	0.394403	0.754877	8.197320
C	-0.651483	1.715339	7.610617
C	-2.018057	1.456606	8.263675
C	-2.444902	0.005706	7.993231
C	-1.403670	-0.959450	8.580783
C	-0.038577	-0.694278	7.927217
C	-1.908071	1.679938	9.779860
C	0.498258	0.980570	9.713639
C	-1.294814	-0.729741	10.096078
C	-0.865147	0.719246	10.371887
H	1.367755	0.940829	7.730289
H	-0.344481	2.754712	7.778503
H	-0.721347	1.576820	6.524853
H	-2.762040	2.142834	7.844309
H	-3.429086	-0.185770	8.437726
H	-2.546482	-0.162202	6.914183
H	-1.709743	-1.993160	8.386327
H	0.710239	-1.390639	8.323855

H	-0.097384	-0.874969	6.846999
H	-1.623812	2.718465	9.988412
H	-2.882739	1.516901	10.255542
H	1.257177	0.314493	10.141870
H	0.825140	2.006897	9.919937
H	-2.258611	-0.934492	10.577730
H	-0.568744	-1.427174	10.531250
H	-0.788592	0.881301	11.452686
C	-6.930430	-0.682660	2.650771
C	-7.400549	-2.125052	2.407713
C	-8.777987	-2.338070	3.054429
C	-8.676390	-2.079187	4.565423
C	-8.209444	-0.636651	4.814340
C	-6.833645	-0.427851	4.162784
C	-9.787125	-1.357422	2.437371
C	-7.944325	0.293485	2.034245
C	-9.219700	0.339748	4.192440
C	-9.322757	0.086990	2.680574
H	-5.948276	-0.532995	2.189160
H	-7.454503	-2.327261	1.331175
H	-6.674785	-2.832348	2.827177
H	-9.111443	-3.366887	2.879414
H	-9.649692	-2.247609	5.042050
H	-7.973279	-2.786104	5.022324
H	-8.137208	-0.454519	5.892143
H	-6.479498	0.593143	4.350181
H	-6.097731	-1.105253	4.612823
H	-9.885220	-1.544693	1.361200
H	-10.779599	-1.512794	2.877472
H	-7.609490	1.327267	2.183160
H	-8.009055	0.134638	0.951038
H	-10.202516	0.214236	4.662692
H	-8.908182	1.374346	4.380930
H	-10.043834	0.783642	2.239016
C	-4.001073	0.839411	-1.197759
C	-5.501866	0.695362	-0.902031
C	-6.037272	-0.591476	-1.548823
C	-5.817462	-0.527108	-3.068030
C	-4.317443	-0.386100	-3.369639
C	-3.786334	0.899998	-2.717856
C	-5.281997	-1.801028	-0.976693
C	-3.251257	-0.373779	-0.626016
C	-3.566379	-1.595877	-2.792511
C	-3.780511	-1.663031	-1.272681
H	-3.621462	1.757448	-0.736008
H	-5.671714	0.671394	0.181163
H	-6.046140	1.565348	-1.289124
H	-7.107272	-0.692425	-1.336230
H	-6.213946	-1.432154	-3.544003
H	-6.367733	0.321255	-3.492691
H	-4.163170	-0.339856	-4.453202
H	-2.719838	1.023036	-2.942386
H	-4.300343	1.773745	-3.136593
H	-5.447050	-1.871333	0.105273

H	-5.668667	-2.728189	-1.416797
H	-2.175163	-0.272966	-0.812714
H	-3.380981	-0.417917	0.462094
H	-3.922911	-2.519757	-3.263837
H	-2.496081	-1.517397	-3.018935
H	-3.244772	-2.526426	-0.863068
C	0.822427	3.713490	-3.429337
C	1.341116	5.003650	-2.775859
C	1.123899	4.939448	-1.256106
C	-0.375981	4.782617	-0.962488
C	-0.899413	3.491902	-1.611318
C	-0.676662	3.559620	-3.130114
C	1.882849	3.732172	-0.684030
C	1.582664	2.509581	-2.851765
C	-0.135066	2.288285	-1.038768
C	1.365377	2.438786	-1.332479
H	0.978014	3.761411	-4.512668
H	2.406625	5.136748	-2.998810
H	0.818742	5.872708	-3.193970
H	1.494928	5.860013	-0.792227
H	-0.546290	4.755646	0.120620
H	-0.927958	5.648132	-1.348571
H	-1.968947	3.382449	-1.401161
H	-1.064336	2.651827	-3.608221
H	-1.234296	4.403262	-3.554718
H	2.958470	3.841023	-0.868490
H	1.751266	3.686501	0.403827
H	1.235653	1.583219	-3.325509
H	2.652760	2.597737	-3.075109
H	-0.301367	2.217104	0.042942
H	-0.513591	1.357913	-1.479326
H	1.908740	1.580062	-0.923089
C	-1.781282	3.432299	2.633700
C	-2.221144	1.986504	2.356604
C	-3.581593	1.721590	3.019894
C	-3.459746	1.939301	4.535876
C	-3.022957	3.384994	4.818885
C	-1.664131	3.645792	4.150662
C	-4.624418	2.694785	2.448757
C	-2.828746	4.400863	2.063043
C	-4.066883	4.354056	4.242803
C	-4.190350	4.142455	2.726153
H	-0.811198	3.618982	2.160207
H	-2.289112	1.814089	1.275685
H	-1.471531	1.285268	2.742998
H	-3.893497	0.690439	2.820571
H	-4.420274	1.733656	5.023681
H	-2.732130	1.236817	4.960076
H	-2.936216	3.537754	5.900175
H	-1.331142	4.669272	4.361784
H	-0.904547	2.973796	4.568314
H	-4.736856	2.536216	1.369382
H	-5.605059	2.502767	2.900760
H	-2.516038	5.437761	2.236403

H	-2.908632	4.271710	0.976905
H	-5.038053	4.191244	4.725648
H	-3.776808	5.390064	4.456131
H	-4.935412	4.833803	2.317289
C	-2.119011	-3.147818	2.228759
C	-1.556620	-1.858604	2.847004
C	-1.752836	-1.885025	4.370735
C	-3.251816	-1.995348	4.689068
C	-3.818894	-3.284892	4.075549
C	-3.617038	-3.255086	2.552621
C	-1.017755	-3.099954	4.957586
C	-1.382471	-4.359301	2.821013
C	-3.078313	-4.496299	4.662719
C	-1.578940	-4.392320	4.344419
H	-1.978337	-3.126850	1.142536
H	-0.491389	-1.758860	2.606037
H	-2.061916	-0.984813	2.417917
H	-1.350661	-0.965210	4.809463
H	-3.406654	-1.994989	5.774832
H	-3.786320	-1.123631	4.292498
H	-4.887711	-3.361167	4.303279
H	-4.035724	-4.161988	2.099635
H	-4.158350	-2.405885	2.118090
H	0.057513	-3.023906	4.755470
H	-1.134309	-3.119223	6.047943
H	-1.760941	-5.285761	2.372204
H	-0.313854	-4.304603	2.580549
H	-3.230338	-4.540260	5.747972
H	-3.487895	-5.425224	4.247622
H	-1.052520	-5.256530	4.764267
C	3.426932	2.194507	4.329591
C	1.928311	2.093245	4.652803
C	1.351487	0.806300	4.042885
C	1.547187	0.832960	2.519258
C	3.044862	0.931078	2.190246
C	3.617457	2.217493	2.805239
C	2.087145	-0.408769	4.628722
C	4.157040	0.975926	4.915208
C	3.776389	-0.284008	2.781151
C	3.586310	-0.313578	4.305370
H	3.836097	3.112684	4.765074
H	1.776184	2.096469	5.738887
H	1.398558	2.967744	4.255859
H	0.283107	0.735892	4.275034
H	1.121228	-0.072126	2.069552
H	1.010291	1.685193	2.085327
H	3.181948	0.950488	1.103563
H	4.682662	2.309809	2.560735
H	3.117513	3.094343	2.376087
H	1.938683	-0.452001	5.714505
H	1.671070	-1.335433	4.215191
H	5.231943	1.046602	4.708849
H	4.044619	0.958123	6.005990
H	3.389993	-1.208789	2.335847

```
H      4.844298     -0.236359      2.535660
H      4.108068     -1.180802      4.724821
```

Adamantane$_{23}$

```
         598
E=-984.3352
C     -1.620705     -5.913743      9.751329
C     -1.428229     -5.919127      8.227024
C      0.070501     -5.863809      7.892803
C      0.676379     -4.580772      8.482018
C      0.489359     -4.571640     10.007091
C     -1.010417     -4.630670     10.335923
C      0.773764     -7.085428      8.504243
C     -0.913334     -7.135608     10.357645
C      1.191107     -5.796751     10.613214
C      0.586640     -7.082839     10.029395
H     -2.689741     -5.952991      9.987406
H     -1.877766     -6.820774      7.793802
H     -1.945042     -5.061119      7.780169
H      0.205389     -5.868315      6.805572
H      1.742709     -4.519655      8.232896
H      0.196507     -3.699641      8.039088
H      0.921538     -3.655837     10.424912
H     -1.158844     -4.604747     11.422322
H     -1.520171     -3.750195      9.926174
H      0.364932     -8.009009      8.076819
H      1.841915     -7.067529      8.256116
H     -1.060710     -7.154587     11.444345
H     -1.352715     -8.059463      9.962429
H      2.266566     -5.756945     10.401975
H      1.082256     -5.791142     11.704583
H      1.088536     -7.955176     10.462266
C     -2.038666    -10.941329      4.033766
C     -0.657240    -10.720299      3.398634
C     -0.181903     -9.285683      3.675916
C     -1.187651     -8.291298      3.075817
C     -2.570668     -8.505938      3.709930
C     -3.039730     -9.942667      3.432916
C     -0.091549     -9.063818      5.193612
C     -1.943099    -10.715553      5.550617
C     -2.474264     -8.286552      5.227596
C     -1.471772     -9.280813      5.833153
H     -2.375754    -11.964496      3.834741
H      0.063325    -11.440860      3.803984
H     -0.708348    -10.897107      2.317394
H      0.803385     -9.130509      3.222688
H     -0.848978     -7.262770      3.249852
H     -1.247417     -8.425582      1.988963
H     -3.286148     -7.796358      3.280240
H     -4.035542    -10.103138      3.863697
H     -3.132707    -10.106135      2.352355
H      0.639040     -9.753906      5.632763
H      0.265512     -8.048505      5.404515
```

H	-2.919269	-10.890523	6.019099
H	-1.245115	-11.435293	5.995086
H	-2.158597	-7.257854	5.439620
H	-3.460183	-8.417028	5.690094
H	-1.403965	-9.122553	6.915098
C	3.361442	7.549849	2.617805
C	2.957216	6.104083	2.290781
C	1.607186	5.780849	2.949486
C	1.730789	5.947290	4.471809
C	2.131975	7.392664	4.804750
C	3.480463	7.711921	4.140974
C	0.537154	6.746787	2.417710
C	2.286893	8.511025	2.086361
C	1.060923	8.354621	4.267888
C	0.935542	8.194267	2.745124
H	4.324151	7.778140	2.147502
H	2.888428	5.968726	1.204651
H	3.726313	5.408929	2.648837
H	1.320689	4.749825	2.714548
H	0.778186	5.699789	4.955697
H	2.478111	5.248974	4.867688
H	2.220032	7.508853	5.890473
H	3.788291	8.735400	4.387346
H	4.258933	7.045117	4.531277
H	0.423565	6.624067	1.333794
H	-0.436076	6.513664	2.866387
H	2.573921	9.548738	2.295556
H	2.205063	8.418769	0.996613
H	0.096566	8.149817	4.748397
H	1.325542	9.389437	4.517083
H	0.171138	8.880462	2.364240
C	-6.415908	4.086495	8.920829
C	-7.898382	3.952367	9.301440
C	-8.453267	2.622806	8.767283
C	-8.311099	2.589691	7.237859
C	-6.829666	2.720398	6.851623
C	-6.278814	4.049518	7.391020
C	-7.658365	1.457862	9.377061
C	-5.626442	2.917860	9.530733
C	-6.038715	1.555528	7.466609
C	-6.175153	1.585938	8.996546
H	-6.022382	5.035016	9.302344
H	-8.013066	3.997772	10.391173
H	-8.469924	4.791848	8.887238
H	-9.510134	2.529040	9.040276
H	-8.722320	1.653100	6.842192
H	-8.890317	3.405116	6.787920
H	-6.730771	2.697097	5.760878
H	-5.226263	4.164526	7.104883
H	-6.821765	4.890888	6.943495
H	-7.767872	1.456980	10.468318
H	-8.057759	0.501597	9.018069
H	-4.562180	3.013213	9.283247
H	-5.700612	2.943716	10.624621

H	-6.409620	0.600700	7.074752
H	-4.982030	1.625975	7.181270
H	-5.611062	0.754443	9.433200
C	5.567019	4.850631	8.184815
C	5.151380	3.406462	7.865126
C	3.796041	3.099142	8.520537
C	3.915243	3.273917	10.042277
C	4.327816	4.717759	10.367874
C	5.681598	5.021083	9.707410
C	2.736581	4.071098	7.978653
C	4.503000	5.817867	7.643274
C	3.267322	5.685712	9.820925
C	3.146397	5.517035	8.298699
H	6.533497	5.067569	7.716874
H	5.085584	3.264989	6.779590
H	5.912962	2.706832	8.230453
H	3.501411	2.069230	8.290837
H	2.958650	3.037766	10.523960
H	4.654878	2.571551	10.445361
H	4.412713	4.839892	11.453196
H	5.997448	6.043334	9.948673
H	6.452678	4.349920	10.104845
H	2.626090	3.942668	6.895079
H	1.759624	3.849286	8.424957
H	4.798319	6.854304	7.847193
H	4.424556	5.719585	6.553804
H	2.299363	5.492336	10.298920
H	3.540053	6.719695	10.064772
H	2.389516	6.207524	7.910618
C	10.205782	-1.084027	3.551238
C	9.715852	-2.501941	3.219465
C	8.340278	-2.739429	3.861607
C	8.455433	-2.579692	5.385310
C	8.942230	-1.161974	5.722963
C	10.316080	-0.928363	5.075731
C	7.339410	-1.708869	3.316628
C	9.200105	-0.057937	3.006536
C	7.940159	-0.135327	5.172886
C	7.823616	-0.288878	3.648719
H	11.186575	-0.916878	3.092746
H	9.652012	-2.633696	2.132590
H	10.435477	-3.243562	3.587012
H	7.992681	-3.750703	3.623304
H	7.483355	-2.766633	5.857512
H	9.152666	-3.323072	5.790411
H	9.024106	-1.050555	6.809669
H	10.684352	0.074055	5.325729
H	11.046439	-1.642248	5.475634
H	7.231625	-1.825206	2.231417
H	6.348079	-1.880201	3.753367
H	9.548966	0.959957	3.219103
H	9.125992	-0.145863	1.915878
H	6.959066	-0.278924	5.641560
H	8.265986	0.881113	5.425176

H	7.108393	0.443488	3.258404
C	8.643533	6.354545	4.244303
C	7.163734	6.258169	4.646164
C	6.555617	4.964974	4.081547
C	6.671777	4.974432	2.549599
C	8.150329	5.067598	2.142032
C	8.754487	6.360325	2.711941
C	7.320444	3.755910	4.641899
C	9.402805	5.141971	4.804764
C	8.911294	3.858578	2.707660
C	8.800777	3.846212	4.239939
H	9.075045	7.277187	4.647588
H	7.068328	6.273665	5.738587
H	6.614273	7.128623	4.267541
H	5.500746	4.898069	4.369743
H	6.222757	4.064735	2.132838
H	6.113252	5.822213	2.134664
H	8.230689	5.074741	1.049539
H	9.805550	6.449001	2.411328
H	8.233120	7.232744	2.299580
H	7.228669	3.724946	5.734352
H	6.883171	2.825032	4.260990
H	10.465533	5.209426	4.541979
H	9.347290	5.136459	5.900048
H	8.502007	2.929203	2.293457
H	9.964994	3.902576	2.406420
H	9.343426	2.983291	4.641371
C	3.532257	-1.833403	9.675494
C	3.728388	-1.825962	8.151663
C	5.228074	-1.772139	7.821514
C	5.836293	-0.496124	8.423431
C	5.645628	-0.499840	9.948076
C	4.144896	-0.557325	10.272801
C	5.926275	-3.001156	8.423862
C	4.234577	-3.062633	10.272726
C	6.342316	-1.732298	10.545073
C	5.735486	-3.011414	9.948528
H	2.462545	-1.871565	9.908668
H	3.277252	-2.722435	7.709460
H	3.215172	-0.962538	7.711141
H	5.365568	-1.767490	6.734610
H	6.903395	-0.435972	8.177399
H	5.360077	0.390279	7.987124
H	6.079483	0.410976	10.374963
H	3.993930	-0.540510	11.359029
H	3.638717	0.328219	9.869597
H	5.515766	-3.919734	7.987361
H	6.995068	-2.984233	8.178449
H	4.084529	-3.090724	11.358863
H	3.793441	-3.981678	9.868356
H	7.418392	-1.693815	10.336755
H	6.230853	-1.735956	11.636186
H	6.233777	-3.889001	10.374907
C	-4.739330	-3.396125	8.260157

C	-5.731789	-2.389653	7.657977
C	-7.118784	-2.587325	8.288947
C	-7.605656	-4.017594	8.010140
C	-6.618069	-5.028548	8.613091
C	-5.232257	-4.824377	7.981596
C	-7.023090	-2.369739	9.806935
C	-4.649657	-3.175906	9.778185
C	-6.523205	-4.804540	10.130179
C	-6.033685	-3.376310	10.414393
H	-3.751328	-3.253665	7.808861
H	-5.381375	-1.365103	7.831876
H	-5.790471	-2.524476	6.571086
H	-7.824595	-1.868370	7.858573
H	-8.604365	-4.165079	8.438725
H	-7.697438	-4.180362	6.929428
H	-6.966878	-6.047470	8.412646
H	-4.521708	-5.553662	8.389309
H	-5.281867	-5.001826	6.900380
H	-6.696079	-1.345035	10.021133
H	-8.011521	-2.489251	10.266886
H	-3.928167	-3.875401	10.217682
H	-4.280580	-2.165293	9.990793
H	-7.502735	-4.966213	10.596226
H	-5.836039	-5.533894	10.576059
H	-5.967189	-3.218271	11.496451
C	-4.138922	-8.244477	-1.632198
C	-2.742142	-7.986730	-2.217992
C	-2.310795	-6.543236	-1.915966
C	-3.318670	-5.568752	-2.544471
C	-4.717157	-5.820140	-1.959711
C	-5.142193	-7.265601	-2.261266
C	-2.277894	-6.329564	-0.394765
C	-4.100876	-8.026766	-0.111638
C	-4.678150	-5.608804	-0.438346
C	-3.673719	-6.583278	0.195501
H	-4.444593	-9.273922	-1.848803
H	-2.019015	-8.692865	-1.792325
H	-2.751886	-8.157384	-3.301383
H	-1.314489	-6.361873	-2.334019
H	-3.010684	-4.533742	-2.352753
H	-3.337882	-5.697061	-3.633518
H	-5.434107	-5.124700	-2.409594
H	-6.148117	-7.452356	-1.866040
H	-5.194110	-7.423908	-3.345342
H	-1.546636	-7.005227	0.065210
H	-1.952488	-5.307581	-0.165647
H	-5.088125	-8.227809	0.321856
H	-3.401674	-8.732875	0.352350
H	-4.394459	-4.574422	-0.209455
H	-5.675992	-5.765555	-0.010904
H	-3.646874	-6.430779	1.280062
C	1.042393	-4.140511	-1.694350
C	2.427079	-3.873438	-2.304187
C	2.862623	-2.433269	-1.992470

C	1.841456	-1.452342	-2.588664
C	0.455036	-1.713046	-1.979748
C	0.025813	-3.155179	-2.291142
C	2.925205	-2.238767	-0.469630
C	1.110101	-3.941955	-0.172201
C	0.523728	-1.520872	-0.456894
C	1.541558	-2.501862	0.144710
H	0.733703	-5.167567	-1.917904
H	3.159253	-4.583877	-1.901782
H	2.396215	-4.030419	-3.389248
H	3.850301	-2.245251	-2.427703
H	2.151964	-0.419403	-2.389966
H	1.800967	-1.566922	-3.678658
H	-0.271426	-1.012987	-2.406618
H	-0.971925	-3.348314	-1.878609
H	-0.047244	-3.299865	-3.375904
H	3.666149	-2.919157	-0.032702
H	3.253881	-1.219303	-0.234048
H	0.131804	-4.149834	0.278043
H	1.819110	-4.652885	0.268985
H	0.810678	-0.489065	-0.220562
H	-0.465328	-1.684401	-0.011932
H	1.589563	-2.363025	1.230376
C	8.013743	-3.621875	-0.354744
C	6.523443	-3.737788	0.000437
C	5.948012	-5.033823	-0.591276
C	6.111337	-5.012391	-2.118794
C	7.600605	-4.899662	-2.479739
C	8.171920	-3.604177	-1.882862
C	6.709046	-6.238023	-0.015498
C	8.769262	-4.829648	0.220948
C	8.357616	-6.103898	-1.898807
C	8.199953	-6.128176	-0.370802
H	8.421922	-2.697236	0.067801
H	6.394153	-3.730975	1.089460
H	5.975945	-2.870995	-0.389267
H	4.885580	-5.114668	-0.336344
H	5.685927	-5.924190	-2.555265
H	5.556165	-4.168119	-2.545224
H	7.714597	-4.884008	-3.569147
H	9.230678	-3.501520	-2.150179
H	7.653522	-2.734867	-2.305404
H	6.583921	-6.277625	1.073360
H	6.294513	-7.171118	-0.415910
H	9.838755	-4.748341	-0.008339
H	8.679997	-4.843409	1.313926
H	7.972065	-7.034942	-2.331619
H	9.419544	-6.045882	-2.166964
H	8.739851	-6.987673	0.041539
C	8.028571	1.724589	-2.032824
C	7.599883	0.287133	-2.365287
C	6.222642	0.000072	-1.747422
C	6.302916	0.172005	-0.222810
C	6.728372	1.609175	0.115475

C	8.104134	1.892268	-0.507488
C	5.193280	0.988421	-2.316958
C	6.994570	2.708287	-2.602146
C	5.698053	2.593539	-0.459157
C	5.616163	2.427732	-1.984295
H	9.010649	1.927081	-2.473993
H	7.561593	0.147398	-3.452366
H	8.340477	-0.424263	-1.980075
H	5.918753	-1.025077	-1.986183
H	5.330050	-0.049858	0.232346
H	7.020501	-0.541819	0.199544
H	6.785467	1.729283	1.202840
H	8.428801	2.909427	-0.256598
H	8.853778	1.209168	-0.089890
H	5.110461	0.862406	-3.403280
H	4.201244	0.781229	-1.897708
H	7.299951	3.739967	-2.389131
H	6.944401	2.611932	-3.693456
H	4.714579	2.414622	-0.007951
H	5.979730	3.623084	-0.206847
H	4.880750	3.129933	-2.392134
C	-6.761916	-8.442035	4.462273
C	-8.256408	-8.585333	4.788679
C	-9.038734	-7.421041	4.161508
C	-8.514912	-6.092333	4.727719
C	-7.020955	-5.942392	4.401350
C	-6.243831	-7.110675	5.027558
C	-8.841279	-7.437404	2.637850
C	-6.570611	-8.456245	2.937757
C	-6.828458	-5.962981	2.877127
C	-7.348041	-7.291259	2.306200
H	-6.206042	-9.272846	4.910454
H	-8.634992	-9.541749	4.408319
H	-8.404202	-8.595470	5.875421
H	-10.104024	-7.525545	4.395008
H	-9.079427	-5.253738	4.302351
H	-8.667808	-6.059028	5.813246
H	-6.649555	-4.994747	4.806416
H	-5.172325	-7.004943	4.818605
H	-6.356147	-7.095214	6.118487
H	-9.230089	-8.372683	2.217236
H	-9.410895	-6.621788	2.176178
H	-5.504827	-8.375200	2.691721
H	-6.919496	-9.409872	2.523469
H	-7.362951	-5.121930	2.419122
H	-5.767394	-5.836251	2.630048
H	-7.210472	-7.303900	1.219386
C	5.023319	-5.187043	3.599079
C	4.549887	-6.616550	3.294190
C	3.170221	-6.852772	3.927968
C	3.268654	-6.658194	5.448789
C	3.738898	-5.228782	5.759531
C	5.116926	-4.996566	5.120734
C	2.165347	-5.844091	3.350401

C	4.013674	-4.182871	3.021842
C	2.732907	-4.224139	5.176943
C	2.633033	-4.412556	3.655506
H	6.007056	-5.020811	3.146600
H	4.498138	-6.772979	2.209947
H	5.272640	-7.342978	3.685190
H	2.834407	-7.872333	3.708857
H	2.293678	-6.843826	5.915501
H	3.968660	-7.385760	5.877152
H	3.808870	-5.092517	6.844235
H	5.473378	-3.985401	5.351936
H	5.849833	-5.694446	5.543568
H	2.069498	-5.985486	2.267047
H	1.171329	-6.015088	3.781124
H	4.350948	-3.157250	3.215090
H	3.951285	-4.295665	1.932740
H	1.748551	-4.366603	5.639074
H	3.046766	-3.199308	5.409708
H	1.914994	-3.695843	3.241995
C	2.836694	-7.758749	-0.232667
C	1.341516	-7.881512	0.098931
C	0.786372	-9.190559	-0.483447
C	0.975493	-9.189900	-2.008138
C	2.469761	-9.070411	-2.345468
C	3.020707	-7.761872	-1.757988
C	1.546877	-10.380141	0.122621
C	3.591714	-8.951949	0.373274
C	3.226156	-10.259987	-1.734315
C	3.042711	-10.263439	-0.209001
H	3.230393	-6.824841	0.183178
H	1.193666	-7.859997	1.185397
H	0.793978	-7.024882	-0.312577
H	-0.279580	-9.276263	-0.245354
H	0.564703	-10.111310	-2.438383
H	0.421083	-8.356379	-2.456163
H	2.602148	-9.069591	-3.432906
H	4.083027	-7.654568	-2.008844
H	2.502802	-6.902965	-2.201872
H	1.403556	-10.405002	1.209670
H	1.146514	-11.322235	-0.271082
H	4.664286	-8.865356	0.160933
H	3.483974	-8.950625	1.464672
H	2.855306	-11.200265	-2.159949
H	4.292008	-10.197303	-1.985304
H	3.582208	-11.112509	0.224903
C	0.405021	0.750455	8.201870
C	-0.628400	1.726024	7.618012
C	-1.998791	1.481241	8.268446
C	-2.442955	0.036788	7.991460
C	-1.414246	-0.943421	8.576143
C	-0.045257	-0.692180	7.925225
C	-1.887874	1.696898	9.785674
C	0.509836	0.968547	9.719234
C	-1.304380	-0.721369	10.092506

C	-0.857424	0.721113	10.374852
H	1.381113	0.926479	7.736715
H	-0.308961	2.760871	7.790586
H	-0.698670	1.592891	6.531600
H	-2.733868	2.178223	7.851115
H	-3.429919	-0.144533	8.434044
H	-2.545305	-0.125373	6.911606
H	-1.732656	-1.972506	8.377024
H	0.694556	-1.399258	8.319790
H	-0.104992	-0.867639	6.844196
H	-1.591233	2.731007	9.998884
H	-2.865014	1.543768	10.259574
H	1.260083	0.291496	10.145530
H	0.848950	1.989948	9.930188
H	-2.271165	-0.916368	10.572214
H	-0.587370	-1.429404	10.525575
H	-0.780170	0.877720	11.456404
C	-6.938706	-0.681935	2.645841
C	-7.394297	-2.123049	2.369782
C	-8.778605	-2.358410	2.993681
C	-8.699337	-2.126919	4.510447
C	-8.247000	-0.685793	4.792322
C	-6.864233	-0.454573	4.163483
C	-9.786948	-1.374069	2.381220
C	-7.951777	0.297859	2.033763
C	-9.256362	0.294382	4.174925
C	-9.337124	0.069020	2.657383
H	-5.951630	-0.516330	2.200458
H	-7.432206	-2.305600	1.289056
H	-6.668763	-2.832549	2.785910
H	-9.101676	-3.386271	2.795154
H	-9.677619	-2.311491	4.970615
H	-7.996960	-2.836907	4.963700
H	-8.190672	-0.523202	5.874190
H	-6.520578	0.565392	4.374525
H	-6.129192	-1.134677	4.610868
H	-9.869113	-1.542022	1.300537
H	-10.784009	-1.545056	2.804854
H	-7.627015	1.331181	2.206308
H	-8.000708	0.158705	0.947025
H	-10.244404	0.152760	4.629385
H	-8.955451	1.327608	4.386733
H	-10.057593	0.768324	2.219034
C	-3.993781	0.831538	-1.227469
C	-5.490949	0.680946	-0.916998
C	-6.025998	-0.610652	-1.554534
C	-5.820971	-0.550128	-3.075964
C	-4.324617	-0.402599	-3.392297
C	-3.793814	0.888254	-2.749728
C	-5.259238	-1.814543	-0.985750
C	-3.232465	-0.376020	-0.659008
C	-3.562019	-1.606719	-2.818476
C	-3.761348	-1.670000	-1.296467
H	-3.614422	1.752960	-0.772299

H	-5.650367	0.659652	0.167836
H	-6.043253	1.546934	-1.301676
H	-7.093406	-0.716261	-1.331435
H	-6.217402	-1.458689	-3.545235
H	-6.379518	0.294086	-3.498081
H	-4.180889	-0.359109	-4.477424
H	-2.730135	1.015894	-2.984801
H	-4.316174	1.758051	-3.166349
H	-5.413639	-1.882153	0.097959
H	-5.645404	-2.745053	-1.419176
H	-2.158716	-0.270432	-0.856270
H	-3.351612	-0.417266	0.430423
H	-3.918362	-2.533900	-3.283421
H	-2.494331	-1.523623	-3.055334
H	-3.217399	-2.529367	-0.889211
C	0.832493	3.718448	-3.447232
C	1.350217	5.010508	-2.796752
C	1.143834	4.944699	-1.275559
C	-0.353291	4.781357	-0.971611
C	-0.875725	3.488719	-1.617411
C	-0.663814	3.558067	-3.137684
C	1.911862	3.740406	-0.709343
C	1.601833	2.517524	-2.875529
C	-0.102311	2.288107	-1.050757
C	1.395403	2.445121	-1.354805
H	0.980348	3.767518	-4.531594
H	2.413577	5.148238	-3.027029
H	0.821264	5.877524	-3.210810
H	1.514158	5.866626	-0.813824
H	-0.515960	4.753174	0.112640
H	-0.911610	5.644690	-1.353438
H	-1.943298	3.374622	-1.399887
H	-1.050937	2.648848	-3.613524
H	-1.227965	4.399521	-3.558003
H	2.985704	3.853914	-0.901214
H	1.788031	3.693686	0.379379
H	1.255481	1.589907	-3.347297
H	2.669968	2.610333	-3.106252
H	-0.260793	2.215733	0.032046
H	-0.479927	1.356332	-1.489123
H	1.945241	1.588533	-0.949608
C	-1.775456	3.442321	2.612824
C	-2.207197	1.999233	2.310036
C	-3.582965	1.726443	2.937570
C	-3.497826	1.928255	4.458251
C	-3.069299	3.371177	4.766874
C	-1.695030	3.639903	4.134326
C	-4.612641	2.704906	2.351765
C	-2.809776	4.416146	2.027338
C	-4.099953	4.345559	4.176056
C	-4.186702	4.149862	2.654733
H	-0.794433	3.634627	2.164803
H	-2.248981	1.838168	1.226042
H	-1.466452	1.294465	2.706964

H	-3.889037	0.697241	2.719968
H	-4.469622	1.716845	4.920609
H	-2.779980	1.221826	4.892430
H	-3.008738	3.512597	5.851485
H	-1.368157	4.661323	4.364150
H	-0.945098	2.964050	4.563049
H	-4.698932	2.557640	1.268390
H	-5.603696	2.507485	2.777993
H	-2.502282	5.451368	2.219066
H	-2.863390	4.298387	0.938294
H	-5.082308	4.177024	4.633650
H	-3.816046	5.379455	4.407184
H	-4.922347	4.844983	2.235348
C	-2.131518	-3.133253	2.237956
C	-1.568462	-1.844143	2.855813
C	-1.764681	-1.870009	4.379553
C	-3.263715	-1.979469	4.697930
C	-3.831458	-3.268905	4.084799
C	-3.629597	-3.239657	2.561860
C	-1.030218	-3.085139	4.966761
C	-1.395595	-4.344937	2.830567
C	-3.091494	-4.480516	4.672325
C	-1.592070	-4.377400	4.353984
H	-1.990841	-3.112681	1.151726
H	-0.503182	-1.745017	2.614808
H	-2.073314	-0.970221	2.426468
H	-1.362031	-0.950270	4.818003
H	-3.418545	-1.978707	5.783694
H	-3.797776	-1.107596	4.301103
H	-4.900313	-3.344565	4.312559
H	-4.048752	-4.146479	2.109148
H	-4.170478	-2.390308	2.127080
H	0.045087	-3.009702	4.764615
H	-1.146775	-3.104023	6.057124
H	-1.774543	-5.271337	2.382037
H	-0.326951	-4.290858	2.590078
H	-3.243534	-4.524076	5.757592
H	-3.501555	-5.409355	4.257508
H	-1.066089	-5.241754	4.774086
C	3.414901	2.202854	4.331300
C	1.915787	2.098153	4.651108
C	1.342840	0.810962	4.038064
C	1.541791	0.840236	2.514906
C	3.039979	0.941798	2.189292
C	3.608687	2.228444	2.807401
C	2.079633	-0.403503	4.623727
C	4.146152	0.984865	4.916727
C	3.772631	-0.272699	2.780014
C	3.579304	-0.304873	4.303771
H	3.821300	3.121201	4.769009
H	1.761297	2.099489	5.736863
H	1.385163	2.972181	4.254288
H	0.274100	0.738100	4.267789
H	1.118604	-0.065033	2.062958

H	1.004149	1.692038	2.081052
H	3.179384	0.963067	1.102939
H	4.674235	2.323225	2.565346
H	3.107939	3.104928	2.378442
H	1.928900	-0.448616	5.709121
H	1.666293	-1.330384	4.207943
H	5.221358	1.057970	4.712806
H	4.031400	0.965246	6.007234
H	3.389035	-1.197592	2.332525
H	4.840972	-0.222579	2.536913
H	4.101869	-1.171675	4.723091

Adamantane$_{24}$

624
E=-1049.3692

C	-1.626771	-5.922076	9.752326
C	-1.435542	-5.934895	8.227908
C	0.062627	-5.874250	7.892101
C	0.662852	-4.585122	8.473759
C	0.477063	-4.568525	9.998919
C	-1.022140	-4.632917	10.329359
C	0.772234	-7.089125	8.509617
C	-0.913059	-7.137206	10.364716
C	1.185167	-5.786929	10.611140
C	0.586371	-7.079070	10.034893
H	-2.695409	-5.965138	9.989534
H	-1.881123	-6.841040	7.800018
H	-1.956825	-5.081829	7.776806
H	0.196629	-5.884070	6.804795
H	1.728670	-4.520277	8.223390
H	0.178403	-3.708735	8.026423
H	0.905205	-3.648391	10.411347
H	-1.169783	-4.601750	11.415727
H	-1.536439	-3.757147	9.915233
H	0.367468	-8.016978	8.087610
H	1.840080	-7.067482	8.260479
H	-1.059436	-7.150932	11.451631
H	-1.348345	-8.065302	9.974945
H	2.260247	-5.743142	10.398761
H	1.077200	-5.775857	11.702554
H	1.092795	-7.946614	10.472104
C	-2.035876	-10.935622	4.030721
C	-0.654318	-10.713653	3.396205
C	-0.179306	-9.279260	3.675192
C	-1.185065	-8.284391	3.075917
C	-2.568215	-8.499968	3.709421
C	-3.036953	-9.936473	3.430701
C	-0.089399	-9.059050	5.193156
C	-1.940757	-10.711498	5.547846
C	-2.472258	-8.282235	5.227354
C	-1.469755	-9.276988	5.832086
H	-2.372732	-11.958628	3.830481
H	0.066264	-11.434534	3.800955

H	-0.705104	-10.889280	2.314757
H	0.806078	-9.123417	3.222401
H	-0.846618	-7.255996	3.251174
H	-1.244515	-8.417490	1.988900
H	-3.283704	-7.790041	3.280320
H	-4.032853	-10.097591	3.861037
H	-3.129611	-10.098769	2.349935
H	0.641193	-9.749493	5.631745
H	0.267429	-8.043907	5.405272
H	-2.917023	-10.887153	6.015871
H	-1.242767	-11.431605	5.991710
H	-2.156827	-7.253716	5.440594
H	-3.458278	-8.413392	5.689442
H	-1.402267	-9.119907	6.914223
C	3.417772	7.548826	2.585105
C	2.987162	6.112227	2.251428
C	1.605601	5.829407	2.861558
C	1.677507	6.002488	4.386458
C	2.104850	7.438816	4.725937
C	3.484951	7.717667	4.110705
C	0.582208	6.819997	2.285200
C	2.389726	8.534779	2.008939
C	1.080519	8.425421	4.144482
C	1.007018	8.258469	2.619041
H	4.402927	7.748294	2.149454
H	2.954792	5.971622	1.164270
H	3.723602	5.399195	2.641549
H	1.300351	4.804861	2.621942
H	0.701430	5.783637	4.836165
H	2.390717	5.287124	4.813588
H	2.155970	7.559743	5.813509
H	3.810886	8.734177	4.362577
H	4.230329	7.032937	4.533239
H	0.505343	6.693233	1.198527
H	-0.412791	6.615837	2.698878
H	2.696634	9.565828	2.222811
H	2.345619	8.437582	0.917441
H	0.093973	8.249541	4.590140
H	1.363491	9.454433	4.397514
H	0.275865	8.962270	2.206332
C	-1.231627	8.142546	8.913994
C	-1.054776	8.148346	7.387798
C	-1.613892	6.845485	6.795434
C	-0.859447	5.648870	7.394910
C	-1.035566	5.636446	8.921257
C	-0.478301	6.942567	9.508156
C	-3.106811	6.729378	7.139679
C	-2.725689	8.023528	9.252459
C	-2.529947	5.523898	9.260215
C	-3.288644	6.720090	8.665445
H	-0.832221	9.072034	9.334276
H	-1.571038	9.012269	6.952087
H	0.006999	8.251928	7.133131
H	-1.487270	6.852037	5.707220

H	-1.236154	4.713026	6.964624
H	0.205530	5.708813	7.139790
H	-0.496453	4.782888	9.346514
H	-0.579946	6.937995	10.600224
H	0.593569	7.025283	9.290828
H	-3.661452	7.567168	6.700001
H	-3.522464	5.811986	6.705562
H	-2.867297	8.038948	10.339982
H	-3.272636	8.884608	8.849781
H	-2.936070	4.585710	8.863120
H	-2.667900	5.493523	10.347878
H	-4.353925	6.637858	8.907653
C	-6.391187	4.025791	8.994890
C	-7.884301	3.907693	9.337307
C	-8.450751	2.606630	8.747967
C	-8.273716	2.619702	7.221882
C	-6.781574	2.734580	6.873711
C	-6.219285	4.035112	7.468181
C	-7.691581	1.407945	9.337549
C	-5.637522	2.823517	9.584358
C	-6.026474	1.535878	7.468411
C	-6.197842	1.519979	8.995050
H	-5.989420	4.954012	9.415720
H	-8.023425	3.920429	10.425063
H	-8.430680	4.770659	8.937667
H	-9.515208	2.524238	8.993789
H	-8.692625	1.704191	6.786897
H	-8.827376	3.459915	6.785581
H	-6.657806	2.744265	5.785301
H	-5.158450	4.139233	7.209815
H	-6.736277	4.900488	7.035953
H	-7.826410	1.374278	10.425448
H	-8.099879	0.471378	8.938842
H	-4.566236	2.906540	9.364240
H	-5.736602	2.815816	10.676549
H	-6.405504	0.601257	7.037555
H	-4.962337	1.595365	7.209494
H	-5.659287	0.664417	9.417215
C	5.588276	4.845500	8.177772
C	5.143132	3.410366	7.857114
C	3.775327	3.135032	8.500740
C	3.884631	3.310348	10.023161
C	4.326648	4.745235	10.349700
C	5.692852	5.016599	9.701014
C	2.742985	4.129418	7.947335
C	4.551266	5.835270	7.624678
C	3.293227	5.735631	9.791228
C	3.182343	5.566451	8.268293
H	6.563598	5.039628	7.718239
H	5.084014	3.268080	6.771300
H	5.885420	2.694562	8.230725
H	3.459672	2.111521	8.270365
H	2.918633	3.096818	10.496563
H	4.604574	2.592383	10.434333

H	4.404429	4.867757	11.435511
H	6.029444	6.031999	9.943160
H	6.444985	4.329109	10.106764
H	2.639461	4.001181	6.863050
H	1.757273	3.930606	8.385146
H	4.867991	6.865234	7.829267
H	4.480511	5.736439	6.534731
H	2.316864	5.565114	10.260756
H	3.586954	6.763736	10.035542
H	2.444756	6.272962	7.871990
C	10.193455	-1.065932	3.545238
C	9.707697	-2.486223	3.217524
C	8.332542	-2.725700	3.859826
C	8.446620	-2.561068	5.383089
C	8.929244	-1.140964	5.716682
C	10.302688	-0.905385	5.069302
C	7.328969	-1.699631	3.311359
C	9.185085	-0.044347	2.997060
C	7.924480	-0.118828	5.163129
C	7.808997	-0.277279	3.639382
H	11.173956	-0.897368	3.086639
H	9.644676	-2.621418	2.131024
H	10.429278	-3.224688	3.587581
H	7.987920	-3.738669	3.624418
H	7.474885	-2.749359	5.855461
H	9.145800	-3.301243	5.790696
H	9.010359	-1.026056	6.803082
H	10.668004	0.098822	5.316439
H	11.034912	-1.615987	5.471636
H	7.221958	-1.819527	2.226458
H	6.337951	-1.872474	3.748213
H	9.530962	0.975169	3.206713
H	9.111668	-0.135753	1.906641
H	6.943609	-0.263810	5.631839
H	8.247311	0.899288	5.412499
H	7.091852	0.451875	3.246587
C	8.629508	6.366606	4.238269
C	7.148068	6.261607	4.631836
C	6.549519	4.967072	4.060108
C	6.673888	4.981697	2.528845
C	8.154128	5.083498	2.129555
C	8.748692	6.377528	2.706553
C	7.317389	3.760188	4.620999
C	9.391840	5.156184	4.799229
C	8.918107	3.876623	2.695702
C	8.799394	3.859118	4.227317
H	9.054191	7.290190	4.646616
H	7.046697	6.273354	5.723769
H	6.596283	7.130422	4.252831
H	5.493460	4.894014	4.342397
H	6.231705	4.071010	2.106967
H	6.113349	5.827904	2.113412
H	8.240340	5.094313	1.037539
H	9.800901	6.472374	2.411891

H	8.225172	7.248550	2.293969
H	7.219883	3.725496	5.712843
H	6.886866	2.828273	4.234972
H	10.455616	5.229756	4.542394
H	9.330450	5.147117	5.894176
H	8.515742	2.946450	2.276538
H	9.973181	3.926807	2.400295
H	9.344207	2.997732	4.629116
C	3.531576	-1.823451	9.671236
C	3.724678	-1.831517	8.147021
C	5.223280	-1.770353	7.813251
C	5.823251	-0.483289	8.399722
C	5.635590	-0.471449	9.924697
C	4.135959	-0.536343	10.253087
C	5.931691	-2.987433	8.427793
C	4.244098	-3.040770	10.280656
C	6.342503	-1.692040	10.533931
C	5.743954	-2.982135	9.952863
H	2.462631	-1.866875	9.906991
H	3.279299	-2.736143	7.715718
H	3.204256	-0.976839	7.697979
H	5.358617	-1.776783	6.726085
H	6.889400	-0.418039	8.150870
H	5.339667	0.394684	7.954565
H	6.063554	0.447221	10.340560
H	3.986989	-0.508558	11.339366
H	3.622483	0.340920	9.841101
H	5.527112	-3.913799	8.002355
H	6.999850	-2.965389	8.180038
H	4.096377	-3.057882	11.367340
H	3.808959	-3.967471	9.887415
H	7.417859	-1.647972	10.323014
H	6.233196	-1.684382	11.625241
H	6.249527	-3.851241	10.387950
C	-4.753873	-3.404729	8.263250
C	-5.749977	-2.400846	7.662768
C	-7.138777	-2.611929	8.285369
C	-7.614702	-4.043494	7.994572
C	-6.623444	-5.051896	8.595770
C	-5.235868	-4.834319	7.972681
C	-7.052163	-2.403887	9.805240
C	-4.673297	-3.194093	9.783147
C	-6.537697	-4.837431	10.114778
C	-6.059138	-3.407924	10.410982
H	-3.764588	-3.252703	7.817926
H	-5.407275	-1.375184	7.845266
H	-5.802298	-2.528771	6.574720
H	-7.847191	-1.894802	7.856225
H	-8.614547	-4.200464	8.417097
H	-7.699968	-4.199625	6.912348
H	-6.964446	-6.071743	8.386773
H	-4.522534	-5.561592	8.379121
H	-5.278862	-5.004846	6.890068
H	-6.733066	-1.378495	10.027917

H	-8.042073	-2.533026	10.259369
H	-3.949380	-3.891718	10.221623
H	-4.312034	-2.182503	10.004353
H	-7.518458	-5.008711	10.574767
H	-5.847936	-5.565185	10.559264
H	-5.999133	-3.256699	11.494393
C	-4.132556	-8.242327	-1.635619
C	-2.735440	-7.983114	-2.219963
C	-2.305445	-6.539518	-1.916501
C	-3.313478	-5.565331	-2.545210
C	-4.712305	-5.818186	-1.961897
C	-5.135989	-7.263744	-2.264884
C	-2.274077	-6.326926	-0.395116
C	-4.096047	-8.025692	-0.114868
C	-4.674831	-5.607924	-0.440345
C	-3.670245	-6.582109	0.193704
H	-4.437263	-9.271843	-1.853246
H	-2.012171	-8.689018	-1.794154
H	-2.744077	-8.152989	-3.303485
H	-1.308897	-6.357108	-2.333520
H	-3.006439	-4.530230	-2.352464
H	-3.331609	-5.692863	-3.634366
H	-5.429367	-5.122955	-2.411924
H	-6.142130	-7.451537	-1.870703
H	-5.186808	-7.421304	-3.349122
H	-1.542731	-7.002376	0.065030
H	-1.949642	-5.304867	-0.164963
H	-5.083537	-8.227785	0.317587
H	-3.396737	-8.731615	0.349241
H	-4.392119	-4.573497	-0.210448
H	-5.672941	-5.765730	-0.013920
H	-3.644495	-6.430376	1.278399
C	1.041299	-4.136984	-1.686820
C	2.425306	-3.869890	-2.298187
C	2.859815	-2.428734	-1.989600
C	1.837355	-1.449867	-2.586962
C	0.451607	-1.710596	-1.976527
C	0.023421	-3.153709	-2.284793
C	2.923334	-2.231204	-0.467188
C	1.109941	-3.935397	-0.165111
C	0.521239	-1.515388	-0.454102
C	1.540365	-2.494310	0.148674
H	0.733347	-5.164744	-1.908143
H	3.158396	-4.578901	-1.894930
H	2.393790	-4.029017	-3.382916
H	3.847010	-2.240702	-2.425919
H	2.147101	-0.416272	-2.390507
H	1.796173	-1.566612	-3.676701
H	-0.275779	-1.012008	-2.404233
H	-0.973846	-3.346912	-1.871156
H	-0.050297	-3.300577	-3.369216
H	3.665192	-2.910091	-0.029473
H	3.251287	-1.210995	-0.233837
H	0.132155	-4.143252	0.286251

H	1.819893	-4.644842	0.276945
H	0.807456	-0.482871	-0.219994
H	-0.467350	-1.678914	-0.008100
H	1.589038	-2.353312	1.234031
C	8.012171	-3.615449	-0.355071
C	6.521924	-3.732479	-0.000033
C	5.947970	-5.029864	-0.590222
C	6.111572	-5.010188	-2.117735
C	7.600791	-4.896349	-2.478532
C	8.170628	-3.599512	-1.883179
C	6.710156	-6.232536	-0.012778
C	8.768846	-4.821701	0.222291
C	8.358953	-6.099056	-1.895934
C	8.201017	-6.121571	-0.367931
H	8.419296	-2.689850	0.066386
H	6.392414	-3.724428	1.088955
H	5.973592	-2.866755	-0.390937
H	4.885575	-5.111503	-0.335394
H	5.687205	-5.922983	-2.553137
H	5.555596	-4.167037	-2.545337
H	7.714979	-4.881949	-3.567937
H	9.229330	-3.496081	-2.150420
H	7.651400	-2.731281	-2.306918
H	6.584860	-6.270896	1.076105
H	6.296682	-7.166570	-0.412093
H	9.838297	-4.739560	-0.006890
H	8.679382	-4.834177	1.315268
H	7.974465	-7.031049	-2.327646
H	9.420872	-6.040262	-2.163958
H	8.741737	-6.979980	0.045599
C	8.018827	1.727550	-2.042315
C	7.593729	0.286944	-2.365627
C	6.218187	-0.000323	-1.744083
C	6.300150	0.180881	-0.220634
C	6.722026	1.621227	0.108506
C	8.096092	1.904492	-0.518110
C	5.185225	0.981724	-2.317984
C	6.981239	2.704934	-2.615968
C	5.688110	2.599257	-0.470467
C	5.604514	2.424170	-1.994476
H	8.999698	1.930186	-2.486094
H	7.554283	0.140652	-3.451801
H	8.336879	-0.420064	-1.977271
H	5.916856	-1.027723	-1.976320
H	5.328565	-0.041016	0.237233
H	7.020351	-0.528398	0.204910
H	6.780331	1.747947	1.195055
H	8.418240	2.924032	-0.273727
H	8.848258	1.225998	-0.097551
H	5.101212	0.849031	-3.403420
H	4.194377	0.774231	-1.896082
H	7.284007	3.738717	-2.409518
H	6.929787	2.601963	-3.706614
H	4.705790	2.420251	-0.016790

H	5.967237	3.631071	-0.224675
H	4.866539	3.121863	-2.405411
C	-6.760478	-8.446628	4.449807
C	-8.255109	-8.593750	4.773870
C	-9.039298	-7.430919	4.146314
C	-8.519532	-6.101339	4.714212
C	-7.025452	-5.947575	4.390189
C	-6.246454	-7.114409	5.016773
C	-8.839518	-7.445769	2.622943
C	-6.566852	-8.459338	2.925571
C	-6.830619	-5.966662	2.866243
C	-7.346140	-7.295799	2.293636
H	-6.203276	-9.276402	4.898259
H	-8.630814	-9.550816	4.392293
H	-8.404508	-8.604984	5.860381
H	-10.104682	-7.538150	4.378142
H	-9.085428	-5.263818	4.288564
H	-8.674136	-6.069143	5.799530
H	-6.656947	-4.999314	4.796455
H	-5.174894	-7.005952	4.809501
H	-6.360444	-7.099962	6.107541
H	-9.225439	-8.381695	2.201112
H	-9.410405	-6.631213	2.160970
H	-5.500898	-8.375557	2.681192
H	-6.912814	-9.413520	2.510113
H	-7.366451	-5.126590	2.408007
H	-5.769494	-5.837206	2.620845
H	-7.206910	-7.307367	1.207022
C	5.017165	-5.168055	3.598549
C	4.546672	-6.599009	3.295909
C	3.168497	-6.837801	3.931964
C	3.268694	-6.641330	5.452427
C	3.736007	-5.210472	5.760923
C	5.112546	-4.975695	5.119857
C	2.160396	-5.832131	3.354772
C	4.004304	-4.166904	3.021698
C	2.726791	-4.208847	5.178716
C	2.625139	-4.399161	3.657633
H	5.999844	-4.999992	3.144448
H	4.493708	-6.756745	2.211914
H	5.271713	-7.323299	3.686640
H	2.834776	-7.858392	3.714456
H	2.294843	-6.828758	5.920768
H	3.971044	-7.366771	5.880556
H	3.807243	-5.072857	6.845375
H	5.466943	-3.963439	5.349435
H	5.847720	-5.671379	5.542375
H	2.063297	-5.974935	2.271714
H	1.167416	-6.005007	3.787137
H	4.339433	-3.140278	3.213335
H	3.940590	-4.281035	1.932812
H	1.743452	-4.353133	5.642443
H	3.038564	-3.183023	5.409906
H	1.904801	-3.684600	3.244394

C	2.839571	-7.752470	-0.229171
C	1.344372	-7.876853	0.101724
C	0.791226	-9.187138	-0.479771
C	0.981178	-9.187615	-2.004358
C	2.475475	-9.066512	-2.340984
C	3.024420	-7.756737	-1.754389
C	1.552926	-10.375193	0.127787
C	3.595791	-8.944151	0.378261
C	3.233063	-10.254563	-1.728342
C	3.048790	-10.256871	-0.203126
H	3.231844	-6.817681	0.186043
H	1.195901	-7.854544	1.188090
H	0.795959	-7.021299	-0.310858
H	-0.274745	-9.273995	-0.242181
H	0.571805	-10.109941	-2.433993
H	0.425942	-8.355212	-2.453440
H	2.608454	-9.066507	-3.428350
H	4.086738	-7.648296	-2.004763
H	2.505656	-6.898898	-2.199331
H	1.409044	-10.399255	1.214781
H	1.153988	-11.318157	-0.265280
H	4.668367	-8.856373	0.166425
H	3.487455	-8.941978	1.469598
H	2.863652	-11.195702	-2.153326
H	4.298970	-10.190738	-1.978808
H	3.589140	-11.104855	0.231841
C	0.415365	0.728890	8.223824
C	-0.606353	1.721713	7.648518
C	-1.981396	1.482414	8.291129
C	-2.438693	0.045931	7.994747
C	-1.421735	-0.951496	8.570805
C	-0.048029	-0.705720	7.927786
C	-1.873803	1.678222	9.811285
C	0.516879	0.927191	9.744126
C	-1.315128	-0.749273	10.090172
C	-0.855077	0.685147	10.391898
H	1.394794	0.901031	7.764263
H	-0.277413	2.751158	7.834994
H	-0.674035	1.602700	6.560305
H	-2.708111	2.191694	7.879919
H	-3.428964	-0.131121	8.431637
H	-2.538761	-0.101872	6.912622
H	-1.749495	-1.974861	8.357861
H	0.683405	-1.424860	8.316123
H	-0.105609	-0.867223	6.844467
H	-1.567813	2.706651	10.038306
H	-2.854087	1.528875	10.279871
H	1.258927	0.237580	10.164600
H	0.865224	1.942524	9.968880
H	-2.285487	-0.940657	10.564083
H	-0.606642	-1.469620	10.516919
H	-0.780165	0.827622	11.475567
C	-6.946127	-0.682985	2.646193
C	-7.397895	-2.125828	2.372893

C	-8.783930	-2.362050	2.992617
C	-8.710477	-2.125822	4.508944
C	-8.261984	-0.682957	4.788049
C	-6.877463	-0.450893	4.163393
C	-9.792044	-1.381566	2.373627
C	-7.958962	0.292943	2.027584
C	-9.271092	0.293344	4.164133
C	-9.346049	0.063241	2.647004
H	-5.957813	-0.516767	2.203794
H	-7.431626	-2.311716	1.292597
H	-6.672435	-2.832626	2.793723
H	-9.104269	-3.391145	2.796064
H	-9.690012	-2.310944	4.966217
H	-8.008305	-2.833042	4.966819
H	-8.209798	-0.516987	5.869612
H	-6.536570	0.570385	4.372557
H	-6.142667	-1.128183	4.615423
H	-9.870061	-1.552945	1.293174
H	-10.790256	-1.553252	2.794258
H	-7.636851	1.327424	2.198144
H	-8.003779	0.150411	0.941105
H	-10.260451	0.151135	4.615532
H	-8.972971	1.327800	4.373872
H	-10.066344	0.759786	2.204003
C	-3.997584	0.829400	-1.230530
C	-5.494655	0.679947	-0.919039
C	-6.030743	-0.612094	-1.554801
C	-5.826547	-0.553409	-3.076414
C	-4.330296	-0.407026	-3.393764
C	-3.798451	0.884278	-2.752966
C	-5.264292	-1.815739	-0.985081
C	-3.236579	-0.377910	-0.661126
C	-3.568004	-1.610894	-2.819004
C	-3.766503	-1.672337	-1.296811
H	-3.617484	1.751139	-0.776624
H	-5.653469	0.659972	0.165908
H	-6.046722	1.545787	-1.304390
H	-7.098080	-0.716888	-1.330978
H	-6.223721	-1.462296	-3.544426
H	-6.384891	0.290617	-3.499176
H	-4.187160	-0.364847	-4.479020
H	-2.734838	1.011092	-2.988786
H	-4.320590	1.753874	-3.170280
H	-5.418114	-1.882035	0.098792
H	-5.651192	-2.746540	-1.417229
H	-2.162887	-0.273111	-0.859116
H	-3.355131	-0.417853	0.428419
H	-3.925097	-2.538416	-3.282691
H	-2.500407	-1.528628	-3.056561
H	-3.222774	-2.531525	-0.888885
C	0.816524	3.704589	-3.465123
C	1.346096	4.997266	-2.825497
C	1.150179	4.940991	-1.302538
C	-0.345636	4.787772	-0.987089

C	-0.879887	3.494569	-1.621999
C	-0.678417	3.554357	-3.144096
C	1.915459	3.735516	-0.735116
C	1.583173	2.502506	-2.892242
C	-0.109198	2.292748	-1.054197
C	1.387171	2.439635	-1.369716
H	0.956931	3.746858	-4.550761
H	2.408545	5.127726	-3.064064
H	0.819075	5.864962	-3.240594
H	1.528940	5.863339	-0.848555
H	-0.500739	4.766471	0.098428
H	-0.901813	5.652128	-1.369727
H	-1.946507	3.387695	-1.396286
H	-1.074004	2.644734	-3.612136
H	-1.240822	4.396656	-3.565062
H	2.988528	3.841907	-0.935217
H	1.799121	3.695485	0.354699
H	1.228281	1.574278	-3.356397
H	2.650142	2.588017	-3.131040
H	-0.260372	2.227226	0.030087
H	-0.495139	1.360721	-1.484707
H	1.935070	1.582200	-0.963686
C	-1.782319	3.445822	2.609204
C	-2.215832	2.002189	2.311588
C	-3.595870	1.736069	2.932577
C	-3.519032	1.945770	4.452633
C	-3.088769	3.389276	4.756067
C	-1.710211	3.651308	4.130077
C	-4.619744	2.713884	2.335626
C	-2.810851	4.419011	2.012570
C	-4.113601	4.362985	4.154118
C	-4.192029	4.159400	2.633379
H	-0.798254	3.633368	2.165863
H	-2.251740	1.835455	1.228240
H	-1.479122	1.297757	2.716541
H	-3.903195	0.706466	2.718674
H	-4.494001	1.739174	4.910467
H	-2.805441	1.239931	4.894711
H	-3.034135	3.536322	5.840245
H	-1.382174	4.673145	4.356364
H	-0.964426	2.975938	4.566723
H	-4.700128	2.561060	1.252566
H	-5.613726	2.521131	2.757144
H	-2.501938	5.454494	2.200576
H	-2.858453	4.295585	0.923874
H	-5.098996	4.199263	4.606899
H	-3.828507	5.397408	4.381394
H	-4.923533	4.854053	2.206051
C	-2.139914	-3.126148	2.241030
C	-1.577585	-1.835578	2.856498
C	-1.771805	-1.859712	4.380522
C	-3.270295	-1.970532	4.700978
C	-3.837305	-3.261427	4.090243
C	-3.637446	-3.233903	2.567008

C	-1.035147	-3.073216	4.968344
C	-1.401793	-4.336196	2.834247
C	-3.095149	-4.471405	4.678370
C	-1.596261	-4.366928	4.357957
H	-2.000665	-3.106810	1.154592
H	-0.512735	-1.735504	2.613988
H	-2.084025	-0.962807	2.426681
H	-1.369677	-0.938933	4.817267
H	-3.423723	-1.968553	5.786940
H	-3.805899	-1.099803	4.303721
H	-4.905774	-3.338055	4.319483
H	-4.056112	-4.141803	2.116006
H	-4.179892	-2.385755	2.131835
H	0.039806	-2.996769	4.764709
H	-1.150272	-3.090832	6.058880
H	-1.780224	-5.263621	2.387401
H	-0.333526	-4.281165	2.592307
H	-3.245735	-4.513745	5.763888
H	-3.504647	-5.401261	4.265280
H	-1.068716	-5.230118	4.778492
C	3.397504	2.234126	4.324541
C	1.898627	2.130085	4.645677
C	1.326111	0.839603	4.039183
C	1.524211	0.861624	2.515792
C	3.022160	0.962497	2.188866
C	3.590440	2.252456	2.800429
C	2.063947	-0.371577	4.630310
C	4.129799	1.019418	4.915451
C	3.755857	-0.248693	2.785055
C	3.563384	-0.273607	4.309056
H	3.803595	3.154821	4.757577
H	1.744731	2.136584	5.731497
H	1.367266	3.001868	4.244924
H	0.257541	0.767220	4.269846
H	1.101315	-0.046072	2.068462
H	0.985824	1.710998	2.078117
H	3.160956	0.978592	1.102346
H	4.655798	2.346697	2.557332
H	3.088935	3.126558	2.367510
H	1.913836	-0.411526	5.715992
H	1.650932	-1.300704	4.219244
H	5.204850	1.092172	4.710587
H	4.015657	1.005007	6.006104
H	3.372567	-1.175967	2.342257
H	4.824035	-0.199116	2.541125
H	4.086694	-1.138060	4.732277

Adamantane$_{25}$

650
E=-1091.4360

C	-1.629489	-5.918611	9.753382
C	-1.437152	-5.931141	8.229101
C	0.061307	-5.871519	7.894409

C	0.662068	-4.583004	8.476872
C	0.475173	-4.566700	10.001900
C	-1.024319	-4.630067	10.331224
C	0.769555	-7.087098	8.512100
C	-0.917133	-7.134447	10.365950
C	1.181919	-5.785805	10.614294
C	0.582582	-7.077336	10.037242
H	-2.698333	-5.960942	9.989794
H	-1.883095	-6.836833	7.800627
H	-1.957468	-5.077559	7.777859
H	0.196099	-5.881131	6.807199
H	1.728117	-4.518883	8.227302
H	0.178600	-3.706129	8.029429
H	0.903699	-3.647002	10.414902
H	-1.172735	-4.599097	11.417492
H	-1.537662	-3.753796	9.916969
H	0.364406	-8.014529	8.089534
H	1.837599	-7.066181	8.263750
H	-1.064316	-7.148371	11.452753
H	-1.352825	-8.062108	9.975597
H	2.257186	-5.742761	10.402715
H	1.073161	-5.774962	11.705632
H	1.088038	-7.945381	10.474578
C	-2.031456	-10.943806	4.035425
C	-0.649244	-10.722392	3.402140
C	-0.174268	-9.287932	3.680845
C	-1.179281	-8.293211	3.080073
C	-2.563083	-8.508233	3.712342
C	-3.031784	-9.944806	3.433911
C	-0.085804	-9.066948	5.198781
C	-1.937777	-10.718910	5.552525
C	-2.468568	-8.289727	5.230254
C	-1.466818	-9.284329	5.836479
H	-2.368284	-11.966861	3.835388
H	0.070826	-11.443181	3.807967
H	-0.699005	-10.898573	2.320735
H	0.811581	-9.132485	3.228933
H	-0.840837	-7.264780	3.255126
H	-1.237694	-8.426864	1.993068
H	-3.278037	-7.798412	3.282175
H	-4.028129	-10.105538	3.863361
H	-3.123415	-10.107648	2.353141
H	0.644247	-9.757282	5.638440
H	0.270983	-8.051754	5.410717
H	-2.914527	-10.894162	6.019691
H	-1.240338	-11.438899	5.997443
H	-2.153177	-7.261149	5.443268
H	-3.455060	-8.420483	5.691450
H	-1.400358	-9.126696	6.918600
C	3.426900	7.552126	2.589120
C	2.980347	6.122796	2.245332
C	1.598970	5.848539	2.859770
C	1.679918	6.007834	4.385719
C	2.123230	7.436875	4.735323

C	3.503083	7.707223	4.115756
C	0.582783	6.854172	2.296870
C	2.406016	8.553166	2.026394
C	1.106041	8.438576	4.167296
C	1.023555	8.285430	2.640866
H	4.411900	7.745494	2.150377
H	2.941357	5.991833	1.157187
H	3.711482	5.399151	2.625768
H	1.282342	4.829174	2.612935
H	0.703870	5.794875	4.838308
H	2.387979	5.281771	4.803257
H	2.180774	7.547972	5.823622
H	3.840374	8.718242	4.374678
H	4.243594	7.011510	4.528801
H	0.499441	6.737487	1.209546
H	-0.412212	6.656399	2.713649
H	2.724244	9.579239	2.247535
H	2.355703	8.465762	0.934337
H	0.119934	8.268722	4.616252
H	1.400502	9.462514	4.427694
H	0.297507	8.999986	2.237738
C	-1.218177	8.136832	8.919139
C	-1.013762	8.155628	7.396495
C	-1.583991	6.868776	6.780212
C	-0.860759	5.653247	7.380107
C	-1.064558	5.627815	8.902843
C	-0.496026	6.918015	9.513665
C	-3.084619	6.774535	7.096477
C	-2.719895	8.039761	9.229573
C	-2.566493	5.537212	9.213835
C	-3.294067	6.752340	8.618565
H	-0.810802	9.054914	9.356494
H	-1.507444	9.032751	6.960989
H	0.054029	8.243758	7.161956
H	-1.437673	6.884585	5.694566
H	-1.245369	4.728529	6.933086
H	0.209504	5.697698	7.144702
H	-0.547667	4.760775	9.328417
H	-0.617410	6.903719	10.603631
H	0.580831	6.984720	9.316411
H	-3.617039	7.626226	6.656038
H	-3.507784	5.868968	6.645130
H	-2.880810	8.046181	10.314498
H	-3.244896	8.914218	8.826515
H	-2.981149	4.610303	8.799448
H	-2.724529	5.497784	10.298471
H	-4.364777	6.685747	8.840814
C	-6.376392	4.048347	8.972371
C	-7.871725	3.941520	9.308750
C	-8.444311	2.642016	8.721905
C	-8.260609	2.648063	7.196565
C	-6.766244	2.751655	6.854440
C	-6.197844	4.050675	7.446399
C	-7.695722	1.440562	9.319329

C	-5.633330	2.843341	9.569670
C	-6.021746	1.550230	7.456964
C	-6.199804	1.541320	8.982894
H	-5.970248	4.975473	9.391411
H	-8.015454	3.959349	10.395836
H	-8.410594	4.806574	8.903465
H	-9.510346	2.567665	8.963416
H	-8.683747	1.733707	6.763248
H	-8.806751	3.490272	6.754674
H	-6.637717	2.756347	5.766549
H	-5.135231	4.146731	7.192246
H	-6.707170	4.917818	7.008644
H	-7.835467	1.411961	10.406752
H	-8.108547	0.505219	8.922415
H	-4.560574	2.918374	9.353891
H	-5.737172	2.840483	10.661442
H	-6.405153	0.616514	7.028021
H	-4.956128	1.601628	7.202444
H	-5.668807	0.683810	9.410640
C	5.586263	4.847733	8.181005
C	5.140671	3.413985	7.854819
C	3.774360	3.135458	8.500242
C	3.887035	3.303871	10.023196
C	4.329512	4.737345	10.355268
C	5.694213	5.011925	9.704772
C	2.740598	4.132147	7.953673
C	4.547834	5.839804	7.634736
C	3.294661	5.730067	9.803616
C	3.180404	5.567791	8.280180
H	6.560519	5.044141	7.720181
H	5.079153	3.276625	6.768504
H	5.883926	2.696635	8.223512
H	3.458381	2.112943	8.265921
H	2.922137	3.088001	10.497780
H	4.608029	2.584185	10.429489
H	4.409697	4.854944	11.441449
H	6.031155	6.026280	9.950780
H	6.447379	4.322745	10.105709
H	2.634674	4.008820	6.869050
H	1.755904	3.931152	8.392777
H	4.864824	6.868889	7.843298
H	4.474662	5.745916	6.544512
H	2.319382	5.557224	10.274545
H	3.588741	6.757108	10.051946
H	2.441801	6.275952	7.888743
C	-3.511807	10.724013	3.361138
C	-5.009262	10.542436	3.653100
C	-5.518279	9.260062	2.976805
C	-5.300165	9.363781	1.459533
C	-3.803423	9.542439	1.161622
C	-3.298687	10.823870	1.842887
C	-4.738208	8.053457	3.521216
C	-2.737120	9.513667	3.905076
C	-3.027557	8.335407	1.711021

C	-3.239935	8.229005	3.228855
H	-3.151023	11.638808	3.843935
H	-5.178348	10.490130	4.735415
H	-5.571366	11.409721	3.285938
H	-6.585935	9.132316	3.186754
H	-5.678089	8.461950	0.962811
H	-5.867828	10.210193	1.054315
H	-3.650366	9.616698	0.079445
H	-2.234990	10.973896	1.621511
H	-3.830615	11.696261	1.444184
H	-4.901548	7.954962	4.601241
H	-5.105858	7.128899	3.059849
H	-1.663363	9.640809	3.721015
H	-2.865675	9.441913	4.991853
H	-3.365149	7.415464	1.218524
H	-1.959144	8.441018	1.486732
H	-2.686496	7.367616	3.618680
C	10.193524	-1.064826	3.543596
C	9.707291	-2.484976	3.215978
C	8.332085	-2.723972	3.858352
C	8.446282	-2.559296	5.381601
C	8.929382	-1.139331	5.715098
C	10.302874	-0.904233	5.067647
C	7.328822	-1.697607	3.309873
C	9.185462	-0.042942	2.995406
C	7.924926	-0.116898	5.161533
C	7.809326	-0.275393	3.637800
H	11.174060	-0.896605	3.084946
H	9.644179	-2.620209	2.129488
H	10.428648	-3.223656	3.586044
H	7.987125	-3.736841	3.623013
H	7.474506	-2.747246	5.854025
H	9.145239	-3.299676	5.789218
H	9.010580	-1.024391	6.801489
H	10.668527	0.099869	5.314715
H	11.034884	-1.615050	5.469987
H	7.221726	-1.817526	2.224983
H	6.337767	-1.870104	3.746779
H	9.531679	0.976473	3.204990
H	9.111969	-0.134382	1.904995
H	6.944028	-0.261537	5.630294
H	8.248098	0.901126	5.410835
H	7.092402	0.453973	3.244997
C	8.634028	6.365166	4.239268
C	7.153283	6.261189	4.635712
C	6.552437	4.967623	4.064202
C	6.673763	4.983297	2.532705
C	8.153294	5.084080	2.130538
C	8.750163	6.377142	2.707326
C	7.320346	3.759630	4.622646
C	9.396395	5.153639	4.797790
C	8.917322	3.876095	2.694246
C	8.801650	3.857536	4.226082
H	9.060350	7.288061	4.647464

H	7.054101	6.272200	5.727854
H	6.601519	7.130784	4.258467
H	5.496878	4.895294	4.348543
H	6.229925	4.073325	2.111022
H	6.113153	5.830319	2.119032
H	8.237335	5.095646	1.038361
H	9.801867	6.471273	2.410636
H	8.226600	7.248943	2.296446
H	7.224988	3.724197	5.714657
H	6.888220	2.828392	4.236776
H	10.459722	5.226456	4.538888
H	9.337182	5.143797	5.892850
H	8.513290	2.946600	2.275183
H	9.971849	3.925562	2.396771
H	9.346494	2.995360	4.626141
C	3.531132	-1.823095	9.672350
C	3.724578	-1.830654	8.148177
C	5.223258	-1.769424	7.814765
C	5.823136	-0.482575	8.401805
C	5.635131	-0.471243	9.926742
C	4.135423	-0.536201	10.254772
C	5.931492	-2.986732	8.429058
C	4.243479	-3.040641	10.281522
C	6.341868	-1.692061	10.535725
C	5.743411	-2.981942	9.954088
H	2.462133	-1.866566	9.907849
H	3.279269	-2.735121	7.716469
H	3.204284	-0.975809	7.699305
H	5.358840	-1.775491	6.727628
H	6.889342	-0.417274	8.153217
H	5.339679	0.395562	7.956834
H	6.063028	0.447274	10.343011
H	3.986209	-0.508778	11.341026
H	3.622067	0.341215	9.842965
H	5.526981	-3.912942	8.003217
H	6.999708	-2.964637	8.181552
H	4.095513	-3.058114	11.368167
H	3.808401	-3.967197	9.887871
H	7.417273	-1.647954	10.325066
H	6.232315	-1.684766	11.627013
H	6.248860	-3.851209	10.388996
C	-4.758431	-3.393993	8.259173
C	-5.754678	-2.392588	7.654802
C	-7.143436	-2.601415	8.278257
C	-7.619187	-4.034165	7.993063
C	-6.627785	-5.040087	8.598169
C	-5.240252	-4.824768	7.974201
C	-7.056810	-2.387429	9.797302
C	-4.677844	-3.177415	9.778234
C	-6.542028	-4.819681	10.116326
C	-6.063643	-3.388968	10.406934
H	-3.769176	-3.243582	7.813235
H	-5.412101	-1.366178	7.833285
H	-5.807010	-2.524768	6.567262

H	-7.851951	-1.886059	7.846334
H	-8.619001	-4.189611	8.416224
H	-7.704459	-4.194531	6.911460
H	-6.968663	-6.060784	8.393164
H	-4.526816	-5.550358	8.383459
H	-5.283252	-4.999526	6.892263
H	-6.737837	-1.361135	10.015966
H	-8.046693	-2.514920	10.251956
H	-3.953828	-3.873231	10.219412
H	-4.316704	-2.164922	9.995480
H	-7.522756	-4.989289	10.577005
H	-5.852164	-5.545607	10.563633
H	-6.003630	-3.233506	11.489745
C	-4.133791	-8.251425	-1.634673
C	-2.735873	-7.993490	-2.217663
C	-2.304627	-6.550453	-1.913319
C	-3.311060	-5.574987	-2.542609
C	-4.710681	-5.826561	-1.960650
C	-5.135618	-7.271567	-2.264513
C	-2.274414	-6.338410	-0.391835
C	-4.098431	-8.035344	-0.113816
C	-4.674364	-5.616856	-0.438994
C	-3.671384	-6.592317	0.195633
H	-4.439389	-9.280543	-1.852926
H	-2.013737	-8.700303	-1.791439
H	-2.743708	-8.162988	-3.301250
H	-1.307509	-6.368955	-2.329374
H	-3.003101	-4.540277	-2.349233
H	-3.328339	-5.702130	-3.631825
H	-5.426600	-5.130419	-2.411089
H	-6.142314	-7.458430	-1.871308
H	-5.185621	-7.428705	-3.348850
H	-1.544199	-7.014790	0.068744
H	-1.949106	-5.316774	-0.161040
H	-5.086526	-8.236540	0.317676
H	-3.400289	-8.742164	0.350686
H	-4.390767	-4.582806	-0.208488
H	-5.673026	-5.773751	-0.013526
H	-3.646455	-6.440981	1.280403
C	1.037097	-4.128679	-1.686712
C	2.419245	-3.858214	-2.300797
C	2.852139	-2.416927	-1.990558
C	1.826711	-1.438620	-2.583734
C	0.442810	-1.702720	-1.970565
C	0.016243	-3.145954	-2.280501
C	2.918879	-2.222102	-0.467935
C	1.108951	-3.929787	-0.164797
C	0.515666	-1.510207	-0.447947
C	1.537773	-2.488586	0.150649
H	0.730294	-5.156527	-1.909218
H	3.154416	-4.566778	-1.900555
H	2.385464	-4.015391	-3.385742
H	3.838012	-2.226490	-2.428820
H	2.135241	-0.404887	-2.386090

H	1.783183	-1.553421	-3.673588
H	-0.286698	-1.004524	-2.395289
H	-0.979746	-3.341536	-1.864907
H	-0.059760	-3.290941	-3.365020
H	3.662850	-2.900592	-0.033200
H	3.245723	-1.201794	-0.233462
H	0.132555	-4.140059	0.288450
H	1.821076	-4.638894	0.274297
H	0.800757	-0.477661	-0.212598
H	-0.471617	-1.676158	0.000046
H	1.588742	-2.349510	1.236149
C	8.007872	-3.608750	-0.356613
C	6.517516	-3.725381	-0.001901
C	5.942649	-5.021186	-0.594668
C	6.106366	-4.998637	-2.122128
C	7.595691	-4.885185	-2.482606
C	8.166442	-3.589936	-1.884676
C	6.703914	-6.225546	-0.019531
C	8.763623	-4.816686	0.218434
C	8.352932	-6.089588	-1.902316
C	8.194879	-6.114981	-0.374370
H	8.415648	-2.684278	0.066683
H	6.387940	-3.719369	1.087092
H	5.969846	-2.858491	-0.391144
H	4.880177	-5.102544	-0.340069
H	5.681358	-5.910266	-2.559345
H	5.551037	-4.154243	-2.548114
H	7.709963	-4.868736	-3.571973
H	9.225237	-3.486759	-2.151646
H	7.647878	-2.720496	-2.306748
H	6.578517	-6.265947	1.069266
H	6.289780	-7.158492	-0.420702
H	9.833149	-4.734881	-0.010517
H	8.674077	-4.831237	1.311379
H	7.967788	-7.020451	-2.335878
H	9.414911	-6.031049	-2.170155
H	8.734941	-6.974594	0.037512
C	8.007250	1.738198	-2.043135
C	7.583772	0.297324	-2.367375
C	6.208792	0.007939	-1.745570
C	6.291064	0.188106	-0.222015
C	6.711322	1.628712	0.108053
C	8.084830	1.914096	-0.518826
C	5.174451	0.989171	-2.318382
C	6.968290	2.714761	-2.615701
C	5.676031	2.605929	-0.469833
C	5.592118	2.431879	-1.993943
H	8.987723	1.942344	-2.487101
H	7.544126	0.151794	-3.453644
H	8.327906	-0.409080	-1.979803
H	5.908616	-1.019649	-1.978470
H	5.319905	-0.035301	0.236021
H	7.012265	-0.520624	0.202750
H	6.769851	1.754692	1.194677

H	8.405837	2.933841	-0.273793
H	8.837956	1.236193	-0.099032
H	5.090223	0.857186	-3.403887
H	4.184000	0.780172	-1.896293
H	7.269886	3.748753	-2.408583
H	6.916583	2.612541	-3.706405
H	4.694084	2.425405	-0.015950
H	5.954002	3.637895	-0.223368
H	4.853162	3.128990	-2.404102
C	-6.757418	-8.451524	4.456982
C	-8.251768	-8.599637	4.781890
C	-9.037207	-7.437849	4.153967
C	-8.518227	-6.107480	4.720736
C	-7.024435	-5.952725	4.395865
C	-6.244184	-7.118522	5.022820
C	-8.838177	-7.453532	2.630507
C	-6.564544	-8.465074	2.932658
C	-6.830348	-5.972651	2.871835
C	-7.345085	-7.302576	2.300354
H	-6.199324	-9.280556	4.905697
H	-8.626893	-9.557255	4.401126
H	-8.400614	-8.610282	5.868482
H	-10.102388	-7.545785	4.386398
H	-9.085011	-5.270692	4.294826
H	-8.672315	-6.074699	5.806110
H	-6.656489	-5.003902	4.801327
H	-5.172815	-7.009337	4.814941
H	-6.357640	-7.103454	6.113636
H	-9.223556	-8.390045	2.209480
H	-9.409951	-6.639738	2.168287
H	-5.498781	-8.380595	2.687690
H	-6.909946	-9.419806	2.517996
H	-7.367085	-5.133310	2.413318
H	-5.769451	-5.842501	2.625821
H	-7.206389	-7.314742	1.213677
C	5.017918	-5.166688	3.598733
C	4.547309	-6.597695	3.296527
C	3.169434	-6.836415	3.933259
C	3.270335	-6.639715	5.453645
C	3.737768	-5.208803	5.761709
C	5.114004	-4.974098	5.119967
C	2.161048	-5.830849	3.356385
C	4.004772	-4.165641	3.022203
C	2.728264	-4.207282	5.179822
C	2.625907	-4.397826	3.658815
H	6.000383	-4.998675	3.144150
H	4.493843	-6.755594	2.212580
H	5.272545	-7.321914	3.687029
H	2.835630	-7.857045	3.716058
H	2.296706	-6.827089	5.922468
H	3.972898	-7.365079	5.881556
H	3.809506	-5.071024	6.846107
H	5.468490	-3.961802	5.349229
H	5.849387	-5.669706	5.542248

H	2.063448	-5.973817	2.273394
H	1.168272	-6.003678	3.789237
H	4.339972	-3.138980	3.213531
H	3.940553	-4.279936	1.933364
H	1.745143	-4.351516	5.644028
H	3.040126	-3.181418	5.410713
H	1.905365	-3.683340	3.245804
C	2.839672	-7.747758	-0.226182
C	1.344531	-7.873958	0.104286
C	0.792567	-9.183714	-0.479520
C	0.982736	-9.181435	-2.004078
C	2.476979	-9.058506	-2.340282
C	3.024742	-7.749272	-1.751378
C	1.555176	-10.372164	0.126124
C	3.596805	-8.939838	0.379328
C	3.235475	-10.246964	-1.729556
C	3.050988	-10.252024	-0.204372
H	3.231102	-6.813350	0.190680
H	1.195887	-7.853625	1.190667
H	0.795460	-7.018163	-0.306917
H	-0.273365	-9.271869	-0.242231
H	0.574197	-10.103370	-2.435342
H	0.426866	-8.348734	-2.451821
H	2.610112	-9.056537	-3.427627
H	4.087004	-7.639514	-2.001414
H	2.505321	-6.891112	-2.194934
H	1.411160	-10.398198	1.213054
H	1.157085	-11.314790	-0.268606
H	4.669337	-8.850800	0.167796
H	3.488312	-8.939615	1.470652
H	2.866914	-11.187686	-2.156196
H	4.301364	-10.181818	-1.979760
H	3.591987	-11.100294	0.229227
C	0.410994	0.732766	8.223617
C	-0.613633	1.722215	7.647672
C	-1.988033	1.479106	8.290228
C	-2.440965	0.041101	7.994520
C	-1.421084	-0.952966	8.571218
C	-0.048041	-0.703383	7.928250
C	-1.881228	1.676012	9.810297
C	0.511712	0.932147	9.743830
C	-1.315284	-0.749648	10.090495
C	-0.859593	0.686304	10.391548
H	1.389960	0.907620	7.764093
H	-0.287817	2.752740	7.833665
H	-0.680814	1.602444	6.559511
H	-2.716826	2.185985	7.878562
H	-3.430756	-0.138708	8.431372
H	-2.540446	-0.107554	6.912457
H	-1.745734	-1.977422	8.358754
H	0.685505	-1.420120	8.317049
H	-0.104993	-0.865611	6.845007
H	-1.578365	2.705473	10.036833
H	-2.861119	1.523953	10.278834

H	1.255778	0.244987	10.164751
H	0.856971	1.948638	9.968110
H	-2.285125	-0.943711	10.564379
H	-0.604689	-1.467641	10.517701
H	-0.785252	0.829557	11.475154
C	-6.947919	-0.688385	2.638322
C	-7.399056	-2.132854	2.372672
C	-8.784655	-2.366620	2.994302
C	-8.710567	-2.122560	4.509357
C	-8.262701	-0.678041	4.780813
C	-6.878618	-0.448458	4.154273
C	-9.793601	-1.389866	2.370787
C	-7.961584	0.283812	2.015214
C	-9.272642	0.294505	4.152394
C	-9.348238	0.056563	2.636507
H	-5.959916	-0.523921	2.194575
H	-7.433231	-2.324312	1.293364
H	-6.673010	-2.837095	2.796764
H	-9.104544	-3.396882	2.803205
H	-9.689773	-2.305847	4.968070
H	-8.007788	-2.827044	4.970508
H	-8.210062	-0.506484	5.861482
H	-6.538164	0.574062	4.358011
H	-6.143234	-1.123026	4.609410
H	-9.872068	-1.566840	1.291269
H	-10.791510	-1.559916	2.792799
H	-7.639939	1.319327	2.180290
H	-8.006871	0.135671	0.929506
H	-10.261699	0.154094	4.605018
H	-8.974967	1.330183	4.356661
H	-10.069125	0.750439	2.190293
C	-3.999970	0.819190	-1.240843
C	-5.497574	0.671340	-0.931155
C	-6.033638	-0.621575	-1.565157
C	-5.827279	-0.565980	-3.086595
C	-4.330489	-0.421209	-3.402137
C	-3.798676	0.870981	-2.763103
C	-5.268788	-1.824623	-0.992034
C	-3.240568	-0.387519	-0.668036
C	-3.569806	-1.624467	-2.823979
C	-3.770469	-1.682824	-1.301949
H	-3.619887	1.741555	-0.788195
H	-5.657914	0.653576	0.153607
H	-6.048523	1.536798	-1.318955
H	-7.101356	-0.725224	-1.342622
H	-6.224408	-1.475508	-3.553398
H	-6.384468	0.277596	-3.511772
H	-4.185811	-0.381232	-4.487272
H	-2.734650	0.996629	-2.997682
H	-4.319650	1.740114	-3.182832
H	-5.424166	-1.888713	0.091750
H	-5.655707	-2.756002	-1.422915
H	-2.166531	-0.283820	-0.864729
H	-3.360667	-0.425269	0.421418

H	-3.926871	-2.552649	-3.286364
H	-2.501823	-1.543374	-3.060204
H	-3.227885	-2.541581	-0.891598
C	0.791861	3.728991	-3.449682
C	1.325095	5.020854	-2.811453
C	1.140312	4.961521	-1.287219
C	-0.353051	4.806546	-0.961186
C	-0.890929	3.514146	-1.594665
C	-0.700590	3.576994	-3.118071
C	1.910615	3.755556	-0.727690
C	1.563570	2.526409	-2.884697
C	-0.115217	2.311838	-1.034789
C	1.378703	2.460481	-1.360908
H	0.924328	3.773441	-4.536229
H	2.385680	5.152588	-3.057496
H	0.794409	5.888934	-3.221041
H	1.521669	5.883293	-0.834240
H	-0.500231	4.783054	0.125388
H	-0.912652	5.671201	-1.338111
H	-1.955797	3.406019	-1.361400
H	-1.098885	2.667961	-3.584960
H	-1.266682	4.419661	-3.533319
H	2.982118	3.863156	-0.935392
H	1.802243	3.713355	0.362864
H	1.206012	1.598795	-3.348033
H	2.628707	2.613199	-3.131090
H	-0.258445	2.244130	0.050439
H	-0.503576	1.380337	-1.464262
H	1.930193	1.602696	-0.960518
C	-1.766722	3.402403	2.611419
C	-2.205361	1.959856	2.316058
C	-3.589786	1.702034	2.930751
C	-3.519794	1.916136	4.450524
C	-3.084434	3.358588	4.751686
C	-1.701482	3.612313	4.131997
C	-4.606053	2.682677	2.325497
C	-2.787669	4.378443	2.006472
C	-4.101652	4.335115	4.141442
C	-4.173203	4.127134	2.620959
H	-0.779536	3.584030	2.172574
H	-2.236471	1.789899	1.233063
H	-1.474003	1.253312	2.726988
H	-3.900760	0.673191	2.718466
H	-4.498045	1.715462	4.903967
H	-2.811750	1.208407	4.898464
H	-3.034694	3.508775	5.835669
H	-1.369897	4.633334	4.356796
H	-0.961078	2.934889	4.574576
H	-4.681575	2.526836	1.242517
H	-5.603068	2.495821	2.742484
H	-2.474948	5.413076	2.192851
H	-2.830245	4.251830	0.917934
H	-5.090106	4.177347	4.589646
H	-3.812955	5.368922	4.366970

H	-4.899286	4.823805	2.187711
C	-2.141962	-3.143965	2.239923
C	-1.579779	-1.851151	2.850797
C	-1.772331	-1.870705	4.375098
C	-3.270403	-1.981474	4.697522
C	-3.837262	-3.274601	4.091388
C	-3.639072	-3.251652	2.567859
C	-1.034273	-3.081928	4.965860
C	-1.402438	-4.351716	2.836069
C	-3.093708	-4.482294	4.682438
C	-1.595234	-4.377869	4.360077
H	-2.003903	-3.127892	1.153281
H	-0.515256	-1.751159	2.606823
H	-2.087233	-0.980028	2.418841
H	-1.370309	-0.948332	4.808564
H	-3.422655	-1.976241	5.783639
H	-3.806985	-1.112309	4.298163
H	-4.905433	-3.351191	4.322023
H	-4.057656	-4.161200	2.120114
H	-4.182524	-2.405190	2.130661
H	0.040411	-3.005437	4.760824
H	-1.148205	-3.096252	6.056570
H	-1.780769	-5.280751	2.392496
H	-0.334468	-4.296763	2.592800
H	-3.243090	-4.521380	5.768244
H	-3.503068	-5.413677	4.272663
H	-1.066690	-5.239427	4.782698
C	3.397830	2.237281	4.324953
C	1.899962	2.130918	4.650011
C	1.328253	0.838666	4.046531
C	1.522456	0.859197	2.522617
C	3.019386	0.962385	2.191775
C	3.586873	2.254112	2.800336
C	2.069778	-0.370458	4.637250
C	4.133820	1.024619	4.915474
C	3.756786	-0.246751	2.787566
C	3.568217	-0.270164	4.312079
H	3.803346	3.159235	4.755845
H	1.748804	2.138455	5.736209
H	1.366008	3.001250	4.249553
H	0.260403	0.764629	4.279988
H	1.100075	-0.049805	2.077458
H	0.981424	1.707063	2.085282
H	3.155403	0.977412	1.104888
H	4.651440	2.349985	2.554427
H	3.082690	3.126778	2.367633
H	1.922489	-0.409361	5.723357
H	1.657409	-1.300829	4.228355
H	5.208215	1.099071	4.707799
H	4.022466	1.011325	6.006429
H	3.374058	-1.175254	2.346863
H	4.824252	-0.195536	2.540871
H	4.094163	-1.133154	4.735018

Adamantane$_{26}$

```
       676
E=-1144.9100
C     -1.628998     -5.916763      9.754500
C     -1.436953     -5.929400      8.230183
C      0.061454     -5.870076      7.895204
C      0.662569     -4.581647      8.477494
C      0.475966     -4.565238     10.002557
C     -1.023476     -4.628307     10.332167
C      0.769589     -7.085760      8.512817
C     -0.916756     -7.132705     10.366989
C      1.182598     -5.784449     10.614873
C      0.582907     -7.075893     10.037994
H     -2.697805     -5.958881      9.991116
H     -1.883148     -6.835026      7.801835
H     -1.957193     -5.075739      7.779000
H      0.196038     -5.879764      6.807969
H      1.728582     -4.517740      8.227719
H      0.179182     -3.704702      8.030102
H      0.904743     -3.645602     10.415435
H     -1.171680     -4.597259     11.418462
H     -1.536731     -3.751958      9.917969
H      0.364185     -8.013134      8.090370
H      1.837590     -7.065057      8.264264
H     -1.063737     -7.146552     11.453820
H     -1.352698     -8.060301      9.976761
H      2.257833     -5.741617     10.403088
H      1.074048     -5.773535     11.706231
H      1.088282     -7.944013     10.475274
C     -2.026922    -10.945322      4.031822
C     -0.645802    -10.723104      3.396439
C     -0.170620     -9.288805      3.675614
C     -1.176805     -8.293736      3.077384
C     -2.559518     -8.509562      3.711759
C     -3.028424     -9.945974      3.432845
C     -0.079677     -9.069136      5.193595
C     -1.930767    -10.721740      5.548961
C     -2.462523     -8.292370      5.229703
C     -1.459592     -9.287324      5.833393
H     -2.363899    -11.968262      3.831446
H      0.075068    -11.444120      3.800438
H     -0.697326    -10.898346      2.314964
H      0.814450     -9.132784      3.222205
H     -0.838254     -7.265399      3.252778
H     -1.236997     -8.426447      1.990361
H     -3.275310     -7.799492      3.283401
H     -4.024027    -10.107261      3.863805
H     -3.121820    -10.107884      2.352085
H      0.651225     -9.759725      5.631436
H      0.277280     -8.054064      5.405830
H     -2.906709    -10.897577      6.017592
H     -1.232463    -11.441995      5.992089
H     -2.146963     -7.263923      5.443096
H     -3.448226     -8.423708      5.692419
```

H	-1.391365	-9.130629	6.915539
C	3.436216	7.553147	2.594650
C	2.987625	6.124025	2.252655
C	1.607363	5.851351	2.870296
C	1.691823	6.011431	4.395973
C	2.137186	7.440270	4.743791
C	3.515909	7.709032	4.121027
C	0.590836	6.857578	2.309074
C	2.414987	8.554785	2.033615
C	1.119642	8.442563	4.177447
C	1.033648	8.288634	2.651289
H	4.420417	7.745384	2.153625
H	2.946115	5.992485	1.164673
H	3.718950	5.399938	2.631884
H	1.289278	4.832132	2.624735
H	0.716587	5.799601	4.850835
H	2.400154	5.284968	4.812355
H	2.197232	7.551927	5.831898
H	3.854675	8.719894	4.378633
H	4.256707	7.012888	4.532828
H	0.504989	6.740358	1.222002
H	-0.403413	6.660932	2.728160
H	2.734622	9.580696	2.253475
H	2.362185	8.466813	0.941722
H	0.134377	8.273846	4.628675
H	1.415593	9.466384	4.436616
H	0.307352	9.003614	2.249362
C	-1.240592	8.147489	8.899966
C	-1.030407	8.155564	7.378013
C	-1.599162	6.864824	6.768537
C	-0.879016	5.653032	7.379602
C	-1.088589	5.638322	8.901696
C	-0.521509	6.932373	9.505681
C	-3.101038	6.773747	7.079766
C	-2.743537	8.053543	9.205379
C	-2.591749	5.550848	9.207621
C	-3.316255	6.762274	8.601167
H	-0.834260	9.068329	9.332467
H	-1.521852	9.029954	6.934554
H	0.038322	8.241375	7.146912
H	-1.448729	6.872988	5.683369
H	-1.262551	4.725474	6.937568
H	0.192159	5.695157	7.147947
H	-0.573891	4.773930	9.335241
H	-0.647023	6.925736	10.595253
H	0.556131	6.997012	9.312047
H	-3.631219	7.622697	6.631404
H	-3.523098	5.865336	6.633127
H	-2.908549	8.067612	10.289616
H	-3.266426	8.925514	8.794267
H	-3.005455	4.621346	8.798121
H	-2.753912	5.519065	10.291899
H	-4.387842	6.697916	8.819819
C	-6.357944	4.043430	8.973728

C	-7.860059	3.957465	9.284924
C	-8.444349	2.674678	8.673354
C	-8.235994	2.695724	7.151318
C	-6.734807	2.778539	6.834323
C	-6.154793	4.060850	7.450933
C	-7.725305	1.453980	9.268050
C	-5.644483	2.819272	9.568167
C	-6.019935	1.557877	7.434053
C	-6.222703	1.533868	8.956733
H	-5.943455	4.958663	9.410370
H	-8.020992	3.964811	10.369718
H	-8.378056	4.836078	8.881601
H	-9.515217	2.615179	8.896933
H	-8.667032	1.793708	6.700286
H	-8.761047	3.552052	6.711004
H	-6.588691	2.793969	5.748754
H	-5.086786	4.142256	7.214923
H	-6.642696	4.941447	7.015642
H	-7.883021	1.414837	10.352683
H	-8.146965	0.530379	8.853323
H	-4.567299	2.879038	9.370430
H	-5.765953	2.805220	10.658032
H	-6.411641	0.635795	6.987792
H	-4.949650	1.594589	7.197182
H	-5.712815	0.662661	9.382480
C	5.574199	4.853751	8.188193
C	5.132072	3.418733	7.862874
C	3.765433	3.138017	8.506650
C	3.875593	3.308252	10.029586
C	4.314597	4.742995	10.360787
C	5.679642	5.019755	9.711939
C	2.730363	4.131969	7.957577
C	4.534472	5.843075	7.639418
C	3.278454	5.732972	9.806633
C	3.166699	5.568871	8.283207
H	6.548695	5.051719	7.728545
H	5.072383	3.280114	6.776617
H	5.876303	2.703327	8.233368
H	3.451928	2.114599	8.272950
H	2.910477	3.090855	10.503027
H	4.597515	2.590502	10.437652
H	4.392994	4.861893	11.446957
H	6.014110	6.035069	9.957364
H	6.433679	4.332573	10.114662
H	2.626237	4.007291	6.872934
H	1.745470	3.929369	8.395494
H	4.849010	6.873038	7.847353
H	4.463043	5.747898	6.549189
H	2.302871	5.558577	10.276359
H	3.570031	6.760885	10.054307
H	2.427171	6.275075	7.889984
C	-3.507934	10.713043	3.331273
C	-5.015506	10.543821	3.574531
C	-5.513932	9.268501	2.877334

C	-5.246494	9.375735	1.368220
C	-3.739470	9.542066	1.118982
C	-3.245489	10.816464	1.821006
C	-4.762356	8.053008	3.441692
C	-2.761756	9.493860	3.894972
C	-2.992259	8.326166	1.688190
C	-3.254007	8.216196	3.198038
H	-3.154714	11.622826	3.828907
H	-5.219578	10.489127	4.650680
H	-5.557900	11.417419	3.192974
H	-6.588843	9.149556	3.052564
H	-5.616283	8.479119	0.856173
H	-5.793411	10.228648	0.948368
H	-3.551229	9.618855	0.042545
H	-2.173973	10.957763	1.634316
H	-3.756670	11.695012	1.408908
H	-4.961020	7.952084	4.515556
H	-5.123210	7.133447	2.965221
H	-1.681581	9.612051	3.745810
H	-2.925634	9.419340	4.976799
H	-3.322035	7.411063	1.181582
H	-1.916329	8.423021	1.498565
H	-2.720946	7.348488	3.601992
C	-8.910107	5.502167	1.217458
C	-8.384421	6.766028	1.915235
C	-8.603388	6.650950	3.431732
C	-10.104431	6.493004	3.718735
C	-10.634868	5.228457	3.025581
C	-10.410346	5.346983	1.510164
C	-7.852081	5.420266	3.962531
C	-8.157497	4.274652	1.753865
C	-9.878142	4.001382	3.556982
C	-8.376573	4.153001	3.269669
H	-8.753260	5.586348	0.136522
H	-7.317944	6.900398	1.697737
H	-8.901287	7.652010	1.526858
H	-8.227364	7.552827	3.927223
H	-10.275877	6.429854	4.800158
H	-10.650951	7.374521	3.362225
H	-11.705218	5.118180	3.231102
H	-10.802927	4.458458	1.000881
H	-10.962617	6.207997	1.114400
H	-6.775670	5.529022	3.782675
H	-7.984915	5.338036	5.048084
H	-8.509530	3.367178	1.248333
H	-7.086699	4.364094	1.534434
H	-10.045839	3.894074	4.635491
H	-10.261741	3.088948	3.084457
H	-7.838649	3.277522	3.649700
C	10.197542	-1.073980	3.542596
C	9.708794	-2.492751	3.212750
C	8.333113	-2.730276	3.854652
C	8.447488	-2.568156	5.378162
C	8.933102	-1.149575	5.713887

C	10.307063	-0.915936	5.066901
C	7.331731	-1.701272	3.307684
C	9.191353	-0.049449	2.995910
C	7.930521	-0.124492	5.161826
C	7.814754	-0.280428	3.637842
H	11.178413	-0.906807	3.084279
H	9.645524	-2.626193	2.126048
H	10.428799	-3.233291	3.581729
H	7.986359	-3.742162	3.617724
H	7.475341	-2.755096	5.850224
H	9.145087	-3.310414	5.784688
H	9.014422	-1.036457	6.800460
H	10.674493	0.087127	5.315546
H	11.037769	-1.628683	5.468198
H	7.224504	-1.819324	2.222602
H	6.340335	-1.872669	3.744250
H	9.539378	0.969019	3.207092
H	9.117780	-0.139075	1.905353
H	6.949329	-0.268099	5.630290
H	8.255495	0.892566	5.412723
H	7.099166	0.450825	3.246112
C	8.643538	6.356760	4.249641
C	7.162373	6.257392	4.645699
C	6.557604	4.965799	4.073863
C	6.679364	4.981295	2.542399
C	8.159306	5.077476	2.140618
C	8.760097	6.368575	2.717730
C	7.321566	3.755321	4.632339
C	9.401948	5.142766	4.808192
C	8.919387	3.867017	2.704356
C	8.803271	3.848617	4.236160
H	9.072659	7.278254	4.658068
H	7.062951	6.268569	5.737817
H	6.613444	7.128769	4.268431
H	5.501751	4.896754	4.357930
H	6.232771	4.072781	2.120482
H	6.121527	5.830132	2.128698
H	8.243659	5.088922	1.048463
H	9.812166	6.459435	2.421317
H	8.239383	7.242075	2.306835
H	7.225822	3.720042	5.724321
H	6.886610	2.825498	4.246235
H	10.465564	5.212271	4.549567
H	9.342428	5.132964	5.903235
H	8.512537	2.938853	2.285067
H	9.974139	3.913204	2.407153
H	9.345298	2.984677	4.636241
C	3.532539	-1.822685	9.670708
C	3.726537	-1.827730	8.146595
C	5.225348	-1.766181	7.813830
C	5.825216	-0.480410	8.403236
C	5.636659	-0.471594	9.928121
C	4.136822	-0.536862	10.255497
C	5.933166	-2.984626	8.426347

C	4.244472	-3.041360	10.278105
C	6.342982	-1.693539	10.535321
C	5.744532	-2.982352	9.951313
H	2.463447	-1.866379	9.905747
H	3.281242	-2.731404	7.713216
H	3.206541	-0.972054	7.698962
H	5.361324	-1.770455	6.726734
H	6.891523	-0.414864	8.155144
H	5.342060	0.398546	7.959556
H	6.064550	0.446158	10.346079
H	3.987217	-0.511229	11.341742
H	3.623755	0.341323	9.844970
H	5.528663	-3.910060	7.998813
H	7.001476	-2.962288	8.179265
H	4.096108	-3.060624	11.364665
H	3.809390	-3.967188	9.882749
H	7.418470	-1.649252	10.325127
H	6.233033	-1.688050	11.626580
H	6.249685	-3.852425	10.384953
C	-4.766196	-3.385287	8.262097
C	-5.770535	-2.391903	7.657881
C	-7.156716	-2.609578	8.284038
C	-7.622797	-4.046103	8.001883
C	-6.623270	-5.044036	8.606848
C	-5.238361	-4.819879	7.980170
C	-7.069021	-2.392610	9.802598
C	-4.684559	-3.165769	9.780680
C	-6.536491	-4.820656	10.124513
C	-6.067752	-3.386141	10.412081
H	-3.778792	-3.228576	7.814231
H	-5.434930	-1.362817	7.834184
H	-5.823782	-2.526150	6.570639
H	-7.871013	-1.899929	7.852221
H	-8.620764	-4.207958	8.426997
H	-7.708774	-4.208759	6.920677
H	-6.957262	-6.067440	8.404012
H	-4.519110	-5.539762	8.389330
H	-5.281964	-4.996629	6.898580
H	-6.756951	-1.363744	10.019123
H	-8.057201	-2.526391	10.259146
H	-3.954884	-3.855753	10.221695
H	-4.330226	-2.150408	9.995735
H	-7.515207	-4.996479	10.587134
H	-5.840744	-5.540982	10.571762
H	-6.006998	-3.228566	11.494545
C	-4.141871	-8.239694	-1.635189
C	-2.746256	-7.978109	-2.222060
C	-2.316292	-6.535153	-1.915528
C	-3.326289	-5.559717	-2.539129
C	-4.723620	-5.814942	-1.953268
C	-5.147271	-7.259858	-2.259345
C	-2.281479	-6.326838	-0.393627
C	-4.101919	-8.027332	-0.113922
C	-4.682699	-5.608956	-0.431220

C	-3.676136	-6.584405	0.197729
H	-4.446559	-9.268749	-1.855010
H	-2.021634	-8.684837	-1.799935
H	-2.757339	-8.144930	-3.306034
H	-1.320814	-6.351051	-2.334357
H	-3.019330	-4.525006	-2.344178
H	-3.346900	-5.684185	-3.628596
H	-5.442087	-5.118815	-2.399654
H	-6.152393	-7.449284	-1.863347
H	-5.200543	-7.414384	-3.343901
H	-1.548714	-7.003206	0.062902
H	-1.957029	-5.305265	-0.161347
H	-5.088293	-8.231157	0.320265
H	-3.401166	-8.734200	0.346557
H	-4.399980	-4.575035	-0.199064
H	-5.679729	-5.768481	-0.002912
H	-3.647929	-6.435721	1.282785
C	1.028230	-4.120177	-1.688076
C	2.410078	-3.848678	-2.302379
C	2.842545	-2.407499	-1.991043
C	1.816564	-1.429059	-2.583045
C	0.432961	-1.694193	-1.969651
C	0.006822	-3.137318	-2.280688
C	2.909711	-2.213957	-0.468276
C	1.100508	-3.922566	-0.166014
C	0.506243	-1.502962	-0.446893
C	1.528905	-2.481479	0.150530
H	0.721731	-5.147947	-1.911365
H	3.145640	-4.557315	-1.902986
H	2.376001	-4.004935	-3.387448
H	3.828205	-2.216324	-2.429464
H	2.124780	-0.395384	-2.384613
H	1.772723	-1.542940	-3.672983
H	-0.296942	-0.995901	-2.393537
H	-0.988960	-3.333622	-1.864937
H	-0.069482	-3.281401	-3.365306
H	3.654072	-2.892547	-0.034366
H	3.236257	-1.193731	-0.233033
H	0.124337	-4.133585	0.287371
H	1.813037	-4.631789	0.272238
H	0.791032	-0.470513	-0.210750
H	-0.480832	-1.669659	0.001280
H	1.580177	-2.343317	1.236132
C	8.001040	-3.606101	-0.360682
C	6.510559	-3.721458	-0.006079
C	5.934011	-5.015498	-0.601063
C	6.097662	-4.990498	-2.128493
C	7.587108	-4.878310	-2.488868
C	8.159539	-3.584829	-1.888721
C	6.693782	-6.221823	-0.028073
C	8.755292	-4.815994	0.212212
C	8.342855	-6.084680	-1.910724
C	8.184864	-6.112532	-0.382814
H	8.410016	-2.682885	0.064196

H	6.381058	-3.717176	1.082931
H	5.963966	-2.853196	-0.393777
H	4.871452	-5.095950	-0.346539
H	5.671470	-5.900824	-2.567269
H	5.543380	-4.144660	-2.552972
H	7.701333	-4.860109	-3.578212
H	9.218448	-3.482531	-2.155578
H	7.642054	-2.713998	-2.309246
H	6.568402	-6.263959	1.060660
H	6.278439	-7.153543	-0.430840
H	9.824908	-4.735149	-0.016664
H	8.665796	-4.832334	1.305136
H	7.956503	-7.014298	-2.345881
H	9.404892	-6.027023	-2.178528
H	8.723860	-6.973546	0.027537
C	8.008688	1.741791	-2.039496
C	7.583629	0.302212	-2.367398
C	6.207606	0.013282	-1.747694
C	6.288504	0.189939	-0.223655
C	6.710337	1.629241	0.110073
C	8.084888	1.914184	-0.514716
C	5.175184	0.997173	-2.319407
C	6.971641	2.721019	-2.610977
C	5.676965	2.609131	-0.466724
C	5.594434	2.438593	-1.991306
H	8.989903	1.945615	-2.481969
H	7.544939	0.159159	-3.454031
H	8.326407	-0.406050	-1.980619
H	5.906303	-1.013382	-1.983204
H	5.316563	-0.033189	0.232856
H	7.008307	-0.520702	0.200289
H	6.767883	1.752718	1.197036
H	8.406998	2.932949	-0.267069
H	8.836663	1.234339	-0.095642
H	5.091929	0.867723	-3.405292
H	4.184008	0.788559	-1.898831
H	7.274399	3.754142	-2.401234
H	6.920953	2.621302	-3.701961
H	4.694298	2.428908	-0.014282
H	5.956054	3.640170	-0.217662
H	4.856846	3.137606	-2.400689
C	-6.749809	-8.464521	4.466122
C	-8.243184	-8.615509	4.794176
C	-9.032499	-7.456581	4.165827
C	-8.515476	-6.124075	4.729352
C	-7.022674	-5.966446	4.401325
C	-6.238539	-7.129395	5.028724
C	-8.836383	-7.474375	2.642012
C	-6.559856	-8.480197	2.941452
C	-6.831492	-5.988494	2.876957
C	-7.344283	-7.320556	2.308710
H	-6.188947	-9.291516	4.915146
H	-8.616848	-9.574623	4.415749
H	-8.389902	-8.624664	5.881071

H	-10.096978	-7.566565	4.400504
H	-9.085001	-5.289306	4.303137
H	-8.667537	-6.089818	5.814967
H	-6.656122	-5.016102	4.804479
H	-5.167828	-7.018108	4.818584
H	-6.349918	-7.112750	6.119731
H	-9.220427	-8.412475	2.223307
H	-9.410915	-6.662672	2.179535
H	-5.494765	-8.393689	2.694276
H	-6.903868	-9.436414	2.529065
H	-7.371040	-5.151158	2.418073
H	-5.771375	-5.856330	2.628668
H	-7.207663	-7.334234	1.221789
C	5.016216	-5.168256	3.595395
C	4.544817	-6.599185	3.294052
C	3.167264	-6.837090	3.931784
C	3.269248	-6.639845	5.452028
C	3.737474	-5.209007	5.759231
C	5.113384	-4.975119	5.116491
C	2.158912	-5.831331	3.355187
C	4.003103	-4.167013	3.019147
C	2.728000	-4.207295	5.177623
C	2.624562	-4.398383	3.656758
H	5.998450	-5.000824	3.140100
H	4.490572	-6.757480	2.210201
H	5.270011	-7.323553	3.684355
H	2.832896	-7.857666	3.715197
H	2.295851	-6.826637	5.921564
H	3.971794	-7.365335	5.879755
H	3.809984	-5.070840	6.843528
H	5.468439	-3.962880	5.345129
H	5.848758	-5.670867	5.538556
H	2.060539	-5.974676	2.272316
H	1.166351	-6.003582	3.788760
H	4.338853	-3.140417	3.209857
H	3.938119	-4.281701	1.930395
H	1.745126	-4.350943	5.642533
H	3.040438	-3.181471	5.407912
H	1.904043	-3.683758	3.243946
C	2.833769	-7.741770	-0.231983
C	1.338532	-7.867136	0.098371
C	0.784981	-9.174699	-0.488837
C	0.974929	-9.168578	-2.013413
C	2.469263	-9.046467	-2.349512
C	3.018613	-7.739435	-1.757206
C	1.546317	-10.365631	0.113525
C	3.589623	-8.936325	0.370237
C	3.226486	-10.237415	-1.742066
C	3.042216	-10.246326	-0.216874
H	3.226331	-6.808924	0.187306
H	1.190071	-7.849525	1.184824
H	0.790382	-7.009620	-0.310469
H	-0.281016	-9.262264	-0.251625
H	0.565271	-10.088893	-2.447069

H	0.419949	-8.334051	-2.458854
H	2.602239	-9.041755	-3.436868
H	4.080963	-7.630229	-2.007108
H	2.500111	-6.879501	-2.198397
H	1.402429	-10.394394	1.200404
H	1.147087	-11.306746	-0.283654
H	4.662225	-8.847953	0.158782
H	3.481290	-8.938883	1.461574
H	2.856784	-11.176575	-2.171154
H	4.292412	-10.172825	-1.992255
H	3.582306	-11.096367	0.214384
C	0.408706	0.735034	8.224025
C	-0.618131	1.725185	7.653246
C	-1.990846	1.478965	8.298213
C	-2.443130	0.041397	7.999406
C	-1.421033	-0.953378	8.570936
C	-0.049681	-0.700685	7.925579
C	-1.880775	1.671609	9.818596
C	0.512689	0.930147	9.744573
C	-1.311974	-0.754321	10.090547
C	-0.856917	0.681182	10.394688
H	1.386467	0.912108	7.762789
H	-0.292839	2.755473	7.841457
H	-0.687670	1.608473	6.564901
H	-2.721219	2.186349	7.890231
H	-3.431762	-0.140578	8.437982
H	-2.544929	-0.104245	6.917149
H	-1.745226	-1.977519	8.356269
H	0.685401	-1.417857	8.310656
H	-0.108942	-0.859859	6.842006
H	-1.578342	2.700696	10.047399
H	-2.859461	1.517303	10.288913
H	1.258337	0.242470	10.161833
H	0.857524	1.946309	9.970987
H	-2.280560	-0.950637	10.566068
H	-0.599755	-1.472880	10.514080
H	-0.780250	0.821394	11.478529
C	-6.947596	-0.711656	2.650783
C	-7.393500	-2.156166	2.376660
C	-8.781307	-2.396492	2.990817
C	-8.714903	-2.158950	4.507264
C	-8.272298	-0.714425	4.787179
C	-6.885972	-0.478265	4.168086
C	-9.790058	-1.419758	2.366956
C	-7.961055	0.260507	2.027286
C	-9.282008	0.258120	4.158390
C	-9.349933	0.026696	2.641131
H	-5.958010	-0.542512	2.212356
H	-7.422152	-2.342930	1.296368
H	-6.667477	-2.860284	2.800997
H	-9.097456	-3.426771	2.793685
H	-9.695710	-2.346959	4.960616
H	-8.012319	-2.863539	4.968549
H	-8.225132	-0.547518	5.868829

H	-6.549295	0.544273	4.377920
H	-6.150837	-1.152821	4.623641
H	-9.863041	-1.592158	1.286313
H	-10.789434	-1.594418	2.783578
H	-7.643043	1.296159	2.198430
H	-8.000909	0.117058	0.940735
H	-10.272756	0.112987	4.605797
H	-8.988149	1.293696	4.368613
H	-10.070666	0.720562	2.194651
C	-4.011422	0.838007	-1.228546
C	-5.506963	0.686849	-0.910597
C	-6.044465	-0.605479	-1.544580
C	-5.846967	-0.545923	-3.067043
C	-4.352267	-0.397834	-3.390849
C	-3.818986	0.893751	-2.751812
C	-5.274277	-1.808566	-0.978732
C	-3.246678	-0.368750	-0.662993
C	-3.586217	-1.601149	-2.819943
C	-3.778012	-1.663457	-1.296927
H	-3.630312	1.759948	-0.775900
H	-5.661024	0.666239	0.175023
H	-6.061611	1.552276	-1.293160
H	-7.110705	-0.711490	-1.316151
H	-6.245225	-1.455024	-3.533716
H	-6.408032	0.297699	-3.486998
H	-4.213909	-0.355036	-4.476700
H	-2.756545	1.021782	-2.992211
H	-4.343850	1.762979	-3.166467
H	-5.423302	-1.875491	0.105772
H	-5.662083	-2.739585	-1.409595
H	-2.173970	-0.262739	-0.865616
H	-3.360436	-0.409288	0.427042
H	-3.944356	-2.528844	-3.282473
H	-2.519752	-1.517661	-3.062117
H	-3.231610	-2.522251	-0.891750
C	0.793509	3.741632	-3.447044
C	1.326382	5.033002	-2.807518
C	1.143881	4.971037	-1.283114
C	-0.348849	4.814044	-0.955156
C	-0.886359	3.522124	-1.589926
C	-0.698304	3.587608	-3.113505
C	1.916204	3.764948	-0.726646
C	1.567242	2.538918	-2.885116
C	-0.108631	2.319697	-1.033113
C	1.384663	2.470359	-1.361168
H	0.924348	3.787959	-4.533711
H	2.386475	5.166193	-3.054893
H	0.794232	5.901206	-3.214938
H	1.524977	5.892462	-0.829210
H	-0.494422	4.788660	0.131592
H	-0.909864	5.678742	-1.329876
H	-1.950778	3.412556	-1.355285
H	-1.096371	2.678927	-3.581273
H	-1.265843	4.430373	-3.526574

H	2.987294	3.873955	-0.935735
H	1.809464	3.720888	0.363995
H	1.209936	1.611691	-3.349421
H	2.631932	2.627170	-3.132921
H	-0.250208	2.250103	0.052212
H	-0.496685	1.388497	-1.463516
H	1.937593	1.612485	-0.962960
C	-1.745764	3.388819	2.607536
C	-2.196981	1.951822	2.304175
C	-3.580710	1.700578	2.923144
C	-3.502457	1.903603	4.444034
C	-3.054495	3.340491	4.753210
C	-1.672293	3.587684	4.129223
C	-4.591865	2.693298	2.329122
C	-2.761635	4.376970	2.013810
C	-4.066656	4.329136	4.154174
C	-4.146423	4.132249	2.632622
H	-0.759095	3.565759	2.165626
H	-2.234115	1.789613	1.220181
H	-1.469414	1.236758	2.707016
H	-3.900661	0.675691	2.705144
H	-4.480279	1.707446	4.900370
H	-2.798053	1.187280	4.883979
H	-2.998882	3.482778	5.837973
H	-1.331738	4.604502	4.359592
H	-0.935315	2.901448	4.563874
H	-4.673299	2.545550	1.245436
H	-5.588500	2.511356	2.749181
H	-2.440004	5.407814	2.205936
H	-2.809919	4.258231	0.924623
H	-5.054366	4.175999	4.605612
H	-3.768885	5.359069	4.385548
H	-4.868889	4.837554	2.207372
C	-2.147224	-3.148285	2.242766
C	-1.585487	-1.853648	2.850179
C	-1.774807	-1.870771	4.374914
C	-3.272048	-1.982969	4.700685
C	-3.838451	-3.277917	4.098022
C	-3.643496	-3.257389	2.574041
C	-1.033889	-3.079966	4.966251
C	-1.404834	-4.353996	2.839477
C	-3.092042	-4.483574	4.689630
C	-1.594390	-4.377719	4.363932
H	-2.011472	-3.133942	1.155809
H	-0.521613	-1.752667	2.603789
H	-2.095012	-0.983964	2.417760
H	-1.373108	-0.947100	4.805909
H	-3.422023	-1.976024	5.787109
H	-3.810630	-1.115226	4.300927
H	-4.906032	-3.355524	4.331040
H	-4.061804	-4.168282	2.128780
H	-4.188996	-2.412424	2.136499
H	0.040257	-3.002404	4.758819
H	-1.145508	-3.092519	6.057222

H	-1.782855	-5.284314	2.398338
H	-0.337452	-4.298047	2.593865
H	-3.239088	-4.520945	5.775815
H	-3.501017	-5.416222	4.282357
H	-1.063808	-5.237826	4.786955
C	3.406346	2.232971	4.326977
C	1.908442	2.128158	4.652373
C	1.334893	0.837327	4.047600
C	1.528657	0.859341	2.523651
C	3.025618	0.960986	2.192468
C	3.594946	2.251292	2.802322
C	2.075048	-0.373417	4.636718
C	4.140961	1.018697	4.915894
C	3.761649	-0.249770	2.786659
C	3.573515	-0.274674	4.311202
H	3.813174	3.153914	4.758792
H	1.757625	2.134654	5.738625
H	1.375482	2.999628	4.253068
H	0.267021	0.764393	4.281300
H	1.104976	-0.048613	2.077589
H	0.988579	1.708396	2.087445
H	3.161322	0.977075	1.105558
H	4.659560	2.346081	2.556196
H	3.091749	3.125095	2.370765
H	1.928041	-0.413366	5.722825
H	1.661362	-1.302794	4.226893
H	5.215388	1.092007	4.707974
H	4.029923	1.004306	6.006867
H	3.377597	-1.177280	2.345018
H	4.829104	-0.199643	2.539695
H	4.098484	-1.138818	4.732999

Adamantane$_{27}$

702
E=-1209.9943

C	-1.636110	-5.911169	9.754990
C	-1.442378	-5.925897	8.230905
C	0.056387	-5.866756	7.897499
C	0.656614	-4.577401	8.478652
C	0.468324	-4.558898	10.003484
C	-1.031469	-4.621792	10.331526
C	0.764069	-7.081442	8.517590
C	-0.924314	-7.126119	10.369963
C	1.174510	-5.777118	10.618282
C	0.575701	-7.069481	10.042545
H	-2.705169	-5.953160	9.990485
H	-1.887929	-6.832206	7.803331
H	-1.962281	-5.072967	7.777957
H	0.192173	-5.877936	6.810428
H	1.722891	-4.513640	8.229966
H	0.173556	-3.701173	8.029503
H	0.896472	-3.638606	10.415551
H	-1.180878	-4.589256	11.417613

H	-1.544432	-3.746120	9.915538
H	0.359306	-8.009482	8.095991
H	1.832340	-7.060882	8.270188
H	-1.072493	-7.138477	11.456649
H	-1.359650	-8.054342	9.980549
H	2.249970	-5.734378	10.407625
H	1.064752	-5.764702	11.709503
H	1.080756	-7.936894	10.481594
C	-2.030452	-10.943028	4.042910
C	-0.648123	-10.722487	3.409575
C	-0.173084	-9.287725	3.686612
C	-1.177927	-8.293659	3.084473
C	-2.561845	-8.507810	3.716784
C	-3.030610	-9.944685	3.440024
C	-0.084843	-9.064903	5.204293
C	-1.936997	-10.716295	5.559751
C	-2.467554	-8.287466	5.234444
C	-1.465974	-9.281406	5.842036
H	-2.367325	-11.966301	3.844063
H	0.071828	-11.442836	3.816392
H	-0.697727	-10.899979	2.328377
H	0.812848	-9.132901	3.234668
H	-0.839433	-7.265042	3.258330
H	-1.236178	-8.428630	1.997622
H	-3.276678	-7.798459	3.285641
H	-4.027035	-10.104821	3.869511
H	-3.122083	-10.108833	2.359438
H	0.645086	-9.754758	5.644905
H	0.271986	-8.049479	5.415051
H	-2.913833	-10.890907	6.026975
H	-1.239682	-11.435796	6.005654
H	-2.152120	-7.258654	5.446257
H	-3.454129	-8.417588	5.695642
H	-1.399674	-9.122463	6.923975
C	3.422933	7.562251	2.590473
C	2.977043	6.133542	2.243260
C	1.596580	5.856564	2.858530
C	1.679170	6.011712	4.384819
C	2.121826	7.440113	4.737851
C	3.500763	7.713195	4.117443
C	0.578999	6.862988	2.299576
C	2.400659	8.564078	2.031686
C	1.103240	8.442619	4.173760
C	1.019107	8.293620	2.647009
H	4.407283	7.757560	2.151132
H	2.936895	6.005550	1.154804
H	3.709152	5.409395	2.620857
H	1.280422	4.837647	2.609250
H	0.703803	5.796780	4.837942
H	2.388250	5.285028	4.799537
H	2.180543	7.548253	5.826385
H	3.837603	8.723747	4.378762
H	4.242265	7.016897	4.527714
H	0.494489	6.749239	1.212031

H	-0.415368	6.663327	2.716953
H	2.718381	9.589773	2.255289
H	2.349151	8.479647	0.939451
H	0.117778	8.270795	4.623381
H	1.397242	9.466055	4.436641
H	0.292065	9.008744	2.246690
C	-1.223505	8.130836	8.922198
C	-1.014679	8.151806	7.400181
C	-1.586512	6.867556	6.779970
C	-0.868193	5.649065	7.379758
C	-1.076420	5.621458	8.901857
C	-0.506259	6.909070	9.516607
C	-3.088283	6.776695	7.091772
C	-2.726355	8.037159	9.228160
C	-2.579473	5.534249	9.208388
C	-3.302148	6.752342	8.613212
H	-0.814979	9.047065	9.362357
H	-1.504809	9.030997	6.964833
H	0.054007	8.237547	7.168856
H	-1.437044	6.884912	5.694777
H	-1.253945	4.726157	6.929986
H	0.202853	5.691121	7.147495
H	-0.563027	4.752306	9.327357
H	-0.630803	6.893153	10.606195
H	0.571330	6.973295	9.322555
H	-3.617203	7.630565	6.651335
H	-3.512526	5.873048	6.637598
H	-2.890361	8.042071	10.312630
H	-3.247905	8.913709	8.825166
H	-2.995369	4.609171	8.791160
H	-2.740719	5.493305	10.292496
H	-4.373662	6.688167	8.832278
C	-6.355371	4.044843	8.966832
C	-7.857338	3.958716	9.278700
C	-8.441734	2.675797	8.667511
C	-8.234054	2.696724	7.145382
C	-6.733018	2.779700	6.827715
C	-6.152895	4.062143	7.443946
C	-7.722272	1.455247	9.262006
C	-5.641491	2.820833	9.561073
C	-6.017726	1.559186	7.427246
C	-6.219818	1.535297	8.950018
H	-5.940806	4.960171	9.403203
H	-8.017792	3.966147	10.363565
H	-8.375625	4.837225	8.875521
H	-9.512496	2.616183	8.891569
H	-8.665177	1.794610	6.694626
H	-8.759411	3.552942	6.705217
H	-6.587383	2.795044	5.742080
H	-5.085003	4.143662	7.207456
H	-6.641103	4.942636	7.008786
H	-7.879504	1.416188	10.346713
H	-8.143997	0.531553	8.847554
H	-4.564402	2.880717	9.362854

H	-5.762478	2.806870	10.650994
H	-6.409511	0.637011	6.981247
H	-4.947550	1.596011	7.189899
H	-5.709630	0.664195	9.375623
C	5.583204	4.851802	8.179990
C	5.138820	3.417669	7.853845
C	3.773155	3.137715	8.500016
C	3.886546	3.306008	10.022931
C	4.327820	4.739863	10.354963
C	5.691876	5.015869	9.703719
C	2.738106	4.133479	7.954196
C	4.543495	5.842943	7.634472
C	3.291684	5.731660	9.804060
C	3.176703	5.569502	8.280666
H	6.557002	5.049228	7.718632
H	5.076807	3.280414	6.767546
H	5.882986	2.700988	8.222000
H	3.458036	2.114928	8.265723
H	2.922134	3.089127	10.498040
H	4.608476	2.586964	10.428698
H	4.408519	4.857377	11.441115
H	6.027973	6.030514	9.949685
H	6.445944	4.327363	10.104116
H	2.631675	4.010213	6.869616
H	1.753863	3.931460	8.393839
H	4.859603	6.872305	7.843006
H	4.469782	5.749148	6.544276
H	2.316846	5.557796	10.275527
H	3.584908	6.758949	10.052375
H	2.437185	6.277003	7.889762
C	-3.499023	10.741174	3.399179
C	-5.000619	10.591103	3.687749
C	-5.534282	9.318068	3.012814
C	-5.310329	9.414074	1.495884
C	-3.809491	9.561227	1.201371
C	-3.280139	10.833419	1.881267
C	-4.780591	8.096796	3.561663
C	-2.750790	9.516273	3.947551
C	-3.060025	8.339626	1.755199
C	-3.278301	8.240821	3.272710
H	-3.120656	11.649357	3.881001
H	-5.173421	10.544573	4.769740
H	-5.543821	11.469010	3.317392
H	-6.604860	9.212788	3.220339
H	-5.705529	8.519176	1.000069
H	-5.859425	10.271146	1.087502
H	-3.652263	9.630037	0.119432
H	-2.213032	10.961031	1.662259
H	-3.792988	11.715743	1.479435
H	-4.948595	8.003963	4.641474
H	-5.166076	7.179039	3.101275
H	-1.674189	9.620876	3.765929
H	-2.883482	9.449473	5.034146
H	-3.415286	7.425799	1.263747

H	-1.989111	8.422729	1.533380
H	-2.743696	7.369035	3.665695
C	-8.945369	5.465016	1.230460
C	-8.408057	6.739804	1.898887
C	-8.621067	6.658523	3.418418
C	-10.121856	6.517014	3.715160
C	-10.663903	5.241671	3.051390
C	-10.445311	5.326385	1.532842
C	-7.875753	5.434290	3.972089
C	-8.198706	4.244062	1.789665
C	-9.913143	4.021022	3.605563
C	-8.411873	4.156195	3.308643
H	-8.792754	5.525102	0.147312
H	-7.341672	6.862201	1.674001
H	-8.920638	7.620845	1.493943
H	-8.236758	7.568139	3.892961
H	-10.288926	6.478065	4.798409
H	-10.663978	7.394472	3.342298
H	-11.734052	5.143133	3.263806
H	-10.846148	4.429937	1.044296
H	-10.993497	6.182550	1.121196
H	-6.799440	5.531812	3.785338
H	-8.004323	5.376089	5.059708
H	-8.559107	3.328472	1.305099
H	-7.128312	4.321471	1.563784
H	-10.076775	3.937838	4.686823
H	-10.304997	3.101392	3.154237
H	-7.878201	3.285332	3.704929
C	10.195012	-1.067487	3.546174
C	9.708176	-2.487536	3.219013
C	8.332978	-2.725825	3.861666
C	8.447478	-2.560786	5.384853
C	8.931182	-1.140920	5.717895
C	10.304664	-0.906529	5.070165
C	7.330028	-1.699217	3.313070
C	9.187262	-0.045358	2.997869
C	7.927037	-0.118245	5.164214
C	7.811136	-0.277102	3.640541
H	11.175542	-0.899770	3.087326
H	9.644841	-2.623035	2.132569
H	10.429304	-3.226397	3.589163
H	7.987587	-3.738623	3.626652
H	7.475704	-2.748232	5.857480
H	9.146212	-3.301329	5.792559
H	9.012595	-1.025721	6.804242
H	10.670746	0.097497	5.316907
H	11.036461	-1.617523	5.472581
H	7.222715	-1.819385	2.228229
H	6.338974	-1.871212	3.750178
H	9.533907	0.973978	3.207126
H	9.113561	-0.137062	1.907494
H	6.946156	-0.262377	5.633166
H	8.250644	0.899721	5.413192
H	7.094433	0.452437	3.247655

C	8.631624	6.366133	4.236110
C	7.150202	6.263039	4.630248
C	6.549835	4.969149	4.058969
C	6.673649	4.983354	2.527657
C	8.153865	5.083251	2.127796
C	8.750248	6.376642	2.704348
C	7.316417	3.761411	4.619782
C	9.392664	5.154864	4.796995
C	8.916559	3.875527	2.693867
C	8.798397	3.858435	4.225530
H	9.057604	7.289261	4.644137
H	7.049254	6.275102	5.722217
H	6.599353	7.132472	4.251299
H	5.493791	4.897449	4.341665
H	6.230179	4.073143	2.106102
H	6.114005	5.830184	2.112286
H	8.239682	5.093770	1.035746
H	9.802463	6.470133	2.409276
H	8.227654	7.248242	2.291807
H	7.219276	3.727028	5.711669
H	6.884594	2.829963	4.234078
H	10.456434	5.227072	4.539750
H	9.331672	5.146062	5.891966
H	8.512883	2.945781	2.275015
H	9.971584	3.924352	2.398057
H	9.342291	2.996443	4.627275
C	3.529015	-1.820581	9.670949
C	3.724326	-1.827019	8.147008
C	5.223448	-1.766362	7.815484
C	5.823328	-0.480326	8.404298
C	5.633458	-0.470118	9.929012
C	4.133313	-0.534497	10.255150
C	5.930245	-2.984564	8.429662
C	4.239930	-3.039019	10.280010
C	6.338763	-1.691824	10.537875
C	5.740296	-2.980898	9.954463
H	2.459703	-1.863640	9.905102
H	3.279039	-2.730886	7.714025
H	3.205063	-0.971520	7.698189
H	5.360360	-1.771629	6.728510
H	6.889875	-0.415424	8.157070
H	5.340910	0.398442	7.959444
H	6.061361	0.447822	10.346547
H	3.982783	-0.507867	11.341243
H	3.620956	0.343540	9.843423
H	5.525736	-3.910203	8.002577
H	6.998776	-2.962870	8.183483
H	4.090622	-3.057285	11.366459
H	3.804815	-3.965011	9.885078
H	7.414450	-1.648152	10.328570
H	6.227876	-1.685349	11.629033
H	6.244722	-3.850801	10.389288
C	-4.769888	-3.381330	8.256282
C	-5.772276	-2.386347	7.651459

C	-7.159652	-2.603217	8.275244
C	-7.626748	-4.038996	7.990977
C	-6.629178	-5.038525	8.596538
C	-5.243067	-4.815170	7.972236
C	-7.074106	-2.387800	9.794147
C	-4.690397	-3.163358	9.775201
C	-6.544541	-4.816694	10.114550
C	-6.074797	-3.382933	10.404232
H	-3.781628	-3.225191	7.810107
H	-5.435904	-1.357772	7.829277
H	-5.823962	-2.519493	6.564006
H	-7.872554	-1.892428	7.842998
H	-8.625541	-4.200247	8.414378
H	-7.711203	-4.200523	6.909482
H	-6.963890	-6.061393	8.392194
H	-4.525185	-5.536177	8.381822
H	-5.285161	-4.990835	6.890409
H	-6.761331	-1.359460	10.012150
H	-8.063133	-2.521018	10.249025
H	-3.962112	-3.854506	10.216688
H	-4.335371	-2.148564	9.991788
H	-7.524155	-4.991969	10.575475
H	-5.850223	-5.538156	10.562189
H	-6.015572	-3.226462	11.486941
C	-4.136899	-8.247751	-1.634875
C	-2.737191	-7.994996	-2.215838
C	-2.299950	-6.554383	-1.908580
C	-3.301359	-5.573444	-2.537376
C	-4.702741	-5.819829	-1.957435
C	-5.133682	-7.262440	-2.264209
C	-2.270548	-6.345025	-0.386708
C	-4.102331	-8.034376	-0.113618
C	-4.667245	-5.612835	-0.435388
C	-3.669294	-6.593764	0.198741
H	-4.446771	-9.275148	-1.855205
H	-2.018661	-8.705694	-1.789980
H	-2.744530	-8.162642	-3.299717
H	-1.301567	-6.376580	-2.323194
H	-2.989073	-4.540425	-2.341911
H	-3.317949	-5.698685	-3.626823
H	-5.415072	-5.119789	-2.407519
H	-6.141641	-7.445528	-1.872465
H	-5.183133	-7.417540	-3.348864
H	-1.543845	-7.025383	0.073565
H	-1.941014	-5.325217	-0.153826
H	-5.091796	-8.231944	0.316410
H	-3.407839	-8.745040	0.350490
H	-4.379366	-4.580431	-0.202823
H	-5.667075	-5.766045	-0.011322
H	-3.644944	-6.444355	1.283791
C	1.034426	-4.124089	-1.683821
C	2.418137	-3.855997	-2.295422
C	2.851484	-2.414403	-1.987248
C	1.828138	-1.436537	-2.584733

C	0.442682	-1.698265	-1.974065
C	0.015659	-3.141811	-2.281918
C	2.915023	-2.216439	-0.464894
C	1.103084	-3.922064	-0.162171
C	0.512334	-1.502618	-0.451697
C	1.532347	-2.480541	0.151200
H	0.727303	-5.152161	-1.904850
H	3.151865	-4.564297	-1.892077
H	2.386623	-4.015422	-3.380108
H	3.838470	-2.225659	-2.423734
H	2.137048	-0.402635	-2.388574
H	1.786922	-1.553588	-3.674437
H	-0.285337	-1.000389	-2.401858
H	-0.981397	-3.335740	-1.868112
H	-0.058068	-3.289011	-3.366295
H	3.657498	-2.894599	-0.027099
H	3.242155	-1.195899	-0.231838
H	0.125525	-4.130619	0.289361
H	1.813679	-4.630808	0.279976
H	0.797720	-0.469805	-0.217883
H	-0.476064	-1.666854	-0.005536
H	1.581034	-2.339231	1.236516
C	8.006645	-3.614099	-0.353165
C	6.515942	-3.728975	0.000659
C	5.940412	-5.025152	-0.590648
C	6.105228	-5.004941	-2.118023
C	7.594902	-4.893251	-2.477609
C	8.166311	-3.597618	-1.881141
C	6.700263	-6.229318	-0.013239
C	8.760980	-4.821835	0.224156
C	8.350726	-6.097447	-1.895049
C	8.191571	-6.120507	-0.367180
H	8.414894	-2.689358	0.069088
H	6.385601	-3.721286	1.089550
H	5.969271	-2.862190	-0.390220
H	4.877692	-5.105257	-0.336686
H	5.679768	-5.916843	-2.554228
H	5.550906	-4.160698	-2.545618
H	7.709957	-4.878466	-3.566917
H	9.225381	-3.495709	-2.147509
H	7.648773	-2.728355	-2.304831
H	6.574064	-6.268045	1.075526
H	6.285634	-7.162495	-0.413359
H	9.830736	-4.741253	-0.004155
H	8.670649	-4.834737	1.317057
H	7.965111	-7.028612	-2.327543
H	9.412943	-6.040180	-2.162220
H	8.730624	-6.979977	0.046322
C	8.023484	1.725216	-2.043017
C	7.597739	0.284925	-2.366878
C	6.221175	-0.001319	-1.747130
C	6.301282	0.179831	-0.223576
C	6.723796	1.619865	0.106109
C	8.098889	1.902108	-0.518713

C	5.189691	0.981491	-2.322383
C	6.987371	2.703367	-2.618028
C	5.691363	2.598659	-0.474217
C	5.609627	2.423627	-1.998333
H	9.005083	1.927123	-2.485516
H	7.559603	0.138657	-3.453102
H	8.339857	-0.422632	-1.977550
H	5.919385	-1.028497	-1.979756
H	5.328936	-0.041343	0.233023
H	7.020401	-0.529980	0.202910
H	6.780777	1.746547	1.192733
H	8.421474	2.921411	-0.273913
H	8.850002	1.223058	-0.097169
H	5.106998	0.848855	-3.407927
H	4.198140	0.774735	-1.901774
H	7.290636	3.736926	-2.411186
H	6.937266	2.600429	-3.708740
H	4.708319	2.420384	-0.021822
H	5.970934	3.630267	-0.228065
H	4.872707	3.121865	-2.410234
C	-6.755602	-8.456756	4.458073
C	-8.250161	-8.604524	4.782178
C	-9.035434	-7.444412	4.150957
C	-8.517252	-6.112589	4.715031
C	-7.023252	-5.958180	4.390947
C	-6.243165	-7.122303	5.021207
C	-8.835230	-7.463580	2.627690
C	-6.561555	-8.473794	2.933933
C	-6.827991	-5.981592	2.867117
C	-7.341927	-7.312982	2.298332
H	-6.197627	-9.284591	4.909140
H	-8.624733	-9.563126	4.403352
H	-8.399838	-8.612686	5.868678
H	-10.100764	-7.552101	4.382821
H	-9.083936	-5.276948	4.286744
H	-8.672181	-6.077329	5.800208
H	-6.655877	-5.008317	4.794486
H	-5.171667	-7.013308	4.813896
H	-6.357463	-7.104733	6.111897
H	-9.220031	-8.401173	2.208544
H	-9.406871	-6.651019	2.163143
H	-5.495627	-8.389592	2.689587
H	-6.906378	-9.429581	2.521226
H	-7.364605	-5.143465	2.406240
H	-5.766941	-5.851723	2.621615
H	-7.202394	-7.327634	1.211793
C	-5.776777	4.445260	-2.765787
C	-6.758778	5.437193	-3.407913
C	-6.335478	6.876166	-3.074557
C	-6.336792	7.066894	-1.550067
C	-5.353772	6.079130	-0.902842
C	-5.779338	4.642203	-1.242080
C	-4.920480	7.129143	-3.617304
C	-4.363367	4.704472	-3.309871

C	-3.941323	6.333722	-1.451390
C	-3.933910	6.141228	-2.975574
H	-6.080463	3.420114	-3.004449
H	-6.782260	5.293674	-4.494919
H	-7.775374	5.249819	-3.041338
H	-7.036645	7.581663	-3.533823
H	-6.055373	8.096944	-1.299750
H	-7.346169	6.908425	-1.151443
H	-5.356774	6.216526	0.183970
H	-5.096571	3.925871	-0.769150
H	-6.778724	4.440597	-0.837654
H	-4.909302	7.016532	-4.708189
H	-4.614128	8.160051	-3.402337
H	-3.655597	3.988726	-2.874249
H	-4.343396	4.548696	-4.395271
H	-3.617499	7.350723	-1.199070
H	-3.225877	5.647807	-0.981685
H	-2.926257	6.323672	-3.364932
C	5.015227	-5.164815	3.602330
C	4.544848	-6.596312	3.302092
C	3.166511	-6.834015	3.938203
C	3.266301	-6.634859	5.458343
C	3.733499	-5.203447	5.764437
C	5.110202	-4.969769	5.123321
C	2.158539	-5.829389	3.358970
C	4.002496	-4.164708	3.023446
C	2.724413	-4.202876	5.180197
C	2.623167	-4.395876	3.659425
H	5.998022	-4.997529	3.148193
H	4.492175	-6.755961	2.218362
H	5.269804	-7.319894	3.694292
H	2.832872	-7.854997	3.722407
H	2.292331	-6.821484	5.926756
H	3.968556	-7.359526	5.887937
H	3.804445	-5.063918	6.848663
H	5.464513	-3.957101	5.351207
H	5.845281	-5.664688	5.547261
H	2.061730	-5.974106	2.276140
H	1.165449	-6.001526	3.791377
H	4.337549	-3.137737	3.213360
H	3.939072	-4.280761	1.934746
H	1.740955	-4.346368	5.643918
H	3.036100	-3.176638	5.409658
H	1.902922	-3.682063	3.244736
C	2.837657	-7.747508	-0.221412
C	1.342170	-7.873171	0.107698
C	0.790223	-9.182638	-0.476770
C	0.981761	-9.180238	-2.001157
C	2.476353	-9.057846	-2.336004
C	3.024094	-7.748899	-1.746442
C	1.551829	-10.371461	0.129405
C	3.593784	-8.939958	0.384624
C	3.233841	-10.246675	-1.724750
C	3.047983	-10.251858	-0.199733

H	3.229074	-6.813304	0.195920
H	1.192560	-7.852918	1.193948
H	0.793800	-7.017111	-0.303888
H	-0.275955	-9.270411	-0.240449
H	0.573253	-10.101961	-2.432905
H	0.426616	-8.347265	-2.449292
H	2.610462	-9.055789	-3.423230
H	4.086622	-7.639521	-1.995511
H	2.505404	-6.890481	-2.190353
H	1.406827	-10.397578	1.216203
H	1.153727	-11.313883	-0.265802
H	4.666539	-8.851308	0.174066
H	3.484311	-8.939832	1.475851
H	2.865298	-11.187200	-2.151841
H	4.299979	-10.181910	-1.973989
H	3.588264	-11.100393	0.234243
C	0.409606	0.736932	8.217525
C	-0.616028	1.728053	7.646265
C	-1.989645	1.482062	8.289397
C	-2.442321	0.044958	7.988956
C	-1.421430	-0.950785	8.560957
C	-0.049172	-0.698320	7.917438
C	-1.881300	1.673495	9.810056
C	0.511865	0.930837	9.738344
C	-1.314092	-0.752938	10.080848
C	-0.858651	0.682096	10.386624
H	1.388012	0.913845	7.757597
H	-0.290424	2.758027	7.835648
H	-0.684321	1.612203	6.557749
H	-2.719159	2.190137	7.881075
H	-3.431574	-0.136833	8.426206
H	-2.542896	-0.099811	6.906467
H	-1.745899	-1.974594	8.345123
H	0.685072	-1.416168	8.302853
H	-0.107215	-0.856640	6.833674
H	-1.578604	2.702249	10.040003
H	-2.860629	1.519344	10.279080
H	1.256652	0.242454	10.155976
H	0.856957	1.946647	9.965943
H	-2.283350	-0.949108	10.555057
H	-0.602757	-1.472190	10.504690
H	-0.783212	0.821446	11.470663
C	-6.943480	-0.701789	2.638748
C	-7.389800	-2.144645	2.356708
C	-8.778675	-2.387238	2.967552
C	-8.714432	-2.157344	4.485270
C	-8.271418	-0.714499	4.773097
C	-6.884020	-0.476047	4.157294
C	-9.785916	-1.406798	2.347073
C	-7.955432	0.274081	2.018603
C	-9.279614	0.261777	4.147668
C	-9.345373	0.038006	2.629166
H	-5.953133	-0.531026	2.202671
H	-7.416923	-2.325972	1.275451

H	-6.664827	-2.851306	2.778604
H	-9.095119	-3.416331	2.764776
H	-9.696033	-2.347051	4.936189
H	-8.012953	-2.864648	4.944077
H	-8.225795	-0.553048	5.855640
H	-6.547072	0.545229	4.372764
H	-6.149966	-1.153309	4.610570
H	-9.857361	-1.573733	1.265470
H	-10.786022	-1.582962	2.761303
H	-7.637083	1.308676	2.195421
H	-7.993722	0.136107	0.931288
H	-10.271122	0.114980	4.592842
H	-8.985477	1.296113	4.363527
H	-10.065029	0.734522	2.185084
C	-4.009739	0.834729	-1.277482
C	-5.503949	0.686672	-0.951908
C	-6.039962	-0.620331	-1.556380
C	-5.847070	-0.593006	-3.080353
C	-4.353711	-0.448144	-3.411715
C	-3.821902	0.858213	-2.802160
C	-5.265018	-1.809037	-0.967117
C	-3.240231	-0.357666	-0.688418
C	-3.582889	-1.636980	-2.817377
C	-3.770070	-1.667030	-1.292809
H	-3.629689	1.767142	-0.845881
H	-5.654782	0.689005	0.134359
H	-6.061947	1.542278	-1.351318
H	-7.105253	-0.724095	-1.322553
H	-6.244342	-1.512925	-3.526202
H	-6.411537	0.239981	-3.516660
H	-4.218639	-0.428340	-4.498642
H	-2.760502	0.983726	-3.048417
H	-4.350219	1.717006	-3.233826
H	-5.410699	-1.853018	0.119013
H	-5.651676	-2.750073	-1.376725
H	-2.168397	-0.253331	-0.896465
H	-3.350697	-0.375058	0.402570
H	-3.939982	-2.575299	-3.258805
H	-2.517356	-1.556028	-3.064463
H	-3.220270	-2.515540	-0.870908
C	0.814046	3.725299	-3.451857
C	1.360093	5.017556	-2.825368
C	1.184966	4.969102	-1.299611
C	-0.307218	4.826232	-0.962828
C	-0.857881	3.533498	-1.584533
C	-0.677169	3.585436	-3.109522
C	1.950866	3.761547	-0.737473
C	1.581412	2.521152	-2.884295
C	-0.086545	2.329566	-1.022107
C	1.406153	2.466101	-1.358939
H	0.939646	3.761989	-4.539510
H	2.419890	5.140601	-3.079183
H	0.832530	5.886577	-3.236990
H	1.575448	5.891134	-0.855014

H	-0.447390	4.810598	0.124814
H	-0.863504	5.692229	-1.341576
H	-1.921887	3.434008	-1.343619
H	-1.084595	2.676168	-3.567998
H	-1.240343	4.429247	-3.526410
H	3.021671	3.860628	-0.952866
H	1.849403	3.726969	0.354013
H	1.214620	1.593052	-3.339367
H	2.645458	2.599221	-3.138203
H	-0.223067	2.269683	0.064444
H	-0.483939	1.398001	-1.443091
H	1.954528	1.607175	-0.956718
C	-1.760249	3.406896	2.588376
C	-2.205008	1.967917	2.284876
C	-3.586396	1.709543	2.906135
C	-3.506428	1.911965	4.427016
C	-3.064913	3.350816	4.736336
C	-1.685042	3.605135	4.110060
C	-4.603423	2.697713	2.314556
C	-2.781969	4.390475	1.997098
C	-4.082936	4.334910	4.139743
C	-4.164448	4.138619	2.618207
H	-0.775239	3.588921	2.144832
H	-2.243279	1.806228	1.200845
H	-1.473255	1.256142	2.685959
H	-3.901743	0.683251	2.688034
H	-4.482472	1.710757	4.884958
H	-2.797767	1.198795	4.865243
H	-3.008066	3.492673	5.821091
H	-1.349029	4.623450	4.340488
H	-0.943962	2.922213	4.542954
H	-4.686063	2.550270	1.230920
H	-5.598412	2.510653	2.736265
H	-2.465016	5.422747	2.189327
H	-2.831610	4.272204	0.907921
H	-5.069085	4.176677	4.592832
H	-3.789768	5.366131	4.371261
H	-4.891091	4.840674	2.194700
C	-2.143524	-3.144611	2.238972
C	-1.583104	-1.849125	2.845791
C	-1.775672	-1.864145	4.370142
C	-3.273603	-1.975922	4.692877
C	-3.838698	-3.271714	4.090798
C	-3.640497	-3.253287	2.567209
C	-1.035996	-3.072508	4.964727
C	-1.402387	-4.349482	2.838929
C	-3.093531	-4.476537	4.685660
C	-1.595191	-4.371103	4.363008
H	-2.005457	-3.131768	1.152288
H	-0.518710	-1.748464	2.601529
H	-2.091722	-0.980050	2.411086
H	-1.374908	-0.939872	4.800715
H	-3.425892	-1.967480	5.778968
H	-3.811346	-1.108743	4.290773

H	-4.906772	-3.349019	4.321648
H	-4.057840	-4.164803	2.122317
H	-4.185078	-2.408938	2.127339
H	0.038589	-2.995211	4.759476
H	-1.149939	-3.083555	6.055473
H	-1.779452	-5.280417	2.398272
H	-0.334486	-4.293852	2.595515
H	-3.242891	-4.512410	5.771580
H	-3.501622	-5.409755	4.278806
H	-1.065496	-5.230613	4.788349
C	3.404151	2.237955	4.325355
C	1.905560	2.133644	4.647734
C	1.332757	0.843096	4.041652
C	1.529585	0.865227	2.518098
C	3.027244	0.966371	2.189929
C	3.595814	2.256396	2.801084
C	2.071291	-0.367989	4.632103
C	4.137144	1.023342	4.915594
C	3.761643	-0.244724	2.785445
C	3.570443	-0.269748	4.309605
H	3.810446	3.158698	4.758099
H	1.752567	2.140062	5.733682
H	1.373717	3.005357	4.247469
H	0.264392	0.770520	4.273202
H	1.106471	-0.042518	2.071074
H	0.990690	1.714532	2.080915
H	3.165133	0.982544	1.103295
H	4.660954	2.350829	2.557105
H	3.093801	3.130434	2.368627
H	1.922091	-0.408018	5.717908
H	1.658091	-1.297165	4.221334
H	5.212012	1.096289	4.709838
H	4.023912	1.008857	6.006340
H	3.378142	-1.172041	2.342920
H	4.829609	-0.194954	2.540629
H	4.094252	-1.134134	4.732347

Adamantane$_{28}$

728
E=-1261.0416

C	-1.634772	-5.912660	9.758856
C	-1.442916	-5.931450	8.234578
C	0.055279	-5.869605	7.899107
C	0.653048	-4.577049	8.475664
C	0.466625	-4.554468	10.000670
C	-1.032595	-4.620084	10.330789
C	0.766737	-7.080690	8.521917
C	-0.919207	-7.124013	10.376543
C	1.176588	-5.769106	10.618199
C	0.580252	-7.064651	10.047066
H	-2.703428	-5.956586	9.995823
H	-1.886763	-6.840125	7.810265
H	-1.965492	-5.081157	7.779750

H	0.189729	-5.883689	6.811903
H	1.718852	-4.511399	8.225444
H	0.167264	-3.703357	8.024519
H	0.893015	-3.631899	10.409457
H	-1.180722	-4.584681	11.416962
H	-1.548241	-3.746923	9.912845
H	0.363740	-8.010977	8.103592
H	1.834642	-7.058232	8.273107
H	-1.065991	-7.133499	11.463447
H	-1.352736	-8.054463	9.990442
H	2.251675	-5.724341	10.406059
H	1.068169	-5.753710	11.709516
H	1.087998	-7.929504	10.488057
C	-2.028394	-10.938995	4.036929
C	-0.650226	-10.717426	3.394945
C	-0.171033	-9.284762	3.675661
C	-1.178561	-8.286232	3.085513
C	-2.558316	-8.501403	3.726515
C	-3.031236	-9.936191	3.446023
C	-0.071753	-9.069540	5.193756
C	-1.923899	-10.719866	5.554167
C	-2.452989	-8.288669	5.244535
C	-1.448686	-9.287094	5.840160
H	-2.368238	-11.960767	3.835445
H	0.071463	-11.440852	3.793131
H	-0.707723	-10.889522	2.313266
H	0.811927	-9.129203	3.217536
H	-0.837264	-7.258994	3.262023
H	-1.244680	-8.415767	1.998451
H	-3.275075	-7.788863	3.303914
H	-4.024854	-10.096935	3.881739
H	-3.130576	-10.094885	2.365316
H	0.660200	-9.762653	5.625816
H	0.288119	-8.055704	5.406972
H	-2.897686	-10.895300	6.027410
H	-1.224569	-11.442601	5.991602
H	-2.134484	-7.261388	5.459164
H	-3.436488	-8.419569	5.712040
H	-1.374516	-9.133575	6.922371
C	3.422146	7.559212	2.584264
C	2.975555	6.130331	2.238662
C	1.595517	5.854291	2.855307
C	1.679407	6.010744	4.381392
C	2.122769	7.439323	4.732816
C	3.501274	7.711463	4.111037
C	0.577770	6.860525	2.296313
C	2.399704	8.560850	2.025445
C	1.104011	8.441634	4.168689
C	1.018580	8.291331	2.642138
H	4.406191	7.753851	2.143944
H	2.934475	6.001403	1.150351
H	3.707764	5.406300	2.616288
H	1.278858	4.835251	2.607174
H	0.704351	5.796491	4.835503

H	2.388616	5.284215	4.796160
H	2.182412	7.548394	5.821206
H	3.838623	8.722144	4.371200
H	4.242911	7.015306	4.521305
H	0.492333	6.745854	1.208937
H	-0.416311	6.661517	2.714681
H	2.717908	9.586647	2.247893
H	2.347273	8.475483	0.933326
H	0.118869	8.270489	4.619270
H	1.398527	9.465213	4.430436
H	0.291417	9.006318	2.241793
C	-1.221684	8.133022	8.917454
C	-1.012009	8.152685	7.395536
C	-1.584202	6.868261	6.776017
C	-0.866878	5.649849	7.377154
C	-1.075958	5.623549	8.899160
C	-0.505433	6.911331	9.513215
C	-3.086193	6.778464	7.087062
C	-2.724753	8.040405	9.222662
C	-2.579227	5.537399	9.204931
C	-3.300909	6.755420	8.608403
H	-0.812900	9.049372	9.357120
H	-1.501419	9.031802	6.959230
H	0.056851	8.237662	7.164733
H	-1.434126	6.884686	5.690893
H	-1.252886	4.726800	6.927893
H	0.204318	5.691139	7.145448
H	-0.563274	4.754450	9.325623
H	-0.630584	6.896335	10.602746
H	0.572298	6.974816	9.319706
H	-3.614404	7.632278	6.645665
H	-3.510680	5.874694	6.633363
H	-2.889353	8.046256	10.307037
H	-3.245602	8.916923	8.818693
H	-2.995398	4.612222	8.788199
H	-2.741092	5.497393	10.288981
H	-4.372578	6.692002	8.826929
C	-6.346891	4.049565	8.979654
C	-7.847886	3.964936	9.296568
C	-8.435515	2.682367	8.687747
C	-8.232906	2.702618	7.164925
C	-6.732863	2.784096	6.842216
C	-6.149491	4.066194	7.456094
C	-7.715206	1.461337	9.280225
C	-5.632168	2.825080	9.571898
C	-6.016707	1.563106	7.439744
C	-6.213730	1.539889	8.963191
H	-5.930019	4.964646	9.414344
H	-8.004706	3.972860	10.381961
H	-8.366701	4.843799	8.894843
H	-9.505577	2.623822	8.915402
H	-8.666372	1.800763	6.715902
H	-8.758934	3.559186	6.726245
H	-6.590845	2.798960	5.756096

H	-5.082320	4.146644	7.216010
H	-6.638332	4.947003	7.022287
H	-7.868847	1.422769	10.365463
H	-8.139173	0.537905	8.867481
H	-4.555692	2.883898	9.370061
H	-5.749523	2.811575	10.662221
H	-6.410838	0.641155	6.995353
H	-4.947297	1.598860	7.198810
H	-5.702932	0.668448	9.387367
C	5.583063	4.854005	8.177813
C	5.138634	3.419856	7.851798
C	3.772935	3.140021	8.497950
C	3.886281	3.308464	10.020851
C	4.327599	4.742334	10.352754
C	5.691688	5.018222	9.701529
C	2.737944	4.135770	7.951993
C	4.543410	5.845131	7.632159
C	3.291520	5.734116	9.801715
C	3.176586	5.571809	8.278333
H	6.556884	5.051346	7.716469
H	5.076653	3.282493	6.765510
H	5.882759	2.703184	8.220050
H	3.457785	2.117222	8.263750
H	2.921844	3.091668	10.495949
H	4.608169	2.589432	10.426716
H	4.408265	4.859955	11.438897
H	6.027816	6.032879	9.947404
H	6.445716	4.329726	10.102021
H	2.631545	4.012398	6.867422
H	1.753678	3.933833	8.391623
H	4.859551	6.874502	7.840599
H	4.469732	5.751228	6.541969
H	2.316659	5.560338	10.273165
H	3.584776	6.761419	10.049936
H	2.437108	6.279298	7.887332
C	-3.491956	10.742750	3.392299
C	-4.994019	10.595269	3.679770
C	-5.529242	9.322803	3.004996
C	-5.303955	9.417754	1.488198
C	-3.802650	9.562317	1.194784
C	-3.271743	10.833949	1.874516
C	-4.777978	8.100548	3.554983
C	-2.746155	9.516874	3.941805
C	-3.055615	8.339742	1.749746
C	-3.275230	8.241982	3.267131
H	-3.112476	11.650531	3.874004
H	-5.167736	10.549512	4.761648
H	-5.535495	11.473896	3.308595
H	-6.600151	9.219370	3.211738
H	-5.700235	8.523280	0.992481
H	-5.851330	10.275539	1.079002
H	-3.644470	9.630379	0.112936
H	-2.204259	10.959714	1.656278
H	-3.782835	11.716928	1.471887

H	-4.946972	8.008480	4.634705
H	-5.164608	7.183215	3.094710
H	-1.669244	9.619632	3.760972
H	-2.879799	9.450784	5.028327
H	-3.411991	7.426274	1.258431
H	-1.984396	8.420991	1.528721
H	-2.742359	7.369500	3.660925
C	-8.952962	5.479956	1.246448
C	-8.411036	6.755318	1.910038
C	-8.618160	6.677587	3.430568
C	-10.117981	6.539045	3.733539
C	-10.664624	5.263145	3.074626
C	-10.451908	5.344301	1.555050
C	-7.872559	5.453370	3.983888
C	-8.205985	4.259027	1.805290
C	-9.913570	4.042504	3.628420
C	-8.413278	4.174709	3.325282
H	-8.804539	5.537510	0.162581
H	-7.345361	6.875577	1.680680
H	-8.923849	7.636297	1.505252
H	-8.230568	7.587606	3.901656
H	-10.280825	6.502649	4.817520
H	-10.660213	7.416554	3.360959
H	-11.734076	5.166723	3.291478
H	-10.856063	4.447447	1.069997
H	-11.000392	6.200445	1.143756
H	-6.796843	5.548819	3.792679
H	-7.996916	5.397672	5.072128
H	-8.569718	3.342976	1.324098
H	-7.136374	4.334291	1.575018
H	-10.073053	3.961864	4.710492
H	-10.308629	3.122531	3.180600
H	-7.879392	3.303858	3.721305
C	10.196735	-1.063354	3.547847
C	9.710584	-2.483582	3.220444
C	8.334930	-2.722204	3.861996
C	8.448134	-2.556963	5.385259
C	8.931147	-1.136918	5.718539
C	10.305090	-0.902195	5.071909
C	7.332129	-1.695952	3.312464
C	9.189134	-0.041582	2.998603
C	7.927155	-0.114599	5.163922
C	7.812550	-0.273660	3.640172
H	11.177591	-0.895399	3.089784
H	9.648180	-2.619219	2.133964
H	10.431626	-3.222190	3.591268
H	7.990030	-3.735130	3.626812
H	7.476027	-2.744643	5.857109
H	9.146750	-3.297255	5.793619
H	9.011636	-1.021575	6.804940
H	10.670675	0.101966	5.318840
H	11.036766	-1.612929	5.475004
H	7.225741	-1.816271	2.227549
H	6.340768	-1.868190	3.748779

H	9.535308	0.977879	3.208030
H	9.116355	-0.133428	1.908178
H	6.945933	-0.258968	5.632086
H	8.250259	0.903489	5.413052
H	7.095955	0.455625	3.246618
C	8.631364	6.368414	4.233984
C	7.150068	6.264749	4.628445
C	6.550107	4.970566	4.057402
C	6.673590	4.984693	2.526063
C	8.153681	5.085159	2.125880
C	8.749659	6.378842	2.702196
C	7.317300	3.763188	4.618155
C	9.393016	5.157502	4.794810
C	8.916986	3.877794	2.691891
C	8.799156	3.860783	4.223580
H	9.057055	7.291749	4.641843
H	7.049346	6.276863	5.720435
H	6.598784	7.133926	4.249540
H	5.494153	4.898461	4.340329
H	6.230402	4.074266	2.104679
H	6.113513	5.831259	2.110740
H	8.239261	5.095620	1.033811
H	9.801774	6.472736	2.406893
H	8.226623	7.250193	2.289693
H	7.220405	3.728858	5.710065
H	6.885775	2.831532	4.232622
H	10.456703	5.230122	4.537333
H	9.332260	5.148768	5.889794
H	8.513601	2.947848	2.273203
H	9.971929	3.927023	2.395853
H	9.343487	2.999047	4.625282
C	3.530903	-1.818254	9.671099
C	3.726051	-1.824994	8.147139
C	5.225096	-1.763436	7.815433
C	5.824186	-0.476834	8.403818
C	5.634477	-0.466321	9.928549
C	4.134411	-0.531605	10.254870
C	5.932769	-2.980994	8.429878
C	4.242694	-3.036047	10.280427
C	6.340660	-1.687386	10.537680
C	5.742985	-2.977023	9.954699
H	2.461646	-1.861958	9.905382
H	3.281317	-2.729280	7.714460
H	3.206171	-0.969967	7.698135
H	5.361893	-1.768919	6.728445
H	6.890663	-0.411294	8.156454
H	5.341136	0.401487	7.958768
H	6.061815	0.452020	10.345777
H	3.983982	-0.504766	11.340971
H	3.621425	0.345975	9.842951
H	5.528829	-3.907022	8.003100
H	7.001259	-2.958660	8.183576
H	4.093516	-3.054105	11.366897
H	3.808151	-3.962441	9.885805

H	7.416295	-1.643059	10.328245
H	6.229888	-1.680676	11.628849
H	6.248037	-3.846467	10.389715
C	-4.770909	-3.389962	8.254402
C	-5.772865	-2.397277	7.645104
C	-7.160432	-2.611027	8.269540
C	-7.627922	-4.047870	7.991361
C	-6.630785	-5.045096	8.601415
C	-5.244480	-4.824863	7.976434
C	-7.075120	-2.389103	9.787520
C	-4.691650	-3.165480	9.772385
C	-6.546378	-4.816762	10.118476
C	-6.076243	-3.381914	10.402079
H	-3.782512	-3.236052	7.807757
H	-5.436206	-1.368051	7.818562
H	-5.824378	-2.535084	6.558224
H	-7.873027	-1.901882	7.834098
H	-8.626849	-4.206986	8.415253
H	-7.712213	-4.214023	6.910554
H	-6.965777	-6.068729	8.401407
H	-4.526905	-5.544324	8.389262
H	-5.286416	-5.005167	6.895365
H	-6.762066	-1.359933	10.001158
H	-8.064278	-2.520054	10.242769
H	-3.963669	-3.854950	10.216987
H	-4.336349	-2.149875	9.984675
H	-7.526139	-4.989746	10.579955
H	-5.852375	-5.536508	10.569353
H	-6.017183	-3.220805	11.484117
C	-11.216418	-4.712935	-1.567090
C	-10.683340	-3.422201	-0.926452
C	-9.170907	-3.307056	-1.171892
C	-8.460679	-4.519548	-0.550694
C	-8.987959	-5.813359	-1.190174
C	-10.500927	-5.921906	-0.945044
C	-8.901322	-3.279852	-2.684299
C	-10.941801	-4.681608	-3.078563
C	-8.719324	-5.779685	-2.702601
C	-9.430099	-4.570409	-3.329256
H	-12.294649	-4.793184	-1.390774
H	-11.200606	-2.551442	-1.347103
H	-10.890402	-3.424201	0.150615
H	-8.793101	-2.385856	-0.714792
H	-7.377400	-4.438931	-0.701908
H	-8.628677	-4.540150	0.532915
H	-8.480650	-6.676426	-0.745427
H	-10.886887	-6.851941	-1.379825
H	-10.705098	-5.967757	0.131614
H	-9.385879	-2.406849	-3.138068
H	-7.825654	-3.178383	-2.872651
H	-11.336298	-5.589269	-3.551342
H	-11.462860	-3.833150	-3.538318
H	-7.640598	-5.721620	-2.891805
H	-9.073116	-6.707479	-3.168427

H	-9.237006	-4.547744	-4.407426
C	-4.112013	-8.253255	-1.638551
C	-2.722888	-7.998401	-2.243500
C	-2.277647	-6.560732	-1.933946
C	-3.288904	-5.573714	-2.536941
C	-4.679711	-5.822189	-1.932937
C	-5.118647	-7.261868	-2.242138
C	-2.219722	-6.362517	-0.411400
C	-4.048928	-8.051027	-0.116687
C	-4.615694	-5.626348	-0.410346
C	-3.607797	-6.613389	0.198045
H	-4.427613	-9.278546	-1.860555
H	-1.997772	-8.713275	-1.836200
H	-2.750574	-8.158131	-3.328245
H	-1.286820	-6.381430	-2.365686
H	-2.971342	-4.542623	-2.339801
H	-3.325872	-5.690985	-3.626774
H	-5.399095	-5.117804	-2.364651
H	-6.119481	-7.446274	-1.833124
H	-5.188431	-7.408982	-3.326788
H	-1.485748	-7.047316	0.030377
H	-1.884244	-5.344937	-0.177268
H	-5.030586	-8.250219	0.330155
H	-3.347138	-8.766110	0.329302
H	-4.321849	-4.596106	-0.175666
H	-5.607752	-5.781125	0.031050
H	-3.563111	-6.471932	1.283514
C	1.040290	-4.126708	-1.677053
C	2.421138	-3.856795	-2.294296
C	2.852870	-2.413944	-1.989760
C	1.825365	-1.438879	-2.584680
C	0.442755	-1.702433	-1.968372
C	0.017354	-3.147223	-2.272608
C	2.921829	-2.213754	-0.467933
C	1.114358	-3.922448	-0.155955
C	0.517833	-1.504548	-0.446551
C	1.542026	-2.479669	0.153794
H	0.734314	-5.155674	-1.895495
H	3.157766	-4.563121	-1.892780
H	2.385798	-4.017779	-3.378634
H	3.837818	-2.223901	-2.430267
H	2.133022	-0.404113	-2.391123
H	1.780221	-1.557515	-3.674057
H	-0.288237	-1.006553	-2.394343
H	-0.977741	-3.342501	-1.854735
H	-0.060222	-3.296064	-3.356494
H	3.667278	-2.889876	-0.032042
H	3.247874	-1.192264	-0.237530
H	0.138933	-4.132263	0.299589
H	1.828002	-4.629209	0.284452
H	0.802112	-0.470864	-0.215247
H	-0.468538	-1.670073	0.003604
H	1.594576	-2.336765	1.238721
C	8.013434	-3.613384	-0.349266

C	6.522442	-3.725635	0.004185
C	5.944451	-5.020117	-0.588432
C	6.109581	-4.998810	-2.115759
C	7.599538	-4.889738	-2.474975
C	8.173407	-3.595797	-1.877199
C	6.701811	-6.226324	-0.012010
C	8.765270	-4.823150	0.227062
C	8.352869	-6.095973	-1.893403
C	8.193394	-6.120140	-0.365585
H	8.423439	-2.689848	0.073920
H	6.391922	-3.718702	1.093059
H	5.977561	-2.857404	-0.385983
H	4.881529	-5.098352	-0.334733
H	5.682392	-5.909461	-2.552888
H	5.557010	-4.153072	-2.542664
H	7.714818	-4.874167	-3.564248
H	9.232724	-3.495739	-2.143283
H	7.657669	-2.725115	-2.300171
H	6.575339	-6.265815	1.076696
H	6.285405	-7.158304	-0.413073
H	9.835224	-4.744476	-0.000983
H	8.674717	-4.836891	1.319935
H	7.965487	-7.025968	-2.326833
H	9.415246	-6.040562	-2.160332
H	8.730668	-6.981061	0.047212
C	8.018603	1.729514	-2.045659
C	7.595340	0.288172	-2.368094
C	6.220050	-0.000387	-1.746592
C	6.301531	0.181920	-0.223250
C	6.721571	1.623002	0.105013
C	8.095392	1.907554	-0.521558
C	5.185971	0.980004	-2.321313
C	6.979904	2.705237	-2.620129
C	5.686544	2.599372	-0.474776
C	5.603422	2.423180	-1.998684
H	8.999298	1.933071	-2.489407
H	7.556259	0.141116	-3.454179
H	8.339298	-0.417662	-1.979149
H	5.920028	-1.028312	-1.978204
H	5.330145	-0.040877	0.234600
H	7.022537	-0.526189	0.202881
H	6.779536	1.750509	1.191489
H	8.416238	2.927652	-0.277791
H	8.848325	1.230267	-0.100426
H	5.102306	0.846493	-3.406676
H	4.195310	0.771563	-1.899441
H	7.281359	3.739529	-2.414308
H	6.928763	2.601485	-3.710716
H	4.704370	2.419450	-0.021146
H	5.964354	3.631692	-0.229617
H	4.864654	3.119689	-2.410201
C	-6.797052	-8.462690	4.422522
C	-8.303697	-8.577501	4.700773
C	-9.040282	-7.385144	4.070947

C	-8.504731	-6.078586	4.676370
C	-6.998475	-5.957122	4.398259
C	-6.267147	-7.153334	5.026894
C	-8.796446	-7.379924	2.553941
C	-6.559287	-8.455162	2.904511
C	-6.759669	-5.956003	2.880463
C	-7.290829	-7.262148	2.270462
H	-6.273836	-9.313484	4.872526
H	-8.691559	-9.518405	4.292123
H	-8.485051	-8.602867	5.782170
H	-10.114313	-7.469357	4.270077
H	-9.037112	-5.220204	4.249042
H	-8.690182	-6.060389	5.757171
H	-6.618746	-5.025245	4.831225
H	-5.187633	-7.068455	4.852505
H	-6.412610	-7.153990	6.114009
H	-9.192818	-8.298778	2.105128
H	-9.333316	-6.543774	2.089767
H	-5.484898	-8.394196	2.692449
H	-6.916366	-9.393373	2.462907
H	-7.260990	-5.095292	2.421474
H	-5.688950	-5.849245	2.668029
H	-7.120195	-7.259318	1.188277
C	-5.784016	4.437959	-2.760718
C	-6.768963	5.426096	-3.404185
C	-6.349624	6.866787	-3.073249
C	-6.351091	7.059960	-1.549067
C	-5.365136	6.076003	-0.900508
C	-5.786748	4.637343	-1.237328
C	-4.935480	7.122866	-3.616764
C	-4.371477	4.700268	-3.305579
C	-3.953546	6.333682	-1.449826
C	-3.945976	6.138760	-2.973701
H	-6.084880	3.411581	-2.997655
H	-6.792314	5.280764	-4.490953
H	-7.784936	5.236455	-3.037050
H	-7.052887	7.569571	-3.533469
H	-6.072505	8.091197	-1.300477
H	-7.359919	6.899295	-1.149931
H	-5.368251	6.215137	0.186083
H	-5.101851	3.923693	-0.763424
H	-6.785462	4.433579	-0.832323
H	-4.924260	7.008534	-4.707470
H	-4.631972	8.154975	-3.403532
H	-3.661589	3.987214	-2.868990
H	-4.351341	4.542804	-4.390733
H	-3.632518	7.351993	-1.199222
H	-3.236058	5.650535	-0.979203
H	-2.938938	6.323409	-3.363609
C	5.016651	-5.162679	3.606215
C	4.547812	-6.594979	3.307399
C	3.169018	-6.833017	3.942394
C	3.267056	-6.631747	5.462370
C	3.732707	-5.199528	5.767038

C	5.109875	-4.965522	5.127042
C	2.160788	-5.830031	3.360774
C	4.003664	-4.164216	3.024943
C	2.723372	-4.200604	5.180414
C	2.623874	-4.395720	3.659797
H	5.999774	-4.995155	3.152877
H	4.496403	-6.756120	2.223830
H	5.272980	-7.317415	3.701318
H	2.836480	-7.854572	3.727615
H	2.292760	-6.818583	5.930020
H	3.969485	-7.355237	5.893661
H	3.802406	-5.058490	6.851150
H	5.463081	-3.952247	5.353944
H	5.845109	-5.659244	5.552673
H	2.065230	-5.976276	2.278038
H	1.167397	-6.002444	3.792377
H	4.337639	-3.136705	3.213834
H	3.941473	-4.281776	1.936334
H	1.739555	-4.344321	5.643304
H	3.033940	-3.173793	5.408829
H	1.903449	-3.683080	3.243407
C	2.867549	-7.758967	-0.219083
C	1.370374	-7.876291	0.105398
C	0.812357	-9.181392	-0.483057
C	1.008405	-9.177406	-2.006867
C	2.504682	-9.063337	-2.337095
C	3.058476	-7.758741	-1.743558
C	1.565067	-10.375822	0.123206
C	3.614759	-8.956992	0.387018
C	3.253260	-10.257758	-1.725765
C	3.062872	-10.264563	-0.201313
H	3.263296	-6.827865	0.201086
H	1.217681	-7.857090	1.191238
H	0.828336	-7.016237	-0.306251
H	-0.255019	-9.263223	-0.250033
H	0.595685	-10.095900	-2.441481
H	0.459563	-8.340334	-2.455127
H	2.642011	-9.060135	-3.423915
H	4.122369	-7.655260	-1.989302
H	2.546227	-6.896448	-2.187441
H	1.416703	-10.403019	1.209523
H	1.162520	-11.315142	-0.274874
H	4.688641	-8.874370	0.179777
H	3.502068	-8.958166	1.477916
H	2.880378	-11.195301	-2.155638
H	4.320496	-10.198913	-1.971749
H	3.596800	-11.117082	0.232715
C	0.409429	0.735116	8.215512
C	-0.616181	1.726522	7.644703
C	-1.989580	1.480924	8.288450
C	-2.442795	0.043946	7.988222
C	-1.421930	-0.952081	8.559775
C	-0.049889	-0.700008	7.915642
C	-1.880501	1.672338	9.809059

C	0.512421	0.929004	9.736284
C	-1.313858	-0.754253	10.079616
C	-0.857875	0.680654	10.385178
H	1.387679	0.911750	7.755146
H	-0.290201	2.756406	7.833933
H	-0.684992	1.610684	6.556218
H	-2.719075	2.189202	7.880448
H	-3.431904	-0.137562	8.425915
H	-2.543894	-0.100803	6.905779
H	-1.746785	-1.975800	8.344094
H	0.684325	-1.418061	8.300734
H	-0.108460	-0.858321	6.831905
H	-1.577412	2.701008	10.038863
H	-2.859664	1.518467	10.278522
H	1.257201	0.240413	10.153589
H	0.857903	1.944717	9.963721
H	-2.282959	-0.950146	10.554259
H	-0.602536	-1.473703	10.503146
H	-0.781913	0.819991	11.469182
C	-6.946542	-0.674488	2.650147
C	-7.398712	-2.113615	2.358530
C	-8.787069	-2.355698	2.970750
C	-8.718564	-2.137212	4.489966
C	-8.269688	-0.698128	4.787377
C	-6.882833	-0.460143	4.170172
C	-9.792160	-1.367101	2.359801
C	-7.956360	0.309553	2.039510
C	-9.275760	0.286345	4.171450
C	-9.345761	0.073993	2.651499
H	-5.956573	-0.504086	2.213073
H	-7.428934	-2.286881	1.276034
H	-6.675345	-2.825965	2.773561
H	-9.107688	-3.382125	2.761139
H	-9.699822	-2.326700	4.941723
H	-8.018611	-2.850393	4.941955
H	-8.221035	-0.544812	5.870971
H	-6.541710	0.558300	4.392372
H	-6.150209	-1.143359	4.616776
H	-9.866653	-1.525814	1.277167
H	-10.791958	-1.542709	2.775011
H	-7.633875	1.341667	2.223199
H	-7.997607	0.179724	0.951299
H	-10.266784	0.139845	4.617801
H	-8.977399	1.318001	4.394234
H	-10.063897	0.776344	2.214196
C	-4.016807	0.819439	-1.268214
C	-5.516495	0.679298	-0.965098
C	-6.052575	-0.619697	-1.586534
C	-5.837669	-0.582903	-3.107359
C	-4.338771	-0.445910	-3.416296
C	-3.806972	0.852433	-2.789844
C	-5.294183	-1.817815	-0.994802
C	-3.263872	-0.382329	-0.676785
C	-3.584574	-1.644166	-2.819551

C	-3.793796	-1.683729	-1.298069
H	-3.636715	1.746153	-0.824545
H	-5.682870	0.674973	0.118892
H	-6.062934	1.541506	-1.366291
H	-7.121784	-0.717829	-1.368705
H	-6.234693	-1.496904	-3.565431
H	-6.390200	0.256992	-3.545698
H	-4.187996	-0.419335	-4.501013
H	-2.741329	0.972427	-3.020000
H	-4.323250	1.717852	-3.222841
H	-5.455717	-1.868488	0.088792
H	-5.681256	-2.753263	-1.416638
H	-2.188487	-0.283854	-0.868739
H	-3.390086	-0.406690	0.412359
H	-3.941612	-2.576873	-3.272760
H	-2.515087	-1.568753	-3.050797
H	-3.255829	-2.538944	-0.874454
C	0.803875	3.718199	-3.451622
C	1.350434	5.010463	-2.825594
C	1.175660	4.962446	-1.299782
C	-0.316483	4.820081	-0.962604
C	-0.867658	3.527344	-1.583848
C	-0.687297	3.578842	-3.108894
C	1.941356	3.754820	-0.737520
C	1.571038	2.513982	-2.883937
C	-0.096525	2.323339	-1.021300
C	1.396131	2.459368	-1.358525
H	0.929223	3.754577	-4.539315
H	2.410205	5.133145	-3.079695
H	0.823016	5.879527	-3.237311
H	1.566507	5.884482	-0.855514
H	-0.456398	4.804763	0.125075
H	-0.872617	5.686138	-1.341440
H	-1.931634	3.428213	-1.342653
H	-1.095088	2.669571	-3.567040
H	-1.250335	4.422705	-3.525862
H	3.012138	3.853545	-0.953196
H	1.840146	3.720549	0.353999
H	1.203877	1.585869	-3.338684
H	2.635046	2.591687	-3.138121
H	-0.232803	2.263770	0.065299
H	-0.494282	1.391777	-1.441951
H	1.944361	1.600391	-0.956216
C	-1.764432	3.404559	2.587608
C	-2.208612	1.964383	2.288975
C	-3.589072	1.706973	2.912693
C	-3.507385	1.914016	4.432860
C	-3.066442	3.354079	4.737318
C	-1.687502	3.607419	4.108597
C	-4.607470	2.692695	2.319390
C	-2.787519	4.385688	1.994626
C	-4.085838	4.335709	4.139013
C	-4.169074	4.134794	2.618174
H	-0.780083	3.585896	2.142318

H	-2.248098	1.799413	1.205482
H	-1.475904	1.254296	2.691302
H	-3.904010	0.679824	2.698060
H	-4.482738	1.713546	4.892595
H	-2.797721	1.202630	4.872362
H	-3.008367	3.499231	5.821572
H	-1.351879	4.626641	4.335555
H	-0.945446	2.926286	4.542636
H	-4.691333	2.541942	1.236303
H	-5.601821	2.506251	2.742872
H	-2.471012	5.418740	2.183366
H	-2.838409	4.264112	0.905871
H	-5.071330	4.178193	4.593779
H	-3.793068	5.367813	4.367075
H	-4.896695	4.835097	2.193448
C	-2.146022	-3.145576	2.236982
C	-1.586224	-1.849865	2.843895
C	-1.781793	-1.863616	4.367875
C	-3.280405	-1.974483	4.687752
C	-3.844888	-3.270493	4.085570
C	-3.643685	-3.253339	2.562360
C	-1.043821	-3.071841	4.964851
C	-1.406597	-4.350306	2.839330
C	-3.101423	-4.475181	4.682832
C	-1.602405	-4.370657	4.363043
H	-2.005814	-3.133639	1.150562
H	-0.521307	-1.749862	2.601647
H	-2.093603	-0.980905	2.407516
H	-1.381468	-0.939184	4.798517
H	-3.434824	-1.965129	5.773535
H	-3.816974	-1.107380	4.283919
H	-4.913447	-3.347150	4.314380
H	-4.060555	-4.165017	2.117356
H	-4.187028	-2.409093	2.120765
H	0.031200	-2.995176	4.761653
H	-1.159912	-3.081990	6.055380
H	-1.783206	-5.281417	2.398655
H	-0.338195	-4.295335	2.597972
H	-3.252932	-4.510144	5.768484
H	-3.509125	-5.408536	4.275901
H	-1.073927	-5.230070	4.790091
C	3.402616	2.239216	4.324696
C	1.904244	2.133874	4.647755
C	1.332251	0.842485	4.042700
C	1.528434	0.863844	2.519051
C	3.025874	0.966015	2.190205
C	3.593638	2.256880	2.800335
C	2.072020	-0.367632	4.633588
C	4.136848	1.025566	4.915377
C	3.761511	-0.244113	2.786160
C	3.570958	-0.268359	4.310413
H	3.808334	3.160556	4.756709
H	1.751692	2.140831	5.733762
H	1.371522	3.004905	4.247175

```
H      0.264041      0.769175      4.274733
H      1.105880     -0.044521      2.072758
H      0.988663      1.712439      2.081570
H      3.163303      0.981635      1.103504
H      4.658599      2.352037      2.555859
H      3.090730      3.130240      2.367550
H      1.923299     -0.407117      5.719479
H      1.659414     -1.297398      4.223559
H      5.211570      1.099268      4.709134
H      4.024076      1.011657      6.006178
H      3.378588     -1.172015      2.344361
H      4.829336     -0.193617      2.540874
H      4.095650     -1.132057      4.733468
```

Adamantane$_{29}$

```
        754
E=-1324.3205
C     -1.644711     -5.909539      9.751614
C     -1.450854     -5.927785      8.227583
C      0.047835     -5.867043      7.894123
C      0.645924     -4.575256      8.472069
C      0.457502     -4.553224      9.996838
C     -1.042208     -4.617733     10.324943
C      0.757451     -7.079013      8.517313
C     -0.930981     -7.121778     10.369686
C      1.165628     -5.768743     10.614742
C      0.568961     -7.063522     10.042221
H     -2.713717     -5.952677      9.987147
H     -1.894901     -6.835890      7.802262
H     -1.972115     -5.076844      7.772458
H      0.183711     -5.880737      6.807092
H      1.712113     -4.510385      8.223290
H      0.161471     -3.700948      8.020687
H      0.884124     -3.631202     10.406616
H     -1.191743     -4.582708     11.410935
H     -1.556568     -3.743946      9.906722
H      0.354228     -8.008767      8.098024
H      1.825703     -7.057336      8.269926
H     -1.079212     -7.131643     11.456390
H     -1.364779     -8.051685      9.982581
H      2.241031     -5.724782     10.404046
H      1.055778     -5.753760     11.705922
H      1.075398     -7.929005     10.483482
C     -2.034944    -10.935476      4.029789
C     -0.655974    -10.714279      3.389400
C     -0.176612     -9.281821      3.670879
C     -1.183130     -8.282857      3.079744
C     -2.563684     -8.497657      3.719150
C     -3.036774     -9.932240      3.437901
C     -0.078977     -9.066866      5.189119
C     -1.932093    -10.716616      5.547178
C     -2.460004     -8.285191      5.237321
C     -1.456716     -9.284050      5.833929
```

H	-2.374908	-11.957102	3.827763
H	0.065017	-11.438011	3.788292
H	-0.712305	-10.886189	2.307630
H	0.806919	-9.126527	3.213891
H	-0.841684	-7.255763	3.256799
H	-1.248062	-8.412203	1.992588
H	-3.279721	-7.784807	3.295847
H	-4.030940	-10.092712	3.872468
H	-3.134945	-10.090734	2.357059
H	0.652249	-9.760295	5.621901
H	0.280998	-8.053185	5.402898
H	-2.906475	-10.891791	6.019290
H	-1.233505	-11.439657	5.985293
H	-2.141391	-7.258052	5.452469
H	-3.444076	-8.415829	5.703691
H	-1.383719	-9.130722	6.916247
C	3.427275	7.561544	2.590231
C	2.979904	6.133918	2.240479
C	1.597342	5.858691	2.851811
C	1.676129	6.012260	4.378460
C	2.120251	7.439578	4.734022
C	3.501293	7.710917	4.117545
C	0.582951	6.867358	2.291105
C	2.408182	8.565622	2.029675
C	1.104862	8.444334	4.168173
C	1.024543	8.296917	2.641063
H	4.413119	7.755605	2.153696
H	2.942438	6.007021	1.151799
H	3.709783	5.408181	2.619337
H	1.280132	4.840545	2.600725
H	0.699199	5.798547	4.828782
H	2.382877	5.283989	4.794375
H	2.176251	7.546592	5.822811
H	3.839139	8.720652	4.380717
H	4.240526	7.012982	4.529128
H	0.501145	6.754778	1.203231
H	-0.412859	6.668982	2.705645
H	2.727037	9.590569	2.255095
H	2.359440	8.482308	0.937228
H	0.117918	8.273750	4.615008
H	1.399888	9.467023	4.432805
H	0.299776	9.013645	2.239488
C	-1.225034	8.135043	8.914461
C	-1.014928	8.156477	7.392627
C	-1.586451	6.872523	6.771517
C	-0.868835	5.653718	7.371507
C	-1.078342	5.625647	8.893422
C	-0.508486	6.912964	9.509072
C	-3.088498	6.781807	7.082029
C	-2.728155	8.041512	9.219133
C	-2.581665	5.538584	9.198664
C	-3.303643	6.756990	8.603282
H	-0.816726	9.051061	9.355263
H	-1.504549	9.035890	6.957156

H	0.053966	8.242118	7.162226
H	-1.436070	6.890211	5.686455
H	-1.254360	4.731021	6.921110
H	0.202413	5.695676	7.140156
H	-0.565448	4.756272	9.319068
H	-0.633946	6.896711	10.598549
H	0.569275	6.977077	9.315944
H	-3.616908	7.635908	6.641429
H	-3.512508	5.878378	6.627204
H	-2.893069	8.046095	10.303467
H	-3.249223	8.918279	8.815989
H	-2.997363	4.613711	8.780785
H	-2.743827	5.497311	10.282623
H	-4.375350	6.692919	8.821428
C	-6.347804	4.050963	8.979531
C	-7.849155	3.965473	9.294525
C	-8.435982	2.684291	8.682023
C	-8.231629	2.708131	7.159486
C	-6.731224	2.790483	6.838688
C	-6.148660	4.071183	7.456242
C	-7.716253	1.461930	9.272458
C	-5.633661	2.825148	9.569718
C	-6.015654	1.568151	7.434169
C	-6.214421	1.541343	8.957330
H	-5.931505	4.965054	9.416845
H	-8.007219	3.970837	10.379753
H	-8.367580	4.845236	8.894269
H	-9.506299	2.625127	8.908313
H	-8.664507	1.807299	6.707851
H	-8.757224	3.565685	6.722215
H	-6.587962	2.807908	5.752769
H	-5.081221	4.152280	7.217570
H	-6.637075	4.952970	7.023943
H	-7.871134	1.420802	10.357426
H	-8.139672	0.539435	8.857063
H	-4.556959	2.884524	9.369253
H	-5.752265	2.809074	10.659871
H	-6.409201	0.647215	6.987164
H	-4.945972	1.604554	7.194545
H	-5.704039	0.668949	9.380045
C	5.581139	4.851882	8.178938
C	5.135993	3.417907	7.853139
C	3.769786	3.139117	8.498667
C	3.882443	3.307964	10.021575
C	4.324476	4.741665	10.353260
C	5.689073	5.016507	9.702659
C	2.735695	4.135335	7.951862
C	4.542383	5.843481	7.632435
C	3.289296	5.733915	9.801374
C	3.175054	5.571205	8.277983
H	6.555322	5.048478	7.718039
H	5.074492	3.280244	6.766861
H	5.879484	2.700890	8.222001
H	3.454125	2.116440	8.264621

H	2.917626	3.091913	10.496238
H	4.603675	2.588613	10.428039
H	4.404649	4.859575	11.439409
H	6.025700	6.031033	9.948394
H	6.442467	4.327670	10.103759
H	2.629784	4.011691	6.867274
H	1.751075	3.934144	8.391043
H	4.859052	6.872721	7.840720
H	4.469214	5.749284	6.542237
H	2.314083	5.560887	10.272371
H	3.583058	6.761115	10.049428
H	2.436218	6.279029	7.886378
C	-3.491304	10.747305	3.392462
C	-4.993146	10.594825	3.678479
C	-5.525164	9.324809	2.996592
C	-5.299458	9.428492	1.480428
C	-3.798365	9.578084	1.188451
C	-3.270664	10.847229	1.875290
C	-4.771328	8.101318	3.540275
C	-2.742923	9.520183	3.935642
C	-3.048760	8.354237	1.737107
C	-3.268788	8.247755	3.253845
H	-3.114112	11.653330	3.879240
H	-5.167213	10.542810	4.760018
H	-5.536481	11.474206	3.311834
H	-6.595920	9.217803	3.202307
H	-5.693475	8.535811	0.979702
H	-5.848627	10.287224	1.075647
H	-3.639887	9.652368	0.107056
H	-2.203380	10.976622	1.658199
H	-3.783612	11.731203	1.477228
H	-4.940563	8.003015	4.619410
H	-5.155658	7.185608	3.074874
H	-1.666175	9.626389	3.755835
H	-2.876872	9.447902	5.021732
H	-3.402832	7.442629	1.240697
H	-1.977637	8.439137	1.516988
H	-2.734081	7.374377	3.643138
C	-8.978468	5.467729	1.261958
C	-8.428433	6.749991	1.905269
C	-8.623616	6.691685	3.428246
C	-10.121282	6.560421	3.744811
C	-10.675999	5.277691	3.106220
C	-10.475209	5.339420	1.584110
C	-7.876365	5.472721	3.990859
C	-8.229791	4.252118	1.830041
C	-9.923283	4.062295	3.669219
C	-8.425162	4.187220	3.352563
H	-8.838552	5.511434	0.176314
H	-7.364352	6.864901	1.665981
H	-8.942487	7.627052	1.493608
H	-8.230257	7.606596	3.884857
H	-10.275572	6.537908	4.830419
H	-10.664506	7.434483	3.365629

H	-11.743903	5.186469	3.332736
H	-10.885212	4.437538	1.113502
H	-11.025038	6.191651	1.166535
H	-6.801996	5.563276	3.789945
H	-7.992178	5.430870	5.080661
H	-8.599385	3.330994	1.363212
H	-7.161881	4.322016	1.590368
H	-10.074325	3.995510	4.753444
H	-10.323947	3.137739	3.236056
H	-7.890084	3.320127	3.755168
C	10.194603	-1.065841	3.552976
C	9.708684	-2.486173	3.225676
C	8.332438	-2.724487	3.866072
C	8.444269	-2.558669	5.389373
C	8.927045	-1.138518	5.722548
C	10.301585	-0.904105	5.077074
C	7.330182	-1.698399	3.315240
C	9.187547	-0.044234	3.002429
C	7.923604	-0.116366	5.166631
C	7.810372	-0.276003	3.642839
H	11.175881	-0.898106	3.095738
H	9.647258	-2.622222	2.139191
H	10.429355	-3.224671	3.597434
H	7.987705	-3.737487	3.630962
H	7.471727	-2.746123	5.860415
H	9.142481	-3.298836	5.798650
H	9.006555	-1.022763	6.808977
H	10.666991	0.100134	5.323952
H	11.032862	-1.614718	5.481103
H	7.224771	-1.819128	2.230275
H	6.338418	-1.870425	3.750721
H	9.533578	0.975291	3.211780
H	9.115751	-0.136493	1.911973
H	6.941951	-0.260511	5.633961
H	8.246528	0.901803	5.415665
H	7.094167	0.453164	3.248357
C	8.629664	6.366737	4.235913
C	7.147780	6.262467	4.628000
C	6.549277	4.968012	4.056043
C	6.675213	4.982134	2.524903
C	8.155901	5.083204	2.127093
C	8.750413	6.377158	2.704316
C	7.316074	3.760976	4.618072
C	9.390921	5.156165	4.798006
C	8.918803	3.876179	2.694373
C	8.798521	3.859176	4.225872
H	9.054313	7.290265	4.644420
H	7.045300	6.274579	5.719825
H	6.596741	7.131400	4.248177
H	5.492900	4.895475	4.337277
H	6.233083	4.071506	2.102843
H	6.115449	5.828451	2.108650
H	8.243231	5.093661	1.035162
H	9.802961	6.471481	2.410699

H	8.227675	7.248275	2.290941
H	7.217441	3.726646	5.709827
H	6.885559	2.829125	4.231881
H	10.454989	5.229220	4.542234
H	9.328411	5.147446	5.892892
H	8.516479	2.946050	2.275074
H	9.974198	3.925839	2.400028
H	9.342567	2.997682	4.628480
C	3.524289	-1.819017	9.670337
C	3.720473	-1.826090	8.146511
C	5.219778	-1.765430	7.815819
C	5.819196	-0.479082	8.404424
C	5.628453	-0.468238	9.929025
C	4.128128	-0.532624	10.254332
C	5.926342	-2.983299	8.430924
C	4.234974	-3.037124	10.280328
C	6.333528	-1.689614	10.538815
C	5.735520	-2.978997	9.955614
H	2.454848	-1.862080	9.903897
H	3.275521	-2.730187	7.713661
H	3.201383	-0.970834	7.697028
H	5.357313	-1.771150	6.728926
H	6.885878	-0.414183	8.157778
H	5.336948	0.399448	7.958917
H	6.056027	0.449922	10.346410
H	3.976973	-0.505541	11.340326
H	3.615921	0.345187	9.841934
H	5.522167	-3.909160	8.004005
H	6.995011	-2.961607	8.185348
H	4.085045	-3.054938	11.366699
H	3.800175	-3.963328	9.885544
H	7.409330	-1.645928	10.330108
H	6.222015	-1.682681	11.629907
H	6.239782	-3.848664	10.391102
C	-4.777501	-3.383130	8.245480
C	-5.777442	-2.388214	7.636510
C	-7.166003	-2.601041	8.259051
C	-7.635083	-4.036849	7.978216
C	-6.639968	-5.036299	8.587930
C	-5.252660	-4.816988	7.964853
C	-7.082126	-2.381473	9.777453
C	-4.699674	-3.160995	9.763883
C	-6.556987	-4.810318	10.105422
C	-6.085270	-3.376518	10.391680
H	-3.788391	-3.229875	7.800188
H	-5.439614	-1.359695	7.811872
H	-5.827899	-2.524348	6.549370
H	-7.877158	-1.890307	7.823847
H	-8.634703	-4.195262	8.400736
H	-7.718363	-4.201293	6.897070
H	-6.976091	-6.059190	8.386029
H	-4.536513	-5.538012	8.377433
H	-5.293603	-4.995640	6.883472
H	-6.767949	-1.353036	9.992968

H	-8.071975	-2.511779	10.231382
H	-3.973116	-3.852089	10.208293
H	-4.343267	-2.146179	9.978077
H	-7.537502	-4.982680	10.565530
H	-5.864455	-5.531651	10.556024
H	-6.027229	-3.217084	11.474021
C	-11.213191	-4.710358	-1.564114
C	-10.685112	-3.413029	-0.932724
C	-9.175863	-3.286475	-1.191769
C	-8.450381	-4.490903	-0.572513
C	-8.972628	-5.791257	-1.202785
C	-10.482469	-5.911218	-0.944069
C	-8.919682	-3.262536	-2.706559
C	-10.952006	-4.682251	-3.078029
C	-8.717451	-5.760856	-2.717610
C	-9.443501	-4.559668	-3.342306
H	-12.289156	-4.798735	-1.378106
H	-11.213109	-2.548028	-1.351919
H	-10.882768	-3.412827	0.146111
H	-8.801624	-2.360591	-0.741246
H	-7.369153	-4.402026	-0.733466
H	-8.608767	-4.508961	0.512587
H	-8.454439	-6.648561	-0.759436
H	-10.864594	-6.845924	-1.372172
H	-10.676880	-5.954845	0.134486
H	-9.415281	-2.395145	-3.159164
H	-7.846559	-3.153003	-2.904656
H	-11.343181	-5.594789	-3.544133
H	-11.483955	-3.839722	-3.536214
H	-7.640923	-5.694704	-2.916431
H	-9.067700	-6.693171	-3.177045
H	-9.259991	-4.539323	-4.422194
C	-4.116545	-8.246318	-1.647760
C	-2.725258	-7.993668	-2.248652
C	-2.278814	-6.556580	-1.938138
C	-3.286819	-5.568223	-2.544374
C	-4.679776	-5.814491	-1.944434
C	-5.119920	-7.253599	-2.254576
C	-2.225109	-6.358065	-0.415477
C	-4.057673	-8.043798	-0.125766
C	-4.619983	-5.618359	-0.421709
C	-3.615353	-6.606733	0.189914
H	-4.432999	-9.271198	-1.870443
H	-2.002410	-8.709510	-1.839026
H	-2.749964	-8.153633	-3.333435
H	-1.286448	-6.378851	-2.366984
H	-2.968320	-4.537553	-2.346551
H	-3.320730	-5.685716	-3.634283
H	-5.396836	-5.109155	-2.378454
H	-6.122233	-7.436423	-1.848485
H	-5.186706	-7.400885	-3.339391
H	-1.493460	-7.043835	0.028645
H	-1.888824	-5.340922	-0.180604
H	-5.040944	-8.241427	0.318214

H	-3.358265	-8.759803	0.322479
H	-4.325314	-4.588493	-0.186415
H	-5.613573	-5.771559	0.016783
H	-3.573676	-6.465066	1.275475
C	1.048756	-4.130538	-1.680803
C	2.428617	-3.860503	-2.300196
C	2.860857	-2.417728	-1.996020
C	1.832415	-1.442511	-2.589070
C	0.450790	-1.706187	-1.970608
C	0.024881	-3.150901	-2.274495
C	2.932257	-2.217888	-0.474259
C	1.125262	-3.926628	-0.159779
C	0.528309	-1.508653	-0.448864
C	1.553447	-2.483926	0.149616
H	0.742415	-5.159450	-1.898991
H	3.165877	-4.566931	-1.900022
H	2.391537	-4.021237	-3.384512
H	3.845102	-2.227598	-2.438061
H	2.140397	-0.407795	-2.395768
H	1.785525	-1.560897	-3.678400
H	-0.280873	-1.010200	-2.395248
H	-0.969547	-3.346261	-1.855073
H	-0.054433	-3.299493	-3.358288
H	3.678393	-2.894121	-0.039718
H	3.258686	-1.196456	-0.244144
H	0.150565	-4.136533	0.297279
H	1.839601	-4.633501	0.279322
H	0.812973	-0.475026	-0.217779
H	-0.457342	-1.674267	0.002832
H	1.607738	-2.341272	1.234490
C	8.020369	-3.621012	-0.345632
C	6.528953	-3.731514	0.006579
C	5.950119	-5.025699	-0.585864
C	6.116601	-5.005367	-2.113057
C	7.606985	-4.898046	-2.471032
C	8.181690	-3.604395	-1.873433
C	6.705710	-6.232398	-0.008151
C	8.770432	-4.831264	0.231985
C	8.358542	-6.104765	-1.888173
C	8.197712	-6.127964	-0.360481
H	8.430974	-2.697685	0.077428
H	6.397492	-3.723872	1.095335
H	5.985323	-2.862916	-0.384518
H	4.886895	-5.102686	-0.333050
H	5.688838	-5.915798	-2.550081
H	5.565291	-4.159274	-2.540886
H	7.723229	-4.883167	-3.560212
H	9.241343	-3.505588	-2.138647
H	7.667235	-2.733394	-2.297310
H	6.578249	-6.271185	1.080466
H	6.288675	-7.164151	-0.409088
H	9.840667	-4.753832	0.004831
H	8.678914	-4.844337	1.324785
H	7.970562	-7.034581	-2.321452

H	9.421208	-6.050610	-2.154204
H	8.733722	-6.989232	0.053235
C	8.024587	1.722489	-2.045691
C	7.601237	0.280795	-2.366433
C	6.226287	-0.007204	-1.743920
C	6.308551	0.176664	-0.220807
C	6.728681	1.618104	0.105764
C	8.102160	1.902091	-0.521813
C	5.191854	0.972542	-2.319104
C	6.985535	2.697568	-2.620617
C	5.693299	2.593824	-0.474484
C	5.609393	2.416070	-1.998167
H	9.005040	1.925646	-2.490157
H	7.561598	0.132627	-3.452346
H	8.345436	-0.424600	-1.977154
H	5.926201	-1.035382	-1.974325
H	5.337415	-0.045719	0.237776
H	7.029818	-0.530969	0.205672
H	6.787206	1.746724	1.192079
H	8.423077	2.922455	-0.279256
H	8.855350	1.225276	-0.100381
H	5.107631	0.837917	-3.404285
H	4.201424	0.764478	-1.896503
H	7.287040	3.732086	-2.416010
H	6.933832	2.592698	-3.711070
H	4.711371	2.414312	-0.020158
H	5.971179	3.626409	-0.230524
H	4.870372	3.112118	-2.410011
C	-6.798623	-8.455128	4.412969
C	-8.305058	-8.569895	4.692373
C	-9.041868	-7.376536	4.064711
C	-8.505445	-6.070896	4.671345
C	-6.999397	-5.949478	4.392089
C	-6.267846	-7.146689	5.018559
C	-8.799340	-7.369436	2.547503
C	-6.562167	-8.445719	2.894764
C	-6.761901	-5.946478	2.874090
C	-7.293937	-7.251698	2.262875
H	-6.275247	-9.306638	4.861429
H	-8.693525	-9.510171	4.282853
H	-8.485486	-8.596597	5.773893
H	-10.115749	-7.460717	4.264661
H	-9.037965	-5.211826	4.245576
H	-8.689958	-6.054035	5.752328
H	-6.619045	-5.018257	4.825920
H	-5.188461	-7.061874	4.843347
H	-6.412371	-7.148699	6.105798
H	-9.196346	-8.287609	2.097857
H	-9.336388	-6.532550	2.084865
H	-5.487946	-8.384768	2.681852
H	-6.919878	-9.383268	2.452266
H	-7.263389	-5.085045	2.416637
H	-5.691338	-5.839733	2.660868
H	-7.124237	-7.247527	1.180547

C	-5.771689	4.403523	-2.707123
C	-6.801746	5.356116	-3.333328
C	-6.409378	6.812013	-3.037754
C	-6.367359	7.029217	-1.517390
C	-5.336241	6.080908	-0.886192
C	-5.731119	4.626890	-1.187604
C	-5.020303	7.095125	-3.630298
C	-4.384422	4.692798	-3.301055
C	-3.949944	6.365461	-1.484477
C	-3.985810	6.146669	-3.004704
H	-6.053373	3.366239	-2.918799
H	-6.855887	5.193036	-4.416475
H	-7.800304	5.146687	-2.930869
H	-7.144803	7.489409	-3.485607
H	-6.107688	8.070979	-1.293425
H	-7.358507	6.849439	-1.083616
H	-5.308390	6.237120	0.197722
H	-5.013329	3.938386	-0.725321
H	-6.710865	4.404381	-0.747642
H	-5.040826	6.963843	-4.718958
H	-4.736878	8.137827	-3.442483
H	-3.642863	4.004931	-2.876877
H	-4.394748	4.518718	-4.383805
H	-3.647470	7.395407	-1.259671
H	-3.200481	5.708220	-1.026981
H	-2.996774	6.350521	-3.429560
C	5.013504	-5.163388	3.605096
C	4.544064	-6.595325	3.305485
C	3.164964	-6.832987	3.939958
C	3.262642	-6.632459	5.460055
C	3.728893	-5.200604	5.765517
C	5.106366	-4.966971	5.126041
C	2.157393	-5.829250	3.358493
C	4.001174	-4.164171	3.023977
C	2.720216	-4.200925	5.179046
C	2.621081	-4.395299	3.658310
H	5.996843	-4.996132	3.152130
H	4.492902	-6.755946	2.221827
H	5.268764	-7.318290	3.699292
H	2.831997	-7.854284	3.724612
H	2.288115	-6.819038	5.927326
H	3.964592	-7.356485	5.891226
H	3.798333	-5.060095	6.849714
H	5.459993	-3.953970	5.353512
H	5.841137	-5.661241	5.551576
H	2.062090	-5.974954	2.275662
H	1.163789	-6.001380	3.789719
H	4.335588	-3.136908	3.213437
H	3.939253	-4.281204	1.935295
H	1.736191	-4.344379	5.641575
H	3.031211	-3.174369	5.408023
H	1.901125	-3.682121	3.242029
C	2.863897	-7.759552	-0.222051
C	1.366612	-7.876381	0.102100

C	0.808497	-9.181739	-0.485695
C	1.004957	-9.178738	-2.009455
C	2.501346	-9.065167	-2.339349
C	3.055235	-7.760311	-1.746474
C	1.560810	-10.375940	0.121512
C	3.610709	-8.957347	0.384996
C	3.249524	-10.259355	-1.727076
C	3.058724	-10.265175	-0.202672
H	3.259714	-6.828267	0.197647
H	1.213630	-7.856477	1.187886
H	0.824854	-7.016477	-0.310229
H	-0.258957	-9.263215	-0.252908
H	0.592174	-10.097420	-2.443609
H	0.456400	-8.341837	-2.458382
H	2.638968	-9.062667	-3.426135
H	4.119214	-7.657192	-1.991995
H	2.543276	-6.898194	-2.191030
H	1.412148	-10.402432	1.207806
H	1.158186	-11.315428	-0.276092
H	4.684663	-8.875064	0.177994
H	3.497724	-8.957821	1.475864
H	2.876574	-11.197092	-2.156467
H	4.316838	-10.200871	-1.972809
H	3.592368	-11.117530	0.232030
C	0.406681	0.737343	8.211966
C	-0.617967	1.729615	7.640935
C	-1.991820	1.484866	8.284036
C	-2.445942	0.048281	7.983295
C	-1.426043	-0.948608	8.555066
C	-0.053543	-0.697384	7.911580
C	-1.883257	1.675864	9.804735
C	0.509159	0.930820	9.732825
C	-1.318482	-0.751194	10.074998
C	-0.861596	0.683315	10.381074
H	1.385257	0.913372	7.752060
H	-0.291325	2.759222	7.830534
H	-0.686393	1.614067	6.552395
H	-2.720628	2.193760	7.875877
H	-3.435370	-0.132609	8.420522
H	-2.546680	-0.096154	6.900777
H	-1.751543	-1.972044	8.339019
H	0.679987	-1.416051	8.296829
H	-0.111761	-0.855414	6.827783
H	-1.579526	2.704265	10.034897
H	-2.862734	1.522596	10.273742
H	1.253261	0.241600	10.150298
H	0.855275	1.946233	9.960635
H	-2.287929	-0.946493	10.549180
H	-0.607863	-1.471252	10.498675
H	-0.786000	0.822358	11.465142
C	-6.957575	-0.661441	2.625853
C	-7.402389	-2.103844	2.339150
C	-8.786041	-2.353339	2.958998
C	-8.711013	-2.131683	4.477447

C	-8.269463	-0.689333	4.769961
C	-6.887314	-0.443954	4.145143
C	-9.800345	-1.372042	2.351515
C	-7.976575	0.315272	2.018715
C	-9.284748	0.287825	4.157513
C	-9.361313	0.072293	2.638326
H	-5.970950	-0.485754	2.183344
H	-7.437151	-2.279253	1.257135
H	-6.672514	-2.810985	2.751688
H	-9.101422	-3.382094	2.752881
H	-9.688734	-2.326375	4.934627
H	-8.004355	-2.839732	4.927065
H	-8.216142	-0.533758	5.853013
H	-6.551314	0.576966	4.363756
H	-6.148197	-1.121848	4.589154
H	-9.879467	-1.533171	1.269566
H	-10.796879	-1.553034	2.772221
H	-7.659497	1.349678	2.198890
H	-8.022659	0.183220	0.930966
H	-10.272526	0.136050	4.609261
H	-8.991588	1.321695	4.376908
H	-10.086011	0.769427	2.203507
C	-10.911796	0.337577	-2.678180
C	-9.542400	0.653827	-3.298924
C	-9.161059	2.111920	-2.999764
C	-10.223251	3.048385	-3.595775
C	-11.594633	2.738426	-2.975982
C	-11.969579	1.278618	-3.274849
C	-9.096417	2.319300	-1.478710
C	-10.842194	0.549201	-1.157878
C	-11.523796	2.943716	-1.454939
C	-10.464902	2.007096	-0.853496
H	-11.181840	-0.702196	-2.892791
H	-8.780693	-0.024901	-2.896602
H	-9.574028	0.488216	-4.382679
H	-8.184192	2.335016	-3.442760
H	-9.951776	4.093933	-3.406069
H	-10.266173	2.924735	-4.684684
H	-12.350384	3.406833	-3.402736
H	-12.956444	1.049861	-2.854450
H	-12.043843	1.123660	-4.358108
H	-8.326894	1.671176	-1.042125
H	-8.806033	3.352345	-1.252152
H	-11.808821	0.306729	-0.699800
H	-10.103370	-0.130645	-0.716620
H	-11.275578	3.987483	-1.227439
H	-12.502928	2.745105	-1.002370
H	-10.415438	2.155245	0.230872
C	-3.969496	0.813067	-1.266214
C	-5.468650	0.691780	-0.952511
C	-6.026488	-0.597832	-1.574341
C	-5.822274	-0.558625	-3.096578
C	-4.323993	-0.440456	-3.416107
C	-3.770408	0.848542	-2.789232

C	-5.279764	-1.807944	-0.992257
C	-3.228279	-0.400623	-0.684406
C	-3.581429	-1.650668	-2.828980
C	-3.779992	-1.692721	-1.306137
H	-3.573879	1.733115	-0.822247
H	-5.627112	0.685908	0.132656
H	-6.006511	1.562543	-1.346757
H	-7.095279	-0.682527	-1.348958
H	-6.234757	-1.465689	-3.554780
H	-6.366795	0.290037	-3.527999
H	-4.180840	-0.412127	-4.501811
H	-2.704981	0.955188	-3.026842
H	-4.278302	1.722227	-3.215492
H	-5.434004	-1.860222	0.092323
H	-5.682317	-2.736710	-1.414370
H	-2.153117	-0.315755	-0.883959
H	-3.346812	-0.427079	0.405552
H	-3.954144	-2.576984	-3.282677
H	-2.512759	-1.588647	-3.067858
H	-3.250344	-2.556457	-0.889384
C	0.811723	3.722472	-3.451299
C	1.367483	5.010571	-2.824788
C	1.193073	4.962893	-1.298924
C	-0.299866	4.830651	-0.961145
C	-0.860242	3.542108	-1.582869
C	-0.680221	3.593228	-3.107968
C	1.950660	3.749684	-0.737697
C	1.570803	2.512659	-2.884647
C	-0.097193	2.332482	-1.021357
C	1.396214	2.458385	-1.359187
H	0.936827	3.758603	-4.539028
H	2.427961	5.126072	-3.079303
H	0.845897	5.883493	-3.235771
H	1.590484	5.881953	-0.854311
H	-0.439390	4.815680	0.126589
H	-0.850172	5.700746	-1.339235
H	-1.924769	3.450195	-1.341244
H	-1.094494	2.687058	-3.566444
H	-1.237603	4.441199	-3.524200
H	3.022000	3.841130	-0.953806
H	1.849710	3.715491	0.353849
H	1.197030	1.587364	-3.339753
H	2.635207	2.583154	-3.139273
H	-0.233388	2.273238	0.065271
H	-0.501569	1.403931	-1.442354
H	1.938676	1.595411	-0.957616
C	-1.770852	3.415418	2.581238
C	-2.212162	1.974318	2.282811
C	-3.592132	1.714265	2.906516
C	-3.510914	1.921705	4.426654
C	-3.072840	3.362687	4.730906
C	-1.694382	3.618665	4.102198
C	-4.612461	2.697873	2.313022
C	-2.795861	4.394423	1.988066

C	-4.094159	4.342200	4.132412
C	-4.176939	4.140885	2.611601
H	-0.786848	3.598639	2.135957
H	-2.251279	1.809102	1.199341
H	-1.478061	1.265748	2.685274
H	-3.905023	0.686460	2.692030
H	-4.485884	1.719371	4.886384
H	-2.799856	1.211796	4.866293
H	-3.015094	3.508121	5.815140
H	-1.360791	4.638586	4.329012
H	-0.950992	2.939073	4.536370
H	-4.695984	2.546787	1.229956
H	-5.606457	2.509522	2.736496
H	-2.481411	5.428131	2.176658
H	-2.846469	4.272579	0.899327
H	-5.079354	4.182798	4.587165
H	-3.803446	5.374918	4.360325
H	-4.905932	4.839677	2.186739
C	-2.144267	-3.137873	2.232367
C	-1.582523	-1.844563	2.842593
C	-1.780753	-1.860622	4.366208
C	-3.280166	-1.968600	4.683303
C	-3.846603	-3.262207	4.077799
C	-3.642734	-3.242759	2.554971
C	-1.046606	-3.071631	4.962258
C	-1.408668	-4.345400	2.833798
C	-3.106956	-4.469693	4.674150
C	-1.607151	-4.368059	4.357132
H	-2.002157	-3.124292	1.146213
H	-0.516962	-1.746586	2.602361
H	-2.087139	-0.973641	2.406921
H	-1.379036	-0.937901	4.799216
H	-3.436436	-1.960856	5.768834
H	-3.814032	-1.099528	4.280123
H	-4.915730	-3.336805	4.304630
H	-4.060941	-4.152662	2.107596
H	-4.183361	-2.396459	2.113975
H	0.028938	-2.997087	4.761050
H	-1.164601	-3.083488	6.052565
H	-1.786667	-5.274838	2.390786
H	-0.339727	-4.292465	2.594379
H	-3.260419	-4.506272	5.759475
H	-3.516112	-5.401363	4.264824
H	-1.081398	-5.229465	4.783528
C	3.400086	2.240568	4.323789
C	1.901161	2.135788	4.644457
C	1.329653	0.844607	4.038500
C	1.528277	0.865879	2.515167
C	3.026278	0.967488	2.188710
C	3.593548	2.258146	2.799735
C	2.068026	-0.365780	4.630579
C	4.132921	1.026650	4.915652
C	3.760511	-0.242909	2.785850
C	3.567516	-0.267070	4.309797

H	3.805458	3.161761	4.756442
H	1.746878	2.142812	5.730219
H	1.369404	3.007014	4.243019
H	0.261046	0.771698	4.268828
H	1.106097	-0.042333	2.068207
H	0.989522	1.714671	2.076817
H	3.165448	0.983047	1.102231
H	4.658934	2.352904	2.556960
H	3.091658	3.131691	2.366140
H	1.917557	-0.405200	5.716231
H	1.655728	-1.295396	4.219899
H	5.207999	1.099949	4.711125
H	4.018403	1.012792	6.006272
H	3.377948	-1.170672	2.343448
H	4.828745	-0.192814	2.542269
H	4.091209	-1.130959	4.733697

Adamantane$_{30}$

780
E=-1368.3366

C	-1.641522	-5.909535	9.750784
C	-1.448147	-5.927270	8.226686
C	0.050416	-5.865966	7.892764
C	0.648292	-4.574165	8.470902
C	0.460352	-4.552644	9.995737
C	-1.039233	-4.617714	10.324303
C	0.760606	-7.077901	8.515366
C	-0.927219	-7.121737	10.368267
C	1.169051	-5.768127	10.613053
C	0.572601	-7.062921	10.040339
H	-2.710438	-5.953074	9.986647
H	-1.892050	-6.835386	7.801237
H	-1.969816	-5.076354	7.771980
H	0.185949	-5.879295	6.805685
H	1.714380	-4.508891	8.221800
H	0.163424	-3.699873	8.019934
H	0.886820	-3.630612	10.405652
H	-1.188431	-4.583057	11.410353
H	-1.553997	-3.743962	9.906506
H	0.357536	-8.007656	8.095931
H	1.828773	-7.055821	8.267644
H	-1.075098	-7.131971	11.455016
H	-1.360854	-8.051664	9.981025
H	2.244373	-5.723772	10.402026
H	1.059546	-5.753502	11.704273
H	1.079446	-7.928378	10.481181
C	-2.033176	-10.936456	4.030418
C	-0.654341	-10.715147	3.389777
C	-0.174902	-9.282751	3.671437
C	-1.181536	-8.283658	3.080717
C	-2.561955	-8.498569	3.720376
C	-3.035122	-9.933091	3.438944
C	-0.076940	-9.068098	5.189698

C	-1.929997	-10.717898	5.547828
C	-2.457947	-8.286406	5.238567
C	-1.454542	-9.285394	5.834761
H	-2.373196	-11.958038	3.828262
H	0.066727	-11.438967	3.788370
H	-0.710907	-10.886843	2.307985
H	0.808532	-9.127378	3.214269
H	-0.840040	-7.256603	3.257901
H	-1.246703	-8.412788	1.993549
H	-3.278075	-7.785627	3.297368
H	-4.029197	-10.093638	3.873692
H	-3.133527	-10.091370	2.358091
H	0.654372	-9.761621	5.622186
H	0.283093	-8.054464	5.403602
H	-2.904280	-10.893155	6.020115
H	-1.231324	-11.441034	5.985650
H	-2.139277	-7.259312	5.453850
H	-3.441920	-8.417124	5.705122
H	-1.381311	-9.132282	6.917094
C	3.433420	7.572877	2.609944
C	2.983691	6.147178	2.255387
C	1.599906	5.872786	2.864322
C	1.677253	6.021605	4.391514
C	2.123725	7.446985	4.751877
C	3.505988	7.717507	4.137784
C	0.588103	6.885096	2.305509
C	2.416906	8.580607	2.051263
C	1.110921	8.455401	4.187909
C	1.032056	8.312738	2.660272
H	4.420132	7.766344	2.175112
H	2.947219	6.023631	1.166288
H	3.711737	5.418893	2.632882
H	1.281017	4.856016	2.609810
H	0.699400	5.808428	4.840085
H	2.382119	5.290720	4.806033
H	2.178693	7.550612	5.841046
H	3.845483	8.725790	4.404380
H	4.243403	7.016908	4.548099
H	0.507318	6.775950	1.217209
H	-0.408559	6.687400	2.718322
H	2.737482	9.604251	2.280130
H	2.369246	8.500677	0.958515
H	0.123143	8.285382	4.633110
H	1.407620	9.476717	4.455954
H	0.309131	9.032072	2.260038
C	-1.230310	8.137381	8.918233
C	-1.018082	8.158172	7.396685
C	-1.588691	6.873932	6.775327
C	-0.871868	5.655410	7.376839
C	-1.083497	5.627982	8.898472
C	-0.514548	6.915584	9.514366
C	-3.091166	6.783292	7.083783
C	-2.733851	8.043924	9.220848
C	-2.587242	5.540993	9.201654

C	-3.308432	6.759118	8.604744
H	-0.822652	9.053603	9.359213
H	-1.507127	9.037381	6.960155
H	0.051129	8.243755	7.167738
H	-1.436797	6.891161	5.690468
H	-1.256730	4.732506	6.926299
H	0.199700	5.697309	7.146965
H	-0.571166	4.758808	9.325205
H	-0.641526	6.899792	10.603674
H	0.563480	6.979654	9.322714
H	-3.618993	7.637185	6.642080
H	-3.514508	5.879653	6.628753
H	-2.900278	8.048966	10.304949
H	-3.254389	8.920500	8.816602
H	-3.002322	4.615926	8.783591
H	-2.750914	5.500178	10.285403
H	-4.380441	6.695101	8.821422
C	-6.350126	4.052560	8.980115
C	-7.851546	3.966698	9.294677
C	-8.437932	2.685497	8.681792
C	-8.233161	2.709643	7.159315
C	-6.732685	2.792368	6.838949
C	-6.150563	4.073085	7.456884
C	-7.718108	1.463186	9.272216
C	-5.635887	2.826794	9.570289
C	-6.017021	1.570085	7.434418
C	-6.216205	1.542972	8.957519
H	-5.934141	4.966663	9.417701
H	-8.009912	3.971842	10.379862
H	-8.370046	4.846420	8.894429
H	-9.508299	2.626068	8.907774
H	-8.665723	1.808796	6.707405
H	-8.758816	3.567160	6.722046
H	-6.589125	2.810010	5.753072
H	-5.083075	4.154448	7.218523
H	-6.639044	4.954842	7.024601
H	-7.873282	1.421839	10.357135
H	-8.141216	0.540674	8.856545
H	-4.559142	2.886432	9.370134
H	-5.754790	2.810508	10.660406
H	-6.410249	0.649142	6.987145
H	-4.947280	1.606755	7.195097
H	-5.705756	0.670613	9.380226
C	5.580479	4.852066	8.184791
C	5.136194	3.417861	7.858832
C	3.770408	3.137996	8.504787
C	3.883496	3.306582	10.027692
C	4.324670	4.740510	10.359538
C	5.688846	5.016426	9.708509
C	2.735443	4.133630	7.958574
C	4.540852	5.843078	7.638880
C	3.288617	5.732177	9.808243
C	3.173940	5.569727	8.284856
H	6.554362	5.049428	7.723587

H	5.074398	3.280397	6.772546
H	5.880305	2.701268	8.227269
H	3.455361	2.115156	8.270627
H	2.918996	3.089767	10.502652
H	4.605364	2.587632	10.433738
H	4.405152	4.858233	11.445684
H	6.024870	6.031126	9.954349
H	6.442853	4.328014	10.109186
H	2.629227	4.010155	6.873997
H	1.751118	3.931671	8.398062
H	4.856894	6.872487	7.847280
H	4.467357	5.749073	6.548687
H	2.313691	5.558379	10.279550
H	3.581767	6.759521	10.056420
H	2.434481	6.277134	7.893673
C	-3.487299	10.744710	3.378565
C	-4.990654	10.593565	3.657244
C	-5.519811	9.322346	2.975373
C	-5.286325	9.422671	1.460161
C	-3.783690	9.570916	1.175525
C	-3.258875	10.841276	1.862328
C	-4.769358	8.099661	3.525512
C	-2.742300	9.518397	3.928182
C	-3.037493	8.347887	1.730616
C	-3.265305	8.244762	3.246437
H	-3.112149	11.651596	3.865320
H	-5.170260	10.543951	4.737990
H	-5.531681	11.472418	3.285951
H	-6.591655	9.216295	3.175852
H	-5.678223	8.529107	0.959344
H	-5.833002	10.280796	1.050747
H	-3.619663	9.642806	0.094796
H	-2.190435	10.969692	1.650407
H	-3.769353	11.724641	1.459763
H	-4.944143	8.003753	4.603978
H	-5.151760	7.183140	3.060118
H	-1.664597	9.623699	3.753642
H	-2.881822	9.448507	5.013727
H	-3.389477	7.435388	1.234359
H	-1.965220	8.431802	1.515782
H	-2.733020	7.371964	3.640321
C	-8.969961	5.468775	1.249876
C	-8.420104	6.751014	1.893386
C	-8.615919	6.692755	3.416283
C	-10.113725	6.561600	3.732233
C	-10.668266	5.278894	3.093441
C	-10.466843	5.340576	1.571413
C	-7.868984	5.473752	3.979231
C	-8.221601	4.253125	1.818293
C	-9.915866	4.063459	3.656777
C	-8.417605	4.188274	3.340736
H	-8.829594	5.512447	0.164289
H	-7.355916	6.865846	1.654534
H	-8.933927	7.628101	1.481494

H	-8.222686	7.607648	3.873037
H	-10.268464	6.539122	4.817778
H	-10.656732	7.435691	3.352808
H	-11.736269	5.187750	3.319519
H	-10.876714	4.438712	1.100656
H	-11.016442	6.192835	1.153592
H	-6.794526	5.564228	3.778758
H	-7.985249	5.431933	5.068986
H	-8.591066	3.332017	1.351332
H	-7.153588	4.322946	1.579060
H	-10.067359	3.996708	4.740941
H	-10.316414	3.138921	3.223469
H	-7.882752	3.321153	3.743581
C	10.194031	-1.067898	3.540827
C	9.705959	-2.487120	3.211923
C	8.330620	-2.725052	3.854406
C	8.445404	-2.562029	5.377790
C	8.930344	-1.142995	5.712573
C	10.303963	-0.908954	5.065008
C	7.328490	-1.696903	3.307199
C	9.187097	-0.044225	2.993906
C	7.927015	-0.118771	5.160276
C	7.810836	-0.275611	3.636416
H	11.174660	-0.900435	3.082097
H	9.642407	-2.621196	2.125315
H	10.426495	-3.227059	3.581074
H	7.984348	-3.737260	3.618150
H	7.473515	-2.749246	5.850272
H	9.143547	-3.303678	5.784496
H	9.011957	-1.029233	6.799057
H	10.670921	0.094450	5.312981
H	11.035195	-1.621075	5.466459
H	7.220974	-1.815612	2.222218
H	6.337332	-1.868607	3.744184
H	9.534628	0.974552	3.204413
H	9.113217	-0.134492	1.903423
H	6.946057	-0.262661	5.629141
H	8.251509	0.898605	5.410506
H	7.094716	0.455031	3.244518
C	8.630137	6.362835	4.233304
C	7.148400	6.260644	4.626491
C	6.547378	4.967566	4.054062
C	6.672121	4.982610	2.522833
C	8.152653	5.081608	2.123921
C	8.749688	6.374187	2.701620
C	7.312691	3.758895	4.614597
C	9.389906	5.150637	4.793905
C	8.914075	3.872951	2.689712
C	8.794981	3.855015	4.221293
H	9.056585	7.285384	4.642150
H	7.046806	6.272118	5.718406
H	6.598449	7.130733	4.247742
H	5.491109	4.896509	4.336082
H	6.228203	4.072999	2.100457

H	6.113380	5.830124	2.107645
H	8.239133	5.092727	1.031930
H	9.802152	6.467045	2.407236
H	8.228015	7.246441	2.289298
H	7.214869	3.723921	5.706405
H	6.880382	2.828016	4.228067
H	10.453886	5.222181	4.537342
H	9.328250	5.141214	5.888834
H	8.509934	2.943772	2.270051
H	9.969315	3.921142	2.394564
H	9.337970	2.992359	4.622837
C	3.528310	-1.821070	9.667016
C	3.723654	-1.825498	8.143072
C	5.222766	-1.763997	7.811658
C	5.822274	-0.478550	8.402135
C	5.632371	-0.470352	9.926856
C	4.132237	-0.535569	10.252880
C	5.929890	-2.982788	8.424286
C	4.239553	-3.040090	10.274528
C	6.338004	-1.692641	10.534165
C	5.739909	-2.981133	9.949087
H	2.459005	-1.864728	9.901091
H	3.278629	-2.728933	7.708920
H	3.204161	-0.969568	7.695340
H	5.359702	-1.767831	6.724680
H	6.888809	-0.413033	8.155012
H	5.339621	0.400654	7.958399
H	6.060008	0.447170	10.345578
H	3.981676	-0.510374	11.339002
H	3.619643	0.342853	9.842269
H	5.525649	-3.907991	7.996006
H	6.998420	-2.960481	8.178158
H	4.090227	-3.059792	11.360949
H	3.804705	-3.965696	9.878398
H	7.413683	-1.648401	10.324940
H	6.227092	-1.687597	11.625329
H	6.244569	-3.851452	10.382806
C	-4.777377	-3.381615	8.247944
C	-5.777403	-2.386940	7.638721
C	-7.165858	-2.599462	8.261603
C	-7.635024	-4.035380	7.981476
C	-6.639824	-5.034590	8.591446
C	-5.252622	-4.815584	7.968025
C	-7.081703	-2.379236	9.779894
C	-4.699272	-3.158823	9.766235
C	-6.556565	-4.807952	10.108824
C	-6.084761	-3.374039	10.394375
H	-3.788344	-3.228579	7.802407
H	-5.439519	-1.358353	7.813576
H	-5.828059	-2.523545	6.551649
H	-7.877073	-1.888900	7.826218
H	-8.634571	-4.193585	8.404244
H	-7.718502	-4.200292	6.900417
H	-6.976008	-6.057560	8.390049

H	-4.536418	-5.536446	8.380789
H	-5.293764	-4.994704	6.886728
H	-6.767462	-1.350714	9.994907
H	-8.071474	-2.509321	10.234058
H	-3.972652	-3.849742	10.210815
H	-4.342802	-2.143922	9.979925
H	-7.537001	-4.980090	10.569183
H	-5.863969	-5.529106	10.559616
H	-6.026522	-3.214137	11.476636
C	-11.215644	-4.711839	-1.560735
C	-10.686160	-3.414971	-0.929574
C	-9.176227	-3.291544	-1.186128
C	-8.454128	-4.496816	-0.564566
C	-8.977786	-5.796720	-1.194598
C	-10.488299	-5.913555	-0.938379
C	-8.917393	-3.269429	-2.700494
C	-10.951797	-4.685566	-3.074222
C	-8.719943	-5.768142	-2.709006
C	-9.442607	-4.566112	-3.336005
H	-12.292095	-4.797987	-1.376505
H	-11.211772	-2.549328	-1.350436
H	-10.885673	-3.413441	0.148918
H	-8.800987	-2.365984	-0.735772
H	-7.372456	-4.410158	-0.723733
H	-8.614417	-4.513616	0.520275
H	-8.462010	-6.654627	-0.749604
H	-10.871481	-6.847901	-1.366321
H	-10.684650	-5.955858	0.139876
H	-9.410544	-2.401487	-3.154713
H	-7.843722	-3.162131	-2.896837
H	-11.343922	-5.597761	-3.540200
H	-11.481337	-3.842420	-3.534062
H	-7.642950	-5.704232	-2.906030
H	-9.071191	-6.700186	-3.168227
H	-9.257199	-4.547069	-4.415594
C	-4.117364	-8.247799	-1.646044
C	-2.726176	-7.996599	-2.247773
C	-2.277717	-6.560223	-1.936867
C	-3.284901	-5.570307	-2.541925
C	-4.677751	-5.815123	-1.941142
C	-5.119912	-7.253527	-2.251682
C	-2.222713	-6.362519	-0.414146
C	-4.057188	-8.046096	-0.123992
C	-4.616662	-5.619810	-0.418363
C	-3.612850	-6.609741	0.192083
H	-4.435256	-9.272172	-1.869008
H	-2.003943	-8.713545	-1.838996
H	-2.751831	-8.156002	-3.332617
H	-1.285425	-6.383527	-2.366311
H	-2.964974	-4.540133	-2.343819
H	-3.319711	-5.687225	-3.631867
H	-5.394226	-5.108677	-2.374323
H	-6.122173	-7.435293	-1.844988
H	-5.187632	-7.400199	-3.336523

H	-1.491617	-7.049422	0.029135
H	-1.884991	-5.345913	-0.179010
H	-5.040399	-8.242710	0.320570
H	-3.358368	-8.763195	0.323420
H	-4.320540	-4.590429	-0.182771
H	-5.610140	-5.771980	0.020739
H	-3.570246	-6.468657	1.277684
C	-0.994995	-1.407023	-7.284601
C	-2.034186	-0.473544	-7.924354
C	-1.672711	0.989315	-7.623800
C	-1.654657	1.207037	-6.103037
C	-0.614550	0.277860	-5.458277
C	-0.978493	-1.183165	-5.764702
C	-0.281455	1.298144	-8.198103
C	0.394357	-1.092064	-7.860315
C	0.773976	0.588081	-6.038357
C	0.762102	0.368884	-7.558902
H	-1.254654	-2.449306	-7.499852
H	-2.071092	-0.637439	-9.008101
H	-3.033893	-0.701457	-7.535005
H	-2.414596	1.653064	-8.081325
H	-1.417250	2.253389	-5.875786
H	-2.647916	1.008904	-5.682282
H	-0.603809	0.434403	-4.374106
H	-0.254222	-1.858349	-5.292977
H	-1.959643	-1.423813	-5.337606
H	-0.285269	1.166664	-9.286925
H	-0.019843	2.345881	-8.006688
H	1.142879	-1.766166	-7.426381
H	0.401452	-1.266148	-8.943091
H	1.054384	1.623410	-5.809703
H	1.529398	-0.055260	-5.571007
H	1.752687	0.591047	-7.970770
C	1.036786	-4.131251	-1.670756
C	2.409013	-3.860059	-2.306392
C	2.839040	-2.413817	-2.015851
C	1.800755	-1.446142	-2.604083
C	0.426732	-1.710988	-1.969398
C	0.003044	-3.159144	-2.259706
C	2.925753	-2.204128	-0.496212
C	1.128583	-3.917479	-0.151934
C	0.519571	-1.503583	-0.449826
C	1.554612	-2.471292	0.143871
H	0.732010	-5.162636	-1.879241
H	3.153093	-4.561237	-1.909624
H	2.361089	-4.027759	-3.389228
H	3.817845	-2.222862	-2.469462
H	2.106888	-0.409103	-2.420535
H	1.742810	-1.571563	-3.692093
H	-0.311971	-1.020384	-2.390624
H	-0.986162	-3.355509	-1.828571
H	-0.087151	-3.314854	-3.341648
H	3.678959	-2.874863	-0.065362
H	3.250771	-1.180073	-0.275966

H	0.159557	-4.128078	0.316717
H	1.850156	-4.618941	0.284004
H	0.802791	-0.467482	-0.228245
H	-0.460630	-1.669966	0.013301
H	1.619818	-2.321604	1.227195
C	8.014367	-3.618631	-0.361800
C	6.524033	-3.725718	-0.004000
C	5.939543	-5.017585	-0.595950
C	6.100203	-4.995616	-2.123744
C	7.589474	-4.891700	-2.487310
C	8.169854	-3.600363	-1.890187
C	6.694193	-6.227032	-0.022775
C	8.763480	-4.831618	0.211296
C	8.340110	-6.101164	-1.908972
C	8.185097	-6.126014	-0.380705
H	8.429009	-2.696953	0.060918
H	6.396780	-3.719210	1.085264
H	5.981176	-2.855175	-0.391832
H	4.877102	-5.092139	-0.339149
H	5.668383	-5.904335	-2.560344
H	5.549465	-4.147508	-2.548310
H	7.701567	-4.875647	-3.576908
H	9.228734	-3.503962	-2.159345
H	7.656051	-2.727449	-2.310908
H	6.570819	-6.266963	1.066271
H	6.273185	-7.157150	-0.423358
H	9.833032	-4.756671	-0.019872
H	8.676131	-4.845933	1.304422
H	7.948037	-7.029377	-2.342006
H	9.401883	-6.049421	-2.179020
H	8.720441	-6.989238	0.029785
C	8.009535	1.721444	-2.057300
C	7.581405	0.281521	-2.379654
C	6.207306	-0.003903	-1.754086
C	6.294056	0.176852	-0.230849
C	6.718980	1.616518	0.097342
C	8.091584	1.897943	-0.533295
C	5.174055	0.979753	-2.324705
C	6.971653	2.700444	-2.627659
C	5.684756	2.596159	-0.478346
C	5.596379	2.421524	-2.002137
H	8.989373	1.922766	-2.503950
H	7.538521	0.135522	-3.465738
H	8.324688	-0.426639	-1.993660
H	5.903808	-1.030818	-1.985645
H	5.323518	-0.043748	0.229857
H	7.014498	-0.533555	0.192401
H	6.780697	1.742918	1.183741
H	8.415923	2.916966	-0.289653
H	8.844020	1.218277	-0.115117
H	5.086627	0.847417	-3.409914
H	4.184169	0.773593	-1.899903
H	7.276520	3.733745	-2.421889
H	6.916811	2.597783	-3.718169

H	4.703532	2.418468	-0.021789
H	5.966096	3.627517	-0.233165
H	4.858190	3.120367	-2.410727
C	-6.797130	-8.454720	4.416203
C	-8.303552	-8.569710	4.695581
C	-9.040506	-7.376362	4.068066
C	-8.504249	-6.070734	4.674871
C	-6.998214	-5.949093	4.395642
C	-6.266520	-7.146293	5.021965
C	-8.797967	-7.369039	2.550861
C	-6.560663	-8.445088	2.898001
C	-6.760708	-5.945869	2.877645
C	-7.292577	-7.251078	2.266260
H	-6.273650	-9.306222	4.864559
H	-8.691899	-9.509982	4.285939
H	-8.483985	-8.596573	5.777096
H	-10.114378	-7.460702	4.267998
H	-9.036873	-5.211676	4.249208
H	-8.688772	-6.054034	5.755855
H	-6.617982	-5.017880	4.829594
H	-5.187143	-7.061322	4.846771
H	-6.411052	-7.148460	6.109202
H	-9.194855	-8.287203	2.101094
H	-9.335115	-6.532160	2.088326
H	-5.486448	-8.383976	2.685105
H	-6.918254	-9.382625	2.455382
H	-7.262298	-5.084441	2.420298
H	-5.690156	-5.838964	2.664445
H	-7.122869	-7.246748	1.183934
C	-5.757290	4.384916	-2.721911
C	-6.781611	5.337544	-3.357404
C	-6.389031	6.793559	-3.062694
C	-6.357005	7.014393	-1.542610
C	-5.331649	6.066060	-0.902127
C	-5.726697	4.611914	-1.202696
C	-4.995532	7.073126	-3.646470
C	-4.365580	4.670647	-3.307102
C	-3.940888	6.347059	-1.491666
C	-3.966764	6.124628	-3.011568
H	-6.039117	3.347549	-2.932985
H	-6.828645	5.171915	-4.440495
H	-7.783198	5.130605	-2.961233
H	-7.120360	7.470977	-3.517171
H	-6.097267	8.056302	-1.319407
H	-7.351350	6.837171	-1.115146
H	-5.310920	6.224863	0.181570
H	-5.013118	3.923449	-0.733882
H	-6.709747	4.391958	-0.768864
H	-5.008863	6.939230	-4.734923
H	-4.711794	8.115852	-3.459257
H	-3.627973	3.982690	-2.876231
H	-4.368820	4.493951	-4.389473
H	-3.638371	7.377089	-1.267303
H	-3.195555	5.689797	-1.027499

H	-2.974556	6.325949	-3.430186
C	5.011803	-5.158926	3.596335
C	4.542501	-6.590886	3.296621
C	3.164411	-6.829442	3.932950
C	3.264199	-6.629702	5.453013
C	3.730321	-5.197827	5.758583
C	5.106779	-4.963299	5.117251
C	2.155605	-5.825789	3.353483
C	3.998241	-4.159795	3.017219
C	2.720404	-4.198231	5.174111
C	2.619155	-4.391817	3.653414
H	5.994423	-4.991031	3.142044
H	4.489842	-6.750937	2.212950
H	5.268055	-7.313777	3.688990
H	2.831540	-7.850753	3.717528
H	2.290421	-6.816923	5.921587
H	3.967057	-7.353682	5.882778
H	3.801268	-5.057881	6.842756
H	5.460330	-3.950281	5.344764
H	5.842437	-5.657508	5.541349
H	2.058800	-5.970942	2.270711
H	1.162692	-5.998549	3.786047
H	4.332519	-3.132502	3.206757
H	3.934798	-4.276260	1.928564
H	1.737103	-4.342328	5.637978
H	3.031320	-3.171676	5.403198
H	1.898317	-3.678699	3.238558
C	2.863568	-7.759909	-0.225679
C	1.367117	-7.876963	0.102218
C	0.807477	-9.181867	-0.485133
C	1.000033	-9.177716	-2.009388
C	2.495572	-9.063916	-2.343030
C	3.051000	-7.759516	-1.750588
C	1.561325	-10.376538	0.119241
C	3.611915	-8.958173	0.378545
C	3.245299	-10.258577	-1.733579
C	3.058406	-10.265548	-0.208696
H	3.260473	-6.828947	0.193708
H	1.216918	-7.857878	1.188408
H	0.824317	-7.016740	-0.308071
H	-0.259378	-9.263505	-0.249673
H	0.586124	-10.096063	-2.443178
H	0.450339	-8.340468	-2.456275
H	2.630410	-9.060595	-3.430162
H	4.114348	-7.656225	-1.998757
H	2.537916	-6.897056	-2.193179
H	1.415447	-10.403851	1.205892
H	1.157669	-11.315719	-0.278042
H	4.685336	-8.875748	0.168854
H	3.501725	-8.959471	1.469698
H	2.871235	-11.195983	-2.162723
H	4.311981	-10.199921	-1.982002
H	3.593148	-11.118239	0.223992
C	0.407253	0.737881	8.215760

C	-0.617300	1.730409	7.645003
C	-1.991205	1.485530	8.287944
C	-2.445400	0.049075	7.986696
C	-1.425596	-0.948070	8.558192
C	-0.053043	-0.696716	7.914868
C	-1.882717	1.676013	9.808712
C	0.509656	0.930843	9.736689
C	-1.318110	-0.751171	10.078196
C	-0.861151	0.683207	10.384778
H	1.385866	0.914003	7.755970
H	-0.290605	2.759931	7.834965
H	-0.685671	1.615230	6.556420
H	-2.719946	2.194607	7.879981
H	-3.434864	-0.131901	8.423806
H	-2.546085	-0.094992	6.904124
H	-1.751148	-1.971413	8.341783
H	0.680419	-1.415558	8.299918
H	-0.111210	-0.854379	6.831015
H	-1.578935	2.704318	10.039236
H	-2.862231	1.522649	10.277612
H	1.253692	0.241436	10.153975
H	0.855823	1.946158	9.964860
H	-2.287596	-0.946568	10.552257
H	-0.607559	-1.471415	10.501672
H	-0.785608	0.821882	11.468896
C	-6.960648	-0.661010	2.627879
C	-7.406277	-2.103701	2.343905
C	-8.789842	-2.351399	2.964667
C	-8.714189	-2.127067	4.482692
C	-8.271822	-0.684416	4.772478
C	-6.889763	-0.440844	4.146754
C	-9.803864	-1.370684	2.355775
C	-7.979369	0.315124	2.019341
C	-9.286829	0.292151	4.158628
C	-9.364017	0.073942	2.639855
H	-5.974086	-0.486605	2.184722
H	-7.441494	-2.281026	1.262218
H	-6.676614	-2.810467	2.757459
H	-9.105805	-3.380364	2.760496
H	-9.691851	-2.320455	4.940550
H	-8.007730	-2.834663	4.933336
H	-8.218055	-0.526933	5.855232
H	-6.553182	0.580298	4.363428
H	-6.150833	-1.118310	4.591726
H	-9.883434	-1.533707	1.274142
H	-10.800346	-1.550428	2.777141
H	-7.661715	1.349692	2.197560
H	-8.025888	0.181151	0.931846
H	-10.274529	0.141675	4.610982
H	-8.993080	1.326265	4.376075
H	-10.088515	0.770658	2.204036
C	-10.907898	0.331410	-2.684024
C	-9.537746	0.647565	-3.303149
C	-9.157295	2.106044	-3.004737

C	-10.218966	3.041641	-3.603036
C	-11.591104	2.731775	-2.984871
C	-11.965161	1.271583	-3.282979
C	-9.094827	2.314775	-1.483777
C	-10.840468	0.544386	-1.163813
C	-11.522439	2.938417	-1.463911
C	-10.464073	2.002670	-0.860185
H	-11.177305	-0.708638	-2.898099
H	-8.776377	-0.030562	-2.899180
H	-9.567821	0.480997	-4.386801
H	-8.179889	2.329072	-3.446576
H	-9.948095	4.087443	-3.413869
H	-10.260341	2.917025	-4.691895
H	-12.346482	3.399562	-3.413255
H	-12.952531	1.042871	-2.863746
H	-12.037874	1.115655	-4.366205
H	-8.325697	1.667285	-1.045561
H	-8.805092	3.348112	-1.257721
H	-11.807650	0.301998	-0.706862
H	-10.102035	-0.134832	-0.720939
H	-11.274877	3.982464	-1.236981
H	-12.502132	2.739882	-1.012524
H	-10.416158	2.151783	0.224121
C	-3.971911	0.812586	-1.268017
C	-5.472256	0.697479	-0.957698
C	-6.030440	-0.598110	-1.566659
C	-5.821380	-0.576491	-3.088590
C	-4.321896	-0.464565	-3.404702
C	-3.767982	0.830475	-2.790703
C	-5.287707	-1.802862	-0.968569
C	-3.234690	-0.395736	-0.670179
C	-3.583339	-1.669352	-2.801581
C	-3.786754	-1.693821	-1.278994
H	-3.576048	1.736907	-0.833238
H	-5.634132	0.704165	0.126960
H	-6.007323	1.564663	-1.363465
H	-7.100083	-0.678381	-1.343709
H	-6.234042	-1.487960	-3.537803
H	-6.363031	0.268185	-3.531301
H	-4.175286	-0.448776	-4.490200
H	-2.701626	0.932561	-3.026138
H	-4.272976	1.700166	-3.228420
H	-5.445442	-1.842592	0.116040
H	-5.690590	-2.735640	-1.381418
H	-2.158757	-0.315012	-0.867284
H	-3.356690	-0.409647	0.419629
H	-3.956280	-2.600090	-3.245946
H	-2.513816	-1.611907	-3.037769
H	-3.259958	-2.553710	-0.850827
C	0.810195	3.738702	-3.420579
C	1.363334	5.031884	-2.802272
C	1.196196	4.990138	-1.275421
C	-0.294583	4.853017	-0.930132
C	-0.852313	3.559392	-1.543629

C	-0.679576	3.604608	-3.069756
C	1.961558	3.782632	-0.712445
C	1.577061	2.534622	-2.852200
C	-0.081503	2.355494	-0.980409
C	1.409771	2.486278	-1.325723
H	0.930105	3.770608	-4.509024
H	2.422118	5.150785	-3.062216
H	0.836120	5.900771	-3.214623
H	1.591726	5.912817	-0.836666
H	-0.429003	4.842210	0.158294
H	-0.850352	5.719099	-1.309445
H	-1.915306	3.464004	-1.296659
H	-1.092086	2.694685	-3.522351
H	-1.242502	4.448369	-3.487080
H	3.031485	3.877694	-0.933929
H	1.865811	3.752783	0.379697
H	1.205148	1.605762	-3.301530
H	2.639963	2.608536	-3.112076
H	-0.212410	2.300423	0.107088
H	-0.483847	1.423398	-1.395475
H	1.957775	1.627386	-0.922925
C	-1.767600	3.415303	2.589819
C	-2.209318	1.974802	2.289112
C	-3.590986	1.715303	2.909279
C	-3.513258	1.921394	4.429784
C	-3.074791	3.361775	4.736308
C	-1.694626	3.617204	4.111130
C	-4.609116	2.700211	2.314169
C	-2.790415	4.395612	1.995015
C	-4.093903	4.342593	4.136191
C	-4.173185	4.142629	2.615016
H	-0.782385	3.598128	2.147059
H	-2.245960	1.810533	1.205412
H	-1.476740	1.265317	2.692737
H	-3.904163	0.687926	2.693172
H	-4.489488	1.719436	4.886998
H	-2.803814	1.210557	4.870528
H	-3.019537	3.506248	5.820800
H	-1.360784	4.636671	4.339609
H	-0.952814	2.936663	4.546511
H	-4.690154	2.550106	1.230778
H	-5.604274	2.512281	2.735094
H	-2.475611	5.428913	2.185238
H	-2.838501	4.274728	0.906056
H	-5.080312	4.183580	4.588440
H	-3.802931	5.374890	4.365677
H	-4.900609	4.842351	2.188996
C	-2.143250	-3.138966	2.236734
C	-1.581810	-1.845119	2.846103
C	-1.781599	-1.859442	4.369531
C	-3.281362	-1.966701	4.685214
C	-3.847499	-3.260839	4.080564
C	-3.642071	-3.243127	2.557924
C	-1.048359	-3.069971	4.967670

```
C     -1.408563     -4.346006      2.840253
C     -3.108759     -4.467845      4.679006
C     -1.608606     -4.366930      4.363407
H     -2.000028     -3.126622      1.150712
H     -0.515981     -1.747669      2.606850
H     -2.085767     -0.974556      2.408951
H     -1.380097     -0.936341      4.801927
H     -3.438738     -1.957717      5.770576
H     -3.814602     -1.097945      4.280527
H     -4.916875     -3.334923      4.306386
H     -4.060045     -4.153422      2.111130
H     -4.182039     -2.397183      2.115440
H      0.027408     -2.995913      4.767478
H     -1.167470     -3.080591      6.057868
H     -1.786338     -5.275841      2.397884
H     -0.339365     -4.293598      2.601867
H     -3.263338     -4.503185      5.764214
H     -3.517726     -5.399867      4.270295
H     -1.083501     -5.227992      4.791293
C      3.401669      2.241569      4.330696
C      1.902691      2.135924      4.650832
C      1.331363      0.846202      4.041610
C      1.530263      0.871216      2.518369
C      3.028319      0.973703      2.192437
C      3.595409      2.262891      2.806725
C      2.069693     -0.365594      4.630857
C      4.134461      1.026243      4.919718
C      3.762507     -0.238114      2.786743
C      3.569235     -0.266019      4.310592
H      3.806913      3.161720      4.765678
H      1.748210      2.140281      5.736580
H      1.370961      3.008103      4.251433
H      0.262718      0.772672      4.271563
H      1.108214     -0.035921      2.069109
H      0.991543      1.721050      2.082002
H      3.167687      0.991931      1.106024
H      4.660834      2.358299      2.564377
H      3.093551      3.137469      2.375180
H      1.919027     -0.407681      5.716382
H      1.657519     -1.294222      4.217825
H      5.209573      1.100100      4.715567
H      4.019745      1.009708      6.010280
H      3.380075     -1.164811      2.342000
H      4.830783     -0.187366      2.543481
H      4.092897     -1.130916      4.732470
```

Adamantane$_{31}$

```
         806
E=-1423.1078
C     -1.639467     -5.910548      9.749712
C     -1.443237     -5.930031      8.226000
C      0.055852     -5.866986      7.894777
C      0.650772     -4.573546      8.472297
```

C	0.459970	-4.550267	9.996751
C	-1.040126	-4.617086	10.322622
C	0.766650	-7.077053	8.520313
C	-0.924547	-7.120883	10.370137
C	1.169291	-5.763892	10.617004
C	0.575791	-7.060316	10.044913
H	-2.708755	-5.955332	9.983649
H	-1.885027	-6.839360	7.800942
H	-1.965300	-5.080484	7.769192
H	0.193423	-5.881566	6.807970
H	1.717225	-4.507049	8.225087
H	0.165470	-3.700563	8.019265
H	0.884333	-3.627068	10.406225
H	-1.191391	-4.581201	11.408346
H	-1.555385	-3.744644	9.902705
H	0.365713	-8.007953	8.101372
H	1.835241	-7.053746	8.274543
H	-1.074429	-7.129884	11.456623
H	-1.356108	-8.051956	9.983333
H	2.244937	-5.718249	10.407912
H	1.057739	-5.747972	11.707997
H	1.083077	-7.924445	10.487850
C	-2.023881	-10.940634	4.028441
C	-0.648820	-10.719996	3.379513
C	-0.168464	-9.286806	3.655519
C	-1.178916	-8.289387	3.068504
C	-2.555570	-8.503629	3.716447
C	-3.029652	-9.938941	3.440637
C	-0.061964	-9.069011	5.172758
C	-1.912172	-10.718932	5.544791
C	-2.443024	-8.288320	5.233586
C	-1.435770	-9.285627	5.826093
H	-2.364562	-11.962783	3.830306
H	0.074848	-11.442668	3.775469
H	-0.711459	-10.893924	2.298412
H	0.812279	-9.131912	3.192445
H	-0.836903	-7.261814	3.241649
H	-1.250208	-8.420764	1.981990
H	-3.274423	-7.791883	3.296070
H	-4.021160	-10.099059	3.881362
H	-3.134132	-10.099470	2.360686
H	0.672126	-9.761312	5.602488
H	0.298801	-8.054776	5.382543
H	-2.883668	-10.893676	6.022972
H	-1.210675	-11.440851	5.980101
H	-2.123620	-7.260643	5.444958
H	-3.424264	-8.418541	5.706000
H	-1.356453	-9.130273	6.907677
C	3.436817	7.572527	2.608210
C	2.987195	6.146848	2.253438
C	1.603286	5.872375	2.862054
C	1.680304	6.021069	4.389276
C	2.126668	7.446428	4.749853
C	3.509057	7.717031	4.136077

C	0.591580	6.884711	2.303111
C	2.420400	8.580282	2.049396
C	1.113963	8.454870	4.185753
C	1.035427	8.312332	2.658088
H	4.423618	7.766052	2.173604
H	2.950958	6.023391	1.164320
H	3.715175	5.418547	2.631027
H	1.284473	4.855620	2.607390
H	0.702360	5.807833	4.837620
H	2.385098	5.290164	4.803884
H	2.181402	7.549966	5.839042
H	3.848473	8.725299	4.402829
H	4.246400	7.016413	4.546491
H	0.511029	6.775653	1.214784
H	-0.405166	6.686958	2.715695
H	2.740906	9.603914	2.278417
H	2.372975	8.500442	0.956631
H	0.126093	8.284792	4.630730
H	1.410583	9.476170	4.453947
H	0.312571	9.031685	2.257760
C	-1.232212	8.137903	8.912843
C	-1.017842	8.157883	7.391584
C	-1.586740	6.872887	6.770220
C	-0.869982	5.655205	7.373507
C	-1.083750	5.628588	8.894856
C	-0.516506	6.916941	9.510751
C	-3.089592	6.781467	7.076604
C	-2.736120	8.043662	9.213386
C	-2.587866	5.540815	9.195962
C	-3.308997	6.758100	8.597271
H	-0.825772	9.054663	9.353828
H	-1.506837	9.036503	6.953814
H	0.051636	8.244015	7.164099
H	-1.433320	6.889538	5.685567
H	-1.253609	4.731771	6.922999
H	0.201883	5.697653	7.145125
H	-0.571462	4.760011	9.322856
H	-0.645018	6.901745	10.599888
H	0.561750	6.981589	9.320587
H	-3.617344	7.634742	6.633621
H	-3.511704	5.877271	6.621539
H	-2.904086	8.049271	10.297246
H	-3.256651	8.919648	8.807856
H	-3.001755	4.615219	8.777888
H	-2.753047	5.500569	10.279503
H	-4.381270	6.693524	8.812470
C	-6.350062	4.052574	8.974344
C	-7.851602	3.965936	9.288121
C	-8.436703	2.683701	8.676171
C	-8.231065	2.706480	7.153790
C	-6.730463	2.789973	6.834213
C	-6.149629	4.071727	7.451210
C	-7.716337	1.462501	9.268231
C	-5.635278	2.827914	9.566154

C	-6.014260	1.568803	7.431315
C	-6.214310	1.543062	8.954326
H	-5.934994	4.967414	9.411262
H	-8.010603	3.972046	10.373207
H	-8.370507	4.844883	8.886695
H	-9.507159	2.623721	8.901591
H	-8.662712	1.804870	6.702527
H	-8.757087	3.563180	6.715362
H	-6.586285	2.806639	5.748403
H	-5.082062	4.153627	7.213388
H	-6.638498	4.952699	7.017766
H	-7.872112	1.422122	10.353099
H	-8.138536	0.539269	8.853232
H	-4.558462	2.888134	9.366564
H	-5.754804	2.812627	10.656218
H	-6.406562	0.647131	6.984730
H	-4.944407	1.606011	7.192578
H	-5.703475	0.671495	9.378198
C	5.581924	4.855598	8.192543
C	5.137081	3.421909	7.865076
C	3.770156	3.142641	8.508876
C	3.881150	3.310231	10.032044
C	4.322872	4.743642	10.365394
C	5.688193	5.018965	9.716517
C	2.736703	4.139349	7.961757
C	4.543806	5.847687	7.645717
C	3.288337	5.736385	9.813189
C	3.175759	5.574932	8.289541
H	6.556619	5.052536	7.732875
H	5.076766	3.285142	6.778619
H	5.880140	2.704560	8.234164
H	3.454714	2.120170	8.273643
H	2.915804	3.093826	10.505471
H	4.601908	2.590518	10.438708
H	4.401859	4.860654	11.451726
H	6.024589	6.033276	9.963455
H	6.441120	4.329770	10.117875
H	2.631977	4.016602	6.876952
H	1.751595	3.937835	8.399692
H	4.860285	6.876743	7.855196
H	4.471828	5.754390	6.555362
H	2.312601	5.563007	10.282974
H	3.581865	6.763369	10.062410
H	2.437379	6.283108	7.897708
C	-3.486282	10.739812	3.371387
C	-4.989863	10.588266	3.648626
C	-5.518038	9.316933	2.966207
C	-5.283133	9.417370	1.451222
C	-3.780265	9.566016	1.168025
C	-3.256436	10.836490	1.855372
C	-4.767793	8.094425	3.517021
C	-2.741489	9.513673	3.921673
C	-3.034280	8.343163	1.723786
C	-3.263512	8.239927	3.239385

H	-3.111833	11.646779	3.858531
H	-5.170487	10.538569	4.729199
H	-5.530763	11.466992	3.276847
H	-6.590045	9.210596	3.165660
H	-5.674320	8.523722	0.950001
H	-5.829642	10.275368	1.041316
H	-3.615225	9.637986	0.087455
H	-2.187828	10.965190	1.644475
H	-3.766759	11.719735	1.452351
H	-4.943582	7.998434	4.595316
H	-5.149512	7.177820	3.051231
H	-1.663647	9.619262	3.748165
H	-2.882028	9.443711	5.007082
H	-3.385553	7.430589	1.227163
H	-1.961824	8.427364	1.509979
H	-2.731375	7.367253	3.633747
C	-8.968134	5.462682	1.243234
C	-8.418891	6.745555	1.886004
C	-8.614859	6.688168	3.408915
C	-10.112645	6.556580	3.724774
C	-10.666572	5.273240	3.086723
C	-10.464999	5.334053	1.564679
C	-7.867475	5.469833	3.972713
C	-8.219327	4.247704	1.812501
C	-9.913724	4.058476	3.650907
C	-8.415480	4.183725	3.334961
H	-8.827660	5.505732	0.157636
H	-7.354725	6.860687	1.647203
H	-8.933037	7.622167	1.473502
H	-8.222064	7.603514	3.865140
H	-10.267500	6.534717	4.810315
H	-10.655977	7.430204	3.344738
H	-11.734562	5.181788	3.312734
H	-10.874435	4.431721	1.094440
H	-11.014909	6.185818	1.146261
H	-6.793031	5.560638	3.772308
H	-7.983848	5.428649	5.062481
H	-8.588350	3.326147	1.346075
H	-7.151316	4.317825	1.573347
H	-10.065315	3.992341	4.735095
H	-10.313833	3.133498	3.218133
H	-7.880308	3.317082	3.738411
C	10.194227	-1.071402	3.537678
C	9.706059	-2.490972	3.210421
C	8.331595	-2.728751	3.854831
C	8.448285	-2.564603	5.377948
C	8.933328	-1.145217	5.711089
C	10.306067	-0.911331	5.061603
C	7.328533	-1.701225	3.308161
C	9.186362	-0.048352	2.991303
C	7.929062	-0.121620	5.159333
C	7.810975	-0.279586	3.635736
H	11.174231	-0.904047	3.077576
H	9.641151	-2.625847	2.123993

H	10.427234	-3.230480	3.579186
H	7.985252	-3.741208	3.619747
H	7.477042	-2.751699	5.851805
H	9.147115	-3.305799	5.784298
H	9.016302	-1.030652	6.797384
H	10.673112	0.092334	5.308382
H	11.037972	-1.622997	5.462634
H	7.219660	-1.820743	2.223404
H	6.337973	-1.872839	3.746534
H	9.533929	0.970655	3.200632
H	9.111111	-0.139424	1.900981
H	6.948735	-0.265395	5.629552
H	8.253643	0.896010	5.408414
H	7.094189	0.450610	3.244224
C	8.635338	6.362597	4.244758
C	7.153511	6.260714	4.637684
C	6.551842	4.968678	4.063584
C	6.676733	4.985569	2.532386
C	8.157355	5.084268	2.133732
C	8.755034	6.375801	2.713100
C	7.316453	3.758894	4.622676
C	9.394402	5.149289	4.803911
C	8.918073	3.874493	2.698078
C	8.798831	3.854706	4.229625
H	9.062246	7.284404	4.654797
H	7.051823	6.270877	5.729604
H	6.604063	7.131573	4.259975
H	5.495511	4.897838	4.345419
H	6.232362	4.076727	2.108833
H	6.118487	5.833904	2.118208
H	8.243940	5.096706	1.041763
H	9.807575	6.468459	2.418928
H	8.233869	7.248851	2.301824
H	7.218513	3.722607	5.714430
H	6.883676	2.828732	4.234942
H	10.458444	5.220580	4.547534
H	9.332642	5.138530	5.898822
H	8.513469	2.946059	2.277219
H	9.973366	3.922484	2.403086
H	9.341317	2.991256	4.630139
C	3.531463	-1.820092	9.667508
C	3.728618	-1.824096	8.143796
C	5.228068	-1.761252	7.814170
C	5.825771	-0.475389	8.405571
C	5.634053	-0.467610	9.930068
C	4.133590	-0.534171	10.254302
C	5.935513	-2.979538	8.427432
C	4.243033	-3.038603	10.275659
C	6.340016	-1.689395	10.538009
C	5.743722	-2.978302	9.952007
H	2.461920	-1.864709	9.900307
H	3.284884	-2.727841	7.708965
H	3.208921	-0.968538	7.695591
H	5.366296	-1.764785	6.727354

H	6.892541	-0.408914	8.159724
H	5.342888	0.403474	7.961410
H	6.060405	0.450209	10.349449
H	3.981721	-0.509288	11.340249
H	3.620729	0.343879	9.843230
H	5.532575	-3.905016	7.998518
H	7.004314	-2.956271	8.182575
H	4.092436	-3.058615	11.361899
H	3.809450	-3.964516	9.878859
H	7.415904	-1.644195	10.330066
H	6.227806	-1.684630	11.629041
H	6.248616	-3.848260	10.386179
C	-4.774010	-3.381108	8.252058
C	-5.772103	-2.384748	7.642417
C	-7.161472	-2.595816	8.263753
C	-7.632242	-4.030945	7.982278
C	-6.638983	-5.031835	8.592657
C	-5.250858	-4.814279	7.970785
C	-7.078577	-2.376620	9.782263
C	-4.697163	-3.159337	9.770563
C	-6.556975	-4.806224	10.110257
C	-6.083575	-3.373106	10.397158
H	-3.784322	-3.229106	7.807623
H	-5.433043	-1.356713	7.818240
H	-5.821828	-2.520629	6.555212
H	-7.871306	-1.884054	7.828073
H	-8.632429	-4.188089	8.403930
H	-7.714833	-4.195094	6.901034
H	-6.976308	-6.054239	8.390298
H	-4.536026	-5.536334	8.383844
H	-5.291131	-4.992692	6.889339
H	-6.763202	-1.348642	9.998219
H	-8.068982	-2.505675	10.235337
H	-3.971908	-3.851481	10.215466
H	-4.339575	-2.145036	9.985231
H	-7.538107	-4.977348	10.569511
H	-5.865791	-5.528563	10.561318
H	-6.026231	-3.213936	11.479575
C	-11.212505	-4.718009	-1.556385
C	-10.680502	-3.421438	-0.926733
C	-9.171201	-3.298946	-1.187424
C	-8.448141	-4.504634	-0.567789
C	-8.974318	-5.804244	-1.196326
C	-10.484195	-5.920143	-0.935969
C	-8.916500	-3.277054	-2.702495
C	-10.952787	-4.691961	-3.070589
C	-8.720604	-5.775888	-2.711437
C	-9.444246	-4.573443	-3.336509
H	-12.288500	-4.803490	-1.369205
H	-11.206734	-2.555491	-1.346193
H	-10.877060	-3.419740	0.152301
H	-8.794163	-2.373597	-0.738139
H	-7.366856	-4.418645	-0.729920
H	-8.605470	-4.521290	0.517488

H	-8.457850	-6.662447	-0.752709
H	-10.869119	-6.854272	-1.362819
H	-10.677619	-5.962280	0.142822
H	-9.410361	-2.408830	-3.155400
H	-7.843305	-3.170423	-2.901781
H	-11.346743	-5.603936	-3.535451
H	-11.483067	-3.848512	-3.529015
H	-7.644115	-5.712646	-2.911411
H	-9.073678	-6.707737	-3.169653
H	-9.261782	-4.554560	-4.416601
C	-4.149870	-8.251845	-1.627524
C	-2.763608	-8.017125	-2.247016
C	-2.296651	-6.584378	-1.946784
C	-3.300903	-5.586238	-2.543142
C	-4.688773	-5.814560	-1.924601
C	-5.149449	-7.249399	-2.224535
C	-2.221067	-6.381930	-0.425570
C	-4.069081	-8.045454	-0.107057
C	-4.607128	-5.614566	-0.403394
C	-3.606182	-6.612666	0.198394
H	-4.480969	-9.273655	-1.842899
H	-2.043857	-8.740056	-1.844449
H	-2.804118	-8.180047	-3.330883
H	-1.307902	-6.419438	-2.388888
H	-2.968012	-4.558736	-2.352669
H	-3.350192	-5.706601	-3.632150
H	-5.403172	-5.102273	-2.351601
H	-6.148495	-7.419386	-1.805040
H	-5.231886	-7.399156	-3.307935
H	-1.491725	-7.074791	0.011282
H	-1.870072	-5.368049	-0.198237
H	-5.048772	-8.230351	0.350127
H	-3.372271	-8.768161	0.334428
H	-4.297590	-4.587479	-0.175146
H	-5.596693	-5.754934	0.048294
H	-3.548905	-6.468235	1.282879
C	-1.028473	-1.393778	-7.314612
C	-2.049367	-0.431274	-7.940668
C	-1.659183	1.019883	-7.619960
C	-1.636376	1.215969	-6.096320
C	-0.614480	0.257679	-5.465199
C	-1.007097	-1.191515	-5.791750
C	-0.262325	1.309368	-8.190728
C	0.366603	-1.098108	-7.886705
C	0.779675	0.548688	-6.041722
C	0.763017	0.351004	-7.565168
H	-1.308586	-2.427666	-7.544222
H	-2.089828	-0.579276	-9.026573
H	-3.053226	-0.644959	-7.553941
H	-2.388060	1.704365	-8.067744
H	-1.378427	2.254194	-5.854642
H	-2.633194	1.031490	-5.677778
H	-0.600324	0.398838	-4.378956
H	-0.296051	-1.887281	-5.329917

H	-1.992639	-1.418822	-5.367467
H	-0.269065	1.193193	-9.281275
H	0.019840	2.349003	-7.984877
H	1.101903	-1.792740	-7.462657
H	0.369935	-1.257172	-8.971806
H	1.080401	1.575031	-5.798820
H	1.522488	-0.115793	-5.583837
H	1.757635	0.559435	-7.974487
C	1.034667	-4.108079	-1.672393
C	2.411096	-3.835623	-2.298330
C	2.844340	-2.393557	-1.992223
C	1.812341	-1.417055	-2.576920
C	0.434142	-1.683150	-1.951886
C	0.007230	-3.127153	-2.257742
C	2.923761	-2.198154	-0.470284
C	1.119189	-3.908602	-0.151206
C	0.519682	-1.490050	-0.430002
C	1.548407	-2.466627	0.160142
H	0.727603	-5.136480	-1.891968
H	3.150775	-4.542899	-1.904165
H	2.368308	-3.993189	-3.382901
H	3.826135	-2.201699	-2.438939
H	2.120911	-0.382775	-2.382222
H	1.759699	-1.532263	-3.666328
H	-0.300067	-0.986240	-2.370576
H	-0.984866	-3.324179	-1.833606
H	-0.077793	-3.272597	-3.341530
H	3.672486	-2.875333	-0.041711
H	3.250979	-1.177259	-0.238911
H	0.147030	-4.120275	0.310418
H	1.836156	-4.616447	0.282006
H	0.805136	-0.456982	-0.197401
H	-0.463478	-1.657421	0.026448
H	1.608415	-2.327134	1.245126
C	8.006672	-3.613655	-0.363928
C	6.514975	-3.719807	-0.011570
C	5.929933	-5.007266	-0.612507
C	6.095618	-4.977225	-2.139627
C	7.586264	-4.874210	-2.497771
C	8.167179	-3.587301	-1.891678
C	6.680378	-6.221306	-0.043535
C	8.751574	-4.831223	0.204927
C	8.332679	-6.088288	-1.923650
C	8.172633	-6.121224	-0.396055
H	8.421707	-2.695118	0.065194
H	6.384183	-3.719029	1.077294
H	5.975064	-2.846095	-0.396375
H	4.866518	-5.081164	-0.359576
H	5.663475	-5.882693	-2.582615
H	5.547902	-4.125731	-2.561314
H	7.701942	-4.852396	-3.586895
H	9.227115	-3.491482	-2.156857
H	7.656434	-2.711094	-2.309265
H	6.553375	-6.266974	1.044868

H	6.258888	-7.148391	-0.450590
H	9.822016	-4.757087	-0.022347
H	8.660632	-4.851368	1.297668
H	7.940234	-7.013345	-2.363051
H	9.395425	-6.037127	-2.189955
H	8.704972	-6.987726	0.011424
C	8.014640	1.735144	-2.048721
C	7.589350	0.294893	-2.373357
C	6.214767	0.006521	-1.750206
C	6.298905	0.185688	-0.226635
C	6.720982	1.625670	0.103834
C	8.094083	1.910046	-0.524393
C	5.180784	0.989140	-2.321283
C	6.976033	2.713099	-2.619550
C	5.686039	2.604279	-0.472319
C	5.600265	2.431230	-1.996439
H	8.994827	1.938568	-2.493649
H	7.548356	0.150057	-3.459669
H	8.333198	-0.412494	-1.987034
H	5.913296	-1.020624	-1.983390
H	5.328029	-0.037015	0.232342
H	7.019859	-0.524025	0.196907
H	6.780840	1.750937	1.190468
H	8.416390	2.929319	-0.279103
H	8.846989	1.231131	-0.105840
H	5.095222	0.857894	-3.406774
H	4.190590	0.780886	-1.898224
H	7.278902	3.746661	-2.412145
H	6.923018	2.611587	-3.710258
H	4.704413	2.424472	-0.017459
H	5.965324	3.635815	-0.225542
H	4.861560	3.129333	-2.405362
C	-6.799699	-8.458399	4.427834
C	-8.306415	-8.568732	4.707507
C	-9.039963	-7.373824	4.078972
C	-8.499822	-6.069201	4.684492
C	-6.993474	-5.952215	4.404963
C	-6.265196	-7.150961	5.032310
C	-8.797596	-7.368622	2.561730
C	-6.563397	-8.450871	2.909595
C	-6.756152	-5.951099	2.886934
C	-7.291905	-7.255320	2.276832
H	-6.278649	-9.311007	4.876919
H	-8.697556	-9.508248	4.298789
H	-8.486788	-8.594061	5.789069
H	-10.114051	-7.454844	4.279115
H	-9.029990	-5.208990	4.258095
H	-8.684158	-6.050956	5.765484
H	-6.610470	-5.021713	4.838000
H	-5.185599	-7.069304	4.856904
H	-6.409596	-7.151694	6.119567
H	-9.197219	-8.286043	2.112869
H	-9.332359	-6.530611	2.098482
H	-5.489036	-8.393093	2.696509

H	-6.923779	-9.387773	2.467894
H	-7.255285	-5.088637	2.428847
H	-5.685319	-5.847518	2.673501
H	-7.122323	-7.252493	1.194481
C	-5.754814	4.375700	-2.725897
C	-6.778610	5.327532	-3.363423
C	-6.386422	6.783908	-3.069979
C	-6.355846	7.006451	-1.550114
C	-5.331020	6.058919	-0.907602
C	-5.725671	4.604405	-1.206910
C	-4.992396	7.062930	-3.652755
C	-4.362575	4.660883	-3.310096
C	-3.939726	6.339366	-1.496145
C	-3.964153	6.115227	-3.015820
H	-6.036361	3.338074	-2.936072
H	-6.824611	5.160683	-4.446372
H	-7.780554	5.120959	-2.967964
H	-7.117376	7.460757	-3.525905
H	-6.096400	8.048630	-1.327837
H	-7.350579	6.829630	-1.123389
H	-5.311325	6.218941	0.175936
H	-5.012480	3.916524	-0.736651
H	-6.709112	4.384859	-0.773757
H	-5.004691	6.927811	-4.741070
H	-4.708916	8.105888	-3.466446
H	-3.625321	3.973470	-2.877759
H	-4.364782	4.482972	-4.392271
H	-3.637501	7.369672	-1.272654
H	-3.194780	5.682685	-1.030538
H	-2.971566	6.316157	-3.433729
C	5.016069	-5.164461	3.601247
C	4.549476	-6.598740	3.308465
C	3.171910	-6.836872	3.946086
C	3.271472	-6.629644	5.465162
C	3.734885	-5.195430	5.763800
C	5.110826	-4.961352	5.121196
C	2.161126	-5.837944	3.361919
C	4.000538	-4.170064	3.017452
C	2.722998	-4.200585	5.174647
C	2.621964	-4.401665	3.654908
H	5.998319	-4.996872	3.146046
H	4.497013	-6.764094	2.225581
H	5.276451	-7.318349	3.704220
H	2.840971	-7.859840	3.735608
H	2.298101	-6.816474	5.934737
H	3.975757	-7.350207	5.898320
H	3.805675	-5.050143	6.847280
H	5.462462	-3.946579	5.343802
H	5.847854	-5.652108	5.548540
H	2.064489	-5.988480	2.279867
H	1.168590	-6.010524	3.795417
H	4.332870	-3.141235	3.202018
H	3.937207	-4.291877	1.929376
H	1.740022	-4.344333	5.639310

H	3.031973	-3.172349	5.398767
H	1.899721	-3.691927	3.236713
C	2.872864	-7.741842	-0.212385
C	1.367878	-7.853326	0.076014
C	0.814761	-9.145716	-0.544261
C	1.045772	-9.120845	-2.062965
C	2.549971	-9.012526	-2.357177
C	3.098745	-7.720684	-1.731927
C	1.545305	-10.354162	0.061342
C	3.597890	-8.953842	0.392877
C	3.276267	-10.221008	-1.746603
C	3.050882	-10.248747	-0.227170
H	3.265108	-6.819797	0.230432
H	1.190423	-7.848919	1.158244
H	0.841229	-6.983543	-0.335169
H	-0.258210	-9.223407	-0.336934
H	0.636976	-10.029952	-2.520422
H	0.513007	-8.273458	-2.511241
H	2.712239	-8.994410	-3.440407
H	4.168672	-7.621135	-1.951728
H	2.602633	-6.848403	-2.174618
H	1.371856	-10.396184	1.143463
H	1.145701	-11.284699	-0.359670
H	4.676778	-8.875781	0.211485
H	3.460172	-8.970163	1.480777
H	2.907062	-11.149513	-2.198692
H	4.349242	-10.166108	-1.967227
H	3.568959	-11.111266	0.206311
C	0.409583	0.738485	8.218851
C	-0.613864	1.731632	7.647189
C	-1.988591	1.487279	8.288571
C	-2.443151	0.051109	7.986513
C	-1.424456	-0.946654	8.558908
C	-0.051078	-0.695823	7.917142
C	-1.881677	1.677378	9.809499
C	0.510413	0.931066	9.739934
C	-1.318539	-0.750138	10.079071
C	-0.861223	0.683953	10.386467
H	1.388784	0.914234	7.760171
H	-0.286880	2.760955	7.837733
H	-0.681099	1.616723	6.558507
H	-2.716541	2.196797	7.879965
H	-3.433181	-0.129483	8.422500
H	-2.542720	-0.092674	6.903800
H	-1.750265	-1.969792	8.341919
H	0.681615	-1.415105	8.302839
H	-0.108133	-0.853223	6.833191
H	-1.577650	2.705485	10.040580
H	-2.861777	1.524386	10.277293
H	1.253658	0.241209	10.157883
H	0.856821	1.946164	9.968705
H	-2.288638	-0.945168	10.552028
H	-0.608801	-1.470817	10.503168
H	-0.786800	0.822355	11.470697

C	-6.953458	-0.664625	2.622885
C	-7.397991	-2.107706	2.339178
C	-8.781505	-2.356245	2.959719
C	-8.706322	-2.131483	4.477704
C	-8.265051	-0.688443	4.767223
C	-6.883040	-0.444031	4.141719
C	-9.796107	-1.376410	2.350378
C	-7.972756	0.310625	2.013898
C	-9.280635	0.287242	4.152924
C	-9.357357	0.068602	2.634189
H	-5.966931	-0.489621	2.179886
H	-7.432861	-2.285325	1.257527
H	-6.667905	-2.813845	2.753055
H	-9.096687	-3.385487	2.755740
H	-9.683938	-2.325460	4.935412
H	-7.999446	-2.838459	4.928667
H	-8.211618	-0.530653	5.849949
H	-6.547237	0.577406	4.358209
H	-6.143714	-1.120856	4.587009
H	-9.875340	-1.539758	1.268770
H	-10.792545	-1.556766	2.771586
H	-7.655883	1.345466	2.191924
H	-8.018957	0.176350	0.926426
H	-10.268318	0.136169	4.605114
H	-8.987673	1.321622	4.370175
H	-10.082267	0.764689	2.198050
C	-10.903199	0.322006	-2.687753
C	-9.532885	0.637759	-3.306723
C	-9.152786	2.096608	-3.009675
C	-10.214407	3.031409	-3.609306
C	-11.586708	2.721944	-2.991304
C	-11.960412	1.261383	-3.288044
C	-9.090886	2.306911	-1.488908
C	-10.836339	0.536554	-1.167738
C	-11.518611	2.930160	-1.470533
C	-10.460298	1.995213	-0.865476
H	-11.172355	-0.718307	-2.900855
H	-8.771541	-0.039824	-2.901791
H	-9.562552	0.470073	-4.390214
H	-8.175263	2.319349	-3.451401
H	-9.943780	4.077451	-3.421119
H	-10.255378	2.905668	-4.698051
H	-12.342049	3.389162	-3.420638
H	-12.947891	1.032934	-2.868922
H	-12.032719	1.104330	-4.371134
H	-8.321800	1.660002	-1.049759
H	-8.801407	3.340529	-1.263812
H	-11.803640	0.294471	-0.710877
H	-10.097946	-0.142083	-0.723908
H	-11.271306	3.974481	-1.244588
H	-12.498428	2.731921	-1.019286
H	-10.412789	2.145447	0.218693
C	-3.967880	0.805300	-1.278773
C	-5.467872	0.684960	-0.968730

C	-6.021367	-0.612690	-1.577581
C	-5.812084	-0.590592	-3.099474
C	-4.312942	-0.473438	-3.415310
C	-3.763714	0.823650	-2.801421
C	-5.274513	-1.814716	-0.979140
C	-3.226525	-0.400316	-0.680584
C	-3.570264	-1.675513	-2.811838
C	-3.773892	-1.700441	-1.289288
H	-3.575362	1.731084	-0.844073
H	-5.629985	0.691260	0.115895
H	-6.005910	1.550186	-1.374750
H	-7.090765	-0.696691	-1.354828
H	-6.221444	-1.503585	-3.548613
H	-6.356620	0.252096	-3.542435
H	-4.166173	-0.457316	-4.500781
H	-2.697679	0.929453	-3.036663
H	-4.271683	1.691482	-3.239386
H	-5.432322	-1.854819	0.105445
H	-5.674025	-2.748977	-1.391910
H	-2.150845	-0.315835	-0.877490
H	-3.348691	-0.414473	0.409202
H	-3.939835	-2.607634	-3.256119
H	-2.500903	-1.614340	-3.047825
H	-3.244155	-2.558396	-0.860871
C	0.808430	3.732284	-3.422458
C	1.361087	5.028538	-2.810185
C	1.197498	4.992176	-1.282811
C	-0.292287	4.854161	-0.933609
C	-0.849524	3.557474	-1.541060
C	-0.680339	3.597316	-3.067734
C	1.965909	3.787868	-0.717147
C	1.578352	2.531423	-2.851407
C	-0.075670	2.356783	-0.975176
C	1.414619	2.488461	-1.324383
H	0.925807	3.760352	-4.511285
H	2.419099	5.148021	-3.072987
H	0.831662	5.895132	-3.224524
H	1.592678	5.917041	-0.848364
H	-0.424205	4.847170	0.155155
H	-0.850187	5.718030	-1.314835
H	-1.911810	3.461451	-1.291308
H	-1.092550	2.685133	-3.516031
H	-1.245450	4.438714	-3.486873
H	3.035187	3.883669	-0.941427
H	1.872699	3.761905	0.375314
H	1.206773	1.600374	-3.296461
H	2.640548	2.605924	-3.113985
H	-0.204012	2.305530	0.112812
H	-0.477596	1.422578	-1.385886
H	1.964798	1.631858	-0.919680
C	-1.764619	3.411963	2.585800
C	-2.204268	1.970522	2.286566
C	-3.585819	1.709856	2.906505
C	-3.508951	1.917784	4.426802

C	-3.072554	3.359106	4.731852
C	-1.692499	3.615696	4.106908
C	-4.605058	2.692704	2.309887
C	-2.788535	4.390206	1.989492
C	-4.092767	4.337858	4.130231
C	-4.171197	4.136053	2.609255
H	-0.779484	3.595621	2.143206
H	-2.240274	1.804970	1.203041
H	-1.470883	1.262492	2.691278
H	-3.897520	0.681810	2.691449
H	-4.485081	1.715023	4.883876
H	-2.798712	1.208413	4.868625
H	-3.017910	3.504889	5.816199
H	-1.360127	4.635874	4.334351
H	-0.949930	2.936658	4.543345
H	-4.685478	2.541256	1.226636
H	-5.600120	2.503904	2.730647
H	-2.475206	5.424149	2.178657
H	-2.836043	4.268016	0.900652
H	-5.079132	4.178021	4.582287
H	-3.803282	5.370809	4.358651
H	-4.899407	4.834303	2.182161
C	-2.142230	-3.141488	2.238365
C	-1.579993	-1.847458	2.846607
C	-1.776914	-1.861751	4.370408
C	-3.276051	-1.969394	4.688917
C	-3.842978	-3.263716	4.085402
C	-3.640417	-3.246032	2.562378
C	-1.042227	-3.072051	4.967234
C	-1.406087	-4.348299	2.840567
C	-3.102791	-4.470492	4.682520
C	-1.603261	-4.369193	4.364097
H	-2.001053	-3.129166	1.152075
H	-0.514640	-1.749736	2.605346
H	-2.085002	-0.977054	2.410356
H	-1.374847	-0.938519	4.801999
H	-3.431390	-1.960392	5.774571
H	-3.810284	-1.100802	4.285185
H	-4.911908	-3.338074	4.313237
H	-4.058987	-4.156464	2.116419
H	-4.181443	-2.400257	2.120863
H	0.033142	-2.997716	4.765016
H	-1.159286	-3.082643	6.057655
H	-1.784445	-5.278259	2.398960
H	-0.337353	-4.295619	2.600169
H	-3.255321	-4.505813	5.768018
H	-3.512277	-5.402646	4.274628
H	-1.077122	-5.230091	4.791042
C	0.657944	-5.015431	-5.831774
C	-0.845405	-5.160761	-5.549988
C	-1.363201	-6.473128	-6.158702
C	-1.124556	-6.459290	-7.676367
C	0.378117	-6.317207	-7.963933
C	0.891557	-5.005441	-7.350261

```
C     -0.606519     -7.656480     -5.535886
C      1.409199     -6.202475     -5.209287
C      1.130526     -7.500645     -7.336168
C      0.897571     -7.517203     -5.817713
H      1.024988     -4.079189     -5.397217
H     -1.028841     -5.148816     -4.468813
H     -1.391003     -4.308789     -5.973568
H     -2.435059     -6.574931     -5.956111
H     -1.508384     -7.383168     -8.125792
H     -1.675514     -5.630264     -8.136818
H      0.545822     -6.307023     -9.046439
H      1.959904     -4.882000     -7.565585
H      0.376598     -4.150566     -7.805215
H     -0.784808     -7.690849     -4.454281
H     -0.980831     -8.601152     -5.948583
H      2.486822     -6.099871     -5.385919
H      1.265967     -6.210209     -4.122004
H      0.786724     -8.442879     -7.779803
H      2.203021     -7.421771     -7.551802
H      1.434299     -8.361880     -5.371963
C      3.406841      2.243271      4.336608
C      1.908443      2.138389      4.659694
C      1.335480      0.848455      4.052456
C      1.531464      0.872352      2.528820
C      3.028928      0.974073      2.199941
C      3.597662      2.263476      2.812254
C      2.074503     -0.363203      4.641117
C      4.140324      1.028084      4.925056
C      3.763819     -0.237602      2.793670
C      3.573463     -0.264391      4.317905
H      3.813251      3.163574      4.770179
H      1.756048      2.143546      5.745733
H      1.376262      3.010486      4.260717
H      0.267253      0.775470      4.284512
H      1.108226     -0.034940      2.080994
H      0.992214      1.722081      2.092903
H      3.166216      0.991504      1.113250
H      4.662654      2.358332      2.567795
H      3.095292      3.137939      2.381072
H      1.925907     -0.404491      5.726958
H      1.661202     -1.291965      4.229516
H      5.215068      1.101411      4.718791
H      4.027696      1.012339      6.015848
H      3.380199     -1.164465      2.350298
H      4.831644     -0.187408      2.548321
H      4.097622     -1.129187      4.739371
```

Adamantane$_{32}$

```
        832
E=-1488.7683
C     -1.644550     -5.913015      9.742479
C     -1.447820     -5.930526      8.218807
C      0.051430     -5.868170      7.888178
```

C	0.647145	-4.575992	8.467701
C	0.455845	-4.554690	9.992122
C	-1.044412	-4.620813	10.317393
C	0.761088	-7.079652	8.512267
C	-0.930769	-7.124761	10.361457
C	1.164025	-5.769722	10.610922
C	0.569726	-7.064892	10.036825
H	-2.713952	-5.957305	9.975992
H	-1.890162	-6.838923	7.792333
H	-1.969076	-5.079943	7.763007
H	0.189357	-5.881341	6.801399
H	1.713733	-4.509968	8.220944
H	0.162666	-3.702007	8.015722
H	0.880777	-3.632389	10.403025
H	-1.196017	-4.586324	11.403115
H	-1.558860	-3.747391	9.898518
H	0.359579	-8.009659	8.091894
H	1.829779	-7.056822	8.266891
H	-1.081026	-7.135161	11.447879
H	-1.362912	-8.054963	9.973211
H	2.239777	-5.724613	10.402257
H	1.052116	-5.755237	11.701899
H	1.076199	-7.930026	10.478728
C	-2.029541	-10.935061	4.009926
C	-0.653931	-10.714652	3.362082
C	-0.172109	-9.282556	3.641191
C	-1.181176	-8.282834	3.055711
C	-2.558371	-8.496843	3.702581
C	-3.033921	-9.931069	3.423667
C	-0.066076	-9.067928	5.158914
C	-1.918294	-10.716530	5.526770
C	-2.446294	-8.284708	5.220202
C	-1.440428	-9.284324	5.811173
H	-2.371267	-11.956430	3.809579
H	0.068746	-11.438924	3.756923
H	-0.716259	-10.886339	2.280606
H	0.809022	-9.127825	3.178886
H	-0.838100	-7.255993	3.231079
H	-1.252106	-8.411950	1.968902
H	-3.276234	-7.783454	3.283301
H	-4.025813	-10.090968	3.863609
H	-3.138074	-10.089312	2.343348
H	0.667041	-9.761908	5.587594
H	0.295719	-8.054519	5.370903
H	-2.890207	-10.891151	6.004147
H	-1.217805	-11.440103	5.960956
H	-2.125845	-7.257814	5.433787
H	-3.427899	-8.414784	5.691897
H	-1.361444	-9.131231	6.893104
C	3.436751	7.574829	2.612743
C	2.987383	6.149037	2.258102
C	1.603687	5.874242	2.867057
C	1.681039	6.022979	4.394258
C	2.127149	7.448451	4.754704

C	3.509325	7.719375	4.140590
C	0.591604	6.886327	2.308342
C	2.419958	8.582331	2.054158
C	1.114067	8.456641	4.190833
C	1.035195	8.314060	2.663189
H	4.423400	7.768583	2.177895
H	2.950913	6.025554	1.168995
H	3.715630	5.420916	2.635527
H	1.285056	4.857407	2.612486
H	0.703254	5.809516	4.842842
H	2.386108	5.292250	4.808707
H	2.182121	7.552019	5.843878
H	3.848564	8.727729	4.407244
H	4.246934	7.018940	4.550837
H	0.510817	6.777233	1.220037
H	-0.404994	6.688342	2.721170
H	2.740273	9.606044	2.283085
H	2.372288	8.502462	0.961406
H	0.126345	8.286334	4.636051
H	1.410507	9.478016	4.458938
H	0.312071	9.033233	2.263024
C	-1.227335	8.140270	8.922616
C	-1.015871	8.160495	7.400953
C	-1.587029	6.876144	6.780328
C	-0.870126	5.657700	7.381901
C	-1.080993	5.630837	8.903650
C	-0.511497	6.918548	9.518811
C	-3.089364	6.785887	7.089573
C	-2.730741	8.047194	9.226021
C	-2.584600	5.544229	9.207621
C	-3.305868	6.762280	8.610652
H	-0.819287	9.056569	9.363073
H	-1.504975	9.039642	6.964366
H	0.053240	8.245802	7.171439
H	-1.435679	6.892970	5.695388
H	-1.255385	4.734710	6.931875
H	0.201334	5.699321	7.151472
H	-0.568605	4.761717	9.330425
H	-0.637929	6.903156	10.608188
H	0.566446	6.982353	9.326594
H	-3.617257	7.639724	6.647841
H	-3.513102	5.882168	6.635070
H	-2.896620	8.052641	10.310204
H	-3.251321	8.923725	8.821734
H	-3.000061	4.619094	8.790087
H	-2.747733	5.503820	10.291466
H	-4.377779	6.698534	8.827893
C	-6.355415	4.055453	8.967820
C	-7.857324	3.970378	9.280252
C	-8.442710	2.687531	8.669856
C	-8.235549	2.707634	7.147645
C	-6.734574	2.789556	6.829416
C	-6.153460	4.071931	7.444856
C	-7.723779	1.466820	9.264660

C	-5.642068	2.831284	9.562374
C	-6.019811	1.568885	7.429258
C	-6.221386	1.545818	8.952111
H	-5.940144	4.970730	9.403626
H	-8.017394	3.978403	10.365169
H	-8.375220	4.849015	8.876851
H	-9.513429	2.628669	8.894315
H	-8.667375	1.805574	6.697454
H	-8.760542	3.563968	6.707271
H	-6.589310	2.804314	5.743723
H	-5.085602	4.152696	7.207955
H	-6.641288	4.952520	7.009462
H	-7.880655	1.428354	10.349440
H	-8.146207	0.543191	8.850780
H	-4.565012	2.890426	9.363752
H	-5.762682	2.817894	10.652343
H	-6.412310	0.646743	6.983819
H	-4.949696	1.604954	7.191520
H	-5.711576	0.674603	9.377938
C	5.581717	4.851744	8.194024
C	5.136120	3.418514	7.865576
C	3.769138	3.139459	8.509348
C	3.880409	3.305891	10.032623
C	4.322887	4.738840	10.366955
C	5.688262	5.013954	9.718102
C	2.736111	4.137077	7.963083
C	4.544024	5.844744	7.648049
C	3.288775	5.732498	9.815601
C	3.175923	5.572205	8.291850
H	6.556452	5.048529	7.734375
H	5.075598	3.282563	6.779028
H	5.878868	2.700527	8.234049
H	3.453156	2.117316	8.273415
H	2.915015	3.089624	10.506017
H	4.600861	2.585525	10.438673
H	4.402070	4.855026	11.453361
H	6.025194	6.027918	9.965732
H	6.440897	4.324093	10.118865
H	2.631186	4.015168	6.878203
H	1.750959	3.935736	8.400997
H	4.861041	6.873491	7.858233
H	4.471861	5.752273	6.557636
H	2.313013	5.559265	10.285385
H	3.582846	6.759155	10.065528
H	2.437846	6.281031	7.900625
C	-3.487326	10.748684	3.381123
C	-4.990396	10.596995	3.661045
C	-5.519449	9.325054	2.980441
C	-5.287160	9.424461	1.464985
C	-3.784814	9.573245	1.179108
C	-3.260099	10.844329	1.864648
C	-4.767982	8.103111	3.530843
C	-2.741312	9.523109	3.931010
C	-3.037600	8.350959	1.734466

C	-3.264212	8.248756	3.250529
H	-3.112250	11.656084	3.866977
H	-5.169158	10.548031	4.741961
H	-5.532132	11.475332	3.289564
H	-6.591088	9.218617	3.181805
H	-5.679001	8.530365	0.965074
H	-5.834566	10.282041	1.055401
H	-3.621641	9.644479	0.098206
H	-2.191882	10.973121	1.651830
H	-3.771313	11.727170	1.461870
H	-4.941903	8.007852	4.609506
H	-5.150289	7.186087	3.066363
H	-1.663793	9.628818	3.755581
H	-2.879977	9.453891	5.016708
H	-3.389513	7.437951	1.239097
H	-1.965531	8.435250	1.518762
H	-2.731202	7.376485	3.644603
C	-8.961901	5.453562	1.226794
C	-8.415480	6.737859	1.869124
C	-8.618971	6.684252	3.391186
C	-10.118333	6.553834	3.699975
C	-10.669461	5.269082	3.062340
C	-10.460368	5.326116	1.541166
C	-7.874713	5.467103	3.961642
C	-8.216246	4.239783	1.802717
C	-9.919741	4.055504	3.633201
C	-8.419922	4.179582	3.324343
H	-8.816062	5.493918	0.141800
H	-7.350117	6.852125	1.635294
H	-8.927343	7.613595	1.451949
H	-8.228177	7.600607	3.847102
H	-10.278546	6.534668	4.784789
H	-10.659543	7.426668	3.315128
H	-11.738579	5.178466	3.283304
H	-10.867730	4.422744	1.071122
H	-11.007973	6.177000	1.117958
H	-6.799270	5.557132	3.766317
H	-7.996471	5.428617	5.050920
H	-8.583219	3.317185	1.336732
H	-7.147050	4.309036	1.568663
H	-10.076695	3.992062	4.716787
H	-10.317967	3.129575	3.200723
H	-7.886987	3.313788	3.732547
C	10.191307	-1.073392	3.536925
C	9.702725	-2.492823	3.209683
C	8.328317	-2.730291	3.854327
C	8.445322	-2.566251	5.377432
C	8.930781	-1.147003	5.710559
C	10.303461	-0.913428	5.060838
C	7.325415	-1.702485	3.307891
C	9.183601	-0.050062	2.990784
C	7.926672	-0.123126	5.159037
C	7.808271	-0.280984	3.635453
H	11.171271	-0.906259	3.076656

H	9.637588	-2.627625	2.123259
H	10.423780	-3.232530	3.578281
H	7.981679	-3.742649	3.619254
H	7.474118	-2.753127	5.851454
H	9.144039	-3.307642	5.783618
H	9.013978	-1.032516	6.796845
H	10.670802	0.090132	5.307603
H	11.035260	-1.625298	5.461701
H	7.216316	-1.821920	2.223147
H	6.334890	-1.873874	3.746433
H	9.531461	0.968848	3.200103
H	9.108131	-0.141058	1.900471
H	6.946394	-0.266679	5.629425
H	8.251553	0.894409	5.408112
H	7.091598	0.449411	3.244107
C	8.635361	6.360818	4.247130
C	7.153529	6.259466	4.640177
C	6.551181	4.967972	4.065569
C	6.675907	4.985487	2.534366
C	8.156530	5.083657	2.135588
C	8.754890	6.374647	2.715464
C	7.315278	3.757575	4.624035
C	9.393909	5.146899	4.805656
C	8.916736	3.873269	2.699309
C	8.797656	3.852856	4.230860
H	9.062755	7.282239	4.657530
H	7.051970	6.269191	5.732113
H	6.604455	7.130755	4.262918
H	5.494847	4.897510	4.347492
H	6.231055	4.077046	2.110457
H	6.118018	5.834272	2.120628
H	8.242998	5.096541	1.043614
H	9.807442	6.466935	2.421214
H	8.234095	7.248129	2.304635
H	7.217443	3.720848	5.715784
H	6.882014	2.827793	4.235936
H	10.457956	5.217797	4.549190
H	9.332268	5.135682	5.900569
H	8.511641	2.945215	2.278082
H	9.972017	3.920888	2.404219
H	9.339777	2.988969	4.630928
C	3.526818	-1.823179	9.666468
C	3.724027	-1.826921	8.142762
C	5.223510	-1.764489	7.813204
C	5.821605	-0.478945	8.404902
C	5.629833	-0.471430	9.929393
C	4.129337	-0.537576	10.253558
C	5.930540	-2.983134	8.426233
C	4.237973	-3.042048	10.274385
C	6.335381	-1.693571	10.537100
C	5.738693	-2.982161	9.950801
H	2.457252	-1.867500	9.899219
H	3.280018	-2.730430	7.707723
H	3.204622	-0.971101	7.694721

H	5.361777	-1.767836	6.726394
H	6.888405	-0.412762	8.159108
H	5.339021	0.400168	7.960910
H	6.056465	0.446163	10.348986
H	3.977436	-0.512876	11.339505
H	3.616774	0.340726	9.842654
H	5.527319	-3.908391	7.997107
H	6.999358	-2.960159	8.181420
H	4.087329	-3.062243	11.360615
H	3.804107	-3.967737	9.877373
H	7.411291	-1.648673	10.329206
H	6.223132	-1.689002	11.628129
H	6.243291	-3.852374	10.384806
C	-4.777079	-3.381879	8.249794
C	-5.774383	-2.384659	7.640269
C	-7.164090	-2.595213	8.261022
C	-7.635591	-4.029939	7.978715
C	-6.643124	-5.031686	8.588975
C	-5.254656	-4.814644	7.967689
C	-7.081600	-2.376770	9.779662
C	-4.700634	-3.160859	9.768429
C	-6.561516	-4.806828	10.106708
C	-6.087388	-3.374118	10.394441
H	-3.787148	-3.230242	7.805776
H	-5.434790	-1.356902	7.816688
H	-5.823807	-2.520006	6.552983
H	-7.873361	-1.882839	7.825425
H	-8.636016	-4.186700	8.399944
H	-7.717899	-4.193537	6.897366
H	-6.980969	-6.053801	8.386023
H	-4.540386	-5.537303	8.380661
H	-5.294654	-4.992530	6.886146
H	-6.765706	-1.349076	9.996206
H	-8.072237	-2.505464	10.232330
H	-3.975934	-3.853628	10.213263
H	-4.342535	-2.146864	9.983694
H	-7.542907	-4.977598	10.565540
H	-5.870906	-5.529775	10.557675
H	-6.030330	-3.215483	11.476952
C	-11.222520	-4.721342	-1.547010
C	-10.683966	-3.415683	-0.942199
C	-9.176556	-3.299544	-1.216389
C	-8.450346	-4.496260	-0.583191
C	-8.983071	-5.804949	-1.186884
C	-10.491051	-5.914499	-0.913128
C	-8.933499	-3.302148	-2.733527
C	-10.974434	-4.719785	-3.063386
C	-8.740993	-5.801089	-2.704157
C	-9.467830	-4.607702	-3.342734
H	-12.297155	-4.802273	-1.350236
H	-11.212263	-2.555762	-1.371351
H	-10.872208	-3.396519	0.138148
H	-8.794842	-2.367706	-0.784813
H	-7.370229	-4.414430	-0.754968

H	-8.599337	-4.495405	0.503389
H	-8.464342	-6.656727	-0.733627
H	-10.880489	-6.854748	-1.322072
H	-10.676219	-5.939174	0.167650
H	-9.429680	-2.440531	-3.196404
H	-7.861729	-3.200259	-2.942720
H	-11.373168	-5.638474	-3.510620
H	-11.507109	-3.882974	-3.531108
H	-7.665992	-5.742599	-2.913372
H	-9.098821	-6.739603	-3.144743
H	-9.293663	-4.606283	-4.424359
C	-4.154060	-8.246612	-1.636401
C	-2.772466	-8.005336	-2.263750
C	-2.313581	-6.568257	-1.971888
C	-3.327386	-5.579349	-2.567462
C	-4.710636	-5.814243	-1.941082
C	-5.163224	-7.253370	-2.232668
C	-2.231840	-6.358844	-0.451936
C	-4.067145	-8.033225	-0.117237
C	-4.622818	-5.607244	-0.421156
C	-3.612292	-6.596112	0.179863
H	-4.479409	-9.271504	-1.845816
H	-2.045940	-8.721756	-1.861713
H	-2.817239	-8.173130	-3.346706
H	-1.328145	-6.398641	-2.419586
H	-3.000407	-4.548859	-2.382981
H	-3.381246	-5.704666	-3.655694
H	-5.431860	-5.108535	-2.367533
H	-6.159035	-7.428197	-1.807506
H	-5.250009	-7.408277	-3.315004
H	-1.495754	-7.044985	-0.015789
H	-1.886485	-5.341699	-0.230628
H	-5.043315	-8.222674	0.345579
H	-3.363368	-8.749408	0.323829
H	-4.319002	-4.577164	-0.198784
H	-5.609186	-5.752254	0.036026
H	-3.550627	-6.446692	1.263431
C	-1.056507	-1.430342	-7.277450
C	-2.108543	-0.493258	-7.890377
C	-1.737321	0.968135	-7.594612
C	-1.683348	1.181871	-6.074134
C	-0.630272	0.249075	-5.456212
C	-1.004094	-1.210465	-5.757784
C	-0.359257	1.275847	-8.200454
C	0.319633	-1.116493	-7.884643
C	0.744931	0.558199	-6.067762
C	0.697139	0.342976	-7.588170
H	-1.323136	-2.471574	-7.489240
H	-2.171057	-0.654268	-8.973382
H	-3.099327	-0.720308	-7.478374
H	-2.488401	1.634459	-8.032993
H	-1.438677	2.227182	-5.849818
H	-2.666895	0.984507	-5.630808
H	-0.593921	0.402780	-4.372191

H	-0.270315	-1.888228	-5.304826
H	-1.975470	-1.450382	-5.308510
H	-0.388743	1.147203	-9.289223
H	-0.091222	2.322591	-8.012525
H	1.076775	-1.793121	-7.470028
H	0.301111	-1.287777	-8.967730
H	1.032602	1.592403	-5.843076
H	1.509812	-0.087768	-5.619823
H	1.678266	0.564352	-8.022488
C	1.031052	-4.102831	-1.681783
C	2.407373	-3.827733	-2.306799
C	2.839618	-2.386227	-1.996669
C	1.807038	-1.408807	-2.578800
C	0.428941	-1.677543	-1.954670
C	0.003031	-3.120982	-2.264554
C	2.918725	-2.194964	-0.474188
C	1.115258	-3.907488	-0.160042
C	0.514169	-1.488578	-0.432250
C	1.543475	-2.466090	0.155328
H	0.724702	-5.130828	-1.904227
H	3.147478	-4.535599	-1.914493
H	2.364821	-3.982340	-3.391805
H	3.821338	-2.192484	-2.442735
H	2.114893	-0.374862	-2.381217
H	1.754603	-1.521050	-3.668528
H	-0.305682	-0.979973	-2.371529
H	-0.988985	-3.319838	-1.841084
H	-0.081765	-3.263497	-3.348749
H	3.667851	-2.872820	-0.047390
H	3.245234	-1.174491	-0.239964
H	0.143184	-4.121081	0.300879
H	1.832645	-4.616045	0.271306
H	0.798906	-0.455964	-0.196769
H	-0.468934	-1.657862	0.023618
H	1.603260	-2.329546	1.240700
C	8.002644	-3.612988	-0.366270
C	6.510539	-3.717922	-0.015280
C	5.924655	-5.004171	-0.617987
C	6.091666	-4.972793	-2.144935
C	7.582723	-4.870989	-2.501716
C	8.164474	-3.585291	-1.893857
C	6.673344	-6.219561	-0.049583
C	8.745786	-4.831900	0.202009
C	8.327376	-6.086417	-1.928166
C	8.166001	-6.120697	-0.400740
H	8.418280	-2.695313	0.064113
H	6.378825	-3.718084	1.073473
H	5.971871	-2.843263	-0.399677
H	4.860949	-5.077202	-0.366029
H	5.658949	-5.877367	-2.589185
H	5.545202	-4.120307	-2.566243
H	7.699346	-4.848218	-3.590719
H	9.224735	-3.490324	-2.158044
H	7.655002	-2.708135	-2.311008

H	6.545370	-6.266172	1.038667
H	6.251226	-7.145799	-0.457912
H	9.816498	-4.758664	-0.024285
H	8.653897	-4.853031	1.294652
H	7.934333	-7.010627	-2.368814
H	9.390401	-6.036109	-2.193521
H	8.697084	-6.988161	0.006331
C	8.015047	1.736379	-2.047570
C	7.589463	0.296543	-2.373659
C	6.214366	0.008152	-1.751651
C	6.297560	0.186049	-0.227880
C	6.719927	1.625613	0.104039
C	8.093544	1.910014	-0.523048
C	5.181108	0.991601	-2.322613
C	6.977164	2.715166	-2.618290
C	5.685711	2.605057	-0.472002
C	5.600884	2.433278	-1.996319
H	8.995600	1.939818	-2.491684
H	7.549136	0.152605	-3.460115
H	8.332805	-0.411421	-1.987419
H	5.912687	-1.018696	-1.985870
H	5.326302	-0.036683	0.230274
H	7.017983	-0.524263	0.195561
H	6.779110	1.749975	1.190814
H	8.416048	2.928973	-0.276716
H	8.845933	1.230493	-0.104550
H	5.096218	0.861267	-3.408266
H	4.190561	0.783353	-1.900378
H	7.280261	3.748452	-2.409845
H	6.924834	2.614560	-3.709115
H	4.703722	2.425227	-0.017937
H	5.965198	3.636292	-0.224201
H	4.862697	3.131974	-2.405162
C	-6.797985	-8.453815	4.423963
C	-8.304429	-8.566154	4.704301
C	-9.040142	-7.373524	4.073971
C	-8.501960	-6.066988	4.677105
C	-6.995895	-5.947997	4.396898
C	-6.265446	-7.144475	5.026049
C	-8.798262	-7.370500	2.556646
C	-6.562175	-8.448476	2.905637
C	-6.759053	-5.949067	2.878795
C	-7.292849	-7.255202	2.271078
H	-6.275391	-9.304800	4.874328
H	-8.694151	-9.507005	4.297303
H	-8.484419	-8.589943	5.785962
H	-10.114032	-7.455972	4.274593
H	-9.033678	-5.208375	4.249417
H	-8.685985	-6.047210	5.758122
H	-6.614288	-5.016131	4.828234
H	-5.186040	-7.061338	4.850161
H	-6.409502	-7.143599	6.113351
H	-9.196515	-8.289338	2.109470
H	-9.334550	-6.534158	2.092146

H	-5.487977	-8.389291	2.692112
H	-6.921152	-9.386719	2.465642
H	-7.259750	-5.088208	2.419403
H	-5.688459	-5.844085	2.664846
H	-7.123613	-7.253934	1.188670
C	-5.750752	4.390727	-2.722618
C	-6.774027	5.344612	-3.357910
C	-6.379138	6.800163	-3.063994
C	-6.345931	7.021547	-1.544016
C	-5.321612	6.071957	-0.903737
C	-5.718970	4.618276	-1.203511
C	-4.985559	7.077448	-3.648665
C	-4.358953	4.674177	-3.308704
C	-3.930771	6.350676	-1.494169
C	-3.957831	6.127685	-3.013968
H	-6.034226	3.353693	-2.933125
H	-6.821915	5.178626	-4.440909
H	-7.775695	5.139304	-2.961094
H	-7.109725	7.478479	-3.518327
H	-6.084533	8.063160	-1.321369
H	-7.340295	6.845958	-1.115923
H	-5.300039	6.231156	0.179886
H	-5.006141	3.928946	-0.734827
H	-6.702099	4.399939	-0.769039
H	-4.999700	6.943144	-4.737057
H	-4.700180	8.119828	-3.462022
H	-3.622117	3.985305	-2.877978
H	-4.363063	4.497061	-4.391004
H	-3.626611	7.380348	-1.270382
H	-3.186146	5.692499	-1.030162
H	-2.965563	6.327381	-3.433224
C	-6.252329	-5.506308	-7.272507
C	-4.870052	-5.211548	-7.875009
C	-4.471231	-3.758704	-7.573114
C	-5.511182	-2.807728	-8.185155
C	-6.895339	-3.096171	-7.583642
C	-7.287795	-4.550823	-7.885154
C	-4.424271	-3.549444	-6.051668
C	-6.200336	-5.292879	-5.751753
C	-6.842215	-2.889092	-6.062119
C	-5.805638	-3.840163	-5.444691
H	-6.534820	-6.542368	-7.489043
H	-4.124059	-5.900729	-7.461177
H	-4.889350	-5.378710	-8.958816
H	-3.485210	-3.550939	-8.003093
H	-5.226835	-1.765962	-7.993512
H	-5.541079	-2.932777	-9.274340
H	-7.635197	-2.417444	-8.021805
H	-8.283587	-4.764143	-7.477897
H	-7.349568	-4.706700	-8.969065
H	-3.670477	-4.208075	-5.603522
H	-4.121706	-2.520391	-5.822898
H	-7.176613	-5.520142	-5.306521
H	-5.477770	-5.982765	-5.299300

H	-6.581662	-1.848691	-5.833000
H	-7.830268	-3.072324	-5.622640
H	-5.768776	-3.690725	-4.359998
C	5.013983	-5.165189	3.598037
C	4.545742	-6.598584	3.303565
C	3.167735	-6.835763	3.940588
C	3.267172	-6.630324	5.459915
C	3.732233	-5.196996	5.760242
C	5.108611	-4.963863	5.118231
C	2.158293	-5.834982	3.357273
C	3.999789	-4.168933	3.015088
C	2.721684	-4.200291	5.171935
C	2.620782	-4.399580	3.651952
H	5.996545	-4.998279	3.143259
H	4.493345	-6.762685	2.220488
H	5.271756	-7.319499	3.698706
H	2.835621	-7.858102	3.728905
H	2.293463	-6.816502	5.929047
H	3.970487	-7.352207	5.892453
H	3.802931	-5.052985	6.843898
H	5.461410	-3.949758	5.342038
H	5.844705	-5.655973	5.544996
H	2.061740	-5.984214	2.275033
H	1.165444	-6.006847	3.790339
H	4.333310	-3.140707	3.200864
H	3.936578	-4.289475	1.926863
H	1.738422	-4.343370	5.636201
H	3.031838	-3.172673	5.397261
H	1.899493	-3.688517	3.234361
C	2.871804	-7.739111	-0.216225
C	1.367157	-7.846306	0.075546
C	0.807429	-9.133285	-0.550029
C	1.034569	-9.101338	-2.069185
C	2.538402	-8.997273	-2.366782
C	3.093798	-7.710853	-1.736224
C	1.534874	-10.347697	0.047314
C	3.593716	-8.957053	0.380774
C	3.261612	-10.211730	-1.764457
C	3.040089	-10.246570	-0.244602
H	3.268765	-6.820919	0.230379
H	1.192546	-7.846893	1.158247
H	0.842803	-6.972357	-0.329692
H	-0.265289	-9.207928	-0.340290
H	0.621069	-10.006452	-2.530318
H	0.503916	-8.249565	-2.511615
H	2.697911	-8.974101	-3.450326
H	4.163524	-7.614275	-1.958315
H	2.599907	-6.834357	-2.173036
H	1.364089	-10.394726	1.129653
H	1.130578	-11.274466	-0.377510
H	4.672421	-8.882199	0.196955
H	3.458777	-8.978549	1.468932
H	2.887641	-11.136422	-2.220427
H	4.334212	-10.159808	-1.987613

H	3.555958	-11.113341	0.182993
C	0.405941	0.737905	8.220440
C	-0.617015	1.731361	7.648436
C	-1.992068	1.487311	8.289234
C	-2.446874	0.051282	7.986874
C	-1.428671	-0.946789	8.559608
C	-0.054965	-0.696261	7.918427
C	-1.885731	1.677261	9.810221
C	0.506196	0.930338	9.741579
C	-1.323329	-0.750421	10.079831
C	-0.865769	0.683527	10.387529
H	1.385376	0.913437	7.762176
H	-0.289844	2.760585	7.839197
H	-0.683832	1.616556	6.559718
H	-2.719668	2.197049	7.880385
H	-3.437129	-0.129089	8.422439
H	-2.546035	-0.092389	6.904108
H	-1.754655	-1.969826	8.342404
H	0.677384	-1.415762	8.304367
H	-0.111615	-0.853560	6.834440
H	-1.581533	2.705272	10.041508
H	-2.866062	1.524484	10.277599
H	1.249091	0.240256	10.159779
H	0.852771	1.945329	9.970574
H	-2.293672	-0.945239	10.552373
H	-0.613951	-1.471317	10.504162
H	-0.791757	0.821823	11.471801
C	-6.955914	-0.666491	2.627840
C	-7.403082	-2.107849	2.339546
C	-8.790010	-2.353749	2.953480
C	-8.720975	-2.131924	4.472189
C	-8.277095	-0.690609	4.766280
C	-6.891658	-0.448823	4.147369
C	-9.799237	-1.370071	2.341411
C	-7.969850	0.312615	2.016096
C	-9.287292	0.288931	4.149242
C	-9.357847	0.073233	2.629787
H	-5.966956	-0.493367	2.189548
H	-7.433618	-2.283425	1.257431
H	-6.676757	-2.816709	2.755385
H	-9.107064	-3.381764	2.746238
H	-9.701139	-2.324071	4.925195
H	-8.018028	-2.841625	4.925008
H	-8.228054	-0.534913	5.849517
H	-6.554060	0.571308	4.367199
H	-6.156154	-1.128451	4.594715
H	-9.874097	-1.531257	1.259168
H	-10.798024	-1.548480	2.757859
H	-7.650974	1.346270	2.197404
H	-8.011575	0.180422	0.928188
H	-10.277383	0.139724	4.596763
H	-8.992504	1.322119	4.369666
H	-10.078924	0.772068	2.191692
C	-10.893880	0.305429	-2.714046

C	-9.513826	0.601187	-3.321141
C	-9.119405	2.057231	-3.029042
C	-10.163837	3.000829	-3.644861
C	-11.545793	2.711376	-3.038791
C	-11.933844	1.253547	-3.330507
C	-9.070136	2.276069	-1.509018
C	-10.839593	0.528450	-1.194749
C	-11.490352	2.928055	-1.518689
C	-10.449283	1.984383	-0.897476
H	-11.173237	-0.732914	-2.923599
H	-8.764646	-0.082857	-2.904555
H	-9.534707	0.427251	-4.403852
H	-8.134962	2.265707	-3.462282
H	-9.882693	4.044730	-3.460240
H	-10.195472	2.868935	-4.733190
H	-12.288852	3.384836	-3.479655
H	-12.928083	1.039342	-2.919936
H	-11.997238	1.090749	-4.413308
H	-8.313190	1.622814	-1.058323
H	-8.770657	3.307551	-1.287228
H	-11.814195	0.300604	-0.746157
H	-10.113759	-0.156140	-0.739492
H	-11.232924	3.970742	-1.296536
H	-12.476893	2.744163	-1.076097
H	-10.410776	2.140663	0.186195
C	-3.960406	0.826059	-1.250468
C	-5.458193	0.711078	-0.927980
C	-6.020697	-0.586050	-1.529644
C	-5.823751	-0.567692	-3.053233
C	-4.326870	-0.455905	-3.381500
C	-3.768595	0.840684	-2.774762
C	-5.272767	-1.789212	-0.934840
C	-3.217988	-0.380675	-0.655864
C	-3.583075	-1.659093	-2.781631
C	-3.774371	-1.680297	-1.257426
H	-3.561458	1.751478	-0.820875
H	-5.611445	0.720084	0.157914
H	-5.996804	1.577173	-1.331379
H	-7.088503	-0.666227	-1.298023
H	-6.239617	-1.480297	-3.497153
H	-6.369230	0.275811	-3.493473
H	-4.188899	-0.442443	-4.468161
H	-2.704184	0.942648	-3.018890
H	-4.277389	1.709226	-3.210355
H	-5.421860	-1.826621	0.151073
H	-5.678557	-2.723042	-1.342429
H	-2.143688	-0.299986	-0.861692
H	-3.331317	-0.392241	0.434907
H	-3.959176	-2.590941	-3.220978
H	-2.515486	-1.601770	-3.026440
H	-3.243856	-2.539049	-0.831578
C	0.811529	3.740164	-3.425488
C	1.365386	5.034437	-2.810117
C	1.201191	4.994843	-1.282888

C	-0.288873	4.857691	-0.934543
C	-0.847313	3.562980	-1.545096
C	-0.677519	3.606046	-3.071615
C	1.968066	3.788428	-0.719634
C	1.579916	2.537182	-2.856838
C	-0.074992	2.360174	-0.981612
C	1.415570	2.490990	-1.329975
H	0.929340	3.770536	-4.514207
H	2.423627	5.153340	-3.072262
H	0.837070	5.902538	-3.222713
H	1.597229	5.918299	-0.846230
H	-0.421203	4.848412	0.154153
H	-0.845680	5.723024	-1.314043
H	-1.909797	3.467569	-1.295950
H	-1.090569	2.695322	-3.522100
H	-1.241548	4.449001	-3.489080
H	3.037532	3.883551	-0.943306
H	1.874423	3.760127	0.372732
H	1.207476	1.607540	-3.304109
H	2.642291	2.611099	-3.118859
H	-0.203794	2.306632	0.106212
H	-0.477796	1.427334	-1.394556
H	1.964655	1.632878	-0.926985
C	-1.761199	3.423212	2.596011
C	-2.203642	1.983489	2.292653
C	-3.586202	1.724089	2.910870
C	-3.510043	1.927851	4.431767
C	-3.070857	3.367447	4.740938
C	-1.689798	3.622787	4.117704
C	-4.602930	2.710639	2.316082
C	-2.782615	4.405166	2.001517
C	-4.088564	4.349916	4.141137
C	-4.166273	4.152282	2.619576
H	-0.775349	3.605968	2.154638
H	-2.239179	1.820866	1.208668
H	-1.472047	1.272859	2.696046
H	-3.899894	0.697268	2.692873
H	-4.486939	1.725934	4.887575
H	-2.801624	1.215831	4.872250
H	-3.016724	3.510259	5.825707
H	-1.355460	4.641663	4.348083
H	-0.948982	2.941046	4.552906
H	-4.682853	2.562212	1.232376
H	-5.598702	2.522819	2.735599
H	-2.467263	5.437948	2.193640
H	-2.829559	4.285944	0.912324
H	-5.075602	4.190958	4.592032
H	-3.797089	5.381653	4.372493
H	-4.892696	4.853179	2.193779
C	-2.140714	-3.133353	2.236748
C	-1.579688	-1.840983	2.849622
C	-1.779265	-1.859602	4.373031
C	-3.278943	-1.968310	4.688618
C	-3.844667	-3.260985	4.080461

C	-3.639452	-3.238977	2.557849
C	-1.045480	-3.071502	4.967717
C	-1.405483	-4.341778	2.836822
C	-3.105384	-4.469361	4.675460
C	-1.605312	-4.366997	4.359940
H	-1.997644	-3.117946	1.150744
H	-0.513928	-1.742462	2.610497
H	-2.084037	-0.969406	2.414951
H	-1.378058	-0.937548	4.807928
H	-3.436176	-1.962392	5.774021
H	-3.812572	-1.098641	4.286410
H	-4.913984	-3.336105	4.306221
H	-4.057138	-4.148191	2.108591
H	-4.179804	-2.392018	2.117782
H	0.030231	-2.996477	4.767586
H	-1.164440	-3.085187	6.057898
H	-1.782963	-5.270529	2.391930
H	-0.336338	-4.288301	2.598438
H	-3.259802	-4.507765	5.760585
H	-3.514049	-5.400404	4.264223
H	-1.079819	-5.229039	4.785369
C	0.703129	-5.037165	-5.841532
C	-0.796652	-5.153588	-5.528981
C	-1.353762	-6.451584	-6.133785
C	-1.146683	-6.433707	-7.656036
C	0.352233	-6.320482	-7.974370
C	0.905081	-5.023028	-7.364520
C	-0.608541	-7.653682	-5.533455
C	1.442842	-6.242842	-5.241438
C	1.093285	-7.522609	-7.369003
C	0.891855	-7.543325	-5.846097
H	1.098226	-4.111129	-5.409716
H	-0.957148	-5.144252	-4.444138
H	-1.333477	-4.288248	-5.936360
H	-2.423002	-6.532788	-5.909249
H	-1.558616	-7.346953	-8.102298
H	-1.690107	-5.590983	-8.100289
H	0.497430	-6.307366	-9.060093
H	1.970987	-4.920045	-7.601595
H	0.398283	-4.155223	-7.803895
H	-0.764830	-7.690758	-4.448543
H	-1.010618	-8.588127	-5.943310
H	2.518405	-6.161107	-5.440138
H	1.322258	-6.254034	-4.151441
H	0.721106	-8.455054	-7.810418
H	2.162421	-7.464265	-7.606717
H	1.420428	-8.401322	-5.416327
C	3.405198	2.242961	4.336406
C	1.906755	2.138783	4.659517
C	1.332986	0.849552	4.051548
C	1.528896	0.874235	2.527914
C	3.026401	0.975256	2.199008
C	3.595941	2.263956	2.812052
C	2.071319	-0.362896	4.639447

```
C      4.137988      1.026987      4.924090
C      3.760602     -0.237209      2.791975
C      3.570319     -0.264788      4.316206
H      3.812183      3.162763      4.770498
H      1.754427      2.143388      5.745568
H      1.375072      3.011434      4.261088
H      0.264729      0.777068      4.283623
H      1.105089     -0.032538      2.079576
H      0.990128      1.724546      2.092533
H      3.163636      0.993250      1.112320
H      4.660976      2.358321      2.567588
H      3.094069      3.138975      2.381418
H      1.922762     -0.404739      5.725272
H      1.657439     -1.291167      4.227319
H      5.212764      1.099795      4.717806
H      4.025414      1.010663      6.014879
H      3.376403     -1.163580      2.348077
H      4.828443     -0.187508      2.546595
H      4.093986     -1.130148      4.737128
```

Adamantane$_{33}$

```
         858
E=-1554.4791
C     -1.647566     -5.910069      9.745679
C     -1.450478     -5.928668      8.222067
C      0.048820     -5.865926      7.891733
C      0.643851     -4.573048      8.470398
C      0.452192     -4.550656      9.994757
C     -1.048112     -4.617169     10.319736
C      0.758852     -7.076625      8.516916
C     -0.933409     -7.121034     10.365753
C      1.160749     -5.764908     10.614654
C      0.567134     -7.060774     10.041418
H     -2.717002     -5.954636      9.978982
H     -1.892335     -6.837582      7.796190
H     -1.971993     -5.078660      7.765493
H      0.187002     -5.879875      6.804995
H      1.710466     -4.506759      8.223833
H      0.159102     -3.699619      8.017635
H      0.876636     -3.627857     10.405048
H     -1.199980     -4.581909     11.405396
H     -1.562836     -3.744289      9.900071
H      0.357837     -8.007126      8.097168
H      1.827590     -7.053527      8.271767
H     -1.083910     -7.130662     11.452148
H     -1.365066     -8.051719      9.978124
H      2.236529     -5.719500     10.406201
H      1.048583     -5.749631     11.705595
H      1.073875     -7.925350     10.484103
C     -2.028625    -10.934288      4.019583
C     -0.652185    -10.713903      3.373497
C     -0.171837     -9.281012      3.651060
C     -1.180735     -8.282964      3.062441
```

C	-2.558763	-8.496951	3.707542
C	-3.032838	-9.931968	3.430187
C	-0.068288	-9.063888	5.168599
C	-1.919863	-10.713259	5.536245
C	-2.449169	-8.282316	5.224992
C	-1.443480	-9.280252	5.819097
H	-2.369297	-11.956225	3.820342
H	0.070415	-11.437015	3.770598
H	-0.712729	-10.887359	2.292201
H	0.809888	-9.126298	3.190012
H	-0.838679	-7.255593	3.236697
H	-1.249905	-8.413862	1.975732
H	-3.276504	-7.784757	3.286025
H	-4.025286	-10.091892	3.868862
H	-3.135219	-10.092008	2.349963
H	0.664678	-9.756646	5.599508
H	0.292441	-8.049877	5.379527
H	-2.892379	-10.887832	6.012411
H	-1.219512	-11.435625	5.972657
H	-2.129798	-7.254848	5.437430
H	-3.431401	-8.412360	5.695389
H	-1.366266	-9.125377	6.900902
C	3.433675	7.575107	2.609660
C	2.984463	6.149218	2.255211
C	1.601630	5.873696	2.865796
C	1.680796	6.022080	4.392938
C	2.126758	7.447646	4.753196
C	3.508065	7.719298	4.137453
C	0.588442	6.885502	2.308579
C	2.415779	8.582328	2.052579
C	1.112566	8.455558	4.190824
C	1.031877	8.313329	2.663242
H	4.419709	7.769379	2.173650
H	2.946706	6.025994	1.166119
H	3.713474	5.421304	2.631558
H	1.283108	4.856793	2.611360
H	0.703651	5.808099	4.842668
H	2.386676	5.291539	4.806336
H	2.183024	7.550962	5.842328
H	3.847214	8.727725	4.403945
H	4.246468	7.019066	4.546617
H	0.506363	6.776648	1.220347
H	-0.407566	6.687000	2.722581
H	2.735951	9.606116	2.281371
H	2.366800	8.502715	0.959866
H	0.125462	8.284729	4.637212
H	1.408911	9.476988	4.458823
H	0.307964	9.032303	2.264147
C	-1.221525	8.134819	8.931559
C	-1.011085	8.158436	7.409803
C	-1.583438	6.875867	6.786600
C	-0.866859	5.655605	7.384864
C	-1.076705	5.625349	8.906690
C	-0.506016	6.911291	9.524438

C	-3.085617	6.785797	7.096660
C	-2.724779	8.041943	9.235774
C	-2.580157	5.538941	9.211486
C	-3.301099	6.758801	8.617828
H	-0.812625	9.049852	9.373855
H	-1.499957	9.038885	6.975584
H	0.057920	8.243632	7.179757
H	-1.432817	6.895112	5.701598
H	-1.252979	4.733890	6.932968
H	0.204468	5.697116	7.153800
H	-0.564551	4.754936	9.331105
H	-0.631716	6.893456	10.613863
H	0.571833	6.974895	9.331634
H	-3.613297	7.640970	6.657264
H	-3.510207	5.883387	6.640357
H	-2.889917	8.044983	10.320079
H	-3.245107	8.919720	8.833870
H	-2.996459	4.615023	8.792097
H	-2.742577	5.496122	10.295345
H	-4.372899	6.695197	8.835652
C	-6.357607	4.054052	8.963954
C	-7.859766	3.968453	9.275041
C	-8.444367	2.685916	8.663246
C	-8.235908	2.707127	7.141227
C	-6.734678	2.789577	6.824340
C	-6.154353	4.071639	7.441175
C	-7.725696	1.464934	9.257810
C	-5.644519	2.829612	9.558261
C	-6.020180	1.568631	7.423938
C	-6.223052	1.544456	8.946602
H	-5.942896	4.969108	9.400757
H	-8.020766	3.975686	10.359826
H	-8.377496	4.847267	8.871813
H	-9.515266	2.626678	8.886748
H	-8.667165	1.805295	6.690035
H	-8.760699	3.563663	6.701004
H	-6.588488	2.805125	5.738783
H	-5.086309	4.152786	7.205245
H	-6.641987	4.952433	7.005981
H	-7.883493	1.425676	10.342428
H	-8.147582	0.541509	8.842922
H	-4.567306	2.889112	9.360601
H	-5.766063	2.815434	10.648116
H	-6.412109	0.646722	6.977518
H	-4.949870	1.605084	7.187142
H	-5.713430	0.673047	9.372254
C	5.584474	4.852387	8.191003
C	5.138653	3.419344	7.862044
C	3.772273	3.139805	8.506883
C	3.884924	3.305267	10.030163
C	4.327631	4.738025	10.365008
C	5.692399	5.013628	9.715087
C	2.738697	4.137717	7.962199
C	4.546231	5.845681	7.646610

C	3.292965	5.731981	9.815233
C	3.178732	5.572657	8.291483
H	6.558779	5.049518	7.730592
H	5.077148	3.284086	6.775465
H	5.881774	2.701161	8.229380
H	3.456131	2.117795	8.270584
H	2.919974	3.088646	10.504297
H	4.605784	2.584680	10.435094
H	4.407798	4.853520	11.451416
H	6.029503	6.027450	9.963059
H	6.445436	4.323551	10.114722
H	2.632789	4.016496	6.877337
H	1.753954	3.936043	8.400882
H	4.863386	6.874310	7.857163
H	4.473079	5.753904	6.556205
H	2.317640	5.558396	10.285795
H	3.587209	6.758493	10.065549
H	2.440261	6.281694	7.901384
C	-3.484007	10.753128	3.398111
C	-4.985210	10.602963	3.688669
C	-5.519840	9.330274	3.013846
C	-5.297968	9.427014	1.496657
C	-3.797531	9.574266	1.200154
C	-3.267205	10.846109	1.879944
C	-4.765434	8.108710	3.561062
C	-2.735060	9.527936	3.944854
C	-3.047343	8.352371	1.752355
C	-3.263538	8.252831	3.270116
H	-3.104951	11.661064	3.879856
H	-5.156527	10.555909	4.770874
H	-5.528892	11.481067	3.319488
H	-6.590135	9.224926	3.222790
H	-5.693877	8.532372	1.000948
H	-5.847597	10.284303	1.089449
H	-3.641787	9.643600	0.118034
H	-2.200397	10.973795	1.659533
H	-3.780579	11.728645	1.479249
H	-4.931958	8.015355	4.641057
H	-5.151580	7.191191	3.100755
H	-1.658706	9.632595	3.761805
H	-2.866261	9.460609	5.031598
H	-3.403308	7.438795	1.260944
H	-1.976733	8.435549	1.529105
H	-2.728421	7.380836	3.661939
C	-8.958469	5.456738	1.223968
C	-8.410947	6.738211	1.870985
C	-8.615388	6.679658	3.392736
C	-10.115080	6.549916	3.700210
C	-10.667312	5.267978	3.057885
C	-10.457265	5.329949	1.537035
C	-7.872864	5.459718	3.959477
C	-8.214547	4.240148	1.796188
C	-9.919322	4.051601	3.625046
C	-8.419181	4.175003	3.317482

H	-8.811951	5.500619	0.139203
H	-7.345317	6.852045	1.638163
H	-8.921559	7.615951	1.456496
H	-8.223808	7.594005	3.851997
H	-10.275948	6.527243	4.784859
H	-10.655061	7.424678	3.318023
H	-11.736662	5.177841	3.277919
H	-10.865391	4.428652	1.063682
H	-11.003644	6.182898	1.116409
H	-6.797204	5.549173	3.765084
H	-7.995302	5.417664	5.048547
H	-8.582308	3.319565	1.326852
H	-7.145135	4.308965	1.562992
H	-10.076982	3.984652	4.708318
H	-10.318359	3.127609	3.189187
H	-7.887479	3.307211	3.723045
C	10.191481	-1.068903	3.538655
C	9.703715	-2.488766	3.212069
C	8.329317	-2.726629	3.856588
C	8.445957	-2.561728	5.379628
C	8.930598	-1.142047	5.712098
C	10.303272	-0.908081	5.062504
C	7.325966	-1.699645	3.309431
C	9.183328	-0.046397	2.991795
C	7.926044	-0.118995	5.159857
C	7.808004	-0.277715	3.636334
H	11.171438	-0.901489	3.078476
H	9.638847	-2.624173	2.125704
H	10.425098	-3.227895	3.581185
H	7.983261	-3.739295	3.621982
H	7.474767	-2.748874	5.853572
H	9.144996	-3.302533	5.786330
H	9.013537	-1.026945	6.798340
H	10.670033	0.095805	5.308809
H	11.035378	-1.619349	5.463873
H	7.217128	-1.819707	2.224730
H	6.335453	-1.871332	3.747883
H	9.530606	0.972808	3.200642
H	9.108105	-0.138005	1.901516
H	6.945757	-0.262825	5.630142
H	8.250337	0.898844	5.408457
H	7.091013	0.452093	3.244476
C	8.634257	6.364050	4.242547
C	7.152641	6.262114	4.636256
C	6.550640	4.970201	4.062228
C	6.674702	4.987390	2.530967
C	8.155109	5.086141	2.131530
C	8.753124	6.377550	2.710828
C	7.315532	3.760294	4.620669
C	9.393602	5.150619	4.801052
C	8.916112	3.876243	2.695228
C	8.797698	3.856158	4.226835
H	9.061403	7.285769	4.652535
H	7.051545	6.272066	5.728232

H	6.603004	7.133057	4.259014
H	5.494459	4.899324	4.344621
H	6.230086	4.078638	2.107476
H	6.116246	5.835815	2.117256
H	8.241103	5.098791	1.039517
H	9.805507	6.470248	2.416103
H	8.231751	7.250690	2.300004
H	7.218182	3.723796	5.712469
H	6.882529	2.830216	4.232988
H	10.457507	5.221941	4.544113
H	9.332435	5.139647	5.895994
H	8.511264	2.947898	2.274406
H	9.971245	3.924273	2.399674
H	9.340387	2.992620	4.626887
C	3.526860	-1.821237	9.668898
C	3.723932	-1.825493	8.145176
C	5.223368	-1.762794	7.815459
C	5.821184	-0.476876	8.406626
C	5.629549	-0.468846	9.931132
C	4.129099	-0.535262	10.255457
C	5.930770	-2.981029	8.428874
C	4.238386	-3.039697	10.277202
C	6.335468	-1.690580	10.539227
C	5.739062	-2.979541	9.953459
H	2.457326	-1.865750	9.901762
H	3.280118	-2.729278	7.710511
H	3.204264	-0.969973	7.696865
H	5.361537	-1.766507	6.728637
H	6.887944	-0.410507	8.160711
H	5.338332	0.401947	7.962353
H	6.055981	0.449012	10.350346
H	3.977290	-0.510199	11.341409
H	3.616271	0.342756	9.844275
H	5.527750	-3.906549	8.000127
H	6.999559	-2.957867	8.183956
H	4.087847	-3.059530	11.363453
H	3.804724	-3.965645	9.880571
H	7.411347	-1.645480	10.331219
H	6.223317	-1.685636	11.630264
H	6.243925	-3.849462	10.387740
C	-4.779342	-3.379812	8.247224
C	-5.776809	-2.383118	7.637106
C	-7.166708	-2.594206	8.257250
C	-7.637441	-4.029180	7.974923
C	-6.644808	-5.030402	8.585775
C	-5.256152	-4.812827	7.965095
C	-7.085011	-2.375520	9.775898
C	-4.703692	-3.158550	9.765864
C	-6.563996	-4.805301	10.103514
C	-6.090637	-3.372341	10.391269
H	-3.789275	-3.227795	7.803640
H	-5.437755	-1.355186	7.813541
H	-5.825675	-2.518635	6.549816
H	-7.876095	-1.882207	7.821231

H	-8.637989	-4.186329	8.395715
H	-7.719181	-4.192962	6.893559
H	-6.982106	-6.052695	8.382807
H	-4.541750	-5.535113	8.378494
H	-5.295576	-4.990879	6.883559
H	-6.769673	-1.347656	9.992447
H	-8.075798	-2.504592	10.228130
H	-3.978888	-3.850937	10.211125
H	-4.346142	-2.144368	9.981155
H	-7.545521	-4.976446	10.561920
H	-5.873272	-5.527880	10.554896
H	-6.034146	-3.213534	11.473784
C	-11.210030	-4.731192	-1.559739
C	-10.680215	-3.420843	-0.957354
C	-9.174518	-3.292971	-1.235701
C	-8.437140	-4.483409	-0.603578
C	-8.961098	-5.796740	-1.204858
C	-10.467419	-5.918029	-0.926950
C	-8.935537	-3.294807	-2.753487
C	-10.966052	-4.728826	-3.076781
C	-8.723148	-5.792121	-2.722782
C	-9.461141	-4.605019	-3.360279
H	-12.283456	-4.820486	-1.360003
H	-11.216467	-2.565465	-1.385716
H	-10.865691	-3.402347	0.123483
H	-8.799042	-2.357807	-0.805849
H	-7.358173	-4.393152	-0.778333
H	-8.583204	-4.482905	0.483399
H	-8.434414	-6.644034	-0.752371
H	-10.850492	-6.861647	-1.334142
H	-10.649473	-5.943345	0.154343
H	-9.439779	-2.437503	-3.215662
H	-7.865177	-3.184586	-2.965650
H	-11.358694	-5.650988	-3.522254
H	-11.506602	-3.896621	-3.543683
H	-7.649213	-5.725272	-2.934943
H	-9.074710	-6.733777	-3.161703
H	-9.289906	-4.603046	-4.442372
C	-4.143963	-8.251497	-1.625375
C	-2.761206	-8.009383	-2.249832
C	-2.306288	-6.570266	-1.961831
C	-3.320652	-5.585724	-2.563656
C	-4.705076	-5.821463	-1.940195
C	-5.153694	-7.262614	-2.227896
C	-2.229140	-6.355287	-0.442416
C	-4.061647	-8.032536	-0.106749
C	-4.621846	-5.608884	-0.420782
C	-3.610776	-6.593373	0.186479
H	-4.466479	-9.277844	-1.832031
H	-2.034195	-8.722762	-1.843287
H	-2.802666	-8.181114	-3.332302
H	-1.320017	-6.400053	-2.407457
H	-2.996451	-4.553865	-2.381949
H	-3.371279	-5.715016	-3.651578

H	-5.426695	-5.118871	-2.371101
H	-6.150267	-7.438140	-1.804815
H	-5.237195	-7.421547	-3.309906
H	-1.492729	-7.038245	-0.001844
H	-1.886633	-5.336600	-0.223786
H	-5.038650	-8.222507	0.354091
H	-3.357492	-8.745589	0.338762
H	-4.320909	-4.577352	-0.201245
H	-5.609129	-5.754459	0.034238
H	-3.552385	-6.439979	1.269673
C	-1.048678	-1.428890	-7.289964
C	-2.085318	-0.468563	-7.893067
C	-1.687475	0.983679	-7.587017
C	-1.626142	1.184682	-6.065083
C	-0.588372	0.228585	-5.456955
C	-0.988796	-1.221713	-5.768791
C	-0.305583	1.271529	-8.193901
C	0.331397	-1.134859	-7.898147
C	0.790671	0.517941	-6.069528
C	0.735491	0.315342	-7.591420
H	-1.334276	-2.463556	-7.509077
H	-2.153227	-0.620074	-8.977117
H	-3.079014	-0.681150	-7.480360
H	-2.427614	1.666604	-8.018404
H	-1.362396	2.223719	-5.833359
H	-2.612000	1.001405	-5.620843
H	-0.546750	0.373249	-4.371880
H	-0.266125	-1.915880	-5.322871
H	-1.963228	-1.447798	-5.318960
H	-0.339907	1.151836	-9.283548
H	-0.018536	2.311865	-7.998610
H	1.077359	-1.828010	-7.490619
H	0.307290	-1.297422	-8.982467
H	1.097214	1.545108	-5.837626
H	1.544993	-0.144949	-5.628461
H	1.719378	0.522600	-8.026461
C	1.032040	-4.105871	-1.678646
C	2.408866	-3.831027	-2.302661
C	2.840898	-2.389422	-1.992696
C	1.808807	-1.412183	-2.575995
C	0.430207	-1.680665	-1.952869
C	0.004508	-3.124204	-2.262583
C	2.918796	-2.197623	-0.470219
C	1.115037	-3.909992	-0.156907
C	0.514225	-1.491164	-0.430449
C	1.543038	-2.468493	0.158295
H	0.725842	-5.133939	-1.900971
H	3.148640	-4.538772	-1.909515
H	2.367176	-3.986017	-3.387646
H	3.822979	-2.195861	-2.438047
H	2.116530	-0.378175	-2.378532
H	1.757238	-1.524809	-3.665725
H	-0.304067	-0.983224	-2.370561
H	-0.987850	-3.322886	-1.839834

H	-0.079427	-3.267100	-3.346795
H	3.667565	-2.875346	-0.042585
H	3.245143	-1.177075	-0.236096
H	0.142591	-4.123398	0.303314
H	1.832063	-4.618414	0.275264
H	0.798799	-0.458474	-0.195106
H	-0.469246	-1.660263	0.024694
H	1.601960	-2.331567	1.243666
C	8.002845	-3.611675	-0.362183
C	6.510819	-3.717385	-0.011092
C	5.925944	-5.004755	-0.612386
C	6.092946	-4.974930	-2.139366
C	7.583927	-4.872357	-2.496244
C	8.164670	-3.585537	-1.889798
C	6.675573	-6.218933	-0.042634
C	8.746930	-4.829380	0.207447
C	8.329522	-6.086572	-1.921347
C	8.168157	-6.119293	-0.393886
H	8.417761	-2.693202	0.067191
H	6.379093	-3.716450	1.077659
H	5.971473	-2.843571	-0.396459
H	4.862293	-5.078336	-0.360358
H	5.660939	-5.880331	-2.582623
H	5.545822	-4.123335	-2.561619
H	7.700544	-4.850697	-3.585271
H	9.224858	-3.490034	-2.154079
H	7.654519	-2.709239	-2.307921
H	6.547625	-6.264443	1.045664
H	6.254183	-7.145950	-0.449946
H	9.817588	-4.755559	-0.018917
H	8.655047	-4.849377	1.300112
H	7.937203	-7.011573	-2.360980
H	9.392509	-6.035728	-2.186747
H	8.699912	-6.985893	0.014146
C	-6.162703	3.525416	-6.931940
C	-5.370713	2.340338	-6.358420
C	-5.891338	1.026974	-6.962553
C	-7.379917	0.863624	-6.619193
C	-8.177368	2.044917	-7.193236
C	-7.650639	3.355716	-6.588829
C	-5.723231	1.067147	-8.489287
C	-5.993336	3.559139	-8.458742
C	-8.003139	2.082888	-8.719322
C	-6.516205	2.249244	-9.068035
H	-5.790155	4.460616	-6.499830
H	-4.302933	2.459064	-6.578606
H	-5.468495	2.316618	-5.266253
H	-5.324906	0.183495	-6.552694
H	-7.758719	-0.080846	-7.028267
H	-7.512397	0.813643	-5.531624
H	-9.238178	1.926639	-6.946780
H	-8.225150	4.206153	-6.975689
H	-7.788266	3.350091	-5.500709
H	-4.662098	1.162837	-8.749434

H	-6.073861	0.126507	-8.930976
H	-6.537778	4.413658	-8.878716
H	-4.936752	3.698959	-8.717276
H	-8.393216	1.160074	-9.165495
H	-8.584421	2.910367	-9.144004
H	-6.393880	2.275566	-10.156425
C	8.013011	1.736093	-2.049127
C	7.587938	0.295819	-2.373949
C	6.212969	0.007469	-1.751640
C	6.296154	0.186721	-0.228027
C	6.718009	1.626726	0.102625
C	8.091500	1.911081	-0.524760
C	5.179333	0.990045	-2.323418
C	6.974751	2.714005	-2.620660
C	5.683417	2.605293	-0.474229
C	5.598597	2.432158	-1.998393
H	8.993474	1.939502	-2.493455
H	7.547624	0.150922	-3.460278
H	8.331552	-0.411539	-1.987121
H	5.911654	-1.019692	-1.984955
H	5.324993	-0.035965	0.230356
H	7.016851	-0.522960	0.196005
H	6.777188	1.752055	1.189289
H	8.413644	2.930371	-0.279326
H	8.844151	1.232197	-0.105699
H	5.094450	0.858737	-3.408955
H	4.188877	0.781805	-1.900966
H	7.277480	3.747582	-2.413124
H	6.922418	2.612432	-3.711395
H	4.701509	2.425501	-0.019972
H	5.962538	3.636845	-0.227335
H	4.860141	3.130230	-2.407816
C	-6.798279	-8.454044	4.425783
C	-8.304677	-8.567015	4.706113
C	-9.041154	-7.375868	4.073877
C	-8.503964	-6.068034	4.675080
C	-6.997949	-5.948410	4.394871
C	-6.266734	-7.143412	5.025933
C	-8.799095	-7.374989	2.556578
C	-6.562290	-8.450857	2.907480
C	-6.760925	-5.951631	2.876799
C	-7.293728	-7.259067	2.271013
H	-6.275139	-9.303973	4.877509
H	-8.693688	-9.508761	4.300506
H	-8.484780	-8.589280	5.787788
H	-10.115010	-7.458766	4.274497
H	-9.036236	-5.210449	4.246018
H	-8.688133	-6.046735	5.756044
H	-6.617050	-5.015618	4.824829
H	-5.187366	-7.059783	4.850047
H	-6.410921	-7.140978	6.113216
H	-9.196647	-8.294789	2.110757
H	-9.335916	-6.539734	2.090738
H	-5.488109	-8.391241	2.693992

H	-6.920554	-9.390022	2.468874
H	-7.262173	-5.091826	2.416034
H	-5.690380	-5.846221	2.662817
H	-7.124363	-7.259331	1.188624
C	-5.765057	4.430691	-2.738532
C	-6.776567	5.410014	-3.353641
C	-6.359497	6.854459	-3.036491
C	-6.317719	7.049363	-1.513102
C	-5.305086	6.074234	-0.892937
C	-5.724618	4.631794	-1.215903
C	-4.964095	7.121452	-3.621605
C	-4.371351	4.703898	-3.324951
C	-3.912422	6.342798	-1.483778
C	-3.948075	6.146196	-3.007038
H	-6.064338	3.401620	-2.965585
H	-6.830679	5.263249	-4.439119
H	-7.779714	5.212506	-2.956604
H	-7.081726	7.550950	-3.476460
H	-6.040403	8.083125	-1.273750
H	-7.313021	6.880925	-1.084315
H	-5.277383	6.214584	0.193149
H	-5.020255	3.924303	-0.761706
H	-6.709285	4.420348	-0.781508
H	-4.984021	7.005964	-4.712065
H	-4.662916	8.156248	-3.418330
H	-3.643110	3.997165	-2.908788
H	-4.381849	4.545359	-4.410084
H	-3.592518	7.363983	-1.243651
H	-3.175828	5.666073	-1.033847
H	-2.954487	6.338613	-3.426571
C	-6.247918	-5.547503	-7.279009
C	-4.849992	-5.290273	-7.862291
C	-4.417254	-3.847971	-7.556587
C	-5.423015	-2.870881	-8.184430
C	-6.822639	-3.121748	-7.602171
C	-7.249070	-4.566025	-7.907400
C	-4.386224	-3.637578	-6.034890
C	-6.211738	-5.333076	-5.757938
C	-6.785507	-2.913697	-6.080307
C	-5.783196	-3.890785	-5.447126
H	-6.554581	-6.576098	-7.498235
H	-4.128354	-5.998224	-7.437113
H	-4.858426	-5.458610	-8.946055
H	-3.420133	-3.666976	-7.972853
H	-5.113997	-1.836670	-7.990077
H	-5.440856	-2.996848	-9.273773
H	-7.538079	-2.424447	-8.051579
H	-8.255784	-4.752352	-7.513986
H	-7.299661	-4.721961	-8.991883
H	-3.656465	-4.315140	-5.575328
H	-4.059854	-2.616496	-5.803139
H	-7.199849	-5.533794	-5.326260
H	-5.514079	-6.041052	-5.294472
H	-6.500836	-1.880163	-5.848816

H	-7.784145	-3.070098	-5.654602
H	-5.757686	-3.740627	-4.362206
C	5.014325	-5.161688	3.605493
C	4.547120	-6.595544	3.311619
C	3.168570	-6.832943	3.947382
C	3.266318	-6.626304	5.466656
C	3.730336	-5.192512	5.766384
C	5.107264	-4.959164	5.125632
C	2.159229	-5.833118	3.362257
C	4.000233	-4.166390	3.020732
C	2.719894	-4.196767	5.176269
C	2.620680	-4.397257	3.656333
H	5.997276	-4.994621	3.151615
H	4.495938	-6.760491	2.228611
H	5.273088	-7.315790	3.708062
H	2.837197	-7.855611	3.736128
H	2.292215	-6.812623	5.934914
H	3.969547	-7.347501	5.900474
H	3.799830	-5.047645	6.850004
H	5.459314	-3.944710	5.349039
H	5.843264	-5.650576	5.553689
H	2.063881	-5.983217	2.280031
H	1.166016	-6.005161	3.794418
H	4.333037	-3.137854	3.206078
H	3.938220	-4.287787	1.932534
H	1.736222	-4.339996	5.639617
H	3.029291	-3.168822	5.401140
H	1.899466	-3.686878	3.237452
C	2.876546	-7.740955	-0.203830
C	1.371833	-7.849601	0.087063
C	0.814496	-9.139073	-0.535504
C	1.042768	-9.110845	-2.054564
C	2.546676	-9.005347	-2.351276
C	3.099678	-7.716426	-1.723727
C	1.543274	-10.350806	0.065643
C	3.599796	-8.956228	0.396977
C	3.271214	-10.217119	-1.745151
C	3.048563	-10.248229	-0.225380
H	3.271801	-6.820987	0.240627
H	1.196383	-7.847555	1.169627
H	0.846502	-6.977514	-0.320912
H	-0.258273	-9.214745	-0.326395
H	0.630965	-10.017798	-2.513597
H	0.511198	-8.261044	-2.499677
H	2.706991	-8.984831	-3.434755
H	4.169432	-7.618857	-1.945249
H	2.604830	-6.841832	-2.163260
H	1.371718	-10.395198	1.147971
H	1.140679	-11.279305	-0.357017
H	4.678532	-8.880267	0.213792
H	3.464044	-8.975019	1.485084
H	2.898965	-11.143578	-2.198939
H	4.343909	-10.164203	-1.967617
H	3.565382	-11.113090	0.204927

C	0.405725	0.738476	8.219965
C	-0.617255	1.731947	7.648029
C	-1.992473	1.487324	8.288252
C	-2.446858	0.051322	7.985132
C	-1.428631	-0.946765	8.557795
C	-0.054760	-0.695664	7.917191
C	-1.886699	1.676657	9.809355
C	0.505416	0.930292	9.741220
C	-1.323853	-0.751014	10.078136
C	-0.866716	0.682906	10.386595
H	1.385278	0.914418	7.762111
H	-0.290376	2.761163	7.839335
H	-0.683673	1.617586	6.559239
H	-2.720089	2.197073	7.879453
H	-3.437223	-0.129451	8.420282
H	-2.545616	-0.091915	6.902272
H	-1.754314	-1.969782	8.340049
H	0.677615	-1.415165	8.303079
H	-0.111005	-0.852519	6.833119
H	-1.582808	2.704638	10.041180
H	-2.867157	1.523468	10.276333
H	1.248320	0.240198	10.159384
H	0.851688	1.945263	9.970760
H	-2.294316	-0.946245	10.550264
H	-0.614462	-1.471932	10.502408
H	-0.793106	0.820762	11.470950
C	-6.954519	-0.669255	2.618806
C	-7.399164	-2.111810	2.332609
C	-8.785338	-2.359470	2.947539
C	-8.715977	-2.135536	4.465924
C	-8.274618	-0.693022	4.757919
C	-6.889930	-0.449485	4.138018
C	-9.796677	-1.378456	2.334680
C	-7.970559	0.307178	2.006279
C	-9.286921	0.283846	4.140095
C	-9.357814	0.066035	2.620958
H	-5.966096	-0.494877	2.179806
H	-7.429901	-2.288855	1.250739
H	-6.671330	-2.818785	2.749018
H	-9.100593	-3.388338	2.741792
H	-9.695565	-2.328901	4.919657
H	-8.011501	-2.843346	4.919325
H	-8.225339	-0.535822	5.840927
H	-6.554110	0.571554	4.356353
H	-6.152955	-1.127170	4.585891
H	-9.871764	-1.541192	1.252684
H	-10.794930	-1.558166	2.751846
H	-7.653505	1.341656	2.186083
H	-8.012567	0.173489	0.918565
H	-10.276518	0.133395	4.588293
H	-8.993935	1.317863	4.359028
H	-10.080392	0.762965	2.182303
C	-10.933172	0.309432	-2.694322
C	-9.545478	0.568947	-3.300572

C	-9.118449	2.017769	-3.018590
C	-10.139762	2.980295	-3.643820
C	-11.529212	2.727117	-3.038646
C	-11.949914	1.276368	-3.320218
C	-9.067290	2.246956	-1.500155
C	-10.876901	0.542679	-1.176634
C	-11.471933	2.954005	-1.520102
C	-10.453995	1.991524	-0.889486
H	-11.235781	-0.723815	-2.896657
H	-8.812993	-0.128862	-2.877181
H	-9.568122	0.387320	-4.381984
H	-8.128612	2.200392	-3.451209
H	-9.835189	4.018868	-3.466455
H	-10.172183	2.840901	-4.731190
H	-12.255770	3.414023	-3.486222
H	-12.949630	1.088070	-2.910246
H	-12.014805	1.106848	-4.401898
H	-8.326407	1.580007	-1.042870
H	-8.744746	3.272976	-1.285487
H	-11.857373	0.340584	-0.728495
H	-10.167848	-0.154850	-0.714620
H	-11.191174	3.992191	-1.305250
H	-12.463324	2.796078	-1.078322
H	-10.414134	2.155103	0.193059
C	-3.957928	0.832126	-1.260500
C	-5.457472	0.717978	-0.945981
C	-6.018295	-0.577005	-1.553805
C	-5.813523	-0.555011	-3.076313
C	-4.314859	-0.444042	-3.396624
C	-3.758290	0.850393	-2.783750
C	-5.274732	-1.782489	-0.958228
C	-3.219880	-0.376925	-0.665161
C	-3.575457	-1.649561	-2.796004
C	-3.774586	-1.674408	-1.272857
H	-3.560181	1.756016	-0.826526
H	-5.616277	0.724407	0.139132
H	-5.993066	1.585682	-1.349938
H	-7.087361	-0.656592	-1.327860
H	-6.228100	-1.466032	-3.524669
H	-6.355821	0.290201	-3.517203
H	-4.171306	-0.427984	-4.482526
H	-2.692532	0.951804	-3.022163
H	-4.263901	1.720593	-3.219741
H	-5.429430	-1.822480	0.126809
H	-5.679443	-2.714839	-1.370255
H	-2.144452	-0.296897	-0.865278
H	-3.338810	-0.391124	0.424982
H	-3.950314	-2.579880	-3.239631
H	-2.506565	-1.592794	-3.035194
H	-3.247194	-2.534818	-0.846474
C	0.809090	3.734569	-3.429852
C	1.363081	5.030342	-2.817768
C	1.198472	4.994836	-1.290484
C	-0.291717	4.858950	-0.942173

C	-0.850293	3.562752	-1.549438
C	-0.680083	3.601731	-3.076021
C	1.964919	3.789742	-0.723828
C	1.577049	2.532923	-2.857810
C	-0.078398	2.361268	-0.982561
C	1.412287	2.490817	-1.330873
H	0.927197	3.762026	-4.518616
H	2.421419	5.148307	-3.079945
H	0.835075	5.897467	-3.232805
H	1.594606	5.919355	-0.856170
H	-0.424338	4.852588	0.146509
H	-0.848224	5.723401	-1.324114
H	-1.912864	3.468247	-1.300323
H	-1.093223	2.689910	-3.524200
H	-1.243807	4.443705	-3.495870
H	3.034466	3.884027	-0.947467
H	1.870980	3.764360	0.368584
H	1.204514	1.602184	-3.302713
H	2.639510	2.605901	-3.119743
H	-0.207501	2.310640	0.105366
H	-0.481306	1.427428	-1.393137
H	1.961067	1.633652	-0.925463
C	-1.761621	3.423688	2.594084
C	-2.204706	1.984690	2.288233
C	-3.587004	1.724565	2.906732
C	-3.509924	1.925405	4.427972
C	-3.070093	3.364265	4.739637
C	-1.689299	3.620340	4.116114
C	-4.603742	2.712579	2.314396
C	-2.783048	4.407106	2.002036
C	-4.087814	4.348208	4.142280
C	-4.166442	4.153498	2.620389
H	-0.775959	3.606962	2.152506
H	-2.240905	1.824144	1.203961
H	-1.473117	1.273054	2.689860
H	-3.901155	0.698263	2.686957
H	-4.486630	1.722939	4.883943
H	-2.801491	1.212316	4.866699
H	-3.015303	3.504993	5.824645
H	-1.354499	4.638667	4.348245
H	-0.948463	2.937530	4.549600
H	-4.684322	2.566242	1.230455
H	-5.599340	2.524285	2.734115
H	-2.467250	5.439417	2.195948
H	-2.830643	4.289974	0.912645
H	-5.074650	4.188714	4.593427
H	-3.795870	5.379407	4.375438
H	-4.892874	4.855442	2.196337
C	-2.141454	-3.133394	2.234453
C	-1.581027	-1.840190	2.846115
C	-1.781069	-1.857240	4.369482
C	-3.280810	-1.966086	4.684722
C	-3.845936	-3.259592	4.077777
C	-3.640257	-3.239150	2.555206

C	-1.047084	-3.068268	4.965692
C	-1.406025	-4.340942	2.836049
C	-3.106453	-4.467094	4.674298
C	-1.606317	-4.364592	4.359132
H	-1.998054	-3.119106	1.148478
H	-0.515225	-1.741587	2.607213
H	-2.085518	-0.969240	2.410355
H	-1.380289	-0.934593	4.803514
H	-3.438380	-1.959055	5.770070
H	-3.814590	-1.097018	4.281418
H	-4.915299	-3.334809	4.303287
H	-4.057517	-4.148978	2.106794
H	-4.180743	-2.392835	2.114064
H	0.028665	-2.993117	4.765813
H	-1.166376	-3.080823	6.055851
H	-1.783073	-5.270289	2.392037
H	-0.336823	-4.287380	2.597938
H	-3.261195	-4.504385	5.759417
H	-3.514696	-5.398707	4.263933
H	-1.080682	-5.226011	4.785646
C	0.714628	-5.044114	-5.844763
C	-0.784093	-5.166048	-5.529246
C	-1.335710	-6.470330	-6.125502
C	-1.130994	-6.460465	-7.648145
C	0.366870	-6.341768	-7.969425
C	0.914235	-5.038018	-7.368114
C	-0.583686	-7.665172	-5.519197
C	1.461161	-6.242562	-5.238664
C	1.114732	-7.536627	-7.358077
C	0.915680	-7.549300	-5.834770
H	1.105806	-4.113603	-5.419042
H	-0.943014	-5.151068	-4.444234
H	-1.325779	-4.305788	-5.940927
H	-2.404202	-6.555453	-5.898863
H	-1.539093	-7.378355	-8.088363
H	-1.679222	-5.623071	-8.096556
H	0.510380	-6.334378	-9.055426
H	1.979266	-4.931208	-7.607416
H	0.402517	-4.175335	-7.811850
H	-0.738171	-7.696579	-4.433847
H	-0.981770	-8.603994	-5.922897
H	2.536011	-6.156724	-5.439481
H	1.342261	-6.247881	-4.148438
H	0.746489	-8.473491	-7.793393
H	2.183213	-7.474443	-7.597758
H	1.449111	-8.402125	-5.400730
C	3.404561	2.244744	4.334716
C	1.906198	2.140252	4.658096
C	1.332796	0.850433	4.051029
C	1.528492	0.874208	2.527354
C	3.025918	0.975539	2.198182
C	3.595092	2.264829	2.810324
C	2.071629	-0.361381	4.639605
C	4.137853	1.029402	4.923080

C	3.760620	-0.236291	2.791826
C	3.570552	-0.262960	4.316100
H	3.811284	3.164966	4.768164
H	1.754014	2.145499	5.744164
H	1.374158	3.012463	4.259180
H	0.264595	0.777727	4.283294
H	1.104940	-0.032999	2.079653
H	0.989370	1.724052	2.091501
H	3.163000	0.992885	1.111464
H	4.660061	2.359407	2.565655
H	3.092858	3.139398	2.379197
H	1.923232	-0.402581	5.725477
H	1.658016	-1.290060	4.228128
H	5.212576	1.102450	4.716605
H	4.025431	1.013737	6.013894
H	3.376683	-1.163079	2.348573
H	4.828410	-0.186376	2.546270
H	4.094575	-1.127868	4.737506

Adamantane$_{34}$

884
E=-1620.2644

C	-1.648152	-5.912641	9.735936
C	-1.448044	-5.931139	8.212716
C	0.051922	-5.868708	7.885367
C	0.646098	-4.576022	8.465337
C	0.451417	-4.553732	9.989315
C	-1.049544	-4.619933	10.311307
C	0.760434	-7.079629	8.511842
C	-0.935505	-7.123829	10.357310
C	1.158464	-5.768206	10.610501
C	0.565693	-7.063881	10.035962
H	-2.718060	-5.956985	9.967110
H	-1.889262	-6.839910	7.785875
H	-1.968456	-5.080966	7.755190
H	0.192259	-5.882583	6.798905
H	1.713216	-4.509953	8.220898
H	0.162449	-3.702438	8.011697
H	0.875257	-3.631070	10.400537
H	-1.203560	-4.584743	11.396667
H	-1.563234	-3.746896	9.890706
H	0.360041	-8.009998	8.091208
H	1.829662	-7.056752	8.268818
H	-1.088166	-7.133527	11.443403
H	-1.366605	-8.054378	9.968735
H	2.234667	-5.723025	10.404189
H	1.044136	-5.753009	11.701218
H	1.071356	-7.928616	10.479568
C	-2.009978	-10.937279	3.999177
C	-0.635240	-10.712351	3.351034
C	-0.158091	-9.278662	3.629995
C	-1.170577	-8.282297	3.044695
C	-2.546921	-8.500831	3.691865

C	-3.017791	-9.936629	3.413099
C	-0.052446	-9.063634	5.147688
C	-1.899131	-10.718331	5.515990
C	-2.435224	-8.288276	5.209454
C	-1.425937	-9.284545	5.800245
H	-2.348371	-11.959778	3.798937
H	0.089908	-11.434219	3.745746
H	-0.697230	-10.884279	2.269577
H	0.822426	-9.120708	3.167476
H	-0.830857	-7.254323	3.219957
H	-1.241311	-8.411683	1.957905
H	-3.267225	-7.789830	3.272713
H	-4.009057	-10.099787	3.853255
H	-3.121650	-10.095251	2.332807
H	0.683050	-9.755175	5.576235
H	0.306046	-8.049029	5.359567
H	-2.870361	-10.896145	5.993579
H	-1.196165	-11.439572	5.950051
H	-2.118122	-7.260322	5.422938
H	-3.416294	-8.421577	5.681362
H	-1.347230	-9.131156	6.882154
C	3.444125	7.567196	2.598994
C	2.995896	6.140510	2.246516
C	1.612768	5.865261	2.856555
C	1.690766	6.016138	4.383513
C	2.135742	7.442509	4.741799
C	3.517351	7.713879	4.126606
C	0.599461	6.875651	2.296991
C	2.426114	8.573000	2.039569
C	1.121438	8.448997	4.177084
C	1.041913	8.304274	2.649676
H	4.430371	7.761273	2.163376
H	2.958980	6.015518	1.157596
H	3.725010	5.413575	2.624551
H	1.294947	4.847787	2.603525
H	0.713410	5.802379	4.832889
H	2.396724	5.286624	4.798588
H	2.191178	7.547603	5.830803
H	3.855794	8.722906	4.391720
H	4.255819	7.014683	4.537421
H	0.518216	6.765008	1.208876
H	-0.396741	6.677305	2.710600
H	2.745599	9.597318	2.266944
H	2.377956	8.491607	0.946951
H	0.134103	8.278380	4.623041
H	1.417070	9.471008	4.443654
H	0.317918	9.022235	2.248909
C	-1.219120	8.137591	8.917737
C	-1.006514	8.159276	7.396253
C	-1.578157	6.876006	6.773841
C	-0.862596	5.656396	7.374650
C	-1.074610	5.628073	8.896213
C	-0.504623	6.914710	9.513162
C	-3.080787	6.786527	7.081878

C	-2.722819	8.045300	9.219930
C	-2.578505	5.542250	9.198978
C	-3.298435	6.761464	8.602771
H	-0.810724	9.053121	9.359468
H	-1.494648	9.039247	6.960238
H	0.062829	8.244040	7.167620
H	-1.425991	6.893872	5.689031
H	-1.248200	4.734169	6.923358
H	0.209064	5.697473	7.145058
H	-0.563179	4.758123	9.322444
H	-0.631873	6.898254	10.602429
H	0.573508	6.977926	9.321811
H	-3.607724	7.641221	6.640663
H	-3.504852	5.883604	6.626102
H	-2.889497	8.049718	10.303995
H	-3.242454	8.922643	8.816189
H	-2.994337	4.617864	8.780155
H	-2.742471	5.500809	10.282659
H	-4.370553	6.698277	8.819151
C	-6.350614	4.057064	8.958226
C	-7.852760	3.971924	9.269505
C	-8.437857	2.689630	8.657675
C	-8.229593	2.710907	7.135630
C	-6.728378	2.792899	6.818551
C	-6.147556	4.074719	7.435422
C	-7.719503	1.468363	9.252036
C	-5.637844	2.832342	9.552331
C	-6.014195	1.571670	7.417948
C	-6.216875	1.547426	8.940636
H	-5.935549	4.971948	9.395054
H	-8.013614	3.979113	10.354312
H	-8.370259	4.850941	8.866422
H	-9.508745	2.630719	8.881313
H	-8.661201	1.809254	6.684416
H	-8.754164	3.567651	6.695551
H	-6.582327	2.808496	5.732976
H	-5.079517	4.155542	7.199358
H	-6.634963	4.955709	7.000370
H	-7.877168	1.429061	10.336671
H	-8.141742	0.545111	8.837122
H	-4.560638	2.891510	9.354535
H	-5.759249	2.818107	10.642201
H	-6.406482	0.649926	6.971498
H	-4.943905	1.607797	7.181013
H	-5.707477	0.675815	9.366144
C	5.583751	4.851713	8.198943
C	5.138084	3.419051	7.868120
C	3.769502	3.140154	8.508552
C	3.877445	3.305063	10.032233
C	4.319979	4.737440	10.368931
C	5.686963	5.012403	9.723411
C	2.738268	4.138879	7.960926
C	4.547846	5.845823	7.651592
C	3.287669	5.732211	9.816210

C	3.178150	5.573442	8.292057
H	6.559626	5.048387	7.741673
H	5.079927	3.284176	6.781309
H	5.879598	2.700292	8.237571
H	3.453476	2.118416	8.270929
H	2.910869	3.088889	10.503249
H	4.596577	2.583900	10.439208
H	4.396787	4.852541	11.455624
H	6.023909	6.025938	9.972770
H	6.438307	4.321733	10.125202
H	2.635712	4.018068	6.875697
H	1.752021	3.937676	8.396433
H	4.864971	6.874189	7.863473
H	4.478081	5.754438	6.560931
H	2.310756	5.559081	10.283635
H	3.581756	6.758461	10.067782
H	2.441353	6.283060	7.899855
C	-3.472059	10.750191	3.379407
C	-4.973314	10.601370	3.670387
C	-5.509029	9.328555	2.996662
C	-5.287394	9.423951	1.479354
C	-3.786908	9.569855	1.182432
C	-3.255497	10.841831	1.861125
C	-4.755421	8.106856	3.544674
C	-2.723913	9.524867	3.926951
C	-3.037518	8.347831	1.735430
C	-3.253477	8.249631	3.253311
H	-3.092228	11.658219	3.860370
H	-5.144445	10.555285	4.752664
H	-5.516417	11.479592	3.300634
H	-6.579359	9.224167	3.205906
H	-5.684070	8.529219	0.984421
H	-5.836467	10.281332	1.071591
H	-3.631333	9.638231	0.100226
H	-2.188639	10.968551	1.640397
H	-3.768295	11.724437	1.459848
H	-4.921794	8.014464	4.624776
H	-5.142344	7.189267	3.085160
H	-1.647519	9.628582	3.743602
H	-2.854943	9.458482	5.013774
H	-3.394263	7.434138	1.244802
H	-1.966892	8.430038	1.511898
H	-2.718930	7.377543	3.645704
C	-8.952055	5.457800	1.220962
C	-8.401681	6.738748	1.866597
C	-8.604424	6.681398	3.388622
C	-10.103961	6.554279	3.697936
C	-10.659040	5.272877	3.056998
C	-10.450686	5.333635	1.535866
C	-7.863222	5.460573	3.955187
C	-8.209444	4.240325	1.793002
C	-9.912366	4.055605	3.623975
C	-8.412390	4.176384	3.314574
H	-8.806744	5.500822	0.135999

H	-7.336142	6.850710	1.632455
H	-8.911350	7.617083	1.452207
H	-8.210812	7.595368	3.846896
H	-10.263587	6.532489	4.782787
H	-10.642965	7.429703	3.315884
H	-11.728276	5.184611	3.278344
H	-10.860838	4.432735	1.063512
H	-10.996168	6.187235	1.115395
H	-6.787647	5.548162	3.759476
H	-7.984444	5.419342	5.044425
H	-8.579259	3.320075	1.324628
H	-7.140197	4.307265	1.558506
H	-10.068858	3.989534	4.707471
H	-10.313423	3.132016	3.189117
H	-7.881626	3.307958	3.720008
C	10.204994	-1.076917	3.573604
C	9.718963	-2.497031	3.245529
C	8.339327	-2.732972	3.879476
C	8.443932	-2.565573	5.403117
C	8.926792	-1.145629	5.737051
C	10.304742	-0.913593	5.098023
C	7.341005	-1.706302	3.322615
C	9.201866	-0.054721	3.017010
C	7.927305	-0.122899	5.175104
C	7.821308	-0.284116	3.650959
H	11.188691	-0.910875	3.120966
H	9.662669	-2.634230	2.158911
H	10.436923	-3.235976	3.621623
H	7.994522	-3.745821	3.643823
H	7.468881	-2.751330	5.869622
H	9.139248	-3.306118	5.816621
H	9.001146	-1.028747	6.823724
H	10.670153	0.090483	5.345554
H	11.033185	-1.624629	5.506407
H	7.240738	-1.828126	2.237285
H	6.346924	-1.876647	3.753450
H	9.548103	0.964623	3.226904
H	9.135274	-0.148122	1.926322
H	6.943213	-0.265339	5.637805
H	8.250239	0.895160	5.424569
H	7.107914	0.445466	3.252173
C	8.650158	6.359382	4.266736
C	7.168478	6.261019	4.661112
C	6.563600	4.969610	4.088977
C	6.687138	4.984559	2.557650
C	8.167601	5.079734	2.157550
C	8.768494	6.370653	2.734957
C	7.326194	3.758849	4.648709
C	9.407199	5.145109	4.826537
C	8.926308	3.868997	2.722539
C	8.808412	3.851140	4.254214
H	9.079357	7.280747	4.675374
H	7.067801	6.272593	5.753112
H	6.620504	7.132606	4.282942

H	5.507378	4.901281	4.371845
H	6.240492	4.076181	2.135499
H	6.130285	5.833601	2.143043
H	8.253222	5.090792	1.065490
H	9.820959	6.460795	2.439731
H	8.248776	7.244336	2.323191
H	7.229168	3.723966	5.740590
H	6.891130	2.829167	4.262390
H	10.471154	5.213900	4.569120
H	9.346408	5.135681	5.921514
H	8.519389	2.940946	2.303067
H	9.981430	3.914464	2.426541
H	9.349461	2.987001	4.655187
C	3.521289	-1.821774	9.670945
C	3.721722	-1.826356	8.147662
C	5.221904	-1.764243	7.821245
C	5.818868	-0.478436	8.413514
C	5.623872	-0.470082	9.937592
C	4.122688	-0.535912	10.258617
C	5.927519	-2.982624	8.436424
C	4.231040	-3.040382	10.281022
C	6.328016	-1.691962	10.547448
C	5.732448	-2.980812	9.960582
H	2.451229	-1.865870	9.901456
H	3.278548	-2.730057	7.712171
H	3.203347	-0.970727	7.698062
H	5.362470	-1.768187	6.734731
H	6.886193	-0.412486	8.169943
H	5.337309	0.400483	7.968029
H	6.049703	0.447696	10.357593
H	3.968491	-0.510613	11.344226
H	3.611078	0.342217	9.846157
H	5.525119	-3.908073	8.006944
H	6.996855	-2.959881	8.193861
H	4.078097	-3.059979	11.366941
H	3.797926	-3.966244	9.883591
H	7.404367	-1.647278	10.341807
H	6.213460	-1.686795	11.638235
H	6.236043	-3.850838	10.396122
C	-4.777479	-3.377922	8.245622
C	-5.774782	-2.381213	7.635261
C	-7.164650	-2.591728	8.255667
C	-7.635772	-4.026692	7.973940
C	-6.643304	-5.027928	8.585037
C	-5.254677	-4.810926	7.964094
C	-7.082700	-2.372492	9.774222
C	-4.701574	-3.156109	9.764169
C	-6.562237	-4.802278	10.102681
C	-6.088490	-3.369326	10.389836
H	-3.787432	-3.226313	7.801850
H	-5.435453	-1.353298	7.811266
H	-5.823824	-2.517126	6.548028
H	-7.873921	-1.879718	7.819474
H	-8.636303	-4.183438	8.394922

H	-7.717694	-4.190860	6.892648
H	-6.980878	-6.050214	8.382497
H	-4.540397	-5.533231	8.377669
H	-5.294286	-4.989373	6.882629
H	-6.767083	-1.344624	9.990344
H	-8.073460	-2.501152	10.226633
H	-3.976881	-3.848506	10.209594
H	-4.343748	-2.141933	9.979032
H	-7.543744	-4.973010	10.561280
H	-5.871631	-5.524856	10.554243
H	-6.031818	-3.210126	11.472284
C	-11.213909	-4.740101	-1.548185
C	-10.684237	-3.428701	-0.947966
C	-9.179228	-3.299527	-1.229417
C	-8.439377	-4.488675	-0.597755
C	-8.963187	-5.803050	-1.196880
C	-10.468826	-5.925642	-0.915871
C	-8.943266	-3.302407	-2.747674
C	-10.972951	-4.738773	-3.065711
C	-8.728261	-5.799476	-2.715277
C	-9.468735	-4.613670	-3.352309
H	-12.286843	-4.830322	-1.346238
H	-11.222215	-2.574235	-1.375983
H	-10.867581	-3.409477	0.133222
H	-8.803854	-2.363616	-0.801104
H	-7.360852	-4.397465	-0.774734
H	-8.583278	-4.487398	0.489510
H	-8.434736	-6.649422	-0.744725
H	-10.851743	-6.869996	-1.321502
H	-10.648703	-5.950227	0.165803
H	-9.449303	-2.446011	-3.209569
H	-7.873443	-3.191273	-2.962059
H	-11.365536	-5.661713	-3.509621
H	-11.515281	-3.907516	-3.532241
H	-7.654819	-5.731710	-2.929632
H	-9.079733	-6.741863	-3.152701
H	-9.299656	-4.612440	-4.434742
C	-4.149485	-8.251110	-1.625749
C	-2.768892	-8.012393	-2.256273
C	-2.309963	-6.573849	-1.971792
C	-3.324921	-5.587994	-2.570455
C	-4.707173	-5.820333	-1.940927
C	-5.159805	-7.260922	-2.225132
C	-2.226016	-6.357336	-0.452955
C	-4.060367	-8.030625	-0.107727
C	-4.617150	-5.606232	-0.422115
C	-3.605462	-6.592022	0.181997
H	-4.474867	-9.277052	-1.829902
H	-2.041574	-8.726740	-1.851985
H	-2.815231	-8.185243	-3.338367
H	-1.325240	-6.406060	-2.421743
H	-2.997947	-4.556570	-2.391264
H	-3.380367	-5.718394	-3.658010
H	-5.429222	-5.116810	-2.369587

H	-6.154934	-7.434030	-1.797678
H	-5.248158	-7.420893	-3.306605
H	-1.489096	-7.041244	-0.014714
H	-1.880608	-5.339079	-0.236902
H	-5.035795	-8.218173	0.357420
H	-3.355741	-8.744559	0.335622
H	-4.313282	-4.575047	-0.204996
H	-5.602797	-5.749371	0.037206
H	-3.542225	-6.437541	1.264765
C	-1.119422	-1.411241	-7.325337
C	-2.150255	-0.438060	-7.917694
C	-1.742034	1.008403	-7.598234
C	-1.677267	1.194298	-6.074523
C	-0.645269	0.225291	-5.477102
C	-1.056066	-1.219155	-5.802325
C	-0.359018	1.292611	-8.204273
C	0.261818	-1.120827	-7.932613
C	0.734901	0.511079	-6.088812
C	0.676277	0.323545	-7.612505
H	-1.412416	-2.441763	-7.554000
H	-2.220666	-0.578643	-9.003056
H	-3.144830	-0.647799	-7.505646
H	-2.478046	1.700512	-8.021975
H	-1.406078	2.229220	-5.833175
H	-2.663760	1.013509	-5.630673
H	-0.601189	0.359198	-4.390744
H	-0.337578	-1.922526	-5.364136
H	-2.031419	-1.442880	-5.353313
H	-0.395633	1.183667	-9.294973
H	-0.064569	2.329023	-7.999378
H	1.003551	-1.822973	-7.532836
H	0.235131	-1.272760	-9.018413
H	1.048805	1.533836	-5.847461
H	1.485247	-0.161188	-5.655218
H	1.660976	0.528238	-8.046922
C	1.015696	-4.098344	-1.672099
C	2.395575	-3.839353	-2.296150
C	2.841405	-2.400824	-1.991479
C	1.819292	-1.415867	-2.579350
C	0.437702	-1.668482	-1.956213
C	-0.001814	-3.109005	-2.260622
C	2.920087	-2.203854	-0.469703
C	1.099517	-3.897347	-0.151072
C	0.522482	-1.473871	-0.434480
C	1.541311	-2.458865	0.158834
H	0.699658	-5.124242	-1.890647
H	3.128143	-4.552727	-1.899699
H	2.353147	-3.998161	-3.380555
H	3.825640	-2.218571	-2.436846
H	2.136930	-0.384141	-2.385685
H	1.767402	-1.532236	-3.668671
H	-0.289450	-0.965561	-2.377166
H	-0.996359	-3.296367	-1.837843
H	-0.086368	-3.255306	-3.344331

H	3.661918	-2.887162	-0.038873
H	3.256190	-1.185627	-0.239311
H	0.124713	-4.099480	0.309249
H	1.809303	-4.611025	0.284389
H	0.816931	-0.443089	-0.202947
H	-0.462912	-1.631611	0.020584
H	1.600794	-2.318286	1.243707
C	8.018093	-3.600347	-0.315053
C	6.518270	-3.701547	0.002554
C	5.937522	-4.973790	-0.633907
C	6.137070	-4.918039	-2.156247
C	7.635948	-4.819865	-2.479717
C	8.212453	-3.548233	-1.838203
C	6.666610	-6.203260	-0.070323
C	8.741616	-4.833300	0.248229
C	8.360893	-6.049433	-1.911075
C	8.166951	-6.108129	-0.388187
H	8.430057	-2.692656	0.139361
H	6.363493	-3.719011	1.088128
H	5.993185	-2.817168	-0.378381
H	4.868288	-5.044189	-0.405719
H	5.708402	-5.812291	-2.624664
H	5.604839	-4.055164	-2.574615
H	7.775780	-4.779697	-3.565480
H	9.278634	-3.455675	-2.078320
H	7.717266	-2.660998	-2.251221
H	6.515302	-6.267179	1.014046
H	6.247646	-7.119839	-0.502972
H	9.817311	-4.763169	0.045847
H	8.626446	-4.872048	1.338180
H	7.971693	-6.963632	-2.375384
H	9.429592	-6.001526	-2.153051
H	8.684017	-6.985644	0.015373
C	-6.209186	3.523631	-6.941687
C	-5.409830	2.342980	-6.369256
C	-5.929069	1.026094	-6.966881
C	-7.415148	0.858408	-6.614887
C	-8.219954	2.035253	-7.187800
C	-7.694600	3.349596	-6.589928
C	-5.769493	1.062612	-8.494622
C	-6.048326	3.553703	-8.469485
C	-8.054243	2.069592	-8.714920
C	-6.569851	2.240264	-9.072266
H	-5.837614	4.461350	-6.514221
H	-4.343706	2.464889	-6.595641
H	-5.501527	2.321955	-5.276507
H	-5.357385	0.185781	-6.557812
H	-7.792813	-0.088540	-7.019257
H	-7.541474	0.810985	-5.526471
H	-9.278965	1.913889	-6.935185
H	-8.274260	4.196903	-6.975972
H	-7.826228	3.346513	-5.501055
H	-4.710153	1.161351	-8.760866
H	-6.119181	0.119504	-8.931772

H	-6.598116	4.405106	-8.888820
H	-4.993684	3.696559	-8.734213
H	-8.443466	1.144157	-9.156386
H	-8.640801	2.893811	-9.138685
H	-6.453599	2.263989	-10.161382
C	8.045096	1.760845	-2.013563
C	7.618882	0.326175	-2.360903
C	6.235417	0.035454	-1.758858
C	6.300675	0.196036	-0.232292
C	6.723600	1.630380	0.120835
C	8.105635	1.917172	-0.486351
C	5.212329	1.028672	-2.331221
C	7.017366	2.749415	-2.585793
C	5.699604	2.619653	-0.456663
C	5.632738	2.465201	-1.983725
H	9.031617	1.965952	-2.443467
H	7.591287	0.194521	-3.449314
H	8.355204	-0.388544	-1.973657
H	5.933308	-0.987698	-2.008211
H	5.323216	-0.028551	0.211545
H	7.013621	-0.521370	0.191846
H	6.769984	1.742393	1.209573
H	8.428395	2.932230	-0.224724
H	8.850702	1.230504	-0.066423
H	5.140208	0.910762	-3.419216
H	4.216065	0.819036	-1.923369
H	7.321225	3.779287	-2.362121
H	6.977960	2.661179	-3.678230
H	4.711602	2.438048	-0.016562
H	5.979370	3.647115	-0.193950
H	4.901816	3.170888	-2.393613
C	-6.788540	-8.457424	4.427512
C	-8.294610	-8.570505	4.709561
C	-9.031816	-7.379068	4.078721
C	-8.493906	-6.071508	4.679875
C	-6.988220	-5.951774	4.397951
C	-6.256277	-7.147065	5.027622
C	-8.791538	-7.377515	2.561139
C	-6.554335	-8.453562	2.908934
C	-6.752979	-5.954321	2.879601
C	-7.286508	-7.261481	2.273858
H	-6.264880	-9.307560	4.878244
H	-8.684106	-9.512066	4.303991
H	-8.473442	-8.593250	5.791436
H	-10.105436	-7.462045	4.280565
H	-9.026672	-5.213727	4.251822
H	-8.676805	-6.050689	5.761064
H	-6.606806	-5.019178	4.827878
H	-5.177115	-7.063369	4.850505
H	-6.399187	-7.145114	6.115074
H	-9.189624	-8.297112	2.115376
H	-9.328897	-6.542048	2.096303
H	-5.480404	-8.393862	2.694212
H	-6.913124	-9.392528	2.470331

H	-7.254758	-5.094305	2.419809
H	-5.682685	-5.848827	2.664409
H	-7.118414	-7.261265	1.191271
C	-5.766927	4.420772	-2.745809
C	-6.779675	5.400120	-3.358837
C	-6.359813	6.844664	-3.045850
C	-6.312056	7.042237	-1.522980
C	-5.298161	6.067091	-0.904908
C	-5.720504	4.624545	-1.223706
C	-4.966341	7.109062	-3.636714
C	-4.375149	4.691384	-3.337982
C	-3.907446	6.333054	-1.501490
C	-3.949081	6.133776	-3.024251
H	-6.068201	3.391630	-2.969888
H	-6.838056	5.251480	-4.443839
H	-7.781531	5.204434	-2.957657
H	-7.082932	7.541171	-3.484328
H	-6.032694	8.076116	-1.286528
H	-7.305913	6.875671	-1.090132
H	-5.266193	6.209347	0.180813
H	-5.015209	3.917083	-0.770911
H	-6.703754	4.414968	-0.785212
H	-4.990521	6.991652	-4.726882
H	-4.663252	8.143884	-3.436429
H	-3.646120	3.984586	-2.923313
H	-4.389928	4.530922	-4.422783
H	-3.585508	7.354309	-1.264401
H	-3.169904	5.656314	-1.053139
H	-2.956875	6.324340	-3.447883
C	-6.269305	-5.558909	-7.280784
C	-4.877871	-5.296611	-7.877187
C	-4.447590	-3.852691	-7.575683
C	-5.462788	-2.879381	-8.194140
C	-6.855952	-3.135322	-7.598745
C	-7.279914	-4.581195	-7.899832
C	-4.403050	-3.642001	-6.054362
C	-6.219635	-5.344167	-5.760138
C	-6.805300	-2.926953	-6.077315
C	-5.793502	-3.900272	-5.453487
H	-6.574226	-6.588652	-7.497032
H	-4.149675	-6.001853	-7.458741
H	-4.895860	-5.465108	-8.960809
H	-3.455093	-3.668081	-8.001307
H	-5.155765	-1.844018	-8.002785
H	-5.490391	-3.005544	-9.283257
H	-7.578139	-2.440712	-8.041476
H	-8.282199	-4.771175	-7.496968
H	-7.340110	-4.737447	-8.983779
H	-3.666522	-4.316820	-5.601613
H	-4.078277	-2.619698	-5.825774
H	-7.202905	-5.548465	-5.319184
H	-5.515057	-6.049517	-5.303181
H	-6.522272	-1.892352	-5.848595
H	-7.799316	-3.086974	-5.642239

H	-5.758360	-3.749891	-4.368867
C	5.018302	-5.165169	3.601963
C	4.550361	-6.599129	3.309773
C	3.171907	-6.835229	3.946227
C	3.270229	-6.627007	5.465248
C	3.734984	-5.193103	5.763292
C	5.111814	-4.961059	5.121855
C	2.162828	-5.835579	3.360351
C	4.004470	-4.170043	3.016458
C	2.724802	-4.197538	5.172431
C	2.625015	-4.399611	3.652742
H	6.001183	-4.999029	3.147595
H	4.498761	-6.765213	2.226959
H	5.276132	-7.319276	3.706757
H	2.840008	-7.857974	3.736173
H	2.296191	-6.812388	5.934013
H	3.973273	-7.348054	5.899615
H	3.804887	-5.047107	6.846734
H	5.464388	-3.946525	5.344064
H	5.847640	-5.652342	5.550419
H	2.067069	-5.986794	2.278316
H	1.169675	-6.006713	3.793011
H	4.337793	-3.141459	3.200597
H	3.942057	-4.292578	1.928410
H	1.741212	-4.339829	5.636244
H	3.034730	-3.169491	5.396104
H	1.903986	-3.689358	3.233329
C	2.886382	-7.719763	-0.226448
C	1.381405	-7.836915	0.059735
C	0.832650	-9.127857	-0.567392
C	1.065258	-9.094986	-2.085701
C	2.569464	-8.980973	-2.377713
C	3.113872	-7.690616	-1.745618
C	1.565965	-10.337137	0.033175
C	3.614190	-8.932610	0.373756
C	3.298526	-10.190324	-1.772179
C	3.071552	-10.226050	-0.253149
H	3.275514	-6.798758	0.221255
H	1.202748	-7.838245	1.141776
H	0.852734	-6.966654	-0.347826
H	-0.240322	-9.209598	-0.361637
H	0.659554	-10.003029	-2.548000
H	0.530575	-8.246959	-2.530469
H	2.732870	-8.957158	-3.460662
H	4.183752	-7.586965	-1.963745
H	2.615765	-6.817617	-2.184640
H	1.391448	-10.384884	1.114887
H	1.169475	-11.266759	-0.392772
H	4.693051	-8.850608	0.193943
H	3.475324	-8.954582	1.461410
H	2.932462	-11.117678	-2.229159
H	4.371583	-10.131310	-1.991342
H	3.591606	-11.089180	0.176735
C	0.407949	0.738540	8.215047

C	-0.614083	1.732507	7.642276
C	-1.990027	1.488301	8.281098
C	-2.444666	0.052528	7.977277
C	-1.427390	-0.946052	8.550770
C	-0.052791	-0.695369	7.911566
C	-1.885682	1.677327	9.802339
C	0.506211	0.930050	9.736433
C	-1.324038	-0.750608	10.071248
C	-0.866652	0.683080	10.380410
H	1.388021	0.914184	7.758192
H	-0.286996	2.761562	7.834086
H	-0.679470	1.618362	6.553401
H	-2.716965	2.198403	7.871706
H	-3.435530	-0.127939	8.411417
H	-2.542410	-0.090480	6.894295
H	-1.753253	-1.968906	8.332522
H	0.678925	-1.415221	8.298051
H	-0.108025	-0.852012	6.827411
H	-1.581623	2.705149	10.034644
H	-2.866659	1.524434	10.268321
H	1.248436	0.239596	10.155209
H	0.852648	1.944847	9.966494
H	-2.295041	-0.945548	10.542383
H	-0.615344	-1.471875	10.496092
H	-0.794060	0.820718	11.464861
C	-6.952310	-0.669518	2.616878
C	-7.395867	-2.112879	2.333061
C	-8.780851	-2.361266	2.950375
C	-8.709435	-2.135458	4.468387
C	-8.269155	-0.692136	4.758007
C	-6.885661	-0.447880	4.135730
C	-9.794144	-1.382043	2.337881
C	-7.970299	0.305119	2.004729
C	-9.283417	0.282934	4.140556
C	-9.356371	0.063248	2.621786
H	-5.964733	-0.494623	2.176181
H	-7.428048	-2.291238	1.251449
H	-6.666662	-2.818594	2.749208
H	-9.095332	-3.390707	2.746323
H	-9.688134	-2.329315	4.923826
H	-8.003532	-2.841990	4.921563
H	-8.218409	-0.533600	5.840753
H	-6.550588	0.573770	4.352348
H	-6.147299	-1.124258	4.583292
H	-9.870691	-1.546141	1.256193
H	-10.791578	-1.562307	2.756766
H	-7.654063	1.340142	2.182829
H	-8.013806	0.170096	0.917239
H	-10.272178	0.131975	4.590424
H	-8.991190	1.317518	4.357821
H	-10.080343	0.758897	2.183397
C	-10.945088	0.304421	-2.678227
C	-9.559444	0.562926	-3.289577
C	-9.131631	2.012321	-3.011758

C	-10.155118	2.973561	-3.635409
C	-11.542522	2.721392	-3.025141
C	-11.964006	1.270072	-3.302568
C	-9.075449	2.244385	-1.493937
C	-10.883796	0.540545	-1.161179
C	-11.480220	2.951158	-1.507228
C	-10.460089	1.989972	-0.878179
H	-11.248259	-0.729236	-2.877600
H	-8.825483	-0.134009	-2.867304
H	-9.585663	0.379251	-4.370562
H	-8.143257	2.194222	-3.448009
H	-9.850066	4.012498	-3.461022
H	-10.191139	2.832107	-4.722399
H	-12.270635	3.407378	-3.471597
H	-12.962334	1.082451	-2.888919
H	-12.032475	1.098498	-4.383703
H	-8.332981	1.578376	-1.037857
H	-8.752301	3.270841	-1.282284
H	-11.862752	0.339202	-0.709401
H	-10.173138	-0.156038	-0.700205
H	-11.198856	3.989776	-1.295274
H	-12.470119	2.793970	-1.061857
H	-10.416648	2.155604	0.203915
C	-3.965420	0.830362	-1.271405
C	-5.463374	0.712482	-0.950745
C	-6.023044	-0.584816	-1.554681
C	-5.824419	-0.564201	-3.078024
C	-4.327360	-0.449511	-3.404467
C	-3.771923	0.847235	-2.795462
C	-5.273780	-1.787485	-0.960559
C	-3.221662	-0.375889	-0.677493
C	-3.582237	-1.652219	-2.805284
C	-3.775207	-1.675671	-1.281323
H	-3.568494	1.755898	-0.840194
H	-5.617858	0.719858	0.134986
H	-6.002972	1.578190	-1.353654
H	-7.090974	-0.667061	-1.324362
H	-6.238268	-1.476933	-3.523564
H	-6.370807	0.278950	-3.517809
H	-4.188193	-0.434443	-4.490954
H	-2.707410	0.951278	-3.038265
H	-4.281673	1.715481	-3.230528
H	-5.424029	-1.826517	0.125138
H	-5.677559	-2.721472	-1.369784
H	-2.147267	-0.293154	-0.882011
H	-3.336194	-0.389025	0.413134
H	-3.956294	-2.584134	-3.246231
H	-2.514470	-1.592812	-3.048820
H	-3.243742	-2.534079	-0.855965
C	0.818130	3.719631	-3.446171
C	1.372689	5.016090	-2.836056
C	1.209279	4.982402	-1.308602
C	-0.280644	4.847028	-0.958958
C	-0.839783	3.550151	-1.564250

C	-0.670773	3.587312	-3.091011
C	1.976092	3.777928	-0.741124
C	1.586459	2.518611	-2.873312
C	-0.067523	2.349286	-0.996559
C	1.422896	2.478323	-1.346197
H	0.935382	3.745792	-4.535059
H	2.430828	5.133673	-3.099205
H	0.844414	5.882759	-3.251703
H	1.605817	5.907407	-0.875694
H	-0.412408	4.841964	0.129835
H	-0.837394	5.711064	-1.341483
H	-1.902165	3.456012	-1.314187
H	-1.084325	2.674990	-3.537785
H	-1.234771	4.428827	-3.511412
H	3.045470	3.871876	-0.965716
H	1.883011	3.753844	0.351391
H	1.213512	1.587371	-3.316820
H	2.648719	2.591207	-3.136167
H	-0.195773	2.299954	0.091529
H	-0.470816	1.414988	-1.405713
H	1.971938	1.621600	-0.940205
C	-1.754815	3.420951	2.581477
C	-2.197955	1.981678	2.277001
C	-3.579744	1.721824	2.896750
C	-3.501518	1.923767	4.417785
C	-3.061628	3.362906	4.728079
C	-1.681347	3.618706	4.103309
C	-4.597061	2.709285	2.304487
C	-2.776822	4.403816	1.989509
C	-4.079933	4.346291	4.130799
C	-4.159707	4.150479	2.609109
H	-0.769517	3.604031	2.139009
H	-2.234968	1.820349	1.192872
H	-1.465968	1.270423	2.678576
H	-3.893935	0.695326	2.677954
H	-4.477847	1.721506	4.874654
H	-2.792658	1.211081	4.856480
H	-3.006022	3.504420	5.812943
H	-1.346496	4.637241	4.334451
H	-0.940092	2.936300	4.536716
H	-4.678457	2.562160	1.220713
H	-5.592312	2.521168	2.725107
H	-2.461004	5.436306	2.182437
H	-2.825240	4.285896	0.900239
H	-5.066401	4.186998	4.582820
H	-3.787939	5.377694	4.362991
H	-4.886554	4.852027	2.185112
C	-2.138670	-3.132537	2.231575
C	-1.577990	-1.839543	2.843447
C	-1.778058	-1.856790	4.366808
C	-3.277826	-1.965381	4.682009
C	-3.843206	-3.258677	4.074853
C	-3.637500	-3.238038	2.552288
C	-1.044328	-3.068061	4.962841

C	-1.403496	-4.340329	2.832993
C	-3.103977	-4.466423	4.671196
C	-1.603815	-4.364176	4.356068
H	-1.995251	-3.118108	1.145604
H	-0.512163	-1.741119	2.604577
H	-2.082297	-0.968422	2.407816
H	-1.377097	-0.934293	4.800991
H	-3.435411	-1.958487	5.767356
H	-3.811423	-1.096141	4.278833
H	-4.912587	-3.333711	4.300335
H	-4.054937	-4.147711	2.103727
H	-4.177806	-2.391545	2.111271
H	0.031440	-2.993097	4.762989
H	-1.163639	-3.080762	6.052995
H	-1.780725	-5.269529	2.388829
H	-0.334279	-4.286947	2.594906
H	-3.258742	-4.503851	5.756307
H	-3.512403	-5.397889	4.260680
H	-1.078362	-5.225769	4.782456
C	3.761712	-2.277663	-6.205050
C	4.359127	-1.014063	-5.567089
C	5.859663	-0.930706	-5.887086
C	6.569318	-2.172947	-5.327016
C	5.977671	-3.439788	-5.964178
C	4.476753	-3.516530	-5.644334
C	6.053081	-0.878306	-7.410448
C	3.960305	-2.221284	-7.727656
C	6.170303	-3.380898	-7.487385
C	5.459745	-2.141744	-8.052937
H	2.692077	-2.335279	-5.975548
H	3.843573	-0.122299	-5.943510
H	4.206775	-1.033648	-4.481107
H	6.283385	-0.028805	-5.431761
H	7.645270	-2.115614	-5.531705
H	6.455824	-2.212214	-4.236864
H	6.484699	-4.324128	-5.563037
H	4.045793	-4.427532	-6.077132
H	4.326151	-3.580006	-4.559775
H	5.568565	0.015598	-7.821563
H	7.120087	-0.799166	-7.651419
H	3.519471	-3.108916	-8.197518
H	3.438420	-1.350895	-8.143369
H	7.239216	-3.345196	-7.730219
H	5.769947	-4.289792	-7.952779
H	5.598621	-2.101140	-9.138900
C	0.716876	-5.040218	-5.816981
C	-0.788027	-5.149024	-5.527129
C	-1.343567	-6.441600	-6.144846
C	-1.113807	-6.419521	-7.663783
C	0.390298	-6.313888	-7.959442
C	0.941499	-5.021846	-7.336745
C	-0.614284	-7.650139	-5.537909
C	1.440635	-6.252302	-5.210374
C	1.115337	-7.522448	-7.347531

C	0.891200	-7.547402	-5.827865
H	1.110849	-4.118036	-5.375987
H	-0.964558	-5.142609	-4.444758
H	-1.313747	-4.279133	-5.939272
H	-2.416474	-6.517380	-5.936487
H	-1.524325	-7.328764	-8.119424
H	-1.645708	-5.572077	-8.112977
H	0.551675	-6.297760	-9.042838
H	2.011384	-4.924224	-7.557612
H	0.446292	-4.149552	-7.780426
H	-0.786877	-7.690158	-4.455576
H	-1.015611	-8.580766	-5.957079
H	2.519483	-6.176121	-5.392805
H	1.303816	-6.266665	-4.122333
H	0.744386	-8.451138	-7.797806
H	2.188205	-7.469481	-7.569151
H	1.408379	-8.409980	-5.393429
C	3.412812	2.242203	4.331973
C	1.914534	2.137410	4.655648
C	1.341079	0.847912	4.047948
C	1.536411	0.872612	2.524240
C	3.033751	0.974248	2.194772
C	3.602978	2.263213	2.807548
C	2.080140	-0.364200	4.635623
C	4.146332	1.026562	4.919437
C	3.768682	-0.237885	2.787517
C	3.578979	-0.265478	4.311819
H	3.819573	3.162195	4.765874
H	1.762608	2.141997	5.741755
H	1.382336	3.009821	4.257380
H	0.272939	0.774990	4.280424
H	1.112818	-0.034358	2.076098
H	0.997124	1.722677	2.089024
H	3.170573	0.992252	1.108032
H	4.667882	2.358014	2.562682
H	3.100578	3.138002	2.377064
H	1.932005	-0.406059	5.721505
H	1.666496	-1.292663	4.223690
H	5.221000	1.099812	4.712749
H	4.034171	1.010237	6.010268
H	3.384705	-1.164435	2.343801
H	4.836409	-0.187745	2.541736
H	4.103165	-1.130599	4.732584

Adamantane$_{35}$

910

E=-1672.3350

C	5.855569	4.446864	-7.673589
C	5.464073	2.993785	-7.983228
C	4.105513	2.673955	-7.340595
C	4.204208	2.865815	-5.819446
C	4.592573	4.318582	-5.503932
C	5.949692	4.634345	-6.151594

C	3.039001	3.625263	-7.904827
C	4.784561	5.393391	-8.237482
C	3.525143	5.265821	-6.073200
C	3.424645	5.080030	-7.594918
H	6.824382	4.672718	-8.132394
H	5.413139	2.840400	-9.067945
H	6.231024	2.308754	-7.601824
H	3.828121	1.637698	-7.563107
H	3.245385	2.621030	-5.346586
H	4.948807	2.178119	-5.400518
H	4.662879	4.452900	-4.419006
H	6.248271	5.663360	-5.917193
H	6.725360	3.978235	-5.738268
H	2.943173	3.484305	-8.988240
H	2.060036	3.394168	-7.467728
H	5.062856	6.435921	-8.040808
H	4.720421	5.282971	-9.326726
H	2.554400	5.063607	-5.604601
H	3.780416	6.305983	-5.836841
H	2.662793	5.755758	-7.998925
C	-1.648346	-5.913180	9.736831
C	-1.448654	-5.930809	8.213547
C	0.051219	-5.868127	7.885820
C	0.645497	-4.575742	8.466357
C	0.451234	-4.554321	9.990401
C	-1.049636	-4.620770	10.312767
C	0.759956	-7.079370	8.511418
C	-0.935475	-7.124686	10.357327
C	1.158505	-5.769114	10.610707
C	0.565633	-7.064490	10.035600
H	-2.718188	-5.957701	9.968274
H	-1.889949	-6.839359	7.786314
H	-1.969230	-5.080402	7.756643
H	0.191258	-5.881383	6.799312
H	1.712545	-4.509488	8.221662
H	0.161685	-3.701924	8.013343
H	0.875145	-3.631872	10.402027
H	-1.203356	-4.586199	11.398189
H	-1.563481	-3.747518	9.892799
H	0.359489	-8.009520	8.090369
H	1.829117	-7.056308	8.268114
H	-1.087837	-7.135004	11.443456
H	-1.366639	-8.055034	9.968344
H	2.234648	-5.723768	10.404126
H	1.044475	-5.754537	11.701464
H	1.071456	-7.929453	10.478580
C	-2.013189	-10.936012	3.999398
C	-0.638296	-10.711631	3.351396
C	-0.160604	-9.278133	3.630406
C	-1.172634	-8.281364	3.045003
C	-2.549130	-8.499350	3.692032
C	-3.020544	-9.934961	3.413216
C	-0.055029	-9.063148	5.148110
C	-1.902411	-10.717110	5.516222

C	-2.437504	-8.286841	5.209633
C	-1.428675	-9.283513	5.800527
H	-2.351969	-11.958376	3.799121
H	0.086525	-11.433788	3.746181
H	-0.700243	-10.883534	2.269932
H	0.820023	-9.120569	3.167988
H	-0.832522	-7.253526	3.220301
H	-1.243307	-8.410721	1.958207
H	-3.269108	-7.788062	3.272807
H	-4.011920	-10.097724	3.853271
H	-3.124356	-10.093541	2.332914
H	0.680147	-9.754982	5.576732
H	0.303846	-8.048686	5.360027
H	-2.873761	-10.894537	5.993711
H	-1.199777	-11.438631	5.950355
H	-2.120015	-7.259014	5.423150
H	-3.418676	-8.419751	5.681440
H	-1.350018	-9.130156	6.882444
C	3.437536	7.569841	2.603542
C	2.988492	6.143649	2.250104
C	1.604951	5.868982	2.859467
C	1.682471	6.018961	4.386538
C	2.128259	7.444837	4.745783
C	3.510280	7.715629	4.131264
C	0.592528	6.880355	2.300080
C	2.420405	8.576632	2.044290
C	1.114837	8.452312	4.181242
C	1.035795	8.308488	2.653724
H	4.424076	7.763504	2.168406
H	2.951906	6.019285	1.161101
H	3.716980	5.416020	2.628013
H	1.286550	4.851860	2.605753
H	0.704803	5.805602	4.835425
H	2.387786	5.288747	4.801476
H	2.183352	7.549291	5.834866
H	3.849293	8.724284	4.397065
H	4.248128	7.015715	4.541971
H	0.511621	6.770369	1.211873
H	-0.403963	6.682442	2.713202
H	2.740485	9.600611	2.272354
H	2.372607	8.495876	0.951609
H	0.127220	8.282104	4.626731
H	1.411047	9.473978	4.448491
H	0.312429	9.027152	2.253082
C	-1.218150	8.136686	8.925708
C	-1.008209	8.159603	7.403873
C	-1.580703	6.876716	6.781455
C	-0.863868	5.656767	7.380054
C	-1.073215	5.627212	8.901963
C	-0.502387	6.913470	9.518924
C	-3.082776	6.786716	7.092049
C	-2.721300	8.043879	9.230459
C	-2.576563	5.540872	9.207291
C	-3.297759	6.760420	8.613300

H	-0.809150	9.051945	9.367443
H	-1.497267	9.039825	6.969403
H	0.060717	8.244744	7.173437
H	-1.430438	6.895460	5.696395
H	-1.250092	4.734823	6.928715
H	0.207381	5.698223	7.148621
H	-0.560879	4.757022	9.326616
H	-0.627729	6.896137	10.608399
H	0.575396	6.977036	9.325737
H	-3.610641	7.641658	6.652426
H	-3.507471	5.884072	6.636307
H	-2.886083	8.047416	10.314816
H	-3.241803	8.921442	8.828315
H	-2.992957	4.616738	8.788470
H	-2.738626	5.498552	10.291224
H	-4.369485	6.696865	8.831505
C	-6.356166	4.056641	8.963925
C	-7.858322	3.971260	9.275086
C	-8.442989	2.688440	8.663949
C	-8.234561	2.708877	7.141914
C	-6.733334	2.791105	6.824955
C	-6.152943	4.073453	7.441133
C	-7.724356	1.467727	9.259111
C	-5.643116	2.832471	9.558833
C	-6.018873	1.570432	7.425152
C	-6.221716	1.547031	8.947832
H	-5.941408	4.971900	9.400258
H	-8.019299	3.979045	10.359871
H	-8.376024	4.849893	8.871426
H	-9.513886	2.629359	8.887502
H	-8.665864	1.806836	6.691185
H	-8.759325	3.565213	6.701271
H	-6.587166	2.806102	5.739387
H	-5.084900	4.154438	7.205140
H	-6.640550	4.954048	7.005506
H	-7.882132	1.429021	10.343752
H	-8.146289	0.544112	8.844696
H	-4.565905	2.891826	9.361121
H	-5.764639	2.818846	10.648697
H	-6.410850	0.648314	6.979204
H	-4.948567	1.606722	7.188315
H	-5.712120	0.675815	9.373912
C	5.586453	4.850897	8.190873
C	5.140251	3.417970	7.861926
C	3.773026	3.139359	8.505375
C	3.884104	3.305293	10.028718
C	4.327186	4.737939	10.363547
C	5.692802	5.012612	9.715017
C	2.740558	4.137614	7.959219
C	4.549315	5.844537	7.645005
C	3.293631	5.732237	9.812302
C	3.180975	5.572442	8.288484
H	6.561360	5.047366	7.731453
H	5.079859	3.282366	6.775327

H	5.882602	2.699532	8.230320
H	3.456614	2.117431	8.269086
H	2.918527	3.089334	10.501877
H	4.604151	2.584475	10.434684
H	4.406230	4.853771	11.450002
H	6.030159	6.026347	9.963003
H	6.445048	4.322286	10.115711
H	2.635768	4.016071	6.874285
H	1.755234	3.936601	8.396900
H	4.866771	6.873076	7.855546
H	4.477303	5.752419	6.554552
H	2.317706	5.559318	10.281862
H	3.588132	6.758684	10.062581
H	2.443295	6.281724	7.897335
C	-3.478537	10.753500	3.389423
C	-4.979509	10.603582	3.681300
C	-5.514752	9.330512	3.007680
C	-5.294115	9.426330	1.490252
C	-3.793915	9.573330	1.192434
C	-3.262972	10.845557	1.871025
C	-4.759956	8.109242	3.555013
C	-2.729201	9.528601	3.936292
C	-3.043334	8.351732	1.744756
C	-3.258292	8.253115	3.262753
H	-3.099043	11.661707	3.870312
H	-5.149943	10.557187	4.763673
H	-5.523451	11.481490	3.312036
H	-6.584880	9.225342	3.217563
H	-5.690472	8.531408	0.995406
H	-5.844037	10.283400	1.082978
H	-3.639052	9.642006	0.110145
H	-2.196339	10.973059	1.649664
H	-3.776632	11.727876	1.470218
H	-4.925601	8.016545	4.635201
H	-5.146523	7.191465	3.095575
H	-1.652993	9.633098	3.752299
H	-2.859517	9.461933	5.023183
H	-3.399744	7.437877	1.254186
H	-1.972903	8.434724	1.520581
H	-2.722896	7.381331	3.654663
C	-8.956364	5.458920	1.224805
C	-8.407419	6.740198	1.871002
C	-8.610973	6.682382	3.392901
C	-10.110579	6.553958	3.701343
C	-10.664233	5.272221	3.059841
C	-10.455070	5.333451	1.538839
C	-7.869079	5.462060	3.959646
C	-8.213067	4.241950	1.797027
C	-9.916869	4.055458	3.627002
C	-8.416819	4.177542	3.318471
H	-8.810476	5.502275	0.139934
H	-7.341841	6.853090	1.637484
H	-8.917581	7.618191	1.456496
H	-8.218378	7.596588	3.851576

H	-10.270800	6.531822	4.786099
H	-10.650093	7.429009	3.319157
H	-11.733519	5.183025	3.280565
H	-10.864207	4.432303	1.066076
H	-11.001022	6.186679	1.118226
H	-6.793467	5.550580	3.764560
H	-7.990882	5.420515	5.048807
H	-8.581853	3.321485	1.328265
H	-7.143743	4.309822	1.563149
H	-10.073918	3.989044	4.710396
H	-10.316914	3.131622	3.191736
H	-7.885564	3.309477	3.724035
C	10.199402	-1.064322	3.563702
C	9.717502	-2.487112	3.241173
C	8.340140	-2.725799	3.879026
C	8.447661	-2.553285	5.401894
C	8.926404	-1.130658	5.730290
C	10.302082	-0.895897	5.087371
C	7.337024	-1.704302	3.321270
C	9.191496	-0.047315	3.006237
C	7.922125	-0.113121	5.167462
C	7.813190	-0.279460	3.644074
H	11.181483	-0.896324	3.108286
H	9.659189	-2.627897	2.155120
H	10.438856	-3.222413	3.617914
H	7.998277	-3.740556	3.647325
H	7.474325	-2.740925	5.871218
H	9.146463	-3.290146	5.816101
H	9.002846	-1.010128	6.816419
H	10.664607	0.110194	5.330931
H	11.033896	-1.603153	5.496290
H	7.234689	-1.829858	2.236558
H	6.344524	-1.876707	3.754920
H	9.534709	0.973861	3.212156
H	9.122726	-0.144349	1.916001
H	6.939591	-0.257487	5.632868
H	8.242131	0.906814	5.413010
H	7.096383	0.446424	3.244661
C	8.636222	6.367404	4.247815
C	7.154271	6.263059	4.639629
C	6.554987	4.970398	4.064443
C	6.680946	4.988258	2.533345
C	8.161700	5.089419	2.135800
C	8.756992	6.381568	2.716250
C	7.321044	3.761499	4.623469
C	9.396737	5.154973	4.806897
C	8.923861	3.880520	2.700078
C	8.803554	3.859774	4.231529
H	9.061428	7.289654	4.658626
H	7.051787	6.272513	5.731480
H	6.603765	7.133270	4.261968
H	5.498563	4.897803	4.345487
H	6.238267	4.078954	2.109013
H	6.121701	5.835950	2.119196

H	8.249047	5.102543	1.043900
H	9.809601	6.475983	2.422878
H	8.234789	7.254031	2.305043
H	7.222378	3.724508	5.715134
H	6.889965	2.830875	4.234954
H	10.460852	5.228017	4.551317
H	9.334210	5.143563	5.901757
H	8.520976	2.951683	2.278458
H	9.979290	3.930272	2.405866
H	9.347072	2.996949	4.631993
C	3.521595	-1.821470	9.670734
C	3.721835	-1.827011	8.147429
C	5.221994	-1.765510	7.820786
C	5.819391	-0.479519	8.412218
C	5.624589	-0.470206	9.936315
C	4.123426	-0.535428	10.257567
C	5.927346	-2.983723	8.436600
C	4.231083	-3.039914	10.281444
C	6.328468	-1.691921	10.546808
C	5.732467	-2.980952	9.960780
H	2.451551	-1.865130	9.901405
H	3.278355	-2.730847	7.712529
H	3.203643	-0.971505	7.697387
H	5.362422	-1.770138	6.734257
H	6.886703	-0.414011	8.168474
H	5.338021	0.399270	7.966273
H	6.050728	0.447702	10.355718
H	3.969373	-0.509442	11.343181
H	3.612010	0.342599	9.844651
H	5.524634	-3.909315	8.007719
H	6.996658	-2.961422	8.193889
H	4.078270	-3.058824	11.367394
H	3.797661	-3.965891	9.884616
H	7.404806	-1.647659	10.341005
H	6.214050	-1.686076	11.637605
H	6.235874	-3.850861	10.396773
C	-4.777794	-3.379253	8.245692
C	-5.774864	-2.381899	7.636003
C	-7.164857	-2.592733	8.256019
C	-7.636137	-4.027397	7.973030
C	-6.643903	-5.029278	8.583451
C	-5.255150	-4.811956	7.962902
C	-7.083112	-2.374766	9.774768
C	-4.702094	-3.158709	9.764434
C	-6.563040	-4.804896	10.101294
C	-6.089136	-3.372249	10.389710
H	-3.787658	-3.227416	7.802199
H	-5.435417	-1.354177	7.812911
H	-5.823755	-2.516904	6.548650
H	-7.873960	-1.880262	7.820306
H	-8.636755	-4.184351	8.393727
H	-7.717914	-4.190657	6.891589
H	-6.981590	-6.051349	8.380011
H	-4.541035	-5.534704	8.375990

H	-5.294616	-4.989502	6.881284
H	-6.767383	-1.347122	9.991790
H	-8.073959	-2.503663	10.226918
H	-3.977567	-3.851577	10.209397
H	-4.344159	-2.144762	9.980193
H	-7.544642	-4.975871	10.559599
H	-5.872605	-5.527946	10.552364
H	-6.032610	-3.213955	11.472298
C	-11.209960	-4.734740	-1.556277
C	-10.680635	-3.424221	-0.953833
C	-9.175358	-3.294887	-1.233774
C	-8.436238	-4.484986	-0.603048
C	-8.959703	-5.798482	-1.204396
C	-10.465609	-5.921233	-0.924888
C	-8.938048	-3.295769	-2.751823
C	-10.967655	-4.731416	-3.073585
C	-8.723429	-5.792910	-2.722578
C	-9.463167	-4.606146	-3.358680
H	-12.283086	-4.825077	-1.355403
H	-11.218110	-2.569105	-1.381183
H	-10.864936	-3.406418	0.127217
H	-8.800231	-2.359604	-0.803876
H	-7.357543	-4.393694	-0.778946
H	-8.581105	-4.485143	0.484090
H	-8.431775	-6.645534	-0.752905
H	-10.848300	-6.864989	-1.332122
H	-10.646450	-5.947240	0.156592
H	-9.443552	-2.438683	-3.213021
H	-7.868019	-3.184502	-2.965110
H	-11.359976	-5.653705	-3.519078
H	-11.509451	-3.899458	-3.539483
H	-7.649787	-5.725012	-2.935888
H	-9.074646	-6.734660	-3.161574
H	-9.293126	-4.603492	-4.440959
C	-4.150982	-8.248610	-1.627881
C	-2.769760	-8.011104	-2.257484
C	-2.309374	-6.573234	-1.971951
C	-3.322878	-5.585908	-2.570654
C	-4.705752	-5.817035	-1.942045
C	-5.159843	-7.256957	-2.227300
C	-2.226054	-6.357648	-0.452948
C	-4.062485	-8.029058	-0.109688
C	-4.616357	-5.603867	-0.423065
C	-3.606127	-6.591128	0.181086
H	-4.477403	-9.274073	-1.832784
H	-2.043479	-8.726491	-1.853172
H	-2.815674	-8.183308	-3.339699
H	-1.324206	-6.406308	-2.421246
H	-2.994846	-4.554952	-2.390710
H	-3.377848	-5.715649	-3.658312
H	-5.426762	-5.112464	-2.370732
H	-6.155411	-7.429178	-1.800509
H	-5.247757	-7.416235	-3.308910
H	-1.490156	-7.042625	-0.014661

H	-1.879623	-5.339899	-0.236139
H	-5.038391	-8.215762	0.354798
H	-3.358918	-8.744028	0.333672
H	-4.311453	-4.573145	-0.205206
H	-5.602428	-5.746148	0.035614
H	-3.543336	-6.437311	1.263974
C	-1.094254	-1.398845	-7.321940
C	-2.136538	-0.434066	-7.907968
C	-1.735660	1.015865	-7.595001
C	-1.659745	1.205597	-6.072277
C	-0.616283	0.245011	-5.481204
C	-1.019797	-1.202934	-5.799917
C	-0.359616	1.308180	-8.212937
C	0.279962	-1.100341	-7.941112
C	0.756859	0.538874	-6.104791
C	0.687096	0.347544	-7.627543
H	-1.382025	-2.431857	-7.545960
H	-2.214833	-0.577549	-8.992410
H	-3.126256	-0.649687	-7.487352
H	-2.479835	1.701975	-8.014218
H	-1.393704	2.242888	-5.835405
H	-2.641321	1.019052	-5.619990
H	-0.564264	0.381640	-4.395536
H	-0.292949	-1.900392	-5.366085
H	-1.989898	-1.432327	-5.342465
H	-0.404378	1.196555	-9.303062
H	-0.070624	2.347038	-8.012709
H	1.029731	-1.796500	-7.545884
H	0.245463	-1.254874	-9.026325
H	1.065694	1.564291	-5.868238
H	1.515311	-0.127273	-5.675882
H	1.666792	0.558002	-8.070436
C	1.019643	-4.099226	-1.674203
C	2.400039	-3.841395	-2.297590
C	2.847377	-2.403562	-1.991848
C	1.826588	-1.417083	-2.579468
C	0.444487	-1.668536	-1.956992
C	0.003461	-3.108371	-2.262469
C	2.925728	-2.207641	-0.469920
C	1.103138	-3.899284	-0.153019
C	0.528934	-1.474981	-0.435105
C	1.546438	-2.461496	0.157956
H	0.702530	-5.124630	-1.893514
H	3.131659	-4.555844	-1.901324
H	2.357826	-3.999471	-3.382110
H	3.831978	-2.222136	-2.436744
H	2.145316	-0.385839	-2.385036
H	1.774963	-1.532706	-3.668881
H	-0.281719	-0.964531	-2.377764
H	-0.991448	-3.294880	-1.840170
H	-0.080864	-3.253893	-3.346301
H	3.666633	-2.892055	-0.039253
H	3.262894	-1.189938	-0.238763
H	0.127939	-4.100610	0.306821

H	1.811962	-4.614035	0.282249
H	0.824459	-0.444678	-0.202815
H	-0.456802	-1.631899	0.019501
H	1.605685	-2.321669	1.242939
C	8.022310	-3.609578	-0.308554
C	6.522271	-3.711036	0.007952
C	5.942237	-4.983447	-0.628823
C	6.142902	-4.927797	-2.151020
C	7.642001	-4.829366	-2.473388
C	8.217787	-3.557565	-1.831564
C	6.671142	-6.212726	-0.064587
C	8.745651	-4.842341	0.255377
C	8.366759	-6.058744	-1.904098
C	8.171701	-6.117338	-0.381349
H	8.433763	-2.701766	0.146083
H	6.366694	-3.728430	1.093412
H	5.997299	-2.826791	-0.373452
H	4.872848	-5.054029	-0.401420
H	5.714752	-5.822173	-2.619673
H	5.610816	-4.065061	-2.569861
H	7.782629	-4.789270	-3.559051
H	9.284128	-3.464826	-2.070900
H	7.722737	-2.670463	-2.245029
H	6.519044	-6.276576	1.019675
H	6.252674	-7.129424	-0.497463
H	9.821482	-4.772023	0.053785
H	8.629681	-4.881012	1.345246
H	7.978078	-6.973059	-2.368612
H	9.435628	-6.010654	-2.145287
H	8.688635	-6.994717	0.022674
C	-6.195646	3.531237	-6.935340
C	-5.398944	2.348458	-6.363603
C	-5.919717	1.033267	-6.963621
C	-7.406481	0.867613	-6.613564
C	-8.208639	2.046596	-7.185795
C	-7.681756	3.359233	-6.585525
C	-5.758321	1.071598	-8.491127
C	-6.032977	3.563120	-8.462909
C	-8.041113	2.082737	-8.712675
C	-6.556024	2.251388	-9.068082
H	-5.822984	4.467744	-6.506167
H	-4.332356	2.468874	-6.588596
H	-5.491935	2.326098	-5.270989
H	-5.349924	0.191432	-6.555044
H	-7.785279	-0.078144	-7.019661
H	-7.534140	0.818919	-5.525360
H	-9.268144	1.926677	-6.934563
H	-8.259540	4.208044	-6.971078
H	-7.814643	3.354888	-5.496810
H	-4.698510	1.168910	-8.756019
H	-6.109096	0.129679	-8.929965
H	-6.580845	4.416022	-8.881713
H	-4.977791	3.704555	-8.726230
H	-8.431389	1.158563	-9.155851

H	-8.625790	2.908523	-9.135988
H	-6.438479	2.276401	-10.157030
C	8.011684	1.754631	-2.048904
C	7.597259	0.310727	-2.371357
C	6.227376	0.011202	-1.743137
C	6.315488	0.191801	-0.219960
C	6.726722	1.635413	0.108336
C	8.095139	1.930927	-0.524952
C	5.183188	0.984888	-2.310939
C	6.962912	2.723614	-2.616439
C	5.681590	2.605078	-0.464536
C	5.591789	2.430583	-1.988259
H	8.988536	1.966008	-2.497446
H	7.553574	0.165028	-3.457448
H	8.348365	-0.390245	-1.987389
H	5.933648	-1.018534	-1.974770
H	5.348158	-0.038769	0.242605
H	7.043854	-0.511670	0.201309
H	6.789435	1.761700	1.194691
H	8.409811	2.952970	-0.281284
H	8.855180	1.258517	-0.108800
H	5.094827	0.852407	-3.396055
H	4.196292	0.768587	-1.884228
H	7.257892	3.759767	-2.410594
H	6.906828	2.621137	-3.706903
H	4.703138	2.417310	-0.006067
H	5.953145	3.639026	-0.219232
H	4.845823	3.122303	-2.394840
C	-6.790883	-8.455861	4.423951
C	-8.297024	-8.568842	4.705657
C	-9.033935	-7.377039	4.075165
C	-8.495957	-6.069804	4.676965
C	-6.990198	-5.950170	4.395386
C	-6.258550	-7.145826	5.024707
C	-8.793359	-7.374894	2.557631
C	-6.556378	-8.451405	2.905421
C	-6.754658	-5.952123	2.877081
C	-7.288255	-7.258957	2.270693
H	-6.267433	-9.306257	4.874435
H	-8.686576	-9.510180	4.299621
H	-8.476073	-8.592009	5.787488
H	-10.107607	-7.459946	4.276763
H	-9.028516	-5.211771	4.249161
H	-8.679065	-6.049407	5.758126
H	-6.608736	-5.017806	4.825773
H	-5.179341	-7.062211	4.847837
H	-6.401674	-7.144304	6.112131
H	-9.191489	-8.294250	2.111409
H	-9.330507	-6.539158	2.093034
H	-5.482397	-8.391770	2.690934
H	-6.915215	-9.390138	2.466359
H	-7.256224	-5.091846	2.417546
H	-5.684307	-5.846694	2.662144
H	-7.119948	-7.258318	1.188140

C	-5.763313	4.426061	-2.740743
C	-6.775544	5.406068	-3.353572
C	-6.356131	6.850265	-3.038389
C	-6.309908	7.045968	-1.515230
C	-5.296535	6.070157	-0.897350
C	-5.718423	4.627964	-1.218344
C	-4.962097	7.115522	-3.627540
C	-4.370972	4.697533	-3.331197
C	-3.905253	6.336985	-1.492221
C	-3.945354	6.139578	-3.015267
H	-6.064265	3.397168	-2.966388
H	-6.832831	5.258758	-4.438814
H	-7.777779	5.209794	-2.953630
H	-7.078880	7.547243	-3.476729
H	-6.030880	8.079580	-1.277228
H	-7.304179	6.878775	-1.083576
H	-5.265661	6.211079	0.188576
H	-5.013512	3.920012	-0.765720
H	-6.702089	4.417755	-0.781088
H	-4.985182	6.999451	-4.717875
H	-4.659306	8.150125	-3.425680
H	-3.642288	3.990293	-2.916674
H	-4.384657	4.538405	-4.416208
H	-3.583649	7.357978	-1.253554
H	-3.168092	5.659763	-1.043970
H	-2.952745	6.330757	-3.437676
C	-6.257306	-5.545124	-7.287305
C	-4.864669	-5.283088	-7.881007
C	-4.433497	-3.840121	-7.576226
C	-5.446774	-2.864736	-8.194562
C	-6.841134	-3.120412	-7.601860
C	-7.265990	-4.565338	-7.906218
C	-4.391196	-3.632200	-6.054460
C	-6.209870	-5.333154	-5.766198
C	-6.792724	-2.914817	-6.079979
C	-5.782855	-3.890218	-5.456277
H	-6.562861	-6.574186	-7.505887
H	-4.137816	-5.989773	-7.462660
H	-4.881080	-5.449626	-8.964957
H	-3.440143	-3.655697	-7.999928
H	-5.139072	-1.830011	-8.000860
H	-5.472751	-2.988922	-9.283947
H	-7.561949	-2.424320	-8.044501
H	-8.269100	-4.755081	-7.505301
H	-7.324596	-4.719590	-8.990538
H	-3.656037	-4.308532	-5.601743
H	-4.065816	-2.610619	-5.823520
H	-7.194040	-5.537302	-5.327188
H	-5.506698	-6.039994	-5.309379
H	-6.509077	-1.880898	-5.848952
H	-7.787588	-3.074667	-5.646784
H	-5.749309	-3.741814	-4.371335
C	5.019416	-5.167264	3.604290
C	4.550626	-6.600782	3.311296

C	3.171600	-6.836113	3.946795
C	3.269111	-6.628498	5.465952
C	3.734711	-5.195036	5.764797
C	5.112113	-4.963758	5.124313
C	2.163613	-5.835528	3.360635
C	4.006673	-4.171199	3.018496
C	2.725622	-4.198533	5.173650
C	2.626650	-4.399997	3.653827
H	6.002705	-5.001672	3.150602
H	4.499592	-6.766446	2.228392
H	5.275627	-7.321592	3.708485
H	2.839097	-7.858544	3.736169
H	2.294643	-6.813342	5.934035
H	3.971360	-7.350206	5.900508
H	3.804034	-5.049473	6.848335
H	5.465279	-3.949557	5.347104
H	5.847170	-5.655723	5.553097
H	2.068430	-5.986291	2.278486
H	1.170064	-6.006099	3.792606
H	4.340621	-3.142921	3.203210
H	3.944860	-4.293304	1.930366
H	1.741636	-4.340279	5.636790
H	3.036149	-3.170789	5.397882
H	1.906400	-3.689076	3.234209
C	2.885331	-7.721820	-0.225560
C	1.380152	-7.837423	0.060194
C	0.830174	-9.127614	-0.567408
C	1.063220	-9.094607	-2.085647
C	2.567626	-8.982139	-2.377230
C	3.113255	-7.692530	-1.744661
C	1.562026	-10.337836	0.033046
C	3.611673	-8.935603	0.374528
C	3.295224	-10.192430	-1.771812
C	3.067808	-10.228298	-0.252851
H	3.275335	-6.801349	0.222481
H	1.201207	-7.838837	1.142187
H	0.852527	-6.966490	-0.347285
H	-0.242941	-9.208252	-0.361959
H	0.656661	-10.002094	-2.548285
H	0.529569	-8.245890	-2.530340
H	2.731345	-8.958225	-3.460130
H	4.183304	-7.589977	-1.962479
H	2.616205	-6.818884	-2.183593
H	1.387170	-10.385670	1.114699
H	1.164648	-11.266922	-0.393244
H	4.690669	-8.854718	0.195022
H	3.472494	-8.957703	1.462140
H	2.928282	-11.119272	-2.229125
H	4.368401	-10.134515	-1.990675
H	3.586818	-11.092097	0.176951
C	0.405587	0.738693	8.218697
C	-0.616953	1.732349	7.646295
C	-1.992417	1.488235	8.286186
C	-2.447126	0.052306	7.983207

C	-1.429340	-0.945963	8.556335
C	-0.055222	-0.695372	7.916061
C	-1.887006	1.677821	9.807283
C	0.504915	0.930763	9.739943
C	-1.324924	-0.749960	10.076669
C	-0.867465	0.683886	10.384987
H	1.385316	0.914272	7.761079
H	-0.289835	2.761507	7.837500
H	-0.683105	1.617805	6.557508
H	-2.719719	2.198115	7.877055
H	-3.437662	-0.128105	8.418119
H	-2.545627	-0.091103	6.900347
H	-1.755254	-1.968929	8.338689
H	0.676842	-1.415010	8.302285
H	-0.111213	-0.852412	6.832003
H	-1.582887	2.705758	10.039000
H	-2.867636	1.524996	10.274020
H	1.247508	0.240536	10.158439
H	0.851412	1.945678	9.969390
H	-2.295572	-0.944828	10.548566
H	-0.615854	-1.471001	10.501268
H	-0.794113	0.821923	11.469337
C	-6.952939	-0.668383	2.617279
C	-7.396658	-2.111444	2.332197
C	-8.781832	-2.360103	2.948974
C	-8.710752	-2.135525	4.467183
C	-8.270311	-0.692505	4.758067
C	-6.886626	-0.447973	4.136324
C	-9.794819	-1.380227	2.337017
C	-7.970622	0.306908	2.005663
C	-9.284266	0.283222	4.141150
C	-9.356883	0.064765	2.622187
H	-5.965226	-0.493293	2.176964
H	-7.428604	-2.288932	1.250435
H	-6.667668	-2.817610	2.747957
H	-9.096429	-3.389331	2.744019
H	-9.689593	-2.329589	4.922228
H	-8.005073	-2.842532	4.919965
H	-8.219804	-0.534846	5.840953
H	-6.551441	0.573449	4.353844
H	-6.148481	-1.124828	4.583525
H	-9.871128	-1.543446	1.255179
H	-10.792384	-1.560666	2.755513
H	-7.654264	1.341737	2.184670
H	-8.013885	0.172764	0.918054
H	-10.273161	0.132062	4.590655
H	-8.991925	1.317584	4.359317
H	-10.080637	0.760882	2.184179
C	-10.939879	0.309577	-2.682008
C	-9.553811	0.568868	-3.292065
C	-9.126163	2.017884	-3.012027
C	-10.149205	2.979972	-3.635100
C	-11.537031	2.727019	-3.026118
C	-11.958351	1.276076	-3.305762

C	-9.071015	2.247930	-1.493860
C	-10.879621	0.543683	-1.164605
C	-11.475763	2.954766	-1.507858
C	-10.456082	1.992725	-0.879390
H	-11.242932	-0.723808	-2.882962
H	-8.820152	-0.128642	-2.870216
H	-9.579294	0.386630	-4.373311
H	-8.137487	2.200346	-3.447359
H	-9.844253	4.018670	-3.459123
H	-10.184484	2.839964	-4.722302
H	-12.264826	3.413611	-3.472160
H	-12.956966	1.087925	-2.893046
H	-12.026083	1.105941	-4.387171
H	-8.328872	1.581301	-1.038158
H	-8.747994	3.274097	-1.280622
H	-11.858889	0.341758	-0.713766
H	-10.169291	-0.153525	-0.704072
H	-11.194526	3.993096	-1.294332
H	-12.465970	2.797005	-1.063374
H	-10.413378	2.156917	0.202954
C	-3.960238	0.830270	-1.266234
C	-5.459027	0.714142	-0.948855
C	-6.019387	-0.581380	-1.555956
C	-5.817580	-0.558708	-3.078850
C	-4.319678	-0.445759	-3.402020
C	-3.763562	0.849209	-2.789860
C	-5.273156	-1.786089	-0.962149
C	-3.219517	-0.378012	-0.672656
C	-3.577598	-1.650508	-2.803163
C	-3.773757	-1.676027	-1.279641
H	-3.562820	1.754542	-0.832771
H	-5.615746	0.720071	0.136565
H	-5.996494	1.581281	-1.351536
H	-7.087914	-0.662379	-1.327972
H	-6.231872	-1.470128	-3.526659
H	-6.361794	0.285941	-3.518457
H	-4.178241	-0.429220	-4.488192
H	-2.698395	0.952031	-3.030300
H	-4.271111	1.718890	-3.224634
H	-5.425709	-1.826574	0.123173
H	-5.677484	-2.718835	-1.373655
H	-2.144579	-0.296572	-0.874824
H	-3.336326	-0.392662	0.417711
H	-3.952137	-2.581178	-3.246325
H	-2.509242	-1.592326	-3.044398
H	-3.244458	-2.535888	-0.854516
C	0.820793	3.693794	-3.422819
C	1.379144	4.992845	-2.821742
C	1.203791	4.975945	-1.295334
C	-0.289629	4.853837	-0.955874
C	-0.852600	3.554447	-1.552160
C	-0.671629	3.574791	-3.077867
C	1.958305	3.772433	-0.709623
C	1.576806	2.493782	-2.831750

C	-0.092616	2.354512	-0.966231
C	1.401259	2.470280	-1.305616
H	0.946574	3.707982	-4.510977
H	2.440021	5.100853	-3.077878
H	0.859773	5.858594	-3.250327
H	1.603052	5.902770	-0.868868
H	-0.429782	4.860837	0.131860
H	-0.837745	5.717479	-1.351534
H	-1.917465	3.469775	-1.309384
H	-1.087714	2.660616	-3.518461
H	-1.226849	4.415571	-3.511225
H	3.029969	3.857131	-0.926874
H	1.856681	3.760203	0.382328
H	1.201175	1.560461	-3.268570
H	2.641506	2.556782	-3.087099
H	-0.229539	2.317217	0.121280
H	-0.498875	1.418684	-1.368902
H	1.941535	1.614243	-0.886627
C	-1.763611	3.422812	2.590640
C	-2.207210	1.983372	2.287627
C	-3.588565	1.724221	2.908635
C	-3.509146	1.927341	4.429452
C	-3.068792	3.366652	4.738284
C	-1.688949	3.621745	4.112260
C	-4.606180	2.711380	2.316378
C	-2.785914	4.405376	1.998685
C	-4.087397	4.349732	4.141013
C	-4.168366	4.152742	2.619539
H	-0.778622	3.605391	2.147276
H	-2.245077	1.821201	1.203654
H	-1.475028	1.272315	2.689199
H	-3.903085	0.697602	2.690882
H	-4.485157	1.725592	4.887224
H	-2.800063	1.214886	4.868161
H	-3.012334	3.509005	5.822994
H	-1.353761	4.640408	4.342350
H	-0.947469	2.939561	4.545633
H	-4.688427	2.563421	1.232782
H	-5.601138	2.523749	2.737905
H	-2.469786	5.437966	2.190563
H	-2.835183	4.286612	0.909544
H	-5.073545	4.190948	4.593912
H	-3.795064	5.381269	4.372176
H	-4.895426	4.854074	2.195549
C	-2.138300	-3.130372	2.232029
C	-1.577888	-1.837908	2.845263
C	-1.778231	-1.856665	4.368570
C	-3.278043	-1.965744	4.683392
C	-3.843156	-3.258516	4.074873
C	-3.637175	-3.236368	2.552366
C	-1.044463	-3.068427	4.963555
C	-1.403089	-4.338661	2.832403
C	-3.103889	-4.466753	4.670173
C	-1.603682	-4.364018	4.355418

H	-1.994684	-3.114867	1.146099
H	-0.512030	-1.739122	2.606684
H	-2.082221	-0.966423	2.410390
H	-1.377461	-0.934543	4.803726
H	-3.435827	-1.959928	5.768716
H	-3.811672	-1.096176	4.280966
H	-4.912569	-3.333900	4.300087
H	-4.054421	-4.145654	2.102842
H	-4.177504	-2.389510	2.112076
H	0.031332	-2.993138	4.763973
H	-1.163970	-3.082206	6.053675
H	-1.780125	-5.267474	2.387265
H	-0.333836	-4.284918	2.594563
H	-3.258848	-4.505259	5.755218
H	-3.512128	-5.397868	4.258673
H	-1.078202	-5.225962	4.781061
C	3.772710	-2.314769	-6.214702
C	4.366465	-1.045617	-5.584390
C	5.867861	-0.962601	-5.900417
C	6.577468	-2.199647	-5.328906
C	5.989483	-3.472025	-5.958377
C	4.487696	-3.548429	-5.642553
C	6.065874	-0.921327	-7.423531
C	3.975891	-2.269503	-7.737073
C	6.186702	-3.424261	-7.481386
C	5.476212	-2.190344	-8.058360
H	2.702455	-2.372132	-5.988043
H	3.850866	-0.157400	-5.969044
H	4.210812	-1.057292	-4.498762
H	6.288971	-0.056742	-5.450560
H	7.653964	-2.142373	-5.530731
H	6.460687	-2.230918	-4.238842
H	6.496469	-4.352646	-5.549086
H	4.059292	-4.463231	-6.069839
H	4.333857	-3.604000	-4.558014
H	5.581418	-0.031185	-7.842799
H	7.133507	-0.842530	-7.661827
H	3.537694	-3.161226	-8.201626
H	3.454110	-1.402962	-8.160878
H	7.256305	-3.388912	-7.721215
H	5.788998	-4.337157	-7.941189
H	5.618360	-2.157670	-9.144167
C	0.722601	-5.042450	-5.810562
C	-0.783112	-5.146261	-5.523103
C	-1.342758	-6.434970	-6.145179
C	-1.110791	-6.409564	-7.663729
C	0.394122	-6.308906	-7.957005
C	0.949419	-5.020728	-7.329957
C	-0.618981	-7.647988	-5.540591
C	1.440842	-6.258990	-5.206322
C	1.113649	-7.521942	-7.347449
C	0.887292	-7.550246	-5.828170
H	1.119503	-4.123018	-5.366462
H	-0.961131	-5.142168	-4.440964

```
H     -1.304904     -4.273213     -5.933557
H     -2.416239     -6.507194     -5.938521
H     -1.524167     -7.315952     -8.122464
H     -1.638798     -5.558835     -8.111305
H      0.557074     -6.290395     -9.040127
H      2.019980     -4.926617     -7.549066
H      0.458193     -4.145307     -7.771899
H     -0.793240     -7.690343     -4.458614
H     -1.023298     -8.575896     -5.962901
H      2.520229     -6.186459     -5.387043
H      1.302447     -6.275842     -4.118516
H      0.739756     -8.447944     -7.800817
H      2.187021     -7.472494     -7.567432
H      1.400540     -8.416012     -5.395415
C      3.406919      2.243477      4.334356
C      1.908084      2.139194      4.655611
C      1.335870      0.848297      4.049716
C      1.533890      0.869695      2.526306
C      3.031797      0.970808      2.199262
C      3.599776      2.261183      2.810227
C      2.074045     -0.362432      4.641343
C      4.139554      1.029219      4.925768
C      3.765834     -0.239930      2.795951
C      3.573441     -0.264219      4.319973
H      3.812794      3.164468      4.766965
H      1.754239      2.146134      5.741435
H      1.376480      3.010663      4.254498
H      0.267330      0.775742      4.280463
H      1.111206     -0.038307      2.079398
H      0.995265      1.718737      2.088282
H      3.170537      0.986457      1.112729
H      4.665099      2.355589      2.567036
H      3.098026      3.134964      2.376946
H      1.923997     -0.401939      5.727050
H      1.661249     -1.291846      4.230708
H      5.214577      1.102158      4.720820
H      4.025468      1.015262      6.016432
H      3.382761     -1.167498      2.353583
H      4.833988     -0.190187      2.551948
H      4.096994     -1.128351      4.743551
```

Adamantane$_{36}$

```
         936
E=-1736.4710
C      5.873592      4.432810     -7.683929
C      5.445027      2.992403     -8.003529
C      4.087594      2.696865     -7.347033
C      4.209603      2.870303     -5.825363
C      4.635102      4.310407     -5.499853
C      5.990940      4.601964     -6.161400
C      3.036250      3.678182     -7.887852
C      4.817647      5.409446     -8.224401
C      3.582684      5.287731     -6.045737
```

C	3.459006	5.120407	-7.567890
H	6.841543	4.641346	-8.152643
H	5.377083	2.851717	-9.089034
H	6.200747	2.286049	-7.638968
H	3.783746	1.669621	-7.576671
H	3.251378	2.642464	-5.342918
H	4.943384	2.161398	-5.423027
H	4.721978	4.431588	-4.414581
H	6.315949	5.621359	-5.920219
H	6.756434	3.923987	-5.764735
H	2.923728	3.550929	-8.971356
H	2.057759	3.464868	-7.440766
H	5.122220	6.443213	-8.020544
H	4.737430	5.312060	-9.313823
H	2.613468	5.102767	-5.566962
H	3.864683	6.319235	-5.801932
H	2.707888	5.817584	-7.955211
C	-1.650477	-5.911835	9.733757
C	-1.450775	-5.929307	8.210472
C	0.049088	-5.866315	7.882757
C	0.643119	-4.573864	8.463398
C	0.448844	-4.552599	9.987443
C	-1.052014	-4.619358	10.309797
C	0.758051	-7.077474	8.508263
C	-0.937380	-7.123255	10.354160
C	1.156342	-5.767307	10.607657
C	0.563718	-7.062751	10.032445
H	-2.720311	-5.956578	9.965191
H	-1.891896	-6.837907	7.783166
H	-1.971510	-5.078962	7.753632
H	0.189134	-5.879460	6.796249
H	1.710156	-4.507389	8.218714
H	0.159143	-3.700101	8.010450
H	0.872580	-3.630103	10.399143
H	-1.205745	-4.584901	11.395221
H	-1.566022	-3.746170	9.889895
H	0.357761	-8.007666	8.087139
H	1.827208	-7.054191	8.264965
H	-1.089745	-7.133687	11.440288
H	-1.368367	-8.053655	9.965103
H	2.232478	-5.721742	10.401084
H	1.042304	-5.752838	11.698414
H	1.069702	-7.927653	10.475359
C	-2.014265	-10.934436	3.996786
C	-0.639022	-10.710106	3.349508
C	-0.161511	-9.276565	3.628606
C	-1.173241	-8.279887	3.042530
C	-2.550086	-8.497822	3.688833
C	-3.021319	-9.933476	3.409933
C	-0.056770	-9.061394	5.146341
C	-1.904320	-10.715348	5.513644
C	-2.439294	-8.285127	5.206469
C	-1.430768	-9.281707	5.798034
H	-2.352915	-11.956831	3.796448

H	0.085597	-11.432201	3.744776
H	-0.700375	-10.882141	2.268031
H	0.819365	-9.119037	3.166705
H	-0.833246	-7.252021	3.217888
H	-1.243318	-8.409376	1.955710
H	-3.269849	-7.786600	3.269128
H	-4.012932	-10.096206	3.849466
H	-3.124537	-10.092189	2.329593
H	0.678186	-9.753162	5.575449
H	0.301969	-8.046899	5.358332
H	-2.875927	-10.892737	5.990624
H	-1.201908	-11.436803	5.948248
H	-2.121942	-7.257268	5.420034
H	-3.420721	-8.418000	5.677755
H	-1.352704	-9.128218	6.879975
C	3.432012	7.570181	2.601000
C	2.983578	6.143334	2.249437
C	1.600127	5.868897	2.859111
C	1.677526	6.020933	4.385984
C	2.122704	7.447471	4.743355
C	3.504636	7.718025	4.128529
C	0.587303	6.879104	2.298344
C	2.414481	8.575804	2.040374
C	1.108883	8.453772	4.177440
C	1.029960	8.307889	2.650110
H	4.418487	7.763679	2.165645
H	2.947085	6.017510	1.160599
H	3.712354	5.416511	2.628338
H	1.282161	4.851306	2.606734
H	0.699929	5.807762	4.835117
H	2.383130	5.291566	4.801917
H	2.177711	7.553392	5.832301
H	3.843217	8.727174	4.393005
H	4.242760	7.018965	4.540193
H	0.506485	6.767641	1.210281
H	-0.409121	6.681322	2.711690
H	2.734124	9.600218	2.267092
H	2.366759	8.493578	0.947799
H	0.121320	8.283743	4.623116
H	1.404656	9.475916	4.443344
H	0.306309	9.025719	2.248488
C	-1.221723	8.136344	8.926558
C	-1.011917	8.160959	7.404730
C	-1.584254	6.878658	6.780960
C	-0.867166	5.658175	7.378164
C	-1.076377	5.626922	8.900058
C	-0.505707	6.912599	9.518375
C	-3.086285	6.788072	7.091585
C	-2.724832	8.042957	9.231336
C	-2.579684	5.540001	9.205421
C	-3.301132	6.760078	8.612825
H	-0.812836	9.051188	9.369257
H	-1.501157	9.041575	6.971265
H	0.056975	8.246529	7.174295

H	-1.434085	6.898614	5.695909
H	-1.253276	4.736661	6.925851
H	0.204057	5.700061	7.146684
H	-0.563860	4.756354	9.323716
H	-0.630952	6.894055	10.607841
H	0.572049	6.976554	9.325165
H	-3.614329	7.643407	6.652943
H	-3.510871	5.885857	6.634893
H	-2.889522	8.045282	10.315711
H	-3.245513	8.920873	8.830197
H	-2.995961	4.616257	8.785626
H	-2.741646	5.496469	10.289320
H	-4.372829	6.696108	8.831052
C	-6.359325	4.056122	8.962011
C	-7.861690	3.971089	9.272255
C	-8.446199	2.688203	8.661103
C	-8.236814	2.708186	7.139195
C	-6.735374	2.790066	6.823153
C	-6.155145	4.072482	7.439342
C	-7.728152	1.467528	9.257051
C	-5.646862	2.831991	9.557701
C	-6.021503	1.569433	7.424132
C	-6.225303	1.546485	8.946691
H	-5.944681	4.971429	9.398353
H	-8.023345	3.979200	10.356936
H	-8.378986	4.849701	8.868030
H	-9.517245	2.629370	8.884002
H	-8.667992	1.806097	6.688443
H	-8.761153	3.564492	6.697988
H	-6.588524	2.804739	5.737672
H	-5.086941	4.153216	7.203996
H	-6.642326	4.953042	7.003169
H	-7.886613	1.429148	10.341603
H	-8.149986	0.543873	8.842625
H	-4.569517	2.891105	9.360648
H	-5.769069	2.818687	10.647493
H	-6.413362	0.647261	6.978191
H	-4.951042	1.605471	7.187955
H	-5.716126	0.675297	9.373329
C	5.582665	4.853266	8.184908
C	5.137169	3.420112	7.855991
C	3.770519	3.140525	8.500236
C	3.882432	3.306270	10.023539
C	4.324812	4.739139	10.358340
C	5.689853	5.014789	9.709013
C	2.737081	4.138218	7.954889
C	4.544561	5.846341	7.639850
C	3.290287	5.732875	9.807902
C	3.176791	5.573269	8.284127
H	6.557163	5.050432	7.724920
H	5.076192	3.284655	6.769407
H	5.880202	2.702083	8.223804
H	3.454609	2.118436	8.263968
H	2.917285	3.089617	10.497258

H	4.603187	2.585839	10.428938
H	4.404454	4.854836	11.444765
H	6.026721	6.028695	9.956964
H	6.442784	4.324872	10.109125
H	2.631698	4.016794	6.869999
H	1.752155	3.936505	8.393144
H	4.861494	6.875045	7.850370
H	4.471934	5.754363	6.549426
H	2.314762	5.559257	10.278035
H	3.584292	6.759466	10.058176
H	2.438420	6.282149	7.893555
C	-3.483643	10.755538	3.396376
C	-4.983815	10.603628	3.691321
C	-5.519454	9.331585	3.016081
C	-5.302349	9.430979	1.498374
C	-3.802960	9.579983	1.197482
C	-3.271609	10.851169	1.877701
C	-4.762334	8.109774	3.558977
C	-2.731980	9.530093	3.938811
C	-3.050042	8.357829	1.745375
C	-3.261465	8.255639	3.263632
H	-3.103866	11.663010	3.878426
H	-5.151750	10.554671	4.773972
H	-5.529374	11.481871	3.325250
H	-6.589009	9.224991	3.228156
H	-5.699033	8.536808	1.002434
H	-5.853955	10.288462	1.094260
H	-3.650616	9.651207	0.115000
H	-2.205594	10.980117	1.654206
H	-3.786960	11.733919	1.480029
H	-4.925443	8.014523	4.639328
H	-5.149127	7.192677	3.098372
H	-1.656285	9.635961	3.752611
H	-2.859768	9.460889	5.025843
H	-3.406753	7.444751	1.253579
H	-1.980197	8.442276	1.518958
H	-2.724407	7.383462	3.652382
C	-8.962129	5.462513	1.234906
C	-8.414090	6.743643	1.882164
C	-8.622377	6.686746	3.403457
C	-10.123034	6.559743	3.707343
C	-10.675796	5.278161	3.064766
C	-10.461900	5.338466	1.544385
C	-7.883266	5.466076	3.973080
C	-8.221629	4.245196	1.810009
C	-9.931214	4.061045	3.634819
C	-8.430118	4.181707	3.330849
H	-8.812866	5.505212	0.150468
H	-7.347702	6.855518	1.651873
H	-8.922229	7.621864	1.465660
H	-8.230421	7.600845	3.862892
H	-10.286611	6.538276	4.791612
H	-10.660627	7.435063	3.323071
H	-11.745832	5.189978	3.282240

H	-10.870344	4.437433	1.070806
H	-11.005832	6.191950	1.121677
H	-6.806984	5.553589	3.781263
H	-8.008457	5.425169	5.061881
H	-8.589751	3.324813	1.340562
H	-7.151533	4.312048	1.579390
H	-10.091653	3.995296	4.717757
H	-10.330700	3.137334	3.198775
H	-7.900851	3.313392	3.738471
C	10.197094	-1.058743	3.562560
C	9.715835	-2.481660	3.239634
C	8.338200	-2.720856	3.876706
C	8.444826	-2.548448	5.399648
C	8.922924	-1.125695	5.728440
C	10.298879	-0.890426	5.086301
C	7.335059	-1.699633	3.318491
C	9.189164	-0.042012	3.004634
C	7.918623	-0.108433	5.165153
C	7.810582	-0.274666	3.641690
H	11.179371	-0.890382	3.107701
H	9.658167	-2.622363	2.153536
H	10.437221	-3.216761	3.616704
H	7.996796	-3.735702	3.644722
H	7.471293	-2.736448	5.868419
H	9.143639	-3.285119	5.814172
H	8.998728	-1.005241	6.814623
H	10.660942	0.115761	5.330154
H	11.030699	-1.597481	5.495558
H	7.233363	-1.825122	2.233712
H	6.342376	-1.872402	3.751578
H	9.531930	0.979256	3.210837
H	9.121027	-0.138968	1.914351
H	6.935880	-0.253163	5.630004
H	8.238162	0.911584	5.410972
H	7.093759	0.451021	3.241949
C	8.631642	6.372228	4.240734
C	7.150181	6.267216	4.634216
C	6.551238	4.973509	4.061029
C	6.675575	4.989838	2.529781
C	8.155836	5.091659	2.130573
C	8.750793	6.384857	2.709029
C	7.318765	3.765762	4.620529
C	9.393631	5.160945	4.800299
C	8.919474	3.883916	2.695328
C	8.800791	3.864704	4.226926
H	9.056606	7.295222	4.650121
H	7.048838	6.277752	5.726164
H	6.598642	7.136624	4.256213
H	5.495163	4.900440	4.343260
H	6.233115	4.079762	2.106878
H	6.115276	5.836682	2.115324
H	8.242026	5.103691	1.038568
H	9.803023	6.479729	2.414450
H	8.227520	7.256502	2.297447

H	7.221274	3.729856	5.712336
H	6.887960	2.834413	4.233453
H	10.457423	5.234496	4.543523
H	9.332263	5.150650	5.895235
H	8.516825	2.954339	2.275116
H	9.974556	3.934126	2.399954
H	9.345360	3.002701	4.627732
C	3.518635	-1.818873	9.667337
C	3.718573	-1.824540	8.143993
C	5.218651	-1.762693	7.817044
C	5.815837	-0.476481	8.408209
C	5.621336	-0.467041	9.932343
C	4.120254	-0.532610	10.253901
C	5.924437	-2.980654	8.432859
C	4.228555	-3.037066	10.278047
C	6.325649	-1.688506	10.542837
C	5.729860	-2.977757	9.957078
H	2.448648	-1.862780	9.898226
H	3.275237	-2.728539	7.709286
H	3.200072	-0.969218	7.693955
H	5.358864	-1.767411	6.730488
H	6.883083	-0.410728	8.164244
H	5.334154	0.402134	7.962257
H	6.047324	0.451024	10.351555
H	3.966410	-0.506537	11.339543
H	3.608531	0.345239	9.840985
H	5.521877	-3.906398	8.004165
H	6.993695	-2.958108	8.189932
H	4.075964	-3.055889	11.364030
H	3.795291	-3.963199	9.881413
H	7.401935	-1.643992	10.336815
H	6.211446	-1.682563	11.633657
H	6.233577	-3.847486	10.393071
C	-4.779629	-3.378571	8.242238
C	-5.776475	-2.380841	7.632799
C	-7.166674	-2.591990	8.252249
C	-7.637903	-4.026478	7.968289
C	-6.645893	-5.028736	8.578456
C	-5.256935	-4.811099	7.958477
C	-7.085409	-2.374893	9.771148
C	-4.704410	-3.158898	9.761130
C	-6.565510	-4.805224	10.096453
C	-6.091659	-3.372755	10.385839
H	-3.789347	-3.226509	7.799149
H	-5.437056	-1.353230	7.810403
H	-5.825022	-2.515223	6.545354
H	-7.875617	-1.879250	7.816716
H	-8.638660	-4.183644	8.388576
H	-7.719338	-4.189118	6.886730
H	-6.983544	-6.050681	8.374324
H	-4.542973	-5.534104	8.371380
H	-5.296060	-4.988026	6.876744
H	-6.769721	-1.347382	9.988859
H	-8.076405	-2.504019	10.222907

H	-3.980045	-3.852040	10.205929
H	-4.346515	-2.145084	9.977582
H	-7.547264	-4.976432	10.554345
H	-5.875240	-5.528551	10.547331
H	-6.035475	-3.215081	11.468535
C	-11.209542	-4.737684	-1.558754
C	-10.682265	-3.425144	-0.958919
C	-9.177705	-3.292814	-1.241302
C	-8.434963	-4.480371	-0.610041
C	-8.956371	-5.795874	-1.208784
C	-10.461576	-5.921624	-0.926844
C	-8.942671	-3.295268	-2.759703
C	-10.969521	-4.735920	-3.076427
C	-8.722387	-5.791876	-2.727326
C	-9.465749	-4.607662	-3.363956
H	-12.282160	-4.830156	-1.356143
H	-11.222305	-2.571833	-1.386645
H	-10.864985	-3.406256	0.122380
H	-8.804040	-2.356094	-0.813263
H	-7.356741	-4.386897	-0.787687
H	-8.578198	-4.479346	0.477312
H	-8.425861	-6.641110	-0.756916
H	-10.842752	-6.866801	-1.332196
H	-10.640735	-5.946537	0.154942
H	-9.450794	-2.439960	-3.221326
H	-7.873216	-3.181890	-2.974752
H	-11.360434	-5.659706	-3.520054
H	-11.513887	-3.905829	-3.542662
H	-7.649222	-5.721860	-2.942343
H	-9.072142	-6.735022	-3.164490
H	-9.297338	-4.606127	-4.446493
C	-4.151657	-8.246471	-1.630770
C	-2.770139	-8.009271	-2.259839
C	-2.310032	-6.571161	-1.975071
C	-3.323326	-5.584345	-2.574969
C	-4.706495	-5.815166	-1.946899
C	-5.160308	-7.255328	-2.231385
C	-2.227500	-6.354515	-0.456176
C	-4.063948	-8.025858	-0.112685
C	-4.617888	-5.600938	-0.428022
C	-3.607871	-6.587685	0.177323
H	-4.477879	-9.272106	-1.835127
H	-2.043996	-8.724311	-1.854665
H	-2.815490	-8.182230	-3.341958
H	-1.324653	-6.404454	-2.423984
H	-2.995481	-4.553234	-2.395574
H	-3.377734	-5.714845	-3.662565
H	-5.427355	-5.110960	-2.376438
H	-6.156075	-7.427347	-1.804978
H	-5.247660	-7.415364	-3.312929
H	-1.491759	-7.039119	-0.017042
H	-1.881273	-5.336584	-0.239897
H	-5.040070	-8.212332	0.351437
H	-3.360538	-8.740456	0.331526

H	-4.313190	-4.570036	-0.210723
H	-5.604177	-5.742993	0.030257
H	-3.545641	-6.433111	1.260136
C	-1.091082	-1.422624	-7.320983
C	-2.118665	-0.445375	-7.912288
C	-1.699577	0.999783	-7.601102
C	-1.625878	1.191741	-6.078549
C	-0.597078	0.218679	-5.482204
C	-1.018757	-1.224470	-5.799148
C	-0.317870	1.272524	-8.215339
C	0.288883	-1.143672	-7.936475
C	0.781766	0.492995	-6.102104
C	0.714197	0.299358	-7.624663
H	-1.391823	-2.452208	-7.543741
H	-2.195491	-0.590121	-8.996667
H	-3.112454	-0.646968	-7.494322
H	-2.433290	1.694795	-8.024080
H	-1.346848	2.225919	-5.843016
H	-2.611238	1.019181	-5.628946
H	-0.546620	0.356923	-4.396667
H	-0.302566	-1.930568	-5.361586
H	-1.993227	-1.440015	-5.344256
H	-0.360726	1.159177	-9.305362
H	-0.015757	2.307887	-8.016381
H	1.028130	-1.848858	-7.537446
H	0.255705	-1.300050	-9.021465
H	1.103430	1.514733	-5.866731
H	1.529989	-0.182225	-5.669431
H	1.697965	0.495874	-8.064927
C	1.022990	-4.101475	-1.677921
C	2.402327	-3.840276	-2.302250
C	2.845814	-2.400963	-1.997863
C	1.821960	-1.417794	-2.585699
C	0.440900	-1.672620	-1.962285
C	0.003731	-3.113917	-2.266409
C	2.924432	-2.203642	-0.476129
C	1.106741	-3.900119	-0.156938
C	0.525621	-1.477648	-0.440594
C	1.546192	-2.460858	0.152684
H	0.708623	-5.127930	-1.896266
H	3.136151	-4.552372	-1.905823
H	2.359975	-3.999312	-3.386624
H	3.829666	-2.217136	-2.443428
H	2.137912	-0.385513	-2.392237
H	1.770075	-1.534407	-3.674994
H	-0.287494	-0.970972	-2.383213
H	-0.990427	-3.302874	-1.843431
H	-0.080767	-3.260515	-3.350083
H	3.667474	-2.885651	-0.045330
H	3.258879	-1.184823	-0.245943
H	0.132354	-4.103808	0.303582
H	1.817790	-4.612550	0.278503
H	0.818393	-0.446344	-0.209262
H	-0.459430	-1.636963	0.014663

H	1.605629	-2.320023	1.237526
C	8.023104	-3.607293	-0.308823
C	6.523021	-3.708933	0.007411
C	5.943500	-4.982006	-0.628508
C	6.144504	-4.927483	-2.150701
C	7.643650	-4.828874	-2.472796
C	8.218922	-3.556410	-1.831828
C	6.672625	-6.210637	-0.063146
C	8.746666	-4.839410	0.256235
C	8.368626	-6.057601	-1.902381
C	8.173230	-6.115066	-0.379632
H	8.434192	-2.699010	0.145202
H	6.367195	-3.725527	1.092849
H	5.997885	-2.825136	-0.374803
H	4.874078	-5.052717	-0.401300
H	5.716719	-5.822347	-2.618757
H	5.612269	-4.065226	-2.570338
H	7.784520	-4.789583	-3.558458
H	9.285293	-3.463551	-2.070987
H	7.723715	-2.669771	-2.246099
H	6.520292	-6.273686	1.021131
H	6.254519	-7.127792	-0.495405
H	9.822525	-4.768941	0.054839
H	8.630453	-4.877266	1.346107
H	7.980315	-6.972389	-2.366273
H	9.437537	-6.009394	-2.143358
H	8.690320	-6.991982	0.025195
C	-6.145139	3.526164	-6.879512
C	-5.390147	2.316167	-6.308130
C	-5.917565	1.024194	-6.951240
C	-7.417546	0.881683	-6.650913
C	-8.178049	2.088006	-7.223037
C	-7.644583	3.377298	-6.579540
C	-5.707601	1.090089	-8.471886
C	-5.934029	3.585591	-8.400317
C	-7.962022	2.151577	-8.742914
C	-6.463489	2.297224	-9.048576
H	-5.767808	4.446062	-6.419600
H	-4.314866	2.419451	-6.497432
H	-5.517794	2.273632	-5.219622
H	-5.377512	0.162897	-6.542831
H	-7.801914	-0.047803	-7.088148
H	-7.580216	0.813618	-5.568434
H	-9.247065	1.984546	-7.007284
H	-8.193208	4.245264	-6.965042
H	-7.811623	3.353658	-5.495788
H	-4.638295	1.171223	-8.701750
H	-6.062865	0.164533	-8.940892
H	-6.451633	4.457711	-8.818035
H	-4.868527	3.710890	-8.627836
H	-8.356274	1.244646	-9.217109
H	-8.516802	2.997430	-9.166843
H	-6.311367	2.341839	-10.132605
C	8.008219	1.755800	-2.055069

C	7.593012	0.312666	-2.379957
C	6.224393	0.011822	-1.749618
C	6.315638	0.188946	-0.226217
C	6.727666	1.631774	0.104521
C	8.094808	1.928619	-0.530892
C	5.179127	0.986881	-2.313063
C	6.958369	2.726156	-2.618248
C	5.681445	2.602824	-0.464001
C	5.588511	2.431806	-1.987931
H	8.984168	1.968118	-2.505127
H	7.547091	0.169444	-3.466286
H	8.344843	-0.389238	-1.999125
H	5.930104	-1.017360	-1.982992
H	5.349237	-0.042599	0.237801
H	7.044805	-0.515540	0.191958
H	6.792613	1.755583	1.191030
H	8.410065	2.950080	-0.285544
H	8.855642	1.255203	-0.117830
H	5.088533	0.856878	-3.398295
H	4.193088	0.769689	-1.884826
H	7.253857	3.761814	-2.410649
H	6.900045	2.626166	-3.708826
H	4.703918	2.414092	-0.003959
H	5.953589	3.636190	-0.216901
H	4.841773	3.124509	-2.391408
C	-6.791935	-8.454503	4.420564
C	-8.298108	-8.567475	4.702103
C	-9.034962	-7.375757	4.071381
C	-8.497075	-6.068443	4.673092
C	-6.991285	-5.948819	4.391680
C	-6.259694	-7.144390	5.021229
C	-8.794205	-7.373787	2.553876
C	-6.557249	-8.450223	2.902061
C	-6.755563	-5.950948	2.873403
C	-7.289068	-7.257861	2.267105
H	-6.268526	-9.304839	4.871211
H	-8.687597	-9.508866	4.296131
H	-8.477286	-8.590516	5.783915
H	-10.108657	-7.458656	4.272861
H	-9.029596	-5.210468	4.245123
H	-8.680314	-6.047921	5.754229
H	-6.609888	-5.016399	4.822002
H	-5.180466	-7.060780	4.844478
H	-6.402948	-7.142741	6.108636
H	-9.192267	-8.293201	2.107715
H	-9.331309	-6.538113	2.089116
H	-5.483243	-8.390597	2.687696
H	-6.916019	-9.389013	2.463067
H	-7.257087	-5.090732	2.413706
H	-5.685188	-5.845527	2.658581
H	-7.120632	-7.257347	1.184572
C	-5.793994	4.425693	-2.705096
C	-6.802257	5.408696	-3.319663
C	-6.368442	6.852063	-3.020579

C	-6.309728	7.061108	-1.499625
C	-5.300252	6.082336	-0.880053
C	-5.736568	4.640943	-1.184949
C	-4.976448	7.100129	-3.621918
C	-4.403650	4.679977	-3.307782
C	-3.911038	6.331945	-1.487106
C	-3.963643	6.121144	-3.007975
H	-6.105215	3.397400	-2.919257
H	-6.868508	5.252088	-4.403091
H	-7.803240	5.224569	-2.910907
H	-7.088386	7.551174	-3.460135
H	-6.020303	8.094418	-1.272996
H	-7.302278	6.906278	-1.059479
H	-5.260462	6.232789	0.204303
H	-5.034450	3.931139	-0.730884
H	-6.718834	4.443060	-0.738884
H	-5.008271	6.974421	-4.710963
H	-4.663510	8.133901	-3.431605
H	-3.678027	3.970339	-2.891989
H	-4.426400	4.511182	-4.391179
H	-3.579140	7.352281	-1.259998
H	-3.176445	5.652556	-1.037920
H	-2.972489	6.300057	-3.439082
C	-6.255573	-5.545081	-7.290693
C	-4.863228	-5.279256	-7.883397
C	-4.437406	-3.834254	-7.580748
C	-5.453419	-2.863512	-8.201896
C	-6.847507	-3.122984	-7.610204
C	-7.267006	-4.569923	-7.912420
C	-4.397440	-3.623467	-6.059313
C	-6.210485	-5.330228	-5.769919
C	-6.801423	-2.914502	-6.088644
C	-5.788825	-3.885263	-5.462134
H	-6.557311	-6.575590	-7.507753
H	-4.134363	-5.982656	-7.463021
H	-4.877913	-5.447788	-8.967064
H	-3.444253	-3.647128	-8.003734
H	-5.149526	-1.827376	-8.009724
H	-5.477810	-2.989734	-9.291083
H	-7.570271	-2.430200	-8.054849
H	-8.269873	-4.762443	-7.512220
H	-7.323927	-4.726316	-8.996524
H	-3.660411	-4.296424	-5.604612
H	-4.075864	-2.600347	-5.829860
H	-7.194402	-5.537019	-5.331580
H	-5.505339	-6.033797	-5.311095
H	-6.521623	-1.879189	-5.859171
H	-7.796182	-3.077043	-5.656210
H	-5.756944	-3.734803	-4.377424
C	-0.790295	5.127662	-6.990934
C	-0.578043	5.211384	-8.510316
C	-1.321318	6.433371	-9.071782
C	-2.821769	6.299467	-8.769850
C	-3.040092	6.218426	-7.251076

C	-2.292097	4.997226	-6.694194
C	-0.780585	7.708177	-8.405997
C	-0.250422	6.405801	-6.330747
C	-2.494599	7.493510	-6.589765
C	-0.993398	7.630512	-6.886198
H	-0.259831	4.255790	-6.592743
H	0.492347	5.284329	-8.737640
H	-0.942673	4.295710	-8.991501
H	-1.168147	6.491408	-10.155044
H	-3.366530	7.156777	-9.183507
H	-3.225363	5.403058	-9.256077
H	-4.110385	6.122460	-7.038208
H	-2.456115	4.916349	-5.612749
H	-2.686567	4.077645	-7.143351
H	0.286238	7.827511	-8.630681
H	-1.290321	8.589945	-8.812799
H	-0.377474	6.349585	-5.242770
H	0.825742	6.501145	-6.519266
H	-3.033788	8.371865	-6.964710
H	-2.662797	7.457162	-5.506550
H	-0.606369	8.540380	-6.414394
C	5.017987	-5.163215	3.602677
C	4.550005	-6.597011	3.309750
C	3.170769	-6.832843	3.944609
C	3.267425	-6.624864	5.463770
C	3.732216	-5.191125	5.762547
C	5.109831	-4.959347	5.122703
C	2.162615	-5.832846	3.357735
C	4.005079	-4.167740	3.016168
C	2.722963	-4.195212	5.170686
C	2.624844	-4.397040	3.650856
H	6.001426	-4.997266	3.149446
H	4.499588	-6.762926	2.226855
H	5.275138	-7.317403	3.707453
H	2.838842	-7.855471	3.734032
H	2.292808	-6.810059	5.931406
H	3.969789	-7.346158	5.898830
H	3.800929	-5.045303	6.846089
H	5.462418	-3.944937	5.345457
H	5.844992	-5.650883	5.552000
H	2.068043	-5.983880	2.275570
H	1.168928	-6.003783	3.789245
H	4.338461	-3.139269	3.200832
H	3.943866	-4.290102	1.928032
H	1.738811	-4.337313	5.633364
H	3.032905	-3.167278	5.394857
H	1.904476	-3.686539	3.230729
C	2.885861	-7.720473	-0.224026
C	1.380449	-7.836715	0.060238
C	0.831746	-9.127405	-0.567453
C	1.066321	-9.094836	-2.085465
C	2.570967	-8.981733	-2.375559
C	3.115317	-7.691625	-1.742905
C	1.563584	-10.337048	0.034187

C	3.612190	-8.933679	0.377243
C	3.298545	-10.191444	-1.768959
C	3.069601	-10.226871	-0.250218
H	3.274956	-6.799648	0.224076
H	1.200404	-7.837823	1.142049
H	0.852810	-6.966190	-0.348095
H	-0.241537	-9.208497	-0.363066
H	0.660680	-10.002692	-2.548186
H	0.532705	-8.246545	-2.531011
H	2.735776	-8.958131	-3.458300
H	4.185536	-7.588623	-1.959671
H	2.618283	-6.818383	-2.182661
H	1.387650	-10.384574	1.115678
H	1.167097	-11.266484	-0.392169
H	4.691328	-8.852328	0.198806
H	3.471916	-8.955453	1.464721
H	2.932526	-11.118633	-2.226308
H	4.371916	-10.133080	-1.986751
H	3.588599	-11.090258	0.180427
C	0.402728	0.740554	8.213543
C	-0.620640	1.733627	7.641612
C	-1.995669	1.488736	8.282141
C	-2.449704	0.052549	7.979378
C	-1.431087	-0.945142	8.552038
C	-0.057409	-0.693774	7.911127
C	-1.889661	1.678387	9.803189
C	0.502652	0.932685	9.734742
C	-1.326078	-0.749074	10.072323
C	-0.869288	0.685033	10.380423
H	1.382145	0.916686	7.755471
H	-0.294017	2.762971	7.832661
H	-0.687232	1.619041	6.552856
H	-2.723563	2.198202	7.873345
H	-3.439936	-0.128422	8.414750
H	-2.548626	-0.090921	6.896565
H	-1.756522	-1.968293	8.334547
H	0.675243	-1.412995	8.297013
H	-0.113813	-0.850850	6.827095
H	-1.586017	2.706497	10.034761
H	-2.869987	1.525009	10.270380
H	1.245830	0.242881	10.152897
H	0.848681	1.947797	9.964025
H	-2.296396	-0.944490	10.544670
H	-0.616402	-1.469711	10.496596
H	-0.795512	0.823116	11.464739
C	-6.953748	-0.665938	2.612009
C	-7.396424	-2.109071	2.325668
C	-8.781562	-2.359169	2.941943
C	-8.710964	-2.135766	4.460348
C	-8.271568	-0.692683	4.752492
C	-6.887914	-0.446708	4.131247
C	-9.795083	-1.379489	2.330556
C	-7.971962	0.309153	2.000959
C	-9.286052	0.282852	4.136141

C	-9.358192	0.065570	2.616986
H	-5.966060	-0.489822	2.172050
H	-7.428014	-2.285708	1.243756
H	-6.667043	-2.815076	2.741016
H	-9.095414	-3.388445	2.736090
H	-9.689771	-2.330863	4.915024
H	-8.004902	-2.842659	4.912714
H	-8.221403	-0.535862	5.835515
H	-6.553472	0.574766	4.349662
H	-6.149406	-1.123422	4.578062
H	-9.871046	-1.541887	1.248571
H	-10.792615	-1.560944	2.748691
H	-7.656347	1.344053	2.180869
H	-8.014898	0.175857	0.913233
H	-10.274941	0.130656	4.585309
H	-8.994463	1.317237	4.355205
H	-10.082324	0.761548	2.179382
C	-10.945640	0.311217	-2.679038
C	-9.559267	0.559885	-3.292810
C	-9.122960	2.007744	-3.020242
C	-10.141725	2.972699	-3.645884
C	-11.529824	2.730373	-3.033213
C	-11.959813	1.280554	-3.305389
C	-9.063714	2.244464	-1.503257
C	-10.881262	0.551965	-1.162844
C	-11.464471	2.964760	-1.516135
C	-10.449055	1.999897	-0.885092
H	-11.254871	-0.721360	-2.874667
H	-8.828763	-0.139795	-2.869084
H	-9.587773	0.372817	-4.373155
H	-8.134080	2.182629	-3.458213
H	-9.830613	4.010462	-3.475259
H	-10.179801	2.827886	-4.732362
H	-12.254570	3.418992	-3.481092
H	-12.958705	1.099932	-2.889990
H	-12.030499	1.105825	-4.385876
H	-8.324489	1.575772	-1.045836
H	-8.734533	3.269768	-1.295346
H	-11.860816	0.357637	-0.709294
H	-10.174015	-0.147105	-0.700393
H	-11.177005	4.002463	-1.307916
H	-12.454726	2.814627	-1.069123
H	-10.403428	2.168828	0.196403
C	-3.957848	0.832938	-1.271510
C	-5.457235	0.716099	-0.957231
C	-6.016179	-0.578651	-1.567278
C	-5.811395	-0.553768	-3.089741
C	-4.312883	-0.440099	-3.409816
C	-3.758194	0.854088	-2.794720
C	-5.270901	-1.784078	-0.973733
C	-3.218080	-0.376063	-0.678209
C	-3.571767	-1.645575	-2.811228
C	-3.770902	-1.673304	-1.288132
H	-3.561441	1.756659	-0.835950

H	-5.616080	0.720451	0.127887
H	-5.994065	1.583720	-1.359726
H	-7.085136	-0.660159	-1.341502
H	-6.224650	-1.464619	-3.539661
H	-6.354895	0.291414	-3.529206
H	-4.169323	-0.421985	-4.495684
H	-2.692575	0.957437	-3.032927
H	-4.265042	1.724302	-3.229243
H	-5.425572	-1.826140	0.111229
H	-5.674260	-2.716305	-1.387361
H	-2.142762	-0.294149	-0.878156
H	-3.337020	-0.392290	0.411905
H	-3.945274	-2.575676	-3.256451
H	-2.502950	-1.586865	-3.050288
H	-3.242286	-2.533681	-0.863200
C	0.835687	3.715954	-3.431207
C	1.396212	5.006326	-2.813684
C	1.205879	4.977606	-1.289249
C	-0.291511	4.862037	-0.965345
C	-0.856705	3.571315	-1.578124
C	-0.660740	3.603437	-3.101774
C	1.947149	3.764450	-0.706532
C	1.578424	2.506242	-2.843088
C	-0.109946	2.361698	-0.995105
C	1.387859	2.470924	-1.318984
H	0.972160	3.738564	-4.517933
H	2.460188	5.109815	-3.058587
H	0.886438	5.878927	-3.239887
H	1.606715	5.898253	-0.851041
H	-0.442220	4.860709	0.120999
H	-0.830363	5.732434	-1.358922
H	-1.924394	3.491299	-1.346419
H	-1.078183	2.695665	-3.554164
H	-1.206470	4.451337	-3.533311
H	3.021387	3.844232	-0.912648
H	1.834805	3.743614	0.384238
H	1.201277	1.579038	-3.291475
H	2.645935	2.564695	-3.087550
H	-0.257698	2.316059	0.090669
H	-0.518068	1.431891	-1.409665
H	1.918691	1.607983	-0.902090
C	-1.768722	3.423242	2.585668
C	-2.211883	1.983049	2.285610
C	-3.592082	1.723989	2.909223
C	-3.510369	1.929534	4.429592
C	-3.070443	3.369606	4.735475
C	-1.691762	3.624599	4.106855
C	-4.611269	2.709574	2.317049
C	-2.792595	4.404229	1.993810
C	-4.090627	4.351103	4.138295
C	-4.173893	4.151686	2.617261
H	-0.784557	3.605755	2.140450
H	-2.251374	1.819160	1.201953
H	-1.478609	1.273085	2.687124

H	-3.906293	0.696831	2.693574
H	-4.485521	1.727880	4.889235
H	-2.800134	1.218217	4.868283
H	-3.012348	3.513690	5.819870
H	-1.356859	4.643833	4.334819
H	-0.949158	2.943565	4.540110
H	-4.695148	2.559870	1.233818
H	-5.605435	2.521969	2.740454
H	-2.476821	5.437319	2.183573
H	-2.843523	4.283733	0.904937
H	-5.075951	4.192400	4.593013
H	-3.798585	5.383187	4.367381
H	-4.902076	4.851892	2.193337
C	-2.140034	-3.129490	2.227652
C	-1.579904	-1.836870	2.840818
C	-1.780258	-1.855584	4.364125
C	-3.280050	-1.964966	4.678938
C	-3.844880	-3.257894	4.070487
C	-3.638889	-3.235788	2.547980
C	-1.046236	-3.067155	4.959185
C	-1.404570	-4.337587	2.828102
C	-3.105360	-4.465939	4.665862
C	-1.605172	-4.362900	4.351116
H	-1.996411	-3.114016	1.141722
H	-0.514066	-1.737870	2.602243
H	-2.084420	-0.965519	2.405890
H	-1.379690	-0.933351	4.799231
H	-3.437845	-1.959122	5.764260
H	-3.813862	-1.095535	4.276458
H	-4.914279	-3.333494	4.295695
H	-4.055936	-4.145189	2.098505
H	-4.179395	-2.389071	2.107637
H	0.029544	-2.991647	4.759609
H	-1.165751	-3.080897	6.049305
H	-1.781402	-5.266506	2.383013
H	-0.335326	-4.283628	2.590269
H	-3.260321	-4.504416	5.750908
H	-3.513396	-5.397164	4.254413
H	-1.079512	-5.224707	4.776814
C	3.778592	-2.322809	-6.215588
C	4.370054	-1.048895	-5.592774
C	5.869991	-0.961839	-5.914589
C	6.586098	-2.193916	-5.340460
C	6.000421	-3.471034	-5.962441
C	4.500069	-3.551479	-5.640855
C	6.062354	-0.926279	-7.438572
C	3.976111	-2.283236	-7.738863
C	6.191966	-3.428986	-7.486343
C	5.474968	-2.200063	-8.065918
H	2.709375	-2.383046	-5.984811
H	3.849880	-0.164158	-5.979286
H	4.218365	-1.056548	-4.506549
H	6.289464	-0.052590	-5.470073
H	7.661646	-2.133642	-5.546426

H	6.473368	-2.221009	-4.249858
H	6.512044	-4.348101	-5.551293
H	4.073415	-4.469602	-6.062736
H	4.350348	-3.603028	-4.555542
H	5.573190	-0.039652	-7.859818
H	7.128829	-0.844667	-7.681066
H	3.539447	-3.178473	-8.198072
H	3.449692	-1.420362	-8.164412
H	7.260562	-3.390821	-7.730195
H	5.795888	-4.345230	-7.940859
H	5.613076	-2.171460	-9.152361
C	0.721652	-5.056553	-5.818363
C	-0.783627	-5.159820	-5.528437
C	-1.343439	-6.451615	-6.143927
C	-1.113499	-6.432971	-7.662883
C	0.390972	-6.332884	-7.958610
C	0.946444	-5.041600	-7.338140
C	-0.618243	-7.661517	-5.534813
C	1.441313	-6.269982	-5.209574
C	1.111925	-7.542784	-7.344523
C	0.887597	-7.564320	-5.824832
H	1.118673	-4.134919	-5.378959
H	-0.960212	-5.150914	-4.446092
H	-1.306408	-4.288903	-5.942147
H	-2.416609	-6.523443	-5.935517
H	-1.527021	-7.341636	-8.116959
H	-1.642533	-5.584544	-8.113605
H	0.552479	-6.319197	-9.042020
H	2.016664	-4.947942	-7.559096
H	0.454186	-4.168436	-7.783390
H	-0.791045	-7.699059	-4.452424
H	-1.022647	-8.591532	-5.952379
H	2.520422	-6.197726	-5.392055
H	1.304369	-6.281976	-4.121520
H	0.737904	-8.471018	-7.793195
H	2.184979	-7.493791	-7.566153
H	1.401859	-8.427858	-5.388843
C	3.402169	2.246039	4.326748
C	1.903293	2.141576	4.647747
C	1.331400	0.850464	4.042007
C	1.529699	0.871587	2.518630
C	3.027650	0.972878	2.191842
C	3.595305	2.263469	2.802651
C	2.069664	-0.360025	4.634014
C	4.134894	1.032020	4.918541
C	3.761774	-0.237620	2.788911
C	3.569103	-0.261633	4.312901
H	3.807815	3.167183	4.759246
H	1.749246	2.148709	5.733542
H	1.371620	3.012878	4.246361
H	0.262829	0.777781	4.272572
H	1.107245	-0.036575	2.071827
H	0.991016	1.720452	2.080335
H	3.166588	0.988331	1.105331

H	4.660657	2.358000	2.559639
H	3.093493	3.137081	2.369101
H	1.919422	-0.399338	5.719702
H	1.657095	-1.289590	4.223491
H	5.209942	1.105093	4.713776
H	4.020609	1.018264	6.009187
H	3.378934	-1.165340	2.346659
H	4.829965	-0.187752	2.545095
H	4.092719	-1.125594	4.736751

Adamantane$_{37}$

```
      962
E=-1802.8568
```

C	5.863542	4.478196	-7.632565
C	5.445469	3.036888	-7.961855
C	4.080388	2.733921	-7.324897
C	4.180723	2.903739	-5.801236
C	4.595651	4.344715	-5.466078
C	5.959223	4.643709	-6.108121
C	3.032469	3.712314	-7.877532
C	4.811039	5.451888	-8.184929
C	3.546745	5.319124	-6.023803
C	3.444721	5.155415	-7.547954
H	6.836964	4.692031	-8.087353
H	5.393055	2.898864	-9.048563
H	6.199064	2.332702	-7.588801
H	3.784045	1.706048	-7.561422
H	3.216918	2.670605	-5.332635
H	4.911872	2.196808	-5.390719
H	4.667080	4.463313	-4.379395
H	6.276599	5.663790	-5.859784
H	6.722030	3.967851	-5.702755
H	2.935398	3.587533	-8.962816
H	2.048826	3.493716	-7.444527
H	5.108433	6.486358	-7.974155
H	4.746224	5.357123	-9.275606
H	2.571820	5.128828	-5.558910
H	3.821027	6.351129	-5.773403
H	2.696080	5.850505	-7.943727
C	-1.650283	-5.916079	9.734907
C	-1.450738	-5.934358	8.211610
C	0.049062	-5.870897	7.883698
C	0.642585	-4.577843	8.463515
C	0.448465	-4.555768	9.987568
C	-1.052330	-4.623000	10.310123
C	0.758627	-7.081375	8.509839
C	-0.936585	-7.126820	10.355945
C	1.156565	-5.769800	10.608419
C	0.564451	-7.065842	10.034032
H	-2.720073	-5.961158	9.966482
H	-1.891504	-6.843403	7.784886
H	-1.971896	-5.084512	7.754328
H	0.188998	-5.884618	6.797182

H	1.709566	-4.511041	8.218677
H	0.158175	-3.704560	8.010106
H	0.871837	-3.632843	10.398681
H	-1.205959	-4.587973	11.395543
H	-1.566768	-3.750286	9.889764
H	0.358703	-8.011991	8.089304
H	1.827748	-7.057763	8.266412
H	-1.088829	-7.136681	11.442095
H	-1.367203	-8.057638	9.967480
H	2.232659	-5.723881	10.401704
H	1.042638	-5.754739	11.699180
H	1.070865	-7.930261	10.477400
C	-2.016470	-10.937449	3.996603
C	-0.641088	-10.713558	3.349471
C	-0.162917	-9.280332	3.629057
C	-1.174146	-8.282988	3.043249
C	-2.551128	-8.500484	3.689410
C	-3.023023	-9.935825	3.410022
C	-0.058154	-9.065701	5.146867
C	-1.906502	-10.718903	5.513538
C	-2.440315	-8.288331	5.207120
C	-1.432290	-9.285577	5.798417
H	-2.355592	-11.959619	3.795918
H	0.083170	-11.436122	3.744544
H	-0.702465	-10.885214	2.267935
H	0.818058	-9.123117	3.167258
H	-0.833676	-7.255339	3.218958
H	-1.244227	-8.412094	1.956384
H	-3.270534	-7.788786	3.269897
H	-4.014735	-10.098230	3.849450
H	-3.126258	-10.094140	2.329625
H	0.676453	-9.757953	5.575790
H	0.301051	-8.051443	5.359204
H	-2.878218	-10.895988	5.990410
H	-1.204454	-11.440829	5.947946
H	-2.122490	-7.260690	5.421035
H	-3.421829	-8.420894	5.678312
H	-1.354211	-9.132474	6.880411
C	3.395724	7.560584	2.557842
C	2.957241	6.123033	2.238837
C	1.590069	5.842565	2.881589
C	1.697172	6.020550	4.403857
C	2.132523	7.457848	4.728739
C	3.498117	7.734332	4.080932
C	0.553850	6.831571	2.325795
C	2.354857	8.544954	2.002361
C	1.095247	8.442853	4.167883
C	0.986537	8.270996	2.645092
H	4.370605	7.758373	2.098935
H	2.899770	5.978928	1.153175
H	3.702343	5.411063	2.614168
H	1.279166	4.817336	2.652395
H	0.731687	5.803421	4.876669
H	2.419904	5.306372	4.816731

H	2.208745	7.582273	5.814445
H	3.829981	8.751560	4.321928
H	4.252908	7.050765	4.488369
H	0.451908	6.701320	1.241600
H	-0.431381	6.629022	2.762977
H	2.666825	9.576604	2.205760
H	2.285559	8.444246	0.912492
H	0.119211	8.268685	4.636749
H	1.384188	9.472599	4.410989
H	0.246198	8.973662	2.247086
C	-1.224631	8.138201	8.921475
C	-1.009618	8.149729	7.400219
C	-1.585757	6.865131	6.784732
C	-0.876297	5.646177	7.394047
C	-1.090746	5.628008	8.915424
C	-0.516241	6.915947	9.525426
C	-3.089221	6.783894	7.090980
C	-2.729166	8.054122	9.221895
C	-2.595451	5.550401	9.216377
C	-3.309289	6.769006	8.611657
H	-0.813024	9.054662	9.358267
H	-1.493347	9.029092	6.958110
H	0.060422	8.228569	7.172732
H	-1.431865	6.875753	5.700070
H	-1.265126	4.722864	6.947772
H	0.195876	5.681316	7.165870
H	-0.583663	4.758508	9.347725
H	-0.645218	6.906658	10.614575
H	0.562440	6.973430	9.335364
H	-3.611852	7.638113	6.643763
H	-3.516422	5.880020	6.640042
H	-2.897475	8.065840	10.305653
H	-3.244456	8.931186	8.812026
H	-3.014566	4.625255	8.802534
H	-2.761242	5.516248	10.300034
H	-4.381993	6.711679	8.826757
C	-6.356790	4.051796	8.963796
C	-7.859505	3.969576	9.273104
C	-8.445990	2.687679	8.661770
C	-8.235608	2.707052	7.139991
C	-6.733821	2.786120	6.824885
C	-6.151620	4.067555	7.441254
C	-7.730569	1.465771	9.258349
C	-5.646959	2.826442	9.560114
C	-6.022579	1.564262	7.426493
C	-6.227382	1.541913	8.948926
H	-5.940735	4.966401	9.400266
H	-8.021829	3.978144	10.357682
H	-8.374926	4.849080	8.868424
H	-9.517283	2.630852	8.884002
H	-8.668163	1.805692	6.689099
H	-8.758090	3.564258	6.698329
H	-6.586259	2.800362	5.739495
H	-5.083120	4.146286	7.206570

H	-6.636902	4.948947	7.004644
H	-7.889785	1.427842	10.342806
H	-8.153843	0.542833	8.843792
H	-4.569382	2.883542	9.363732
H	-5.769878	2.813523	10.649831
H	-6.415855	0.642748	6.980439
H	-4.951905	1.598293	7.190987
H	-5.720080	0.669851	9.376013
C	5.574961	4.861862	8.174864
C	5.132108	3.427132	7.849266
C	3.766916	3.145842	8.495858
C	3.880491	3.314728	10.018693
C	4.320236	4.749178	10.350179
C	5.683821	5.026517	9.698516
C	2.730616	4.140276	7.949987
C	4.534006	5.851668	7.629298
C	3.282852	5.739643	9.799227
C	3.167681	5.576891	8.275910
H	6.548424	5.060240	7.713208
H	5.069983	3.289474	6.763024
H	5.877168	2.711399	8.217457
H	3.452888	2.122630	8.261954
H	2.916439	3.096909	10.494104
H	4.603327	2.596618	10.424503
H	4.401069	4.867115	11.436275
H	6.018839	6.041614	9.944090
H	6.438761	4.338980	10.098939
H	2.624058	4.016559	6.865471
H	1.746708	3.937287	8.389933
H	4.849009	6.881448	7.837440
H	4.460132	5.757457	6.539149
H	2.308325	5.564829	10.270985
H	3.574983	6.767337	10.047156
H	2.427273	6.283441	7.884972
C	-3.465692	10.768696	3.359040
C	-4.969898	10.654255	3.650330
C	-5.526002	9.371512	3.012935
C	-5.299543	9.418359	1.494064
C	-3.796143	9.529721	1.196918
C	-3.244367	10.811846	1.839293
C	-4.794688	8.153754	3.598625
C	-2.739899	9.547394	3.944355
C	-3.069061	8.311858	1.787591
C	-3.289896	8.262129	3.307133
H	-3.071316	11.683856	3.814078
H	-5.144091	10.643011	4.733040
H	-5.497028	11.530381	3.253477
H	-6.598411	9.291688	3.222348
H	-5.710503	8.516363	1.024626
H	-5.832919	10.272638	1.059815
H	-3.637121	9.563513	0.113582
H	-2.175021	10.913711	1.617735
H	-3.741053	11.690905	1.410881
H	-4.964915	8.096084	4.680537

H	-5.196365	7.229791	3.165288
H	-1.661494	9.627205	3.760861
H	-2.874356	9.515308	5.032312
H	-3.440432	7.390355	1.323119
H	-1.996708	8.369088	1.564550
H	-2.771264	7.392948	3.726412
C	-8.950124	5.458218	1.235100
C	-8.393112	6.737479	1.878382
C	-8.602911	6.687332	3.399705
C	-10.104684	6.572445	3.702903
C	-10.666425	5.292781	3.064299
C	-10.450993	5.346299	1.543881
C	-7.873241	5.463200	3.974051
C	-8.219043	4.237447	1.814915
C	-9.931257	4.072165	3.639067
C	-8.429093	4.180708	3.335808
H	-8.799767	5.496099	0.150634
H	-7.325762	6.840694	1.648506
H	-8.894456	7.617993	1.458498
H	-8.204552	7.600082	3.856304
H	-10.269198	6.555899	4.787116
H	-10.635524	7.450385	3.315238
H	-11.737240	5.213241	3.281274
H	-10.865734	4.446687	1.073081
H	-10.988305	6.202320	1.117851
H	-6.796204	5.542113	3.782740
H	-7.999516	5.426947	5.062891
H	-8.593609	3.318203	1.348344
H	-7.148317	4.295613	1.584869
H	-10.092959	4.011311	4.722103
H	-10.337235	3.149938	3.205887
H	-7.906543	3.309913	3.746792
C	10.195997	-1.061973	3.562108
C	9.714929	-2.485300	3.240706
C	8.338252	-2.724702	3.879769
C	8.446840	-2.551304	5.402460
C	8.924755	-1.128140	5.729732
C	10.299747	-0.892668	5.085607
C	7.333919	-1.704263	3.322265
C	9.186877	-0.046028	3.004902
C	7.919258	-0.111665	5.167160
C	7.809251	-0.278888	3.643946
H	11.177591	-0.893466	3.105830
H	9.655869	-2.626700	2.154773
H	10.437140	-3.219852	3.617265
H	7.996982	-3.739840	3.648869
H	7.474017	-2.739439	5.872649
H	9.146529	-3.287414	5.816504
H	9.001960	-1.006980	6.815737
H	10.661696	0.113827	5.328353
H	11.032423	-1.599150	5.494322
H	7.230826	-1.830468	2.237701
H	6.341892	-1.877197	3.756787
H	9.529473	0.975517	3.210014

H	9.117324	-0.143689	1.914772
H	6.937201	-0.256536	5.633416
H	8.238679	0.908643	5.411920
H	7.091576	0.446238	3.244716
C	8.632727	6.373142	4.235465
C	7.152022	6.272836	4.633002
C	6.548142	4.979640	4.063857
C	6.668528	4.992715	2.532261
C	8.148008	5.089823	2.129006
C	8.747915	6.382530	2.703433
C	7.313906	3.770920	4.623669
C	9.392943	5.160900	4.795363
C	8.909896	3.881126	2.694084
C	8.795158	3.865154	4.226020
H	9.061214	7.295779	4.641975
H	7.053556	6.285726	5.725188
H	6.601817	7.142990	4.254772
H	5.492616	4.909929	4.348976
H	6.222543	4.083018	2.112256
H	6.109407	5.840259	2.117643
H	8.231381	5.099540	1.036760
H	9.799622	6.474031	2.405934
H	8.225894	7.254781	2.291546
H	7.219168	3.737358	5.715793
H	6.879613	2.839986	4.239501
H	10.456254	5.231121	4.535678
H	9.334405	5.152859	5.890474
H	8.503678	2.951826	2.276704
H	9.964334	3.927956	2.395868
H	9.338473	3.002468	4.627057
C	3.518316	-1.817431	9.669169
C	3.719217	-1.823335	8.145953
C	5.219492	-1.761345	7.819941
C	5.816134	-0.474951	8.411260
C	5.620669	-0.465274	9.935270
C	4.119393	-0.530986	10.255891
C	5.925052	-2.979105	8.436412
C	4.228012	-3.035423	10.280539
C	6.324758	-1.686538	10.546420
C	5.729512	-2.975970	9.960508
H	2.448189	-1.861441	9.899390
H	3.276276	-2.727469	7.711123
H	3.200887	-0.968160	7.695439
H	5.360393	-1.766233	6.733475
H	6.883526	-0.409098	8.167958
H	5.334616	0.403522	7.964852
H	6.046270	0.452921	10.354591
H	3.964860	-0.504745	11.341431
H	3.607814	0.346723	9.842499
H	5.522885	-3.904978	8.007625
H	6.994459	-2.956459	8.194158
H	4.074737	-3.054078	11.366429
H	3.795122	-3.961682	9.883792
H	7.401168	-1.641917	10.341070

H	6.209867	-1.680421	11.637166
H	6.233069	-3.845557	10.396970
C	-4.780899	-3.384041	8.243068
C	-5.777705	-2.386461	7.633316
C	-7.168051	-2.597778	8.252376
C	-7.639008	-4.032338	7.968324
C	-6.647038	-5.034445	8.578802
C	-5.257932	-4.816641	7.959212
C	-7.087248	-2.380627	9.771292
C	-4.706142	-3.164314	9.761974
C	-6.567118	-4.810879	10.096815
C	-6.093540	-3.378339	10.386294
H	-3.790511	-3.231860	7.800257
H	-5.438474	-1.358800	7.810987
H	-5.825924	-2.520881	6.545861
H	-7.876965	-1.885146	7.816621
H	-8.639864	-4.189625	8.388330
H	-7.720113	-4.195020	6.886745
H	-6.984494	-6.056441	8.374603
H	-4.543992	-5.539538	8.372339
H	-5.296725	-4.993604	6.877474
H	-6.771759	-1.353067	9.989063
H	-8.078355	-2.509872	10.222773
H	-3.981811	-3.857346	10.207000
H	-4.348444	-2.150446	9.978500
H	-7.548979	-4.982205	10.554433
H	-5.876880	-5.534100	10.547910
H	-6.037685	-3.220625	11.469002
C	-11.210440	-4.732615	-1.559786
C	-10.682023	-3.421114	-0.958682
C	-9.177170	-3.290235	-1.240183
C	-8.436071	-4.479092	-0.609439
C	-8.958623	-5.793563	-1.209451
C	-10.464112	-5.917861	-0.928388
C	-8.941350	-3.291797	-2.758464
C	-10.969629	-4.729964	-3.077333
C	-8.723846	-5.788671	-2.727868
C	-9.465566	-4.603153	-3.363984
H	-12.283264	-4.824053	-1.357802
H	-11.220896	-2.566881	-1.386038
H	-10.865283	-3.402845	0.122537
H	-8.802692	-2.354254	-0.811239
H	-7.357654	-4.386677	-0.786455
H	-8.579870	-4.478734	0.477840
H	-8.429283	-6.639728	-0.757950
H	-10.846123	-6.862308	-1.334655
H	-10.643861	-5.943396	0.153286
H	-9.448287	-2.435577	-3.219700
H	-7.871659	-3.179440	-2.972871
H	-11.361332	-5.652980	-3.521864
H	-11.512834	-3.898918	-3.543219
H	-7.650493	-5.719679	-2.942275
H	-9.074417	-6.731097	-3.165930
H	-9.296591	-4.600982	-4.446432

C	-4.151241	-8.249468	-1.634463
C	-2.769595	-8.011075	-2.262800
C	-2.310353	-6.572940	-1.976767
C	-3.323921	-5.586202	-2.576329
C	-4.707221	-5.818216	-1.948986
C	-5.160168	-7.258400	-2.234736
C	-2.228541	-6.357392	-0.457676
C	-4.064254	-8.029951	-0.116177
C	-4.619333	-5.605083	-0.429913
C	-3.609043	-6.591758	0.175094
H	-4.476845	-9.275120	-1.839721
H	-2.043241	-8.726040	-1.857873
H	-2.814424	-8.183244	-3.345067
H	-1.324881	-6.405382	-2.425160
H	-2.996686	-4.555054	-2.396028
H	-3.377827	-5.715912	-3.664044
H	-5.428276	-5.114063	-2.378284
H	-6.156016	-7.431258	-1.808857
H	-5.247005	-7.417668	-3.316435
H	-1.492618	-7.041943	-0.018763
H	-1.882931	-5.339444	-0.240494
H	-5.040465	-8.217283	0.347414
H	-3.360649	-8.744516	0.327778
H	-4.315260	-4.574186	-0.211718
H	-5.605730	-5.747997	0.027865
H	-3.547327	-6.437967	1.258048
C	-1.093318	-1.416083	-7.326208
C	-2.117374	-0.435410	-7.917961
C	-1.697385	1.008106	-7.600433
C	-1.628935	1.194879	-6.076990
C	-0.603673	0.218374	-5.480179
C	-1.026231	-1.223109	-5.803468
C	-0.313077	1.280934	-8.208749
C	0.289260	-1.137038	-7.935765
C	0.777793	0.492799	-6.094164
C	0.715472	0.304339	-7.617596
H	-1.394698	-2.444491	-7.553492
H	-2.190473	-0.576428	-9.003089
H	-3.112955	-0.636985	-7.504271
H	-2.428581	1.705566	-8.023740
H	-1.349306	2.227869	-5.836995
H	-2.616160	1.022218	-5.631536
H	-0.556956	0.352924	-4.394011
H	-0.312625	-1.931679	-5.365677
H	-2.002645	-1.438788	-5.352829
H	-0.352142	1.171284	-9.299294
H	-0.010231	2.315197	-8.005241
H	1.026063	-1.844599	-7.536421
H	0.259796	-1.289748	-9.021384
H	1.100039	1.513289	-5.854216
H	1.523492	-0.184921	-5.661043
H	1.701104	0.500926	-8.053637
C	1.024033	-4.113369	-1.677143
C	2.404579	-3.853513	-2.299360

C	2.849949	-2.415314	-1.992470
C	1.828403	-1.429737	-2.580287
C	0.446146	-1.683216	-1.958980
C	0.007089	-3.123410	-2.265602
C	2.926934	-2.220164	-0.470372
C	1.106158	-3.914191	-0.155785
C	0.529230	-1.490424	-0.436921
C	1.547487	-2.476044	0.156335
H	0.708325	-5.139030	-1.897272
H	3.136769	-4.567301	-1.902956
H	2.363361	-4.011023	-3.384000
H	3.834660	-2.232444	-2.436531
H	2.145738	-0.398218	-2.385033
H	1.777725	-1.544803	-3.669803
H	-0.280600	-0.979851	-2.379891
H	-0.987906	-3.311362	-1.844147
H	-0.076255	-3.268417	-3.349579
H	3.668347	-2.903926	-0.039544
H	3.262696	-1.202185	-0.238388
H	0.130863	-4.116957	0.303217
H	1.815524	-4.628331	0.279603
H	0.823336	-0.459897	-0.203827
H	-0.456652	-1.648793	0.016864
H	1.605760	-2.336762	1.241440
C	8.023174	-3.613249	-0.309843
C	6.523412	-3.715895	0.007587
C	5.944366	-4.989669	-0.627361
C	6.144158	-4.935622	-2.149731
C	7.642981	-4.836012	-2.473020
C	8.217782	-3.562847	-1.833019
C	6.674855	-6.217513	-0.062053
C	8.748103	-4.844585	0.255167
C	8.369326	-6.063954	-1.902655
C	8.175143	-6.120937	-0.379732
H	8.433923	-2.704468	0.143492
H	6.368433	-3.732158	1.093151
H	5.997313	-2.832653	-0.374588
H	4.875173	-5.061095	-0.399302
H	5.716691	-5.831002	-2.617089
H	5.610948	-4.073942	-2.569314
H	7.782987	-4.797062	-3.558805
H	9.283898	-3.469281	-2.073036
H	7.721586	-2.676755	-2.247275
H	6.523403	-6.280230	1.022366
H	6.257112	-7.135163	-0.493613
H	9.823752	-4.773385	0.052915
H	8.632756	-4.882079	1.345143
H	7.981351	-6.979226	-2.365871
H	9.438015	-6.015037	-2.144474
H	8.693209	-6.997295	0.025058
C	-6.148107	3.530203	-6.874057
C	-5.389275	2.322122	-6.303709
C	-5.914609	1.028778	-6.945767
C	-7.413755	0.882694	-6.642997

C	-8.178091	2.087088	-7.214077
C	-7.646701	3.377765	-6.571641
C	-5.707261	1.094940	-8.466760
C	-5.939597	3.589899	-8.395210
C	-7.964673	2.150939	-8.734310
C	-6.466991	2.300156	-9.042415
H	-5.772256	4.451082	-6.414897
H	-4.314553	2.427972	-6.494763
H	-5.515060	2.279451	-5.214990
H	-5.371820	0.168852	-6.538098
H	-7.796584	-0.047786	-7.079467
H	-7.574512	0.814408	-5.560246
H	-9.246504	1.981081	-6.996582
H	-8.198041	4.244343	-6.956391
H	-7.811933	3.353893	-5.487617
H	-4.638526	1.178619	-8.698364
H	-6.061046	0.168455	-8.935050
H	-6.459979	4.460701	-8.812227
H	-4.874770	3.717734	-8.624469
H	-8.357499	1.242985	-9.207728
H	-8.522177	2.995384	-9.157474
H	-6.316728	2.344966	-10.126695
C	8.009276	1.750334	-2.048329
C	7.594286	0.307157	-2.373301
C	6.225629	0.006129	-1.743130
C	6.316672	0.183228	-0.219715
C	6.728482	1.626099	0.111106
C	8.095663	1.923129	-0.524139
C	5.180310	0.981072	-2.306677
C	6.959373	2.720574	-2.611611
C	5.682209	2.597034	-0.457518
C	5.589476	2.426039	-1.981463
H	8.985253	1.962784	-2.498267
H	7.548512	0.163954	-3.459638
H	8.346159	-0.394664	-1.992396
H	5.931495	-1.023085	-1.976564
H	5.350244	-0.048447	0.244183
H	7.045876	-0.521177	0.198530
H	6.793285	1.749891	1.197626
H	8.410764	2.944623	-0.278729
H	8.856531	1.249797	-0.111001
H	5.089861	0.851083	-3.391923
H	4.194247	0.763748	-1.878563
H	7.254709	3.756263	-2.403952
H	6.901191	2.620602	-3.702197
H	4.704650	2.408170	0.002403
H	5.954196	3.630427	-0.210362
H	4.842700	3.118660	-2.385013
C	-6.793338	-8.457822	4.417783
C	-8.299581	-8.571097	4.698822
C	-9.036404	-7.379257	4.068297
C	-8.498879	-6.072083	4.670633
C	-6.993019	-5.952155	4.389723
C	-6.261458	-7.147849	5.019073

C	-8.795183	-7.376718	2.550866
C	-6.558188	-8.452973	2.899354
C	-6.756833	-5.953715	2.871518
C	-7.289975	-7.260486	2.264596
H	-6.269951	-9.308246	4.868289
H	-8.688818	-9.512398	4.292399
H	-8.479086	-8.594546	5.780571
H	-10.110149	-7.462372	4.269419
H	-9.031385	-5.214028	4.242805
H	-8.682450	-6.051967	5.751722
H	-6.611880	-5.019835	4.820491
H	-5.182187	-7.064030	4.842682
H	-6.405044	-7.146604	6.106437
H	-9.192984	-8.296028	2.104258
H	-9.332260	-6.540953	2.086237
H	-5.484125	-8.393126	2.685338
H	-6.916697	-9.391657	2.459919
H	-7.258333	-5.093405	2.411972
H	-5.686407	-5.848074	2.657060
H	-7.121208	-7.259567	1.182114
C	-5.775635	4.433859	-2.701015
C	-6.792025	5.411687	-3.310431
C	-6.362331	6.857314	-3.016358
C	-6.294537	7.068923	-1.496137
C	-5.276919	6.095339	-0.881731
C	-5.709158	4.651665	-1.181602
C	-4.975361	7.110299	-3.627181
C	-4.390349	4.693052	-3.313183
C	-3.892772	6.349846	-1.498252
C	-3.954450	6.136508	-3.018426
H	-6.083926	3.403944	-2.911592
H	-6.864711	5.253150	-4.393165
H	-7.789526	5.223986	-2.894846
H	-7.088072	7.552729	-3.452231
H	-6.007980	8.103782	-1.272957
H	-7.283522	6.910602	-1.049262
H	-5.230660	6.247610	0.202114
H	-5.001105	3.945505	-0.731087
H	-6.687642	4.450344	-0.728811
H	-5.013788	6.982798	-4.715805
H	-4.665532	8.145663	-3.440472
H	-3.659042	3.987099	-2.901095
H	-4.419484	4.522512	-4.396154
H	-3.563686	7.371909	-1.274852
H	-3.152403	5.674229	-1.052875
H	-2.966901	6.318918	-3.456289
C	-6.256281	-5.542409	-7.291266
C	-4.863378	-5.276868	-7.882785
C	-4.437092	-3.832261	-7.578905
C	-5.452190	-2.860641	-8.200177
C	-6.846833	-3.119828	-7.609668
C	-7.266798	-4.566375	-7.913111
C	-4.398137	-3.622460	-6.057309
C	-6.212202	-5.328545	-5.770324

C	-6.801761	-2.912336	-6.087942
C	-5.790081	-3.883975	-5.461312
H	-6.558349	-6.572636	-7.509202
H	-4.135154	-5.980881	-7.462325
H	-4.877351	-5.444703	-8.966569
H	-3.443542	-3.645337	-8.001048
H	-5.147947	-1.824773	-8.007124
H	-5.475846	-2.986159	-9.289462
H	-7.568944	-2.426418	-8.054400
H	-8.270047	-4.758674	-7.513766
H	-7.323001	-4.722052	-8.997355
H	-3.661759	-4.296056	-5.602498
H	-4.076243	-2.599640	-5.826970
H	-7.196537	-5.535147	-5.332834
H	-5.507725	-6.032740	-5.311433
H	-6.521638	-1.877302	-5.857606
H	-7.796913	-3.074679	-5.656338
H	-5.758921	-3.734221	-4.376483
C	-0.783185	5.155734	-6.983996
C	-0.571009	5.225534	-8.504092
C	-1.331803	6.430036	-9.079697
C	-2.830349	6.277383	-8.777200
C	-3.048634	6.210167	-7.257746
C	-2.283129	5.006469	-6.686730
C	-0.810464	7.720076	-8.427934
C	-0.262751	6.449031	-6.337869
C	-2.522536	7.500505	-6.610457
C	-1.023290	7.656329	-6.907489
H	-0.240220	4.296311	-6.575717
H	0.498364	5.311724	-8.731565
H	-0.921710	4.299235	-8.975190
H	-1.178662	6.478175	-10.163448
H	-3.387383	7.121861	-9.200789
H	-3.220289	5.369714	-9.253579
H	-4.117559	6.100799	-7.044469
H	-2.446777	4.935314	-5.604546
H	-2.663633	4.076183	-7.125777
H	0.254657	7.852625	-8.653290
H	-1.332843	8.589602	-8.844922
H	-0.389806	6.403151	-5.249408
H	0.812035	6.558137	-6.526787
H	-3.074339	8.366540	-6.995570
H	-2.691022	7.473829	-5.527006
H	-0.650101	8.577048	-6.445693
C	5.019084	-5.170830	3.605175
C	4.551127	-6.604730	3.312721
C	3.172055	-6.840492	3.947959
C	3.269050	-6.632123	5.467045
C	3.733815	-5.198277	5.765351
C	5.111267	-4.966571	5.125128
C	2.163699	-5.840710	3.361066
C	4.005974	-4.175571	3.018649
C	2.724360	-4.202580	5.173472
C	2.625902	-4.404800	3.653715

H	6.002407	-5.004931	3.151674
H	4.500470	-6.770923	2.229879
H	5.276401	-7.324975	3.710438
H	2.840146	-7.863196	3.737718
H	2.294554	-6.817263	5.934954
H	3.971562	-7.353259	5.902124
H	3.802771	-5.052176	6.848839
H	5.463839	-3.952081	5.347543
H	5.846573	-5.657950	5.554430
H	2.068885	-5.992026	2.278962
H	1.170124	-6.011604	3.792849
H	4.339331	-3.147031	3.202975
H	3.944516	-4.298212	1.930558
H	1.740325	-4.344629	5.636414
H	3.034286	-3.174569	5.397310
H	1.905389	-3.694453	3.233576
C	2.885090	-7.729121	-0.224791
C	1.379713	-7.844559	0.059985
C	0.830413	-9.135723	-0.566210
C	1.064605	-9.104824	-2.084316
C	2.569213	-8.992531	-2.374919
C	3.114161	-7.701943	-1.743760
C	1.562001	-10.344979	0.036508
C	3.611167	-8.941940	0.377561
C	3.296543	-10.201849	-1.767239
C	3.067981	-10.235607	-0.248402
H	3.274610	-6.807957	0.222244
H	1.199949	-7.844473	1.141843
H	0.852260	-6.974286	-0.349124
H	-0.242844	-9.216240	-0.361459
H	0.658539	-10.013028	-2.545979
H	0.531158	-8.256821	-2.530613
H	2.733749	-8.970120	-3.457727
H	4.184358	-7.599528	-1.960911
H	2.617306	-6.828996	-2.184303
H	1.386333	-10.391313	1.118095
H	1.165092	-11.274729	-0.388769
H	4.690286	-8.861138	0.198759
H	3.471168	-8.962526	1.465097
H	2.930094	-11.129394	-2.223521
H	4.369877	-10.144074	-1.985371
H	3.586801	-11.098716	0.183013
C	0.401932	0.735770	8.219648
C	-0.620704	1.729895	7.648234
C	-1.996242	1.484731	8.287566
C	-2.450593	0.049070	7.982785
C	-1.432710	-0.949671	8.554919
C	-0.058521	-0.698029	7.915212
C	-1.891166	1.672523	9.808909
C	0.500924	0.926044	9.741142
C	-1.328631	-0.755461	10.075506
C	-0.871530	0.678112	10.385624
H	1.381713	0.912098	7.762432
H	-0.293838	2.758892	7.840731

H	-0.686621	1.616636	6.559299
H	-2.723612	2.194948	7.879141
H	-3.441176	-0.132066	8.417289
H	-2.548854	-0.093067	6.899736
H	-1.758369	-1.972444	8.335989
H	0.673619	-1.417975	8.300719
H	-0.114269	-0.853787	6.830957
H	-1.587306	2.700246	10.041912
H	-2.871855	1.518937	10.275272
H	1.243579	0.235472	10.158958
H	0.847165	1.940756	9.971868
H	-2.299330	-0.951094	10.546982
H	-0.619493	-1.476861	10.499383
H	-0.798417	0.814870	11.470152
C	1.242779	10.261353	-3.057922
C	0.812500	8.821281	-3.376726
C	-0.548583	8.530000	-2.725910
C	-0.433178	8.706100	-1.204030
C	-0.005991	10.145887	-0.879329
C	1.353510	10.433181	-1.535197
C	-1.595259	9.512588	-3.273432
C	0.191490	11.239271	-3.605110
C	-1.053727	11.124475	-1.431914
C	-1.170786	10.954492	-2.954297
H	2.213331	10.466853	-3.522582
H	0.749228	8.678654	-4.462261
H	1.564974	8.113967	-3.007344
H	-0.853646	7.502988	-2.954972
H	-1.394108	8.481303	-0.725557
H	0.297179	7.996356	-0.796969
H	0.076170	10.268967	0.206097
H	1.679664	11.452320	-1.294478
H	2.115673	9.754284	-1.133719
H	-1.703088	9.383496	-4.357196
H	-2.576262	9.302292	-2.830440
H	0.497411	12.272755	-3.401837
H	0.116059	11.139963	-4.694700
H	-2.025539	10.942569	-0.957244
H	-0.770570	12.155823	-1.188794
H	-1.918574	11.652574	-3.346399
C	-6.954160	-0.669444	2.616378
C	-7.398378	-2.111991	2.329476
C	-8.784484	-2.360358	2.944274
C	-8.715202	-2.137142	4.462768
C	-8.274269	-0.694640	4.755468
C	-6.889648	-0.450395	4.135698
C	-9.796106	-1.379344	2.331883
C	-7.970482	0.306988	2.004318
C	-9.286855	0.282230	4.138111
C	-9.357670	0.065138	2.618867
H	-5.965784	-0.494562	2.177472
H	-7.429054	-2.288519	1.247520
H	-6.670339	-2.818954	2.745548
H	-9.099435	-3.389218	2.738024

H	-9.694737	-2.331016	4.916398
H	-8.010521	-2.844965	4.915831
H	-8.225046	-0.537952	5.838553
H	-6.554131	0.570637	4.354532
H	-6.152478	-1.128080	4.583248
H	-9.871136	-1.541576	1.249807
H	-10.794310	-1.559550	2.748953
H	-7.653734	1.341471	2.184627
H	-8.012441	0.173815	0.916538
H	-10.276411	0.131271	4.586227
H	-8.994175	1.316227	4.357549
H	-10.080451	0.762068	2.180545
C	-10.941645	0.320289	-2.674467
C	-9.554275	0.566887	-3.286820
C	-9.115535	2.013719	-3.012709
C	-10.131965	2.981054	-3.638473
C	-11.521049	2.740803	-3.027218
C	-11.953474	1.291999	-3.300934
C	-9.057189	2.249116	-1.495483
C	-10.878159	0.559706	-1.158025
C	-11.456599	2.973856	-1.509896
C	-10.443523	2.006614	-0.878733
H	-11.252610	-0.711558	-2.871196
H	-8.825438	-0.134481	-2.863013
H	-9.582174	0.380735	-4.367340
H	-8.125948	2.187127	-3.449669
H	-9.819088	4.018104	-3.466743
H	-10.169351	2.837181	-4.725100
H	-12.244126	3.431117	-3.475183
H	-12.953064	1.112891	-2.886559
H	-12.023530	1.118264	-4.381622
H	-8.319604	1.578694	-1.037948
H	-8.726299	3.273643	-1.286461
H	-11.858469	0.366824	-0.705493
H	-10.172611	-0.141039	-0.695513
H	-11.167401	4.010858	-1.300592
H	-12.447524	2.825195	-1.063876
H	-10.398538	2.174596	0.202936
C	-3.954845	0.828295	-1.263681
C	-5.454564	0.713790	-0.950128
C	-6.016019	-0.578270	-1.563563
C	-5.810737	-0.550017	-3.085900
C	-4.311898	-0.438667	-3.405258
C	-3.754700	0.852827	-2.786776
C	-5.273423	-1.786720	-0.972811
C	-3.217766	-0.383717	-0.673184
C	-3.573464	-1.647170	-2.809464
C	-3.773105	-1.678282	-1.286499
H	-3.556647	1.750100	-0.825704
H	-5.613719	0.715767	0.134952
H	-5.989471	1.583526	-1.350612
H	-7.085209	-0.658118	-1.338300
H	-6.225752	-1.458882	-3.538209
H	-6.352350	0.297387	-3.523412

H	-4.167981	-0.418144	-4.491036
H	-2.688799	0.954553	-3.024417
H	-4.259611	1.725174	-3.219274
H	-5.428499	-1.831165	0.111998
H	-5.678597	-2.717072	-1.388879
H	-2.142221	-0.303542	-0.872616
H	-3.337060	-0.402416	0.416852
H	-3.948773	-2.575379	-3.257113
H	-2.504458	-1.590086	-3.048069
H	-3.246403	-2.540813	-0.863561
C	0.833766	3.672247	-3.429250
C	1.393790	4.966547	-2.819544
C	1.203849	4.946786	-1.294917
C	-0.293402	4.832386	-0.969956
C	-0.858093	3.537761	-1.574916
C	-0.662522	3.560932	-3.098780
C	1.945879	3.737487	-0.705191
C	1.577263	2.466425	-2.834143
C	-0.110575	2.332006	-0.984910
C	1.387093	2.440064	-1.309809
H	0.969958	3.688470	-4.516126
H	2.457652	5.069117	-3.065324
H	0.883467	5.836344	-3.250794
H	1.604327	5.870222	-0.862285
H	-0.443840	4.837434	0.116414
H	-0.832792	5.700157	-1.368561
H	-1.925683	3.458583	-1.342472
H	-1.079616	2.650278	-3.545665
H	-1.208789	4.405977	-3.535207
H	3.020025	3.816588	-0.912047
H	1.833817	3.723074	0.385712
H	1.200475	1.536384	-3.276920
H	2.644684	2.523964	-3.079216
H	-0.258035	2.292743	0.101152
H	-0.518329	1.399547	-1.393838
H	1.918466	1.579883	-0.887930
C	-1.765567	3.417263	2.590368
C	-2.212576	1.978863	2.287431
C	-3.593609	1.722351	2.910249
C	-3.511669	1.924733	4.431030
C	-3.067897	3.363009	4.739791
C	-1.688392	3.615469	4.111958
C	-4.609985	2.711846	2.319765
C	-2.786641	4.402173	2.000191
C	-4.085279	4.348429	4.144294
C	-4.168756	4.152180	2.622859
H	-0.780813	3.597961	2.145713
H	-2.252276	1.817178	1.203450
H	-1.481321	1.266134	2.687726
H	-3.910564	0.696470	2.692546
H	-4.487465	1.724841	4.890074
H	-2.803465	1.210641	4.868494
H	-3.009647	3.504837	5.824475
H	-1.350769	4.633345	4.341964

H	-0.947735	2.931582	4.544052
H	-4.694034	2.564465	1.236229
H	-5.604749	2.526125	2.742596
H	-2.468102	5.434032	2.192019
H	-2.837660	4.283921	0.911076
H	-5.071130	4.191525	4.598495
H	-3.790483	5.379270	4.375438
H	-4.894941	4.855181	2.200136
C	-2.140570	-3.133985	2.228869
C	-1.580276	-1.841565	2.842307
C	-1.780936	-1.860423	4.365571
C	-3.280814	-1.969513	4.680074
C	-3.845809	-3.262240	4.071351
C	-3.639512	-3.239992	2.548888
C	-1.047299	-3.072231	4.960627
C	-1.405492	-4.342319	2.829315
C	-3.106674	-4.470522	4.666723
C	-1.606401	-4.367777	4.352286
H	-1.996729	-3.118408	1.142970
H	-0.514368	-1.742771	2.603955
H	-2.084513	-0.970048	2.407387
H	-1.380249	-0.938333	4.800872
H	-3.438822	-1.963769	5.765366
H	-3.814353	-1.099914	4.277596
H	-4.915269	-3.337632	4.296338
H	-4.056671	-4.149244	2.099217
H	-4.179744	-2.393100	2.108543
H	0.028538	-2.996936	4.761273
H	-1.167033	-3.086082	6.050721
H	-1.782442	-5.271099	2.384036
H	-0.336189	-4.288567	2.591701
H	-3.261858	-4.509100	5.751733
H	-3.514834	-5.401607	4.255077
H	-1.081016	-5.229753	4.777981
C	3.777314	-2.307735	-6.215515
C	4.362306	-1.033376	-5.587522
C	5.862517	-0.939181	-5.906032
C	6.582507	-2.170118	-5.334319
C	6.003311	-3.447659	-5.961477
C	4.502659	-3.535245	-5.643176
C	6.057791	-0.898051	-7.429505
C	3.977726	-2.262573	-7.738257
C	6.197741	-3.400046	-7.484850
C	5.476883	-2.172250	-8.062021
H	2.707892	-2.373065	-5.987088
H	3.839292	-0.149563	-5.972312
H	4.208470	-1.045059	-4.501635
H	6.277372	-0.029625	-5.457826
H	7.658210	-2.104803	-5.537922
H	6.467700	-2.201095	-4.244037
H	6.517693	-4.323915	-5.552044
H	4.080614	-4.453774	-6.068790
H	4.350972	-3.590811	-4.558334
H	5.565849	-0.012113	-7.848960

H	7.124407	-0.811321	-7.669588
H	3.545652	-3.158141	-8.201147
H	3.448635	-1.400526	-8.162164
H	7.266659	-3.356748	-7.726422
H	5.806329	-4.316469	-7.943031
H	5.617053	-2.139671	-9.148088
C	0.721976	-5.050146	-5.819930
C	-0.783320	-5.156956	-5.531380
C	-1.339662	-6.449732	-6.147955
C	-1.108426	-6.429868	-7.666699
C	0.396075	-6.326237	-7.961053
C	0.948073	-5.033985	-7.339501
C	-0.612264	-7.658268	-5.538757
C	1.443847	-6.262223	-5.211062
C	1.119225	-7.534784	-7.346884
C	0.893607	-7.557528	-5.827402
H	1.116521	-4.127819	-5.379753
H	-0.960879	-5.148949	-4.449187
H	-1.307707	-4.287034	-5.945152
H	-2.412849	-6.524086	-5.940526
H	-1.519488	-7.339258	-8.121556
H	-1.638982	-5.582434	-8.117499
H	0.558505	-6.311686	-9.044313
H	2.018273	-4.937802	-7.559469
H	0.454232	-4.161733	-7.784784
H	-0.785935	-7.696700	-4.456538
H	-1.014192	-8.589005	-5.957105
H	2.522950	-6.187439	-5.392557
H	1.305972	-6.275028	-4.123135
H	0.747703	-8.463657	-7.796312
H	2.192360	-7.483260	-7.567544
H	1.409440	-8.420100	-5.391355
C	3.403213	2.236638	4.331401
C	1.904519	2.132261	4.653280
C	1.332134	0.841327	4.047626
C	1.529519	0.862734	2.524133
C	3.027283	0.963941	2.196466
C	3.595435	2.254354	2.807192
C	2.070632	-0.369355	4.638947
C	4.136172	1.022427	4.922510
C	3.761645	-0.246749	2.792851
C	3.569888	-0.271048	4.316953
H	3.809210	3.157656	4.763839
H	1.751126	2.139192	5.739169
H	1.372693	3.003696	4.252387
H	0.263695	0.768705	4.278818
H	1.106706	-0.045296	2.077403
H	0.990658	1.711741	2.086332
H	3.165570	0.979598	1.109875
H	4.660650	2.348827	2.563558
H	3.093449	3.128102	2.374118
H	1.921039	-0.408869	5.724717
H	1.657724	-1.298796	4.228486
H	5.211104	1.095434	4.717114

H	4.022541	1.008465	6.013222
H	3.378447	-1.174342	2.350644
H	4.829695	-0.196939	2.548403
H	4.093672	-1.135146	4.740314

Adamantane$_{38}$

988
E=-1880.2854

C	5.858781	4.447418	-7.652563
C	5.452391	2.999365	-7.966528
C	4.075656	2.701785	-7.352525
C	4.144364	2.896899	-5.830140
C	4.547491	4.344705	-5.510368
C	5.922835	4.638224	-6.129419
C	3.035898	3.667088	-7.942341
C	4.814449	5.408008	-8.242124
C	3.506863	5.305938	-6.105228
C	3.436456	5.116917	-7.628238
H	6.840533	4.657403	-8.090946
H	5.422587	2.843372	-9.051659
H	6.200616	2.304193	-7.566717
H	3.787679	1.669080	-7.578083
H	3.172013	2.667881	-5.377437
H	4.869395	2.199513	-5.393290
H	4.596373	4.481345	-4.424556
H	6.231626	5.663410	-5.891497
H	6.679515	3.971940	-5.697586
H	2.961372	3.524193	-9.027174
H	2.044387	3.451946	-7.525865
H	5.103979	6.446891	-8.042396
H	4.772182	5.295158	-9.332187
H	2.523323	5.119649	-5.657161
H	3.772488	6.342919	-5.866315
H	2.693677	5.802640	-8.050509
C	-1.642660	-5.911765	9.733768
C	-1.443941	-5.930142	8.210365
C	0.055697	-5.867057	7.881641
C	0.649848	-4.574128	8.461096
C	0.456556	-4.551957	9.985253
C	-1.044081	-4.618812	10.308620
C	0.765305	-7.077687	8.507439
C	-0.928922	-7.122659	10.354461
C	1.164695	-5.766140	10.605761
C	0.571955	-7.062057	10.031737
H	-2.712336	-5.956575	9.965923
H	-1.885159	-6.839093	7.783909
H	-1.965139	-5.080184	7.753336
H	0.195043	-5.880848	6.795050
H	1.716713	-4.507594	8.215679
H	0.165406	-3.700743	8.007920
H	0.880375	-3.629122	10.396107
H	-1.197116	-4.583713	11.394122
H	-1.558533	-3.745986	9.888509

H	0.364927	-8.008220	8.087150
H	1.834300	-7.054344	8.263434
H	-1.080582	-7.132447	11.440693
H	-1.359976	-8.053385	9.966260
H	2.240688	-5.720490	10.398463
H	1.051361	-5.751017	11.696583
H	1.078398	-7.926585	10.474860
C	-2.012843	-10.932989	3.998834
C	-0.637954	-10.709941	3.350362
C	-0.159146	-9.276648	3.628516
C	-1.170602	-8.279442	3.042866
C	-2.547089	-8.496095	3.690362
C	-3.019620	-9.931504	3.412402
C	-0.053022	-9.060965	5.146083
C	-1.901512	-10.713393	5.515518
C	-2.434915	-8.282892	5.207825
C	-1.426659	-9.279996	5.798967
H	-2.352419	-11.955208	3.799168
H	0.086443	-11.432424	3.745329
H	-0.700307	-10.882351	2.269002
H	0.821476	-9.120035	3.165764
H	-0.829696	-7.251762	3.217550
H	-1.241651	-8.409302	1.956154
H	-3.266657	-7.784497	3.270959
H	-4.011000	-10.093321	3.852796
H	-3.123827	-10.090560	2.332207
H	0.681762	-9.753115	5.574868
H	0.306646	-8.046656	5.357388
H	-2.872867	-10.889869	5.993349
H	-1.199291	-11.435203	5.949837
H	-2.116622	-7.255187	5.420734
H	-3.416061	-8.414847	5.679953
H	-1.347609	-9.126144	6.880785
C	3.393651	7.551070	2.575453
C	2.945139	6.115789	2.260194
C	1.572532	5.849070	2.897191
C	1.673439	6.032546	4.419230
C	2.118788	7.467625	4.740352
C	3.489810	7.730351	4.098309
C	0.547162	6.844087	2.332082
C	2.363595	8.541489	2.010653
C	1.092354	8.458628	4.170192
C	0.989893	8.281290	2.647596
H	4.372393	7.739052	2.120660
H	2.891947	5.967614	1.174861
H	3.682555	5.399394	2.642226
H	1.254485	4.825438	2.670676
H	0.703867	5.825208	4.888056
H	2.388279	5.314283	4.838701
H	2.190569	7.595968	5.825904
H	3.828693	8.745862	4.336764
H	4.236984	7.042415	4.512378
H	0.449607	6.710132	1.247935
H	-0.441858	6.651338	2.765118

H	2.682889	9.571426	2.211352
H	2.298950	8.436789	0.920875
H	0.112599	8.294316	4.634842
H	1.388408	9.487012	4.410489
H	0.257278	8.988247	2.242952
C	-1.226581	8.144139	8.925972
C	-1.010866	8.156632	7.404823
C	-1.586450	6.872286	6.788293
C	-0.877018	5.653112	7.397201
C	-1.092169	5.633979	8.918467
C	-0.518216	6.921669	9.529513
C	-3.090039	6.790551	7.093794
C	-2.731238	8.059565	9.225643
C	-2.596998	5.555876	9.218674
C	-3.310809	6.774697	8.614360
H	-0.815369	9.060423	9.363509
H	-1.494572	9.036161	6.963021
H	0.059264	8.235833	7.177880
H	-1.432057	6.883596	5.703709
H	-1.265448	4.729988	6.950187
H	0.195253	5.688612	7.169542
H	-0.585105	4.764323	9.350477
H	-0.647696	6.911694	10.618596
H	0.560541	6.979491	9.339986
H	-3.612641	7.644931	6.646852
H	-3.516842	5.886861	6.642112
H	-2.900052	8.070593	10.309330
H	-3.246521	8.936770	8.816066
H	-3.015727	4.630893	8.804078
H	-2.763283	5.521034	10.302233
H	-4.383601	6.717017	8.828927
C	-6.351176	4.051591	8.963270
C	-7.853636	3.968481	9.273578
C	-8.439951	2.686684	8.661871
C	-8.230643	2.707087	7.139957
C	-6.729114	2.787047	6.823850
C	-6.147079	4.068377	7.440595
C	-7.723544	1.464746	9.257202
C	-5.640357	2.826204	9.558342
C	-6.016882	1.565154	7.424212
C	-6.220609	1.541778	8.946774
H	-5.935243	4.966123	9.400009
H	-8.015205	3.976310	10.358275
H	-8.369750	4.847992	8.869798
H	-9.511062	2.629222	8.884817
H	-8.663093	1.805801	6.688816
H	-8.753833	3.564319	6.699185
H	-6.582318	2.802023	5.738366
H	-5.078781	4.147750	7.205213
H	-6.633077	4.949810	7.004865
H	-7.881983	1.426079	10.341746
H	-8.146677	0.541864	8.842375
H	-4.562945	2.883926	9.361241
H	-5.762508	2.812561	10.648136

H	-6.410040	0.643729	6.977870
H	-4.946388	1.599829	7.187978
H	-5.712602	0.669692	9.372972
C	5.576823	4.851548	8.175360
C	5.127589	3.417138	7.857178
C	3.761959	3.144703	8.506628
C	3.877956	3.320448	10.028504
C	4.324087	4.754620	10.352580
C	5.688088	5.023086	9.698079
C	2.729225	4.140838	7.957113
C	4.539414	5.843076	7.626178
C	3.290246	5.746765	9.798004
C	3.172677	5.577159	8.275618
H	6.550588	5.043610	7.711676
H	5.063664	3.274509	6.771681
H	5.870056	2.700066	8.227993
H	3.443380	2.121704	8.278010
H	2.913535	3.108973	10.506026
H	4.598230	2.601273	10.436973
H	4.406636	4.877449	11.438004
H	6.027637	6.037939	9.938383
H	6.440588	4.334322	10.100979
H	2.620927	4.012345	6.873324
H	1.744970	3.944105	8.399121
H	4.858968	6.872516	7.829001
H	4.463918	5.743924	6.536578
H	2.315526	5.578321	10.271678
H	3.586964	6.774406	10.040650
H	2.434799	6.284920	7.882096
C	-3.483469	10.772341	3.359230
C	-4.985698	10.649387	3.657180
C	-5.537353	9.363523	3.022211
C	-5.317897	9.411665	1.502353
C	-3.816484	9.531537	1.198551
C	-3.269131	10.816758	1.838518
C	-4.796569	8.149917	3.604612
C	-2.748189	9.555161	3.941279
C	-3.079910	8.317803	1.785953
C	-3.293722	8.266810	3.306456
H	-3.092265	11.689714	3.812547
H	-5.155023	10.637146	4.740652
H	-5.519531	11.522518	3.262699
H	-6.608354	9.277628	3.236373
H	-5.725823	8.507363	1.034710
H	-5.858020	10.262916	1.070503
H	-3.662459	9.566239	0.114521
H	-2.201371	10.924676	1.612226
H	-3.772678	11.692996	1.412342
H	-4.961670	8.091274	4.687266
H	-5.194928	7.223700	3.173027
H	-1.671077	9.641075	3.753009
H	-2.877637	9.522304	5.029820
H	-3.448115	7.394218	1.323098
H	-2.008897	8.381103	1.558162

H	-2.768326	7.400573	3.723402
C	-8.956538	5.452674	1.237203
C	-8.401914	6.732572	1.881281
C	-8.609982	6.680278	3.402770
C	-10.111152	6.561462	3.707429
C	-10.670500	5.281145	3.068034
C	-10.456806	5.336822	1.547448
C	-7.876770	5.457281	3.975019
C	-8.221918	4.233035	1.814923
C	-9.931799	4.061676	3.640703
C	-8.430221	4.174149	3.335975
H	-8.807420	5.492088	0.152620
H	-7.335059	6.838596	1.650390
H	-8.905812	7.612334	1.462880
H	-8.213329	7.593488	3.859936
H	-10.274478	6.543350	4.791796
H	-10.644507	7.438544	3.321275
H	-11.740890	5.198801	3.286053
H	-10.869886	4.436726	1.076112
H	-10.996620	6.192011	1.122912
H	-6.800128	5.538985	3.782655
H	-8.001805	5.419549	5.063952
H	-8.594772	3.313400	1.347753
H	-7.151578	4.294018	1.583810
H	-10.092207	3.999264	4.723843
H	-10.336022	3.138946	3.206953
H	-7.905149	3.304168	3.745464
C	10.192707	-1.051771	3.546536
C	9.718484	-2.478282	3.229106
C	8.343853	-2.723172	3.870489
C	8.453484	-2.545626	5.392628
C	8.924563	-1.119279	5.715933
C	10.297522	-0.878339	5.069498
C	7.333634	-1.709177	3.311866
C	9.177724	-0.042292	2.988227
C	7.913193	-0.109265	5.152252
C	7.802111	-0.280646	3.629578
H	11.172852	-0.879351	3.088609
H	9.658770	-2.622547	2.143586
H	10.444900	-3.208258	3.606472
H	8.007462	-3.740577	3.642420
H	7.482227	-2.737595	5.864502
H	9.157434	-3.277187	5.807512
H	9.002524	-0.995162	6.801550
H	10.654650	0.130555	5.309409
H	11.034301	-1.580117	5.478941
H	7.229812	-1.838468	2.227734
H	6.343051	-1.886127	3.748062
H	9.515375	0.981465	3.190493
H	9.107289	-0.142882	1.898420
H	6.932477	-0.258028	5.620102
H	8.227726	0.913230	5.394197
H	7.080250	0.439874	3.229557
C	8.636234	6.368195	4.281563

C	7.145053	6.274090	4.639484
C	6.543106	5.000732	4.025340
C	6.702246	5.047965	2.497960
C	8.192316	5.139017	2.134219
C	8.790153	6.411806	2.753530
C	7.282623	3.771513	4.575727
C	9.370168	5.135512	4.831815
C	8.927826	3.909741	2.689772
C	8.774294	3.859546	4.217566
H	9.063332	7.276692	4.720118
H	7.019173	6.262746	5.728868
H	6.613117	7.158416	4.268051
H	5.480086	4.935401	4.282259
H	6.258125	4.152896	2.045781
H	6.162095	5.910622	2.089408
H	8.303331	5.173119	1.044925
H	9.849882	6.499246	2.484652
H	8.287262	7.298714	2.349214
H	7.160016	3.713695	5.664057
H	6.849076	2.854266	4.159162
H	10.440333	5.200684	4.600521
H	9.283922	5.102761	5.924631
H	8.523198	2.994603	2.240754
H	9.989865	3.952513	2.419202
H	9.298839	2.982229	4.611796
C	3.525919	-1.817503	9.664531
C	3.723972	-1.824470	8.140947
C	5.223592	-1.761711	7.812077
C	5.820457	-0.474443	8.401265
C	5.627839	-0.463697	9.925630
C	4.127211	-0.530184	10.249114
C	5.931139	-2.978501	8.428184
C	4.237594	-3.034527	10.275530
C	6.333909	-1.683998	10.536421
C	5.738451	-2.974298	9.952640
H	2.456256	-1.862064	9.896791
H	3.280838	-2.729249	7.707658
H	3.204213	-0.970006	7.690731
H	5.362461	-1.767358	6.725353
H	6.887346	-0.408051	8.155913
H	5.337501	0.403348	7.955068
H	6.053594	0.455119	10.343431
H	3.974692	-0.503194	11.334920
H	3.614257	0.346848	9.835990
H	5.528806	-3.904986	8.000880
H	7.000076	-2.955314	8.183910
H	4.086365	-3.052431	11.361718
H	3.804598	-3.961395	9.880323
H	7.409902	-1.638802	10.329022
H	6.221055	-1.677100	11.627376
H	6.243420	-3.843196	10.388843
C	-4.773321	-3.380539	8.242751
C	-5.771430	-2.383654	7.633995
C	-7.161098	-2.596205	8.254154

C	-7.631129	-4.031093	7.970228
C	-6.637852	-5.032510	8.579712
C	-5.249429	-4.813472	7.959026
C	-7.079227	-2.379263	9.773043
C	-4.697498	-3.161025	9.761635
C	-6.556869	-4.809153	10.097700
C	-6.084211	-3.376283	10.387052
H	-3.783418	-3.227478	7.799157
H	-5.432883	-1.355752	7.811575
H	-5.820430	-2.517917	6.546556
H	-7.870944	-1.884067	7.819108
H	-8.631513	-4.189265	8.391024
H	-7.712988	-4.193646	6.888687
H	-6.974650	-6.054741	8.375605
H	-4.534568	-5.535867	8.371438
H	-5.288965	-4.990272	6.877287
H	-6.764390	-1.351488	9.990742
H	-8.069860	-2.509390	10.225311
H	-3.972244	-3.853553	10.205943
H	-4.340441	-2.146908	9.978051
H	-7.538217	-4.981354	10.556090
H	-5.865679	-5.531898	10.548100
H	-6.027598	-3.218720	11.469742
C	-11.209394	-4.735487	-1.555342
C	-10.681371	-3.423214	-0.955581
C	-9.176871	-3.291436	-1.238547
C	-8.434338	-4.479347	-0.607705
C	-8.956494	-5.794586	-1.206377
C	-10.461635	-5.919782	-0.923852
C	-8.942441	-3.293823	-2.757042
C	-10.969975	-4.733658	-3.073111
C	-8.723111	-5.790520	-2.725011
C	-9.466267	-4.605953	-3.361223
H	-12.281966	-4.827564	-1.352315
H	-11.221249	-2.569648	-1.383003
H	-10.863654	-3.404367	0.125793
H	-8.802674	-2.354906	-0.810559
H	-7.356151	-4.386272	-0.785769
H	-8.577142	-4.478380	0.479706
H	-8.426132	-6.640074	-0.754807
H	-10.843338	-6.864770	-1.329151
H	-10.640375	-5.944738	0.158002
H	-9.450416	-2.438271	-3.218374
H	-7.873028	-3.180838	-2.972503
H	-11.361421	-5.657246	-3.516678
H	-11.514204	-3.903308	-3.539042
H	-7.650005	-5.720897	-2.940447
H	-9.073405	-6.733485	-3.162135
H	-9.298285	-4.604370	-4.443826
C	-4.151504	-8.246156	-1.631650
C	-2.770532	-8.008358	-2.261691
C	-2.310649	-6.570187	-1.976864
C	-3.324694	-5.583512	-2.575721
C	-4.707320	-5.814931	-1.946676

C	-5.160908	-7.255151	-2.231227
C	-2.227040	-6.353944	-0.457970
C	-4.062720	-8.025944	-0.113569
C	-4.617635	-5.601103	-0.427805
C	-3.606863	-6.587713	0.176501
H	-4.477567	-9.271833	-1.836050
H	-2.043866	-8.723290	-1.857265
H	-2.816645	-8.181025	-3.343824
H	-1.325658	-6.403054	-2.426470
H	-2.997028	-4.552351	-2.396282
H	-3.379881	-5.713722	-3.663313
H	-5.428717	-5.110823	-2.375474
H	-6.156302	-7.427593	-1.804120
H	-5.249025	-7.414909	-3.312749
H	-1.490760	-7.038448	-0.019582
H	-1.880960	-5.335968	-0.241665
H	-5.038436	-8.212846	0.351235
H	-3.358758	-8.740452	0.329911
H	-4.313088	-4.570170	-0.210446
H	-5.603536	-5.743588	0.031175
H	-3.543866	-6.433425	1.259311
C	-1.099761	-1.417394	-7.325968
C	-2.128797	-0.440995	-7.916148
C	-1.711550	1.004446	-7.603807
C	-1.637706	1.195170	-6.081105
C	-0.607457	0.222955	-5.485881
C	-1.027300	-1.220475	-5.803979
C	-0.330367	1.279561	-8.218166
C	0.279670	-1.136069	-7.941575
C	0.770858	0.499646	-6.105901
C	0.703148	0.307252	-7.628611
H	-1.399192	-2.447182	-7.549548
H	-2.205715	-0.584894	-9.000634
H	-3.122208	-0.644275	-7.498099
H	-2.446297	1.698852	-8.025985
H	-1.359989	2.229511	-5.844740
H	-2.622718	1.020906	-5.631397
H	-0.556898	0.360316	-4.400237
H	-0.310057	-1.926003	-5.367221
H	-2.001363	-1.437714	-5.349021
H	-0.373358	1.167111	-9.308277
H	-0.029579	2.315151	-8.018381
H	1.019958	-1.840620	-7.543357
H	0.246416	-1.291541	-9.026693
H	1.091226	1.521605	-5.869719
H	1.520092	-0.174957	-5.674015
H	1.686538	0.505460	-8.068960
C	1.022972	-4.107927	-1.679960
C	2.402818	-3.849800	-2.304443
C	2.849297	-2.411303	-2.000574
C	1.827453	-1.426219	-2.588702
C	0.445898	-1.677970	-1.965137
C	0.005732	-3.118464	-2.268743
C	2.928394	-2.213619	-0.478912

C	1.107210	-3.906219	-0.159051
C	0.531093	-1.482648	-0.443518
C	1.549654	-2.467764	0.150053
H	0.706471	-5.133804	-1.897937
H	3.135184	-4.563276	-1.907804
H	2.360086	-4.009121	-3.388760
H	3.833507	-2.229666	-2.446248
H	2.145551	-0.394528	-2.395612
H	1.775276	-1.543101	-3.677955
H	-0.281062	-0.974961	-2.386274
H	-0.988795	-3.305216	-1.845654
H	-0.079120	-3.265262	-3.352362
H	3.670044	-2.897016	-0.047912
H	3.264960	-1.195415	-0.249094
H	0.132426	-4.107732	0.301584
H	1.816804	-4.619970	0.276603
H	0.826010	-0.451873	-0.212555
H	-0.454264	-1.639766	0.011840
H	1.609434	-2.326677	1.234844
C	8.022592	-3.620046	-0.322310
C	6.522884	-3.721835	-0.004350
C	5.944007	-4.997982	-0.634671
C	6.143623	-4.949347	-2.157247
C	7.642392	-4.850614	-2.481055
C	8.217025	-3.575060	-1.845678
C	6.674789	-6.223659	-0.065053
C	8.747814	-4.849217	0.247024
C	8.369029	-6.076371	-1.906379
C	8.175024	-6.127940	-0.383241
H	8.433221	-2.709572	0.127724
H	6.368027	-3.734243	1.081282
H	5.996579	-2.840066	-0.389625
H	4.874853	-5.068791	-0.406240
H	5.716273	-5.846474	-2.621350
H	5.610206	-4.089273	-2.579852
H	7.782272	-4.815524	-3.566988
H	9.283097	-3.482154	-2.086146
H	7.720617	-2.690548	-2.263048
H	6.523467	-6.282523	1.019600
H	6.257170	-7.142925	-0.493280
H	9.823428	-4.778540	0.044399
H	8.632593	-4.882831	1.337140
H	7.981175	-6.993368	-2.366274
H	9.437682	-6.028120	-2.148489
H	8.693298	-7.002746	0.024626
C	-6.158727	3.526311	-6.878753
C	-5.397346	2.319647	-6.308802
C	-5.923114	1.025012	-6.947895
C	-7.421355	0.877939	-6.641149
C	-8.188238	2.080910	-7.211815
C	-7.656411	3.372884	-6.572355
C	-5.719673	1.089440	-8.469490
C	-5.954118	3.584273	-8.400503
C	-7.978723	2.143033	-8.732663

C	-6.481962	2.293230	-9.044743
H	-5.782562	4.448113	-6.421709
H	-4.323209	2.426239	-6.502707
H	-5.520339	2.278239	-5.219715
H	-5.378506	0.166100	-6.540515
H	-7.804431	-0.053442	-7.075475
H	-7.579311	0.810875	-5.557910
H	-9.256000	1.974200	-6.991485
H	-8.209518	4.238470	-6.956803
H	-7.818880	3.350233	-5.487888
H	-4.651604	1.173803	-8.703901
H	-6.073789	0.162040	-8.935713
H	-6.476352	4.454071	-8.817301
H	-4.889992	3.712793	-8.632616
H	-8.371911	1.234122	-9.203939
H	-8.538070	2.986431	-9.155481
H	-6.334482	2.336807	-10.129455
C	8.030734	1.718398	-2.055247
C	7.600774	0.277585	-2.371122
C	6.225319	-0.002569	-1.746149
C	6.310159	0.185556	-0.223697
C	6.736905	1.626145	0.098012
C	8.110865	1.902278	-0.532012
C	5.194464	0.979901	-2.323108
C	6.995241	2.696221	-2.631928
C	5.705077	2.604578	-0.483998
C	5.618621	2.422573	-2.007036
H	9.011536	1.915959	-2.501460
H	7.559225	0.126301	-3.456533
H	8.342369	-0.429843	-1.980564
H	5.920518	-1.030130	-1.973085
H	5.338607	-0.031228	0.236681
H	7.028855	-0.523899	0.204094
H	6.797255	1.757802	1.183863
H	8.436478	2.921974	-0.292924
H	8.861611	1.223487	-0.109398
H	5.108391	0.842355	-3.407777
H	4.203638	0.777410	-1.898733
H	7.301458	3.730039	-2.430811
H	6.941809	2.588272	-3.721998
H	4.722913	2.430698	-0.027995
H	5.987708	3.636684	-0.243494
H	4.882139	3.120564	-2.420137
C	-6.787486	-8.455857	4.421260
C	-8.293419	-8.569674	4.703742
C	-9.031376	-7.378545	4.073197
C	-8.493913	-6.070769	4.674282
C	-6.988367	-5.950300	4.391925
C	-6.255671	-7.145285	5.021302
C	-8.791544	-7.376776	2.555545
C	-6.553727	-8.451782	2.902613
C	-6.753569	-5.952633	2.873506
C	-7.286653	-7.260007	2.267831
H	-6.263289	-9.305773	4.871782

H	-8.682586	-9.511392	4.298222
H	-8.471924	-8.592576	5.785668
H	-10.104898	-7.462046	4.275350
H	-9.027213	-5.213213	4.246442
H	-8.676505	-6.050111	5.755526
H	-6.607271	-5.017552	4.821803
H	-5.176601	-7.061064	4.843875
H	-6.398263	-7.143475	6.108795
H	-9.189322	-8.296533	2.109837
H	-9.329437	-6.541534	2.090922
H	-5.479888	-8.391556	2.687581
H	-6.912198	-9.390889	2.464052
H	-7.255892	-5.092826	2.413918
H	-5.683388	-5.846616	2.658008
H	-7.118876	-7.259638	1.185195
C	-5.778019	4.428894	-2.706352
C	-6.798985	5.403813	-3.312772
C	-6.374595	6.850588	-3.016651
C	-6.305749	7.059758	-1.496139
C	-5.283566	6.089079	-0.884723
C	-5.710517	4.644257	-1.186635
C	-4.989383	7.110094	-3.628727
C	-4.394518	4.694604	-3.319760
C	-3.901190	6.350106	-1.502491
C	-3.963912	6.139231	-3.022966
H	-6.082534	3.398155	-2.918385
H	-6.872389	5.246917	-4.395697
H	-7.795229	5.211465	-2.896296
H	-7.103593	7.543927	-3.450390
H	-6.022973	8.095333	-1.271468
H	-7.293553	6.896767	-1.048331
H	-5.236563	6.239603	0.199335
H	-4.999145	3.940078	-0.738251
H	-6.687643	4.438298	-0.733001
H	-5.028657	6.984379	-4.717529
H	-4.683382	8.146331	-3.440554
H	-3.659941	3.990791	-2.909831
H	-4.424324	4.525876	-4.402997
H	-3.575835	7.373052	-1.277674
H	-3.157630	5.676605	-1.059230
H	-2.977629	6.326289	-3.461718
C	-6.259272	-5.544796	-7.287972
C	-4.867644	-5.278669	-7.882220
C	-4.441814	-3.833564	-7.580072
C	-5.458732	-2.863064	-8.200117
C	-6.852107	-3.122840	-7.606880
C	-7.271613	-4.569880	-7.908602
C	-4.400215	-3.622735	-6.058687
C	-6.212553	-5.329901	-5.767254
C	-6.804389	-2.914315	-6.085376
C	-5.790880	-3.884833	-5.459965
H	-6.561017	-6.575378	-7.504677
H	-4.138155	-5.981895	-7.462635
H	-4.883487	-5.447227	-8.965867

H	-3.449171	-3.646222	-8.004159
H	-5.154862	-1.826856	-8.008303
H	-5.484298	-2.989315	-9.289274
H	-7.575519	-2.430228	-8.050741
H	-8.273993	-4.762618	-7.507290
H	-7.329696	-4.726308	-8.992639
H	-3.662531	-4.295515	-5.604786
H	-4.078617	-2.599538	-5.829611
H	-7.195938	-5.536905	-5.327823
H	-5.506741	-6.033300	-5.309195
H	-6.524570	-1.878934	-5.856234
H	-7.798633	-3.077072	-5.651840
H	-5.757834	-3.734344	-4.375294
C	-0.779884	5.162470	-6.993181
C	-0.587341	5.231745	-8.515912
C	-1.357736	6.434456	-9.082422
C	-2.851989	6.279152	-8.760653
C	-3.050654	6.212447	-7.238487
C	-2.275608	5.010548	-6.676627
C	-0.830538	7.725876	-8.438150
C	-0.253670	6.457142	-6.354534
C	-2.518756	7.504172	-6.598748
C	-1.023739	7.662652	-6.915064
H	-0.230099	4.304325	-6.591393
H	0.478863	5.319821	-8.757124
H	-0.942290	4.304498	-8.981935
H	-1.218590	6.482224	-10.168075
H	-3.416010	7.122316	-9.177561
H	-3.246278	5.370456	-9.231465
H	-4.116548	6.101187	-7.011463
H	-2.425238	4.939742	-5.592393
H	-2.659940	4.079276	-7.110217
H	0.231353	7.860303	-8.677216
H	-1.359870	8.594158	-8.848915
H	-0.366676	6.411684	-5.264507
H	0.818397	6.568166	-6.557277
H	-3.077094	8.368928	-6.977264
H	-2.673291	7.477837	-5.513212
H	-0.646412	8.584356	-6.458627
C	5.020607	-5.162823	3.596460
C	4.552931	-6.597194	3.305868
C	3.174776	-6.833021	3.943070
C	3.273608	-6.623264	5.461847
C	3.738100	-5.188944	5.758293
C	5.114631	-4.957179	5.116114
C	2.165218	-5.834216	3.356577
C	4.006298	-4.168543	3.010345
C	2.727439	-4.194230	5.166820
C	2.627139	-4.397838	3.647370
H	6.003276	-4.996878	3.141561
H	4.500972	-6.764367	2.223240
H	5.279038	-7.316757	3.703298
H	2.843066	-7.856060	3.734155
H	2.299793	-6.808433	5.931158

H	3.977002	-7.343697	5.896668
H	3.808368	-5.041854	6.841564
H	5.467023	-3.942332	5.337184
H	5.850797	-5.647843	5.545090
H	2.069097	-5.986531	2.274729
H	1.172271	-6.005179	3.789776
H	4.339421	-3.139689	3.193338
H	3.943512	-4.292174	1.922442
H	1.744059	-4.336316	5.631140
H	3.037182	-3.165880	5.389356
H	1.905770	-3.688190	3.227520
C	2.884184	-7.727927	-0.227093
C	1.378877	-7.841753	0.058701
C	0.827792	-9.132380	-0.567032
C	1.060999	-9.101837	-2.085297
C	2.565530	-8.991156	-2.376916
C	3.112265	-7.701100	-1.746216
C	1.558505	-10.342366	0.035283
C	3.609382	-8.941470	0.374861
C	3.291988	-10.201199	-1.769637
C	3.064409	-10.234607	-0.250644
H	3.274977	-6.807143	0.219615
H	1.199837	-7.841398	1.140679
H	0.852070	-6.970951	-0.350118
H	-0.245412	-9.211747	-0.361557
H	0.653664	-10.009645	-2.546622
H	0.528150	-8.253303	-2.531298
H	2.729364	-8.968997	-3.459836
H	4.182424	-7.599833	-1.964092
H	2.616039	-6.827660	-2.186489
H	1.383513	-10.388436	1.116991
H	1.160328	-11.271726	-0.389661
H	4.688466	-8.861822	0.195330
H	3.470090	-8.961830	1.462492
H	2.924252	-11.128389	-2.225605
H	4.365236	-10.144575	-1.988491
H	3.582605	-11.098233	0.180486
C	0.408887	0.740958	8.211227
C	-0.615143	1.734650	7.641556
C	-1.989743	1.488154	8.282389
C	-2.443422	0.052329	7.977383
C	-1.424142	-0.945982	8.547777
C	-0.050894	-0.693011	7.906574
C	-1.882997	1.675214	9.803705
C	0.509547	0.930495	9.732703
C	-1.318397	-0.752505	10.068343
C	-0.861961	0.681232	10.378681
H	1.387997	0.918236	7.752941
H	-0.288789	2.763780	7.834212
H	-0.682270	1.621922	6.552639
H	-2.718109	2.198064	7.875205
H	-3.433357	-0.129752	8.412965
H	-2.542866	-0.089304	6.894375
H	-1.749324	-1.968873	8.328689

H	0.682221	-1.412633	8.290828
H	-0.107817	-0.848232	6.822303
H	-1.579601	2.703031	10.036895
H	-2.863021	1.520674	10.271150
H	1.253195	0.240237	10.149270
H	0.855332	1.945334	9.963557
H	-2.288394	-0.949089	10.540866
H	-0.608237	-1.473619	10.490993
H	-0.787660	0.817466	11.463194
C	1.207158	10.279680	-3.013776
C	0.782322	8.842034	-3.350336
C	-0.596028	8.550914	-2.736859
C	-0.520308	8.719908	-1.211685
C	-0.098720	10.157250	-0.869312
C	1.278174	10.444409	-1.487899
C	-1.625832	9.538322	-3.307227
C	0.172724	11.262427	-3.583953
C	-1.129458	11.140682	-1.444788
C	-1.206805	10.977812	-2.970480
H	2.190032	10.485068	-3.451820
H	0.747314	8.704433	-4.437797
H	1.523302	8.131364	-2.964525
H	-0.897165	7.525636	-2.978537
H	-1.493969	8.495140	-0.759668
H	0.197566	8.006689	-0.788801
H	-0.044868	10.275261	0.218448
H	1.600071	11.461714	-1.234051
H	2.028056	9.761989	-1.069620
H	-1.705399	9.414349	-4.394028
H	-2.618594	9.328259	-2.891140
H	0.475410	12.294291	-3.368003
H	0.125761	11.168191	-4.675590
H	-2.113805	10.958846	-0.996663
H	-0.850579	12.170283	-1.189614
H	-1.942522	11.679342	-3.378922
C	-6.950370	-0.669792	2.616275
C	-7.393468	-2.112637	2.329139
C	-8.778935	-2.362491	2.944774
C	-8.708837	-2.139771	4.463303
C	-8.269020	-0.696977	4.756240
C	-6.885038	-0.451244	4.135632
C	-9.791860	-1.382172	2.333423
C	-7.967989	0.305940	2.005256
C	-9.282908	0.279199	4.139923
C	-9.354545	0.062602	2.620647
H	-5.962448	-0.493850	2.176771
H	-7.424709	-2.288794	1.247139
H	-6.664508	-2.819091	2.744462
H	-9.093088	-3.391561	2.738356
H	-9.687891	-2.334703	4.917519
H	-8.003210	-2.847120	4.915633
H	-8.219213	-0.540643	5.839350
H	-6.550303	0.570012	4.354618
H	-6.146952	-1.128423	4.582438

H	-9.867467	-1.544073	1.251338
H	-10.789619	-1.563440	2.751096
H	-7.652061	1.340644	2.185736
H	-8.010557	0.173130	0.917457
H	-10.272026	0.127174	4.588646
H	-8.991021	1.313381	4.359546
H	-10.078252	0.759036	2.183066
C	-10.943538	0.314704	-2.672396
C	-9.557053	0.563653	-3.285804
C	-9.120385	2.011109	-3.011680
C	-10.138849	2.976990	-3.636381
C	-11.527058	2.734386	-3.024068
C	-11.957409	1.284968	-3.297802
C	-9.061189	2.246217	-1.494442
C	-10.879209	0.553840	-1.155946
C	-11.461755	2.967159	-1.506739
C	-10.446641	2.001364	-0.876637
H	-11.253027	-0.717585	-2.869136
H	-8.826766	-0.136666	-2.862761
H	-9.585528	0.377729	-4.366347
H	-8.131426	2.186193	-3.449393
H	-9.827476	4.014492	-3.464641
H	-10.176883	2.833331	-4.723014
H	-12.251588	3.423667	-3.471276
H	-12.956379	1.104173	-2.882668
H	-12.028061	1.111395	-4.378478
H	-8.322175	1.576848	-1.037671
H	-8.731754	3.271213	-1.285428
H	-11.858848	0.359292	-0.702673
H	-10.172181	-0.145903	-0.694178
H	-11.174031	4.004566	-1.297407
H	-12.452083	2.816817	-1.059960
H	-10.401050	2.169145	0.205038
C	-3.954393	0.833515	-1.267242
C	-5.453643	0.717710	-0.951928
C	-6.014396	-0.575524	-1.563528
C	-5.810820	-0.548487	-3.086116
C	-4.312453	-0.435842	-3.407230
C	-3.755951	0.856826	-2.790578
C	-5.269861	-1.782627	-0.972459
C	-3.215372	-0.377155	-0.676417
C	-3.572076	-1.642997	-2.811115
C	-3.770006	-1.672887	-1.287902
H	-3.556695	1.756156	-0.830571
H	-5.611604	0.720539	0.133324
H	-5.989916	1.586498	-1.352641
H	-7.083251	-0.656299	-1.337012
H	-6.225364	-1.458219	-3.537113
H	-6.353817	0.297927	-3.523828
H	-4.169754	-0.416188	-4.493186
H	-2.690421	0.959463	-3.029489
H	-4.262267	1.728227	-3.223340
H	-5.423696	-1.826215	0.112562
H	-5.674502	-2.713801	-1.387205

H	-2.140134	-0.296022	-0.877109
H	-3.333446	-0.394953	0.413767
H	-3.946888	-2.572025	-3.257477
H	-2.503395	-1.585000	-3.050951
H	-3.241920	-2.534457	-0.864734
C	0.847560	3.691205	-3.433866
C	1.409097	4.981432	-2.816960
C	1.215549	4.955158	-1.292885
C	-0.282714	4.842696	-0.971935
C	-0.848929	3.552133	-1.584106
C	-0.649745	3.581801	-3.107390
C	1.953449	3.741494	-0.706956
C	1.586923	2.480990	-2.842538
C	-0.105534	2.341999	-0.997870
C	1.393131	2.448118	-1.318786
H	0.986329	3.712070	-4.520336
H	2.473761	5.082693	-3.059793
H	0.901761	5.854347	-3.245424
H	1.617111	5.875697	-0.855115
H	-0.435679	4.843137	0.114094
H	-0.819199	5.713503	-1.367831
H	-1.917237	3.474334	-1.354511
H	-1.067861	2.674147	-3.559394
H	-1.193069	4.430071	-3.541228
H	3.028252	3.819084	-0.910950
H	1.838804	3.722366	0.383605
H	1.209059	1.553839	-3.290428
H	2.655041	2.537209	-3.084861
H	-0.255621	2.298124	0.087656
H	-0.514448	1.412346	-1.411995
H	1.921561	1.584813	-0.899600
C	-1.763736	3.422648	2.584583
C	-2.210497	1.984246	2.281283
C	-3.590681	1.726763	2.905582
C	-3.507018	1.928234	4.426390
C	-3.063487	3.366506	4.735518
C	-1.684836	3.619939	4.106203
C	-4.608176	2.716194	2.316923
C	-2.785928	4.407491	1.996233
C	-4.081993	4.351864	4.141844
C	-4.167196	4.156526	2.620388
H	-0.779588	3.604038	2.138870
H	-2.251418	1.823219	1.197250
H	-1.478466	1.271578	2.680265
H	-3.907460	0.700883	2.687619
H	-4.482183	1.727644	4.886470
H	-2.797992	1.214170	4.862568
H	-3.004007	3.507684	5.820221
H	-1.347370	4.637816	4.336441
H	-0.943377	2.936098	4.536991
H	-4.693451	2.569451	1.233396
H	-5.602358	2.529789	2.740820
H	-2.467597	5.439365	2.188324
H	-2.838191	4.289895	0.907106

H	-5.067236	4.194261	4.597119
H	-3.787358	5.382686	4.373279
H	-4.894180	4.859483	2.198965
C	-2.136967	-3.129225	2.226127
C	-1.577082	-1.835983	2.838203
C	-1.776263	-1.854027	4.361672
C	-3.275753	-1.964075	4.677687
C	-3.840334	-3.257624	4.070329
C	-3.635519	-3.236186	2.547655
C	-1.041118	-3.064889	4.956791
C	-1.400379	-4.336609	2.826633
C	-3.099694	-4.464957	4.665758
C	-1.599804	-4.361253	4.349813
H	-1.994183	-3.114228	1.140080
H	-0.511481	-1.736518	2.598763
H	-2.082411	-0.965133	2.403215
H	-1.375870	-0.931351	4.796000
H	-3.432722	-1.957761	5.763126
H	-3.810351	-1.095144	4.275170
H	-4.909519	-3.333699	4.296393
H	-4.052407	-4.146046	2.098964
H	-4.176828	-2.389992	2.107292
H	0.034468	-2.988890	4.756354
H	-1.159793	-3.078138	6.047009
H	-1.777039	-5.265963	2.382307
H	-0.331347	-4.282183	2.587956
H	-3.253805	-4.502963	5.750941
H	-3.507530	-5.396617	4.255097
H	-1.073343	-5.222552	4.775549
C	3.772846	-2.313609	-6.221688
C	4.361156	-1.039802	-5.595676
C	5.861773	-0.950417	-5.913658
C	6.578014	-2.182374	-5.339439
C	5.995491	-3.459378	-5.964607
C	4.494450	-3.542153	-5.646845
C	6.057860	-0.912533	-7.437110
C	3.974082	-2.271706	-7.744414
C	6.190751	-3.415008	-7.487971
C	5.473637	-2.186199	-8.067644
H	2.703139	-2.375508	-5.993640
H	3.840823	-0.155194	-5.982268
H	4.206791	-1.049130	-4.509841
H	6.278997	-0.041248	-5.466867
H	7.653991	-2.120464	-5.542659
H	6.462621	-2.211099	-4.249157
H	6.507202	-4.336361	-5.553389
H	4.070002	-4.460235	-6.071031
H	4.342111	-3.595372	-4.561977
H	5.568620	-0.025949	-7.858357
H	7.124827	-0.829247	-7.676853
H	3.539686	-3.166865	-8.205921
H	3.447628	-1.408916	-8.170088
H	7.259898	-3.375163	-7.729125
H	5.796956	-4.331128	-7.944714

H	5.614395	-2.155937	-9.153701
C	0.719319	-5.052004	-5.821101
C	-0.785844	-5.157003	-5.531201
C	-1.344365	-6.448934	-6.147580
C	-1.114491	-6.428990	-7.666530
C	0.389869	-6.327169	-7.962233
C	0.944048	-5.035757	-7.340874
C	-0.617921	-7.658525	-5.539338
C	1.440231	-6.265129	-5.213186
C	1.112068	-7.536767	-7.349017
C	0.887810	-7.559597	-5.829335
H	1.115418	-4.130277	-5.381062
H	-0.962405	-5.149036	-4.448845
H	-1.309522	-4.286327	-5.944284
H	-2.417454	-6.521997	-5.939188
H	-1.527105	-7.337756	-8.121230
H	-1.644400	-5.580785	-8.116640
H	0.551327	-6.312558	-9.045638
H	2.014166	-4.940858	-7.561797
H	0.450891	-4.162782	-7.785496
H	-0.790652	-7.697002	-4.456970
H	-1.021395	-8.588658	-5.957543
H	2.519260	-6.191650	-5.395649
H	1.303333	-6.278026	-4.125136
H	0.738975	-8.465066	-7.798328
H	2.185065	-7.486530	-7.570645
H	1.402962	-8.422918	-5.393967
C	4.329204	7.691184	-1.580650
C	4.498033	7.745423	-3.106802
C	5.990080	7.841983	-3.460953
C	6.580796	9.114051	-2.833781
C	6.417365	9.063634	-1.306805
C	4.924256	8.963542	-0.958160
C	6.725621	6.612621	-2.905581
C	5.068776	6.461843	-1.030468
C	7.151374	7.830948	-0.756851
C	6.562209	6.555662	-1.378765
H	3.264894	7.622602	-1.330347
H	4.058859	6.851727	-3.566330
H	3.958285	8.609064	-3.513924
H	6.108115	7.879958	-4.549490
H	7.641787	9.204908	-3.096270
H	6.077605	10.001367	-3.236676
H	6.838678	9.971703	-0.861884
H	4.792272	8.946734	0.130538
H	4.391721	9.848408	-1.327480
H	6.327644	5.697585	-3.360730
H	7.789260	6.660297	-3.168699
H	4.938692	6.400181	0.056856
H	4.640766	5.544761	-1.452991
H	8.222480	7.899301	-0.982411
H	7.059412	7.794429	0.335474
H	7.087096	5.677928	-0.985923
C	3.408313	2.249587	4.322527

C	1.909464	2.142449	4.642772
C	1.339703	0.851411	4.034869
C	1.538573	0.875109	2.511604
C	3.036503	0.979084	2.185565
C	3.602026	2.269587	2.798534
C	2.079505	-0.358883	4.625354
C	4.142580	1.035756	4.912789
C	3.772163	-0.231232	2.781109
C	3.578925	-0.257814	4.304985
H	3.812437	3.170675	4.756567
H	1.754977	2.147727	5.728515
H	1.376675	3.013572	4.242482
H	0.271148	0.776818	4.264899
H	1.117626	-0.032998	2.063271
H	0.998821	1.723842	2.074368
H	3.175848	0.996371	1.099134
H	4.667334	2.366042	2.556088
H	3.099106	3.143113	2.366096
H	1.928891	-0.400045	5.710921
H	1.668460	-1.288434	4.213272
H	5.217601	1.110710	4.708562
H	4.027884	1.020196	6.003367
H	3.390857	-1.158847	2.337314
H	4.840376	-0.179435	2.537793
H	4.103638	-1.121642	4.727746

Adamantane$_{39}$

1014
E=-1934.7855

C	-8.455237	0.182244	0.425371
C	-9.065704	-1.026749	-0.300060
C	-8.612248	-1.034568	-1.768144
C	-9.075780	0.261333	-2.450951
C	-8.465506	1.473909	-1.731137
C	-8.919001	1.475101	-0.263048
C	-7.079257	-1.119876	-1.826899
C	-6.922621	0.093279	0.361169
C	-6.932895	1.382084	-1.789506
C	-6.463913	0.089054	-1.105128
H	-8.779910	0.186058	1.471641
H	-8.759028	-1.956640	0.193887
H	-10.160289	-0.984484	-0.244834
H	-9.048194	-1.897645	-2.283251
H	-8.775227	0.258873	-3.505711
H	-10.170508	0.325552	-2.433577
H	-8.797745	2.396623	-2.219260
H	-8.505926	2.347502	0.257770
H	-10.010974	1.561226	-0.206856
H	-6.735261	-2.051154	-1.360826
H	-6.743914	-1.145650	-2.870792
H	-6.474100	0.940732	0.893755
H	-6.576900	-0.816770	0.866190
H	-6.594497	1.399715	-2.832576

H	-6.484628	2.253167	-1.296316
H	-5.371074	0.025054	-1.148191
C	2.132780	-11.098658	-2.244567
C	1.668147	-11.037556	-0.781363
C	2.309173	-9.828179	-0.083188
C	3.838723	-9.964836	-0.130621
C	4.309244	-10.023299	-1.592161
C	3.662796	-11.231936	-2.286599
C	1.891069	-8.540481	-0.809695
C	1.714591	-9.807719	-2.965357
C	3.885696	-8.735368	-2.315055
C	2.356340	-8.595524	-2.273118
H	1.674867	-11.961570	-2.740357
H	0.574940	-10.962829	-0.736130
H	1.944535	-11.962418	-0.260602
H	1.976086	-9.786259	0.959685
H	4.308095	-9.116797	0.382582
H	4.152716	-10.870709	0.401974
H	5.399871	-10.121267	-1.623535
H	4.006218	-11.296776	-3.326226
H	3.973978	-12.160355	-1.792450
H	0.801928	-8.419348	-0.765714
H	2.327124	-7.667706	-0.308743
H	2.022728	-9.847615	-4.017240
H	0.622271	-9.709963	-2.958980
H	4.356136	-7.865656	-1.840603
H	4.233638	-8.755677	-3.355002
H	2.056281	-7.676501	-2.788493
C	-8.330038	-4.754695	-7.232010
C	-8.788094	-3.447848	-7.897531
C	-8.176245	-2.247134	-7.159371
C	-8.634009	-2.264068	-5.692874
C	-8.175728	-3.567915	-5.021406
C	-8.787245	-4.765270	-5.765269
C	-6.643732	-2.342719	-7.214681
C	-6.797094	-4.843733	-7.287686
C	-6.643269	-3.660480	-5.082515
C	-6.180431	-3.647013	-6.547386
H	-8.767484	-5.609340	-7.759581
H	-8.485797	-3.435904	-8.951669
H	-9.882749	-3.381475	-7.880847
H	-8.503690	-1.316541	-7.635882
H	-8.219641	-1.399892	-5.159623
H	-9.725878	-2.176505	-5.638006
H	-8.503630	-3.578041	-3.976214
H	-8.483648	-5.702641	-5.283470
H	-9.881879	-4.722269	-5.711366
H	-6.301258	-2.310889	-8.256091
H	-6.194725	-1.480289	-6.707295
H	-6.458213	-5.782873	-6.833623
H	-6.458384	-4.856105	-8.330701
H	-6.193762	-2.821124	-4.538234
H	-6.301638	-4.577947	-4.588039
H	-5.087624	-3.712534	-6.588955

C	-2.821464	8.561116	7.097318
C	-2.099937	8.404072	5.749981
C	-2.406366	7.020898	5.155100
C	-1.924496	5.931436	6.125318
C	-2.645408	6.081952	7.473849
C	-2.338703	7.467675	8.062619
C	-3.922141	6.881194	4.946057
C	-4.336200	8.417830	6.882864
C	-4.160610	5.945131	7.258965
C	-4.648157	7.034518	6.291607
H	-2.601519	9.547664	7.519760
H	-2.420305	9.191504	5.057127
H	-1.018283	8.523189	5.887057
H	-1.891408	6.911342	4.194318
H	-2.119667	4.938417	5.702613
H	-0.839764	6.007309	6.268338
H	-2.299932	5.304605	8.164086
H	-2.831809	7.580677	9.035780
H	-1.261307	7.570664	8.240355
H	-4.276837	7.640623	4.238741
H	-4.152148	5.904429	4.503496
H	-4.864824	8.548492	7.834957
H	-4.697196	9.204834	6.209762
H	-4.395284	4.952248	6.856559
H	-4.685953	6.030494	8.217971
H	-5.728494	6.935063	6.139198
C	-8.652725	-10.137692	4.532296
C	-9.153057	-10.073011	5.983526
C	-8.584437	-8.823382	6.673491
C	-7.049539	-8.889792	6.662342
C	-6.543485	-8.951534	5.212858
C	-7.117640	-10.200612	4.526494
C	-9.044527	-7.569146	5.914546
C	-9.113180	-8.880124	3.779004
C	-7.009105	-7.697256	4.457400
C	-8.543831	-7.627748	4.463001
H	-9.059029	-11.029308	4.042350
H	-10.249292	-10.048269	6.003239
H	-8.846458	-10.974967	6.527057
H	-8.942890	-8.779000	7.707816
H	-6.631506	-8.012200	7.170218
H	-6.706528	-9.770835	7.218187
H	-5.449076	-8.999371	5.207365
H	-6.747887	-10.267351	3.496060
H	-6.775520	-11.104965	5.044258
H	-10.138805	-7.497887	5.932251
H	-8.660623	-6.668602	6.408988
H	-8.779530	-8.923797	2.735084
H	-10.208697	-8.833008	3.759477
H	-6.590115	-6.798634	4.926168
H	-6.636877	-7.719315	3.425933
H	-8.873925	-6.732630	3.924436
C	1.817667	-2.326481	10.804204
C	1.167263	-3.541842	10.125635

C	1.586070	-3.597431	8.648319
C	1.131748	-2.311059	7.941717
C	1.781897	-1.092262	8.614580
C	1.362923	-1.043295	10.092081
C	3.115344	-3.714696	8.556884
C	3.346368	-2.447538	10.707432
C	3.310675	-1.216022	8.523715
C	3.770536	-2.499592	9.231686
H	1.517654	-2.288591	11.857136
H	1.467435	-4.464274	10.637247
H	0.075283	-3.476398	10.204688
H	1.121678	-4.464978	8.166667
H	1.407479	-2.347339	6.880815
H	0.039163	-2.224670	7.982354
H	1.456171	-0.176341	8.109566
H	1.804767	-0.165823	10.579711
H	0.274421	-0.934042	10.170862
H	3.452142	-4.639992	9.039824
H	3.425655	-3.774683	7.506674
H	3.823437	-1.595394	11.206685
H	3.686209	-3.350622	11.228686
H	3.624893	-1.232667	7.473092
H	3.787024	-0.341297	8.982966
H	4.860638	-2.586403	9.165411
C	4.396230	-0.971749	-7.841671
C	3.754068	-2.171186	-8.555509
C	2.223339	-2.050055	-8.499390
C	1.768393	-2.022347	-7.032181
C	2.405425	-0.822573	-6.314101
C	3.935689	-0.946621	-6.376151
C	1.790119	-0.748018	-9.190629
C	3.957564	0.327346	-8.535119
C	1.972198	0.476367	-7.011097
C	2.427557	0.455001	-8.478216
H	5.487383	-1.060167	-7.882630
H	4.090646	-2.213165	-9.598430
H	4.074890	-3.106493	-8.080928
H	1.767622	-2.905920	-9.009393
H	0.674947	-1.956172	-6.978745
H	2.054210	-2.955315	-6.531447
H	2.080138	-0.805140	-5.268189
H	4.403130	-0.105774	-5.849426
H	4.259992	-1.860365	-5.863435
H	2.090611	-0.762950	-10.245294
H	0.697100	-0.659311	-9.174127
H	4.426028	1.190762	-8.047213
H	4.296867	0.330461	-9.578011
H	0.882383	0.586801	-6.957024
H	2.404311	1.342463	-6.495228
H	2.117352	1.381592	-8.973647
C	-7.430718	-2.648315	3.461810
C	-7.091915	-1.275651	4.063136
C	-7.813548	-1.106057	5.409032
C	-9.330646	-1.209944	5.189792

C	-9.675509	-2.582426	4.591236
C	-8.948803	-2.748423	3.247625
C	-7.361957	-2.214936	6.371967
C	-6.979587	-3.753033	4.429811
C	-9.218802	-3.687640	5.555941
C	-7.701499	-3.590026	5.776186
H	-6.915491	-2.767080	2.502360
H	-6.008096	-1.182614	4.202462
H	-7.392357	-0.478486	3.372328
H	-7.570119	-0.126993	5.836189
H	-9.858451	-1.070448	6.141012
H	-9.669929	-0.411265	4.519081
H	-10.757452	-2.654035	4.435290
H	-9.202339	-3.716968	2.799735
H	-9.281602	-1.977220	2.542311
H	-6.283019	-2.140024	6.553947
H	-7.856040	-2.093356	7.343529
H	-7.197445	-4.739433	4.002550
H	-5.893854	-3.704488	4.576730
H	-9.744854	-3.592064	6.513517
H	-9.477712	-4.672802	5.149263
H	-7.377810	-4.378397	6.464623
C	9.059642	3.408955	5.416301
C	8.578476	4.682513	4.704183
C	7.047625	4.780174	4.793135
C	6.419231	3.548841	4.122772
C	6.894786	2.272109	4.833201
C	8.426056	2.181056	4.744505
C	6.625479	4.825985	6.269781
C	8.632535	3.458805	6.891442
C	6.473503	2.324401	6.309859
C	7.101927	3.552608	6.985757
H	10.151117	3.341150	5.351525
H	9.039520	5.566527	5.161007
H	8.893890	4.667469	3.653830
H	6.706842	5.689154	4.285287
H	5.325281	3.616572	4.162062
H	6.696682	3.514557	3.062253
H	6.445998	1.395567	4.353293
H	8.777327	1.263026	5.231122
H	8.739096	2.122135	3.694975
H	7.050595	5.712276	6.756196
H	5.535070	4.915219	6.346448
H	8.988161	2.563826	7.416360
H	9.093656	4.321154	7.387908
H	5.380502	2.370211	6.387763
H	6.789595	1.408623	6.824059
H	6.800040	3.588525	8.038289
C	-2.285430	-7.981706	2.391214
C	-1.577283	-8.194270	1.044393
C	-1.947275	-9.572694	0.475176
C	-1.510104	-10.665291	1.462881
C	-2.217921	-10.459254	2.811010
C	-1.847567	-9.078404	3.374070

C	-3.468625	-9.648571	0.273865
C	-3.805925	-8.061303	2.184553
C	-3.738596	-10.532380	2.603805
C	-4.181429	-9.439556	1.618980
H	-2.020159	-6.998641	2.795346
H	-1.865337	-7.405664	0.338802
H	-0.490841	-8.120934	1.175609
H	-1.441751	-9.721862	-0.485283
H	-1.750914	-11.655925	1.058515
H	-0.422479	-10.635181	1.600796
H	-1.904349	-11.238947	3.513719
H	-2.331055	-8.926546	4.346770
H	-0.765921	-9.020274	3.546241
H	-3.792275	-8.886666	-0.445571
H	-3.743599	-10.621771	-0.150522
H	-4.324202	-7.890598	3.135996
H	-4.134501	-7.270974	1.498858
H	-4.018778	-11.520767	2.219868
H	-4.255575	-10.406946	3.562953
H	-5.265749	-9.493581	1.472081
C	-1.551469	-4.294733	-11.188763
C	-1.178330	-2.915957	-10.622787
C	-1.915787	-2.682885	-9.295087
C	-3.431703	-2.745124	-9.537101
C	-3.810936	-4.123443	-10.100418
C	-3.068330	-4.353013	-11.425854
C	-1.515833	-3.775904	-8.292023
C	-1.151922	-5.383427	-10.180767
C	-3.405780	-5.212909	-9.095606
C	-1.889878	-5.156913	-8.852397
H	-1.024893	-4.458777	-12.135291
H	-0.094469	-2.853214	-10.467740
H	-1.441689	-2.130919	-11.342030
H	-1.647840	-1.699570	-8.893112
H	-3.970047	-2.560219	-8.599629
H	-3.734154	-1.956732	-10.236969
H	-4.891963	-4.165340	-10.272679
H	-3.345702	-5.326126	-11.849035
H	-3.364629	-3.593323	-12.159377
H	-0.438124	-3.729824	-8.093893
H	-2.021383	-3.609353	-7.333110
H	-1.394548	-6.374886	-10.582189
H	-0.067706	-5.364965	-10.016777
H	-3.943892	-5.071745	-8.150446
H	-3.689725	-6.201204	-9.477197
H	-1.602964	-5.933976	-8.135387
C	-6.745692	3.866746	4.333611
C	-7.247600	5.144630	3.643912
C	-8.781556	5.202746	3.711395
C	-9.369182	3.972603	3.002952
C	-8.872780	2.691450	3.690802
C	-7.338572	2.639942	3.623735
C	-9.226674	5.203357	5.181993
C	-7.195847	3.871308	5.802721

C	-9.317196	2.698559	5.161576
C	-8.729546	3.925448	5.875479
H	-5.652028	3.827167	4.284185
H	-6.815463	6.028609	4.128237
H	-6.916286	5.162097	2.598503
H	-9.137150	6.114868	3.219589
H	-10.464938	4.012649	3.026862
H	-9.075188	3.970154	1.946346
H	-9.292506	1.815783	3.183765
H	-6.971953	1.719533	4.094270
H	-7.008557	2.613480	2.578093
H	-8.830949	6.088036	5.695395
H	-10.319982	5.264098	5.243820
H	-6.826018	2.973138	6.312146
H	-6.763688	4.732700	6.326172
H	-10.412036	2.715808	5.223590
H	-8.986209	1.778889	5.659191
H	-9.047887	3.929120	6.923761
C	3.668127	-7.489551	-8.763617
C	4.048758	-6.111627	-8.200563
C	3.319493	-5.875473	-6.868888
C	1.802055	-5.933090	-7.102363
C	1.415347	-7.310536	-7.662726
C	2.149785	-7.543203	-8.992166
C	3.721633	-6.969164	-5.867432
C	4.069902	-8.578912	-7.757230
C	1.822711	-8.400689	-6.659553
C	3.340121	-8.349314	-6.424879
H	4.188870	-7.655794	-9.712985
H	5.133663	-6.052199	-8.051632
H	3.783834	-5.326181	-8.918785
H	3.592773	-4.892773	-6.469007
H	1.269560	-5.745954	-6.161998
H	1.498160	-5.144156	-7.800994
H	0.333245	-7.349137	-7.828899
H	1.866992	-8.515684	-9.413207
H	1.851762	-6.783010	-9.724469
H	4.800576	-6.926354	-5.675373
H	3.221994	-6.800472	-4.905800
H	3.821919	-9.569836	-8.156695
H	5.155073	-8.563761	-7.599331
H	1.290353	-8.257290	-5.711475
H	1.533531	-9.388308	-7.038958
H	3.628611	-9.126860	-5.709026
C	-3.379461	-4.190277	0.716426
C	-3.820689	-4.165484	2.187917
C	-3.192855	-2.956032	2.897849
C	-1.661846	-3.060127	2.822872
C	-1.214757	-3.082189	1.352998
C	-1.847964	-4.291083	0.646903
C	-3.649223	-1.664230	2.202316
C	-3.835885	-2.895341	0.026669
C	-1.676511	-1.790333	0.661169
C	-3.207484	-1.682948	0.730708

H	-3.827922	-5.053297	0.212255
H	-4.914282	-4.114229	2.252407
H	-3.516828	-5.093770	2.686823
H	-3.509227	-2.940031	3.946630
H	-1.201335	-2.212054	3.343987
H	-1.320582	-3.968868	3.333346
H	-0.123038	-3.156983	1.301973
H	-1.520740	-4.329840	-0.399227
H	-1.509694	-5.221718	1.118570
H	-4.739718	-1.566479	2.266349
H	-3.222727	-0.791897	2.712188
H	-3.544646	-2.909672	-1.030673
H	-4.929710	-2.820339	0.052728
H	-1.216023	-0.919868	1.143930
H	-1.345681	-1.784478	-0.384528
H	-3.534799	-0.761088	0.237464
C	7.174374	-1.479384	-0.614338
C	6.732228	-1.441657	0.856603
C	7.376979	-0.238350	1.561800
C	8.906376	-0.365066	1.488760
C	9.354420	-0.400141	0.019429
C	8.704301	-1.602795	-0.681938
C	6.940110	1.056874	0.860135
C	6.737489	-0.181001	-1.310240
C	8.912162	0.895230	-0.678536
C	7.382854	1.025212	-0.610949
H	6.713839	-2.338002	-1.115130
H	5.639440	-1.374192	0.919811
H	7.022081	-2.372054	1.359891
H	7.059930	-0.213102	2.610193
H	9.378726	0.478505	2.006573
H	9.233903	-1.276414	2.003566
H	10.444979	-0.491056	-0.030212
H	9.031868	-1.650943	-1.727570
H	9.028550	-2.536163	-0.205844
H	5.851097	1.170782	0.922685
H	7.378812	1.925149	1.366566
H	7.029440	-0.204251	-2.367227
H	5.644849	-0.089958	-1.285567
H	9.384849	1.761023	-0.199175
H	9.243983	0.891639	-1.723929
H	7.069451	1.949551	-1.108569
C	3.072473	6.984128	-7.552801
C	3.506005	5.892313	-6.562562
C	3.185453	4.506787	-7.144383
C	1.674059	4.398573	-7.398207
C	1.235490	5.486581	-8.390579
C	1.561390	6.869593	-7.805760
C	3.938079	4.325539	-8.471596
C	3.825901	6.796486	-8.878728
C	1.993357	5.303233	-9.714495
C	3.505506	5.413653	-9.466584
H	3.301655	7.970986	-7.136030
H	4.580053	5.975245	-6.356952

H	2.988554	6.026878	-5.604914
H	3.495885	3.729920	-6.436996
H	1.429592	3.405886	-7.795278
H	1.124963	4.506577	-6.454977
H	0.157621	5.407869	-8.569609
H	1.234974	7.656688	-8.496339
H	1.009983	7.021581	-6.869904
H	5.020246	4.379922	-8.301912
H	3.732450	3.331859	-8.887709
H	3.540276	7.582759	-9.588088
H	4.905878	6.894779	-8.715186
H	1.754292	4.326644	-10.152604
H	1.674116	6.062164	-10.439115
H	4.044188	5.282145	-10.411453
C	8.304993	3.804984	-5.220438
C	8.759300	2.736305	-4.214399
C	8.432917	1.337497	-4.760076
C	6.917640	1.219589	-4.984757
C	6.458270	2.284402	-5.992755
C	6.790062	3.680777	-5.444123
C	9.162649	1.129314	-6.096092
C	9.035569	3.590438	-6.555045
C	7.193308	2.074205	-7.325524
C	8.709228	2.194190	-7.106815
H	8.538369	4.801356	-4.829429
H	9.836532	2.826676	-4.029641
H	8.258352	2.890375	-3.250934
H	8.758179	0.577159	-4.041482
H	6.669345	0.217779	-5.355677
H	6.384881	1.346702	-4.034564
H	5.377672	2.198821	-6.150969
H	6.449081	4.451664	-6.145857
H	6.254724	3.851695	-4.502221
H	10.247457	1.190379	-5.946766
H	8.952855	0.126200	-6.486711
H	8.735045	4.360204	-7.276254
H	10.117942	3.695262	-6.412778
H	6.949642	1.087605	-7.737913
H	6.858990	2.816210	-8.060796
H	9.231619	2.043515	-8.057948
C	7.450628	-5.220090	-6.048818
C	6.994762	-5.191897	-4.581857
C	7.644656	-4.001884	-3.859041
C	9.173347	-4.143265	-3.918737
C	9.635121	-4.168880	-5.384006
C	8.979829	-5.358222	-6.103037
C	7.227471	-2.695728	-4.552370
C	7.033403	-3.910829	-6.736354
C	9.212514	-2.862557	-6.073685
C	7.683997	-2.717860	-6.019410
H	6.986400	-6.069239	-6.562187
H	5.902143	-5.114057	-4.528407
H	7.270484	-6.129899	-4.084784
H	7.317811	-3.983413	-2.813519

H	9.649074	-3.309441	-3.388380
H	9.486818	-5.062709	-3.409606
H	10.725147	-4.270274	-5.424137
H	9.316936	-5.399723	-7.145918
H	9.290176	-6.299283	-5.632858
H	6.139096	-2.571481	-4.499096
H	7.669910	-1.836757	-4.033478
H	7.335255	-3.926968	-7.790689
H	5.941538	-3.809048	-6.721208
H	9.689171	-2.006152	-5.581561
H	9.554316	-2.859550	-7.115859
H	7.384613	-1.785732	-6.511097
C	-3.115407	-7.929471	-4.739957
C	-1.584052	-8.039699	-4.798147
C	-0.949463	-6.840477	-4.076956
C	-1.396724	-5.539866	-4.761659
C	-2.927695	-5.422986	-4.703595
C	-3.557240	-6.626034	-5.422827
C	-1.408719	-6.827044	-2.610772
C	-3.568806	-7.913700	-3.271922
C	-3.382073	-5.413629	-3.235880
C	-2.939817	-6.713816	-2.547280
H	-3.565783	-8.785159	-5.254806
H	-1.255866	-8.976476	-4.331806
H	-1.249144	-8.070886	-5.842089
H	0.142301	-6.921426	-4.119190
H	-0.934045	-4.676692	-4.267983
H	-1.057989	-5.527472	-5.804712
H	-3.244309	-4.495349	-5.192771
H	-4.650879	-6.544592	-5.405869
H	-3.257110	-6.632697	-6.477747
H	-1.077953	-7.741492	-2.103610
H	-0.946818	-5.985858	-2.079842
H	-4.662707	-7.855920	-3.216788
H	-3.275828	-8.847713	-2.777408
H	-2.954687	-4.548061	-2.715349
H	-4.472545	-5.310040	-3.180154
H	-3.264156	-6.705114	-1.500868
C	2.811733	10.699679	-2.155960
C	3.234682	9.608978	-1.159933
C	2.908012	8.223248	-1.737853
C	1.397006	8.123260	-1.997297
C	0.969005	9.210176	-2.995463
C	1.300996	10.593371	-2.414515
C	3.664778	8.032451	-3.061368
C	3.569257	10.502486	-3.478163
C	1.730987	9.017259	-4.315653
C	3.242784	9.119426	-4.062128
H	3.045263	11.686685	-1.741965
H	4.308391	9.686159	-0.950352
H	2.714266	9.750279	-0.204865
H	3.210902	7.447173	-1.026339
H	1.148078	7.130591	-2.391632
H	0.844842	8.238138	-1.056673

H	-0.108604	9.137338	-3.178495
H	0.982118	11.379834	-3.109323
H	0.746817	10.752218	-1.481438
H	4.746575	8.080899	-2.887574
H	3.454757	7.038481	-3.474586
H	3.291238	11.287809	-4.191585
H	4.649155	10.594834	-3.310684
H	1.487721	8.040500	-4.751064
H	1.419246	9.775387	-5.044367
H	3.784399	8.981102	-5.004343
C	-7.094830	2.713790	-8.195161
C	-6.762863	4.079598	-7.574716
C	-7.521605	4.245755	-6.248950
C	-9.032523	4.155409	-6.512413
C	-9.370625	2.789812	-7.130263
C	-8.606849	2.627196	-8.453573
C	-7.106516	3.126799	-5.281256
C	-6.680315	1.598965	-7.222388
C	-8.950445	1.674469	-6.160627
C	-7.439400	1.758534	-5.896197
H	-6.553133	2.597469	-9.140224
H	-5.682816	4.163078	-7.403657
H	-7.037116	4.883804	-8.268251
H	-7.283002	5.219926	-5.808104
H	-9.586395	4.292615	-5.575793
H	-9.346166	4.961301	-7.186965
H	-10.448153	2.727857	-7.317730
H	-8.854876	1.663764	-8.915360
H	-8.913229	3.405798	-9.162720
H	-6.032716	3.191904	-5.067830
H	-7.627405	3.245685	-4.323460
H	-6.893411	0.617256	-7.662654
H	-5.598917	1.637889	-7.043997
H	-9.503088	1.767701	-5.217912
H	-9.205158	0.694183	-6.581490
H	-7.141774	0.962952	-5.204303
C	2.103776	2.912645	-0.873341
C	1.477078	1.702822	-1.583385
C	1.920105	1.677405	-3.054451
C	1.463774	2.971286	-3.745886
C	2.090273	4.184641	-3.041490
C	1.647145	4.203432	-1.570358
C	3.451791	1.577308	-3.123071
C	3.635015	2.808845	-0.947373
C	3.621521	4.078036	-3.109663
C	4.083339	2.786989	-2.416720
H	1.786520	2.929015	0.175082
H	1.778617	0.774659	-1.083066
H	0.383346	1.755769	-1.520944
H	1.472591	0.813762	-3.558572
H	1.756865	2.956417	-4.802642
H	0.369835	3.045841	-3.721495
H	1.763147	5.105919	-3.535741
H	2.071915	5.076664	-1.060453

H	0.556437	4.300220	-1.507371
H	3.790439	0.647157	-2.650872
H	3.779531	1.538903	-4.168990
H	4.095082	3.656908	-0.425717
H	3.975858	1.900260	-0.436434
H	3.952722	4.083010	-4.155177
H	4.081275	4.949370	-2.627616
H	5.175211	2.712436	-2.466788
C	-3.452372	0.787264	8.351723
C	-4.082450	-0.428585	7.655091
C	-3.656998	-0.459317	6.178947
C	-4.127027	0.829283	5.486826
C	-3.497230	2.048637	6.177796
C	-3.922754	2.072743	7.654040
C	-2.125838	-0.553394	6.092409
C	-1.921708	0.689456	8.259798
C	-1.966475	1.948051	6.091743
C	-1.490954	0.662315	6.785291
H	-3.757095	0.807416	9.403791
H	-3.771031	-1.353046	8.156211
H	-5.175567	-0.379871	7.730413
H	-4.106892	-1.327249	5.684401
H	-3.846574	0.810492	4.426708
H	-5.220898	0.899419	5.524001
H	-3.834132	2.966136	5.683084
H	-3.495573	2.950197	8.154596
H	-5.013026	2.165312	7.729661
H	-1.777643	-1.479826	6.564976
H	-1.810510	-0.595525	5.042825
H	-1.458987	1.541945	8.771801
H	-1.570944	-0.215213	8.770970
H	-1.647864	1.949315	5.042313
H	-1.504640	2.823617	6.564032
H	-0.399457	0.592047	6.722467
C	1.837226	6.680793	4.545860
C	1.217315	5.463336	3.842943
C	1.644740	5.442585	2.367196
C	1.165666	6.730683	1.680363
C	1.785275	7.951668	2.377634
C	1.357829	7.965793	3.853451
C	3.176678	5.360636	2.282100
C	3.368703	6.595134	4.455360
C	3.316858	7.863206	2.292993
C	3.801400	6.578008	2.981273
H	1.531110	6.693820	5.597637
H	1.535218	4.539016	4.340238
H	0.123768	5.503330	3.917153
H	1.202101	4.573491	1.868163
H	1.447512	6.718867	0.620514
H	0.071246	6.792266	1.716521
H	1.441936	8.868802	1.886684
H	1.777676	8.844206	4.358510
H	0.266791	8.049661	3.928161
H	3.531405	4.434757	2.750878

H	3.493564	5.325701	1.232722
H	3.824267	7.448805	4.971796
H	3.725786	5.690862	4.962846
H	3.636694	7.871689	1.243969
H	3.771406	8.740126	2.769818
H	4.893477	6.516391	2.919462
C	-7.370993	6.421642	-2.749443
C	-8.882442	6.323349	-3.007212
C	-9.646385	6.485451	-1.683933
C	-9.218707	5.376694	-0.710179
C	-7.708019	5.472269	-0.446372
C	-6.949037	5.313164	-1.772863
C	-9.317312	7.855311	-1.070757
C	-7.048042	7.791964	-2.133920
C	-7.383691	6.844840	0.163213
C	-7.806754	7.957303	-0.808223
H	-6.828282	6.305604	-3.693958
H	-9.194169	7.096921	-3.719352
H	-9.123344	5.355924	-3.464385
H	-10.723754	6.415603	-1.870167
H	-9.772259	5.469647	0.232050
H	-9.466051	4.392966	-1.127259
H	-7.405287	4.680573	0.247616
H	-5.867645	5.359675	-1.595676
H	-7.155842	4.328117	-2.208629
H	-9.636450	8.657630	-1.746975
H	-9.872024	7.991109	-0.134441
H	-5.968304	7.882787	-1.963879
H	-7.327356	8.592393	-2.829662
H	-7.904609	6.963587	1.121003
H	-6.310104	6.918246	0.375318
H	-7.575256	8.935117	-0.371715
C	-2.232271	1.062821	-3.833513
C	-3.748186	0.963946	-4.063320
C	-4.097230	-0.418036	-4.637120
C	-3.361941	-0.615602	-5.971573
C	-1.844885	-0.520741	-5.746963
C	-1.501634	0.861351	-5.169947
C	-3.655120	-1.506672	-3.647068
C	-1.795343	-0.029495	-2.845137
C	-1.408462	-1.608759	-4.753743
C	-2.138803	-1.413800	-3.416268
H	-1.985576	2.048777	-3.424684
H	-4.283519	1.126337	-3.120039
H	-4.075598	1.751169	-4.753271
H	-5.178520	-0.486503	-4.799633
H	-3.618793	-1.591307	-6.401554
H	-3.683167	0.143809	-6.694736
H	-1.322772	-0.661337	-6.699685
H	-0.418266	0.949495	-5.023280
H	-1.789539	1.646979	-5.879085
H	-4.188112	-1.390109	-2.695665
H	-3.916427	-2.497919	-4.036917
H	-0.717317	0.043338	-2.656585

H	-2.295921	0.114092	-1.879962
H	-1.630553	-2.602000	-5.162356
H	-0.323354	-1.564810	-4.600206
H	-1.826813	-2.190691	-2.709620
C	1.864505	-7.349933	3.202296
C	1.426341	-7.302410	4.674144
C	2.062639	-6.087062	5.366248
C	3.592862	-6.201164	5.289798
C	4.036906	-6.245969	3.819518
C	3.395245	-7.460674	3.131253
C	1.612457	-4.802243	4.653951
C	1.414296	-6.061901	2.495731
C	3.581345	-4.961014	3.110935
C	2.051167	-4.843697	3.181902
H	1.410008	-8.217124	2.710847
H	0.333196	-7.243854	4.740001
H	1.725755	-8.225548	5.185142
H	1.748442	-6.054849	6.415309
H	4.059354	-5.348757	5.798371
H	3.929816	-7.104821	5.812025
H	5.128066	-6.327866	3.767450
H	3.720167	-7.515669	2.085134
H	3.729000	-8.386737	3.614982
H	0.522681	-4.697221	4.718709
H	2.045055	-3.925533	5.151014
H	1.703354	-6.092414	1.438134
H	0.320982	-5.980126	2.522837
H	4.047869	-4.086742	3.580851
H	3.910134	-4.971416	2.064629
H	1.728273	-3.926768	2.676698
C	-2.995667	1.128929	-10.972032
C	-3.470287	1.135445	-9.510773
C	-2.856757	2.334303	-8.770993
C	-1.324614	2.228148	-8.812435
C	-0.844167	2.224330	-10.271908
C	-1.463156	1.025991	-11.007966
C	-3.294961	3.635954	-9.459813
C	-3.434054	2.433638	-11.655037
C	-1.287841	3.525996	-10.957146
C	-2.819818	3.635512	-10.921097
H	-3.433976	0.273438	-11.497512
H	-4.564978	1.188031	-9.470544
H	-3.179451	0.200142	-9.017240
H	-3.196931	2.337281	-7.729571
H	-0.874671	3.068448	-8.269937
H	-0.996616	1.312163	-8.306230
H	0.248304	2.148035	-10.299027
H	-1.112231	1.000202	-12.046789
H	-1.137302	0.088624	-10.540909
H	-4.386466	3.735032	-9.419362
H	-2.878726	4.500726	-8.929013
H	-3.118808	2.432492	-12.705569
H	-4.528061	2.510471	-11.652920
H	-0.837008	4.389187	-10.452697

H	-0.933247	3.544804	-11.994871
H	-3.134241	4.564363	-11.409589
C	8.282596	-5.277574	1.037306
C	6.762515	-5.385135	0.841428
C	6.405086	-6.781891	0.310138
C	7.113214	-7.011844	-1.033783
C	8.634339	-6.908424	-0.843022
C	8.986043	-5.511488	-0.308400
C	6.870133	-7.844217	1.318099
C	8.742439	-6.343631	2.043792
C	9.093765	-7.970062	0.168208
C	8.390652	-7.742630	1.515113
H	8.535263	-4.281095	1.415855
H	6.246329	-5.199888	1.791148
H	6.419052	-4.616293	0.138672
H	5.320847	-6.856519	0.171774
H	6.849931	-7.998630	-1.433523
H	6.775319	-6.271636	-1.769157
H	9.137059	-7.072148	-1.802456
H	10.071984	-5.417587	-0.186415
H	8.681687	-4.744652	-1.031109
H	6.356566	-7.704573	2.277049
H	6.603261	-8.845530	0.958956
H	9.823939	-6.263983	2.208138
H	8.261497	-6.176560	3.015153
H	8.865719	-8.973771	-0.210449
H	10.181695	-7.920165	0.298143
H	8.719015	-8.500749	2.234615
C	2.734935	1.640203	4.049879
C	1.218886	1.532642	3.824911
C	0.876029	0.148971	3.251452
C	1.608138	-0.043581	1.914520
C	3.125334	0.059976	2.134285
C	3.462409	1.443722	2.710978
C	1.327656	-0.937622	4.239451
C	3.181397	0.549902	5.036216
C	3.571282	-1.026033	3.125471
C	2.844145	-0.836062	4.465407
H	2.977215	2.627352	4.458471
H	0.685669	1.691398	4.770007
H	0.884676	2.318329	3.136469
H	-0.205365	0.074308	3.092392
H	1.355572	-1.020525	1.484814
H	1.280167	0.714348	1.192831
H	3.645174	-0.077057	1.179802
H	4.545714	1.538083	2.854197
H	3.167651	2.228054	2.003221
H	0.797072	-0.824685	5.192636
H	1.070857	-1.930156	3.849883
H	4.259585	0.628898	5.221328
H	2.683114	0.690043	6.003084
H	3.353648	-2.020324	2.717011
H	4.656607	-0.975860	3.275529
H	3.162928	-1.611515	5.170602

C	-2.178330	-5.849294	5.926009
C	-1.846579	-4.463895	6.501505
C	-2.571994	-4.271406	7.842279
C	-4.088144	-4.385828	7.621721
C	-4.425961	-5.771018	7.048976
C	-3.695493	-5.959845	5.710430
C	-2.118041	-5.359629	8.827413
C	-1.724866	-6.933260	6.916127
C	-3.966911	-6.855519	8.035821
C	-2.450534	-6.747349	8.257506
H	-1.660415	-5.984377	4.970170
H	-0.763476	-4.363668	6.641413
H	-2.148723	-3.681452	5.794791
H	-2.333591	-3.283285	8.251017
H	-4.618703	-4.230230	8.568902
H	-4.429180	-3.601646	6.934984
H	-5.507239	-5.850132	6.892043
H	-3.943998	-6.937891	5.280737
H	-4.029853	-5.203772	4.989641
H	-1.039842	-5.276722	9.010305
H	-2.614851	-5.221409	9.795353
H	-1.937667	-7.928611	6.507483
H	-0.639682	-6.877362	7.064488
H	-4.495551	-6.743703	8.990209
H	-4.220817	-7.849407	7.647642
H	-2.125174	-7.520949	8.961731
C	-3.135363	6.087108	-3.324542
C	-3.752228	4.876505	-4.041830
C	-3.317115	4.869056	-5.515456
C	-3.791928	6.163675	-6.192924
C	-3.175334	7.377858	-5.481251
C	-3.610472	7.378680	-4.007616
C	-1.784796	4.787024	-5.593309
C	-1.603480	6.001416	-3.407835
C	-1.643377	7.289309	-5.558711
C	-1.163101	5.997573	-4.879741
H	-3.446960	6.090655	-2.274301
H	-3.437405	3.947480	-3.551390
H	-4.846127	4.916436	-3.972962
H	-3.757593	4.004830	-6.024749
H	-3.504559	6.161427	-7.251353
H	-4.886490	6.225544	-6.161912
H	-3.515630	8.299653	-5.965545
H	-3.192812	8.252183	-3.492332
H	-4.701842	7.462477	-3.937832
H	-1.433001	3.856681	-5.131207
H	-1.462454	4.761541	-6.641297
H	-1.150176	6.850052	-2.881215
H	-1.249520	5.092322	-2.906817
H	-1.318064	7.307237	-6.605930
H	-1.190870	8.161558	-5.071491
H	-0.070748	5.935908	-4.936418
C	0.606540	3.868804	8.701987
C	1.094784	2.786086	9.676627

C	2.609756	2.926082	9.891606
C	3.331776	2.767475	8.544766
C	2.849368	3.850058	7.566910
C	1.333720	3.707965	7.358181
C	2.915262	4.315695	10.471633
C	0.916960	5.255892	9.285574
C	3.153780	5.237364	8.152956
C	2.431417	5.402237	9.498785
H	-0.473575	3.767084	8.549950
H	0.568436	2.876506	10.634496
H	0.861759	1.790604	9.279552
H	2.955568	2.153541	10.587222
H	4.416318	2.845541	8.688054
H	3.138052	1.771476	8.128459
H	3.365007	3.734713	6.607296
H	0.979415	4.463199	6.646103
H	1.104579	2.728743	6.920461
H	2.422357	4.435120	11.444062
H	3.992630	4.420972	10.647785
H	0.554542	6.038613	8.608079
H	0.388270	5.390950	10.236891
H	4.235199	5.359143	8.289043
H	2.832327	6.019690	7.454673
H	2.650044	6.391650	9.915334
C	3.397978	-3.697518	-3.297084
C	3.737584	-2.313322	-2.723278
C	3.009098	-2.112992	-1.385320
C	1.492920	-2.217172	-1.610702
C	1.147239	-3.601109	-2.181778
C	1.880797	-3.797809	-3.517498
C	3.452087	-3.202378	-0.396487
C	3.840492	-4.782634	-2.303278
C	1.595346	-4.686807	-1.191225
C	3.111704	-4.588878	-0.964703
H	3.918097	-3.838197	-4.250916
H	4.820897	-2.220449	-2.579961
H	3.443318	-1.530211	-3.432570
H	3.253110	-1.125745	-0.977781
H	0.960330	-2.055906	-0.665610
H	1.159708	-1.432006	-2.300150
H	0.065957	-3.672919	-2.342156
H	1.626824	-4.774953	-3.946046
H	1.554176	-3.040869	-4.240919
H	4.530231	-3.126702	-0.210168
H	2.953054	-3.058687	0.569511
H	3.622030	-5.777291	-2.710623
H	4.925544	-4.734086	-2.151411
H	1.064342	-4.569322	-0.238833
H	1.335723	-5.679669	-1.578245
H	3.429257	-5.363316	-0.257840
C	-3.616323	8.597342	-0.071876
C	-4.029020	8.609780	1.408026
C	-3.410158	9.828851	2.109333
C	-1.879231	9.753745	2.004130

C	-1.460695	9.744143	0.525738
C	-2.084863	8.525520	-0.171626
C	-3.904182	11.113765	1.426893
C	-4.110349	9.885373	-0.748531
C	-1.960032	11.029000	-0.152893
C	-3.491091	11.107411	-0.053117
H	-4.058335	7.727444	-0.569884
H	-5.121907	8.640303	1.494032
H	-3.698108	7.685994	1.897994
H	-3.706162	9.836011	3.164140
H	-1.424562	10.608878	2.518796
H	-1.511069	8.850171	2.504994
H	-0.368976	9.690014	0.453157
H	-1.777532	8.495804	-1.224066
H	-1.720045	7.600106	0.290429
H	-4.994853	11.190834	1.512527
H	-3.484190	11.992547	1.931059
H	-3.839667	9.879435	-1.811401
H	-5.204666	9.939740	-0.700869
H	-1.506557	11.906628	0.323497
H	-1.649924	11.043955	-1.204832
H	-3.845217	12.024316	-0.536986
C	8.040023	-1.533590	6.459700
C	6.518863	-1.644498	6.274346
C	6.161167	-3.040831	5.742123
C	6.860306	-3.265668	4.392641
C	8.382484	-3.158886	4.572861
C	8.734478	-1.762396	5.108456
C	6.636021	-4.104474	6.744105
C	8.509670	-2.600974	7.460235
C	8.851738	-4.221864	5.578153
C	8.157642	-3.999542	6.930577
H	8.292877	-0.537418	6.838931
H	6.008973	-1.462906	7.228164
H	6.168494	-0.874786	5.575964
H	5.076164	-3.117849	5.611270
H	6.596662	-4.252121	3.992319
H	6.515345	-2.524495	3.661532
H	8.878786	-3.318965	3.609477
H	9.821019	-1.666060	5.222966
H	8.423077	-0.994542	4.389841
H	6.128930	-3.968501	7.707020
H	6.369113	-5.105564	6.384368
H	9.592108	-2.519007	7.617100
H	8.035223	-2.437522	8.435397
H	8.623521	-5.225204	5.198621
H	9.940435	-4.169546	5.700489
H	8.493003	-4.758610	7.645837
C	8.438297	5.898593	-1.703610
C	6.921499	5.790338	-1.923128
C	6.187320	6.007810	-0.590972
C	6.637234	4.940409	0.418325
C	8.153408	5.046201	0.644004
C	8.882554	4.831592	-0.691374

C	6.529400	7.401887	-0.042956
C	8.774309	7.293232	-1.153134
C	8.490663	6.442839	1.188426
C	8.045413	7.514038	0.181186
H	8.959764	5.743035	-2.654391
H	6.593528	6.533696	-2.659731
H	6.670872	4.804808	-2.334065
H	5.106082	5.930756	-0.749946
H	6.104888	5.072812	1.368027
H	6.381072	3.940265	0.048261
H	8.471988	4.283982	1.363432
H	9.967621	4.884950	-0.540554
H	8.666503	3.829332	-1.080957
H	6.194812	8.175468	-0.744710
H	5.995742	7.576852	0.899079
H	9.857543	7.390567	-1.011261
H	8.479098	8.064093	-1.875202
H	7.991279	6.601648	2.151865
H	9.568697	6.524528	1.373179
H	8.286170	8.509090	0.571260
C	-7.185883	-6.404231	-1.959678
C	-6.839172	-5.023254	-1.382376
C	-7.573287	-4.819156	-0.048070
C	-9.088968	-4.911909	-0.281644
C	-9.441765	-6.292586	-0.856248
C	-8.702563	-6.493093	-2.188285
C	-7.143597	-5.912945	0.941770
C	-6.756663	-7.493752	-0.964862
C	-9.006940	-7.382727	0.135344
C	-7.491131	-7.296228	0.370052
H	-6.661735	-6.547601	-2.910901
H	-5.755985	-4.938525	-1.233182
H	-7.123888	-4.237156	-2.092263
H	-7.324199	-3.834216	0.361973
H	-9.625388	-4.747850	0.660797
H	-9.412718	-4.123494	-0.971893
H	-10.522675	-6.356246	-1.022466
H	-8.961426	-7.467832	-2.619376
H	-9.019750	-5.732912	-2.912506
H	-6.065935	-5.845426	1.133917
H	-7.646702	-5.766733	1.905274
H	-6.980257	-8.486290	-1.374583
H	-5.672108	-7.453371	-0.807164
H	-9.542134	-7.262468	1.085040
H	-9.271788	-8.373192	-0.254270
H	-7.183053	-8.073820	1.077645
C	-2.522022	4.800422	1.610949
C	-4.037634	4.697211	1.381050
C	-4.382474	3.314893	0.805519
C	-3.646619	3.121251	-0.529197
C	-2.129854	3.220458	-0.304501
C	-1.790807	4.602863	0.274246
C	-3.937020	2.226367	1.794191
C	-2.081738	3.708202	2.597940

```
C     -1.690088      2.132528      0.687340
C     -2.420987      2.323575      2.025075
H     -2.278327      5.786611      2.021012
H     -4.573437      4.856781      2.324546
H     -4.367464      5.484299      0.692097
H     -5.463554      3.243332      0.642944
H     -3.900505      2.145309     -0.960400
H     -3.970179      3.880587     -1.251397
H     -1.607339      3.082654     -1.257410
H     -0.707710      4.694129      0.421000
H     -2.081127      5.388502     -0.433896
H     -4.470341      2.340106      2.745752
H     -4.195311      1.234819      1.403101
H     -1.003934      3.784087      2.786560
H     -2.582727      3.849046      3.563306
H     -1.909158      1.139129      0.277483
H     -0.605115      2.179595      0.840909
H     -2.106610      1.546751      2.730739
```

Adamantane$_{40}$

```
        1040
E=-1985.7104
C     -8.454582      0.183081      0.427105
C     -9.064998     -1.025842     -0.298486
C     -8.611391     -1.033551     -1.766524
C     -9.074828      0.262414     -2.449274
C     -8.464604      1.474920     -1.729299
C     -8.918251      1.476003     -0.261257
C     -7.078396     -1.118884     -1.825129
C     -6.921961      0.094091      0.363053
C     -6.931989      1.383071     -1.787519
C     -6.463103      0.089976     -1.103196
H     -8.779362      0.186817      1.473343
H     -8.758390     -1.955778      0.195417
H    -10.159588     -0.983560     -0.243369
H     -9.047301     -1.896578     -2.281746
H     -8.774167      0.260034     -3.504004
H    -10.169556      0.326653     -2.432008
H     -8.796775      2.397680     -2.217382
H     -8.505212      2.348354      0.259674
H    -10.010227      1.562144     -0.205170
H     -6.734466     -2.050206     -1.359095
H     -6.742946     -1.144580     -2.868990
H     -6.473478      0.941493      0.895754
H     -6.576310     -0.816005      0.868036
H     -6.593484      1.400779     -2.830552
H     -6.483756      2.254105     -1.294212
H     -5.370260      0.025958     -1.146153
C      2.138900    -11.095846     -2.239535
C      1.675626    -11.034877     -0.775894
C      2.317437     -9.825665     -0.078157
C      3.846926     -9.962493     -0.127021
C      4.316091    -10.020823     -1.589002
```

C	3.668860	-11.229297	-2.282998
C	1.898812	-8.537825	-0.804112
C	1.720194	-9.804766	-2.959773
C	3.892024	-8.732751	-2.311340
C	2.362723	-8.592734	-2.267971
H	1.680428	-11.958641	-2.735012
H	0.582470	-10.960030	-0.729641
H	1.952388	-11.959839	-0.255508
H	1.985319	-9.783840	0.965028
H	4.316871	-9.114575	0.385856
H	4.161306	-10.868471	0.405167
H	5.406676	-10.118914	-1.621397
H	4.011314	-11.294042	-3.322950
H	3.980391	-12.157815	-1.789256
H	0.809726	-8.416571	-0.759109
H	2.335431	-7.665164	-0.303452
H	2.027355	-9.844563	-4.011945
H	0.627892	-9.706884	-2.952374
H	4.363004	-7.863154	-1.837212
H	4.239002	-8.752967	-3.351610
H	2.062296	-7.673610	-2.782952
C	-8.327991	-4.753575	-7.232146
C	-8.785930	-3.446738	-7.897767
C	-8.174214	-2.246013	-7.159515
C	-8.632238	-2.262927	-5.693099
C	-8.174075	-3.566764	-5.021531
C	-8.785458	-4.764131	-5.765486
C	-6.641691	-2.341598	-7.214552
C	-6.795037	-4.842612	-7.287549
C	-6.641605	-3.659328	-5.082367
C	-6.178507	-3.645881	-6.547156
H	-8.765343	-5.608229	-7.759783
H	-8.483446	-3.434809	-8.951851
H	-9.880588	-3.380366	-7.881278
H	-8.501576	-1.315427	-7.636098
H	-8.217966	-1.398744	-5.159787
H	-9.724117	-2.175365	-5.638427
H	-8.502162	-3.576876	-3.976397
H	-8.481946	-5.701495	-5.283620
H	-9.880102	-4.721130	-5.711778
H	-6.299032	-2.309781	-8.255901
H	-6.192775	-1.479160	-6.707099
H	-6.456236	-5.781745	-6.833413
H	-6.456142	-4.854999	-8.330504
H	-6.192195	-2.819964	-4.538018
H	-6.300060	-4.576788	-4.587818
H	-5.085692	-3.711401	-6.588530
C	-2.804567	8.551791	7.105197
C	-2.084748	8.395056	5.756910
C	-2.393666	7.012844	5.161080
C	-1.912155	5.921840	6.129742
C	-2.631366	6.072045	7.479216
C	-2.322176	7.456811	8.068938
C	-3.909856	6.875336	4.953596

C	-4.319729	8.410705	6.892298
C	-4.146986	5.937425	7.265893
C	-4.634170	7.028359	6.300097
H	-2.582850	9.537653	7.528315
H	-2.404848	9.183558	5.065150
H	-1.002787	8.512617	5.892892
H	-1.879923	6.903508	4.199623
H	-2.109108	4.929475	5.706328
H	-0.827165	5.996148	6.271624
H	-2.286149	5.293598	8.168341
H	-2.814048	7.569553	9.042753
H	-1.244448	7.558213	8.245569
H	-4.264336	7.635894	4.247386
H	-4.141645	5.899290	4.510380
H	-4.847120	8.541173	7.845101
H	-4.680435	9.198813	6.220334
H	-4.383418	4.945230	6.862821
H	-4.671147	6.022584	8.225563
H	-5.714807	6.930471	6.148799
C	-8.660887	-10.140570	4.523575
C	-9.163739	-10.076682	5.973968
C	-8.596053	-8.827607	6.665703
C	-7.061152	-8.894346	6.657194
C	-6.552580	-8.955298	5.208558
C	-7.125808	-10.203823	4.520415
C	-9.054542	-7.572797	5.906736
C	-9.119750	-8.882432	3.770262
C	-7.016606	-7.700448	4.453068
C	-8.551324	-7.630607	4.456032
H	-9.066529	-11.031792	4.032366
H	-10.260002	-10.051711	5.991782
H	-8.858287	-10.979042	6.517473
H	-8.956303	-8.783789	7.699428
H	-6.643815	-8.017162	7.166345
H	-6.719305	-9.775810	7.213089
H	-5.458174	-9.003372	5.204947
H	-6.754269	-10.270003	3.490588
H	-6.784791	-11.108573	5.038213
H	-10.148833	-7.501310	5.922574
H	-8.671306	-6.672646	6.402408
H	-8.784287	-8.925530	2.726900
H	-10.215221	-8.835064	3.748851
H	-6.598239	-6.802210	4.923127
H	-6.642581	-7.721948	3.422239
H	-8.880280	-6.735082	3.917448
C	1.778483	-2.344422	10.810459
C	1.135983	-3.559775	10.124387
C	1.565652	-3.610934	8.650033
C	1.113356	-2.324030	7.943100
C	1.755634	-1.105228	8.623474
C	1.325812	-1.060692	10.097996
C	3.095831	-3.724298	8.569457
C	3.308137	-2.461567	10.724534
C	3.285328	-1.225090	8.543449

C	3.743147	-2.509182	9.251797
H	1.470733	-2.309693	11.861265
H	1.434668	-4.482662	10.636049
H	0.043301	-3.497148	10.195643
H	1.106882	-4.478482	8.163024
H	1.396880	-2.357190	6.884152
H	0.020298	-2.240371	7.975987
H	1.431364	-0.188929	8.118209
H	1.761967	-0.183288	10.590842
H	0.236504	-0.954247	10.169106
H	3.431355	-4.649894	9.052708
H	3.413913	-3.781105	7.521397
H	3.779493	-1.609433	11.229200
H	3.646371	-3.365033	11.246171
H	3.607216	-1.238546	7.495105
H	3.756200	-0.350284	9.008162
H	4.833909	-2.593210	9.193255
C	7.714704	2.244892	11.840743
C	8.189195	1.205567	12.867995
C	7.879777	-0.209587	12.355597
C	6.365181	-0.353698	12.141871
C	5.885673	0.681647	11.112775
C	6.200548	2.094511	11.628153
C	8.605509	-0.437538	11.020628
C	8.441376	2.010623	10.507322
C	6.616754	0.451830	9.781069
C	8.131934	0.597892	9.988774
H	7.936007	3.252972	12.208043
H	9.265979	1.315128	13.044812
H	7.691073	1.374245	13.830479
H	8.219414	-0.948948	13.089272
H	6.129141	-1.366972	11.794858
H	5.835565	-0.212711	13.091863
H	4.805591	0.577425	10.962407
H	5.845131	2.844577	10.911184
H	5.667693	2.279065	12.568889
H	9.690099	-0.357965	11.162638
H	8.407882	-1.452019	10.653741
H	8.126329	2.759697	9.770687
H	9.522878	2.133748	10.641306
H	6.384915	-0.547107	9.392232
H	6.268236	1.172362	9.031181
H	8.651511	0.433185	9.038426
C	4.397052	-0.973267	-7.840563
C	3.755328	-2.173801	-8.552948
C	2.224553	-2.053237	-8.496846
C	1.769715	-2.024005	-7.029632
C	2.406310	-0.823131	-6.313005
C	3.936620	-0.946619	-6.375036
C	1.790739	-0.752186	-9.189567
C	3.957793	0.324837	-8.535490
C	1.972490	0.474816	-7.011481
C	2.427738	0.451926	-8.478611
H	5.488238	-1.061282	-7.881509

H	4.091837	-2.216858	-9.595848
H	4.076574	-3.108421	-8.077303
H	1.769148	-2.909885	-9.005812
H	0.676247	-1.958219	-6.976184
H	2.055958	-2.956269	-6.527834
H	2.081100	-0.804611	-5.267087
H	4.403757	-0.104964	-5.849330
H	4.261342	-1.859630	-5.861280
H	2.091151	-0.768225	-10.244239
H	0.697684	-0.663912	-9.173079
H	4.425939	1.189015	-8.048631
H	4.297009	0.326875	-9.578413
H	0.882633	0.584863	-6.957448
H	2.404286	1.341692	-6.496659
H	2.117109	1.377810	-8.975098
C	-7.431406	-2.647835	3.462544
C	-7.092343	-1.275333	4.064089
C	-7.814292	-1.105639	5.409803
C	-9.331363	-1.209035	5.190137
C	-9.676486	-2.581355	4.591359
C	-8.949463	-2.747454	3.247933
C	-7.363310	-2.214747	6.372759
C	-6.980884	-3.752783	4.430566
C	-9.220387	-3.686799	5.556087
C	-7.703114	-3.589676	5.776758
H	-6.915953	-2.766672	2.503223
H	-6.008533	-1.182644	4.203721
H	-7.392348	-0.478011	3.373272
H	-7.570676	-0.126690	5.837117
H	-9.859385	-1.069463	6.141225
H	-9.670213	-0.410189	4.519406
H	-10.758409	-2.652614	4.435110
H	-9.203176	-3.715879	2.799884
H	-9.281830	-1.976082	2.542598
H	-6.284398	-2.140186	6.555042
H	-7.857621	-2.093103	7.344197
H	-7.198930	-4.739076	4.003154
H	-5.895176	-3.704588	4.577787
H	-9.746672	-3.591149	6.513528
H	-9.479491	-4.671844	5.149248
H	-7.379858	-4.378212	6.465211
C	9.064119	3.418083	5.398466
C	8.592976	4.702993	4.700173
C	7.061575	4.803282	4.775706
C	6.436743	3.583345	4.081598
C	6.902284	2.295299	4.778101
C	8.434118	2.201613	4.702923
C	6.625217	4.828899	6.248705
C	8.622825	3.447772	6.869970
C	6.466805	2.327395	6.251214
C	7.091605	3.544126	6.950811
H	10.156004	3.348423	5.343284
H	9.051700	5.579153	5.174145
H	8.918541	4.702272	3.652815

H	6.727942	5.720352	4.277752
H	5.342631	3.653303	4.111272
H	6.724399	3.563630	3.023429
H	6.456058	1.426913	4.281275
H	8.778423	1.275773	5.179621
H	8.757194	2.157015	3.655731
H	7.047733	5.707001	6.751949
H	5.534334	4.919811	6.316096
H	8.971169	2.544418	7.385338
H	9.081189	4.301698	7.383266
H	5.373213	2.374876	6.319190
H	6.775670	1.403499	6.755182
H	6.779596	3.565651	8.000781
C	-2.289298	-7.984446	2.389285
C	-1.578827	-8.196679	1.043636
C	-1.947544	-9.575114	0.473621
C	-1.511797	-10.667737	1.461926
C	-2.221938	-10.462031	2.808882
C	-1.852849	-9.081169	3.372742
C	-3.468535	-9.651309	0.269728
C	-3.809423	-8.064360	2.180043
C	-3.742245	-10.535475	2.599097
C	-4.183659	-9.442626	1.613663
H	-2.024933	-7.001372	2.793990
H	-1.865865	-7.408049	0.337659
H	-0.492625	-8.123114	1.176699
H	-1.440363	-9.724047	-0.486000
H	-1.751699	-11.658374	1.057028
H	-0.424413	-10.637399	1.601683
H	-1.909379	-11.241742	3.512022
H	-2.338015	-8.929544	4.344642
H	-0.771509	-9.022817	3.546748
H	-3.791140	-8.889386	-0.450157
H	-3.742571	-10.624518	-0.155246
H	-4.329347	-7.893892	3.130631
H	-4.137018	-7.274019	1.493894
H	-4.021553	-11.523876	2.214562
H	-4.260873	-10.410279	3.557385
H	-5.267717	-9.496877	1.464923
C	-1.548780	-4.296482	-11.187317
C	-1.175821	-2.917627	-10.621417
C	-1.913618	-2.684411	-9.293931
C	-3.429472	-2.746720	-9.536320
C	-3.808527	-4.125118	-10.099564
C	-3.065580	-4.354832	-11.424784
C	-1.513889	-3.777297	-8.290632
C	-1.149459	-5.385042	-10.179088
C	-3.403595	-5.214450	-9.094516
C	-1.887756	-5.158384	-8.850931
H	-1.021961	-4.460629	-12.133693
H	-0.092000	-2.854837	-10.466104
H	-1.439019	-2.132685	-11.340823
H	-1.645799	-1.701040	-8.892009
H	-3.968057	-2.561714	-8.599007

H	-3.731767	-1.958422	-10.236362
H	-4.889509	-4.167065	-10.272092
H	-3.342820	-5.328003	-11.847916
H	-3.361714	-3.595239	-12.158475
H	-0.436232	-3.731164	-8.092236
H	-2.019686	-3.610641	-7.331868
H	-1.391958	-6.376557	-10.580449
H	-0.065285	-5.366532	-10.014826
H	-3.941949	-5.073185	-8.149510
H	-3.687418	-6.202800	-9.476057
H	-1.601003	-5.935352	-8.133754
C	-6.739192	3.865926	4.337331
C	-7.240161	5.145004	3.649163
C	-8.773990	5.204880	3.717985
C	-9.363669	3.976050	3.008969
C	-8.868211	2.693712	3.695289
C	-7.334121	2.640444	3.626889
C	-9.217874	5.204740	5.188957
C	-7.188109	3.869745	5.806823
C	-9.311384	2.700070	5.166442
C	-8.721680	3.925640	5.880915
H	-5.645617	3.825093	4.286952
H	-6.806570	6.028048	4.133893
H	-6.909704	5.162986	2.603493
H	-9.128915	6.117850	3.227271
H	-10.459357	4.017374	3.033833
H	-9.070565	3.974171	1.952114
H	-9.289400	1.818984	3.187845
H	-6.968201	1.719191	4.096316
H	-7.005015	2.614499	2.580947
H	-8.820668	6.088502	5.702794
H	-10.311056	5.266724	5.251754
H	-6.818917	2.970694	6.315157
H	-6.754489	4.730169	6.330657
H	-10.406151	2.718563	5.229390
H	-8.981072	1.779576	5.662980
H	-9.039138	3.928779	6.929467
C	3.671389	-7.490941	-8.760427
C	4.051841	-6.113042	-8.197192
C	3.322113	-5.876924	-6.865765
C	1.804756	-5.934512	-7.099774
C	1.418225	-7.311933	-7.660319
C	2.153126	-7.544565	-8.989510
C	3.723887	-6.970654	-5.864205
C	4.072795	-8.580343	-7.753936
C	1.825222	-8.402126	-6.657041
C	3.342551	-8.350780	-6.421833
H	4.192462	-7.657160	-9.709618
H	5.136695	-6.053635	-8.047878
H	3.787180	-5.327568	-8.915480
H	3.595266	-4.894241	-6.465754
H	1.271934	-5.747400	-6.159590
H	1.501117	-5.145549	-7.798485
H	0.336181	-7.350513	-7.826874

H	1.870468	-8.517027	-9.410684
H	1.855371	-6.784342	-9.721892
H	4.802762	-6.927866	-5.671767
H	3.223912	-6.801988	-4.902743
H	3.824939	-9.571249	-8.153522
H	5.157912	-8.565212	-7.595656
H	1.292534	-8.258752	-5.709146
H	1.536162	-9.389728	-7.036581
H	3.630779	-9.128355	-5.705906
C	-3.379888	-4.190641	0.716866
C	-3.821233	-4.166597	2.188334
C	-3.193063	-2.957782	2.899054
C	-1.662081	-3.062333	2.824142
C	-1.214875	-3.083649	1.354292
C	-1.848419	-4.291906	0.647410
C	-3.648948	-1.665409	2.204266
C	-3.835830	-2.895138	0.027856
C	-1.676148	-1.791222	0.663209
C	-3.207092	-1.683378	0.732685
H	-3.828590	-5.053208	0.212133
H	-4.914814	-4.115023	2.252763
H	-3.517718	-5.095285	2.686702
H	-3.509518	-2.942314	3.947817
H	-1.201337	-2.214728	3.345811
H	-1.321158	-3.971495	3.334093
H	-0.123176	-3.158770	1.303314
H	-1.521119	-4.330136	-0.398715
H	-1.510493	-5.222939	1.118541
H	-4.739417	-1.567340	2.268267
H	-3.222210	-0.793525	2.714704
H	-3.544506	-2.908922	-1.029469
H	-4.929633	-2.819793	0.053869
H	-1.215416	-0.921201	1.146538
H	-1.345228	-1.784841	-0.382456
H	-3.534062	-0.761112	0.239973
C	7.175285	-1.470849	-0.623430
C	6.731302	-1.437012	0.847052
C	7.375270	-0.235650	1.556267
C	8.904747	-0.362304	1.484804
C	9.354627	-0.393493	0.015946
C	8.705286	-1.594212	-0.689444
C	6.939389	1.061481	0.857520
C	6.739381	-0.170573	-1.316407
C	8.913352	0.903777	-0.679108
C	7.383972	1.033712	-0.613088
H	6.715306	-2.328085	-1.127091
H	5.638441	-1.369621	0.909071
H	7.020446	-2.368776	1.348215
H	7.056911	-0.213175	2.604327
H	9.376519	0.479839	2.005460
H	9.231552	-1.275054	1.997583
H	10.445240	-0.484370	-0.032572
H	9.034158	-1.639595	-1.734791
H	9.028860	-2.528877	-0.215441

H	5.850308	1.175317	0.919010
H	7.377529	1.928362	1.366817
H	7.032653	-0.191023	-2.373086
H	5.646719	-0.079501	-1.292859
H	9.385512	1.768245	-0.196844
H	9.246482	0.902950	-1.724091
H	7.071270	1.959404	-1.108629
C	3.071288	6.981718	-7.553791
C	3.504868	5.889715	-6.563780
C	3.183995	4.504308	-7.145710
C	1.672534	4.396344	-7.399234
C	1.233915	5.484541	-8.391377
C	1.560137	6.867432	-7.806452
C	3.936320	4.323120	-8.473102
C	3.824414	6.794134	-8.879898
C	1.991481	5.301251	-9.715474
C	3.503697	5.411422	-9.467863
H	3.300699	7.968490	-7.136944
H	4.578970	5.972466	-6.358382
H	2.987635	6.024233	-5.606008
H	3.494462	3.727307	-6.438485
H	1.427841	3.403743	-7.796379
H	1.123648	4.504308	-6.455876
H	0.155998	5.406007	-8.570194
H	1.233691	7.654662	-8.496864
H	1.008945	7.019381	-6.870462
H	5.018530	4.377325	-8.303635
H	3.730462	3.329522	-8.889297
H	3.538756	7.580538	-9.589099
H	4.904439	6.892251	-8.716568
H	1.752185	4.324752	-10.153657
H	1.672200	6.060320	-10.439931
H	4.042165	5.279956	-10.412860
C	8.304901	3.806161	-5.227149
C	8.758133	2.738841	-4.219185
C	8.432481	1.339291	-4.763395
C	6.917471	1.220973	-4.989660
C	6.459179	2.284424	-5.999585
C	6.790237	3.681545	-5.452408
C	9.163762	1.129408	-6.098299
C	9.037025	3.589916	-6.560633
C	7.195762	2.072532	-7.331231
C	8.711422	2.192920	-7.110941
H	8.537754	4.803064	-4.837183
H	9.835146	2.829537	-4.033309
H	8.256068	2.894139	-3.256498
H	8.756975	0.579924	-4.043430
H	6.669678	0.218657	-5.359547
H	6.383613	1.349294	-4.040248
H	5.378770	2.198553	-6.158926
H	6.450004	4.451483	-6.155546
H	6.253806	3.853660	-4.511347
H	10.248393	1.190752	-5.947808
H	8.954491	0.125766	-6.487839

H	8.737271	4.358711	-7.283197
H	10.119226	3.695010	-6.417262
H	6.952643	1.085372	-7.742602
H	6.862233	2.813544	-8.067861
H	9.234915	2.041034	-8.061275
C	7.451946	-5.222803	-6.041237
C	6.995221	-5.193767	-4.574560
C	7.644528	-4.003217	-3.852102
C	9.173273	-4.144422	-3.910819
C	9.635906	-4.170879	-5.375803
C	8.981198	-5.360756	-6.094479
C	7.227567	-2.697548	-4.546482
C	7.034941	-3.914026	-6.729827
C	9.213519	-2.865041	-6.066536
C	7.684951	-2.720524	-6.013241
H	6.988136	-6.072335	-6.554352
H	5.902561	-5.116046	-4.521795
H	7.270784	-6.131423	-4.076747
H	7.317072	-3.984145	-2.806782
H	9.648576	-3.310204	-3.380701
H	9.486575	-5.063507	-3.400937
H	10.725969	-4.272146	-5.415235
H	9.318919	-5.402855	-7.137138
H	9.291402	-6.301483	-5.623538
H	6.139144	-2.573419	-4.493920
H	7.669584	-1.838194	-4.027864
H	7.337410	-3.930775	-7.783975
H	5.943053	-3.812388	-6.715380
H	9.689770	-2.008265	-5.574664
H	9.555929	-2.862632	-7.108512
H	7.385724	-1.788742	-6.505679
C	-3.112031	-7.929763	-4.739487
C	-1.580619	-8.039508	-4.797078
C	-0.946691	-6.840235	-4.075392
C	-1.394074	-5.539625	-4.760017
C	-2.925105	-5.423227	-4.702550
C	-3.553988	-6.626326	-5.422277
C	-1.406545	-6.827235	-2.609392
C	-3.566030	-7.914424	-3.271633
C	-3.380080	-5.414302	-3.235018
C	-2.937704	-6.714491	-2.546498
H	-3.561935	-8.785487	-5.254689
H	-1.252334	-8.976278	-4.330791
H	-1.245278	-8.070385	-5.840891
H	0.145115	-6.920840	-4.117200
H	-0.931861	-4.676407	-4.265982
H	-1.054920	-5.526919	-5.802931
H	-3.241806	-4.495590	-5.191670
H	-4.647658	-6.545223	-5.405746
H	-3.253428	-6.632686	-6.477076
H	-1.075703	-7.741683	-2.102277
H	-0.945118	-5.986013	-2.078107
H	-4.659971	-7.856991	-3.216931
H	-3.272965	-8.848445	-2.777186

H	-2.953172	-4.548707	-2.714141
H	-4.470606	-5.311060	-3.179713
H	-3.262469	-6.706097	-1.500216
C	2.810399	10.700191	-2.157764
C	3.232524	9.608919	-1.162014
C	2.905909	8.223538	-1.740802
C	1.395037	8.123993	-2.001196
C	0.967860	9.211483	-2.999090
C	1.299795	10.594326	-2.417273
C	3.663425	8.033223	-3.063957
C	3.568671	10.503481	-3.479610
C	1.730590	9.019046	-4.318918
C	3.242257	9.120772	-4.064441
H	3.043889	11.686947	-1.743151
H	4.306124	9.685772	-0.951754
H	2.711568	9.749865	-0.207189
H	3.208211	7.447054	-1.029484
H	1.146136	7.131568	-2.396161
H	0.842335	8.238531	-1.060847
H	-0.109655	9.138961	-3.182801
H	0.981496	11.381193	-3.111889
H	0.745092	10.752837	-1.484450
H	4.745128	8.081360	-2.889494
H	3.453442	7.039499	-3.477783
H	3.291243	11.289208	-4.192817
H	4.648489	10.595520	-3.311441
H	1.487379	8.042550	-4.754949
H	1.419444	9.777593	-5.047451
H	3.784405	8.982792	-5.006400
C	-7.095325	2.714417	-8.193354
C	-6.763019	4.080129	-7.572880
C	-7.521206	4.246172	-6.246782
C	-9.032237	4.155951	-6.509636
C	-9.370677	2.790450	-7.127512
C	-8.607455	2.627946	-8.451155
C	-7.105789	3.127074	-5.279395
C	-6.680479	1.599448	-7.220885
C	-8.950168	1.674964	-6.158183
C	-7.439009	1.758904	-5.894364
H	-6.554023	2.598176	-9.138653
H	-5.682897	4.163522	-7.402254
H	-7.037507	4.884436	-8.266205
H	-7.282362	5.220276	-5.805916
H	-9.585716	4.293079	-5.572772
H	-9.346108	4.961944	-7.183962
H	-10.448286	2.728584	-7.314543
H	-8.855731	1.664585	-8.912956
H	-8.914078	3.406653	-9.160082
H	-6.031898	3.192087	-5.066401
H	-7.626277	3.245877	-4.321370
H	-6.893816	0.617805	-7.661183
H	-5.599006	1.638284	-7.042934
H	-9.502418	1.768116	-5.215230
H	-9.205114	0.694745	-6.579060

H	-7.141148	0.963220	-5.202688
C	2.106390	2.914202	-0.873331
C	1.479143	1.703477	-1.581348
C	1.920412	1.676620	-3.052917
C	1.462930	2.969658	-3.745167
C	2.089971	4.183911	-3.042801
C	1.648602	4.204142	-1.571159
C	3.452040	1.576830	-3.123268
C	3.637565	2.810703	-0.949089
C	3.621163	4.077613	-3.112697
C	4.084133	2.787411	-2.418949
H	1.790386	2.931598	0.175454
H	1.781512	0.775916	-1.080414
H	0.385473	1.756219	-1.517652
H	1.472509	0.812336	-3.555592
H	1.754758	2.953749	-4.802257
H	0.369003	3.043968	-3.719543
H	1.762024	5.104586	-3.537629
H	2.073766	5.078015	-1.062683
H	0.557947	4.300725	-1.506967
H	3.791484	0.647261	-2.650496
H	3.778535	1.537405	-4.169538
H	4.098045	3.659429	-0.428878
H	3.979246	1.902742	-0.437603
H	3.951109	4.081568	-4.158612
H	4.081277	4.949568	-2.632119
H	5.175963	2.713076	-2.470248
C	-3.443930	0.783377	8.352881
C	-4.078978	-0.430109	7.656641
C	-3.656997	-0.460732	6.179500
C	-4.125029	0.829772	5.489577
C	-3.490266	2.046768	6.180159
C	-3.912337	2.070766	7.657395
C	-2.126307	-0.558984	6.089368
C	-1.913759	0.681400	8.257360
C	-1.959997	1.942009	6.090507
C	-1.486462	0.654352	6.781846
H	-3.746184	0.803455	9.405663
H	-3.768981	-1.355869	8.156243
H	-5.171781	-0.378427	7.734514
H	-4.110433	-1.326979	5.685234
H	-3.847059	0.811129	4.428803
H	-5.218613	0.902912	5.529322
H	-3.825750	2.965632	5.687017
H	-3.481574	2.946593	8.157731
H	-5.002171	2.166295	7.735597
H	-1.779605	-1.486792	6.560331
H	-1.813503	-0.601073	5.039027
H	-1.447499	1.532154	8.769040
H	-1.564339	-0.224686	8.766942
H	-1.643789	1.943306	5.040351
H	-1.494651	2.815877	6.562495
H	-0.395311	0.581110	6.716457
C	1.843035	6.674546	4.542826

C	1.219410	5.460543	3.837225
C	1.643909	5.443710	2.360583
C	1.165973	6.734923	1.678825
C	1.789291	7.952473	2.378792
C	1.364758	7.962680	3.855482
C	3.175517	5.359098	2.272222
C	3.374163	6.586242	4.449049
C	3.320532	7.861347	2.290865
C	3.803945	6.573018	2.974069
H	1.539000	6.684783	5.595236
H	1.536505	4.534024	4.330929
H	0.126088	5.502396	3.913706
H	1.198624	4.577074	1.859634
H	1.445723	6.725970	0.618393
H	0.071746	6.798489	1.717326
H	1.446758	8.871835	1.891460
H	1.787281	8.838660	4.362531
H	0.274032	8.048399	3.932599
H	3.529378	4.431040	2.737332
H	3.490284	5.326924	1.222118
H	3.832377	7.437375	4.967325
H	3.730498	5.679662	4.952931
H	3.638334	7.872584	1.241249
H	3.777698	8.735858	2.769608
H	4.895779	6.509506	2.909919
C	-7.370010	6.422663	-2.745950
C	-8.881532	6.324237	-3.003240
C	-9.645059	6.486000	-1.679679
C	-9.216927	5.377113	-0.706274
C	-7.706164	5.472820	-0.442946
C	-6.947600	5.314053	-1.769717
C	-9.315948	7.855786	-1.066356
C	-7.047021	7.792908	-2.130279
C	-7.381800	6.845316	0.166789
C	-7.805316	7.957910	-0.804301
H	-6.827597	6.306866	-3.690666
H	-9.193586	7.097904	-3.715134
H	-9.122469	5.356868	-3.460514
H	-10.722481	6.416058	-1.865572
H	-9.770181	5.469824	0.236155
H	-9.464291	4.393433	-1.123455
H	-7.403109	4.681030	0.250794
H	-5.866156	5.360661	-1.592877
H	-7.154431	4.329062	-2.205598
H	-9.635405	8.658192	-1.742320
H	-9.870368	7.991342	-0.129833
H	-5.967238	7.883829	-1.960576
H	-7.326659	8.593434	-2.825779
H	-7.902416	6.963822	1.124772
H	-6.308152	6.918811	0.378553
H	-7.573792	8.935669	-0.367686
C	-2.231682	1.063059	-3.832847
C	-3.747578	0.963826	-4.062622
C	-4.096354	-0.418379	-4.636048

C	-3.361032	-0.616161	-5.970451
C	-1.843993	-0.520943	-5.745872
C	-1.501010	0.861371	-5.169230
C	-3.654029	-1.506661	-3.645704
C	-1.794537	-0.028905	-2.844179
C	-1.407354	-1.608607	-4.752360
C	-2.137728	-1.413430	-3.414935
H	-1.985178	2.049174	-3.424285
H	-4.282940	1.126367	-3.119382
H	-4.075147	1.750800	-4.752784
H	-5.177632	-0.487100	-4.798538
H	-3.617695	-1.592032	-6.400168
H	-3.682409	0.142992	-6.693817
H	-1.321857	-0.661694	-6.698558
H	-0.417660	0.949767	-5.022591
H	-1.789072	1.646753	-5.878579
H	-4.187040	-1.389946	-2.694330
H	-3.915143	-2.498064	-4.035285
H	-0.716524	0.044190	-2.655651
H	-2.295139	0.114845	-1.879040
H	-1.629253	-2.602002	-5.160705
H	-0.322254	-1.564405	-4.598839
H	-1.825584	-2.190070	-2.708078
C	1.860376	-7.353137	3.206362
C	1.421141	-7.304245	4.677846
C	2.056631	-6.088040	5.369185
C	3.586938	-6.201828	5.293951
C	4.032051	-6.247998	3.824036
C	3.391195	-7.463559	3.136532
C	1.606636	-4.804053	4.655272
C	1.410348	-6.065931	2.498179
C	3.576674	-4.963873	3.113833
C	2.046415	-4.846875	3.183581
H	1.406454	-8.220938	2.715459
H	0.327934	-7.245903	4.742858
H	1.720422	-8.226793	5.189988
H	1.741671	-6.054852	6.417987
H	4.052847	-5.348792	5.802001
H	3.923746	-7.104874	5.817328
H	5.123269	-6.329669	3.772836
H	3.716884	-7.519522	2.090702
H	3.724838	-8.389050	3.621432
H	0.516786	-4.699244	4.719140
H	2.038652	-3.926733	5.151764
H	1.700175	-6.097434	1.440821
H	0.316994	-5.984408	2.524415
H	4.042636	-4.089010	3.583205
H	3.906218	-4.975243	2.067776
H	1.723651	-3.930537	2.677223
C	-2.996397	1.126896	-10.970862
C	-3.471117	1.133791	-9.509637
C	-2.858038	2.333128	-8.770259
C	-1.325857	2.227472	-8.811560
C	-0.845312	2.223277	-10.271000

C	-1.463849	1.024459	-11.006658
C	-3.296633	3.634377	-9.459587
C	-3.435177	2.431206	-11.654377
C	-1.289378	3.524542	-10.956747
C	-2.821393	3.633557	-10.920840
H	-3.434385	0.271065	-11.496056
H	-4.565828	1.186025	-9.469499
H	-3.179999	0.198768	-9.015740
H	-3.198281	2.336376	-7.728861
H	-0.876231	3.068123	-8.269342
H	-0.997584	1.311783	-8.304996
H	0.247186	2.147339	-10.298019
H	-1.112847	0.998405	-12.045447
H	-1.137712	0.087374	-10.539234
H	-4.388174	3.733104	-9.419245
H	-2.880723	4.499485	-8.929078
H	-3.119862	2.429779	-12.704887
H	-4.529210	2.507672	-11.652361
H	-0.838867	4.388070	-10.452587
H	-0.934721	3.543086	-11.994456
H	-3.136096	4.562123	-11.409694
C	8.282254	-5.264237	1.051240
C	6.762855	-5.375848	0.852360
C	6.409674	-6.775311	0.325369
C	7.121316	-7.008600	-1.016119
C	8.641779	-6.901147	-0.822338
C	8.989227	-5.501505	-0.292029
C	6.874783	-7.832936	1.338233
C	8.742170	-6.325609	2.062632
C	9.101258	-7.958085	0.193780
C	8.394631	-7.727294	1.538272
H	8.531892	-4.265834	1.426717
H	6.244137	-5.188278	1.800242
H	6.419288	-4.610326	0.146039
H	5.325915	-6.852822	0.184855
H	6.861089	-7.997411	-1.412846
H	6.783453	-6.271818	-1.754940
H	9.147007	-7.067259	-1.780044
H	10.074683	-5.404776	-0.167961
H	8.684815	-4.737975	-1.018207
H	6.358763	-7.690929	2.295517
H	6.610909	-8.836134	0.982148
H	9.823122	-6.242990	2.229102
H	8.258687	-6.156059	3.032299
H	8.876258	-8.963664	-0.181724
H	10.188783	-7.905326	0.325963
H	8.723037	-8.482065	2.261265
C	2.739057	1.637832	4.052436
C	1.222485	1.531679	3.830343
C	0.877120	0.147891	3.258674
C	1.606568	-0.046547	1.920561
C	3.124274	0.055596	2.137452
C	3.463862	1.439467	2.712367
C	1.329420	-0.938351	4.246751

C	3.186185	0.547885	5.038864
C	3.570901	-1.030054	3.128726
C	2.846428	-0.838198	4.469836
H	2.983124	2.625066	4.459757
H	0.691172	1.691785	4.776285
H	0.887836	2.317142	3.141859
H	-0.204641	0.074233	3.101663
H	1.352187	-1.023584	1.492138
H	1.278069	0.711123	1.198842
H	3.642214	-0.082781	1.182131
H	4.547527	1.532808	2.853516
H	3.168629	2.223518	2.004496
H	0.800708	-0.824060	5.200814
H	1.070863	-1.930939	3.858486
H	4.264794	0.625901	5.221929
H	2.689827	0.689359	6.006528
H	3.351473	-2.024456	2.721498
H	4.656552	-0.980898	3.276748
H	3.165691	-1.613398	5.175093
C	-2.188789	-5.858713	5.923719
C	-1.855288	-4.474772	6.501704
C	-2.581495	-4.283262	7.842189
C	-4.097645	-4.394894	7.620204
C	-4.437215	-5.778621	7.044966
C	-3.705949	-5.966481	5.706719
C	-2.130102	-5.373787	8.825952
C	-1.737881	-6.944989	6.912472
C	-3.980720	-6.865436	8.030450
C	-2.464353	-6.760058	8.253552
H	-1.670306	-5.993100	4.968091
H	-0.772140	-4.376510	6.642661
H	-2.155592	-3.690715	5.795996
H	-2.341842	-3.296181	8.252704
H	-4.628733	-4.239958	8.567198
H	-4.436856	-3.609067	6.934444
H	-5.518489	-5.855746	6.887019
H	-3.955672	-6.943438	5.275257
H	-4.038501	-5.208720	4.986867
H	-1.051922	-5.292906	9.009861
H	-2.627485	-5.236318	9.793705
H	-1.951944	-7.939342	6.502062
H	-0.652732	-6.891074	7.061814
H	-4.509964	-6.754297	8.984581
H	-4.235902	-7.858293	7.640472
H	-2.140816	-7.535306	8.956805
C	-3.135630	6.087678	-3.323275
C	-3.752524	4.876741	-4.039973
C	-3.317851	4.868865	-5.513727
C	-3.793064	6.163185	-6.191486
C	-3.176443	7.377700	-5.480404
C	-3.611141	7.378949	-4.006639
C	-1.785543	4.787041	-5.592011
C	-1.603759	6.002191	-3.406998
C	-1.644496	7.289359	-5.558292

C	-1.163820	5.997924	-4.879034
H	-3.446913	6.091529	-2.272942
H	-3.437412	3.947928	-3.549317
H	-4.846408	4.916528	-3.970791
H	-3.758350	4.004402	-6.022599
H	-3.506011	6.160626	-7.250000
H	-4.887626	6.224897	-6.160167
H	-3.517025	8.299281	-5.964903
H	-3.193460	8.252688	-3.491772
H	-4.702502	7.462602	-3.936556
H	-1.433468	3.856906	-5.129703
H	-1.463510	4.761257	-6.640087
H	-1.150427	6.851073	-2.880798
H	-1.249510	5.093319	-2.905782
H	-1.319498	7.306986	-6.605614
H	-1.191976	8.161840	-5.071499
H	-0.071474	5.936406	-4.936017
C	0.630660	3.857255	8.703080
C	1.117801	2.788017	9.693033
C	2.631042	2.936459	9.914442
C	3.360920	2.764812	8.573448
C	2.879631	3.833900	7.580312
C	1.365689	3.683432	7.365174
C	2.928069	4.333996	10.479645
C	0.932591	5.252328	9.271899
C	3.175541	5.229196	8.151556
C	2.445302	5.407096	9.491462
H	-0.448220	3.749528	8.546482
H	0.585952	2.887663	10.646940
H	0.890751	1.787021	9.306500
H	2.976067	2.173528	10.620969
H	4.444365	2.848800	8.721601
H	3.173276	1.763225	8.167900
H	3.400878	3.709269	6.624897
H	1.012328	4.428830	6.642344
H	1.142680	2.698231	6.937828
H	2.429472	4.462947	11.447947
H	4.004058	4.445553	10.660300
H	0.570824	6.025586	8.583282
H	0.398263	5.396522	10.218715
H	4.255734	5.356798	8.291976
H	2.854851	6.001975	7.442375
H	2.657873	6.402197	9.897463
C	3.397748	-3.697266	-3.294816
C	3.737250	-2.312869	-2.721433
C	3.009555	-2.112588	-1.383038
C	1.493267	-2.217319	-1.607431
C	1.147693	-3.601460	-2.178080
C	1.880460	-3.798108	-3.514241
C	3.453550	-3.201676	-0.394328
C	3.841270	-4.782083	-2.301133
C	1.596805	-4.686857	-1.187653
C	3.113275	-4.588377	-0.962119
H	3.917303	-3.837909	-4.248961

H	4.820623	-2.219605	-2.578825
H	3.442261	-1.529964	-3.430653
H	3.253491	-1.125198	-0.975802
H	0.961229	-2.056095	-0.662021
H	1.159345	-1.432369	-2.296782
H	0.066333	-3.673662	-2.337753
H	1.626545	-4.775402	-3.942480
H	1.553116	-3.041387	-4.237565
H	4.531787	-3.125604	-0.208712
H	2.955088	-3.058011	0.571969
H	3.622886	-5.776875	-2.708189
H	4.926403	-4.733142	-2.149970
H	1.066373	-4.569411	-0.234937
H	1.337273	-5.679865	-1.574359
H	3.431546	-5.362601	-0.255345
C	-3.615143	8.595675	-0.065737
C	-4.024920	8.606577	1.414988
C	-3.404365	9.824711	2.116426
C	-1.873667	9.749333	2.008122
C	-1.458048	9.741265	0.528898
C	-2.083902	8.523574	-0.168585
C	-3.899403	11.110507	1.436385
C	-4.110171	9.884582	-0.739988
C	-1.958392	11.027000	-0.147323
C	-3.489231	11.105690	-0.044442
H	-4.058359	7.726442	-0.563837
H	-5.117628	8.637282	1.503182
H	-3.693280	7.682164	1.903279
H	-3.698288	9.830777	3.171821
H	-1.417766	10.603779	2.522839
H	-1.504751	8.845110	2.507257
H	-0.366488	9.686938	0.454106
H	-1.778653	8.494948	-1.221661
H	-1.718411	7.597555	0.291724
H	-4.989883	11.187759	1.524254
H	-3.478193	11.988622	1.940696
H	-3.841586	9.879753	-1.803396
H	-5.204379	9.939174	-0.690110
H	-1.503754	11.903984	0.329146
H	-1.650353	11.043043	-1.199853
H	-3.844074	12.023221	-0.526595
C	8.000398	-1.500725	6.448531
C	6.484099	-1.626506	6.234989
C	6.147272	-3.034809	5.721002
C	6.874903	-3.278395	4.389962
C	8.392352	-3.156823	4.598467
C	8.723409	-1.748346	5.115709
C	6.609673	-4.076731	6.751232
C	8.457650	-2.546452	7.477278
C	8.849084	-4.198064	5.631883
C	8.126454	-3.956901	6.966009
H	8.238373	-0.496039	6.814706
H	5.953910	-1.431787	7.175051
H	6.142207	-0.872086	5.516022

H	5.065693	-3.122412	5.570005
H	6.626491	-4.273823	4.002315
H	6.539280	-2.553053	3.638902
H	8.908992	-3.330309	3.648186
H	9.806713	-1.641709	5.250051
H	8.420896	-0.995849	4.377324
H	6.082431	-3.927438	7.701266
H	6.357292	-5.086093	6.404353
H	9.536107	-2.453456	7.654139
H	7.962600	-2.369218	8.439709
H	8.635798	-5.209734	5.266026
H	9.934705	-4.135280	5.774884
H	8.452926	-4.700492	7.701358
C	8.448493	5.908694	-1.718364
C	6.932447	5.800655	-1.943122
C	6.193562	6.021424	-0.614113
C	6.639322	4.956056	0.399167
C	8.154721	5.061645	0.630089
C	8.888585	4.843735	-0.702166
C	6.534318	7.416554	-0.067957
C	8.783172	7.304395	-1.169770
C	8.490665	6.459331	1.172629
C	8.049564	7.528498	0.161414
H	8.973318	5.750784	-2.666907
H	6.607490	6.542530	-2.682552
H	6.682841	4.814333	-2.352776
H	5.112869	5.944519	-0.776823
H	6.103610	5.090813	1.346643
H	6.384027	3.955212	0.030397
H	8.470339	4.300876	1.352352
H	9.973126	4.896924	-0.547544
H	8.673470	3.840713	-1.090304
H	6.202632	8.188732	-0.772629
H	5.997342	7.593856	0.871752
H	9.865932	7.401541	-1.024200
H	8.490936	8.073788	-1.894608
H	7.987879	6.620508	2.133902
H	9.568064	6.540929	1.361095
H	8.289379	8.524302	0.550148
C	-7.184841	-6.402964	-1.961049
C	-6.837792	-5.022304	-1.383193
C	-7.572334	-4.818316	-0.049105
C	-9.087962	-4.910384	-0.283286
C	-9.441099	-6.290744	-0.858445
C	-8.701470	-6.491144	-2.190262
C	-7.143464	-5.912580	0.940565
C	-6.756441	-7.492963	-0.966401
C	-9.007093	-7.381362	0.132980
C	-7.491339	-7.295548	0.368293
H	-6.660388	-6.546257	-2.912115
H	-5.754628	-4.938058	-1.233561
H	-7.121919	-4.235875	-2.092950
H	-7.323004	-3.833601	0.361332
H	-9.624676	-4.746396	0.659000

H	-9.411130	-4.121628	-0.973418
H	-10.521971	-6.353916	-1.025095
H	-8.960562	-7.465647	-2.621748
H	-9.018072	-5.730614	-2.914372
H	-6.065847	-5.845555	1.133143
H	-7.646878	-5.766457	1.903922
H	-6.980280	-8.485285	-1.376510
H	-5.671930	-7.453068	-0.808277
H	-9.542600	-7.261176	1.082509
H	-9.272193	-8.371601	-0.257037
H	-7.183845	-8.073480	1.075766
C	-2.519224	4.798677	1.611941
C	-4.035069	4.696138	1.383280
C	-4.380918	3.314200	0.807443
C	-3.646266	3.120859	-0.527980
C	-2.129274	3.219396	-0.304524
C	-1.789216	4.601423	0.274535
C	-3.935039	2.225077	1.795265
C	-2.078518	3.705863	2.598086
C	-1.689080	2.130871	0.686473
C	-2.418774	2.321613	2.024908
H	-2.274809	5.784597	2.022225
H	-4.570013	4.855501	2.327299
H	-4.365185	5.483649	0.694947
H	-5.462162	3.243118	0.645751
H	-3.900885	2.145200	-0.959391
H	-3.970150	3.880631	-1.249576
H	-1.607618	3.081808	-1.257935
H	-0.705960	4.692215	0.420411
H	-2.079837	5.387477	-0.433022
H	-4.467511	2.338605	2.747327
H	-4.194035	1.233796	1.403965
H	-1.000526	3.781260	2.785826
H	-2.578637	3.846478	3.563936
H	-1.908872	1.137732	0.276370
H	-0.603960	2.177461	0.839143
H	-2.104094	1.544365	2.729969
